# Supplementary material for: Systemic Blood Proteome Patterns Reflect Disease Phenotypes in Neovascular Age-Related Macular Degeneration
Source: Int J Mol Sci. 2023 Jun 19;24(12):10327. doi: 10.3390/ijms241210327 (PMC10299094; doi:10.3390/ijms241210327)
Supplement: Supplementary file 1 [file ijms-24-10327-s001.zip › File S1.pdf]

Suppl. Methods:

Sample collection, preparation and mass spectrometry analysis

A blood draw (500 µl whole blood with EDTA as anticoagulant) was performed at date of study inclusion. Flash-freezing of whole-blood samples was performed in liquid nitrogen with subsequent -80°C storage following standard protocols. Sample collection and processing was performed by predetermined personal only, strictly following the study protocol. Logging of time of sample processing reveals no significant differences between any annotated meta-features or calculated clusters (not shown). After collection of all samples, they were cumulatively processed. All samples were maintained at -80°C until processing. Samples were then prepared using the automated MicroLab STAR® system from Hamilton Company. Several recovery standards were added prior to the first step in the extraction process for QC purposes. Samples were extracted with methanol under vigorous shaking for 2 minutes (Glen Mills GenoGrinder 2000) to precipitate protein and dissociate small molecules bound to protein or trapped in the precipitated protein matrix, followed by centrifugation to recover chemically diverse metabolites. The resulting extract is divided into five fractions: two for analysis by two separate reverse phase (RP)/UPLC-MS/MS methods using positive ion mode electrospray ionization (ESI), one for analysis by RP/UPLC-MS/MS using negative ion mode ESI, one for analysis by HILIC/UPLC-MS/MS using negative ion mode ESI, and one reserved for backup. Samples were placed briefly on a TurboVap® (Zymark) to remove the organic solvent. The sample extracts were stored overnight under nitrogen before preparation for further analysis.

Several types of quality control samples were analyzed in concert with the experimental samples. These include: 1) technical replicate samples derived from a pool of well-characterized human plasma (MTRX) or, alternatively, generated by

combining a small portion of each (non-plasma) experimental sample (CMTRX), spaced evenly among experimental samples; 2) extracted water samples (process blanks) and solvent blanks; and 3) a cocktail of QC standards, carefully chosen not to interfere with the measurement of endogenous compounds, spiked into every analyzed sample, allowing instrument performance monitoring and aiding with chromatographic alignment. Instrument variability was determined by calculating the median relative standard deviation (RSD) for the standards that were added to each sample prior to injection into the mass spectrometers. Overall process variability was determined by calculating the median RSD for all endogenous metabolites (i.e., non-instrument standards) present in each of the pooled MTRX (or CMTRX) technical replicate samples. Experimental samples were then randomized across the platform run, with QC samples spaced evenly among the injections. For ultrahigh performance liquid chromatography-tandem mass spectroscopy (UPLC-MS/MS), all methods utilized a Waters ACQUITY ultra-performance liquid chromatography (UPLC) and a Thermo Scientific Q-Exactive high resolution/accurate mass spectrometer interfaced with a heated electrospray ionization (HESI-II) source and Orbitrap mass analyzer operated at 35,000 mass resolution. The sample extract was dried then reconstituted in solvents compatible to each of the four methods. Each reconstitution solvent contained a series of standards at fixed concentrations to ensure injection and chromatographic consistency. One aliquot was analyzed using acidic positive ion conditions, chromatographically optimized for more hydrophilic compounds. In this method, the extract is gradient-eluted from a C18 column (Waters UPLC BEH C18-2.1x100 mm, 1.7 m) using water and methanol, containing 0.05% perfluoropentanoic acid (PFPA) and 0.1% formic acid (FA). A second aliquot was also analyzed using acidic positive ion conditions, but was chromatographically optimized for more hydrophobic compounds. In this method, the extract was gradient eluted from the aforementioned

C18 column using methanol, acetonitrile, water, 0.05% PFPA and 0.01% FA, and was operated at an overall higher organic content. A third aliquot was analyzed using basic negative ion optimized conditions using a separate dedicated C18 column. The basic extracts were gradient-eluted from the column using methanol and water, however with 6.5mM Ammonium Bicarbonate at pH 8. The fourth aliquot was analyzed via negative ionization following elution from a HILIC column (Waters UPLC BEH Amide 2.1x150 mm, 1.7 m) using a gradient consisting of water and acetonitrile with 10mM Ammonium Formate, pH 10.8. The MS analysis alternated between MS and data-dependent MSn scans using dynamic exclusion. The scan range varied slightly between methods, but covered approximately 70-1000 m/z.

Bioinformatics: Data extraction, compound identification, curation, metabolite quantification and block correction

The applied informatics system consisted of four major components, the Laboratory Information Management System (LIMS), the data extraction and peak-identification software, data processing tools for QC and compound identification, and a collection of statistical, visualization, and interpretation tools. The scope of the Metabolon LIMS system encompassed sample accessioning, sample preparation, instrumental analysis and reporting, and advanced data analysis. All the subsequent software systems are grounded in the LIMS data structures.

Raw data were extracted, peak-identified, and QC processed using Metabolon's hardware and software. Compounds were identified by comparison to library entries of purified standards or recurrent unknown entities. Metabolon maintains a library based on authenticated standards that contains the retention time/index (RI), mass to charge ratio (m/z), and chromatographic data (including MS/MS spectral data) on all molecules present in the library. Furthermore, biochemical identifications were based

on three criteria: retention index within a narrow RI window of the proposed identification, accurate mass match to the library +/- 10 ppm, and the MS/MS forward and reverse scores. MS/MS scores were based on a comparison of the ions present in the experimental spectrum to ions present in the library entry spectrum. While there might be similarities between these molecules based on one of these factors, the use of all three data points could be utilized to distinguish and differentiate biochemicals. More than 4500 commercially available purified standard compounds have already been acquired and registered into LIMS for analysis on all platforms for determination of their analytical characteristics. Additional mass spectral entries have been created for structurally unnamed biochemicals, which have been identified by virtue of their recurrent nature (both chromatographic and mass spectral). These compounds hold the potential to be identified by future acquisition of a matching purified standard or by classical structural analysis. A variety of curation procedures were performed to ensure that a high quality data set is made available for statistical analysis and data interpretation. The QC and curation processes were designed to ensure accurate and consistent identification of true chemical entities, and to remove those representing system artifacts, mis-assignments, redundancy, and background noise. Library matches for each compound were checked for each sample and corrected if necessary. Peaks were quantified as area-under-the-curve detector ion counts. A data adjustment step was performed to correct block variation resulting from instrument inter-day tuning differences, while preserving intra-day variance. Essentially, each compound was corrected in balanced run-day blocks by registering the daily medians to equal one (1.00), and adjusting each data point proportionately (termed the "block correction"). In total 899 biochemicals were quantified with this approach. We considered the subset of Erythritol, Xylitol, Sorbitol, Saccharin, Acesulfame, and Aspartate for further analysis.
