# Supplementary material for: Systemic Blood Proteome Patterns Reflect Disease Phenotypes in Neovascular Age-Related Macular Degeneration
Source: Int J Mol Sci. 2023 Jun 19;24(12):10327. doi: 10.3390/ijms241210327 (PMC10299094; doi:10.3390/ijms241210327)
Supplement: Supplementary file 1 [file ijms-24-10327-s001.zip › File S2.pdf]

CHARITÉ

ChariteCentrum für Audiologie / Phoniatrie, Augen und HNO-Heilkunde

STUDY PROTOCOL

Study title:

OCT Angiography and Analysis of systemic biomarkers as new technologies to investigate the different characteristics of patients with neovascular age-related macular degeneration in dependency of treatment demand and frequency

Acronym: **BIOMAC**

Study protocol code:

Sponsor

Charité – University Medicine Berlin

Charitéplatz 1

10117 Berlin

Germany

Principal Coordinating Investigator (PCI):

Prof. Dr. med. Oliver Zeitz

Charité, University Medicine Berlin

Department of Ophthalmology, CBF
Hindenburgdamm 30
12200 Berlin
Germany

Version of 20 August 2018, Version 1.3

The information in this study protocol is strictly confidential. It is for the use of the sponsor, investigator, study personnel, ethics committee, the authorities, and study subjects only. This study protocol may not be passed on to third parties without the express agreement of the sponsor or the Principal Coordinating Investigator (PCI).

Signatures

Protocol authorization signature page

Study code:

Title: **OCT Angiography and Analysis of systemic biomarkers as new technologies to investigate the different characteristics of patients with neovascular age-related macular degeneration in dependency of treatment demand and frequency**

EudraCT-No:

Approved by the following

Prof. Dr. med. Oliver Zeitz	______________________	__________
(Principal Coordinating Investigator, PCI)<br>Charité, Department of Ophthalmology, Berlin;<br>Acting on behalf of the sponsor	Signature	Date

Table of contents

Signatures 2

1. Synopsis 4
2. Abbreviations 9
3. Introduction 10

4. Objectives of the clinical study 11
4.1 Rationale of the clinical study 11
4.2 Primary objective/Hypothesis 11
4.3 Secondary and other objectives 12

5. Conduct of the study 12
5.1 General aspects of study design 12
5.2 Discussion of study design 12
5.3 Selection of study population 13
5.3.1 Inclusion criteria 13
5.3.2 Exclusion criteria 14
5.4 Withdrawal of study subject after study start 14
5.5 Examinations included in study 14
5.6 Description of visits 18
5.7 Outcome parameters 19
5.8 Data quality assurance 19

6. Rates of organisational and administrative aspects of the study 20
6.1 Sponsor 20
6.2 Principal Coordinating Investigator 20
6.3 Laboratory diagnostics 21
6.4 Imaging review board 21
6.5 Central organisation units 21
6.6 Investigators and study sites 21
6.7 Financing 21

7. Ethical and regulatory aspects 22
7.1 Independent ethics comittee 22
7.2 Ethical basis for the clinical study 22
7.2.1 Legislation and guidelines used for preparation 22
7.3 Obtaining informed consent from study subjects 22
7.4 Insurance of study subjects 23
7.5 Data protection 23

8. Statistical methods and sample size calculation 23
9. Use of study findings and publications 24
10. Amendmends to the study protocol 24
11. References 25

1. Synopsis

Sponsor:	Charité – University Medicine Berlin<br>Charitéplatz 1<br>10117 Berlin<br>Germany
Principal Coordinating Investigator:	Prof. Dr. med. Oliver Zeitz<br>Charité University Medicine Berlin<br>Department of Ophthalmology, CBF<br>Hindenburgdamm 30<br>12203 Berlin<br>Germany
Title of the clinical study:	OCT Angiography and Analysis of systemic biomarkers as new technologies to investigate the different characteristics of patients with neovascular age-related macular degeneration in dependency of treatment demand and frequency
Type of study, study design, methodology:	Single-center observational cohort study with bio-banking to support a multi-omics analysis
Study population:	Approximately 50 Patients diagnosed with neovascular AMD and treated with intravitreal anti-VEGF injections at the Charité Deptartment of Ophthalmology at Campus Benjamin Franklin will be enrolled into this study. As treatment is not part of the study, eligible subjects will be identified by a retrospective review of medical records.<br>The overall population will consist of two approximately equally sized cohorts:<br>• Approximately 25 patients requiring a high frequency of intravitreal anti-VEGF (≤ 6 weeks)<br>• Approximately 25 patients requiring a low frequency of intravitreal anti-VEGF (≥ 10 weeks) to supress CNV activity

Primary study objective:	The primary objective of the study is to support a systems medicine approach in order to explore ocular and systemic factors allowing to discriminate between subjects with nAMD requiring high or low treatment frequency.
Outcome parameters:	• Epidemiological: Age, Sex, Gender, Disease Duration<br>• Ophthalmic / functional: best corrected visual acuity (BCVA), intraocular pressure (IOP)<br>• Ophthalmic / morphological: central retinal thickness (CRT), subretinal fluid (SRF), intraretinal cysts (IRC), pigment epithelial detachment (PED), CNV lesion size (CNV), outer retinal tubulation (ORT), external limiting membrane (ELM) and ellipsoid Zone (EZ) abnormalities, hyperreflective foci (HRF), posterior vitreous detachment (PVD), vitreomacular adhesion (VMA), CNV blood flow, CNV pattern, CNV size, superficial and deep vessel density<br>• Systemic: genomic-, immunomic-, proteomic- and metabolomic parameters.
Medical condition:	Age-related macular degeneration

Principal inclusion criteria:	• Men and women ≥ 51 years of age<br>• Receiving intravitreal treatment for CNV secondary to neovascular AMD (all lesion types) in the study eye<br>• BCVA ≤ 0.8 and ≥ 0.05 in the study eye<br>• Cohort 1:<br>o The intervals between the current and the last as well as between the last and second last intravitreal injection in the study eye was ≤ 42 days (6 weeks)<br>o CNV regarded as active in the study eye as evidenced by residual fluid present on OCT at current and the last two visits before the injections<br>• Cohort 2:<br>o The intervals between the current and the last as well as between the last and second last intravitreal injection in the study eye was ≥ 70 days (10 weeks)<br>o CNV activity regarded as controlled in the study eye as evidenced by fluid absent or stable on OCT at current and the last two visits before injections
Principal exclusion criteria:	• Causes of CNV other than nAMD in the study eye<br>• subretinal hemorrhage in the study eye, which warrants surgical intervention except for intravitreal therapy with an VEGF inhibitor<br>• Any contraindication for continuous intravitreal therapy
Name of investigational medicinal product (IMP):	No data are collected on the use of specific medicinal products.

Duration of study:	Total follow-up is 3 month

Time plan:

First patient first visit (FPFV):	01 September 2018
Last patient first visit (LPFV):	31 July 2019
Last patient last visit (LPLV):	31 October 2019
Final study report:	31 July 2020

Statistical methods:	Statistical analysis will be of explorative and descriptive nature. Primary analysis is done at 5% significance level (one-sided).<br><br>All variables will be analyzed with appropriate statistical methods: continuous variables by sample statistics (i.e. mean, standard deviation, median, quartiles, minimum and maximum) and categorical variables by frequency tables (absolute and relative frequencies).<br>Descriptive analyses will be performed using boxplots and histograms for the visualization of value distributions. Furthermore, clustering methods and heatmaps will be used to illustrate sample and feature interrelations.<br>Explorative statistics will be applied for the identification of predictive biomarkers. These methods will either yield single biomarkers or biomarker signatures, including heterogeneous signatures (e.g. a combination of sequence and protein markers).<br>For the identification of single biomarkers, the Mann-Whitney-U-test will be applied for numerical data (e.g. protein expression), and the Fisher-test for categorical data (e.g. mutations).<br>For the identification of complex biomarkers, Logistic Regression will be applied. In order to assess the predictive power of the identified marker signatures, permutation and cross-validation will be used.
GCP conformance:	The present study will be conducted in accordance with the valid versions of the study protocol, ICH Good Clinical Practice Guidelines (ICH-GCP) and applicable local regulatory requirements.
Financing:	This investigator initiated study is financially supported by Novartis Pharma, Nürnberg, Germany. Sponsorship is taken over by Charité University Medicine Berlin.

2. Abbreviations

Abbreviation	Meaning
BCVA	Best corrected visual acuity
CNV	Choroidal neovascularisation
EDTA	Ethylenediaminetetraacetic acid
FAF	Fundus Autofluorescence
IMP	Investigational medicinal product
IOP	Intraocular pressure
nAMD	Neovascular Age-related Macular Degeneration
OCT	Optical coherence tomography
OCTA	Optical coherence tomography angiography
PBMC	Peripheral blood mononuclear cell
PCI	Principal Coordinating Investigator
PCR	Polymerase chain reaction
PRN	pro re nata (as needed = when necessary)
RPE	Retinal pigment epithelium
VEGF	Vascular endothelial growth factor

3. Introduction

Age-related macular degeneration (AMD) is a leading cause of adult blindness in the developed world and has a profound impact on the quality of life of affected individuals [1, 2]. Age-related macular degeneration is classified into 2 main types: nonexudative (also known as “dry“) and exudative (“wet” or “neovascular”), the latter of which accounts for most AMD-related cases of blindness. Neovascular AMD (nAMD) is characterized by the development of choroidal neovascularization (CNV) under the retina or RPE. CNV can result in leakage of fluid and hemorrhage in the intraretinal, subretinal, or sub-RPE space, consequently causing photoreceptor damage [3].

Vascular endothelial growth factor (VEGF), a protein growth factor that promotes angiogenesis and increases vascular permeability, is a major pathogenic factor in nAMD. Uncontrolled expression of VEGF leads to development of abnormal blood vessels and hence stimulates CNV formation. [4].

The administration of intravitreal Anti-vascular endothelial growth factor (anti-VEGF) to supress CNV activity is currently the treatment of choice for nAMD [5]. Three anti-VEGF agents have been approved for intraocular use in nAMD cases: (1) ranibizumab (Lucentis®), (2) aflibercept (Eylea®) and (3) pegaptanib (Macugen®), Bevacizumab (Avastin®) is used as off-label to treat nAMD.

Retinal imaging is essential to detect and evaluate CNV in clinical practice. Leakage of intravenously injected dye in the later phases of the fluorescein angiography (FA) enables to identify the presence of the CNV and is still critical for diagnosis of nAMD. Optical coherence tomography (OCT) is the primary method to control for structural changes such as exudation, neovascular membranes, fibrosis and pigment epithelial detachments over the course of the disease. In recent years, a new imaging modality known as optical coherence tomographic angiography (OCTA) has been developed to simultaneously obtain structural images of the retina as well as assess blood flow within the retinal and choroidal vasculature without the use of intravenous agents.

The treatment response to anti-VEGF and therefore CNV activity is commonly monitored by improvement of visual acuity and integrity of macular anatomy in OCT, i.e. absence of fluid. In case of inactive CNV treatment intervals are extended or treatment is ceased until CNV activity recurs. As of now, the treatment interval is adjusted empirically based on continuous monitoring of CNV activity. This individualisation results in a wide range of treatment frequencies within a patient population [6]. However, as there is a lack of significant factors, which help to predict the optimal treatment frequency for an individual patient, any a priori determination of treatment frequency is impossible. Hence, all patients have to undergo a quite extensive monitoring with

frequent office visits. Previous studies exploring predictive morphological parameters based on OCT [7-10] and more recently on OCT-A imaging [11-13] have yielded conflicting results. Waldstein et al. found patients with subretinal fluid and posterior vitreous detachment to have higher visual gains and reduced injection frequency [9]. In an analysis by Simader et al., presence of subretinal fluid at baseline had no significant effect on visual function, whereas intraretinal cysts were associated with worse visual outcomes [10]. Systemic, and in particular genetic factors, have been found to be associated with high significance with the risk to develop nAMD [14-17]. This may imply that systemic factors may also be associated with treatment demand. Hence the present study is set-up to support a systems medicine model for nAMD patients.

Predictive factors for patient response to therapy would enable an effective a-priori stratification. This would allow individual optimization of treatment regimens, including frequency and number of injections required. This would improve disease management by avoiding unnecessary visits, as well as the risk of injection-related adverse events. Overall, such approach could help to improve efficiency of health-care utilization.

4. Objectives of the clinical study

4.1. Rationale for the clinical study

Adjusting the treatment interval to maximize vision gains while minimizing number of injections is one of the major challenges in treating nAMD with VEGF-inhibitors. This issue has to be regarded as unresolved due to a lack of reliable stratification markers. Results from previous studies focussing merely on ocular morphological parameters have remained inconclusive with regards to stratification on an individual patient level. This study aims at taking a broader approach to search for new systemic biomarkers for effective stratification of nAMD subjects into those requiring frequent and those requiring infrequent treatments. To achieve this objective, an analysis of multiple types of omics data (Genomic, Immunomic, Proteomic, Metabolomic) is intended, offering the opportunity to understand the flow of information underlying varying treatment responses in nAMD.

4.2. Primary objective

The primary objective of the study is to support a systems medicine approach in order to explore ocular and systemic factors allowing to discriminate between subjects with nAMD requiring high or low treatment frequency.

4.3. Secondary objectives

The secondary objective of the study is to explore clinical and morphological discriminatory factors determining the treatment demand in a well characterised population with neovascular AMD using ophthalmic examinations (BCVA, IOP, slit lamp examination, funduscopy) and retinal imaging (optical coherence tomography angiography, optical coherence tomography, fundusautofluorescence, fluorescein angiography)

5. Conduct of the study

5.1. General aspects of study design

This is a single-center observational clinical cohort study.

Table 1: Time plan of the study

First patient first visit (FPFV):	01 September 2018
Last patient first visit (LPFV):	31 July 2019
Last patient last visit (LPLV):	31 October 2019
Final study report:	31 July 2020

<u>End of the clinical study</u>
The end of the study is defined as the last visit of the last patient included (LPLV).

5.2. Discussion of study design

The study will be conducted with a well characterised population of neovascular AMD allowing to discriminate between patients requiring a high treatment frequency and low treatment frequency of intravitreal Anti-VEGF and to determine potential predictive morphological and systemic biomarkers.

There will be a baseline and a follow-up visit after 3 month which will be integrated into routine clinical practice. Additionally to routine clinical examinations, OCT-Angiography at baseline and at a 3 month follow-up will be performed to prospectively assess the discriminatory potential. To explore potential systemic biomarkers, biobanking of venous blood samples (20ml) will be performed at baseline examination. The further analysis of the blood specimen via whole genome sequencing, deep immune analysis, proteomic and metabolomic profiling will be conducted by Alacris Theranostics or subcontracted by Alacris if necessary.

5.3. Selection of study population

Records and imaging results of patients that recently (within preceeding 6 month) presented with neovascular AMD at the ophthalmology department of the Charité (CBF) and are currently treated with intravitreal Anti-VEGF are retrospectively reviewed. Patients will be selected and after informed written consent consequently recruited according to the inclusion and exclusion criteria at the time of their next visit:

The population will consist of 2 cohorts:

- 25 Patients that require a high frequency of intravitreal anti-VEGF (≤ 6 weeks) with insufficient supression of CNV activity
- 25 Patients that require a low frequency of intravitreal anti-VEGF (≥ 10 weeks) to supress CNV activity

Only one eye will be designated as the study eye. For subjects who meet eligibility criteria in both eyes, the eye with the worse visual acuity (VA) will be selected as the study eye. If both eyes have equal VA, the eye with the clearest lens and ocular media and least amount of subfoveal scar or geographic atrophy will be selected.

5.3.1. Inclusion criteria

1. Men and women ≥ 51 years of age
2. Active subfoveal CNV secondary to nAMD (all lesion types) in the study eye
3. BCVA ≤ 0.8 and ≥ 0,05 in the study eye
4. Cohort 1:
   - The intervals between the current and the last as well as between the last and second last intravitreal injection in the study eye was ≤ 42 days (6 weeks)
   - CNV regarded as active in the study eye as evidenced by residual fluid present on OCT at current and the last two visits before the injections
5. Cohort 2:
   - The intervals between the current and the last as well as between the last

and second last intravitreal injection in the study eye was ≥ 70 days (10 weeks)

- CNV activity regarded as controlled in the study eye as evidenced by fluid absent or stable on OCT at current and the last two visits before injections

6. In case both eyes of an individual patient meet the inclusion criteria, the eye with the lower visual acuity will be included
7. Informed written consent

5.3.2.Exclusion criteria

1. Causes of CNV other than neovascular AMD in the study eye
2. Subretinal hemorrhage in the study eye, which warrants surgical intervention except for intravitreal therapy with an VEGF inhibitor
3. Any contraindication for continuous intravitreal therapy
4. Persons with any kind of dependency on the investigator or employed by the sponsor or investigator
5. Persons held in an institution by legal or official order or legally incapacitated

5.4. Withdrawal of study subjects after study start

Study subjects will be early discontinued from participation in the study if

- they withdraw consent from participating in the study
- any illness or other medical condition or situation occurs that would not go along with the best interest of the patient in case of continuing the participation.

5.5. Examinations included in study

The following assessments are performed and results are included in the study database.

Best corrected visual acuity (BCVA)

Visual function of the study eye and the fellow eye will be assessed using the ETDRS protocol (The Early Treatment Diabetic Retinopathy Study Group, 1985) and as decimal BCVA.

Intraocular pressure (IOP)

The intraocular pressure is obtained at every visit by either Goldman tonometry or non-contact tonometry. IOP will be assessed from both the study eye and the fellow eye.

Slit lamp biomicroscopy and Funduscopy

Slit lamp biomicroscopy will be performed for both the study eye and the fellow eye.

Anterior segment assessment: The examination of the anterior segment is to be performed only by the slit lamp without any additive lenses.

Posterior segment assessment (funduscopy): The posterior segment is examined with the slit lamp and an appropriate additional lens. As in clinical routine, for this examination the pupil of the eye must be dilated (mydriasis) with phenylephrin-tropicamid applied topically to the eye. Results are documented by taking a digital colour widefield fundus image (fundus photo)

Autofluorescence Imaging (FAF)

A CE certified device is being used to record FAF. In brief, FAF collects fluorescence from the retina to provide an indicator of RPE integrity.

Optical Coherence Tomography (OCT)

A Spectral Domain OCT of Heidelberg Engineering (Spectralis, Heidelberg Engineering, Heidelberg, Germany) should be used to perform the measurements at every time point indicated in the visit schedule. OCT-images of the study eye and the fellow eye will be captured. OCT technicians and equipment are certified to ensure consistency and quality in image acquisition.

Fluorescein Angiography

Standardized angiography is performed by fluorescein angiography using a confocal scanning laser ophthalmoscope (Spectralis, Heidelberg Engineering) if indicated by the treating physician. Besides central images of the macula, the periphery will be covered with ultra-wide lens using 9 separate images. The angiograms follow a standardised protocol.

Optical Coherence Tomography Angiography (OCT-Angiography)

Optical coherence tomography angiography (OCT-A) is a non-invasive technique for imaging the microvasculature of the retina and choroid (ZEISS Angioplex). OCT-A technology uses laser light reflectance of the surface of moving red blood cells to accurately depict vessels through different segmented areas of the eye, thus eliminating the need for intravascular dyes. The OCT-A will be performed at baseline and follow-up visit in addition to routine clinical practice.

Biosampling of blood specimen

A blood draw will be performed at the baseline visit and further analysed by Alacris Theranostics GmbH. Whole blood samples must be prevented from coagulating, preferably by the addition of EDTA. After blood draw, tubes with blood-EDTA have to be inverted 5-10 times and incubated at room temperature for 30 minutes, prior aliquoting and storage. Aliquots with the minimal needed sample quantity for each analysis have to be prepared immediately in chilled, polypropylene

tubes. Flash-freezing of samples for Whole Genome Sequencing, Whole Proteome Analysis and Metabolome analysis in liquid nitrogen is recommended and storage of the samples in a -80°C freezer or on dry ice until shipment. For Deep Immune Analysis. PBMCs have to be separated immediately from whole blood without flash-freezing (as described below) and stored at -80 °C until shipment . Alternatively, whole blood can be shipped freshly (without freezing in liquid nitrogen) after blood drawing and has to be further processed at Alacris within 24 hours..

Table: Biosampling of blood specimen

Type of Analysis	Required sample	Min. Required Volume	Conservation	Storage	Shipping condition
WGS	Blood-EDTA	1x 2ml	Snap-frozen	-80 °C	Dry ice
Whole Proteome	Blood-EDTA	1x 50 µl	Snap-frozen	-80°C	Dry ice
Metabolome	Blood-EDTA	1x 500 µl	Snap-frozen	-80°C	Dry ice
Deep Immune Analysis	PBMCs	(>100,000 cells)	Lysed in RLT solution (Qiagen) or cryopreserved or dry-frozen pellets	-80 °C	Dry ice
	Blood EDTA	10 ml	fresh	4 °C	4 °C

Biobanking

Blood samples are stored at -80 °C in locked freezers with available emergency backup freezers, controlled room access and documented temperature monitoring. From each sample multiple aliquots will be prepared, each tracked with a unique barcode ensuring security and traceability of every sample. Sample preparation for shipment and courier shipment is available on demand.

Whole Genome Sequencing (WGS)

For whole genome sequencing (WGS) a minimum of 1x 2 ml of blood will be collected with EDTA used as anticoagulation agent. Frozen sample shipment on dry ice is required. Extraction: DNA will be extracted from whole blood for the purpose of whole genome sequencing using the QIAamp DNA blood mini kit. DNA concentration will be determined with fluorometric method (Qubit). Library preparation and sequencing: Whole genome libraries will be prepared using the

TruSeq DNA PCR-free Kit from Illumina. Sequencing is performed on Illumina sequencing platform in PE100 mode aiming at 90 GB of filtered data per library.

Whole Proteome Analysis

For whole proteome analysis a minimum of 50 µl of whole blood samples will be collected, and snap-frozen in liquid nitrogen. Samples need to be shipped frozen on dry-ice. Proteomic Profiling of Human Plasma or Whole Blood Samples will be carried out using HRM™ Mass Spectrometry. A protein inventory (spectral library) for the provided samples is generated using shotgun LC-MS/MS from pooled samples of the provided sample groups. The spectral library is used in a subsequent working step to identify peptide signals in the HRM maps. To obtain a deep library, samples are pre-fractionated.

Metabolome Analysis

For metabolome analysis a minimum of 500 µl of whole blood samples will be collected, and snap-frozen in liquid nitrogen. Samples need to be shipped frozen on dry-ice. For a comprehensive survey of the metabolome 1000-2000 metabolites per sample are detected and their abundance levels are compiled. Metabolites for the following groups of substances can be identified: Amino acid metabolism, carbohydrate metabolism, lipid metabolism, co-factor & vitamin metabolism, energy metabolism, nucleotide metabolism.

Deep Immune Analysis

For deep immune analysis 8 ml of blood will be collected directly into BD Vacutainer® CPT™ Mononuclear Cell Preparation Tube, followed by subsequent separation of mononuclear blood cells (PBMCs) in the same tube. This decreases the complexity of steps for mononuclear cell separation, thereby minimizing variability from sample processing. PBMCs can be stored in RLT solution (Qiagen) or in RNAprotect Cell Reagent (Qiagen), or as cryopreserved pellet. Alternatively, 10 ml of blood can be drawn with EDTA used as anticoagulation agent, stored at RT and send to Alacris within 24 hours for PBMC isolation. Blood samples should be free of clots and processed as soon as possible after collection to ensure optimal results. Delays in processing the blood (storage for 24 hours) is known to reduce the quality of the sample (loss of viability, lower cell recoveries and more contaminating granulocytes and/or erythrocytes, altered expression of surface markers) and is not recommended.

After separation of PBMCs, RNA is extracted with RNeasy Mini Kit from Qiagen. Library preparation and sequencing: Multiplex RT-PCR is performed for targeted amplification of T-cell receptor beta chains (TCRβ) and B-cell receptor heavy chains (IgM, IgA and IgG). Amplicons are tagged with Illumina indices and sequenced with Illumina platform in PE100 (170+45 bases) mode with ≥10 million reads per sample.

5.6. Description of visits

Table 2: Investigations during the clinical study

**Visits**	**1**	**2**
**Month**	**0**	**3**
	can be carried out within ±2 weeks depending on clinical practice	
Retrospective chart review[1]	**x**	
Informed consent[2]	**x**	
BCVA (ETDRS)	**x**	**x**
Intraocular pressure (IOP)	**x**	**x**
Slit lamp + Funduscopy	**x**	**x**
Fundus photography	**x**	**x**
Optical coherence tomography (OCT)	**x**	**x**
Fundusautofluorescence (FAF)	**x**	**x**
Fluorescein angiography (FA)[3]	**(x)**	**(x)**
OCT-Angiography (OCT-A)	**x**	**x**
Blood sampling of 20ml[4]	**x**	

[1]retrospective chart review of patients demographic data and medical history of the preceeding 6 month to screen for inclusion and exclusion criteria

[2] written informed consent will be obtained before any study related investigations are performed

[3]performed as indicated by the treating physician as part of standard clinical routine

[4] blood samples will be handled and further analysed bei Alacris Theranostics

5.7. Outcome parameters

The data will be extracted from the source data collection in order to characterize the overall cohort and the two sub-cohorts. Additional variables may be derived from the source data as appropriate.:

- Epidemiological: Age, Sex, Gender, Disease Duration
- Ophthalmic / functional: best corrected visual acuity (BCVA), intraocular pressure (IOP)
- Ophthalmic / morphological: central retinal thickness (CRT), subretinal fluid (SRF), intraretinal cysts (IRC), pigment epithelial detachment (PED), CNV lesion size (CNV), outer retinal tubulation (ORT), external limiting membrane (ELM) and ellipsoid Zone (EZ) abnormalities, small dense particles (SDP), posterior vitreous detachment (PVD), vitreomacular adhesion (VMA), CNV blood flow, CNV pattern, CNV size, superficial and deep vessel density
- Systemic: genomic, immunomic-, proteomic- and metabolomic parameters

5.8. Data quality assurance

All data relevant to the study are documented soon after measurement by the investigator in the clinical software database. Entering data may be delegated to members of the local physician team.

<u>Data management</u>

The open source clinical study software OpenClinica will be used for electronic clinical data capture and clinical data management. The system will be accessed via a web interface and data will be stored in a relational database independent from the omics data integration platform in order to ensure separation and security of clinical data. Further security measurements include SSL encrypted data connection, a separate user authentication, daily database backup and user account management with regards to user groups, user roles and clinical site control. Data will be captured with CDISC compliant terminology using electronic case report forms (eCRF). The scope and content of eCRFs with regards to clinical variables will be provided by the clinical site. Further adjustment of the variable catalogue and resulting version control of the eCRFs will be conducted in collaboration with the clinical site.

Archiving

All data in the database, informed consent forms and other important study materials will be archived for at least 10 years in accordance with §13 (10) of the GCP-Verordnung.

Biobanking

Storage: Blood samples are stored at -80 °C/-40 °C in locked freezers with controlled room access and emergency backup freezer and documented temperature monitoring.

Sample tracking and management: From each sample multiple aliquots will be prepared, each tracked with a unique barcode ensuring security and traceability of every sample.

Sample receipt and processing: All processes for sample receipt, handling, and storage and access control, including routine temperature monitoring and backup procedures in case storage equipment fails are properly documented.

6. Rates of organisational and administrative aspects of the study

6.1. Sponsor

Sponsor:	Charité – University Medicine Berlin<br>Charitéplatz 1<br>10117 Berlin<br>Germany

6.2. Principal Coordinating Investigator

Principal Coordinating Investigator:	Prof. Dr. med. Oliver Zeitz<br>Charité University Medicine Berlin<br>Department of Ophthalmology, CBF<br>Hindenburgdamm 30<br>12203 Berlin<br>Germany

6.3. Laboratory diagnostics

All laboratory assessments of blood samples will be performed by Alacris Theranostics or subcontracted by Alacris.

Alacris Theranostics GmbH,
CEO Dr. Bodo Lange,
Max-Planck-Straße 3
12489 Berlin
Tel.: +49 (0)30 8431-225-0
Mail: info@alacris.de

There are no other tasks that will be performed by other service providers.

6.4. Imaging review board

Best corrected visual acuity (BCVA) and retinal imaging will be performed and analyzed by experienced and certified image graders that are part of the study team at the Charité.

6.5. Central organisation units

Project management, Clinical Monitoring, Safety Management:

Department of Ophthalmology, CBF
Hindenburgdamm 30
12200 Berlin
Germany

6.6. Investigators and study sites

This clinical study will be carried out as a single-centre study at the Charité department of ophthalmology (Campus Benjamin Franklin) in Berlin, Germany.

Requirements for investigators and study sites

The investigators and involved physicians at the department of ophthalmology should be familiar with clinical procedures. The investigators and study site staff have to proof knowledge of regulatory procedures, GCP and experience with the conduct of the clinical studies.

6.7. Financing

This investigator initiated study is financially supported by Novartis Pharma, Nürnberg, Germany. Sponsorship is taken over by Charité University Medicine Berlin.

7. Ethical and regulatory aspects

7.1. Independent ethics committee

The clinical study will not be started before approval of the competent ethics committee concerning the suitability of the study site and the qualifications of the investigators and study team.

7.2. Ethical basis for the clinical study

The present study protocol and any amendments were and will be prepared in accordance with the Declaration of Helsinki in the current version.

7.2.1. Legislation and guidelines used for preparation

The present clinical study will be conducted in accordance with the published principles of the guidelines for Good Clinical Practice (ICH-GCP) and applicable legislation (GCP-Verordnung). These principles cover, amongst other aspects, ethics committee procedures, the obtaining of informed consent from study subjects, adherence to the study protocol, administrative documentation, data collection, study subjects' medical records (source documents), documentation and reporting of adverse events (AEs), preparation for inspections and audits, and the archiving of study documentation. All investigators and other staff directly concerned with the study will be informed that domestic and foreign supervisory bodies, the competent federal authorities and authorised representatives of the sponsor have the right to review the study documentation and the study subjects' medical records at any time.

7.3. Obtaining informed consent from study subjects

Study subjects may not be enrolled into the present study unless they have consented to take part in the study after having been informed verbally and in writing in comprehensible language of the nature, scope and possible consequences by a study investigator or physician who is member of the study team. Together with the consent to take part in the study, the study subject must also agree to representatives of the sponsor (e.g. monitors or auditors) or the competent supervisory or federal authorities having access to the data recorded within the framework of the clinical study. The study subject will be informed of the potential benefit and possible side effects of the study. It must be clear to study subjects that he or she can withdraw his or her consent at any time without giving reasons and without jeopardizing his / her further course of treatment.

The originally signed consent form is archived in the investigator site file. Study subjects receive copies of the written information sheet and the signed informed consent form. A copy of the

written information sheet and the signed informed consent form will be filed in the patient's record.

The patient information sheet, informed consent form, all other documents handed out to the study subject and any recruitment advertisements must be submitted for approval before use to the ethics committee. Part of the monitoring activities are to check that the most recent informed consent form was used before the study subject was enrolled and that it was dated and signed by the study subject himself or herself.

7.4. Insurance of study subjects

There will be no additional insurance provided for the study subjects

7.5. Data protection

The provisions of data protection legislation will be observed. It is assured by the sponsor that all investigational materials and data will be pseudonymised in accordance with data protection legislation before scientific processing.

Study subjects will be informed that their pseudonymised data will be passed on in accordance with provisions for documentation and notification pursuant to § 12 and § 13 of the GCP Regulations to the recipients described there. Subjects who do not agree that the information may be passed on in this way will not be enrolled into the study.

8. Statistical methods and sample size calculation

Statistical analysis will be of explorative and descriptive nature. Primary analysis is done at 5% significance level (one-sided). All variables will be analyzed with appropriate statistical methods: continuous variables by sample statistics (i.e. mean, standard deviation, median, quartiles, minimum and maximum) and categorical variables by frequency tables (absolute and relative frequencies).

Descriptive analyses will be performed using boxplots and histograms for the visualization of value distributions. Furthermore, clustering methods and heatmaps will be used to illustrate sample and feature interrelations.

Explorative statistics will be applied for the identification of predictive biomarkers. These methods will either yield single biomarkers or biomarker signatures, including heterogeneous signatures (e.g. a combination of sequence and protein markers).

For the identification of single biomarkers, the Mann-Whitney-U-test will be applied for numerical data (e.g. protein expression), and the Fisher-test for categorical data (e.g. mutations).

For the identification of complex biomarkers, Logistic Regression will be applied. In order to assess the predictive power of the identified marker signatures, permutation and cross-validation will be used.

Sample size calculation

With 25 patients per cohort we have a 95% confidence interval of 53,8±5.3 regarding the ETDRS Visual acuity. That gives us the possibility with a power of 80% and alpha of 5% to detect the effect size of 80% corresponding to 10.8 letters on a ETDRS chart in the two cohorts.

9. Use of study findings and publication

It is planned to publish the study results, in mutual agreement with the PCI, in a scientific journal and at German or international congresses. Publication of the results of the study as a whole is intended. Any publication will take account of the 'Uniform requirements for manuscripts submitted to biomedical journals (International Committee of Medical Journal Editors' (ICMJE) [JAMA 1997;277:927-34]).

Any published data will observe data protection legislation covering the study subject and investigators. Success rates or individual findings at individual study sites are known only to the sponsor.

10. Amendments to the study protocol

To ensure that comparable conditions are achieved as far as possible at individual study sites and in the interests of a consistent and valid data analysis, changes to the provisions of this study protocol are not planned. In exceptional cases, however, changes may be made to the study protocol. Such changes can only be made if agreed by the sponsor, sponsor's representative, the PCI and biometrician, and all authors of this study protocol. Any changes to the study procedures must be made in writing and must be documented with reasons and signed by all authors of the original study protocol.

Amendments made in accordance with § 10 Secs. 1 and 4 GCP-Verordnung that require approval are submitted to the ethics committee and the supreme federal authority and will not be implemented until approved. Exceptions to this are amendments made to avoid immediate dangers.

11. References

1. Mitchell, J. and C. Bradley, *Quality of life in age-related macular degeneration: a review of the literature.* Health Qual Life Outcomes, 2006. **4**: p. 97.
2. Wong, W.L., X. Su, X. Li, C.M. Cheung, R. Klein, C.Y. Cheng, and T.Y. Wong, *Global prevalence of age-related macular degeneration and disease burden projection for 2020 and 2040: a systematic review and meta-analysis.* Lancet Glob Health, 2014. **2**(2): p. e106-16.
3. Ambati, J., B.K. Ambati, S.H. Yoo, S. Ianchulev, and A.P. Adamis, *Age-related macular degeneration: etiology, pathogenesis, and therapeutic strategies.* Surv Ophthalmol, 2003. **48**(3): p. 257-93.
4. Ferrara, N., *Vascular endothelial growth factor and age-related macular degeneration: from basic science to therapy.* Nat Med, 2010. **16**(10): p. 1107-11.
5. Schmidt-Erfurth, U., V. Chong, A. Loewenstein, M. Larsen, E. Souied, R. Schlingemann, B. Eldem, J. Mones, G. Richard, and F. Bandello, *Guidelines for the management of neovascular age-related macular degeneration by the European Society of Retina Specialists (EURETINA).* Br J Ophthalmol, 2014. **98**(9): p. 1144-67.
6. Ho, A.C., B.G. Busbee, C.D. Regillo, M.R. Wieland, S.A. Van Everen, Z. Li, R.G. Rubio, and P. Lai, *Twenty-four-month efficacy and safety of 0.5 mg or 2.0 mg ranibizumab in patients with subfoveal neovascular age-related macular degeneration.* Ophthalmology, 2014. **121**(11): p. 2181-92.
7. Ashraf, M., A. Souka, and R.A. Adelman, *Age-related macular degeneration: using morphological predictors to modify current treatment protocols.* Acta Ophthalmol, 2018. **96**(2): p. 120-133.
8. Tsilimbaris, M.K., M.I. Lopez-Galvez, R. Gallego-Pinazo, P. Margaron, and G.N. Lambrou, *Epidemiological and Clinical Baseline Characteristics as Predictive Biomarkers of Response to Anti-VEGF Treatment in Patients with Neovascular AMD.* J Ophthalmol, 2016. **2016**: p. 4367631.
9. Waldstein, S.M., J. Wright, J. Warburton, P. Margaron, C. Simader, and U. Schmidt-Erfurth, *Predictive Value of Retinal Morphology for Visual Acuity Outcomes of Different Ranibizumab Treatment Regimens for Neovascular AMD.* Ophthalmology, 2016. **123**(1): p. 60-9.
10. Simader, C., M. Ritter, M. Bolz, G.G. Deak, U. Mayr-Sponer, I. Golbaz, M. Kundi, and U.M. Schmidt-Erfurth, *Morphologic parameters relevant for visual outcome during anti-angiogenic therapy of neovascular age-related macular degeneration.* Ophthalmology, 2014. **121**(6): p. 1237-45.
11. Al-Sheikh, M., N.A. Iafe, N. Phasukkijwatana, S.R. Sadda, and D. Sarraf, *BIOMARKERS OF NEOVASCULAR ACTIVITY IN AGE-RELATED MACULAR DEGENERATION USING OPTICAL COHERENCE TOMOGRAPHY ANGIOGRAPHY.* Retina, 2018. **38**(2): p. 220-230.
12. Huang, D., Y. Jia, M. Rispoli, O. Tan, and B. Lumbroso, *OPTICAL COHERENCE TOMOGRAPHY ANGIOGRAPHY OF TIME COURSE OF CHOROIDAL NEOVASCULARIZATION IN RESPONSE TO ANTI-ANGIOGENIC TREATMENT.* Retina, 2015. **35**(11): p. 2260-4.
13. Nesper, P.L., B.T. Soetikno, A.D. Treister, and A.A. Fawzi, *Volume-Rendered Projection-Resolved OCT Angiography: 3D Lesion Complexity Is Associated With Therapy Response in Wet Age-Related Macular Degeneration.* Invest Ophthalmol Vis Sci, 2018. **59**(5): p. 1944-1952.
14. Cipriani, V., R.E. Hogg, R. Sofat, A.T. Moore, A.R. Webster, J.R.W. Yates, and A.E. Fletcher, *Association of C-Reactive Protein Genetic Polymorphisms With Late Age-Related Macular Degeneration.* JAMA Ophthalmol, 2017. **135**(9): p. 909-916.
15. Fritsche, L.G., W. Igl, J.N. Bailey, F. Grassmann, S. Sengupta, J.L. Bragg-Gresham, K.P. Burdon, S.J. Hebbring, C. Wen, M. Gorski, I.K. Kim, D. Cho, D. Zack, E. Souied, H.P. Scholl, E. Bala, K.E. Lee, D.J. Hunter, R.J. Sardell, P. Mitchell, J.E. Merriam, V. Cipriani, J.D. Hoffman, T. Schick, Y.T. Lechanteur, R.H. Guymer, M.P. Johnson, Y. Jiang, C.M. Stanton, G.H. Buitendijk, X. Zhan, A.M. Kwong, A. Boleda, M. Brooks, L. Gieser, R. Ratnapriya, K.E. Branham, J.R. Foerster, J.R. Heckenlively, M.I. Othman, B.J. Vote, H.H. Liang, E. Souzeau, I.L. McAllister, T. Isaacs, J. Hall, S. Lake, D.A. Mackey, I.J. Constable, J.E. Craig, T.E. Kitchner, Z. Yang, Z. Su, H. Luo, D. Chen, H. Ouyang, K. Flagg, D. Lin, G. Mao, H. Ferreyra, K. Stark, C.N. von Strachwitz, A. Wolf, C. Brandl, G. Rudolph, M. Olden, M.A. Morrison, D.J. Morgan, M. Schu, J. Ahn, G. Silvestri, E.E. Tsironi, K.H. Park, L.A. Farrer, A. Orlin, A. Brucker, M. Li, C.A. Curcio, S. Mohand-Said, J.A. Sahel, I. Audo, M. Benchaboune, A.J. Cree, C.A. Rennie, S.V. Goverdhan, M. Grunin, S. Hagbi-Levi, P. Campochiaro, N. Katsanis, F.G. Holz, F. Blond, H. Blanche, J.F. Deleuze, R.P. Igo, Jr., B. Truitt,

N.S. Peachey, S.M. Meuer, C.E. Myers, E.L. Moore, R. Klein, M.A. Hauser, E.A. Postel, M.D. Courtenay, S.G. Schwartz, J.L. Kovach, W.K. Scott, G. Liew, A.G. Tan, B. Gopinath, J.C. Merriam, R.T. Smith, J.C. Khan, H. Shahid, A.T. Moore, J.A. McGrath, R. Laux, M.A. Brantley, Jr., A. Agarwal, L. Ersoy, A. Caramoy, T. Langmann, N.T. Saksens, E.K. de Jong, C.B. Hoyng, M.S. Cain, A.J. Richardson, T.M. Martin, J. Blangero, D.E. Weeks, B. Dhillon, C.M. van Duijn, K.F. Doheny, J. Romm, C.C. Klaver, C. Hayward, M.B. Gorin, M.L. Klein, P.N. Baird, A.I. den Hollander, S. Fauser, J.R. Yates, R. Allikmets, J.J. Wang, D.A. Schaumberg, B.E. Klein, S.A. Hagstrom, I. Chowers, A.J. Lotery, T. Leveillard, K. Zhang, M.H. Brilliant, A.W. Hewitt, A. Swaroop, E.Y. Chew, M.A. Pericak-Vance, M. DeAngelis, D. Stambolian, J.L. Haines, S.K. Iyengar, B.H. Weber, G.R. Abecasis and I.M. Heid, *A large genome-wide association study of age-related macular degeneration highlights contributions of rare and common variants.* Nat Genet, 2016. **48**(2): p. 134-43.

16. Klein, R., C.E. Myers, K.J. Cruickshanks, R.E. Gangnon, L.G. Danforth, T.A. Sivakumaran, S.K. Iyengar, M.Y. Tsai, and B.E. Klein, *Markers of inflammation, oxidative stress, and endothelial dysfunction and the 20-year cumulative incidence of early age-related macular degeneration: the Beaver Dam Eye Study.* JAMA Ophthalmol, 2014. **132**(4): p. 446-55.

17. Lains, I., R.S. Kelly, J.B. Miller, R. Silva, D.G. Vavvas, I.K. Kim, J.N. Murta, J. Lasky-Su, J.W. Miller, and D. Husain, *Human Plasma Metabolomics Study across All Stages of Age-Related Macular Degeneration Identifies Potential Lipid Biomarkers.* Ophthalmology, 2018. **125**(2): p. 245-254.
