# Supplementary material for: Systemic Blood Proteome Patterns Reflect Disease Phenotypes in Neovascular Age-Related Macular Degeneration
Source: Int J Mol Sci. 2023 Jun 19;24(12):10327. doi: 10.3390/ijms241210327 (PMC10299094; doi:10.3390/ijms241210327)
Supplement: Supplementary file 1 [file ijms-24-10327-s001.zip › Revised Suppl File.pdf]

Supplementary Material

Künzel, SE et al. Systemic Blood Proteome Patterns Modulate Disease Phenotypes in Neovascular Age-Related Macular Degeneration. IJMS.

Figure S1: TIC chromatograms, unsupervised clustering, QC, and normalization.

Figure S2: Protein quantification and PCA analysis.

Figure S3: Meta-feature analysis.

Figure S4: Meta-feature visualization and biomarker analysis.

Figure S5: Demonstration of the *k*-Nearest-Neighbor predictor for different *k* values.

Figure S6: LOO-CV (k=3) and test accuracy for different *k* values.

Table S1: Processing time per sample.

Table S2: Numbers of identified proteins, peptides, and peptide ion variants per fraction.

Table S3: Total number of protein groups, peptides and peptide ion variants within the three spectral libraries.

Table S4: Numbers of quantified proteins, peptides, and peptide ion variants in each sample.

Table S5: Meta-feature annotation.

Table S6: Protein ratios and significance levels for all proteins and all logged meta-features (dichotomous features, Cluster 2).

Table S7: Protein ratios and significance levels for all proteins and all logged meta-features (numeric features, Cluster 2).

Table S8: Correlation coefficient and significance level for the SVM classifier.

File S1: Supplementary Methods

File S2: Study protocol

Figure S1. TIC chromatograms, unsupervised clustering, QC, and normalization.

a Total ion current (TIC) chromatograms of the five HPRP fractions. Stable acquisition over the whole gradient was observed. The strongest peaks were found at expected intensities. **b** Cumulative numbers of identifications (black), the number of identification in each run (pink), and the number of analytes not previously identified (red). **c** Unsupervised clustering of the whole dataset including the in-house QC samples. **d** Distribution of coefficients of variation (CV) for protein quantification in triplicate preparation of BGS QC plasma. **e** Comparison of expression ranges of all 46 samples before and after normalization.

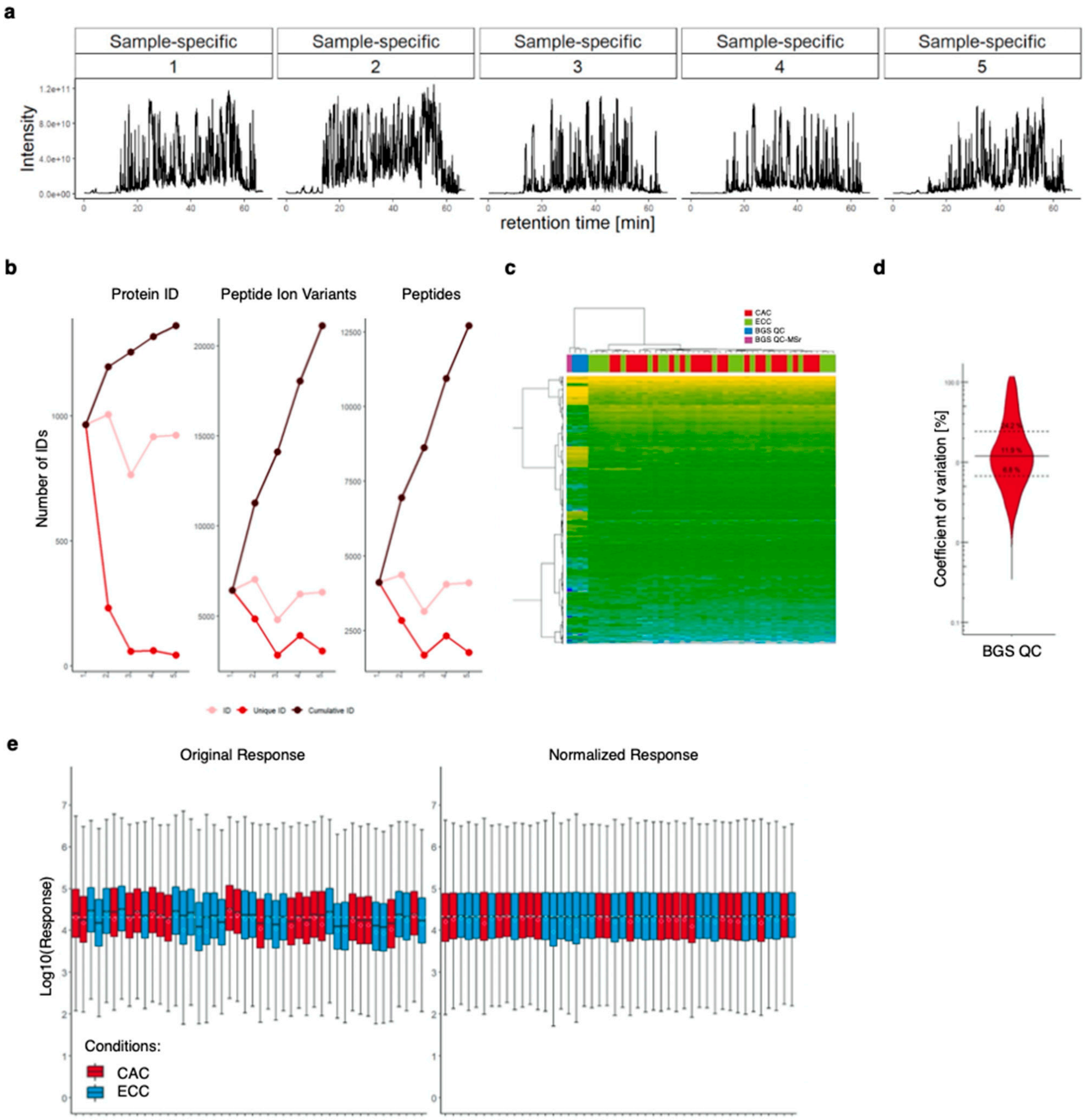

Figure S2. Protein quantification and PCA analysis.

a The violin plot (left) shows the distribution of coefficients of variation (CV) for protein quantification, grouped by divisions. The violin plot (right) shows the distribution of coefficients of variation (CV) for peptide quantification, grouped by cohorts. **b** The number of proteins quantified by HRM across all runs. Red bars: Cumulative sparse profiles accounting for all 1’182 proteins in the data set quantified at least once across the samples with high confidence (sparse data set). Blue bars: Cumulative full profiles accounting for all proteins in the data set quantified in each single sample with high confidence (complete data set). Green dotted line: Size of the spectral library. **c** Principal component analysis (PCA). PC1 and PC2 explain 21.3% of variance between CAC (red) and ECC (blue).

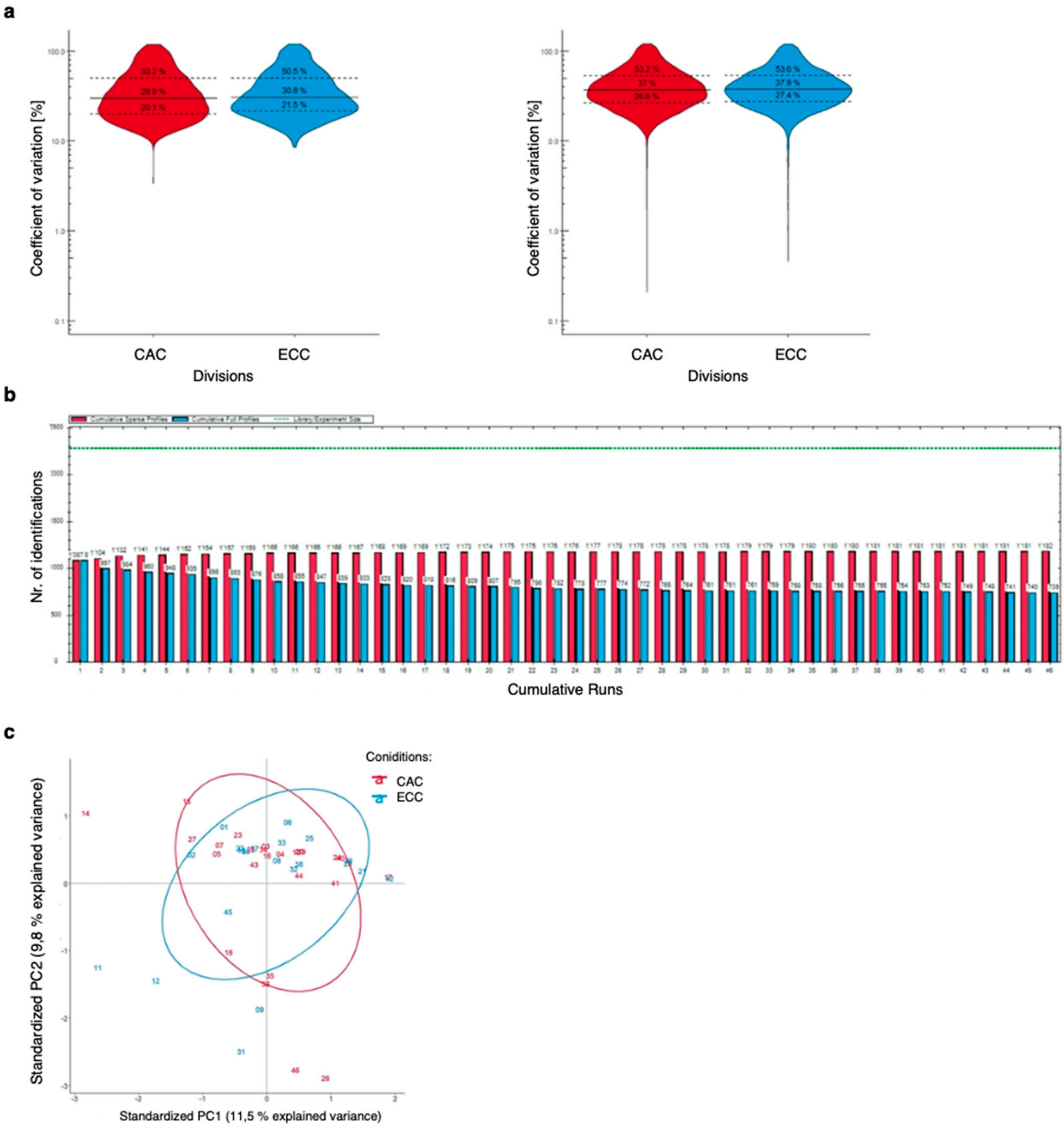

Figure S3. Meta-feature analysis.

a Epidemiological, functional, and morphological baseline characteristics of nAMD patients included in the current study: overall cohort, and as comparison between CAC and ECC divisions. **b** Volcano plot demonstrating significant different meta-features between the two divisions. **c** Significance test between all meta-features.

a

	Overall cohort	CNV control divisions	
		CAC	ECC
N	46	25	21
Age in years (median [IQR])	79.5 [76.0, 83.75]	79.0 [75.0, 82.0]	79.71 [77.0, 85.0]
Sex	23 female, 23 male	10 female, 15 male	13 female, 8 male
Best-corrected visual acuity in LogMAR (median [IQR])	0.46 [0.21, 0.60]	0.48 [0.28, 0.64]	0.46 [0.16, 0.54]
Diabetes Mellitus	8 / 46 (17.4%)	3 / 25 (12.0%)	5 / 21 (23.8%)
Arterial Hypertension	30 / 46 (65.2%)	15 / 25 (60.0%)	15 / 21 (71.4%)
Smoker status	8 / 46 (17.4%)	4 / 25 (16.0%)	4 / 21 (19.0%)
Respiratory dysfunction	15 / 46 (32.6%)	9 / 25 (36.0%)	6 / 21 (28.6%)

b

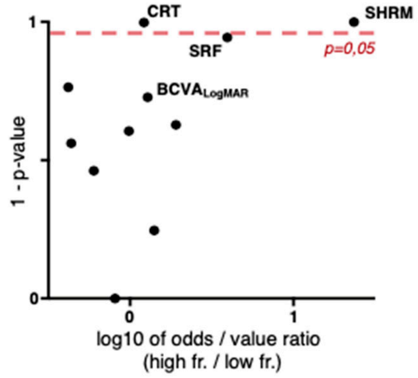

c

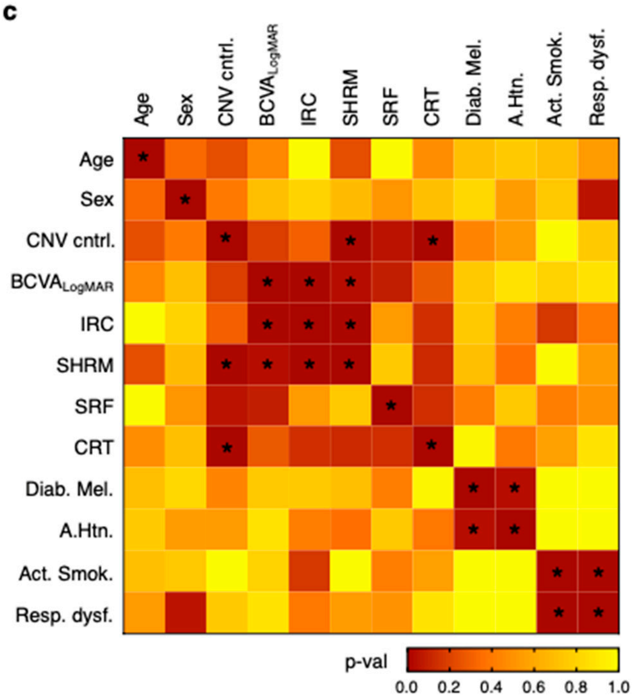

Figure S4. Meta-feature visualization and biomarker analysis.

a Visualization of meta-features on UMAP dimensionality reduction of all 46 patients. **b** Volcano plots for identification of proteomic biomarkers of indicated conditions for all 46 patients. Meta-features without graphic representation yield no significant biomarkers. **c** Comparison of protein values of prefoldin subunit 2 for patients with and without SRF.

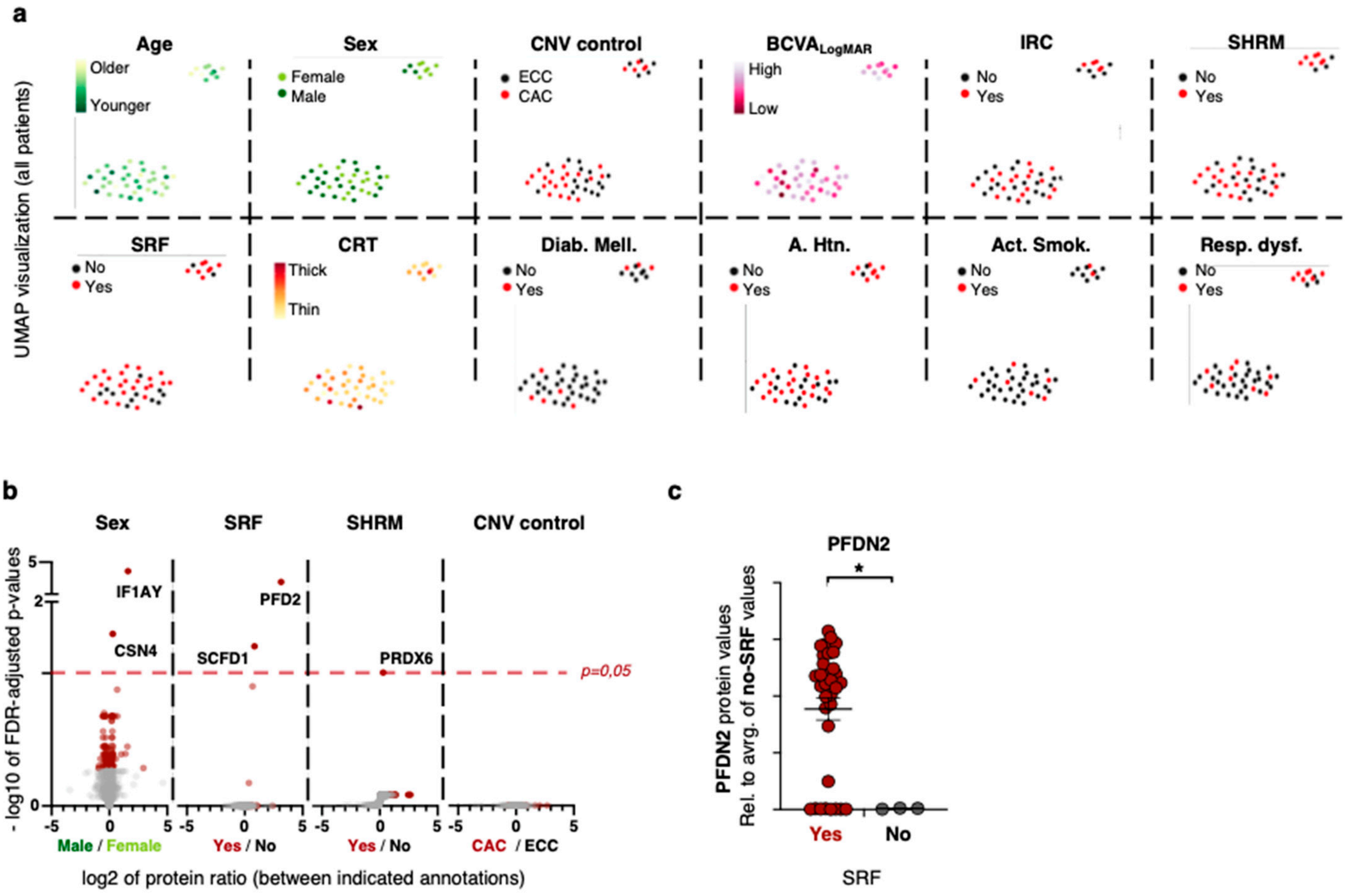

Figure S5. Demonstration of the *k*-Nearest-Neighbor predictor for different *k* values.

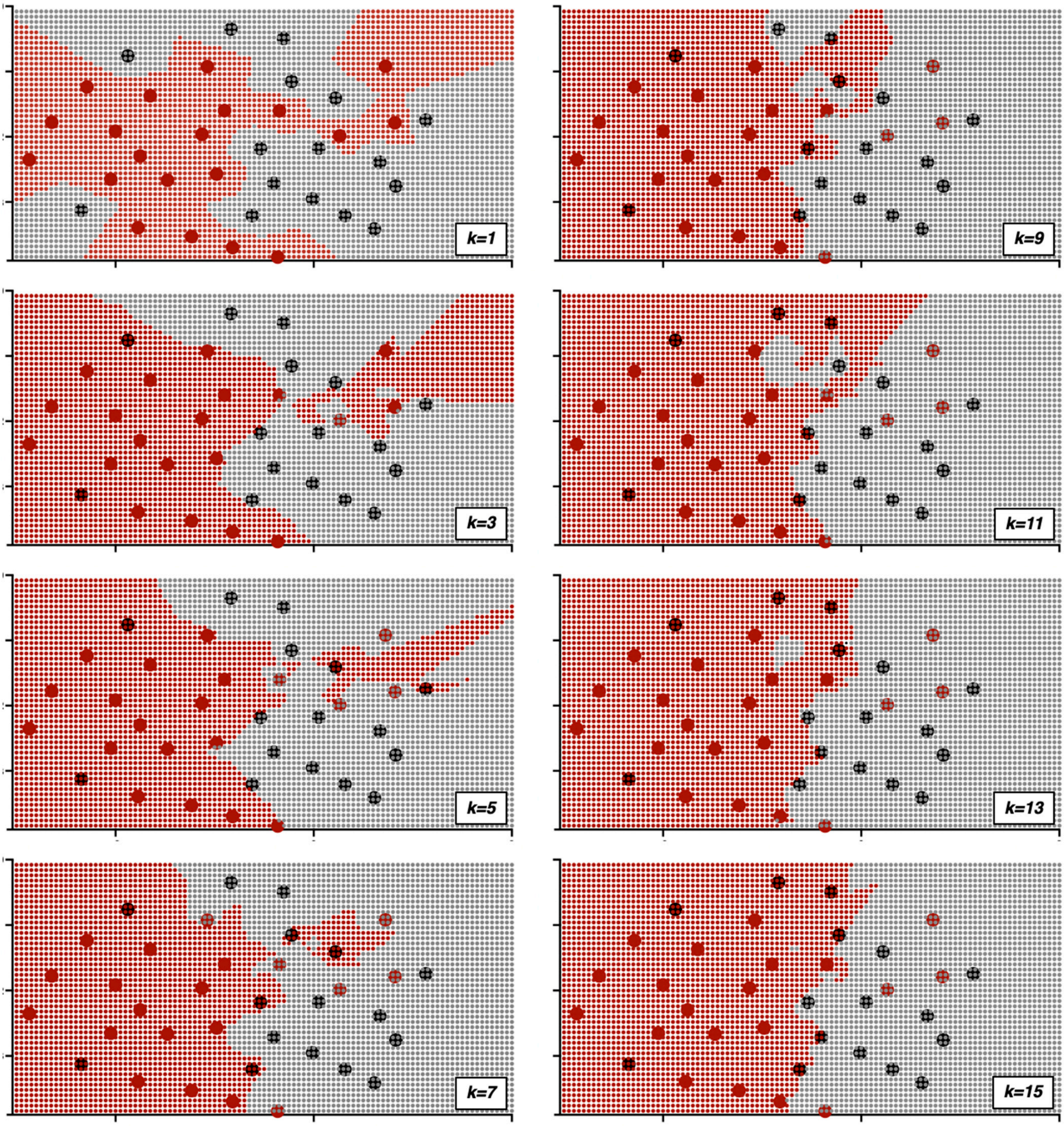

Figure S6. LOO-CV (k=3) and test accuracy for different *k* values.

a LOO-CV for *k*=3 **b** Sensitivity, PPV, NPV, and specificity as a function of *k* for *k*NN in the fully cross validated LOO-CV model.

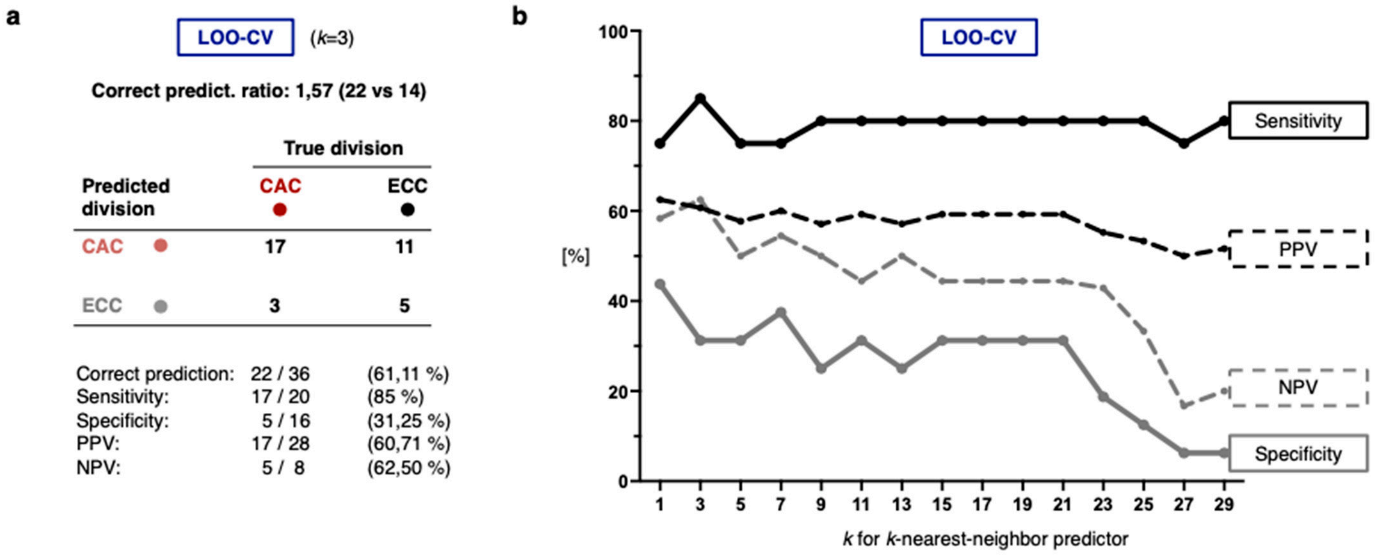

Table S1. Processing time per sample.

**Sample ID**	**Time [minmin:secsec]**
1	08:47
2	09:10
3	07:15
4	10:12
5	09:35
6	09:15
7	09:22
8	08:44
9	10’01
10	09:25
11	09:35
12	09:22
13	07:15
14	06:58
15	10:35
16	09:22
17	08:35
18	09:57
19	09:35
20	09:22
21	09:42
22	09:19
23	09:11
24	08:37
25	09:55
26	09:57
27	09:02
28	09:08
29	10:35
30	09:06
31	08:05
32	09:31
33	09:29
34	09:27
35	10:22
36	08:55
37	07:04
38	10:22
39	11:20
40	09:58
41	09:37
42	09:44
43	10:20
44	09:22
45	08:20
46	09:35

Table S2. Numbers of identified proteins, peptides, and peptide ion variants per fraction.

Fraction	Proteins	Peptides	Peptide Ion Variants
1	965	4’112	6’430
2	1’007	4’371	7’044
3	765	3’144	4’802
4	918	4’055	6’236
5	923	4’096	6’326

Table S3. Total number of protein groups, peptides and peptide ion variants within the three spectral libraries.

Content of the spectral library	Sample-specific	directDIA	Hybrid
Proteins	1’357	996	2’245
Peptides/ protein (average)	9.1	9.1	8.7
Unique peptides	12’414	9’014	19’532
Peptide ion variants (peptides including charges and modifications)	17’776	15’052	32’545

Table S4. Numbers of quantified proteins, peptides, and peptide ion variants in each sample.

Sample ID	Proteins	Peptides	Peptide Ion Variants
1	1’079	10’451	15’737
2	1’066	10’368	15’843
3	1’087	10’485	15’953
4	1’026	9’570	14’543
5	1’099	10’590	15’988
6	1’009	8’520	12’909
7	1’082	10’279	15’643
8	1’087	10’521	15’881
9	1’111	10’652	16’169
10	1’085	10’362	15’553
11	1’119	10’712	16’376
12	1’112	10’947	16’734
13	1’068	10’068	15’126
14	1’090	10’821	16’465
15	1’034	9’141	13’933
16	1’087	10’294	15’668
17	1’059	9’907	14’692
18	1’056	9’417	14’365
19	1’078	10’227	15’536
20	1’063	9’860	14’869
21	1’098	10’574	15’904
22	1’103	10’551	15’986
23	1’070	10’147	15’265
24	1’064	9’875	15’034
25	1’057	9’918	14’858
26	1’101	10’327	15’331
27	1’062	10’250	15’530
28	1’080	10’536	15’964
29	1’071	10’291	15’377
30	1’082	10’393	15’750
31	1’107	10’659	16’204
32	1’091	10’374	15’375
33	1’082	10’384	15’616
34	1’099	10’855	16’209
35	1’098	10’619	15’857
36	1’088	10’240	15’302
37	1’066	10’164	15’127
38	1’049	9’946	14’963
39	1’049	9’840	14’733
40	1’029	9’607	14’112
41	1’042	9’640	14’259
42	1’044	10’021	14’756
43	1’075	10’441	15’892
44	1’072	10’065	15’001
45	1’112	10’670	16’397
46	1’093	10’416	15’592
**Average**	1’076	10’217	15’400
**Total**	1’182	13’326	20’826

Table S5. Meta-feature annotation.

This supplementary material is provided as separate file ('Table S5_Meta-featureannotation').

Table S6. Protein ratios and significance levels for all proteins and all logged meta-features (dichotomous features, Cluster 2).

This supplementary material is provided as separate file ('Table S6_AllProtDichotomousFeatures').

Table S7. Correlation coefficient and significance levels for all proteins and all logged meta-features (numeric features, Cluster 2).

This supplementary material can be found as separate file ('Table S7_AllProtNumericFeatures').

Table S8. Correlation coefficient and significance level for the SVM classifier.

This supplementary material is provided as separate file ('Table S8_CorrelationCoeffSignifSVM').

Study Protocol.

The study protocol is provided as separate file (‘Suppl_Trialprotocol_BIOMAC.pdf’).

Supplementary Methods.

Sample collection and mass spectrometry analysis

A blood draw (50 µl whole blood with EDTA as anticoagulant) was performed at date of study inclusion. Flash-freezing of whole-blood samples was performed in liquid nitrogen with subsequent -80°C storage following standard protocols. Sample collection and processing was performed by predetermined personal only, strictly following the study protocol. Time of sample processing is provided in Table S1 and reveals no significant differences between any annotated meta-features or calculated clusters (not shown). After collection of all samples, they were cumulatively processed. Samples were denatured using Biognosys' Denature Buffer, reduced and alkylated using Biognosys' Reduction and Alkylation Solution for 30 min at 37°C (Biognosys AG, Switzerland). Subsequently, digestion to peptides was carried out using trypsin (Promega, 1:50 protease to total protein ratio per sample) overnight at 37°C. Peptides were then desalted using a C18 Midi spin plate (The Nest Group) according to the manufacturer's instructions and dried down using a SpeedVac system. Peptides were resuspended in LC solvent A (1% acetonitrile, 0·1% formic acid (FA) and spiked with Biognosys' iRT kit calibration peptides. Peptide concentrations were measured using the mBCA assay (Thermo ScientificTM PierceTM). For HPRP fractionation, peptides from each sample were pooled into one pool. Ammonium hydroxide was added to a pH value > 10. The fractionation was performed using a Dionex UltiMate 3000 RS pump (Thermo Scientific) on an Acquity UPLC CSH C18 1·7 µm, 2·1x150 mm column (Waters). The gradient was 2% to 35% solvent B in 10 min, solvents were A: 20 mM ammonium formate in water, B: acetonitrile. Fractions were taken every 30 seconds and sequentially pooled to 5 fraction pools (compare Table S2, Figure S1a). These were dried down and resuspended in solvent A. Prior to mass spectrometric analyses, they were spiked with Biognosys' iRT kit calibration peptides. Peptide concentrations

were determined using a UV/VIS Spectrometer at 280nm (SPECTROstar Nano, BMG Labtech). For DDA LC-MS/MS measurements (Shotgun LC-MS/MS spectral library generation), 1 µg of peptides per sample was injected to an in-house packed reversed phase column (PicoFrit emitter with 75 µm inner diameter, 60 cm length and 10 µm tip from New Objective, packed with 1·7 µm Charged Surface Hybrid C18 particles from Waters) on a Thermo ScientificTM EASYnLCTM 1200 nano-liquid chromatography system connected to a Thermo ScientificTM Q ExactiveTM HF mass spectrometer equipped with a Nanospray FlexTM Ion Source. LC solvents were A: 0·1% FA in water; B: 20% water in acetonitrile with 0·1 % FA. The nonlinear LC gradient was 1 - 59% solvent B in 55 minutes followed by 59 – 90% B in 10 seconds, 90% B for 8 minutes, 90% - 1% B in 10 seconds and 1% B for 5 minutes at 60°C and a flow rate of 250 nl/min. A modified TOP15 method was used. The full range MS1 scan covered the m/z range of 350-1650 with a resolution of 60’000 (AGC target value was 3e6) and was followed by 15 data dependent MS2 scans with a resolution of 15’000 (AGC target value was 1e6). The MS2 acquisition precursor isolation width was 4 m/z while normalized collision energy was centered at 27% and the default charge state was 2+. For DIA LC-MS/MS measurements (HRM mass spectrometry acquisition), 1 µg of peptides per sample were injected to an in-house packed reversed phase column (PicoFrit emitter with 75 µm inner diameter, 60 cm length and 10 µm tip from New Objective, packed with 1·7 µm Charged Surface Hybrid C18 particles from Waters) on a Thermo ScientificTM EASYnLCTM 1200 nano-liquid chromatography system connected to a Thermo ScientificTM Q ExactiveTM HF mass spectrometer equipped with a Nanospray FlexTM Ion Source. LC solvents were A: 0·1% FA in water; B: 20% water in acetonitrile with 0·1 % FA. The nonlinear LC gradient was 1 - 59% solvent B in 55 minutes followed by 59 – 90% B in 10 seconds, 90 % B for 8 minutes, 90% - 1% B in 10 seconds and 1% B for 5 minutes at 60°C and a flow rate of 250 nl/min. The

DIA method consisted of one full range MS1 scan and 21 DIA segments was adopted from Bruderer et al.[12].

The shotgun and HRM mass spectrometric data were then analyzed using Biognosys' search engine SpectroMine (version 2.3), the false discovery rate on peptide and protein level was set to 1%. A human UniProt .fasta database (homo sapiens, 2020-07-01) was used for the search engine, allowing for 2 missed cleavages and variable modifications (N-term acetylation, methionine oxidation, deamidation (NQ) and carbamylation (KR)). The final hybrid resource library was created by searching the obtained data together with a published plasma resource library[13]. HRM mass spectrometric data were then analyzed using Spectronaut Pulsar software (Biognosys). The false discovery rate on peptide and protein level was set to 1%, data was filtered using row-based extraction. The hybrid assay library (protein inventory) generated in this project was used for the analysis. The HRM measurements analyzed with Spectronaut were normalized using global normalization. All solvents were HPLC-grade from Sigma-Aldrich and all chemicals where not stated otherwise were obtained from Sigma-Aldrich. For Spectral Library Generation Five High pH Reversed-phase Peptide (HPRP) fractions of one pool were used to generate a sample-specific spectral library. Each fraction was measured in shotgun LC-MS/MS mode (68 min. gradient per run) on a Thermo Q ExactiveTM HF instrument (compare Figure S1a). Table S2 gives an overview of the numbers of identified proteins, peptides, and peptide ion variants in individual fractions measured. The cumulative number of identifications increases with the number of shotgun measurements, while the number of analytes not previously identified decreases (compare Figure S1b).

All HRM data files were searched using SpectroMineTM software, and a directDIATM spectral library was created. Next, a published plasma resource library was obtained.

The three data sets were used to create a hybrid spectral library (Table S3). For quality control of sample preparation, a Biognosys in-house quality control (BGS QC) plasma sample was prepared together with the patient samples to check the quality of sample preparation. The BGS QC sample was prepared in triplicates. Additionally, an in-house ready to inject plasma sample was measured (BGS QC-MSr). The QC samples show a successful and reproducible sample preparation (Figure S1c, d). Normalization was applied to all runs, in order to minimize variations arising from spray fluctuations and peptide loading on column. Normalization was effective in reducing experimental variation in signal intensity (Figure S1e). Remaining small differences in distribution of peptide intensities are likely resulting from biological differences. Combined biological and technical variation was assessed as coefficient of variance for protein quantities between sample groups (Figure S2a). Median variation was between 29·9% and 30·8% for protein quantification. For quality control or Proteome-Wide Protein Profiling, in total, 1,182 proteins represented by 20’826 peptide ion variants were quantified across all samples. On average 1’076 proteins were quantified per sample. The numbers of quantified proteins, peptides and peptide ion variants in each sample are shown in Table S4 and Suppl. Figure 2b.

Statistical approach and data analysis

Dimensionality reduction and clustering was performed on the identified 1,182 proteomic values without any regards to meta-data annotation in order to reduce potential bias. Expectedly, using Principal Component Analysis (PCA) as a linear model resulted in an unsatisfactory patient arrangement without any obvious patterns indicating a loss of important information during data processing (Figure S2c). To access non-linear patterns, the dimensionality reduction method UMAP (Uniform Manifold Approximation and Projection) was used.[14] The technique has been

successfully applied for similar statistical challenges with single-cell transcriptomic data.[15] Thus, for computational and graphical purposes, we followed the canonical 'Seurat' workflow (Version 4.1.1) with individual samples as 'cells' and proteome values as 'features'.[16] For creation of the SVM classifier, we used the *svm()* function (package 'e1071', no version specified), and for the *k* nearest neighbor approach the *knn()* function (package 'class', version 7.3-20). For cross validation we developed a modified version of leave-one-out cross validation, in terms of not only excluding one sample from cluster 2 (the test sample), but also two random samples from cluster 1, which are of limited relevance for our predictor on cluster 2. This is necessary, as the 'Seurat' workstream with sample reintegration (functions: *FindTransferAnchors*, *TransferData*, *IntegrateEmbeddings*) does not allow to add singular 'cells' (samples). For identification of molecular biomarkers that explain a potential spectrum based on the provided SVM classifier, we calculated the Euclidian distance between the decision boundary and the UMAP coordinate of the sample. Based on this, we performed Pearson correlation testing for every identified protein.

Appropriate statistical methods were applied for descriptive calculations and for hypothesis testing. Test methods and p-values are indicated in figures and corresponding legends. In multiple settings, we tested for molecular marker significances between dichotomous categorial conditions for all 1,182 dimensions (e.g. in Fig. 1b and 2b). To address the multiple-testing problem, we applied the *Mann-Whitney U test* for every protein between the groups and performed a subsequent *Benjamini-Hochberg procedure* for FDR correction of all p-values. If not specified otherwise, data in dot plots is shown as *mean* ± *standard error of the mean* (SEM, e.g. in Fig. 1d and 2c). *Fisher's exact test* was applied for independency testing of all dichotomous categorical variables (e.g. in Fig. 1f,g and 3b,c). For calculations between two numeric variable (e.g. $BCVA_{LogMAR}$ and Age in Figure S3c), we performed Pearson

correlation analyses. In Figure S3a, median and interquartile range (IQR) are demonstrated for continuous and percentages for dichotomous variables. For all quantitative data, statistical analyses were performed using Prism 9.3.1 (Graphpad) and in R (Version 4.2.0).
