# Supplementary material for: Systemic Blood Proteome Patterns Reflect Disease Phenotypes in Neovascular Age-Related Macular Degeneration
Source: Int J Mol Sci. 2023 Jun 19;24(12):10327. doi: 10.3390/ijms241210327 (PMC10299094; doi:10.3390/ijms241210327)
Supplement: Supplementary file 1 [file ijms-24-10327-s001.zip › Table S5.pdf]

Patient_No:	Patient_ID:	CNV cntrl. Division	sex:	enrollment:
1	Pat_76608963	ECC	Male	58
2	Pat_54492928	ECC	Male	77
3	Pat_70941820	CAC	Male	82
4	Pat_40845226	CAC	Male	66
5	Pat_27633233	CAC	Female	85
6	Pat_89310130	ECC	Female	78
7	Pat_31612294	CAC	Male	80
8	Pat_83949912	ECC	Male	84
9	Pat_67500132	ECC	Male	90
10	Pat_67252221	CAC	Male	76
11	Pat_73875872	ECC	Female	85
12	Pat_65778210	ECC	Female	82
13	Pat_31326153	ECC	Female	82
14	Pat_46283813	CAC	Male	80
15	Pat_17245203	CAC	Male	81
16	Pat_68241366	CAC	Male	77
17	Pat_48132856	CAC	Male	82
18	Pat_12571947	CAC	Male	90
19	Pat_45133814	CAC	Male	67
20	Pat_26221381	CAC	Male	71
21	Pat_34733734	ECC	Male	76
22	Pat_38461865	ECC	Female	86
23	Pat_52058796	CAC	Female	91
24	Pat_22604786	CAC	Male	63
25	Pat_42695399	ECC	Male	83
26	Pat_40421987	CAC	Male	94
27	Pat_84309715	CAC	Female	90
28	Pat_86310779	CAC	Female	77
29	Pat_46451988	ECC	Male	82
30	Pat_28774629	ECC	Female	87
31	Pat_86585288	ECC	Female	75
32	Pat_41898454	ECC	Female	79
33	Pat_65733877	ECC	Female	86
34	Pat_47725149	CAC	Female	68
35	Pat_76989158	CCA	Female	68
36	Pat_30673586	CAC	Female	78
37	Pat_57985953	ECC	Female	77
38	Pat_52571904	ECC	Female	71
39	Pat_63089210	ECC	Male	74
40	Pat_28457742	CAC	Female	81
41	Pat_80222324	CAC	Male	82
42	Pat_55380571	ECC	Female	85
43	Pat_58291154	CAC	Male	75
44	Pat_79891072	CAC	Female	76
45	Pat_11846861	ECC	Female	77
46	Pat_25263012	CAC	Female	79

Diabetes:	arterial hypertension:	Smoking, current:	pack years:
yes	yes	no	no
no	yes	no	no
no	yes	no	80
no	no	no	40
no	no	no	15
yes	yes	no	no
no	no	no	30
no	no	yes	60
yes	yes	no	20
no	yes	no	no
yes	yes	no	no
yes	yes	yes	40
no	yes	no	no
no	yes	no	no
yes	yes	no	30
no	no	no	no
no	yes	no	50
no	yes	no	50
no	no	yes	50
yes	yes	no	no
no	yes	no	no
no	no	no	no
no	yes	no	30
no	yes	yes	15
no	no	yes	30
no	no	no	20
no	no	no	no
no	yes	no	no
no	yes	no	no
no	no	no	no
no	yes	no	30
no	yes	no	no
no	yes	yes	60
no	no	no	10
no	yes	no	no
no	yes	yes	30
no	no	no	no
no	no	no	no
no	yes	no	no
no	no	no	10
yes	yes	no	12
no	yes	no	no
no	yes	yes	30
no	yes	no	20
no	yes	no	no
no	no	no	no

Pulmonary dysfunction and cause:	Baseline BCVALogMAR:	Baseline CRT in µm:
none specified	0,16	305
none specified	0,26	325
yes, multiple causes	0,76	510
none specified	1,02	381
none specified	0,60	371
none specified	0,16	293
none specified	0,98	421
COPD (Smoking)	0,24	251
Respiratory distress of unknown cause	0,46	271
none specified	0,72	318
multiple causes, passive smoker	0,60	235
yes, a.e. Smoking	0,62	266
none specified		222
yes, Asthma bronchiale	0,28	188
none specified	1,22	523
none specified	0,24	266
none specified	0,50	297
yes, due to smoking	0,28	325
yes, state after Tuberculosis	0,16	253
none specified	0,52	574
none specified	0,96	340
none specified	0,48	369
none specified	0,82	228
none specified	-0,18	267
COPD (Smoking)	0,74	301
yes, a.e. due to smoking	0,20	392
none specified	0,64	383
none specified	0,44	279
none specified	0,52	243
none specified	0,20	227
none specified	0,46	230
none specified	0,14	275
COPD (Smoking)	0,62	293
none specified	0,38	352
none specified	0,18	373
yes, due to smoking	0,48	524
none specified	0,16	295
none specified	0,02	276
none specified	0,52	167
none specified	0,56	311
none specified	0,28	291
none specified	0,54	273
to smoking, already as a young woman	0,32	338
COPD (Smoking)	0,24	357
none specified	0,12	313
yes, passive smoker, Bronchitis	0,48	284

Baseline IRC present:	Baseline SRF present:	Baseline SHRM present:
not present	present	not present
present	present	not present
not present	present	present
present	present	present
present	present	present
not present	present	not present
not present	not present	present
not applicable	present	not present
present	not present	present
present	present	present
not present	present	not present
present	present	present
not present	present	not present
not present	not present	not present
present	present	present
not present	present	present
present	present	present
not present	present	present
not present	present	present
not present	present	not present
present	not present	not present
present	not present	not present
present	not present	present
not present	present	present
present	not present	present
not present	present	present
present	present	present
present	present	present
present	not present	present
not present	present	not present
not present	not present	not present
not present	present	not present
not present	present	not present
present	not present	present
present	present	present
present	present	present
not present	not present	not present
not present	present	not present
present	not present	present
present	present	present
not present	present	present
not present	not present	not present
present	present	not present
not present	present	present
not present	present	not present
present	present	present
