# Supplementary material for: Systemic Blood Proteome Patterns Reflect Disease Phenotypes in Neovascular Age-Related Macular Degeneration
Source: Int J Mol Sci. 2023 Jun 19;24(12):10327. doi: 10.3390/ijms241210327 (PMC10299094; doi:10.3390/ijms241210327)
Supplement: Supplementary file 1 [file ijms-24-10327-s001.zip › Table S6.pdf]

3 Suppl. Table S6_AllProtDichotomousFeatures

	Sex	Sex	Sex	IVI cohort	IVI cohort	IVI cohort	IRC	IRC	IRC	SHRM	SHRM
.	AVG.Log.Ratio	p-val. (2-sided test)	Adj. p-val. (2-sided test, Benjamini-H. cor.)	AVG.Log.Ratio	p-val. (2-sided test)	Adj. p-val. (2-sided test, Benjamini-H. cor.)	AVG.Log.Ratio	p-val. (1-sided test, IRC > no IRC)	Adj. p-val. (1-sided test, IRC > no IRC, Benjamini-H. cor.)	AVG.Log.Ratio	p-val. (1-sided test, SHRM > no SHRM)
**IGLV4-69**	0.0790085304	7.693066e-01	9.192799e-01	-0.2824281423	0.301230598	0.9995992	7.091512e-02	0.397760115	0.9546035	-0.0822763018	6.174771e-01
**IGLV8-61**	-0.0952694116	6.221051e-01	8.419469e-01	0.0414402407	0.822928548	0.9995992	2.024022e-01	0.152843309	0.9546035	0.1478094643	2.037104e-01
**IGLV4-60**	0.2929770179	5.208075e-01	8.216873e-01	-0.1325837252	0.776402052	0.9995992	6.866525e-01	0.072997623	0.9546035	0.3227181031	2.349314e-01
**IGLV10-54**	1.0234427076	1.352664e-02	3.121263e-01	0.0717843907	0.859070833	0.9995992	-6.207483e-02	0.561187788	0.9546035	0.0243068254	4.751315e-01
**IGLV5-48**	-0.3697934244	5.532319e-01	8.319594e-01	-0.4055677153	0.543920820	0.9995992	3.564972e-01	0.288301864	0.9546035	-0.4766405123	7.455476e-01
**IGLV7-46**	0.3115380616	4.241351e-01	7.973735e-01	0.1080793053	0.785607527	0.9995992	5.427747e-01	0.083363995	0.9546035	-0.0912992834	5.887866e-01
**IGLV2-18**	0.9016328830	2.424313e-01	6.725635e-01	-1.1001381903	0.184688565	0.9995992	4.220229e-01	0.301211531	0.9546035	-1.3081950007	9.291181e-01
**IGLV3-16**	0.0400478603	9.153877e-01	9.738869e-01	0.0981619768	0.798119616	0.9995992	-3.946941e-01	0.853700802	0.9572213	0.0561730446	4.430360e-01
**IGLV3-10**	-0.0050324401	9.799778e-01	9.936282e-01	-0.1063329973	0.603843818	0.9995992	4.346350e-02	0.413436216	0.9546035	0.0682103437	3.767987e-01
**IGLV3-9**	0.1998184882	3.522500e-01	7.498135e-01	-0.2053741316	0.346513530	0.9995992	-3.187268e-01	0.929440842	0.9662261	-0.2423435022	8.531431e-01
**IGKV2-28;IGKV2D-28**	-0.0604973942	7.225903e-01	8.841633e-01	0.1538686972	0.367972408	0.9995992	2.810768e-01	0.050969380	0.9546035	0.1215600383	2.409785e-01
**IGHV3-64**	0.0143344389	9.399950e-01	9.832514e-01	-0.0465801539	0.810826997	0.9995992	1.972611e-01	0.155224720	0.9546035	0.1236242118	2.651522e-01
**IGHV4-4**	-0.0179651940	9.204669e-01	9.747769e-01	-0.1616726611	0.367468149	0.9995992	5.126769e-01	0.001736276	0.6382854	-0.1471588666	7.868350e-01
**IGKV2D-24;IGKV2-24**	0.1270665005	5.096110e-01	8.151018e-01	-0.0848247740	0.668580499	0.9995992	1.132718e-01	0.278440535	0.9546035	-0.0125451157	5.239381e-01
**IGKV2D-29;IGKV2-29**	0.0694084396	9.082921e-01	9.715849e-01	-0.9679559764	0.141144371	0.9995992	-1.619081e-03	0.501066265	0.9546035	-0.7290570641	8.513186e-01
**IGKV1-37;IGKV1D-37**	1.5518676232	8.195747e-03	2.618209e-01	0.8320635804	0.117809153	0.9995992	-9.106978e-01	0.944014956	0.9707604	-0.3526140152	7.259083e-01
**IGLV5-45;IGLV5-39**	0.0499205978	9.005940e-01	9.710488e-01	0.3639016853	0.354952184	0.9995992	9.826827e-02	0.406138824	0.9546035	0.2548387561	2.567556e-01
**IGHV4-30-2**	-0.2037168999	4.035071e-01	7.844497e-01	-0.2313179157	0.354338448	0.9995992	-9.403719e-02	0.648165682	0.9546035	-0.1253745641	6.898302e-01
**IGKV3D-15**	-0.3422413634	4.464571e-01	8.021362e-01	0.5016350578	0.241128886	0.9995992	2.348802e-01	0.306398562	0.9546035	0.4325828616	1.472050e-01
**IGKV1D-8**	0.1601427234	6.023532e-01	8.419469e-01	-0.1728211778	0.563729423	0.9995992	-5.017498e-04	0.500631828	0.9546035	0.0460412236	4.390065e-01
**IGKV2-40;IGKV2D-40**	0.0425271515	8.909348e-01	9.687085e-01	0.0839923464	0.790696041	0.9995992	-1.136757e-01	0.641860993	0.9546035	-0.4361561604	8.967896e-01
**IGKV3D-11;IGKV3-11**	-0.4796929805	2.211251e-02	3.455296e-01	0.1907631703	0.366556633	0.9995992	1.563967e-01	0.232243274	0.9546035	0.1651035892	2.174023e-01
**IGHV1-45**	0.4258925985	2.206340e-01	6.697768e-01	-0.3604567695	0.320907516	0.9995992	5.070984e-01	0.078149681	0.9546035	0.0978306529	3.866036e-01
**IGHV3-49**	0.2955081312	2.199662e-01	6.697768e-01	0.0224854597	0.927036723	0.9995992	-5.652955e-02	0.592479537	0.9546035	-0.0991356916	6.440897e-01
**IGKV6D-21**	-0.9451779711	3.405569e-01	7.413228e-01	-0.9671393299	0.359825551	0.9995992	-9.408146e-01	0.827012536	0.9572213	-0.9275568639	7.973175e-01
**IGKJ1**	0.3664531939	1.442419e-01	6.111413e-01	-0.4264632836	0.096468388	0.9995992	2.272651e-01	0.190184843	0.9546035	-0.2271092611	8.069451e-01
**IGLV1-36**	0.1396139348	6.938892e-01	8.660792e-01	-0.3866030316	0.276609419	0.9995992	-8.672373e-02	0.593798204	0.9546035	-0.2239450757	7.340370e-01
**IGHV6-1**	0.4745664911	2.147198e-01	6.680462e-01	0.1059722806	0.790101369	0.9995992	7.020142e-02	0.428832133	0.9546035	0.5298318397	6.459685e-02
**IGHV3-15**	0.1831268929	6.142640e-01	8.419469e-01	-0.4435254319	0.252111792	0.9995992	-4.685008e-01	0.895898794	0.9579808	-0.6286768786	9.390066e-01
**IGHV2-26**	-0.1387680198	6.291334e-01	8.431244e-01	0.1423490982	0.611114827	0.9995992	1.241414e-01	0.336805370	0.9546035	-0.2862788530	8.347524e-01
**IGHV3-73**	-0.0002163686	9.990571e-01	9.990571e-01	0.0424508357	0.814942918	0.9995992	-4.800626e-02	0.601736084	0.9546035	-0.0002293606	5.005145e-01
**IGHV3-74**	-0.2117599605	5.349300e-01	8.281031e-01	0.2276806785	0.488553318	0.9995992	1.687701e-01	0.313375647	0.9546035	0.1780785102	2.887483e-01
**IGHV3-43**	-0.0507224986	8.241126e-01	9.429827e-01	0.0623823074	0.775486192	0.9995992	-1.837554e-01	0.785853549	0.9572213	0.0483005341	4.147681e-01
**IGLV9-49**	-0.2500868889	7.448934e-01	9.009472e-01	-0.3093044813	0.704923072	0.9995992	-4.706451e-01	0.728000016	0.9546035	-0.2976800390	6.320609e-01
**IGHV3-72**	0.2309817882	2.847421e-01	6.987920e-01	0.0321307060	0.882603259	0.9995992	3.018219e-02	0.445806403	0.9546035	0.2525871901	1.210883e-01
**IGKV1D-13;IGKV1-13**	0.1113290501	5.386365e-01	8.281031e-01	0.1302157263	0.481411022	0.9995992	1.038226e-01	0.286843233	0.9546035	-0.0365817558	5.780572e-01
**IGKV6-21**	-0.0555137106	8.488941e-01	9.510832e-01	-0.6992009378	0.021871712	0.9995992	-1.946407e-01	0.744088331	0.9546035	-0.6013039775	9.705988e-01
**IGKV3D-20**	-0.4113971901	1.013194e-01	5.314990e-01	-0.0139637173	0.958204564	0.9995992	2.338126e-01	0.182059576	0.9546035	-0.1625917242	7.166960e-01
**IGHV1-18**	0.2116047217	3.286030e-01	7.272974e-01	-0.3177054837	0.149114412	0.9995992	-3.473422e-01	0.948585652	0.9707604	-0.2304992510	8.449769e-01
**IGHV3-20**	0.5841938289	1.335294e-01	5.889243e-01	-0.5514232320	0.167660057	0.9995992	-3.359662e-02	0.533652231	0.9546035	-0.3639783345	8.109760e-01
**IGHV1-24**	-0.0755775449	7.699552e-01	9.192799e-01	0.0021639708	0.993438452	0.9995992	-1.878609e-01	0.765085645	0.9572213	-0.0659458846	5.960243e-01
**IGHV4-28**	0.2356666735	2.944828e-01	7.010683e-01	0.0766926971	0.733791528	0.9995992	-1.003974e-01	0.668942755	0.9546035	0.0740800945	3.715164e-01
**IGHV3-35**	-0.0066252428	9.727048e-01	9.926294e-01	0.0543506971	0.784583035	0.9995992	1.015308e-01	0.299496591	0.9546035	0.0264576421	4.477185e-01
**IGHV3-38**	0.3772482390	9.352128e-02	5.196358e-01	0.1127568067	0.621456331	0.9995992	1.048485e-01	0.327350954	0.9546035	0.1976813863	1.901383e-01
**IGHV5-51**	-0.1661371442	4.175242e-01	7.924697e-01	-0.0284865760	0.889868855	0.9995992	4.053416e-01	0.024770749	0.9546035	0.1478833890	2.374772e-01
**IGHV2-70D**	-0.1500767331	6.716634e-01	8.576613e-01	-0.4720493171	0.173635414	0.9995992	-4.836932e-02	0.552782241	0.9546035	-0.3542626763	8.460546e-01
**IGKV1-8**	0.1127158215	3.723527e-01	7.647336e-01	0.0173583270	0.891263422	0.9995992	6.673283e-02	0.303202647	0.9546035	0.0475937670	3.546778e-01
**IGKV1-12;IGKV1D-12**	-0.0723684703	6.703722e-01	8.576613e-01	-0.0670597597	0.692246832	0.9995992	5.268635e-02	0.380587302	0.9546035	0.0515888537	3.765993e-01
**IGHV3-64D**	0.4071183401	4.065198e-01	7.860114e-01	0.5801135566	0.210681963	0.9995992	-4.192616e-01	0.804854618	0.9572213	0.4469542255	1.514232e-01
**IGHV5-10-1**	-0.7814644556	2.244962e-02	3.455296e-01	0.6510868449	0.058316172	0.9995992	9.156627e-01	0.003300702	0.6502383	0.5348446110	6.153219e-02
**IGLC7**	0.2623500451	6.157109e-02	4.562264e-01	0.0958364619	0.503174496	0.9995992	-2.782388e-02	0.576882138	0.9546035	0.1859520469	9.748991e-02
**PGP**	-0.0709120376	5.318064e-01	8.281031e-01	-0.0885532248	0.438399549	0.9995992	1.068621e-02	0.463276434	0.9546035	-0.0551137863	6.841380e-01
**ANXA2P2;ANXA2**	0.0075163606	9.769090e-01	9.928688e-01	0.0912037001	0.728020877	0.9995992	-8.582505e-02	0.627222338	0.9546035	-0.0233910713	5.373852e-01
**SMIM1**	-0.2933879350	5.865272e-01	8.419469e-01	0.0334494986	0.950365243	0.9995992	-5.714844e-02	0.542126339	0.9546035	0.5049967136	1.768077e-01
**PDLIM1**	-0.2428268436	5.403544e-02	4.562264e-01	-0.1076786391	0.407922681	0.9995992	-2.756597e-04	0.500834440	0.9546035	-0.0601755268	6.753728e-01
**ACOT7**	0.2030734847	1.357873e-01	5.932126e-01	-0.1147902408	0.406789356	0.9995992	9.478158e-02	0.235700599	0.9546035	-0.0892634994	7.319992e-01
**SNAP23**	-0.2937139209	2.673130e-01	6.940168e-01	-0.2321691713	0.394773153	0.9995992	2.153947e-02	0.467493848	0.9546035	0.0055978016	4.918603e-01
**MASP2**	-0.1776924445	5.059664e-01	8.151018e-01	0.1939679537	0.446407346	0.9995992	-6.596714e-02	0.597387934	0.9546035	-0.3610637783	8.929400e-01
**PSMD11**	0.0919280284	2.353060e-01	6.697768e-01	-0.1305248475	0.098954315	0.9995992	1.192964e-01	0.066381503	0.9546035	0.0139860960	4.311342e-01
**PSMD12**	-0.0744443021	5.580986e-01	8.319734e-01	-0.2150317746	0.091945132	0.9995992	3.811853e-03	0.488267994	0.9546035	-0.1447429238	8.761842e-01
**PSMD9**	-0.2626133440	2.559060e-02	3.558599e-01	0.0834150546	0.483106804	0.9995992	6.763903e-02	0.291092514	0.9546035	0.1652368429	8.883239e-02
**CLIC1**	0.0953232839	5.758352e-02	4.562264e-01	0.0398044382	0.425372896	0.9995992	2.420479e-02	0.321381924	0.9546035	0.0316718764	2.581402e-01
**QSOX1**	0.2058370392	1.270655e-01	5.775504e-01	-0.1565882217	0.248697241	0.9995992	-6.327488e-02	0.674860302	0.9546035	-0.1500301373	8.514077e-01
**IPO5**	0.1576119406	2.432892e-01	6.725635e-01	0.2266787659	0.092578528	0.9995992	3.402362e-01	0.005517565	0.8152202	0.3240982028	1.039991e-02
**RTCA**	0.1231388363	2.404695e-01	6.725635e-01	0.0149787003	0.889209454	0.9995992	-2.388281e-02	0.589593740	0.9546035	0.1309525413	1.236266e-01
**PSMD14**	0.1815821836	2.422155e-02	3.507394e-01	-0.0579907185	0.475027235	0.9995992	8.051876e-05	0.499617242	0.9546035	0.0072718268	4.658943e-01
**KPNA3**	-0.6161239432	7.453685e-02	4.840800e-01	0.3694596957	0.291278901	0.9995992	-6.269485e-03	0.507317577	0.9546035	0.3062575636	1.974054e-01
**CHL1**	-1.0325999048	3.786134e-01	7.647336e-01	0.6226016856	0.583989920	0.9995992	-8.435253e-03	0.502982585	0.9546035	0.4816269843	3.365460e-01
**FCN1**	-0.4147259839	3.216261e-01	7.225946e-01	0.7461220644	0.063415705	0.9995992	5.526222e-01	0.098881147	0.9546035	0.6787228183	3.736660e-02
**KPNA4**	-0.1369828073	2.947808e-01	7.010683e-01	0.0029685548	0.982252028	0.9995992	5.673232e-02	0.335106249	0.9546035	-0.0116432986	5.336164e-01
**PPP6C**	0.2285349239	1.937883e-01	6.539918e-01	-0.0634623793	0.719639549	0.9995992	-7.498048e-02	0.662127533	0.9546035	0.0215329021	4.528697e-01
**C2CD2L**	-0.3468738122	6.594644e-01	8.528303e-01	0.1030403234	0.895363479	0.9995992	-1.294126e-01	0.565218024	0.9546035	0.9738306909	1.174185e-01
**EIF1AY**	1.6058078169	5.843519e-08	6.907039e-05	0.2086403376	0.512622299	0.9995992	4.656374e-01	0.072599796	0.9546035	0.3511349115	1.431538e-01
**CCS**	-0.0166599713	8.307542e-01	9.460034e-01	0.0047040706	0.952238662	0.9995992	1.210782e-01	0.058385596	0.9546035	0.1415761642	3.801756e-02
**PDCD5**	-0.3011194612	4.260050e-01	7.973735e-01	-0.0683765003	0.856483766	0.9995992	1.397819e-01	0.358109981	0.9546035	-0.3254702456	8.002162e-01
**PRMT5**	0.1332328934	2.477242e-02	3.507394e-01	-0.0287850350	0.640526112	0.9995992	-6.757231e-02	0.861730243	0.9572213	0.0002081962	4.986658e-01
**APOL1**	-0.3314838925	4.948791e-03	2.131357e-01	-0.0068074352	0.955016110	0.9995992	1.778995e-02	0.443568584	0.9546035	-0.1755986287	9.320544e-01
**PSMA7**	0.0181731018	8.470500e-01	9.508196e-01	-0.0551034077	0.571555794	0.9995992	-4.480501e-02	0.679864896	0.9546035	0.0333718369	3.684374e-01
**MYL12B;MYL12A**	-0.2991223961	2.996015e-02	3.864738e-01	-0.0838146788	0.549916555	0.9995992	3.285484e-02	0.407573606	0.9546035	-0.1279935717	8.226195e-01
**HGS**	-0.0568636169	6.864475e-01	8.640904e-01	-0.1044469262	0.487382111	0.9995992	-2.790441e-02	0.579159808	0.9546035	0.0203587180	4.490759e-01
**XPO1**	0.1511211715	6.790334e-02	4.639407e-01	0.0442997629	0.602583547	0.9995992	-1.148434e-01	0.910227154	0.9634873	0.0729758925	2.035225e-01
**PFAS**	0.1931047609	2.669424e-03	1.463013e-01	0.0454287191	0.501071747	0.9995992	2.679925e-02	0.347466439	0.9546035	0.1185555533	4.272842e-02
**ARPC1B**	-0.0744662581	3.510959e-01	7.498135e-01	-0.0231039623	0.774120232	0.9995992	-1.714410e-02	0.583775267	0.9546035	-0.0547498806	7.520995e-01
**ARPC2**	-0.0557774115	5.402372e-01	8.281031e-01	0.0751460964	0.419541873	0.9995992	-1.522120e-01	0.953781646	0.9735491	-0.0029339361	5.123495e-01
**ARPC3**	-0.0315636714	7.298174e-01	8.920829e-01	0.0285319343	0.764793187	0.9995992	-3.383497e-02	0.642047583	0.9546035	-0.0062748394	5.265101e-01
**PLSCR1**	-0.3519770106	5.662333e-01	8.344560e-01	0.0630964194	0.916786073	0.9995992	3.698881e-02	0.476081550	0.9546035	0.5902511437	1.624333e-01
**CLIC2**	-0.1708002802	2.530937e-01	6.814503e-01	-0.3098281216	0.039944583	0.9995992	-3.812605e-02	0.598900507	0.9546035	-0.3183516824	9.809087e-01
**STX7**	-0.4339411453	2.502164e-01	6.761334e-01	-0.2568056953	0.506599729	0.9995992	-1.355515e-01	0.640256290	0.9546035	0.2390273416	2.642416e-01
**ABCC4**	-0.0280087382	9.557935e-01	9.907192e-01	0.2619480233	0.593953895	0.9995992	-1.244340e-01	0.596064279	0.9546035	0.4643768470	1.630369e-01
**CAPN5**	-0.2701948901	4.161436e-01	7.920800e-01	-0.0168381065	0.959039343	0.9995992	-1.742514e-01	0.697606138	0.9546035	0.0758141537	4.064776e-01
**YKT6**	-0.0786216845	4.446271e-01	8.021362e-01	-0.0497590586	0.620502030	0.9995992	-4.225785e-02	0.656311972	0.9546035	-0.0235538057	5.892663e-01
**ARPC5**	0.0509020318	6.060674e-01	8.419469e-01	0.0026893428	0.978478350	0.9995992	-9.524725e-02	0.830300534	0.9572213	-0.0169371836	5.701543e-01
**PSMD3**	0.2034539645	7.216020e-02	4.764992e-01	0.0995207203	0.402032626	0.9995992	8.286422e-02	0.237863376	0.9546035	0.2209073133	3.521388e-02
**TXNL1**	0.0406401955	6.848621e-01	8.640904e-01	-0.0074840176	0.942156862	0.9995992	5.151204e-02	0.308382130	0.9546035	0.0481680850	3.185208e-01
**AKR7A2**	0.0934447468	2.365024e-01	6.697768e-01	0.0937878044	0.237354515	0.9995992	-6.420202e-03	0.531629854	0.9546035	0.1071723755	8.990609e-02
**DENR**	0.2764064434	3.973982e-01	7.802736e-01	0.2870872990	0.359917048	0.9995992	-3.612082e-01	0.867278787	0.9572213	0.2842265466	1.820566e-01
**DNPH1**	0.0958169882	1.808548e-01	6.347267e-01	0.1479495057	0.035629042	0.9995992	1.083776e-01	0.069707811	0.9546035	0.1785345504	5.990003e-03
**RGS10**	-0.0050326667	9.593910e-01	9.912589e-01	0.0245925132	0.800816834	0.9995992	9.439086e-02	0.171459231	0.9546035	0.0456920513	3.198969e-01
**GET3**	0.0065524874	9.060817e-01	9.710488e-01	0.0907169768	0.105987216	0.9995992	-7.797091e-02	0.918071601	0.9644203	0.0631489856	1.365514e-01
**ACTN4**	0.5033731033	1.959121e-01	6.539918e-01	0.2552135670	0.489493303	0.9995992	2.619450e-02	0.472692211	0.9546035	0.6700343067	2.789526e-02
**SGTA**	-0.1092407645	2.928976e-01	7.010683e-01	-0.1433923568	0.192874190	0.9995992	1.153546e-01	0.137899348	0.9546035	-0.0247432729	5.872684e-01
**LANCL1**	0.1505333554	1.225296e-01	5.724507e-01	0.0263136855	0.786334460	0.9995992	1.798119e-02	0.429865539	0.9546035	0.1439382933	5.889347e-02
**CD5L**	0.1926342842	4.494170e-01	8.045226e-01	0.3262810143	0.190755794	0.9995992	5.523701e-01	0.017973098	0.9546035	0.0544837856	4.144665e-01
**GMFG**	-0.4291736269	9.180499e-02	5.171548e-01	-0.7017859528	0.004316373	0.9995992	-5.665097e-01	0.986042865	0.9885519	-0.6216931786	9.934937e-01
**PRPSAP2**	0.2182173813	9.684620e-03	2.792005e-01	-0.0187669555	0.828335988	0.9995992	3.218897e-03	0.485056304	0.9546035	0.0451859291	3.026428e-01
**SNX3**	-0.3085723163	4.607855e-01	8.069339e-01	0.1430883265	0.737045794	0.9995992	-8.974928e-02	0.583510602	0.9546035	0.0164507836	4.851580e-01
**SYNCRIP**	0.0159292838	9.512103e-01	9.905132e-01	-0.1122996634	0.666615295	0.9995992	4.061669e-02	0.439818488	0.9546035	-0.1725690379	7.508514e-01
**GMDS**	0.1764460599	1.625940e-01	6.287166e-01	0.1841714588	0.167884539	0.9995992	1.662858e-03	0.494921356	0.9546035	0.1236356886	1.900950e-01
**DIAPH1**	-0.0571541724	5.260736e-01	8.241536e-01	0.0233875492	0.793424573	0.9995992	-7.214577e-02	0.783435105	0.9572213	0.0067391138	4.704198e-01
**PLIN3**	0.0879853767	3.194167e-01	7.225946e-01	0.0529353893	0.555892120	0.9995992	5.857620e-02	0.259405192	0.9546035	0.0932312941	1.511736e-01
**H2BC12;H2BC11;H2B(**	-0.0022479047	9.863140e-01	9.947297e-01	0.0058227232	0.964543366	0.9995992	-4.779833e-03	0.514445223	0.9546035	0.0670581657	3.057562e-01
**DNAJA2**	0.3080316111	7.744358e-03	2.615380e-01	-0.0438769162	0.720605271	0.9995992	-1.870957e-02	0.565368893	0.9546035	-0.0115352520	5.359467e-01
**CUTA**	0.0725680616	6.357176e-01	8.484797e-01	-0.0353899072	0.825903048	0.9995992	1.517533e-01	0.163555400	0.9546035	0.2388828949	6.232183e-02
**WDR1**	0.0352200290	5.561307e-01	8.319734e-01	-0.0642320278	0.280246025	0.9995992	-9.785245e-02	0.947831780	0.9707604	-0.0711768241	8.812767e-01
**GGCT**	0.0950072714	1.143690e-01	5.517720e-01	0.0793885644	0.202162338	0.9995992	3.855061e-02	0.268730407	0.9546035	0.1088663941	4.212635e-02
**SEMA7A**	-0.3798806007	3.226940e-01	7.225946e-01	0.2939681505	0.431292266	0.9995992	1.730923e-01	0.327935588	0.9546035	0.4914619874	8.836768e-02
**PDCD6**	0.4312568432	3.374164e-01	7.385169e-01	-0.0411359456	0.927262443	0.9995992	-4.842235e-01	0.851364673	0.9572213	-0.3501719526	7.783618e-01
**TBCA**	0.0745542423	2.885030e-01	6.987920e-01	0.0431891159	0.536698757	0.9995992	6.303196e-02	0.187550858	0.9546035	0.0032812052	4.817133e-01
**ATP6V1G1**	0.1506374725	1.540094e-01	6.224838e-01	0.0801898023	0.467115977	0.9995992	1.286500e-01	0.114231358	0.9546035	0.0437375167	3.535909e-01
**VPS4B;FIGNL1;VPS4A**	-0.0303555797	7.979265e-01	9.309940e-01	-0.0002141507	0.998590534	0.9995992	5.598109e-02	0.322879137	0.9546035	0.0967815956	2.174941e-01
**MPDU1**	0.4746628724	6.690633e-01	8.576613e-01	-0.4230395256	0.705925868	0.9995992	2.664655e-01	0.405387678	0.9546035	2.4598974339	3.046612e-02
**SH3BGRL**	0.0112085641	9.687679e-01	9.923248e-01	-0.1599899512	0.584702299	0.9995992	-3.516059e-01	0.889792655	0.9579808	-0.2729128082	8.141943e-01
**SEC22B**	-0.4507450256	2.635687e-01	6.900429e-01	0.0252212112	0.950776421	0.9995992	9.728393e-02	0.405320540	0.9546035	0.4520649891	1.333172e-01
**PGLYRP1**	0.3650500980	1.000600e-01	5.314990e-01	0.1530414865	0.490526568	0.9995992	-7.047746e-02	0.621417933	0.9546035	0.1928441753	1.910150e-01
**LYPLA1**	-0.0238521287	8.608349e-01	9.551688e-01	-0.0721999931	0.605660631	0.9995992	-1.311028e-01	0.826771375	0.9572213	0.0428578255	3.805835e-01
**FCN3**	-0.1363280324	3.480398e-01	7.479692e-01	0.2032788424	0.160520573	0.9995992	8.244885e-03	0.477450975	0.9546035	-0.0290037984	5.775158e-01
**RP2**	0.2818489881	4.287707e-01	7.981212e-01	0.1268559463	0.723529248	0.9995992	-2.155797e-02	0.524951515	0.9546035	0.0875273541	4.043313e-01
**EIF3J**	0.0294973969	8.140470e-01	9.398834e-01	-0.1869202005	0.142709911	0.9995992	8.421661e-02	0.255184707	0.9546035	-0.2189165780	9.473988e-01
**PSMD10**	0.0516968958	3.801560e-01	7.654931e-01	0.0017248229	0.977064382	0.9995992	4.018601e-02	0.251578479	0.9546035	0.0102016102	4.352996e-01
**IDH1**	-0.1721133450	2.800907e-01	6.987920e-01	-0.1297670800	0.420527585	0.9995992	7.689589e-02	0.313953838	0.9546035	-0.1680016715	8.506725e-01
**ATRN**	-0.2105473042	6.758671e-02	4.639407e-01	0.0700023053	0.557323597	0.9995992	-3.272561e-02	0.607698049	0.9546035	-0.0371354738	6.246607e-01
**RBBP9**	0.1825115283	4.306662e-01	7.991327e-01	0.0360746280	0.878031647	0.9995992	-1.577048e-01	0.750373347	0.9546035	0.1245411569	3.045536e-01
**ALDH1L1**	-0.0699658216	5.733581e-01	8.344560e-01	-0.0224396476	0.856117213	0.9995992	-7.622922e-02	0.729916178	0.9546035	0.0423319139	3.685108e-01
**FLOT1**	-0.0798437824	6.858982e-01	8.640904e-01	-0.0645293111	0.745039697	0.9995992	1.212905e-01	0.272475145	0.9546035	0.3573722323	2.813634e-02
**GLRX3**	0.1037965740	2.280462e-01	6.697768e-01	0.0290644129	0.736341839	0.9995992	-1.718245e-02	0.576836994	0.9546035	0.0476277926	2.933525e-01

KRT33A	-1.1433708720	1.833525e-01	6.355609e-01	-1.1006366224	0.224434791	0.9995992	-1.081206e+00	0.894277613	0.9579808	-0.9476069350	8.362922e-01
SEC14L2	0.0850617192	5.336491e-01	8.281031e-01	-0.2308352036	0.087399780	0.9995992	-4.631779e-02	0.628729826	0.9546035	-0.1133430373	8.098751e-01
CIAO1	-0.7487243330	1.084454e-01	5.408542e-01	0.1432553320	0.753639682	0.9995992	-8.547535e-01	0.965727542	0.9774190	-0.1180478926	6.041798e-01
ENDOD1	-0.4715176275	2.026974e-01	6.646753e-01	0.0169527037	0.962973570	0.9995992	-1.267284e-01	0.634052734	0.9546035	0.2859290211	2.185194e-01
RTN3	2.3073732788	8.736584e-02	5.137633e-01	1.7203460394	0.159722806	0.9995992	-1.064167e+00	0.820199238	0.9572213	1.4924341577	1.019169e-01
PGLS	-0.0406305023	5.929075e-01	8.419469e-01	0.0495185509	0.521369533	0.9995992	4.606659e-02	0.273857783	0.9546035	0.1085970066	8.405946e-02
IPO7	-0.0987561125	2.479042e-01	6.760373e-01	0.0470969797	0.586193983	0.9995992	1.196060e-02	0.443890491	0.9546035	-0.0464106655	7.015071e-01
ARIH2	0.2949197986	5.604852e-02	4.562264e-01	0.0110866787	0.942718418	0.9995992	7.874035e-02	0.310667407	0.9546035	0.2293863915	7.351380e-02
APOM	-0.1304783456	4.069704e-01	7.860114e-01	0.2154962296	0.155973982	0.9995992	1.404382e-01	0.193304391	0.9546035	0.0816744805	2.919976e-01
PSMG1	0.0282104411	8.963614e-01	9.687085e-01	0.0223565236	0.918638735	0.9995992	-1.970024e-01	0.815560167	0.9572213	-0.2947785127	9.087450e-01
1,00 VNN	-0.1321368527	7.205828e-01	8.833575e-01	0.2208990684	0.547073612	0.9995992	3.874643e-01	0.148197848	0.9546035	0.4336633238	1.232386e-01
ACSL3	0.0076841483	9.879681e-01	9.952791e-01	-0.2682650329	0.608234777	0.9995992	-5.772724e-01	0.866954151	0.9572213	-0.0376711027	5.281334e-01
OXSR1	0.0718070076	4.747706e-01	8.125882e-01	0.1031837400	0.308290487	0.9995992	1.169729e-01	0.120111599	0.9546035	0.1592576091	5.846198e-02
AP2A1	0.0482597164	6.258512e-01	8.423236e-01	-0.0776702972	0.438879487	0.9995992	1.924169e-02	0.421920131	0.9546035	-0.0730901425	7.679426e-01
CAVIN2	-0.1050284298	4.136819e-01	7.920435e-01	-0.0051818092	0.966911511	0.9995992	-2.289842e-01	0.962032336	0.9774190	-0.0654036466	6.935547e-01
BPNT1	0.1195139876	5.549600e-01	8.319734e-01	0.1930936747	0.336890404	0.9995992	-2.285934e-01	0.873730604	0.9572213	0.0945959360	3.162057e-01
CYB5A	-0.0763834656	5.192517e-01	8.216271e-01	0.0469802339	0.704252330	0.9995992	-9.763589e-02	0.796172101	0.9572213	-0.0563599575	6.676341e-01
LDHA	0.0519200192	4.227864e-01	7.970233e-01	0.0431195332	0.502350096	0.9995992	2.200615e-02	0.370613290	0.9546035	0.0621725778	1.729669e-01
ALDH1A1	0.1205430218	4.356613e-01	8.021362e-01	0.0357316049	0.823067875	0.9995992	-7.074740e-02	0.676013197	0.9546035	0.0022673426	4.948097e-01
CYB5R3	-0.1424528663	4.632881e-01	8.069339e-01	0.0385504358	0.842657925	0.9995992	1.919228e-01	0.164221531	0.9546035	0.2660620700	8.218252e-02
GSR	0.0813784886	3.637597e-01	7.553917e-01	0.0173333408	0.852418296	0.9995992	1.072044e-01	0.119626935	0.9546035	0.2013823400	1.141735e-02
MT-CO2	-1.1004590716	1.745139e-01	6.291668e-01	-0.3796998835	0.631815905	0.9995992	-1.345942e-01	0.568789824	0.9546035	0.4199024093	3.047907e-01
SOD1	0.2248252813	8.954930e-04	1.319384e-01	0.0782701507	0.265274023	0.9995992	-1.339880e-03	0.507318436	0.9546035	0.1108078828	5.571723e-02
CP	-0.1473609919	2.088433e-01	6.658594e-01	-0.0919085683	0.437025199	0.9995992	-3.266919e-02	0.606977601	0.9546035	-0.2755680459	9.855638e-01
F13A1	-0.2108855853	1.610679e-02	3.187282e-01	-0.0324379576	0.721506134	0.9995992	-2.017422e-02	0.588649794	0.9546035	-0.1000803849	8.621350e-01
PNP	0.0890344455	2.413337e-01	6.725635e-01	0.0687023442	0.370472798	0.9995992	9.555609e-02	0.102509323	0.9546035	0.1039533386	9.451544e-02
HPRT1	0.0502193746	4.545832e-01	8.045226e-01	0.0072062627	0.917539460	0.9995992	-3.304995e-02	0.685487560	0.9546035	0.0670558795	1.718331e-01
1,00 PGK	0.0928348678	7.355340e-02	4.803322e-01	0.0406506300	0.443528832	0.9995992	1.089531e-02	0.419349202	0.9546035	0.1005855484	3.298995e-02
AK1	-0.0505636265	6.645861e-01	8.576613e-01	0.0666350727	0.568943901	0.9995992	-5.464389e-02	0.675653384	0.9546035	0.1770521986	6.212604e-02
F2	-0.0740216557	4.223680e-01	7.970233e-01	0.1338833088	0.165773969	0.9995992	3.665274e-02	0.350855082	0.9546035	0.0568649391	2.680326e-01
C1R	-0.1333539675	1.676239e-01	6.287166e-01	-0.1056771955	0.278061946	0.9995992	8.306880e-03	0.466690269	0.9546035	-0.2175402847	9.867066e-01
HP	0.4063158882	6.364420e-02	4.597775e-01	-0.0058124639	0.978639488	0.9995992	1.441944e-01	0.259750352	0.9546035	-0.0955462694	6.578970e-01
HPR	-0.1886178269	3.773427e-01	7.647336e-01	-0.2009486314	0.342320051	0.9995992	1.166150e-01	0.297265931	0.9546035	-0.0509712136	5.935741e-01
F9	0.0940089134	5.476926e-01	8.281031e-01	0.0340969259	0.830944976	0.9995992	4.527861e-01	0.001793009	0.6382854	-0.0448422341	6.069523e-01
F10	-0.1068402489	4.014672e-01	7.843541e-01	0.0557322871	0.662911353	0.9995992	3.143534e-02	0.403357755	0.9546035	-0.1322105888	8.534064e-01
CFD	0.3613883469	5.064062e-01	8.151018e-01	0.4729258137	0.355088495	0.9995992	-5.230635e-01	0.831503155	0.9572213	-0.6585692025	8.506717e-01
PLG	-0.1379225790	1.231230e-01	5.729582e-01	0.1105713036	0.222637019	0.9995992	5.135396e-02	0.289020256	0.9546035	-0.0581948808	7.438352e-01
F12	-0.1372337559	2.149762e-01	6.680462e-01	-0.0946702582	0.404588102	0.9995992	-1.633002e-01	0.925256055	0.9644203	-0.1419733377	8.921652e-01
CFB	0.0393870268	6.824080e-01	8.636041e-01	-0.0995082664	0.299797315	0.9995992	-5.716753e-02	0.719623959	0.9546035	-0.1740882309	9.588730e-01
CA1	0.2202910804	2.232461e-03	1.319384e-01	0.0115212357	0.877609826	0.9995992	2.906778e-02	0.352001097	0.9546035	0.0538933709	2.415502e-01
CA2	0.1592037197	5.781317e-02	4.562264e-01	-0.0205202621	0.812623275	0.9995992	3.909948e-02	0.327404839	0.9546035	0.1100632676	1.110633e-01
SERPINC1	-0.1056107505	1.886815e-01	6.427328e-01	0.0056431894	0.943004974	0.9995992	5.052274e-02	0.271689436	0.9546035	-0.0468074282	7.328513e-01
SERPINA1	0.1180720606	1.805392e-01	6.347267e-01	-0.0397143092	0.655831726	0.9995992	-1.040958e-01	0.875428256	0.9572213	-0.0860951945	8.251346e-01
SERPINA3	-0.0841638775	4.698593e-01	8.121938e-01	0.0566287955	0.626343967	0.9995992	-3.751724e-02	0.624191445	0.9546035	-0.0289140679	5.965931e-01
AGT	-0.2342520774	8.795135e-02	5.146460e-01	0.1481362542	0.272225135	0.9995992	2.600134e-01	0.030450348	0.9546035	0.0871390990	2.635643e-01
A2M	-0.0976583130	5.057729e-01	8.151018e-01	-0.1121123610	0.439975684	0.9995992	1.468895e-01	0.163140573	0.9546035	0.0415894066	3.832176e-01
C3	-0.2199484092	2.492564e-02	3.507394e-01	-0.0910922449	0.366416477	0.9995992	-2.032249e-03	0.508468680	0.9546035	-0.1605590788	9.438459e-01
C5	-0.0774399392	4.262466e-01	7.973735e-01	-0.0634310258	0.506202345	0.9995992	-5.873996e-02	0.723492279	0.9546035	-0.1358283894	9.215856e-01
TIMP1	0.0210604382	8.832344e-01	9.666510e-01	-0.0123452433	0.931040595	0.9995992	-3.273149e-01	0.989986915	0.9916649	-0.2626101016	9.686383e-01
CST3	0.0989296274	7.394595e-01	8.976831e-01	0.2829292586	0.318486335	0.9995992	-3.730614e-01	0.896630008	0.9579808	-0.3853283310	8.692971e-01
KNG1	-0.0969610768	3.291913e-01	7.272974e-01	-0.0118006884	0.904229212	0.9995992	-3.153861e-02	0.620881590	0.9546035	-0.0976725363	8.315723e-01
KRAS	-0.0938303662	6.196170e-01	8.419469e-01	-0.0687805191	0.720746031	0.9995992	-5.309504e-02	0.609765500	0.9546035	0.1177978285	2.755575e-01
IGF2	-0.1696695369	1.764673e-01	6.320739e-01	0.0243477522	0.843425923	0.9995992	-8.389946e-02	0.742757548	0.9546035	-0.2397472017	9.759899e-01
JCHAIN	0.0755490472	7.534903e-01	9.072100e-01	-0.0122909061	0.960393231	0.9995992	2.145708e-01	0.192306055	0.9546035	-0.1412612406	7.078046e-01
IGKV1D-33;IGKV1-33	-0.0510109585	8.173018e-01	9.406531e-01	-0.0536682787	0.808022548	0.9995992	5.572678e-02	0.400170376	0.9546035	-0.1602256610	7.580964e-01
IGKV1-39;IGKV1D-39	-0.0080352499	9.566079e-01	9.907192e-01	-0.1437881692	0.333863177	0.9995992	2.218353e-01	0.068514225	0.9546035	0.0827776184	2.890957e-01
IGKV1-17	-0.2083220906	2.285325e-01	6.697768e-01	0.0482104370	0.778973694	0.9995992	1.219011e-01	0.247365877	0.9546035	0.0282810000	4.354931e-01
IGKV1D-16	-0.1423893837	5.405784e-01	8.281031e-01	0.2364178908	0.304685293	0.9995992	8.023422e-02	0.362577653	0.9546035	0.5650157064	7.487950e-03
IGKV1-5	0.7078849775	2.324877e-01	6.697768e-01	0.5025436281	0.364487964	0.9995992	-3.901618e-01	0.744598341	0.9546035	0.5033444076	1.702603e-01
IGKV3-20	-0.1732723838	2.920314e-01	7.010683e-01	-0.1259257419	0.449994104	0.9995992	-1.290818e-01	0.779957698	0.9572213	-0.1246390641	7.662576e-01
IGKV3-15	-0.0897864370	6.788132e-01	8.610482e-01	-0.3059118635	0.180431631	0.9995992	3.982929e-02	0.428705826	0.9546035	-0.3045528411	8.931313e-01
IGLV1-44	0.0894559870	6.278602e-01	8.423731e-01	-0.1435162960	0.437537202	0.9995992	-7.143447e-02	0.648362789	0.9546035	0.1540549781	2.118862e-01
IGLV1-47	0.0890278553	6.456275e-01	8.488673e-01	-0.0833064142	0.675747232	0.9995992	-2.008071e-01	0.848095922	0.9572213	-0.2274284159	8.722668e-01
IGLV1-51	0.2683256802	7.739129e-02	4.905904e-01	-0.0377227875	0.806325264	0.9995992	-1.854842e-02	0.546745359	0.9546035	-0.0910475018	7.227810e-01
IGLV1-40	0.1967661505	3.126702e-01	7.197712e-01	-0.2665285183	0.172467022	0.9995992	-7.056908e-02	0.638696806	0.9546035	-0.4180211575	9.746369e-01
IGLV2-14	0.9774394246	4.728829e-02	4.562264e-01	0.4319330863	0.373445076	0.9995992	1.083708e-01	0.412604432	0.9546035	-0.3189644745	7.331509e-01
IGLV2-23	0.0920475401	7.860359e-01	9.267668e-01	-0.2011147875	0.556930167	0.9995992	-5.219357e-01	0.934699503	0.9667703	-0.5007665859	9.261054e-01
IGLV2-11	0.3703931769	9.852094e-02	5.314990e-01	0.2575078479	0.246972014	0.9995992	-5.318926e-02	0.593675446	0.9546035	0.2400656422	1.274144e-01
IGLV2-8	0.1393120039	5.825347e-01	8.397024e-01	0.0538484859	0.832063680	0.9995992	-2.275747e-01	0.814115163	0.9572213	-0.2028029241	7.713468e-01
IGLV3-19	0.0596260756	7.588940e-01	9.116018e-01	-0.3351103773	0.084278023	0.9995992	7.989424e-02	0.344217201	0.9546035	-0.1700822279	8.061354e-01
IGLV3-25	0.0436271464	8.092349e-01	9.384947e-01	0.0499637672	0.781719794	0.9995992	-3.823761e-02	0.584285582	0.9546035	0.0500025476	3.965400e-01
IGLV6-57	0.0437111317	8.509958e-01	9.517895e-01	0.1424921838	0.542123166	0.9995992	1.571445e-02	0.472729485	0.9546035	0.0384644496	4.360159e-01
IGHV1-69	-0.0560551186	7.831442e-01	9.256765e-01	-0.2996538321	0.158129413	0.9995992	-4.331919e-02	0.584183372	0.9546035	-0.2005080354	8.092721e-01
IGHV1-46	-0.1852277102	5.575347e-01	8.319734e-01	0.2197303576	0.478823795	0.9995992	3.171472e-01	0.159223727	0.9546035	0.3053709459	1.636817e-01
IGHV3-13	0.3516990830	1.487841e-01	6.170624e-01	-0.1160542356	0.646092092	0.9995992	-3.561654e-01	0.960058527	0.9774190	-0.2939763814	8.709154e-01
IGHV3-30;IGHV3-30-5	0.0802969460	6.566663e-01	8.524806e-01	-0.0721921403	0.701003506	0.9995992	-5.994817e-03	0.512781543	0.9546035	-0.2517243311	8.999893e-01
IGHV3-7	0.0798441447	5.429398e-01	8.281031e-01	0.1755907327	0.170611397	0.9995992	-9.695550e-02	0.767734574	0.9572213	0.1162087816	1.846062e-01
IGHV3-9	-0.0270604833	8.736403e-01	9.614924e-01	0.0946919714	0.576793963	0.9995992	-1.844853e-01	0.857539250	0.9572213	-0.0014520204	5.033931e-01
IGHV2-5	0.0999298988	6.017291e-01	8.419469e-01	0.1448078507	0.447822692	0.9995992	-9.983770e-02	0.694357668	0.9546035	0.0185012057	4.620901e-01
PIGR	0.6914369627	2.172104e-03	1.319384e-01	-0.1614496556	0.480156774	0.9995992	2.196675e-01	0.171541315	0.9546035	-0.1780375074	7.638325e-01
IGKC	-0.0237223560	8.614297e-01	9.551688e-01	0.0950186906	0.472218965	0.9995992	-1.815210e-02	0.552430632	0.9546035	0.0886242199	2.497411e-01
IGHG2	0.1045383263	5.908965e-01	8.419469e-01	0.1346628881	0.484099668	0.9995992	-3.570052e-01	0.970208310	0.9776524	-0.1269804541	7.240343e-01
IGHG3	0.2851383970	2.881704e-01	6.987920e-01	-0.1936610077	0.454912661	0.9995992	-1.613156e-01	0.722107434	0.9546035	-0.3988158011	9.208880e-01
IGHG4	-0.0566716945	8.955078e-01	9.687085e-01	0.6820052880	0.105932798	0.9995992	1.326333e-01	0.378185262	0.9546035	0.0894719766	4.248707e-01
IGHM	0.4118667324	1.481274e-01	6.165020e-01	0.2304518167	0.424554166	0.9995992	6.548320e-01	0.008274249	0.9546035	-0.0661795816	5.855035e-01
IGHA1	0.1089806316	5.765083e-01	8.361138e-01	-0.0600784894	0.758425565	0.9995992	1.614872e-01	0.204852127	0.9546035	0.2938477766	6.565099e-02
IGHD	-1.4471322669	5.213752e-01	8.216873e-01	-3.0446553352	0.288590630	0.9995992	-1.838380e+00	0.784116060	0.9572213	-2.6089289487	8.260794e-01
HBZ	-0.9638619444	9.792286e-02	5.314990e-01	0.8029544556	0.151629368	0.9995992	1.830676e+00	0.002128993	0.6382854	1.1312496439	2.074037e-02
HBD	0.2658027350	3.763916e-03	1.853729e-01	0.0133284560	0.890213731	0.9995992	-3.933010e-02	0.655694652	0.9546035	0.0392635030	3.456990e-01
HBE1	0.2280381632	2.922903e-01	7.010683e-01	-0.1638853829	0.443071560	0.9995992	-2.709451e-01	0.890474642	0.9579808	-0.2120229817	8.453913e-01
MB	-0.2332910584	4.922185e-01	8.151018e-01	-0.2413727116	0.488735348	0.9995992	-3.777275e-01	0.863227206	0.9572213	-0.6074823596	9.557441e-01
KRT6A	0.1514511817	4.388240e-01	8.021362e-01	0.0045713414	0.981355253	0.9995992	-1.545611e-01	0.778464810	0.9572213	-0.1047392172	7.115313e-01
SPTA1	0.1252327850	3.587762e-02	4.077295e-01	-0.0052531540	0.932380605	0.9995992	-3.389728e-03	0.521463989	0.9546035	0.0436108330	2.332839e-01
APOA1	-0.2508075413	1.553640e-02	3.187282e-01	-0.0682083099	0.520160451	0.9995992	9.294694e-02	0.198498503	0.9546035	-0.0479823430	6.833192e-01
APOE	-0.3050110406	4.955856e-02	4.562264e-01	-0.4285425537	0.005491839	0.9995992	6.821712e-02	0.337098493	0.9546035	-0.1381742417	8.209012e-01
APOA2	-0.1864899258	5.878682e-02	4.562264e-01	-0.0621692276	0.533952826	0.9995992	2.132925e-02	0.417344518	0.9546035	-0.1196305789	8.894468e-01
APOC1	-0.2850052532	3.063885e-02	3.894099e-01	0.0531312023	0.689416735	0.9995992	9.245152e-02	0.252330500	0.9546035	-0.0207853236	5.654948e-01
APOC2	-0.0230938256	9.169450e-01	9.741133e-01	-0.0488861961	0.822992864	0.9995992	1.546989e-01	0.247514586	0.9546035	-0.0867678853	6.540348e-01
APOC3	-0.1897160140	3.547018e-01	7.513576e-01	0.1426574269	0.501397980	0.9995992	-4.533968e-02	0.587851247	0.9546035	-0.0879660169	6.573492e-01
FGA	-0.0397949846	7.155849e-01	8.819826e-01	-0.1425496243	0.195848711	0.9995992	-1.254821e-01	0.872870640	0.9572213	-0.2788092892	9.916863e-01
FGB	0.0245366643	7.812566e-01	9.256765e-01	-0.1070021012	0.224701769	0.9995992	-5.638739e-02	0.734679518	0.9546035	-0.1263060326	9.136496e-01
FGG	0.0594152712	6.172547e-01	8.419469e-01	-0.2131557609	0.071925272	0.9995992	-8.340972e-02	0.754876445	0.9546035	-0.2620664819	9.820134e-01
GYPA	0.0115304491	9.817906e-01	9.936282e-01	-0.2346777751	0.643820681	0.9995992	-1.521834e-01	0.616873067	0.9546035	0.5542103626	1.259369e-01
SLC4A1	-0.3518952353	4.209808e-01	7.961589e-01	0.0599992970	0.890169269	0.9995992	-6.867700e-02	0.562329801	0.9546035	0.4983599896	1.296005e-01
CRP	-0.0008065939	9.986172e-01	9.990571e-01	-0.4551341201	0.358733631	0.9995992	2.145348e-01	0.320815494	0.9546035	-0.3224376797	7.346627e-01
APCS	0.1061921909	5.917473e-01	8.419469e-01	0.0132532527	0.948551604	0.9995992	-1.824247e-02	0.536152399	0.9546035	-0.1059722092	6.834240e-01
C1QA	-0.2853612608	3.363182e-02	4.027342e-01	-0.0737421685	0.604977716	0.9995992	-8.667981e-02	0.740174675	0.9546035	-0.1707098380	8.630663e-01
C1QB	0.1534923923	6.440148e-01	8.486349e-01	0.8832727384	0.006802246	0.9995992	3.259986e-01	0.167987897	0.9546035	0.7621501373	8.332829e-03
C1QC	-0.1317957602	1.676323e-01	6.287166e-01	-0.0972527461	0.324883665	0.9995992	-1.608694e-01	0.955855032	0.9748237	-0.1868178194	9.555110e-01
C9	0.2357718923	5.465638e-01	8.281031e-01	-0.1138235046	0.780299208	0.9995992	-7.326225e-01	0.965933527	0.9774190	-0.2043706154	6.809560e-01
APOH	-0.2057816542	2.139315e-01	6.680462e-01	0.2170476072	0.180935010	0.9995992	2.482218e-01	0.071189542	0.9546035	0.2662580659	4.530459e-02
LRG1	0.1220933954	6.138995e-01	8.419469e-01	-0.1166393578	0.623499561	0.9995992	-2.343746e-01	0.829855484	0.9572213	-0.3829792182	9.243066e-01
FN1	-0.3434387780	7.686429e-03	2.615380e-01	-0.2566243911	0.054721433	0.9995992	-1.318736e-01	0.842454167	0.9572213	-0.2870098369	9.814407e-01
RBP4	0.2945955447	2.711440e-01	6.940168e-01	0.2298043420	0.371809103	0.9995992	-1.824253e-01	0.748531899	0.9546035	-0.3032490617	8.446265e-01
AMBP	-0.0574300837	8.182396e-01	9.407844e-01	0.1787216809	0.451311874	0.9995992	-9.538498e-02	0.649117275	0.9546035	-0.2338623115	7.948863e-01
ORM1	0.2404717764	2.770970e-01	6.983553e-01	0.0680899105	0.749120402	0.9995992	3.673481e-02	0.434876035	0.9546035	-0.2063609492	8.005595e-01
AHSG	-0.0754875993	3.533385e-01	7.498135e-01	-0.0580274326	0.469930650	0.9995992	-5.614479e-02	0.749404515	0.9546035	-0.1240584353	9.441749e-01
TTR	0.2015473211	1.799339e-01	6.347267e-01	0.1930095773	0.199931094	0.9995992	-2.468095e-01	0.947134897	0.9707604	-0.1150669228	7.679465e-01
ALB	0.0116664749	8.548398e-01	9.533502e-01	0.1218851470	0.048933324	0.9995992	2.318294e-02	0.359620502	0.9546035	0.0419871674	2.591601e-01
GC	-0.0433306629	6.239809e-01	8.419469e-01	-0.0235792901	0.784662442	0.9995992	-8.162513e-02	0.818573071	0.9572213	-0.1524953344	9.631144e-01
PPBP	-0.1445205809	1.638688e-01	6.287166e-01	-0.0250889800	0.813852850	0.9995992	3.193728e-02	0.380852829	0.9546035	0.0084953932	4.694829e-01
PF4	-0.0735582173	4.773932e-01	8.125882e-01	0.0531532780	0.626269853	0.9995992	-7.149373e-02	0.749890545	0.9546035	-0.0081133867	5.314189e-01
TFRC	0.5708024173	4.511564e-01	8.045226e-01	0.8226599186	0.267234212	0.9995992	1.077654e+00	0.085840026	0.9546035	1.3157147953	3.821411e-02
TF	-0.0269618822	7.397152e-01	8.976831e-01	-0.0596600992	0.467753920	0.9995992	6.457436e-02	0.217035745	0.9546035	-0.1120165952	9.124137e-01
LTF	0.1831785610	2.932141e-01	7.010683e-01	-0.0008539811	0.996047194	0.9995992	1.710660e-03	0.496167727	0.9546035	0.1814674152	1.376549e-01
HPX	-0.1713665662	2.435584e-01	6.725635e-01	0.0127751978	0.932147437	0.9995992	3.889795e-02	0.397478876	0.9546035	-0.0086794496	5.225408e-01
ANG	0.2702753014	6.575758e-01	8.524806e-01	0.0696647785	0.909865315	0.9995992	4.044655e-01	0.253408403	0.9546035	0.4938331521	2.214393e-01
F11	-0.0838876369	4.945424e-01	8.151018e-01	-0.1653819046	0.171419158	0.9995992	-1.348487e-01	0.859395729	0.9572213	-0.1992507675	9.516455e-01
KLKB1	-0.0304367443	8.201174e-01	9.407844e-01	-0.0325355715	0.805482257	0.9995992	-3.516510e-02	0.602950638	0.9546035	-0.1555444374	8.773164e-01
C4BPA	-0.4639296154	4.142528e-04	1.224117e-01	-0.0576677559	0.672538349	0.9995992	-1.568486e-02	0.546271253	0.9546035	-0.1589667426	8.775206e-01
VTN	-0.1993873089	4.769791e-02	4.562264e-01	-0.0762405319	0.474188444	0.9995992	6.852359e-02	0.256956836	0.9546035	-0.0608774408	7.196113e-01
CAT	0.1366582833	1.218610e-01	5.724507e-01	-0.0601167453	0.506660205	0.9995992	-1.025519e-01	0.872804720	0.9572213	-0.0283009225	6.225426e-01
PROC	-0.5348960967	3.621047e-03	1.853729e-01	-0.0188916306	0.920812241	0.9995992	-1.484470e-01	0.784058915	0.9572213	-0.0456773885	5.959509e-01
ALDOA	0.1014245986	1.102037e-01	5.450242e-01	0.0469116412	0.471863236	0.9995992	3.268000e-02	0.306881542	0.9546035	0.1074725531	5.124650e-02
CSTB	0.0148018181	9.428924e-01	9.854101e-01	-0.0520110096	0.799032714	0.9995992	-2.431252e-01	0.874647807	0.9572213	-0.1750529403	8.096694e-01
ANXA1	0.0810496555	5.680156e-01	8.344560e-01	-0.1045688212	0.460800068	0.9995992	1.134735e-02	0.468525645	0.9546035	0.0086070282	4.749197e-01
APOB	-0.3107199176	8.535934e-02	5.044737e-01	0.0653287684	0.725251478	0.9995992	-1.467913e-01	0.796444658	0.9572213	-0.1983528709	8.607599e-01
LCAT	-0.3702108330	1.350239e-02	3.121263e-01	-0.0302743810	0.844734904	0.9995992	1.893826e-01	0.105340620	0.9546035	0.0646716805	3.394275e-01
HRG	-0.2110323435	3.379588e-02	4.027342e-01	0.0543357114	0.596456896	0.9995992	5.570991e-02	0.298455217	0.9546035	-0.0206581895	5.820205e-01

IGLV7-43	0.1992754241	3.137463e-01	7.197712e-01	0.1452901641	0.465110219	0.9995992	3.921394e-02	0.422252806	0.9546035	0.1052650597	2.986314e-01
A1BG	-0.0494782458	6.102293e-01	8.419469e-01	-0.0641291603	0.516551947	0.9995992	-4.490545e-02	0.675418995	0.9546035	-0.0759872728	7.769688e-01
VWF	0.0290776885	8.901807e-01	9.687085e-01	-0.3894991602	0.066106332	0.9995992	-1.322782e-01	0.732236618	0.9546035	-0.3360186116	9.340535e-01
SHBG	-0.4726247962	1.572656e-01	6.224838e-01	-0.6573485195	0.064269298	0.9995992	-6.344897e-01	0.968536797	0.9774190	-0.5702048633	9.364297e-01
GAPDH	0.0321011316	5.734286e-01	8.344560e-01	-0.0113524856	0.839075630	0.9995992	-1.479399e-02	0.599670254	0.9546035	-0.0064749852	5.469767e-01
ASL	-0.0457888431	6.270183e-01	8.423236e-01	0.0617398715	0.502882118	0.9995992	8.044471e-02	0.201767853	0.9546035	0.0881363482	1.693831e-01
IGKV1-16	0.0539472818	7.965238e-01	9.305665e-01	-0.0707399425	0.749591758	0.9995992	-3.032152e-01	0.924798295	0.9644203	-0.2158235438	8.182447e-01
HLA-A	-0.6164053161	9.852429e-02	5.314990e-01	-0.1290942400	0.721973957	0.9995992	-7.211359e-02	0.576232463	0.9546035	-0.0390363095	5.431770e-01
CAPNS1	0.0795917066	2.600748e-01	6.900429e-01	-0.0174084444	0.803584722	0.9995992	8.169504e-02	0.130507056	0.9546035	0.0476963819	2.404474e-01
HSPB1	0.1927060846	6.157195e-01	8.419469e-01	0.3253511394	0.380668674	0.9995992	-9.340590e-02	0.595840643	0.9546035	-0.2444017461	7.122315e-01
CYBB	-0.4520546215	1.294345e-01	5.795137e-01	-0.0642695033	0.827516227	0.9995992	-1.714822e-01	0.715265879	0.9546035	0.2190293298	2.355706e-01
GNAI2	-0.5007605503	2.004181e-01	6.617157e-01	-0.2320262731	0.554003452	0.9995992	-2.381496e-01	0.727812801	0.9546035	0.0625124700	4.381073e-01
GYPC	-0.1639925300	7.385492e-01	8.976831e-01	0.1564506286	0.745091749	0.9995992	-3.243893e-01	0.745586876	0.9546035	0.4839273977	1.542532e-01
ATP1A1	0.5136901953	6.076422e-01	8.419469e-01	1.1650308142	0.264554320	0.9995992	-1.690970e+00	0.938627447	0.9681131	0.6244650131	2.695602e-01
ARG1	-0.1331988362	4.605543e-01	8.069339e-01	0.0077140750	0.967720049	0.9995992	6.582955e-02	0.360282222	0.9546035	0.0231113512	4.490813e-01
APOD	0.0918048497	4.622548e-01	8.069339e-01	-0.0381019677	0.764294142	0.9995992	6.069542e-02	0.319466721	0.9546035	0.0359261887	3.791542e-01
ITGB3	-0.6596410160	5.801728e-02	4.562264e-01	-0.0352863081	0.918954486	0.9995992	-2.781979e-03	0.503170287	0.9546035	0.1921160863	2.921322e-01
ITGB2	-0.1426819573	6.777783e-01	8.610482e-01	0.0890049421	0.795353630	0.9995992	-9.991385e-02	0.612391706	0.9546035	0.1677519263	3.144732e-01
S100A8	0.2350462505	1.964188e-01	6.539918e-01	-0.0458424814	0.803373121	0.9995992	4.534614e-02	0.404025079	0.9546035	0.0391341605	4.154300e-01
SLC25A5;SLC25A4;SL	-0.0533102312	9.111179e-01	9.722363e-01	0.5992182908	0.191812305	0.9995992	2.333971e-01	0.312713080	0.9546035	0.8070458143	3.562287e-02
SERPINA5	0.0934388214	4.791658e-01	8.125882e-01	0.0177832646	0.895812140	0.9995992	-1.172125e-01	0.806844716	0.9572213	-0.0400649564	6.125938e-01
SERPING1	0.0860531219	3.841125e-01	7.708336e-01	-0.1257036127	0.199648533	0.9995992	-7.576300e-03	0.529484214	0.9546035	-0.1586093872	9.432812e-01
CFI	-0.3505094430	2.060289e-03	1.319384e-01	-0.0009170048	0.993707292	0.9995992	-2.643087e-02	0.588770567	0.9546035	-0.2109548809	9.601948e-01
F13B	-0.1681969288	9.363996e-02	5.196358e-01	0.0245668252	0.807553870	0.9995992	6.417812e-02	0.266919867	0.9546035	-0.0529040883	7.012062e-01
ISG15	0.4467794642	3.004082e-01	7.059294e-01	0.2218751483	0.600825646	0.9995992	-2.177130e-01	0.690985587	0.9546035	-0.4218952125	8.109325e-01
MPO	0.1373647499	4.141141e-01	7.920435e-01	-0.0707053223	0.673099954	0.9995992	-1.367632e-01	0.788173894	0.9572213	0.0878279606	3.016454e-01
EIF2S1	0.0853357093	3.213326e-01	7.225946e-01	-0.0272503903	0.761283617	0.9995992	-2.484276e-02	0.609826538	0.9546035	-0.1684992758	9.654357e-01
HMGN2	-0.4873639902	2.348096e-01	6.697768e-01	-0.1785338372	0.674519247	0.9995992	-7.000109e-02	0.566432647	0.9546035	-0.1017883709	5.893486e-01
RPLP2	0.0192325159	9.366048e-01	9.823132e-01	-0.0803862812	0.747359278	0.9995992	-3.535981e-01	0.933106884	0.9667703	-0.3043250650	8.786940e-01
RPLP0;RPLP0P6	0.4043388316	1.221755e-01	5.724507e-01	0.0976887506	0.709306826	0.9995992	-8.751142e-02	0.628137697	0.9546035	0.1363887066	2.986728e-01
CLEC3B	-0.3367999452	1.341898e-03	1.319384e-01	-0.1035373391	0.351265883	0.9995992	-2.581547e-02	0.591696781	0.9546035	-0.1358806948	8.951111e-01
SSB	0.0353451621	7.454537e-01	9.009472e-01	0.0176508338	0.871260028	0.9995992	-1.264982e-01	0.873173673	0.9572213	-0.0033197329	5.128073e-01
SERPINA7	0.0625009537	6.197297e-01	8.419469e-01	0.0164444012	0.894043987	0.9995992	-1.974448e-01	0.938290092	0.9681131	-0.1166745392	8.250089e-01
SERPIND1	-0.0906285847	3.496771e-01	7.487834e-01	-0.0701967338	0.476795481	0.9995992	-5.044152e-03	0.520289068	0.9546035	-0.1118909531	8.659070e-01
ITGB1	-1.7850956338	6.709924e-02	4.639407e-01	-0.4266194583	0.637540598	0.9995992	8.089771e-02	0.462668434	0.9546035	0.0633758547	4.737346e-01
UROD	0.0840782835	4.826060e-01	8.129736e-01	-0.0126371858	0.917896339	0.9995992	-1.000596e-02	0.532659384	0.9546035	0.0176055170	4.458957e-01
BCHE	-0.1851444377	1.828486e-01	6.355609e-01	-0.2189380398	0.132682552	0.9995992	-1.160402e-01	0.800624344	0.9572213	-0.1130568420	7.842025e-01
IGKV2-30	0.0844138941	6.949074e-01	8.664351e-01	-0.0568228472	0.789174411	0.9995992	-3.275210e-01	0.934871799	0.9667703	-0.0664208899	6.198343e-01
IGKV4-1	0.0174555760	9.097211e-01	9.722336e-01	0.2140671409	0.154696024	0.9995992	1.513111e-01	0.160829087	0.9546035	0.1025433432	2.544031e-01
IGHV4-34	-0.0165047298	9.175168e-01	9.741133e-01	-0.0582581719	0.716811027	0.9995992	8.863424e-02	0.292933717	0.9546035	-0.0116658725	5.295207e-01
GSN	-0.1144466602	2.615507e-01	6.900429e-01	0.0668317413	0.512279442	0.9995992	-1.157117e-02	0.544823232	0.9546035	-0.0374838478	6.327592e-01
PTMA	0.2471041762	2.882082e-01	6.987920e-01	-0.0069070839	0.976794939	0.9995992	2.813901e-01	0.117651840	0.9546035	0.1470825977	2.690341e-01
ATP5F1B	-0.3291821384	1.138996e-01	5.517720e-01	0.0209545539	0.920456880	0.9995992	5.651369e-02	0.396122072	0.9546035	0.1491741739	2.384087e-01
C2	-0.0634284887	4.766816e-01	8.125882e-01	-0.0878377464	0.352180811	0.9995992	1.454566e-02	0.433545152	0.9546035	-0.0568969143	7.181192e-01
S100A9	0.2119449019	2.117336e-01	6.680462e-01	-0.0315646233	0.855932420	0.9995992	-1.745255e-03	0.504001008	0.9546035	0.0466570061	3.919767e-01
S100A6	0.1327895878	9.641582e-02	5.300628e-01	-0.0333760741	0.689400266	0.9995992	-3.754484e-04	0.501804980	0.9546035	-0.0025601821	5.124960e-01
APOA4	-0.0898345828	6.987488e-01	8.689477e-01	0.1439549816	0.520456706	0.9995992	-1.279682e-01	0.706162894	0.9546035	-0.0420076609	5.727099e-01
EIF4E	-0.0214551492	8.203410e-01	9.407844e-01	-0.0057707582	0.951364797	0.9995992	8.623333e-02	0.184540780	0.9546035	-0.0434956292	6.676517e-01
ENO1	0.0831536695	2.736164e-01	6.944775e-01	-0.0551905930	0.481145309	0.9995992	3.308382e-02	0.338021378	0.9546035	-0.0613832870	7.786112e-01
PYGL	0.2815549016	8.095964e-02	5.027637e-01	0.0284658928	0.865282999	0.9995992	-2.968213e-02	0.570935822	0.9546035	0.1519329061	1.819388e-01
GPI	0.0488818040	6.485993e-01	8.489971e-01	0.0682102316	0.540686713	0.9995992	6.394490e-02	0.278798302	0.9546035	0.1519538080	9.345424e-02
TPM3	-0.0116311683	8.511349e-01	9.517895e-01	-0.0012798757	0.984323191	0.9995992	-5.446517e-02	0.813830458	0.9572213	0.0383807365	2.802604e-01
LDHB	0.1202713449	1.165528e-01	5.600218e-01	-0.0507991618	0.501205601	0.9995992	-4.620379e-02	0.718426692	0.9546035	0.0457875128	2.720086e-01
GPX1	-0.0665003630	6.030824e-01	8.419469e-01	-0.1753803740	0.175778472	0.9995992	-1.740508e-01	0.911318775	0.9634873	-0.1267603216	8.347726e-01
PROS1	-0.1002157818	3.256900e-01	7.249823e-01	0.1479586916	0.152849430	0.9995992	-4.069772e-02	0.654221875	0.9546035	-0.0455329864	6.628046e-01
P4HB	-0.1835334256	4.899640e-01	8.151018e-01	-0.2660087377	0.335167542	0.9995992	3.788158e-02	0.443928287	0.9546035	0.2030888974	2.300116e-01
ACYP1	-0.0156004440	8.412832e-01	9.500891e-01	0.0926155263	0.238722959	0.9995992	6.155155e-02	0.217179538	0.9546035	0.1582773648	2.572316e-02
CTSD	-0.0970980517	6.014635e-01	8.419469e-01	-0.1495142137	0.436622675	0.9995992	-1.151125e-01	0.731335000	0.9546035	-0.3446355354	9.529084e-01
C8A	-0.1785280157	6.727812e-02	4.639407e-01	-0.0911416532	0.348305266	0.9995992	2.864626e-02	0.388253701	0.9546035	-0.1729078729	9.628642e-01
C8B	-0.1720841527	5.151866e-02	4.562264e-01	-0.0131218931	0.885400240	0.9995992	4.289821e-02	0.320294940	0.9546035	-0.0881288072	8.385344e-01
GP1BA	-0.5724759588	1.429422e-01	6.099555e-01	0.0125550077	0.974514265	0.9995992	-3.439770e-01	0.810733384	0.9572213	-0.1860101383	6.710310e-01
C8G	-0.2290150741	3.920967e-02	4.198744e-01	0.0208584038	0.852623782	0.9995992	2.935931e-02	0.397870085	0.9546035	-0.0854458547	7.817303e-01
CAPN1	0.0026048996	9.651328e-01	9.923248e-01	0.0090252100	0.881777302	0.9995992	1.064753e-01	0.036467226	0.9546035	0.0976774413	4.536262e-02
TUBB	-0.1219428871	2.873874e-01	6.987920e-01	-0.0342010095	0.762495017	0.9995992	5.448674e-02	0.322217448	0.9546035	0.0553491681	3.139452e-01
CA3	0.1052876520	4.390692e-01	8.021362e-01	0.0807831705	0.552830935	0.9995992	-1.073304e-02	0.530665644	0.9546035	0.1085875702	2.162327e-01
PRSS1	0.2056465448	1.824650e-02	3.318055e-01	0.0149333976	0.871473403	0.9995992	8.897487e-02	0.165725442	0.9546035	0.1113575702	1.249887e-01
PFN1	0.0287339416	7.916044e-01	9.288162e-01	0.0812312326	0.455655372	0.9995992	-2.631975e-02	0.595299814	0.9546035	0.0672988816	2.675428e-01
BPGM	0.0744466182	2.731762e-01	6.944775e-01	0.0721316664	0.293263782	0.9995992	-6.913533e-02	0.839996562	0.9572213	0.0452889642	2.562229e-01
APRT	0.1593503728	2.188736e-02	3.455296e-01	0.0030990494	0.965469989	0.9995992	7.058773e-03	0.459721566	0.9546035	-0.0054030321	5.311583e-01
HSP90AA1	0.0988094280	1.532574e-01	6.224838e-01	0.0040488741	0.955381481	0.9995992	-1.027357e-02	0.557304769	0.9546035	-0.0185263957	6.043976e-01
TPM2	-0.0886822876	8.817646e-01	9.661103e-01	-0.1342561824	0.825944889	0.9995992	-1.916519e-01	0.623360311	0.9546035	0.0791465653	4.511392e-01
FH	0.1994419020	1.624283e-02	3.187282e-01	0.0425753832	0.617115760	0.9995992	2.931979e-02	0.368828113	0.9546035	0.0411826375	3.133773e-01
THBS1	-0.4119436236	7.151913e-03	2.615380e-01	0.0419099753	0.789357980	0.9995992	7.264519e-02	0.322240652	0.9546035	0.0571443310	3.609922e-01
RNASE1	0.0153470966	9.758716e-01	9.928688e-01	0.3268167507	0.494738456	0.9995992	-9.590855e-02	0.573329800	0.9546035	-0.4361519729	7.933877e-01
CD55	-0.5866034814	1.250957e-01	5.753431e-01	-0.1897331295	0.617967843	0.9995992	-1.758540e-02	0.518200430	0.9546035	0.1604341171	3.338066e-01
SERPINA6	-0.2265109181	1.716692e-02	3.220842e-01	-0.0912370066	0.361420966	0.9995992	4.129172e-03	0.483331916	0.9546035	-0.1970691649	9.770266e-01
PFKM	0.0960958376	1.722055e-01	6.287166e-01	-0.0129672044	0.855169927	0.9995992	-4.721132e-02	0.743000934	0.9546035	0.0642219500	1.792885e-01
HSP90AB1	0.0630209282	3.759419e-01	7.647336e-01	0.0573996779	0.427128816	0.9995992	1.309010e-02	0.429262878	0.9546035	0.0320646249	3.310874e-01
ELANE	0.2399865559	1.446634e-01	6.111413e-01	-0.1405864922	0.411604344	0.9995992	1.022594e-01	0.273352708	0.9546035	0.2058007231	1.040970e-01
CTSG	0.2885496926	1.730003e-01	6.291668e-01	-0.1869677970	0.380779539	0.9995992	9.553410e-02	0.330101664	0.9546035	0.1628376287	2.268385e-01
HMBS	0.0192075957	8.394899e-01	9.500891e-01	0.0265184736	0.783054905	0.9995992	-4.024806e-02	0.661589151	0.9546035	-0.1019446496	8.426039e-01
ITGA2B	-0.2916340419	2.158767e-02	3.455296e-01	-0.0095547801	0.942114412	0.9995992	-7.826669e-02	0.727973827	0.9546035	-0.0504248339	6.447872e-01
LPA	0.4026675112	9.164902e-02	5.171548e-01	0.0655820927	0.786680020	0.9995992	-3.655386e-01	0.933400815	0.9667703	-0.1653367733	7.490784e-01
PLEK	-0.2410059480	1.411503e-02	3.147917e-01	0.0020962408	0.983963980	0.9995992	-6.829116e-02	0.753675012	0.9546035	-0.0207649971	5.752003e-01
CD14	-0.1731686535	1.833555e-01	6.355609e-01	0.0265559173	0.837297703	0.9995992	-2.074693e-01	0.944753341	0.9707604	-0.2600725913	9.612291e-01
CFH	-0.0757075616	3.131377e-01	7.197712e-01	0.0289365162	0.705552495	0.9995992	1.293857e-02	0.432983108	0.9546035	-0.0738156165	8.358189e-01
FCGR3A	0.0638550340	8.259531e-01	9.430642e-01	0.1202754542	0.674512540	0.9995992	3.553280e-01	0.117722930	0.9546035	0.4020535568	7.519385e-02
VIM	0.2461924703	7.576054e-02	4.893386e-01	-0.1162014659	0.418460915	0.9995992	-2.423182e-02	0.566172192	0.9546035	-0.0035832324	5.105930e-01
SERPINF2	-0.1644184143	2.388704e-02	3.507394e-01	-0.0526111852	0.491507538	0.9995992	-3.502538e-02	0.678559313	0.9546035	-0.1284712051	9.619315e-01
RPS17	-0.2532411417	9.139008e-02	5.171548e-01	-0.1137443915	0.452950076	0.9995992	-1.227202e-01	0.791231406	0.9572213	0.0304242602	4.230234e-01
GNAI3	-0.1253047350	7.920869e-01	9.288162e-01	0.1399156365	0.765919415	0.9995992	-1.775418e-01	0.644852209	0.9546035	0.5050938165	1.440651e-01
ANXA5	0.0215954400	9.306597e-01	9.804276e-01	0.1255002088	0.612775002	0.9995992	4.820730e-01	0.029435053	0.9546035	-0.0305129151	5.471651e-01
RPSA	0.0347497142	9.528033e-01	9.905132e-01	-0.8793365035	0.174135033	0.9995992	1.816152e-01	0.381499158	0.9546035	-0.0745694899	5.511130e-01
ENO2	-0.1039309625	4.456222e-01	8.021362e-01	0.0388341138	0.780020563	0.9995992	-3.515553e-03	0.510044379	0.9546035	-0.0610114844	6.775823e-01
HBQ1	0.1708340536	1.723386e-01	6.287166e-01	0.2289396257	0.063597019	0.9995992	1.064200e-01	0.201727862	0.9546035	0.1320256738	1.667115e-01
DBH	-0.0271209146	9.211725e-01	9.747769e-01	-0.0181695982	0.947277973	0.9995992	3.825209e-02	0.445369574	0.9546035	-0.2449272175	8.170522e-01
GSTP1	-0.1248601731	4.532745e-01	8.045226e-01	-0.1263270211	0.446873021	0.9995992	3.101353e-01	0.030483902	0.9546035	0.0298881312	4.308853e-01
QDPR	0.2138179769	2.667226e-02	3.665885e-01	0.0877489241	0.380835668	0.9995992	2.933615e-02	0.384657762	0.9546035	0.1232716123	1.084898e-01
SPARC	-0.1560159259	4.642259e-01	8.069339e-01	-0.1966124326	0.350024912	0.9995992	-2.442438e-01	0.867659560	0.9572213	-0.3805828175	9.700844e-01
TPM1	0.1552870089	1.031240e-01	5.346166e-01	-0.0143633544	0.881589243	0.9995992	-2.463758e-02	0.600070290	0.9546035	0.0153610335	4.373592e-01
CLTA	0.0983609484	5.411084e-01	8.281031e-01	0.0320659442	0.845063366	0.9995992	-1.068271e-01	0.752826480	0.9546035	-0.0507894617	6.164333e-01
ANXA4	-0.0763333039	7.596682e-01	9.116018e-01	-0.2298947345	0.368813533	0.9995992	1.980716e-01	0.220319776	0.9546035	-0.0839168763	6.421973e-01
CNP	0.0266607785	7.450085e-01	9.009472e-01	-0.0956351133	0.231933925	0.9995992	-6.560869e-02	0.784893519	0.9572213	-0.0415390120	7.027250e-01
HNRNPA1	0.1438936906	4.890906e-01	8.151018e-01	-0.0610076047	0.769066712	0.9995992	-1.614080e-01	0.775983289	0.9572213	-0.1698164256	7.912149e-01
C1S	-0.0502167552	6.056597e-01	8.419469e-01	-0.0994303699	0.298192200	0.9995992	-1.024980e-02	0.541376446	0.9546035	-0.2440957779	9.935253e-01
LTA4H	0.0911089114	1.963101e-01	6.539918e-01	0.0121738019	0.867385105	0.9995992	9.175386e-02	0.097108911	0.9546035	0.0157326507	4.121474e-01
ALDOC	-0.0007704918	9.941007e-01	9.990571e-01	0.0998120336	0.331319170	0.9995992	1.388885e-01	0.094073517	0.9546035	0.2160875399	1.676047e-02
C4A	-0.0332819109	8.683799e-01	9.586081e-01	-0.1063433408	0.589844805	0.9995992	-2.368308e-01	0.883314897	0.9579808	-0.3317062133	9.320026e-01
C4B	0.1378730726	4.769726e-01	8.125882e-01	0.0612318962	0.743156517	0.9995992	-2.246495e-01	0.877404676	0.9579808	-0.2309483553	8.569845e-01
H2AZ1;H2AZ2	-0.0983114248	6.789314e-01	8.610482e-01	-0.2024801593	0.374800019	0.9995992	-1.265306e-01	0.700523624	0.9546035	-0.4575778483	9.651445e-01
H2AC11;H2AC20;H2A(	-0.1228947245	5.241467e-01	8.231924e-01	0.0167679238	0.930628802	0.9995992	-8.126106e-02	0.660853182	0.9546035	0.1280970289	2.565674e-01
UBB;UBC;RPS27A;UB	0.0630724391	4.456456e-01	8.021362e-01	-0.0374979367	0.645027936	0.9995992	1.508146e-01	0.034731664	0.9546035	0.0737786434	1.913924e-01
SAA1	-0.8950760452	4.600043e-01	8.069339e-01	-1.4123893420	0.289195850	0.9995992	-1.317038e+00	0.852122212	0.9572213	-1.3577789975	8.330652e-01
SAA2	-0.7280781643	2.718526e-01	6.940168e-01	-1.0323859141	0.138821143	0.9995992	-7.053095e-01	0.858303702	0.9572213	-0.7239359435	8.405620e-01
HSPA1A;HSPA1B	0.1356502922	8.502239e-02	5.044737e-01	0.0050497510	0.950855059	0.9995992	5.153738e-02	0.259729135	0.9546035	0.1094503838	7.997926e-02
NaN	0.1197095614	5.485775e-01	8.281031e-01	0.0095173221	0.961415609	0.9995992	7.277099e-02	0.357987764	0.9546035	0.2540947375	1.006852e-01
NaN	-0.1898344392	3.149942e-01	7.197712e-01	-0.1786391600	0.360613312	0.9995992	-2.581218e-02	0.553828166	0.9546035	-0.2431095658	8.860918e-01
NaN	1.1596103173	5.938678e-02	4.562264e-01	0.1389742153	0.810066727	0.9995992	7.183597e-01	0.127137174	0.9546035	0.2266439443	3.485270e-01
NaN	0.0348348131	7.768625e-01	9.237942e-01	-0.0175536145	0.887833302	0.9995992	-2.203435e-02	0.570054552	0.9546035	0.0429987069	3.657586e-01
NaN	0.2010067798	5.264264e-01	8.241536e-01	0.4378754482	0.154721616	0.9995992	7.399141e-01	0.012854703	0.9546035	0.1407874888	3.267766e-01
NaN	0.1896144965	2.573027e-01	6.887899e-01	0.1070739165	0.524840613	0.9995992	7.245479e-02	0.337057607	0.9546035	0.0939221954	2.939855e-01
NaN	0.1630814088	2.360513e-01	6.697768e-01	-0.0014676570	0.991760671	0.9995992	-2.491459e-02	0.571022589	0.9546035	0.0059846108	4.839179e-01
IGLC2;IGLC3	0.0123609888	9.468527e-01	9.886748e-01	-0.0148112676	0.936595514	0.9995992	-7.854568e-02	0.663548117	0.9546035	-0.0565312347	6.187971e-01
CALM1;CALM2;CALM	-0.0367739331	5.408309e-01	8.281031e-01	0.0216166907	0.727088751	0.9995992	-1.130010e-01	0.969149763	0.9774190	-0.0571287996	8.283838e-01
SRGN	-0.1358345937	6.095438e-01	8.419469e-01	-0.3122119088	0.248090136	0.9995992	-2.294495e-01	0.803489777	0.9572213	-0.4074360895	9.376042e-01
H1-4	-0.1250536828	3.870356e-01	7.727636e-01	-0.0855909597	0.557496888	0.9995992	-7.451822e-02	0.692641452	0.9546035	-0.0134205821	5.370852e-01
TXN	0.0332035487	6.002618e-01	8.419469e-01	-0.0847446876	0.192195763	0.9995992	-8.518267e-02	0.906883679	0.9613780	-0.0958532217	9.369893e-01
C7	0.0298852299	8.047823e-01	9.350859e-01	-0.0391258121	0.744381556	0.9995992	-1.635743e-01	0.910878365	0.9634873	0.0271203208	4.148666e-01
PRKAR1A	0.0574548099	5.713783e-01	8.344560e-01	0.1153122118	0.261226297	0.9995992	2.907779e-03	0.488758970	0.9546035	0.0671726611	2.754785e-01
UROS	-0.1006076137	6.380122e-01	8.484797e-01	0.0527055541	0.797863742	0.9995992	2.445701e-01	0.135091864	0.9546035	0.0445670917	4.102290e-01
ESD	0.1303310501	5.063496e-02	4.562264e-01	-0.0132384683	0.848477386	0.9995992	6.201133e-02	0.185865815	0.9546035	0.1320324972	2.411951e-02
HSPD1	-0.2331497658	4.991012e-01	8.151018e-01	0.2605465497	0.450256854	0.9995992	2.626156e-01	0.225172008	0.9546035	-0.0879404611	5.916341e-01
CLU	-0.1359250498	1.060881e-01	5.382651e-01	-0.0714853162	0.402371356	0.9995992	-2.290611e-02	0.603523823	0.9546035	-0.1717445890	9.804684e-01
HSPA5	-0.2542825700	2.002269e-01	6.617157e-01	-0.1775777559	0.388301032	0.9995992	1.606418e-02	0.468331985	0.9546035	0.0516760670	3.978904e-01
HSPA8	0.0376962240	6.424538e-01	8.486349e-01	0.0875582648	0.303770728	0.9995992	8.677478e-02	0.143757584	0.9546035	0.1346052866	5.756435e-02
SLC2A1	-0.3057894036	5.244195e-01	8.231924e-01	0.0643425800	0.891171585	0.9995992	-1.787071e-01	0.644681055	0.9546035	0.4921702776	1.469607e-01
SLC2A3;SLC2A14	-1.0924117821	6.069998e-02	4.562264e-01	-0.0447378988	0.934946743	0.9995992	1.935351e-01	0.367586802	0.9546035	0.2947077457	2.987512e-01
EPB41	-0.0358946376	5.338242e-01	8.281031e-01	-0.0244667530	0.670248710	0.9995992	-7.420537e-02	0.905370992	0.9606360	0.0322855598	2.959172e-01
ITGAM	-0.6837302945	7.281331e-02	4.781408e-01	-0.5081671830	0.198541016	0.9995992	-4.577156e-01	0.888128092	0.9579808	-0.5206445684	8.909666e-01

**MBL2**	0.6067574847	1.460993e-01	6.145529e-01	0.4012762315	0.331047693	0.9995992	-2.001593e-01	0.681902920	0.9546035	0.2924340611	2.568950e-01
**SPTB**	0.1053300663	2.767940e-01	6.983553e-01	0.0640089111	0.503826629	0.9995992	-7.730738e-02	0.782920413	0.9572213	0.0942554514	1.569549e-01
**LAMP1**	-0.5637739832	1.811651e-01	6.347267e-01	-0.1905192693	0.661350292	0.9995992	1.131651e-01	0.393941119	0.9546035	0.2378762963	2.941249e-01
**G6PD**	0.0539872698	4.051279e-01	7.850184e-01	0.0437665648	0.511562158	0.9995992	9.183032e-03	0.444985144	0.9546035	0.0763340699	1.328158e-01
**MTHFD1**	-0.0745107566	4.788512e-01	8.125882e-01	-0.1199839924	0.251367909	0.9995992	2.063238e-02	0.423443527	0.9546035	-0.1189227157	8.747956e-01
**CETP**	-0.0250524837	8.962413e-01	9.687085e-01	-0.1790022116	0.351126646	0.9995992	3.275845e-01	0.045226209	0.9546035	-0.0954349359	6.872242e-01
**EPX**	-0.0972330552	6.218368e-01	8.419469e-01	-0.1276360637	0.512776105	0.9995992	-1.594683e-01	0.788065808	0.9572213	0.0104667670	4.788457e-01
**SCGB1A1**	1.2930310495	4.126340e-01	7.920435e-01	1.4339091634	0.351174691	0.9995992	-9.371139e-01	0.727587288	0.9546035	-1.6577290404	8.272087e-01
**ADH5**	-0.0917257455	3.763847e-01	7.647336e-01	-0.0115365689	0.914503651	0.9995992	-5.621296e-02	0.703696570	0.9546035	0.0168148767	4.385016e-01
**PRPS2**	-0.5144567240	1.392358e-01	6.012201e-01	0.2141575723	0.529840169	0.9995992	-1.267741e-01	0.642733718	0.9546035	0.1695318967	3.059008e-01
**HARS1**	0.2116368769	4.935227e-01	8.151018e-01	0.1951940911	0.535987446	0.9995992	3.493695e-01	0.125949212	0.9546035	0.5117833212	5.123141e-02
**F5**	-0.1577887704	2.851315e-01	6.987920e-01	-0.1544061177	0.310333860	0.9995992	-1.449132e-02	0.538111575	0.9546035	-0.1079767766	7.619324e-01
**ANXA3**	-0.6540582944	3.713104e-01	7.647280e-01	-0.7226298188	0.358880053	0.9995992	-9.196776e-01	0.893193593	0.9579808	-0.8808255747	8.491691e-01
**RNASE3**	-0.3633229489	1.524541e-01	6.224838e-01	-0.0699696680	0.785669675	0.9995992	-2.569371e-01	0.843340599	0.9572213	-0.1653280922	7.348239e-01
**ACTN1**	-0.0936782958	4.573970e-01	8.069339e-01	0.1030641344	0.410753663	0.9995992	2.329297e-02	0.428481818	0.9546035	0.1111260527	1.825345e-01
**MYL4**	0.1071012235	5.906854e-01	8.419469e-01	0.0121344003	0.948942331	0.9995992	3.587713e-01	0.039998543	0.9546035	0.1028727546	2.830324e-01
**PEPD**	-0.0322548069	7.697474e-01	9.192799e-01	0.1404269923	0.230374702	0.9995992	2.231507e-02	0.419695147	0.9546035	0.1136398583	1.702837e-01
**COX4I1**	-0.9921140743	1.681023e-01	6.287166e-01	0.0418662360	0.952487217	0.9995992	-1.920924e-01	0.607582871	0.9546035	0.4623518516	2.584397e-01
**GP1BB**	-0.6401482789	7.019804e-02	4.698433e-01	0.0845658078	0.813703224	0.9995992	-1.421528e-01	0.656728934	0.9546035	0.3006790113	2.088184e-01
**LAMP2**	-0.5259289791	2.342588e-01	6.697768e-01	-0.1515662419	0.735967337	0.9995992	-2.887038e-01	0.736433613	0.9546035	-0.5670966268	9.005603e-01
**RNH1**	0.1748240441	2.134508e-02	3.455296e-01	-0.0237326167	0.761033359	0.9995992	-2.567825e-02	0.625580968	0.9546035	0.0713704036	1.801015e-01
**BMP1;TLL2**	-0.1315783652	2.315347e-01	6.697768e-01	-0.0758997734	0.498087532	0.9995992	-1.648482e-01	0.934841531	0.9667703	-0.1549856754	9.146415e-01
**ICAM2**	-0.2377638650	3.217275e-01	7.225946e-01	0.0830341735	0.721845489	0.9995992	-2.862555e-01	0.887664615	0.9579808	-0.0377874709	5.622092e-01
**EEF2**	0.0759157212	6.719341e-01	8.576613e-01	0.1571456890	0.366597310	0.9995992	-1.862277e-02	0.540605252	0.9546035	0.1697190418	1.680025e-01
**PDIA4**	4.6203069898	1.351801e-01	5.932126e-01	-0.2145581293	0.902582731	0.9995992	4.816101e-01	0.394014741	0.9546035	-0.0423107177	5.094535e-01
**C6**	-0.0785439366	5.294156e-01	8.277370e-01	-0.0471716148	0.711853866	0.9995992	1.108261e-01	0.193079492	0.9546035	0.0353474994	3.885821e-01
**TPT1**	0.0672663854	5.668562e-01	8.344560e-01	-0.1507657656	0.211205928	0.9995992	1.078364e-01	0.181983514	0.9546035	-0.0935163367	7.672227e-01
**ALAD**	0.1624822225	3.732572e-02	4.123271e-01	0.0557328576	0.503545925	0.9995992	8.080956e-02	0.156573037	0.9546035	0.1310950655	6.057078e-02
**PRG2**	-0.0416266138	9.627043e-01	9.920806e-01	0.1961605474	0.819672238	0.9995992	3.394880e-01	0.354744635	0.9546035	0.0166411540	4.920698e-01
**LCP1**	0.0041821970	9.613929e-01	9.920806e-01	-0.0251035027	0.772651548	0.9995992	-4.272329e-02	0.685118863	0.9546035	0.0284820666	3.685783e-01
**APEH**	-0.1307088366	3.019807e-01	7.082166e-01	0.0123959498	0.924879513	0.9995992	9.051104e-02	0.242033428	0.9546035	0.1344828483	1.628017e-01
**ETFA**	0.2251713816	2.860200e-01	6.987920e-01	0.2270151643	0.276799415	0.9995992	1.441551e-01	0.253033731	0.9546035	0.0406616888	4.252993e-01
**CD59**	-0.4029429260	3.476438e-01	7.479692e-01	0.0843481091	0.845049360	0.9995992	7.889959e-02	0.427510900	0.9546035	0.4925818952	1.280442e-01
**SELL**	0.0454741071	8.166387e-01	9.406531e-01	-0.2122194472	0.276912257	0.9995992	-2.495727e-02	0.551199349	0.9546035	-0.1739842601	8.014846e-01
**MIF**	-0.0992424934	3.323674e-01	7.329444e-01	-0.0601726709	0.568871340	0.9995992	1.349045e-01	0.092239161	0.9546035	0.1549855003	7.926000e-02
**CD99**	-1.2727464932	2.150918e-01	6.680462e-01	-1.3194204622	0.223569331	0.9995992	-1.086116e+00	0.857524295	0.9572213	-1.3114962973	8.727714e-01
**PRKCSH**	-0.4789454092	1.063199e-01	5.382651e-01	-0.2602827953	0.399618310	0.9995992	-1.863972e-02	0.524814766	0.9546035	0.0280881618	4.623447e-01
**HCLS1**	0.1637335041	3.528271e-01	7.498135e-01	0.1392140053	0.426765961	0.9995992	-3.698004e-01	0.981351015	0.9860085	-0.1267141043	7.529264e-01
**FDPS**	0.1899647343	5.131783e-02	4.562264e-01	0.1732376650	0.095374685	0.9995992	8.130152e-02	0.209613689	0.9546035	0.1271981147	1.196966e-01
**AKR1A1**	0.1476184006	3.227834e-01	7.225946e-01	-0.0319516999	0.829250207	0.9995992	6.980777e-02	0.324434952	0.9546035	0.0570790288	3.542769e-01
**PKM**	0.0700156346	5.615374e-01	8.323189e-01	0.0842395177	0.474546033	0.9995992	-7.752788e-02	0.737648236	0.9546035	-0.0183061180	5.624373e-01
**HSP90B1**	-0.3574952116	1.006901e-01	5.314990e-01	-0.2260487820	0.321086234	0.9995992	1.234331e-01	0.289753198	0.9546035	0.1748339280	2.037289e-01
**IDE**	0.2741196803	1.171972e-02	3.084914e-01	0.0424268820	0.702277929	0.9995992	-6.169686e-02	0.711370998	0.9546035	0.1623382199	6.276234e-02
**GP9**	-0.9295199845	4.151300e-02	4.250863e-01	0.1575785273	0.722386375	0.9995992	6.247226e-02	0.444681979	0.9546035	0.1978679806	3.295142e-01
**MMP9**	0.5658747885	2.035722e-02	3.455296e-01	-0.0490008635	0.841929186	0.9995992	-3.031638e-02	0.548130966	0.9546035	0.2404145462	1.581118e-01
**DARS1**	0.0558762275	4.453179e-01	8.021362e-01	0.0953031274	0.187955433	0.9995992	-5.195629e-02	0.761989634	0.9561271	0.0972122153	9.312136e-02
**UQCRB**	-0.1802744706	1.222581e-01	5.724507e-01	-0.0368237006	0.759220402	0.9995992	-5.707501e-03	0.519035163	0.9546035	-0.1107849369	8.226265e-01
**AKR1B1**	0.0640945175	6.650526e-01	8.576613e-01	-0.1885079938	0.215603444	0.9995992	-4.537372e-03	0.512071310	0.9546035	-0.1053089102	7.497351e-01
**ANPEP**	-0.3652785368	5.118563e-01	8.164833e-01	-0.5506067740	0.322369719	0.9995992	5.930963e-02	0.458636664	0.9546035	-0.7175539637	8.988514e-01
**RAC2**	0.0072684157	9.781102e-01	9.932356e-01	0.1545430963	0.556495910	0.9995992	-2.808892e-02	0.541126970	0.9546035	0.1742381931	2.551103e-01
**CPN1**	-0.1954868577	7.035725e-02	4.698433e-01	-0.1585013577	0.152317714	0.9995992	-4.326080e-02	0.652487465	0.9546035	-0.2065386735	9.647221e-01
**GSPT1**	0.1888516835	1.570570e-01	6.224838e-01	0.0465501977	0.728044215	0.9995992	8.592668e-02	0.266179357	0.9546035	0.0546487591	3.426639e-01
**EZR**	0.1570673993	3.106801e-01	7.192836e-01	-0.1007759141	0.507119749	0.9995992	8.481455e-02	0.296001967	0.9546035	-0.1376299756	8.105757e-01
**UCHL3**	-0.0264598987	7.316882e-01	8.933254e-01	-0.0155301796	0.842917853	0.9995992	-3.952972e-02	0.690575427	0.9546035	0.0450682515	2.813045e-01
**NME1**	0.0939716539	2.294221e-01	6.697768e-01	0.0142124818	0.857528725	0.9995992	-1.008351e-01	0.898390804	0.9579808	0.0075155679	4.617273e-01
**IGLL1**	0.1910273624	2.623314e-01	6.900429e-01	-0.0278155740	0.868447453	0.9995992	-2.857126e-02	0.565473553	0.9546035	0.0720640856	3.315895e-01
**DSP**	0.0977312502	8.732016e-01	9.614924e-01	-0.0240236364	0.969123954	0.9995992	-3.886989e-01	0.733189859	0.9546035	-0.1582955180	5.979201e-01
**CD44**	-0.2310199877	4.814131e-01	8.129736e-01	0.0388381433	0.906477477	0.9995992	-1.000159e-01	0.617832727	0.9546035	0.3467669416	1.367722e-01
**NQO2**	-0.4121446103	1.317055e-01	5.830557e-01	0.3166719615	0.253734767	0.9995992	2.157790e-01	0.219705141	0.9546035	0.3108891367	1.385822e-01
**CBR1**	-0.1097203233	2.258702e-01	6.697768e-01	0.0095622585	0.918557377	0.9995992	-1.074953e-02	0.546088521	0.9546035	0.0374960171	3.460632e-01
**ANK1**	0.0338725770	5.781204e-01	8.363994e-01	0.0402187724	0.515893242	0.9995992	2.287892e-02	0.357305781	0.9546035	0.1323222512	1.935691e-02
**H1-5**	0.0171644247	9.064998e-01	9.710488e-01	-0.0216223987	0.880592721	0.9995992	5.852169e-02	0.347315002	0.9546035	0.0037736400	4.896037e-01
**H1-3**	-0.2657635933	2.678937e-01	6.940168e-01	-0.1063404396	0.659088331	0.9995992	1.655333e-01	0.250003881	0.9546035	0.1519922646	2.602953e-01
**EPB42**	-0.0450044321	6.726329e-01	8.576613e-01	0.0353398501	0.739975863	0.9995992	9.738624e-03	0.463918940	0.9546035	0.1297757282	1.145566e-01
**CD36**	-0.7914375443	8.084960e-02	5.027637e-01	0.3188743297	0.453897601	0.9995992	2.733828e-01	0.273596983	0.9546035	0.1804009926	3.291950e-01
**FAH**	0.1266635768	4.937114e-01	8.151018e-01	-0.0160234400	0.932494611	0.9995992	-5.556769e-02	0.620738193	0.9546035	-0.1362714213	7.677228e-01
**STMN1**	-0.4135975867	3.972017e-01	7.802736e-01	-0.5307091862	0.316129073	0.9995992	-4.851525e-01	0.839129810	0.9572213	-0.5078112108	8.104616e-01
**YBX3**	-0.3064767186	3.608930e-01	7.553917e-01	-0.1447201173	0.671973679	0.9995992	1.321528e-01	0.348194021	0.9546035	0.1591889251	3.247046e-01
**GOT1**	0.0860258752	1.932848e-01	6.539918e-01	0.0160519957	0.818657069	0.9995992	5.916238e-02	0.191849903	0.9546035	0.0728853172	1.537158e-01
**BPI**	0.3730513962	2.063850e-01	6.658594e-01	0.0491214439	0.867477045	0.9995992	2.573697e-01	0.196066469	0.9546035	0.6935779784	8.276548e-03
**PRKACA**	-0.0272937621	7.787512e-01	9.241807e-01	0.0926937934	0.338867246	0.9995992	-9.355290e-03	0.538115217	0.9546035	0.1765156679	3.600314e-02
**CTPS1**	0.1094024855	2.716763e-01	6.940168e-01	-0.0004536900	0.996438396	0.9995992	1.029109e-01	0.153344964	0.9546035	-0.0008703495	5.034540e-01
**PFKL**	0.0690158752	2.804170e-01	6.987920e-01	0.0390160989	0.535709127	0.9995992	4.650333e-02	0.236305458	0.9546035	0.0497719002	2.134344e-01
**CR1**	0.3160529034	5.897783e-01	8.419469e-01	0.2534419744	0.657313959	0.9995992	-6.112248e-01	0.844084557	0.9572213	0.5702224363	1.477132e-01
**LGALS3**	0.0924308688	5.038141e-01	8.151018e-01	-0.0634573520	0.656575716	0.9995992	2.007997e-01	0.071643460	0.9546035	0.0823756227	2.817733e-01
**IGFBP3**	0.2616194608	7.127656e-01	8.803438e-01	2.0529608467	0.010596477	0.9995992	6.157116e-01	0.198190426	0.9546035	-0.1277924264	5.664298e-01
**PSMC3**	0.0870908547	2.447961e-01	6.729045e-01	-0.1105974633	0.152704184	0.9995992	8.617042e-02	0.129582799	0.9546035	0.0380342335	3.243392e-01
**TCP1**	-0.0636841235	4.465361e-01	8.021362e-01	0.0151082778	0.859504730	0.9995992	4.829910e-02	0.283568406	0.9546035	0.0986226859	1.202857e-01
**IGFBP2**	0.2491318162	3.791319e-01	7.647336e-01	0.1204710976	0.654086262	0.9995992	-3.012028e-01	0.853344412	0.9572213	-0.4124687297	9.039916e-01
**RPL7**	0.3460654506	3.747614e-01	7.647336e-01	-0.5175230870	0.179594911	0.9995992	-8.508830e-01	0.982911501	0.9862491	-0.6175369458	9.483020e-01
**VCL**	-0.2993853321	9.031569e-03	2.737260e-01	-0.0063557123	0.957917493	0.9995992	-1.113700e-01	0.827592155	0.9572213	-0.1109252384	8.185438e-01
**LBP**	0.2764321900	1.945314e-01	6.539918e-01	-0.1589225891	0.460055503	0.9995992	-2.625234e-01	0.885538642	0.9579808	-0.2572109631	8.715127e-01
**RHCE**	-0.3046142010	5.739532e-01	8.344560e-01	-0.0352014476	0.947940904	0.9995992	-4.594099e-02	0.533602890	0.9546035	0.6643630842	1.074754e-01
**PGAM1**	0.1140348643	8.336882e-02	5.027637e-01	-0.0014048430	0.982923039	0.9995992	3.854306e-02	0.287710138	0.9546035	0.0447907258	2.475594e-01
**CD58**	-0.3074973410	1.886872e-01	6.427328e-01	0.0034023066	0.988537803	0.9995992	7.508781e-02	0.374296327	0.9546035	0.0088170104	4.851759e-01
**VCAM1**	0.7682046809	1.141149e-01	5.517720e-01	0.4788237954	0.321954735	0.9995992	1.537110e-01	0.378825868	0.9546035	0.8625741205	3.849822e-02
**HK1**	0.0490069397	6.271106e-01	8.423236e-01	-0.0011636159	0.990996805	0.9995992	-5.144663e-02	0.690743820	0.9546035	-0.0545381806	6.924566e-01
**ORM2**	-0.0776652162	5.797652e-01	8.377536e-01	0.0870508996	0.522084306	0.9995992	1.002709e-01	0.234582870	0.9546035	-0.0700538933	6.810796e-01
**ITIH2**	-0.1212960425	2.153347e-01	6.680462e-01	-0.1379282253	0.163308355	0.9995992	-3.247471e-02	0.625937461	0.9546035	-0.1956366005	9.744686e-01
**ITIH1**	-0.1404827150	1.661053e-01	6.287166e-01	-0.0392335290	0.706544862	0.9995992	-9.137387e-02	0.810360150	0.9572213	-0.1587083291	9.408781e-01
**ATP2B1**	-0.0388284917	9.266799e-01	9.771058e-01	0.7035325416	0.088154295	0.9995992	2.227463e-01	0.304925952	0.9546035	0.4777220265	1.198825e-01
**EIF2S2**	-0.1397963681	2.711548e-01	6.940168e-01	-0.1126681508	0.386358574	0.9995992	-6.876378e-02	0.702494060	0.9546035	-0.0755182477	7.141907e-01
**ANXA7**	0.0256106766	6.920997e-01	8.660792e-01	-0.0033689535	0.956996448	0.9995992	2.928671e-02	0.325733343	0.9546035	-0.0412356688	7.463547e-01
**AZU1**	0.2899750622	1.270395e-01	5.775504e-01	-0.1052046488	0.585185586	0.9995992	-1.580222e-01	0.790869838	0.9572213	-0.0680287816	6.349419e-01
**ALOX5AP**	-0.9130470090	2.310838e-01	6.697768e-01	0.1016786680	0.890323382	0.9995992	1.059303e-01	0.443435042	0.9546035	0.7116440089	1.746979e-01
**RAB6A**	-0.0899569512	4.979923e-01	8.151018e-01	0.0915333611	0.481434149	0.9995992	-5.042983e-02	0.646707820	0.9546035	0.0635837591	2.986659e-01
**PSMB1**	0.0224060501	7.751700e-01	9.227099e-01	-0.0510340775	0.529644728	0.9995992	1.305738e-02	0.434281108	0.9546035	0.0536986880	2.621230e-01
**PZP**	1.1907996280	6.124445e-02	4.562264e-01	0.8900950644	0.141120320	0.9995992	-3.119389e-01	0.696547785	0.9546035	0.9196992605	5.810049e-02
**CAST**	-0.0855743699	1.918989e-01	6.517946e-01	-0.0032148146	0.960936757	0.9995992	3.438263e-02	0.307133249	0.9546035	0.0493714916	2.264144e-01
**C4BPB**	-0.0608665034	6.316458e-01	8.451005e-01	-0.1043637686	0.423816533	0.9995992	-3.823731e-02	0.616058452	0.9546035	-0.2186350628	9.480680e-01
**GSTM3**	0.4459046781	2.830001e-01	6.987920e-01	0.7813678506	0.051139600	0.9995992	-2.418167e-01	0.722622503	0.9546035	0.8692601297	1.228050e-02
**ATP6V1B2**	0.1654880193	3.687663e-02	4.112092e-01	-0.0505476514	0.539127713	0.9995992	1.576202e-01	0.027504373	0.9546035	-0.0131063289	5.634603e-01
**CSRP1**	0.7595661641	2.337325e-01	6.697768e-01	1.6604083498	0.014372595	0.9995992	9.839792e-01	0.062916652	0.9546035	1.3261702633	2.319476e-02
**FLNA**	-0.1078501226	3.069870e-01	7.185319e-01	-0.0333479545	0.756543775	0.9995992	-2.027157e-02	0.574614694	0.9546035	-0.0663260489	7.277446e-01
**ACO1**	0.1309628057	2.081839e-01	6.658594e-01	-0.0582580617	0.578314803	0.9995992	5.863181e-03	0.478209445	0.9546035	0.0441982837	3.473996e-01
**CD9**	-0.5339941151	1.350610e-02	3.121263e-01	0.0152143245	0.944119206	0.9995992	-1.182958e-01	0.702695931	0.9546035	0.0995803355	3.244966e-01
**TGM2**	-0.0399148506	6.497887e-01	8.496131e-01	-0.1235131744	0.167439026	0.9995992	-2.542799e-02	0.611160098	0.9546035	-0.1029298529	8.670633e-01
**PCMT1**	0.1170684439	4.943645e-02	4.562264e-01	-0.0287678171	0.639339763	0.9995992	5.120317e-02	0.203591703	0.9546035	0.0197069059	3.777445e-01
**GART**	-0.1326317238	6.086194e-01	8.419469e-01	-0.1301206150	0.625756131	0.9995992	9.521874e-02	0.358899011	0.9546035	-0.1046855118	6.475670e-01
**PAICS**	-0.0574412022	5.722520e-01	8.344560e-01	0.1560128569	0.121103488	0.9995992	-4.746775e-02	0.675877537	0.9546035	0.0886762068	2.004683e-01
**ACHE**	0.0770598373	7.967286e-01	9.305665e-01	0.2681844261	0.361511163	0.9995992	4.017718e-01	0.094844791	0.9546035	0.1087315016	3.509737e-01
**SCP2**	-0.0745266710	5.090615e-01	8.151018e-01	0.0262419787	0.819519256	0.9995992	5.132266e-02	0.325790486	0.9546035	0.1331875510	1.288013e-01
**UBA1**	-0.1836889212	1.627050e-03	1.319384e-01	-0.0488469521	0.435309943	0.9995992	5.470743e-02	0.186887795	0.9546035	0.0076849006	4.547429e-01
**GPX3**	-0.1801426129	1.471131e-01	6.165020e-01	-0.0317708915	0.801098181	0.9995992	1.061834e-01	0.203530523	0.9546035	0.0610737050	3.157741e-01
**NME2**	0.0538609368	3.773466e-01	7.647336e-01	-0.0050395259	0.935762127	0.9995992	7.539837e-02	0.114567906	0.9546035	0.0188853440	3.831383e-01
**HNRNPA2B1**	0.0336169552	8.388799e-01	9.500891e-01	-0.1340401804	0.420322447	0.9995992	6.909835e-02	0.339848141	0.9546035	-0.1171745264	7.497766e-01
**CPN2**	-0.2190718204	5.816873e-02	4.562264e-01	-0.1707986523	0.149652301	0.9995992	-3.092953e-02	0.603124837	0.9546035	-0.2151965362	9.628078e-01
**PROZ**	-0.7697076876	3.153553e-01	7.197712e-01	-0.8007850761	0.327232092	0.9995992	-5.036885e-01	0.747465200	0.9546035	-0.7789352546	8.123067e-01
**IGHV1-2**	0.3842463791	1.043511e-01	5.362738e-01	-0.2498047540	0.317450657	0.9995992	-1.412955e-01	0.715488240	0.9546035	0.0012385648	4.976770e-01
**FBLN1**	-0.2121771590	1.129363e-01	5.517720e-01	-0.1587501082	0.229660469	0.9995992	-7.713473e-02	0.714236098	0.9546035	-0.1640404549	8.742720e-01
**TUBG1;TUBG2**	0.8242129432	6.236364e-02	4.578498e-01	0.4419009335	0.282857868	0.9995992	-7.147078e-01	0.948124089	0.9707604	0.1761276357	3.269790e-01
**KEL**	-0.5040331881	2.711392e-01	6.940168e-01	0.1102611631	0.807463169	0.9995992	4.509684e-02	0.461051380	0.9546035	0.5232042873	1.233721e-01
**PPIB**	-0.0895348971	3.100880e-01	7.192836e-01	-0.0216766442	0.812818181	0.9995992	7.666113e-03	0.465994356	0.9546035	0.0884340012	1.682774e-01
**WARS1**	0.1103541866	1.877484e-01	6.427328e-01	-0.0039434214	0.962833973	0.9995992	3.264425e-02	0.351796082	0.9546035	-0.0294919334	6.348679e-01
**AHCY**	-0.2147067250	7.284848e-03	2.615380e-01	-0.0462809965	0.585254434	0.9995992	-2.922631e-02	0.636784968	0.9546035	0.0106464417	4.512303e-01
**CFL1**	-0.0779337691	4.641883e-01	8.069339e-01	0.1124658008	0.309336190	0.9995992	1.657684e-02	0.440162026	0.9546035	0.1535813240	7.872143e-02
**EIF4B**	-0.0314361642	6.190065e-01	8.419469e-01	-0.0443392096	0.488106736	0.9995992	3.121650e-02	0.314398141	0.9546035	-0.0178514849	6.093120e-01
**ATP2B4**	-0.2391830666	5.089588e-01	8.151018e-01	-0.0781423830	0.828095296	0.9995992	-7.756660e-02	0.584213863	0.9546035	0.2782444501	2.167791e-01
**DTYMK**	-0.0115774061	8.988871e-01	9.703055e-01	-0.0722142862	0.446377331	0.9995992	-4.086302e-02	0.669848512	0.9546035	-0.0760923822	7.759100e-01
**RRM1**	0.5790246451	4.001490e-01	7.830730e-01	0.2132738018	0.739360087	0.9995992	8.914647e-01	0.112677630	0.9546035	0.4049224205	2.569324e-01
**PRTN3**	0.0947769970	6.224723e-01	8.419469e-01	-0.2039487483	0.299063558	0.9995992	-6.269220e-02	0.623965424	0.9546035	0.0258594398	4.461638e-01
**TBXAS1**	-0.2009480045	6.932121e-01	8.660792e-01	-0.1597701176	0.747022175	0.9995992	3.151734e-01	0.267809396	0.9546035	0.6169719854	1.046751e-01
**IGFBP6**	-0.0322480543	9.221972e-01	9.749884e-01	-0.0816058975	0.808931973	0.9995992	3.071994e-01	0.183355375	0.9546035	0.2873298584	1.888905e-01
**IGFBP5**	-0.8997362981	3.475371e-02	4.027342e-01	-0.5764474920	0.177199383	0.9995992	-8.410117e-02	0.578688438	0.9546035	-0.6472490219	9.294617e-01
**ACP1**	0.0207942433	8.114565e-01	9.384947e-01	0.0079796238	0.928026902	0.9995992	4.653927e-02	0.297765229	0.9546035	0.0438691622	3.165595e-01
**TNC**	-0.0498351168	7.888198e-01	9.269293e-01	-0.0387259654	0.839436717	0.9995992	-7.560395e-02	0.655842769	0.9546035	-0.2080099591	8.497573e-01
**CDK2**	-0.0955187088	4.519121e-01	8.045226e-01	0.0803057869	0.520372783	0.9995992	9.569937e-02	0.231998797	0.9546035	0.1411592296	1.311470e-01
**AZGP1**	0.0229264767	8.965881e-01	9.687085e-01	0.1387992557	0.410312097	0.9995992	-1.377926e-01	0.778818547	0.9572213	-0.0920389302	6.902032e-01
**MPST**	0.0877408726	2.254727e-01	6.697768e-01	0.0630943351	0.400168195	0.9995992	-2.349153e-02	0.623175261	0.9546035	0.0808646228	1.249921e-01

RPS12	0.2160733461	1.661508e-01	6.287166e-01	0.0571793306	0.710670779	0.9995992	3.309335e-02	0.418365305	0.9546035	-0.0942243221	7.303717e-01
DNAJB1	0.0740765778	4.842210e-01	8.129736e-01	-0.0093133227	0.929934164	0.9995992	9.301221e-02	0.193973861	0.9546035	0.1789230583	4.022675e-02
DNAJB2	-0.0001709445	9.979405e-01	9.990571e-01	-0.0537228117	0.410712708	0.9995992	6.489804e-02	0.166920425	0.9546035	-0.0065956764	5.373022e-01
ATP5F1A	-0.3622197115	9.837501e-02	5.314990e-01	0.0969489434	0.661729437	0.9995992	8.396333e-02	0.353429293	0.9546035	0.3118405772	7.552365e-02
PSMA1	0.1328307004	5.731460e-02	4.562264e-01	-0.0140480842	0.844800570	0.9995992	-1.291404e-01	0.964765074	0.9774190	-0.0308320771	6.663257e-01
PSMA2	0.0555234051	6.051073e-01	8.419469e-01	0.0535915241	0.610353759	0.9995992	9.900803e-02	0.184970292	0.9546035	0.1609650696	6.189816e-02
PSMA3	0.1036385360	1.050540e-01	5.375490e-01	-0.0587801944	0.377287567	0.9995992	-2.975876e-02	0.672201137	0.9546035	-0.0172147543	6.020244e-01
PSMA4	0.0315326894	6.787933e-01	8.610482e-01	-0.0866111870	0.264560315	0.9995992	2.403888e-02	0.376033063	0.9546035	0.0067343282	4.657562e-01
S100P	0.1409891090	5.421083e-01	8.281031e-01	-0.1916403304	0.392047851	0.9995992	-2.583478e-01	0.865984970	0.9572213	-0.0074338154	5.134483e-01
MSN	-0.0101450502	8.682258e-01	9.586081e-01	0.1034482345	0.101766671	0.9995992	2.677371e-02	0.331201779	0.9546035	0.1173844690	3.402769e-02
S100A4	0.0765848598	6.368983e-01	8.484797e-01	-0.1266841051	0.424930137	0.9995992	-2.702043e-01	0.950934531	0.9714819	0.1196412248	2.244612e-01
HMGB2	0.0862040067	6.235042e-01	8.419469e-01	0.0543471454	0.765062212	0.9995992	8.851705e-02	0.310220005	0.9546035	0.1668267662	1.815242e-01
PTBP1	-0.0198950340	8.763286e-01	9.635539e-01	-0.0373526752	0.774040519	0.9995992	-5.268643e-02	0.673035758	0.9546035	0.0196538826	4.427287e-01
EEF1G	0.0719345674	7.986700e-01	9.309940e-01	-0.0582277790	0.833296170	0.9995992	-1.864996e-01	0.748181843	0.9546035	-0.0475390912	5.674411e-01
MST1	0.0726640321	7.167175e-01	8.824584e-01	-0.1334488437	0.507695388	0.9995992	-2.619741e-01	0.901018705	0.9579808	-0.1187888826	7.204238e-01
STOM	-0.6106067065	3.002441e-01	7.059294e-01	0.1296801748	0.823810313	0.9995992	2.833033e-02	0.480640351	0.9546035	0.6949477199	1.230980e-01
PON1	-0.2599124415	9.128232e-02	5.171548e-01	-0.2209039628	0.153625388	0.9995992	3.696848e-03	0.490739203	0.9546035	-0.1914075777	9.008220e-01
YWHAQ	-0.0376114130	7.041419e-01	8.724274e-01	0.0543497603	0.582119833	0.9995992	-1.085726e-02	0.542997357	0.9546035	-0.0079426795	5.325462e-01
CALR	-0.4114765827	1.286725e-01	5.792482e-01	-0.2972982879	0.294794643	0.9995992	1.197670e-01	0.332003436	0.9546035	0.0719751400	3.988782e-01
CANX	-0.5359919225	2.761775e-01	6.983553e-01	-0.1627789178	0.740657715	0.9995992	5.554457e-02	0.455315734	0.9546035	0.5041057477	1.473489e-01
CFP	-0.3305034223	2.289687e-02	3.469756e-01	0.1007321145	0.519822926	0.9995992	-9.117816e-02	0.744541117	0.9546035	-0.0019087448	5.046997e-01
PSMA5	0.0762833431	2.134435e-01	6.680462e-01	0.0034591689	0.956013477	0.9995992	-7.910994e-02	0.899887807	0.9579808	-0.0008091022	5.050077e-01
PSMB4	0.1034393030	2.200923e-01	6.697768e-01	-0.1155107702	0.170191130	0.9995992	3.485771e-02	0.343708997	0.9546035	0.0360491420	3.350362e-01
PSMB6	0.0809705653	2.836943e-01	6.987920e-01	-0.1105863726	0.150084466	0.9995992	-8.195645e-02	0.851674326	0.9572213	-0.1035777995	9.219502e-01
PSMB5	0.1226798312	1.020730e-01	5.314990e-01	-0.0441420014	0.573115236	0.9995992	1.414610e-02	0.428027253	0.9546035	0.0121007984	4.405662e-01
TMOD1	0.1518284014	2.447524e-02	3.507394e-01	0.0185613699	0.787677931	0.9995992	1.695788e-03	0.490481542	0.9546035	0.0828706566	1.125461e-01
MAPK1	0.2006050718	5.336629e-02	4.562264e-01	0.1544633724	0.138411755	0.9995992	1.092474e-02	0.459449249	0.9546035	0.0614171448	2.835904e-01
GCA	0.1209871765	2.873755e-01	6.987920e-01	-0.0069818411	0.952190554	0.9995992	-6.278434e-02	0.705054236	0.9546035	-0.0261531608	5.899319e-01
LAP3	0.1789466918	5.501934e-01	8.284440e-01	0.6188621281	0.031797917	0.9995992	3.137511e-01	0.155950140	0.9546035	0.6308592324	1.096932e-02
TPP2	0.0957081072	1.761156e-01	6.320739e-01	0.0798671713	0.269762475	0.9995992	-1.963136e-02	0.606926571	0.9546035	0.1216133210	5.294922e-02
TKT	0.1190737785	2.385576e-01	6.725635e-01	0.1200211679	0.230466811	0.9995992	-2.164813e-02	0.583146667	0.9546035	0.0594885410	2.771308e-01
SERPINA4	-0.2511565262	2.156953e-02	3.455296e-01	0.1095653881	0.338014431	0.9995992	4.579773e-02	0.345575996	0.9546035	0.0412113428	3.563453e-01
EEF1D	-0.9862767255	8.396897e-02	5.027637e-01	0.8731775554	0.113154523	0.9995992	1.035517e+00	0.037408813	0.9546035	0.7903632339	7.128375e-02
AQP1	-0.6231562313	4.031736e-01	7.844497e-01	-0.0514458981	0.945505179	0.9995992	-2.697268e-01	0.643171295	0.9546035	0.4472444727	2.879721e-01
PRDX6	0.0191075678	8.162069e-01	9.406531e-01	0.1609518131	0.041654291	0.9995992	1.975536e-01	0.008125859	0.9546035	0.2905915448	4.170238e-05
BLVRB	-0.0454180522	5.093276e-01	8.151018e-01	-0.0072337110	0.915992630	0.9995992	-2.041824e-02	0.612677411	0.9546035	0.0668792217	1.717282e-01
PRDX5	0.0807318863	3.731736e-01	7.647336e-01	-0.0139996777	0.877239414	0.9995992	-1.478773e-01	0.947903090	0.9707604	0.0506063904	2.824350e-01
DDT	0.0510721649	5.187278e-01	8.216271e-01	0.0723674856	0.374518337	0.9995992	-7.620372e-02	0.826381841	0.9572213	0.0795358321	1.637368e-01
CMPK1	0.1435653159	2.227283e-01	6.697768e-01	-0.1449531785	0.214271644	0.9995992	6.989978e-02	0.279960867	0.9546035	-0.0767730337	7.354494e-01
PEBP1	0.0990972599	4.110624e-02	4.250863e-01	0.0097963491	0.841561930	0.9995992	7.122000e-02	0.078075154	0.9546035	0.0653412647	8.885153e-02
PDIA3	-0.4407181436	1.220735e-02	3.084914e-01	-0.1149041619	0.526320668	0.9995992	3.710784e-02	0.418566338	0.9546035	0.0846042000	3.218308e-01
PPP2R1A	0.0944405802	1.378373e-01	5.989843e-01	0.0412919089	0.516461861	0.9995992	1.306239e-01	0.021055173	0.9546035	0.1085814465	4.341396e-02
PPP2R1B	0.0076355228	9.022026e-01	9.710488e-01	-0.0499712096	0.425352846	0.9995992	-3.210105e-02	0.693667674	0.9546035	0.0121524865	4.259205e-01
FCER1G	-0.5831138037	1.418877e-01	6.076494e-01	-0.0835278713	0.836653647	0.9995992	-2.221429e-01	0.709476110	0.9546035	0.1555963165	3.528198e-01
ADSL	-0.0399491706	6.884743e-01	8.657198e-01	-0.0871546170	0.379509225	0.9995992	6.434181e-02	0.265783630	0.9546035	-0.0304320358	6.148386e-01
PKLR	0.0017982677	9.830490e-01	9.936282e-01	0.0006519488	0.993945889	0.9995992	-1.529611e-02	0.570673998	0.9546035	0.0340073470	3.414668e-01
SRI	-0.1717832415	5.479758e-02	4.562264e-01	-0.0135235537	0.885597650	0.9995992	-7.600668e-02	0.800562767	0.9572213	-0.0627982285	7.397914e-01
GSTT1	0.2842542002	2.332726e-01	6.697768e-01	0.2349671195	0.350874540	0.9995992	-8.580481e-02	0.636951584	0.9546035	0.2931399571	1.112308e-01
SERPINB1	0.0500884701	4.756969e-01	8.125882e-01	0.0240043032	0.728696089	0.9995992	-1.819075e-01	0.995533273	0.9955333	-0.0990162392	9.380423e-01
CORO1A	-0.0164141100	8.957465e-01	9.687085e-01	-0.0695112552	0.578501480	0.9995992	-6.942856e-02	0.708870678	0.9546035	-0.1287113685	8.493358e-01
GDI1	-0.0528461684	6.062072e-01	8.419469e-01	0.1687493075	0.093707913	0.9995992	-1.571794e-02	0.564382302	0.9546035	0.1008018121	1.623208e-01
MAT2A	0.0740227342	3.932379e-01	7.802736e-01	0.0973306014	0.248986712	0.9995992	4.864341e-02	0.290119571	0.9546035	0.1170499408	9.201536e-02
ATIC	-0.0035478179	9.711728e-01	9.926294e-01	-0.0628748477	0.514157665	0.9995992	-2.277391e-02	0.590028207	0.9546035	0.0507618487	3.051168e-01
YWHAB	-0.1201759652	3.713654e-01	7.647280e-01	0.1229731902	0.372784463	0.9995992	-1.358657e-01	0.842423100	0.9572213	0.1018540569	2.399298e-01
STIP1	-0.0503247809	5.406764e-01	8.281031e-01	-0.0656981006	0.430829539	0.9995992	-1.910023e-03	0.509129204	0.9546035	-0.0358945676	6.690865e-01
S100A11	0.2985554209	6.893445e-02	4.656029e-01	0.0744029312	0.656726601	0.9995992	-5.451417e-02	0.625837721	0.9546035	0.0564789977	3.693205e-01
PRDX2	0.1784517589	3.463643e-02	4.027342e-01	0.0516622916	0.553617005	0.9995992	4.797175e-03	0.478302550	0.9546035	0.0887700360	1.643492e-01
CDH5	-0.0323559780	8.549502e-01	9.533502e-01	-0.0768695539	0.659084880	0.9995992	-2.605544e-01	0.928247669	0.9658352	-0.1750904406	8.205926e-01
HSPA4	0.1026432520	5.878792e-02	4.562264e-01	0.0029547032	0.958121072	0.9995992	4.791548e-03	0.466342288	0.9546035	0.0945294017	4.692363e-02
RDX	-0.3003704284	4.524120e-02	4.531788e-01	-0.1034500943	0.492393243	0.9995992	8.878546e-02	0.283267926	0.9546035	0.0017769046	4.954596e-01
KRT9	-0.0811312677	9.047794e-01	9.710488e-01	-0.4938951723	0.460296979	0.9995992	1.428810e-01	0.420777168	0.9546035	-0.3066611089	6.788023e-01
SAA4	-0.0147355339	9.233114e-01	9.752941e-01	-0.2273014926	0.140833067	0.9995992	1.158439e-01	0.228039725	0.9546035	-0.2398547457	9.340307e-01
MYH9	-0.0361935621	6.689259e-01	8.576613e-01	0.0496356042	0.554077137	0.9995992	4.296955e-02	0.308407359	0.9546035	0.0527925971	2.616539e-01
MYH10	1.0488239244	3.995107e-01	7.830730e-01	-0.9758331951	0.453817457	0.9995992	-8.322642e-01	0.751490031	0.9546035	-1.0511093238	7.753564e-01
ADD1	-0.0449832164	4.375362e-01	8.021362e-01	0.0526085210	0.365842596	0.9995992	2.985132e-02	0.307428667	0.9546035	0.0542777841	1.831055e-01
ADD2	0.0229791880	6.366905e-01	8.484797e-01	0.0274230902	0.564365413	0.9995992	2.186520e-03	0.482361502	0.9546035	0.0989584749	2.780779e-02
BSG	-0.3700255657	4.542470e-01	8.045226e-01	-0.0661124681	0.893742547	0.9995992	4.745494e-02	0.462141091	0.9546035	0.5440448997	1.310050e-01
GLRX	0.2501056262	1.246009e-01	5.753055e-01	-0.0120613630	0.941322432	0.9995992	-3.621087e-03	0.508641292	0.9546035	0.1252580486	2.280431e-01
PPM1A	-0.3961793745	6.508049e-03	2.564171e-01	-0.1622018704	0.285238758	0.9995992	-1.178657e-01	0.784174415	0.9572213	-0.0415803526	6.033644e-01
IGFALS	-0.5516815174	1.608494e-03	1.319384e-01	0.0690677347	0.697028152	0.9995992	9.704649e-02	0.297995969	0.9546035	-0.1433848061	8.014232e-01
KRT2	2.0962719853	1.476090e-01	6.165020e-01	1.0889634935	0.380181165	0.9995992	-1.305498e+00	0.833027771	0.9572213	1.0489678309	1.887847e-01
PSMC2	0.1529742441	3.008087e-02	3.864738e-01	-0.0745865276	0.303996197	0.9995992	-3.206449e-03	0.517329400	0.9546035	0.0303107037	3.369212e-01
MAP2K2	-0.1395855287	6.152398e-01	8.419469e-01	-0.3161365794	0.291630083	0.9995992	-3.153297e-01	0.869067279	0.9572213	-0.4374996623	9.107509e-01
ATP6V1E1	0.0065824497	9.733143e-01	9.926294e-01	0.0461941231	0.812044261	0.9995992	1.831311e-01	0.180990549	0.9546035	0.1954804381	1.538408e-01
PPP1CC;PPP1CA;PPP	-0.2108347892	1.745911e-01	6.291668e-01	-0.1571792924	0.316945553	0.9995992	-3.898104e-02	0.595676813	0.9546035	-0.0677439265	6.638622e-01
GNL1	-0.0334182350	6.016646e-01	8.419469e-01	0.0286392711	0.651340584	0.9995992	-9.945129e-02	0.936582125	0.9676924	-0.0017759019	5.110266e-01
SERPINF1	0.0917163828	5.548834e-01	8.319734e-01	0.0086890952	0.955455554	0.9995992	-1.462149e-01	0.826296302	0.9572213	-0.2685010370	9.380214e-01
GMPR	-0.0597250970	5.780386e-01	8.363994e-01	-0.0135740054	0.898900946	0.9995992	-2.885438e-02	0.603720639	0.9546035	0.0384053645	3.616586e-01
GPX4	-0.1787526647	2.186794e-01	6.697768e-01	0.2097468781	0.147297664	0.9995992	1.075660e-01	0.236351829	0.9546035	0.2826990507	2.648985e-02
CFHR2	-0.1556344527	3.097019e-01	7.192836e-01	-0.0473471880	0.758061908	0.9995992	-1.303780e-01	0.795981691	0.9572213	-0.1547795336	8.413615e-01
TAGLN2	-0.1601845183	8.754248e-03	2.723032e-01	-0.0021247118	0.974063790	0.9995992	-4.019457e-02	0.734010502	0.9546035	-0.0587331262	8.167099e-01
TALDO1	-0.0990930480	1.718610e-01	6.287166e-01	-0.0597134906	0.421705227	0.9995992	7.194304e-02	0.162923288	0.9546035	-0.0239324257	6.282035e-01
SNCA	0.0003091218	9.970281e-01	9.990571e-01	-0.0252723179	0.755288139	0.9995992	1.306009e-02	0.438415105	0.9546035	-0.0510775054	7.221143e-01
ATP6V1A	0.0313183598	6.237493e-01	8.419469e-01	-0.0517019003	0.441763686	0.9995992	6.492865e-02	0.159137589	0.9546035	-0.0136077206	5.797208e-01
COL18A1	0.1681877536	6.136080e-01	8.419469e-01	-0.0100662401	0.974691601	0.9995992	-4.763885e-01	0.923009844	0.9644203	-0.5593718726	9.328997e-01
ANP32A	-0.3311458986	2.618026e-01	6.900429e-01	-0.5841647269	0.048204089	0.9995992	-1.676140e-01	0.711762997	0.9546035	-0.6074575461	9.818624e-01
GP5	-0.5335029446	7.878140e-02	4.953171e-01	-0.0476333096	0.876187618	0.9995992	-5.934159e-02	0.576850401	0.9546035	0.0768399698	4.036995e-01
CCT6A	-0.0696242827	3.371445e-01	7.385169e-01	0.0084958520	0.911975095	0.9995992	-2.028580e-02	0.611089366	0.9546035	0.0168960655	4.145109e-01
MDH1	0.0488964237	4.785969e-01	8.125882e-01	0.0065904050	0.924954039	0.9995992	2.015814e-03	0.488578238	0.9546035	0.0674532907	1.769899e-01
MDH2	0.0297563044	8.882106e-01	9.687085e-01	-0.0493789975	0.813158688	0.9995992	-2.080177e-01	0.835960699	0.9572213	-0.0248538424	5.470160e-01
EIF2S3	0.0594716266	3.673298e-01	7.603920e-01	0.0213934208	0.745642354	0.9995992	-1.306269e-01	0.975527790	0.9821753	-0.0847190051	8.871064e-01
MNDA	-0.6208445212	5.368805e-01	8.281031e-01	-1.1333815326	0.300977481	0.9995992	-8.339804e-01	0.794229793	0.9572213	-0.5028528478	6.727268e-01
PTGDS	0.1459263467	7.205458e-01	8.833575e-01	0.5056750298	0.215921657	0.9995992	-1.354359e-01	0.631126271	0.9546035	0.0826368098	4.191999e-01
AKR1C3	0.0214157240	9.180730e-01	9.741133e-01	-0.2409008170	0.268045403	0.9995992	-1.724440e-01	0.798000122	0.9572213	-0.2357224683	8.449119e-01
PAFAH1B1	0.0360056415	6.584104e-01	8.524806e-01	-0.0690352545	0.415396135	0.9995992	6.158585e-02	0.228513174	0.9546035	0.0467319831	2.925467e-01
BTD	-0.1458789804	8.213446e-02	5.027637e-01	0.0467734724	0.595340448	0.9995992	-4.546095e-02	0.703737588	0.9546035	-0.0101758389	5.438435e-01
RANBP1	0.0947321742	1.620656e-01	6.287166e-01	-0.0543050506	0.440844308	0.9995992	1.780762e-03	0.489680868	0.9546035	-0.0040414399	5.223802e-01
AFM	-0.1016117711	3.380183e-01	7.385169e-01	0.0368927674	0.721823939	0.9995992	1.230994e-01	0.129673470	0.9546035	0.0138424806	4.468072e-01
PSMC4	0.1049562542	2.845939e-01	6.987920e-01	-0.1588269765	0.105016769	0.9995992	-5.666911e-03	0.522530913	0.9546035	-0.1054666415	8.509531e-01
USP5	0.1779122594	3.195478e-02	3.975847e-01	0.0422116858	0.623170841	0.9995992	2.247708e-02	0.397412512	0.9546035	0.1366599082	6.066012e-02
RANGAP1	-0.2361804293	2.214680e-01	6.697768e-01	0.3128141677	0.095395160	0.9995992	-1.827602e-02	0.536547394	0.9546035	-0.0548236390	6.114923e-01
CRK	0.1142876686	2.628977e-01	6.900429e-01	-0.1382644897	0.173033542	0.9995992	1.426577e-01	0.072369499	0.9546035	-0.0533267575	6.911124e-01
CRKL	0.1871234638	1.018662e-01	5.314990e-01	0.0854584607	0.461293195	0.9995992	3.127120e-01	0.002246610	0.6382854	0.1609474594	8.141289e-02
NSF	0.0722846741	3.422005e-01	7.434558e-01	0.0886883755	0.258844229	0.9995992	3.586855e-02	0.322625523	0.9546035	0.1091631911	6.747796e-02
MAP2K3	-0.2479138221	2.711594e-01	6.940168e-01	-0.2738046390	0.250852980	0.9995992	-1.594748e-01	0.758146602	0.9559168	-0.3420837330	9.058747e-01
RPS10	0.2717844037	5.477901e-01	8.281031e-01	-0.2204588761	0.629274525	0.9995992	-4.343332e-02	0.538068400	0.9546035	0.2650330115	2.785250e-01
GNPDA1	0.0028256264	9.743134e-01	9.927918e-01	-0.0173453847	0.846028071	0.9995992	-1.738571e-01	0.976531800	0.9823494	0.0039841189	4.826150e-01
CAPZA2	0.6676999026	2.241828e-01	6.697768e-01	-0.3127028586	0.582715331	0.9995992	3.417913e-01	0.269290180	0.9546035	-0.3458763872	7.148413e-01
CAPZB	0.0856124832	2.505469e-01	6.761334e-01	0.0104633791	0.890938935	0.9995992	1.396042e-03	0.492330483	0.9546035	0.0257657482	3.690504e-01
EIF1AX	0.1082765132	4.197358e-01	7.950765e-01	0.0905235961	0.517038810	0.9995992	-7.493831e-02	0.710116647	0.9546035	-0.2076231218	9.351787e-01
LIMS1	2.9476464476	4.114854e-02	4.250863e-01	1.2753651176	0.253491187	0.9995992	-6.411473e-01	0.719210074	0.9546035	1.1173447259	1.477349e-01
PIP4K2A	-0.0130614379	8.102858e-01	9.384947e-01	0.0101640534	0.851883489	0.9995992	6.373994e-02	0.125170869	0.9546035	0.0560810149	1.479732e-01
GCLC	0.0821455357	1.713606e-01	6.287166e-01	0.0092121253	0.877910561	0.9995992	2.791418e-02	0.324507435	0.9546035	0.0448667551	2.243619e-01
GCLM	-0.2911944262	1.208309e-02	3.084914e-01	0.0736582017	0.529653003	0.9995992	1.573038e-01	0.096481523	0.9546035	0.1287089942	1.342406e-01
PSMD8	-0.0038125299	9.835406e-01	9.936282e-01	-0.1374990840	0.473139261	0.9995992	3.617197e-01	0.026267417	0.9546035	0.2638287796	6.719826e-02
GSS	0.0619686589	2.393431e-01	6.725635e-01	-0.0181460801	0.734136988	0.9995992	-5.157029e-02	0.830361576	0.9572213	0.0300473115	2.952450e-01
CCT5	0.0089873784	8.908552e-01	9.687085e-01	0.0356830862	0.592534161	0.9995992	-5.873505e-02	0.811382181	0.9572213	0.0178730913	3.994797e-01
CSNK1A1	0.1066060759	3.237830e-01	7.234622e-01	0.1667415622	0.124184141	0.9995992	1.170268e-01	0.135972055	0.9546035	0.2462634338	1.985543e-02
IDH2	-0.3805644614	5.144609e-02	4.562264e-01	-0.1375883378	0.490907509	0.9995992	6.370676e-03	0.487400420	0.9546035	-0.0539284769	6.055780e-01
PITPNB	0.0456492363	4.831763e-01	8.129736e-01	0.0729679119	0.258139520	0.9995992	7.491879e-02	0.117083440	0.9546035	0.0623634972	1.796952e-01
MASP1	-0.3400904240	1.501102e-02	3.187282e-01	-0.1482753565	0.304062260	0.9995992	8.853537e-02	0.267164823	0.9546035	-0.2440108108	9.509483e-01
ALDH9A1	0.2446104526	4.719677e-02	4.562264e-01	-0.1412580688	0.253096362	0.9995992	-1.582461e-01	0.892798858	0.9579808	-0.0819841114	7.496570e-01
RPIA	0.0795354894	2.900984e-01	6.997884e-01	0.0836522719	0.271882779	0.9995992	-3.848015e-02	0.691657180	0.9546035	0.0302467121	3.512324e-01
FASN	-0.4159926100	6.077608e-01	8.419469e-01	0.5964384053	0.439096883	0.9995992	8.332563e-01	0.175321864	0.9546035	0.4907365219	2.503227e-01
CCT3	0.0143928528	8.468025e-01	9.508196e-01	0.0228190937	0.766354336	0.9995992	9.275412e-02	0.110086928	0.9546035	0.0709292325	1.797512e-01
CARS1	0.1354657335	3.636193e-01	7.553917e-01	-0.2065026902	0.185636814	0.9995992	6.082577e-03	0.484275391	0.9546035	-0.1977338596	8.854697e-01
PSMB3	0.1433714473	1.225232e-01	5.724507e-01	-0.0487711619	0.598930040	0.9995992	-1.504294e-01	0.943269605	0.9707604	0.0233232947	4.011402e-01
PSMB2	0.0689628886	4.760564e-01	8.125882e-01	-0.0051400868	0.959171296	0.9995992	-1.411518e-02	0.556783669	0.9546035	0.1285253385	1.132712e-01
COMP	-0.0089879974	9.713419e-01	9.926294e-01	0.2947368231	0.227191645	0.9995992	-4.750121e-02	0.575706405	0.9546035	-0.0381122630	5.597525e-01
HINT1	0.2176427841	1.373144e-02	3.121263e-01	0.0221115157	0.809298084	0.9995992	1.359551e-01	0.070171426	0.9546035	0.0302373287	3.758457e-01
SELENOP	-0.1032546521	2.169911e-01	6.697768e-01	-0.0888322607	0.291618377	0.9995992	-5.945880e-02	0.753890344	0.9546035	-0.0908303526	8.518615e-01
CAMP	-0.1150153133	5.035240e-01	8.151018e-01	-0.1256561429	0.469694940	0.9995992	-1.212905e-01	0.755353496	0.9546035	0.0306347968	4.336413e-01
GMPS	0.8657900418	3.709680e-01	7.647280e-01	-0.1599022621	0.867509096	0.9995992	1.305359e-01	0.445245448	0.9546035	-1.0674684887	8.589352e-01
GDI2	0.0421253553	4.816373e-01	8.129736e-01	0.0313354798	0.605696161	0.9995992	-4.090833e-02	0.749246254	0.9546035	0.0066101963	4.573011e-01
ST13	0.0243525640	7.487404e-01	9.039005e-01	0.0445567960	0.571728960	0.9995992	3.697720e-02	0.317158498	0.9546035	0.0984548007	1.202726e-01
VASP	-0.0480166324	9.338180e-01	9.813741e-01	-0.0514450947	0.930277398	0.9995992	2.334887e-02	0.484155138	0.9546035	-0.2407222979	6.547591e-01
DNM2	-0.0311409197	6.584727e-01	8.524806e-01	0.0645237858	0.364348016	0.9995992	2.440132e-02	0.367766730	0.9546035	0.0929100294	1.151462e-01
NUDT2	0.1569359582	3.468997e-01	7.479692e-01	0.2032690001	0.228547379	0.9995992	-4.437114e-02	0.602814217	0.9546035	0.2947642408	4.140409e-02
BCAM	-0.2069204202	6.856277e-01	8.640904e-01	0.0564305112	0.908876933	0.9995992	-2.385582e-01	0.679353948	0.9546035	0.5962565099	1.042029e-01

**CCT8**	0.0710933646	2.942491e-01	7.010683e-01	0.0444310181	0.528058543	0.9995992	1.570119e-02	0.410216356	0.9546035	0.0696562087	1.651135e-01
**CCT4**	-0.0309137345	6.202426e-01	8.419469e-01	-0.0323549373	0.602345491	0.9995992	-3.784947e-04	0.502353459	0.9546035	-0.0152670161	5.973171e-01
**RAB5C**	-0.1339541696	4.772699e-01	8.125882e-01	-0.0041039636	0.982065851	0.9995992	1.209517e-01	0.260008465	0.9546035	0.1580778798	1.876695e-01
**RAB7A**	-0.3579528333	1.548095e-02	3.187282e-01	0.2056529281	0.176381463	0.9995992	5.977040e-02	0.347033933	0.9546035	0.1680470928	1.348837e-01
**PSMD7**	0.2086796700	3.150096e-02	3.961078e-01	-0.1362341407	0.174332198	0.9995992	3.907627e-02	0.350114829	0.9546035	0.0634774979	2.662089e-01
**RPS6KA3**	0.0712624256	5.731676e-01	8.344560e-01	-0.0593707584	0.634135109	0.9995992	-7.396465e-03	0.522804834	0.9546035	-0.0164612837	5.522266e-01
**LUM**	0.0658041626	6.208122e-01	8.419469e-01	0.0442363267	0.734576013	0.9995992	-1.415153e-01	0.852489809	0.9572213	-0.1385160263	8.459457e-01
**PGD**	0.1965083509	1.901393e-03	1.319384e-01	-0.0065691663	0.921358686	0.9995992	7.132849e-02	0.146329416	0.9546035	0.0697517485	1.436648e-01
**ARHGDIA**	0.0171405689	7.866081e-01	9.267668e-01	0.0702174546	0.266002791	0.9995992	9.393259e-02	0.069822675	0.9546035	0.0690718102	1.389766e-01
**ARHGDIB**	0.1938319446	7.761455e-02	4.905904e-01	0.0574863983	0.616945819	0.9995992	-2.463024e-03	0.508794548	0.9546035	-0.0712576910	7.294789e-01
**HNRNPF**	-0.1356884246	1.564366e-01	6.224838e-01	-0.0074049073	0.937374807	0.9995992	1.028374e-01	0.150935735	0.9546035	0.0224866083	4.057415e-01
**RIDA**	0.1602119544	1.524131e-01	6.224838e-01	0.0184106165	0.871584451	0.9995992	4.338734e-02	0.354557326	0.9546035	0.1576818168	7.702140e-02
**THOP1**	-0.4591724894	6.848204e-02	4.652056e-01	0.2695266120	0.302974274	0.9995992	3.921832e-03	0.493994136	0.9546035	-0.1149554716	6.687859e-01
**CAPZA1**	0.1088527049	2.000761e-01	6.617157e-01	0.0887961433	0.307730860	0.9995992	4.803740e-02	0.289468404	0.9546035	0.1418984056	5.955169e-02
**BLVRA**	0.0514511607	5.997379e-01	8.419469e-01	0.0405725587	0.681816414	0.9995992	5.947553e-02	0.276155461	0.9546035	0.0520607940	3.018337e-01
**PPP5C**	-0.1632434386	3.496856e-01	7.487834e-01	-0.2802868291	0.106130932	0.9995992	6.386353e-03	0.485697227	0.9546035	-0.1806473055	8.446407e-01
**ACLY**	0.0447821293	4.885755e-01	8.151018e-01	0.0341715689	0.610150941	0.9995992	5.773394e-02	0.188747281	0.9546035	0.1084709868	6.680979e-02
**MVD**	0.1896902934	3.385123e-02	4.027342e-01	0.0107781906	0.905539968	0.9995992	-1.266579e-01	0.919228280	0.9644203	-0.0400585282	6.681373e-01
**RABGGTB**	0.1302820791	4.532731e-01	8.045226e-01	0.0588058671	0.734146910	0.9995992	1.450904e-01	0.203378018	0.9546035	0.0440332895	4.005733e-01
**AP2S1**	0.0443243734	6.583006e-01	8.524806e-01	-0.0596291443	0.558874157	0.9995992	4.380109e-02	0.331908643	0.9546035	0.0066086747	4.736218e-01
**SLC16A1**	-0.1650151554	4.917027e-01	8.151018e-01	0.0045231540	0.985059072	0.9995992	1.262174e-01	0.302213406	0.9546035	0.3475540153	6.635043e-02
**CLNS1A**	-0.0493886309	6.381543e-01	8.484797e-01	-0.0016415348	0.987518865	0.9995992	-1.682549e-02	0.563551581	0.9546035	0.1313641917	1.192655e-01
**CRISP3**	0.0447138891	8.252905e-01	9.430642e-01	-0.1910601512	0.357376075	0.9995992	-7.634030e-02	0.643732913	0.9546035	-0.1775909308	7.880780e-01
**YARS1**	0.3187653305	1.634956e-02	3.187282e-01	-0.2254151055	0.099866419	0.9995992	7.147251e-02	0.303700540	0.9546035	-0.1143083958	7.893050e-01
**USP14**	0.0890247713	1.519883e-01	6.224838e-01	0.0381379229	0.549298172	0.9995992	5.525590e-02	0.193428332	0.9546035	0.0996214488	6.529101e-02
**RAD23A**	-0.0001604779	9.985180e-01	9.990571e-01	-0.0083543975	0.924662426	0.9995992	-4.856840e-02	0.709242121	0.9546035	-0.0075246357	5.337106e-01
**RAD23B**	0.0450484421	4.160745e-01	7.920800e-01	-0.0330634708	0.549381426	0.9995992	1.218981e-02	0.415439226	0.9546035	-0.0161199151	6.106059e-01
**NAPA**	0.0454025043	3.960043e-01	7.802736e-01	0.1292878632	0.014101449	0.9995992	-1.388526e-02	0.601374159	0.9546035	0.1191244832	1.745189e-02
**EIF5**	0.0572513529	5.178974e-01	8.216271e-01	0.0149029107	0.871517425	0.9995992	-1.325331e-02	0.557980761	0.9546035	-0.0022359966	5.092835e-01
**PSMD4**	0.0544375736	4.661306e-01	8.079413e-01	-0.1553774136	0.038294523	0.9995992	4.597226e-02	0.273575234	0.9546035	-0.0274015907	6.358992e-01
**APOC4**	0.1251547240	7.872030e-01	9.267668e-01	0.4631940330	0.292397640	0.9995992	3.640019e-01	0.222394769	0.9546035	0.2471556184	2.745259e-01
**PLTP**	0.0362456897	8.036683e-01	9.349763e-01	-0.0453068228	0.754558959	0.9995992	2.125156e-01	0.075749449	0.9546035	0.0002686445	4.992490e-01
**CSE1L**	-0.1841623438	1.571487e-01	6.224838e-01	0.0762115879	0.559820577	0.9995992	5.881948e-02	0.325722390	0.9546035	0.1796884410	7.370626e-02
**VCP**	0.2230394287	8.802727e-04	1.319384e-01	0.0121146523	0.865595052	0.9995992	6.645922e-02	0.178814234	0.9546035	0.0395021203	2.887805e-01
**ADK**	0.3563191644	1.294139e-03	1.319384e-01	0.0222325913	0.844245315	0.9995992	-2.967953e-02	0.597600802	0.9546035	0.0230516062	4.145595e-01
**CDH13**	0.5309787335	2.069747e-01	6.658594e-01	0.7444768080	0.068650338	0.9995992	2.728535e-01	0.259507667	0.9546035	-0.1673195150	6.424347e-01
**NPEPPS**	-0.2828440590	5.347977e-02	4.562264e-01	-0.0127535346	0.931308313	0.9995992	-2.119007e-02	0.555966947	0.9546035	0.1260521608	1.956192e-01
**SUMO3;SUMO2;SUMO**	0.1234075464	1.038962e-01	5.362675e-01	0.0087157606	0.910064375	0.9995992	7.556658e-02	0.167840305	0.9546035	0.0557704843	2.277053e-01
**BID**	0.1413347499	2.965669e-02	3.864738e-01	-0.0796620784	0.213904605	0.9995992	-6.758072e-02	0.842748535	0.9572213	-0.1003186397	9.397329e-01
**EIF6**	-0.1411967828	1.303961e-01	5.816156e-01	-0.1036255446	0.289652960	0.9995992	-3.579658e-02	0.649487788	0.9546035	-0.0491534183	6.795960e-01
**MTPN**	0.1614042283	9.147198e-02	5.171548e-01	0.2152473526	0.025994516	0.9995992	1.018931e-01	0.146644467	0.9546035	0.1290958263	1.067025e-01
**DEFA3**	0.2907584436	1.606024e-01	6.285829e-01	-0.0936289148	0.648964115	0.9995992	-3.751399e-02	0.570508784	0.9546035	0.0649308049	3.776910e-01
**ARPC4**	-0.0445745682	6.037663e-01	8.419469e-01	-0.0211722589	0.803919614	0.9995992	-8.414554e-02	0.834225157	0.9572213	0.0159564889	4.241129e-01
**TPI1**	0.0953851716	1.237726e-01	5.737224e-01	-0.0143910558	0.818548952	0.9995992	-7.751147e-02	0.888363068	0.9579808	-0.0017380940	5.107290e-01
**MYL6**	-0.3037107638	9.308381e-03	2.750627e-01	-0.0281355061	0.819436860	0.9995992	-3.630714e-02	0.618972892	0.9546035	-0.0894481879	7.623619e-01
**ACTB**	0.0195724915	7.323454e-01	8.933254e-01	0.0722950637	0.209042975	0.9995992	-8.097066e-02	0.921255613	0.9644203	-0.0133478192	5.920698e-01
**EIF4A1**	0.0297593360	7.828068e-01	9.256765e-01	-0.1032179148	0.349237676	0.9995992	2.930224e-01	0.002700023	0.6382854	0.0095211125	4.663882e-01
**PRPS1**	0.0658702245	4.462982e-01	8.021362e-01	0.1167619339	0.179299683	0.9995992	1.160925e-01	0.093781595	0.9546035	0.1150033642	9.954216e-02
**PSMA6**	0.1351249244	1.663520e-02	3.187282e-01	-0.0197475164	0.737223812	0.9995992	-5.266559e-05	0.500351943	0.9546035	0.0385599187	2.586570e-01
**CDC42**	-0.0075696254	9.559545e-01	9.907192e-01	-0.0133650474	0.920570836	0.9995992	-7.762173e-02	0.711535374	0.9546035	0.1427799243	1.362227e-01
**GMFB**	-0.0923975031	5.176516e-01	8.216271e-01	0.0277564304	0.847980234	0.9995992	1.058794e-01	0.232570128	0.9546035	0.1519938475	1.571073e-01
**RAB2A**	0.3801970928	8.146084e-02	5.027637e-01	0.3402557658	0.123189349	0.9995992	-1.479621e-01	0.744654812	0.9546035	0.1798194821	2.096326e-01
**RAB5B**	-0.0631550581	4.050099e-01	7.850184e-01	0.1427644826	0.057884889	0.9995992	4.480652e-02	0.276544756	0.9546035	0.1554508310	2.067292e-02
**RAB10**	-0.3710807930	8.329412e-02	5.027637e-01	0.0003346808	0.998786086	0.9995992	-1.758089e-02	0.531834606	0.9546035	0.2245664009	1.608490e-01
**UBE2D3;UBE2D2**	0.0504260479	5.888150e-01	8.419469e-01	0.1594076984	0.095938565	0.9995992	1.561875e-03	0.493495605	0.9546035	0.1655760600	3.626189e-02
**UBE2M**	0.1427340503	3.954155e-01	7.802736e-01	-0.1399866864	0.435354672	0.9995992	3.029198e-01	0.036057862	0.9546035	-0.0088590200	5.187804e-01
**UBE2K**	0.0393866091	7.022193e-01	8.709582e-01	0.0506946233	0.632477626	0.9995992	1.035492e-03	0.496019763	0.9546035	0.0392965042	3.541707e-01
**UBE2N**	0.0716309109	1.777061e-01	6.345879e-01	0.0086250384	0.869833342	0.9995992	-6.594858e-02	0.887103699	0.9579808	0.0196750349	3.567797e-01
**RAB14**	0.2603943615	2.327287e-01	6.697768e-01	-0.0192775997	0.930582334	0.9995992	2.650316e-01	0.119132735	0.9546035	0.2599222092	1.125971e-01
**ACTR3**	0.0374104040	6.526006e-01	8.523468e-01	-0.0213380540	0.797145374	0.9995992	2.120248e-02	0.400502723	0.9546035	0.0088047594	4.568853e-01
**ACTR2**	0.1142781400	1.886670e-01	6.427328e-01	-0.0406591826	0.638954632	0.9995992	-7.930027e-02	0.813319015	0.9572213	-0.0284445095	6.224321e-01
**ACTR1A**	0.0135344905	8.787043e-01	9.651504e-01	-0.0104037748	0.908480634	0.9995992	6.088338e-02	0.245471522	0.9546035	0.0036640584	4.844816e-01
**COPS2**	0.1536466481	1.906722e-02	3.414766e-01	0.0057718650	0.932072601	0.9995992	-7.710161e-02	0.870028283	0.9572213	0.0342675484	3.149693e-01
**ARF3;ARF1**	-0.0491993909	3.642752e-01	7.553917e-01	-0.0540369511	0.317257104	0.9995992	-6.193665e-02	0.869361071	0.9572213	-0.0044043587	5.305114e-01
**RAP1B**	-0.3301632219	2.250912e-02	3.455296e-01	0.0594673702	0.688139492	0.9995992	-2.698232e-02	0.572287406	0.9546035	0.1217468893	2.011200e-01
**RAP2B**	-0.1178534882	7.015277e-01	8.709582e-01	0.0070936733	0.981360217	0.9995992	-9.457419e-02	0.620587819	0.9546035	0.3490784653	1.253497e-01
**RHOA**	-0.1693487304	2.778305e-01	6.987142e-01	-0.0705692344	0.655786233	0.9995992	1.365358e-03	0.496551146	0.9546035	0.1509866731	1.733774e-01
**HSPE1**	0.6922376243	3.635563e-01	7.553917e-01	0.6573779214	0.356458025	0.9995992	-7.196725e-01	0.827575405	0.9572213	0.6768578638	1.576052e-01
**LYZ**	0.3231251442	5.860352e-02	4.562264e-01	0.1122800302	0.507383968	0.9995992	-1.606417e-01	0.818233721	0.9572213	0.0559647796	3.792679e-01
**B2M**	0.5701833141	3.213472e-01	7.225946e-01	0.4621645161	0.387899562	0.9995992	-6.595928e-01	0.873451048	0.9572213	-0.6884268094	8.479242e-01
**NUTF2**	-0.0990923032	4.806849e-01	8.129736e-01	-0.1351371829	0.361199259	0.9995992	1.620644e-03	0.495456089	0.9546035	-0.1502524263	8.254964e-01
**HNRNPK**	0.1042055903	1.001846e-01	5.314990e-01	-0.1462077354	0.024538779	0.9995992	6.516892e-02	0.150028966	0.9546035	-0.0852094253	8.946294e-01
**YWHAG**	0.0123595380	9.146128e-01	9.738869e-01	0.0635199182	0.594546863	0.9995992	8.069070e-02	0.246440156	0.9546035	0.0770383667	2.626947e-01
**PSMC1**	0.0600346266	5.227061e-01	8.226879e-01	-0.0473608441	0.617947455	0.9995992	-3.664006e-02	0.649291562	0.9546035	0.1018883534	1.444652e-01
**PSMC5**	0.1004004811	1.315860e-01	5.830557e-01	-0.1324718953	0.053735438	0.9995992	2.011579e-02	0.383978468	0.9546035	-0.0604703067	8.095023e-01
**YWHAE**	0.1161806576	2.441030e-01	6.725635e-01	-0.1075069227	0.301593223	0.9995992	4.470922e-04	0.498276660	0.9546035	-0.0775056664	7.699777e-01
**3,00 LSM**	0.0960757648	2.999845e-01	7.059294e-01	0.0829220941	0.376684829	0.9995992	6.460134e-02	0.247898844	0.9546035	0.0421506169	3.284723e-01
**TMSB4X**	-0.1403498188	1.447712e-01	6.111413e-01	-0.0673264419	0.501458700	0.9995992	-6.289931e-02	0.738828920	0.9546035	-0.1352113428	9.168328e-01
**PSMC6**	0.1604321544	1.276371e-02	3.121263e-01	-0.0173478813	0.801360684	0.9995992	4.809784e-02	0.236930778	0.9546035	0.0610675840	1.917102e-01
**RAB11A;RAB11B**	-0.2114982979	2.392422e-02	3.507394e-01	-0.0275926098	0.768225062	0.9995992	-7.498273e-02	0.777047274	0.9572213	0.0414100397	3.360615e-01
**ETF1**	0.2153116468	5.746651e-02	4.562264e-01	0.1121016933	0.314011213	0.9995992	2.302398e-01	0.024931167	0.9546035	0.0561687907	3.171138e-01
**ACTA2;ACTG2**	-0.0134755496	8.344838e-01	9.493357e-01	0.0883036895	0.186258643	0.9995992	-6.808754e-02	0.852019470	0.9572213	-0.0003396886	5.019818e-01
**H4C1**	-0.9199948398	1.696893e-01	6.287166e-01	0.0268355101	0.967252894	0.9995992	-3.456154e-02	0.521042021	0.9546035	0.5498697546	2.099744e-01
**RAB1A**	-0.4910850883	7.402088e-03	2.615380e-01	-0.2789060968	0.130110852	0.9995992	2.735801e-02	0.443594550	0.9546035	-0.2653490809	9.273852e-01
**RAN**	0.0289930315	7.211844e-01	8.833575e-01	0.0110765805	0.894490336	0.9995992	4.688380e-03	0.477254696	0.9546035	0.0776196349	1.773942e-01
**RAP1A**	-0.1802686843	1.703129e-01	6.287166e-01	-0.1119276678	0.407294830	0.9995992	-6.616085e-02	0.690834033	0.9546035	0.0328579320	4.035440e-01
**RPS26;RPS26P11**	0.2404713778	3.603767e-02	4.077295e-01	0.0327147691	0.782584365	0.9995992	1.318606e-01	0.131532754	0.9546035	0.1274786695	1.474638e-01
**GNB1**	-0.0537341011	3.367056e-01	7.385169e-01	-0.0063361551	0.913135290	0.9995992	-6.791903e-02	0.885808526	0.9579808	-0.0408150453	7.566824e-01
**RBX1**	0.0771622291	2.149564e-01	6.680462e-01	-0.0394238379	0.522872633	0.9995992	2.197117e-02	0.363563654	0.9546035	0.0247673966	3.513837e-01
**RPL11**	-0.1305011456	5.626020e-01	8.323189e-01	-0.1609448749	0.488353809	0.9995992	2.513178e-01	0.136572181	0.9546035	-0.0935877242	6.493406e-01
**PPIA**	0.0920779705	1.134832e-01	5.517720e-01	0.0476149089	0.417671958	0.9995992	6.405974e-02	0.143379312	0.9546035	0.0959143693	5.117874e-02
**FKBP1A**	0.0192782918	7.381569e-01	8.976831e-01	0.0007347728	0.989590950	0.9995992	8.608455e-02	0.070918772	0.9546035	0.0414028946	2.278104e-01
**RAC1**	-0.2086781515	4.150978e-01	7.920800e-01	-0.0759214525	0.770389852	0.9995992	6.369432e-02	0.402579879	0.9546035	0.2377220295	1.774656e-01
**AP2B1**	0.0912682805	4.323428e-01	7.997328e-01	-0.1515445292	0.200711647	0.9995992	-2.552502e-02	0.588772337	0.9546035	-0.0279537308	5.979467e-01
**VAMP2;VAMP3**	-0.0757770337	8.723978e-01	9.614924e-01	0.2904630565	0.525986694	0.9995992	-3.660498e-01	0.780147101	0.9572213	0.4677984572	1.515312e-01
**GNAS**	-0.4980740933	3.744140e-01	7.647336e-01	0.4111675263	0.456755082	0.9995992	-1.789097e-02	0.512770275	0.9546035	0.6561594967	1.207615e-01
**YWHAZ**	-0.0153459890	8.360457e-01	9.500766e-01	0.0254184211	0.738887114	0.9995992	-3.533721e-02	0.680278479	0.9546035	0.0231195803	3.822384e-01
**DYNLL1**	-0.0426711090	6.671916e-01	8.576613e-01	-0.0612562675	0.522148839	0.9995992	-1.416000e-02	0.555963166	0.9546035	0.0184328234	4.252838e-01
**SKP1**	0.0528407451	5.076780e-01	8.151018e-01	-0.0645315887	0.432292502	0.9995992	1.031697e-01	0.098969161	0.9546035	0.0621295729	2.312048e-01
**EIF5A**	-0.0091031941	9.069694e-01	9.710488e-01	-0.0854143447	0.269157754	0.9995992	6.997862e-02	0.187264870	0.9546035	0.0102353526	4.508057e-01
**TMSB10**	-0.0813313147	5.414716e-01	8.281031e-01	0.0377621489	0.777465569	0.9995992	-1.108475e-01	0.791401350	0.9572213	-0.0847884953	7.353813e-01
**PPP2CA**	-0.0942054541	2.786058e-01	6.987920e-01	0.0019632455	0.982252157	0.9995992	8.231741e-02	0.177008714	0.9546035	0.1126137834	1.065962e-01
**YBX1**	-0.4027260167	3.616575e-01	7.553917e-01	-0.1875512979	0.667289752	0.9995992	1.370061e-01	0.381648129	0.9546035	0.3095648251	2.393777e-01
**TPM4**	0.0946764691	1.178777e-01	5.618203e-01	0.0155247687	0.799838980	0.9995992	-1.090264e-01	0.961927335	0.9774190	-0.0148461421	5.980513e-01
**UBE2L3**	0.1703525351	1.948942e-03	1.319384e-01	0.0498361684	0.398550665	0.9995992	6.701101e-02	0.122084393	0.9546035	0.1187491673	2.748688e-02
**EEF1A1;EEF1A1P5**	-0.0909397352	4.937294e-01	8.151018e-01	-0.1392098381	0.288899947	0.9995992	1.654887e-01	0.112424897	0.9546035	-0.1849865866	9.162180e-01
**TUBA1B**	-0.1944538225	5.676836e-02	4.562264e-01	0.0303419907	0.779721625	0.9995992	-1.714906e-02	0.565732554	0.9546035	0.0804225918	2.410522e-01
**TUBA4A**	-0.2550718690	1.671840e-02	3.187282e-01	-0.0283393048	0.800728916	0.9995992	-2.015769e-01	0.967437345	0.9774190	-0.0988319172	8.080756e-01
**TUBB4B**	-0.0905613077	3.282074e-01	7.272974e-01	0.0327254636	0.726621852	0.9995992	-1.115767e-01	0.885149358	0.9579808	0.0285054905	3.829239e-01
**PAFAH1B2**	0.0020650461	9.829879e-01	9.936282e-01	-0.0336945941	0.726121770	0.9995992	-7.744516e-02	0.782409689	0.9572213	-0.0199640334	5.795069e-01
**H3C1;H3-4;H3C15**	-0.5445432593	1.951203e-01	6.539918e-01	0.0705490911	0.865071899	0.9995992	-1.935946e-02	0.518270088	0.9546035	0.3696582837	1.901621e-01
**HBB**	0.0372967496	6.127998e-01	8.419469e-01	0.0676443809	0.365394877	0.9995992	5.060108e-02	0.250774085	0.9546035	0.1103061400	6.785153e-02
**HBG1**	0.0311543332	7.494268e-01	9.039005e-01	0.0992569260	0.309356387	0.9995992	1.433884e-01	0.070973682	0.9546035	0.1596351358	5.385429e-02
**HBG2**	-0.4047241693	5.108898e-01	8.160429e-01	0.4230669553	0.481526516	0.9995992	-6.077134e-02	0.537483182	0.9546035	-0.3817572003	7.397950e-01
**HBA1**	0.0212131204	8.540300e-01	9.533502e-01	0.0773957079	0.493296667	0.9995992	6.491042e-03	0.477903091	0.9546035	0.1116449552	1.609535e-01
**IGBP1**	0.2379212188	1.584230e-01	6.224838e-01	0.1161811907	0.498724348	0.9995992	2.349464e-01	0.082496909	0.9546035	0.1660224643	1.753759e-01
**SIRPA;SIRPB1;SIRPG**	0.1427898106	6.991280e-01	8.689477e-01	0.3062513966	0.384065444	0.9995992	-4.409152e-01	0.881238475	0.9579808	0.0623321644	4.253563e-01
**CCT2**	-0.0012307942	9.853647e-01	9.946209e-01	0.0423927741	0.544043065	0.9995992	5.236290e-03	0.469015843	0.9546035	0.0157832806	4.118813e-01
**KRT83**	0.1358491069	2.321836e-01	6.697768e-01	0.0426424649	0.703924552	0.9995992	-1.066664e-01	0.820357047	0.9572213	0.0004318660	4.984553e-01
**GSTO1**	0.2636871736	5.048901e-03	2.131357e-01	-0.0043822160	0.964854479	0.9995992	-6.585055e-02	0.745139527	0.9546035	0.0196563736	4.205653e-01
**GPLD1**	-0.0840086561	4.546710e-01	8.045226e-01	0.1100459308	0.320664767	0.9995992	-5.078804e-02	0.669908811	0.9546035	0.0096483225	4.645331e-01
**LCN2**	0.3735093243	3.315269e-02	4.027342e-01	0.0751537295	0.672769014	0.9995992	-7.708464e-02	0.664232182	0.9546035	0.0420596648	4.101631e-01
**S100A12**	0.2015366362	2.334369e-01	6.697768e-01	0.0319571952	0.850530994	0.9995992	-8.722886e-02	0.693148405	0.9546035	0.1409899960	2.054241e-01
**BASP1**	-0.3060125413	4.970183e-01	8.151018e-01	-0.4346377072	0.369027415	0.9995992	-4.765722e-01	0.853115456	0.9572213	-0.5392277156	8.490386e-01
**IGLV3-21**	0.3409232397	4.481401e-01	8.037961e-01	-0.0466725419	0.919050390	0.9995992	-3.983228e-01	0.808920272	0.9572213	-0.3518786457	7.619635e-01
**HSPG2**	0.1480903292	5.138182e-01	8.185083e-01	0.0354051589	0.878938729	0.9995992	-1.852633e-01	0.791650979	0.9572213	0.0537414972	4.116722e-01
**MPP1**	-0.0912878917	6.468251e-01	8.489971e-01	-0.0386074843	0.848412401	0.9995992	-2.643425e-02	0.552446543	0.9546035	0.2564841281	1.065693e-01
**PITPNA**	-0.1744254032	1.507928e-01	6.224838e-01	-0.1084106797	0.381829109	0.9995992	-5.309573e-03	0.517070703	0.9546035	-0.0887511512	7.584771e-01
**SLC25A3**	-0.6471755524	1.065601e-01	5.382651e-01	-0.2801491193	0.489904180	0.9995992	-2.056916e-01	0.694083771	0.9546035	0.0170693952	4.835039e-01
**PURA**	0.2410705019	6.153129e-01	8.419469e-01	-0.8399800505	0.098567215	0.9995992	4.439883e-01	0.167348324	0.9546035	-0.3459009555	7.584350e-01
**CLTC**	0.0894591752	5.633292e-01	8.323189e-01	0.1754614454	0.265729402	0.9995992	-1.003023e-01	0.747153612	0.9546035	0.0737563031	3.313230e-01
**FKBP3**	-0.1227740101	3.622505e-01	7.553917e-01	-0.0679846132	0.621023278	0.9995992	1.106253e-01	0.213610985	0.9546035	-0.1127899677	7.888997e-01
**REEP5**	-0.8791350270	1.082319e-01	5.408542e-01	-0.1697540075	0.749701801	0.9995992	2.357249e-01	0.332477107	0.9546035	0.5565236695	1.424543e-01
**SORD**	0.0772155586	4.534191e-01	8.045226e-01	-0.0471565345	0.656131452	0.9995992	-7.042802e-02	0.746325427	0.9546035	-0.0108754778	5.417281e-01
**AMPD3**	0.0439314537	7.729005e-01	9.209358e-01	-0.2073745572	0.168835843	0.9995992	-2.919523e-02	0.574322961	0.9546035	-0.0890598169	7.175060e-01
**FABP5**	-0.0034841572	9.727640e-01	9.926294e-01	0.1075367099	0.280363911	0.9995992	1.552626e-01	0.069265652	0.9546035	0.1694233235	4.035720e-02
**CAP1**	-0.0520715254	5.843748e-01	8.403054e-01	-0.0668288542	0.496407142	0.9995992	1.084192e-02	0.456071284	0.9546035	0.0015536515	4.934849e-01
**RHAG**	-0.1465580049	7.944630e-01	9.305665e-01	0.2550211270	0.634223108	0.9995992	-8.614528e-02	0.560818865	0.9546035	0.6924137178	9.457872e-02
**MAP2K1**	0.0339084043	5.968447e-01	8.419469e-01	-0.0351102024	0.578997447	0.9995992	3.638521e-02	0.287998896	0.9546035	-0.0248705607	6.521639e-01

**FKBP4**	0.1066968199	5.211716e-01	8.216873e-01	-0.0217878579	0.892922297	0.9995992	-1.255395e-01	0.773192586	0.9572213	0.1191599796	2.203651e-01
**CFHR3**	-0.1242087181	2.434710e-01	6.725635e-01	0.0579642715	0.586928157	0.9995992	5.268850e-02	0.311773684	0.9546035	-0.0565495918	6.912211e-01
**CFHR1**	-0.0894735923	4.722100e-01	8.125882e-01	-0.1607458591	0.188364245	0.9995992	-1.041975e-01	0.795416783	0.9572213	-0.3265010396	9.952505e-01
**UBXN1**	0.0365651810	6.762689e-01	8.610482e-01	0.0387134200	0.645261417	0.9995992	-9.114723e-03	0.541224320	0.9546035	0.0137593843	4.326331e-01
**GBE1**	-0.0231282307	8.265774e-01	9.430642e-01	0.0436421113	0.687370749	0.9995992	-4.145232e-02	0.652067296	0.9546035	0.0775162356	2.486608e-01
**HGFAC**	-0.1690489444	1.740488e-01	6.291668e-01	-0.1318163715	0.283724693	0.9995992	3.678473e-04	0.498865361	0.9546035	-0.0420615901	6.349148e-01
**GLO1**	0.2008689474	4.870294e-03	2.131357e-01	0.0457284757	0.534536944	0.9995992	7.679064e-02	0.150808393	0.9546035	0.1295717920	3.392774e-02
**YWHAH**	0.1138939385	3.109593e-01	7.192836e-01	-0.0035912263	0.975821724	0.9995992	8.357056e-02	0.236561089	0.9546035	-0.1139548187	8.224211e-01
**PLP2**	-0.2712812332	5.461547e-01	8.281031e-01	-0.0524774085	0.907189510	0.9995992	-2.597999e-01	0.718490087	0.9546035	0.2958183579	2.576441e-01
**CLC**	-0.2745298569	3.333753e-01	7.337982e-01	0.3555068943	0.208649822	0.9995992	-4.131672e-02	0.557836495	0.9546035	-0.1527303699	6.942367e-01
**CALD1**	-0.2457610534	1.585174e-01	6.224838e-01	-0.0187224621	0.916254389	0.9995992	2.362539e-02	0.447149308	0.9546035	-0.0068333264	5.147598e-01
**ITIH3**	0.0692743859	7.448141e-01	9.009472e-01	-0.1005454479	0.631738976	0.9995992	-2.287423e-01	0.856735600	0.9572213	-0.4515243335	9.746749e-01
**PTPN11**	0.1235677748	2.638743e-01	6.900429e-01	0.2685426151	0.015802455	0.9995992	1.381762e-01	0.103614537	0.9546035	0.2550533570	1.547331e-02
**PSME1**	0.0666075307	3.189294e-01	7.225946e-01	0.0944984048	0.168628235	0.9995992	-6.550339e-02	0.832762981	0.9572213	0.1095991430	6.554072e-02
**PRDX1**	0.1736439472	1.022660e-02	2.878058e-01	0.0327446603	0.656007158	0.9995992	3.026209e-02	0.336827704	0.9546035	0.0350409358	3.145013e-01
**ARHGAP1**	-0.0385553822	6.933344e-01	8.660792e-01	0.0975372527	0.304022389	0.9995992	4.501036e-02	0.325908900	0.9546035	0.0718357297	2.254744e-01
**DHX9**	0.0785143035	5.611729e-01	8.323189e-01	0.0504177606	0.713049904	0.9995992	1.756954e-01	0.098178372	0.9546035	0.1381325301	1.644588e-01
**LGALS3BP**	-0.2029052482	3.884249e-01	7.742297e-01	-0.2221004023	0.351974696	0.9995992	-1.476022e-01	0.732896240	0.9546035	-0.6164281297	9.932663e-01
**DMTN**	0.0825183332	1.712980e-01	6.287166e-01	-0.0138467406	0.823832026	0.9995992	-7.817878e-03	0.550116948	0.9546035	0.0290238970	3.207790e-01
**CD47**	-0.4455247151	3.515137e-01	7.498135e-01	0.0389736070	0.934621200	0.9995992	3.657286e-02	0.469656697	0.9546035	0.5700957312	1.126522e-01
**VAC14**	-0.1123850552	4.430735e-01	8.021362e-01	-0.0877871283	0.569831810	0.9995992	-1.312165e-01	0.811978246	0.9572213	-0.0530974123	6.261650e-01
**AHNAK**	-0.1739821520	3.140319e-01	7.197712e-01	-0.0182065093	0.916925271	0.9995992	-1.090563e-01	0.733312601	0.9546035	-0.0519910940	6.138862e-01
**AP1B1**	0.1251949281	2.663184e-01	6.940168e-01	-0.1225692299	0.291268611	0.9995992	9.636704e-02	0.196858538	0.9546035	-0.0301607499	6.051004e-01
**EFEMP1**	-0.0783479369	5.443563e-01	8.281031e-01	0.0287392857	0.817814323	0.9995992	-8.340335e-02	0.740296806	0.9546035	-0.0364880065	6.097583e-01
**LMAN2**	-0.3711429224	3.641075e-01	7.553917e-01	0.0490824906	0.905968229	0.9995992	-1.736454e-02	0.516783537	0.9546035	0.3367261219	2.085729e-01
**PTPRJ**	0.2033610283	5.721470e-01	8.344560e-01	-0.1091212705	0.767672269	0.9995992	4.233044e-02	0.454555201	0.9546035	-0.1827970353	6.828396e-01
**COASY**	0.1460531661	5.888118e-02	4.562264e-01	0.0063908861	0.934146790	0.9995992	-7.910955e-02	0.843260225	0.9572213	0.0077041429	4.591205e-01
**GPS1**	-0.0078108992	9.067151e-01	9.710488e-01	-0.0170361793	0.797938771	0.9995992	2.990459e-02	0.330622919	0.9546035	0.0595678935	1.997683e-01
**SPP2**	-0.3442486043	3.867452e-01	7.727636e-01	-0.3730992412	0.379078122	0.9995992	-4.146417e-01	0.854003887	0.9572213	-0.3306026024	7.653025e-01
**MTAP**	-0.0734584552	2.232170e-01	6.697768e-01	0.0056471433	0.928887631	0.9995992	-1.814529e-02	0.616254455	0.9546035	-0.0191514941	6.180011e-01
**PSMD2**	0.0882842597	6.085159e-01	8.419469e-01	-0.2153842524	0.242192571	0.9995992	-1.066263e-01	0.730603732	0.9546035	-0.1816890341	8.177785e-01
**MMRN1**	-0.3497505184	3.942983e-02	4.198744e-01	-0.2618537765	0.131302270	0.9995992	-2.014933e-01	0.874516226	0.9572213	-0.3616491892	9.802025e-01
**SELENBP1**	-0.0593280168	6.453283e-01	8.488673e-01	0.0050631713	0.968065616	0.9995992	-3.872318e-02	0.616109105	0.9546035	0.0896887242	2.336620e-01
**SLC14A1**	-0.4389199060	4.604430e-01	8.069339e-01	0.1961075277	0.734735158	0.9995992	-3.526420e-01	0.724987762	0.9546035	0.4564092734	2.161713e-01
**UBE2V1**	0.0243508894	7.537057e-01	9.072100e-01	-0.0186204598	0.816611232	0.9995992	3.049649e-02	0.350176319	0.9546035	0.0295736384	3.570263e-01
**ILK**	-0.1754711220	2.433562e-01	6.725635e-01	-0.0904950306	0.553770010	0.9995992	-8.064690e-02	0.699925019	0.9546035	-0.1531301863	8.323548e-01
**PIN1**	0.2632814888	1.009497e-03	1.319384e-01	-0.0385699658	0.649788067	0.9995992	-1.592248e-02	0.574955789	0.9546035	-0.0164116084	5.769882e-01
**EIF4EBP1**	0.3234612914	2.737957e-01	6.944775e-01	-0.0051377816	0.986208531	0.9995992	3.702569e-01	0.111322222	0.9546035	0.2632922441	1.834560e-01
**DCTN2**	-0.1286689799	2.613932e-01	6.900429e-01	-0.0796245659	0.486285454	0.9995992	1.955395e-01	0.045556040	0.9546035	-0.0260719359	5.901018e-01
**NAE1**	0.0781673570	4.276487e-01	7.973735e-01	0.0256674207	0.795188834	0.9995992	-3.531267e-02	0.637102586	0.9546035	0.0900575797	1.881905e-01
**CUL1**	0.2217996278	6.256598e-03	2.550103e-01	0.0291650916	0.733152305	0.9995992	5.460318e-02	0.263240959	0.9546035	0.1427801496	5.219331e-02
**CUL2**	0.0470276778	6.417015e-01	8.486349e-01	-0.0324314622	0.754112390	0.9995992	-1.673118e-02	0.564220957	0.9546035	0.0253474783	4.073603e-01
**CUL3**	-0.1055578152	1.720820e-01	6.287166e-01	-0.1068776590	0.162514891	0.9995992	-4.809888e-02	0.731805272	0.9546035	-0.1044467405	9.120938e-01
**TSTA3**	0.0808785527	4.136918e-01	7.920435e-01	-0.0739151151	0.486189552	0.9995992	-3.940423e-02	0.651042761	0.9546035	-0.2177430435	9.765518e-01
**FHL1**	-0.4247534130	1.705445e-01	6.287166e-01	-0.1957193605	0.552098726	0.9995992	-3.648845e-01	0.879759456	0.9579808	-0.1503421635	6.676547e-01
**APOF**	0.0303140728	8.237004e-01	9.429827e-01	-0.0302140787	0.819331064	0.9995992	6.267043e-02	0.326824364	0.9546035	-0.0491772154	6.490058e-01
**BLMH**	0.1051928847	1.275302e-01	5.775504e-01	-0.0528198738	0.459908804	0.9995992	-2.340605e-02	0.627828697	0.9546035	-0.0735466884	8.428549e-01
**COTL1**	0.1384939451	4.931277e-01	8.151018e-01	0.4483212427	0.021638129	0.9995992	-6.696424e-02	0.629345977	0.9546035	0.3857494333	2.456735e-02
**HNRNPD**	0.3703327734	6.129914e-02	4.562264e-01	-0.1223589878	0.561047472	0.9995992	-2.243022e-01	0.872394493	0.9572213	-0.0408116921	5.720099e-01
**DSG2**	-1.6549915304	4.025104e-01	7.844497e-01	-1.8579443896	0.378083040	0.9995992	-1.564327e+00	0.780767724	0.9572213	-1.9898023235	8.133669e-01
**TTLL12**	0.2073159166	5.532618e-02	4.562264e-01	0.1491892656	0.194887823	0.9995992	-3.320082e-02	0.617955485	0.9546035	0.2104580675	2.629197e-02
**DCTN1**	-0.0993706281	4.246677e-01	7.973735e-01	-0.0774788149	0.542401668	0.9995992	1.135305e-01	0.184470019	0.9546035	-0.0103109229	5.327314e-01
**EIF4A2**	0.0524958728	6.661033e-01	8.576613e-01	-0.0898114664	0.461258744	0.9995992	1.987285e-01	0.050930210	0.9546035	0.0764964315	2.601952e-01
**CTTN**	-0.1373879274	1.393691e-01	6.012201e-01	-0.1298369750	0.170391093	0.9995992	-1.359965e-02	0.558196772	0.9546035	-0.0936634390	8.255008e-01
**FLOT2**	-0.3752512385	4.079166e-01	7.865537e-01	-0.1031904550	0.821921059	0.9995992	-3.522830e-02	0.530886022	0.9546035	0.4474498685	1.658384e-01
**WFDC2**	1.4142239543	4.909908e-02	4.562264e-01	0.8638734014	0.221641424	0.9995992	6.603403e-01	0.167807184	0.9546035	0.9994397564	8.676349e-02
**HABP2**	-0.2128120365	1.570559e-02	3.187282e-01	-0.0720336862	0.421991959	0.9995992	-2.879287e-02	0.622550488	0.9546035	-0.1740097349	9.769102e-01
**PRPSAP1**	0.0410932183	6.165708e-01	8.419469e-01	0.0228288755	0.785317088	0.9995992	8.598894e-02	0.150714325	0.9546035	0.1126958914	8.693135e-02
**ITIH4**	-0.0560604878	5.617811e-01	8.323189e-01	-0.1510758110	0.124877568	0.9995992	-1.033552e-01	0.851676519	0.9572213	-0.1967655995	9.819496e-01
**GANAB**	-0.1930894877	3.623884e-01	7.553917e-01	0.0554311305	0.790685397	0.9995992	1.725643e-01	0.209473116	0.9546035	0.3456981728	5.064105e-02
**PPP2R5D**	-0.1709011752	2.150798e-01	6.680462e-01	-0.0250054836	0.854580658	0.9995992	-1.417058e-01	0.841138046	0.9572213	-0.0560217385	6.653184e-01
**LTBP1**	-0.4079135079	4.785755e-02	4.562264e-01	-0.0159598982	0.939835062	0.9995992	-3.492870e-01	0.950487331	0.9714819	-0.1756046316	7.987174e-01
**ICAM4**	-0.4374879241	2.714366e-01	6.940168e-01	0.2251208719	0.567665980	0.9995992	-1.245259e-02	0.512236033	0.9546035	0.5233352199	9.958509e-02
**LASP1**	0.0578565466	4.997228e-01	8.151018e-01	0.0340414896	0.689830448	0.9995992	-3.047386e-02	0.636573150	0.9546035	-0.0231917745	6.044449e-01
**KPNB1**	0.0340026303	8.487581e-01	9.510832e-01	-0.1999187541	0.275882148	0.9995992	-1.055171e-01	0.724171685	0.9546035	-0.2037210376	8.686575e-01
**GAPVD1**	0.0049096448	9.395670e-01	9.832514e-01	-0.0171703853	0.795217668	0.9995992	-1.276090e-02	0.578651423	0.9546035	-0.0290488881	6.653198e-01
**PSMD6**	0.0912587533	2.085830e-01	6.658594e-01	-0.0477597785	0.512350691	0.9995992	5.042086e-02	0.248060550	0.9546035	0.0322364566	3.366798e-01
**ARL6IP1**	-0.5197463791	5.557818e-01	8.319734e-01	-1.1217156586	0.231732949	0.9995992	8.031188e-01	0.185138565	0.9546035	0.6096708315	2.413650e-01
**PDIA6**	0.0109758039	9.571923e-01	9.907192e-01	-0.1315043358	0.526556067	0.9995992	1.159257e-01	0.290364584	0.9546035	0.0347549026	4.304518e-01
**PAFAH1B3**	-0.0476008299	4.661726e-01	8.079413e-01	0.0117912262	0.854593655	0.9995992	6.453497e-02	0.164102788	0.9546035	0.1170572052	3.391970e-02
**PCOLCE**	0.0893181163	8.600805e-01	9.551688e-01	0.4145883455	0.404033945	0.9995992	3.565255e-01	0.244725445	0.9546035	0.3743469039	2.262583e-01
**PPA1**	0.0207897435	8.431840e-01	9.500891e-01	0.0925681324	0.374712919	0.9995992	7.011427e-02	0.259733895	0.9546035	0.0899562290	1.968635e-01
**PTGES3**	-0.0160901609	8.462582e-01	9.508196e-01	0.1054690134	0.211543860	0.9995992	6.849255e-02	0.208632005	0.9546035	0.0013665996	4.934154e-01
**PTPA**	0.1316992976	1.093003e-01	5.428275e-01	0.0280224215	0.742095688	0.9995992	-2.456063e-02	0.613202598	0.9546035	0.0893630556	1.504057e-01
**RAB35**	-0.9601457929	7.670326e-02	4.905904e-01	-0.3489865787	0.511327017	0.9995992	9.878908e-02	0.426011433	0.9546035	0.1066621735	4.212389e-01
**KRT31**	-0.7091955840	3.451469e-01	7.471861e-01	-0.7063185436	0.379510908	0.9995992	-7.569190e-01	0.842489542	0.9572213	-0.9516326630	8.627945e-01
**TMED2**	-0.2440499811	4.954530e-01	8.151018e-01	-0.2838455601	0.418991595	0.9995992	-4.181244e-01	0.874226497	0.9572213	0.1791218471	3.067171e-01
**PCBP1**	0.0445321980	5.449908e-01	8.281031e-01	0.0218792058	0.765299630	0.9995992	2.366568e-02	0.370527591	0.9546035	0.0904974965	1.316489e-01
**PCBP2**	-0.0517074446	4.700005e-01	8.121938e-01	-0.0906624748	0.206047106	0.9995992	-6.258263e-02	0.808193075	0.9572213	-0.0518325129	7.519474e-01
**ELOC**	-0.0349832547	7.819382e-01	9.256765e-01	0.1040294115	0.390932705	0.9995992	1.244948e-01	0.168367879	0.9546035	0.0091370696	4.691815e-01
**ELOB**	-0.0206049467	8.424617e-01	9.500891e-01	-0.0842774846	0.424838721	0.9995992	-5.574463e-02	0.707085255	0.9546035	-0.1242057764	8.779657e-01
**RSU1**	-0.1686308077	2.989710e-02	3.864738e-01	0.0350247402	0.665805618	0.9995992	-5.715314e-02	0.760204540	0.9559168	-0.0113954160	5.536890e-01
**PPP1R7**	-0.0347856648	6.696021e-01	8.576613e-01	0.0056905723	0.944412091	0.9995992	6.292799e-02	0.223780974	0.9546035	0.1164051924	8.033065e-02
**FCN2**	0.5664853270	2.284653e-01	6.697768e-01	-0.6995318334	0.149024189	0.9995992	1.503430e-01	0.375122855	0.9546035	-0.3361504105	7.488303e-01
**TGFBI**	0.1053329451	5.036120e-01	8.151018e-01	0.0641558450	0.676863769	0.9995992	-4.871767e-02	0.623681005	0.9546035	-0.0850692497	6.806312e-01
**TSN**	-0.0384788598	5.084501e-01	8.151018e-01	0.0033879158	0.952605784	0.9995992	-5.119469e-02	0.806203777	0.9572213	-0.0201534732	6.442413e-01
**MAPRE1**	0.0468820166	5.748684e-01	8.347598e-01	-0.1170931273	0.162117956	0.9995992	-4.327783e-02	0.692097005	0.9546035	-0.0553001343	7.320481e-01
**UBE2V2**	0.0138471853	8.794137e-01	9.651504e-01	-0.0168762240	0.854242373	0.9995992	-6.347329e-02	0.750470485	0.9546035	0.0015727272	4.930657e-01
**NEDD8**	0.3393034755	3.621962e-02	4.077295e-01	0.2022866144	0.205148800	0.9995992	-1.144534e-01	0.751886157	0.9546035	0.0999360492	2.704376e-01
**ADIPOQ**	-0.0969720888	6.548635e-01	8.524806e-01	-0.0352941311	0.871230640	0.9995992	1.354576e-01	0.270297331	0.9546035	0.0096638651	4.832548e-01
**ZYX**	-0.0165209792	8.958736e-01	9.687085e-01	0.0118858519	0.924055330	0.9995992	-1.416951e-01	0.865312152	0.9572213	-0.0748157174	7.281044e-01
**ADRM1**	0.3323801451	1.288852e-01	5.792482e-01	-0.2533748641	0.262752184	0.9995992	2.191699e-01	0.165809612	0.9546035	0.1335557414	2.779690e-01
**PSMD5**	0.0591687391	5.601936e-01	8.323189e-01	0.1217435179	0.229575249	0.9995992	4.748418e-02	0.324371974	0.9546035	0.2306525661	1.039696e-02
**DDB1**	-0.0543428249	4.848954e-01	8.129736e-01	0.0376419878	0.643960881	0.9995992	-5.531328e-02	0.758774779	0.9559168	0.0795684744	1.747999e-01
**CDC37**	-0.0004555940	9.982178e-01	9.990571e-01	0.0673237470	0.731101470	0.9995992	-2.990016e-01	0.927603394	0.9658352	-0.0086969652	5.182351e-01
**DPYSL2**	-0.0444897003	8.305782e-01	9.460034e-01	-0.0195455071	0.927714331	0.9995992	-2.067999e-01	0.832802536	0.9572213	-0.2514000824	8.699939e-01
**ECM1**	-0.2418561285	6.731498e-02	4.639407e-01	-0.2286391184	0.092628856	0.9995992	-1.628226e-01	0.884718551	0.9579808	-0.2278786829	9.465970e-01
**FSCN1**	-0.0680254201	4.176892e-01	7.924697e-01	-0.0116695532	0.889061991	0.9995992	5.097050e-02	0.272694492	0.9546035	0.0986717762	1.214080e-01
**GUK1**	-0.0277065242	7.889093e-01	9.269293e-01	-0.0744576698	0.474393393	0.9995992	1.441100e-01	0.080614117	0.9546035	-0.0436145533	6.533847e-01
**HAGH**	-0.0061636011	9.356805e-01	9.822153e-01	-0.0327695559	0.666577356	0.9995992	6.162668e-02	0.209641021	0.9546035	0.0268666334	3.676079e-01
**UGP2**	0.1093010649	5.463306e-02	4.562264e-01	0.0265521722	0.652028974	0.9995992	2.170847e-02	0.357721818	0.9546035	0.0825238047	7.620885e-02
**TXNRD1**	-0.0566197906	6.854384e-01	8.640904e-01	0.0531214876	0.708161207	0.9995992	-1.164831e-01	0.795638936	0.9572213	-0.0783548785	7.057956e-01
**IAH1**	0.1678451116	1.570224e-01	6.224838e-01	0.0971656453	0.419515058	0.9995992	-1.079942e-01	0.811071804	0.9572213	0.0208026994	4.316381e-01
**TKFC**	0.0305586307	7.208963e-01	8.833575e-01	-0.0320468435	0.706124364	0.9995992	-1.231471e-01	0.921055752	0.9644203	-0.0094438057	5.431514e-01
**VPS26B**	0.0585960214	8.455000e-01	9.508196e-01	0.2661875389	0.350104304	0.9995992	1.389911e-01	0.324284425	0.9546035	0.2702247487	1.582127e-01
**TP53I3**	0.0774313959	6.079237e-01	8.419469e-01	-0.0303873385	0.847580982	0.9995992	-2.398614e-02	0.561026589	0.9546035	0.0083368446	4.772886e-01
**PYCR3**	-0.1475248157	5.076465e-01	8.151018e-01	0.2330913940	0.306986334	0.9995992	2.085203e-01	0.178567309	0.9546035	0.2593658416	1.275889e-01
**CYBRD1**	-0.2837814069	5.025705e-01	8.151018e-01	0.0378296832	0.929065448	0.9995992	-6.819818e-02	0.563343461	0.9546035	0.4385003249	1.484191e-01
**PAGE2B;PAGE2**	-0.0092568295	9.680500e-01	9.923248e-01	-0.0645187862	0.773168122	0.9995992	5.252012e-01	0.012329327	0.9546035	0.1203302205	3.030391e-01
**WIPI1**	0.1976736976	4.398009e-01	8.021362e-01	0.1361263837	0.603160677	0.9995992	5.879483e-02	0.411857691	0.9546035	0.0890341928	3.653910e-01
**TBCEL**	0.0271483601	7.191016e-01	8.833575e-01	-0.0502271152	0.513951503	0.9995992	-6.046852e-02	0.791650006	0.9572213	-0.0078217646	5.414639e-01
**TTC38**	-0.0140462252	9.686814e-01	9.923248e-01	-0.3549917423	0.317692204	0.9995992	-5.467292e-01	0.931877175	0.9667703	-0.5153323028	9.293767e-01
**UBR4**	0.0460659878	5.876171e-01	8.419469e-01	-0.0248815564	0.775867761	0.9995992	1.644620e-01	0.025737264	0.9546035	0.1167260831	1.080854e-01
**C9orf64**	0.0811091845	8.898420e-01	9.687085e-01	-0.6467347144	0.292906619	0.9995992	2.368869e-01	0.345442585	0.9546035	-0.0990481152	5.655768e-01
**DDI2**	-0.1245269649	2.323952e-01	6.697768e-01	-0.0651974618	0.538004845	0.9995992	-2.692916e-02	0.598886613	0.9546035	-0.0270895847	5.988440e-01
**YOD1**	-0.1340170856	1.676815e-01	6.287166e-01	0.0916181447	0.341048529	0.9995992	3.918775e-02	0.347864234	0.9546035	0.1513110112	5.870882e-02
**BROX**	0.1129879083	1.562082e-01	6.224838e-01	0.0908046752	0.265754325	0.9995992	-4.076264e-02	0.691748006	0.9546035	0.0761864697	1.886589e-01
**RNF123**	0.0320746024	6.582392e-01	8.524806e-01	-0.0499094292	0.503745471	0.9995992	1.020551e-01	0.080957622	0.9546035	0.0749083997	1.702141e-01
**HBM**	-0.0523206475	8.375464e-01	9.500766e-01	0.0736658829	0.777918207	0.9995992	9.192393e-02	0.364889475	0.9546035	0.0936921834	3.452576e-01
**VASN**	-0.0084055113	9.645857e-01	9.923248e-01	-0.2386252408	0.230094275	0.9995992	-3.485026e-02	0.571031474	0.9546035	-0.0680706716	6.385555e-01
**CIAPIN1**	-0.3338000761	3.244732e-01	7.236365e-01	0.0590399679	0.863064846	0.9995992	1.195164e-01	0.365733274	0.9546035	-0.1878180356	7.099135e-01
**PTRHD1**	-0.0091925175	9.490820e-01	9.901279e-01	-0.0949594327	0.519218710	0.9995992	-9.990485e-02	0.752822972	0.9546035	-0.0132728604	5.350769e-01
**TANGO2**	-0.0599629604	5.329481e-01	8.281031e-01	-0.0776358665	0.424089957	0.9995992	-2.382792e-03	0.509601366	0.9546035	-0.0664221765	7.501564e-01
**PDE12**	0.1131557261	4.764639e-01	8.125882e-01	0.0886912614	0.584227923	0.9995992	-7.701166e-03	0.519147175	0.9546035	0.1810039007	1.425298e-01
**PLBD1**	0.1889314964	6.060321e-01	8.419469e-01	-0.1124285217	0.756450855	0.9995992	-1.615575e-01	0.667006459	0.9546035	-0.0662365733	5.704759e-01
**PGM2L1**	-0.0899950513	3.891519e-01	7.743731e-01	0.0037338200	0.971971414	0.9995992	-9.727033e-02	0.818131109	0.9572213	0.0461666650	3.390190e-01
**NCEH1**	0.1331716576	6.320379e-01	8.451005e-01	-0.0001397780	0.999599219	0.9995992	2.048921e-01	0.230416901	0.9546035	0.2926065216	1.499474e-01
**DHRS11**	0.1939954122	1.005833e-01	5.314990e-01	-0.0133320390	0.909378905	0.9995992	-1.561088e-01	0.900518158	0.9579808	-0.0466821324	6.551573e-01
**SBSN**	-0.9800049521	5.492664e-01	8.281031e-01	-0.4389300885	0.776205965	0.9995992	1.808448e+00	0.157268903	0.9546035	1.2381994617	2.154761e-01
**PI16**	-0.1990042852	3.959663e-01	7.802736e-01	0.0402953859	0.859963731	0.9995992	-2.116347e-01	0.823202958	0.9572213	-0.3832060473	9.352860e-01
**NAPRT**	0.1344796775	2.212616e-01	6.697768e-01	0.1474043711	0.187661500	0.9995992	4.975857e-02	0.327545549	0.9546035	0.2482429813	1.559689e-02
**TUBA1A**	-0.0285378690	8.053447e-01	9.350859e-01	-0.1217647179	0.303899466	0.9995992	-1.659126e-01	0.922827683	0.9644203	-0.0745939988	7.276243e-01
**COPS6**	0.2011785237	2.723035e-03	1.463013e-01	-0.0058982221	0.931377512	0.9995992	5.046581e-02	0.241451176	0.9546035	0.0450244775	2.641970e-01
**ENOSF1**	-0.5219317903	2.089965e-01	6.658594e-01	-0.1876547328	0.653248401	0.9995992	-1.883481e-01	0.674593290	0.9546035	-0.2482524334	7.181633e-01
**MOB1B;MOB1A**	-0.1131644020	3.427948e-01	7.434558e-01	-0.1343703341	0.277877944	0.9995992	4.800199e-03	0.484421259	0.9546035	-0.2520030876	9.822846e-01
**DCXR**	0.1012639151	1.674584e-01	6.287166e-01	0.0499351063	0.513091014	0.9995992	6.367734e-02	0.201172232	0.9546035	0.0582342839	2.188120e-01
**HUWE1**	-0.2857638414	3.283404e-01	7.272974e-01	-0.4256301644	0.172523376	0.9995992	-1.312578e-01	0.671753740	0.9546035	-0.4269193827	8.985717e-01
**FERMT3**	-0.0324979248	6.908187e-01	8.660792e-01	-0.0111436441	0.893636512	0.9995992	4.079776e-02	0.311575189	0.9546035	0.0254380719	3.831092e-01
**CAND1**	0.2253636654	6.630374e-02	4.639407e-01	0.0385713574	0.756390801	0.9995992	2.782184e-01	0.009973043	0.9546035	0.2326687370	2.823088e-02

NR2C2AP	0.2351447992	6.807919e-01	8.624823e-01	0.6781703607	0.239162991	0.9995992	-1.964194e-01	0.635325902	0.9546035	0.0852952732	4.418388e-01
CARM1	-0.0241948448	8.108538e-01	9.384947e-01	-0.0574194153	0.573915982	0.9995992	-7.082692e-02	0.754685539	0.9546035	-0.0483711302	6.764543e-01
NIT1	0.0406256068	4.973558e-01	8.151018e-01	-0.0576792885	0.340316425	0.9995992	-2.872145e-02	0.681004167	0.9546035	-0.0204358210	6.310883e-01
TREML1	-0.5472645883	2.971073e-01	7.050335e-01	0.1193580925	0.823884151	0.9995992	6.822731e-01	0.102608719	0.9546035	0.8851837924	3.718208e-02
KRTAP11-1	0.8912312077	1.650513e-01	6.287166e-01	-0.1820832673	0.770117394	0.9995992	6.433199e-01	0.161523055	0.9546035	0.3639712437	2.795712e-01
CRLF3	0.0556889231	4.445382e-01	8.021362e-01	0.0416385013	0.557514976	0.9995992	5.069287e-02	0.245759093	0.9546035	0.0578086857	2.179471e-01
C9orf40	-0.0527074822	5.581683e-01	8.319734e-01	0.0093697221	0.916987248	0.9995992	1.262125e-01	0.083511187	0.9546035	0.0581117807	2.565943e-01
PNPLA6	0.1118050891	7.962048e-01	9.305665e-01	-0.4137971837	0.346414494	0.9995992	-3.141989e-01	0.767604678	0.9572213	-0.7369862946	9.510796e-01
OSCAR	0.4146391508	3.465175e-01	7.479692e-01	0.0716416232	0.871667425	0.9995992	-1.487941e-01	0.627803693	0.9546035	0.1096338994	4.025905e-01
ALDH16A1	0.2228236826	1.962739e-02	3.455296e-01	0.2061919377	0.037428873	0.9995992	9.836429e-02	0.160773149	0.9546035	0.2094467807	2.408455e-02
SLC43A2	-0.5799799723	2.893737e-01	6.994678e-01	-0.5524055896	0.342457850	0.9995992	-4.413320e-01	0.788956710	0.9572213	-0.5496121232	8.095646e-01
CISD2	-0.0031332833	9.885428e-01	9.952791e-01	-0.2758503491	0.216578790	0.9995992	1.448548e-01	0.255223979	0.9546035	0.0319890846	4.441898e-01
CD177	-3.4209184428	2.834870e-01	6.987920e-01	-3.4756695797	0.288439593	0.9995992	-2.226058e+00	0.800749773	0.9572213	-3.0137333394	8.314778e-01
SLC43A3	-0.7715395117	1.408940e-01	6.055879e-01	-0.3907001892	0.446636486	0.9995992	-3.346208e-01	0.733149817	0.9546035	-0.4364981386	8.009845e-01
TXNDC5	-0.3559372804	2.130129e-01	6.680462e-01	-0.3954692237	0.186868022	0.9995992	8.437929e-04	0.498844048	0.9546035	-0.0614923535	5.862769e-01
NAXE	0.2275501225	2.291146e-01	6.697768e-01	0.2030958472	0.274669731	0.9995992	2.072250e-01	0.140143259	0.9546035	0.1649795497	1.772574e-01
MAPK1IP1L	-0.1013643335	4.322643e-01	7.997328e-01	-0.1442492871	0.256142434	0.9995992	5.162551e-02	0.349335819	0.9546035	-0.0082668805	5.254691e-01
RFESD	0.0409547872	6.250770e-01	8.423236e-01	-0.0017381717	0.983968460	0.9995992	5.506880e-02	0.261025338	0.9546035	-0.0616407910	7.541293e-01
UBA3	-0.2875580409	1.583160e-01	6.224838e-01	-0.1314064493	0.547056760	0.9995992	-6.449886e-02	0.622229226	0.9546035	-0.1984371875	8.046792e-01
RDH11	-2.0455937756	7.191663e-02	4.764992e-01	-0.3886730870	0.701917693	0.9995992	-2.611034e-01	0.605212402	0.9546035	-0.1368799681	5.507266e-01
LRRC20	0.0030207408	9.803258e-01	9.936282e-01	0.0562999818	0.639119104	0.9995992	1.898286e-01	0.061447768	0.9546035	0.2034366759	5.270163e-02
DCAF11	0.1134319315	3.941099e-01	7.802736e-01	0.0698865493	0.609919810	0.9995992	3.709839e-02	0.392062588	0.9546035	0.0616348986	3.299001e-01
RAB2B	-0.1059172295	6.435936e-01	8.486349e-01	0.0722031873	0.754339832	0.9995992	-2.470282e-01	0.863477873	0.9572213	0.1512467560	2.517666e-01
PDCD6IP	-0.0696813237	2.538236e-01	6.818625e-01	-0.0290486118	0.639688452	0.9995992	6.426982e-02	0.150486744	0.9546035	0.0111042315	4.292728e-01
NUDCD2	0.1257823237	8.328650e-02	5.027637e-01	0.0172132113	0.816057319	0.9995992	7.242302e-02	0.137018201	0.9546035	0.0249158218	3.789055e-01
SCFD1	-0.1726541559	4.600265e-01	8.069339e-01	-0.0294436802	0.902739656	0.9995992	-2.701057e-02	0.544827407	0.9546035	-0.1349594674	7.124423e-01
UBE2Q2	-0.1473069825	6.725972e-01	8.576613e-01	-0.0352714887	0.919075004	0.9995992	-4.879651e-01	0.917767864	0.9644203	0.0181359973	4.798875e-01
PCNP	-0.1915563085	2.016150e-01	6.638130e-01	-0.0225790959	0.884946908	0.9995992	9.881272e-03	0.474242106	0.9546035	-0.0143938335	5.364755e-01
DNAJA4	-0.0225202212	8.205996e-01	9.407844e-01	-0.0009187131	0.992474340	0.9995992	4.027075e-02	0.344778839	0.9546035	0.0794248654	2.146171e-01
DNAJC9	-0.8310326752	5.354709e-02	4.562264e-01	0.2189984985	0.590234140	0.9995992	4.325850e-01	0.157703961	0.9546035	0.1799770987	3.274878e-01
ATG4A	-0.0681563868	8.367501e-01	9.500766e-01	0.2671494151	0.393392900	0.9995992	-2.954028e-01	0.812314757	0.9572213	-0.1062910632	6.255040e-01
LZIC	-0.1322236411	2.801399e-01	6.987920e-01	-0.0074800172	0.950984314	0.9995992	1.133551e-01	0.182881101	0.9546035	0.0494414242	3.473507e-01
CFHR4	-0.3839678690	1.009635e-01	5.314990e-01	0.1424003005	0.551355577	0.9995992	-1.599266e-01	0.747291719	0.9546035	-0.2338376245	8.317968e-01
PIEZO1	-0.0876677487	8.564528e-01	9.541256e-01	-0.0020101842	0.996572082	0.9995992	-1.987669e-01	0.658204484	0.9546035	0.5822154279	1.005760e-01
PSMF1	-0.0770404489	3.190932e-01	7.225946e-01	-0.0179270792	0.817057824	0.9995992	1.332841e-01	0.043814467	0.9546035	0.1105053653	7.686266e-02
NRGN	-0.8118077275	9.188030e-02	5.171548e-01	-0.5318154563	0.288107042	0.9995992	-2.637460e-02	0.521908472	0.9546035	-0.0540460464	5.431568e-01
KRT35	-0.5986957729	5.365529e-01	8.281031e-01	-1.4816466885	0.173396842	0.9995992	-5.386179e-01	0.711568821	0.9546035	-1.0962603963	8.354438e-01
STAM	-0.1362520135	5.145593e-01	8.185856e-01	-0.4363685118	0.042605680	0.9995992	2.315725e-03	0.495608625	0.9546035	-0.0244397592	5.462179e-01
GGH	-0.3270183184	6.552213e-01	8.524806e-01	-0.1451114609	0.842031037	0.9995992	1.089008e+00	0.091026440	0.9546035	-1.3616480697	9.490699e-01
HAS1	0.0923888715	3.780213e-01	7.647336e-01	0.1226353954	0.250663946	0.9995992	-1.055632e-01	0.842078007	0.9572213	0.0423450005	3.517659e-01
COPS5	0.4751343974	1.543025e-03	1.319384e-01	0.0266380238	0.867747067	0.9995992	3.780245e-05	0.499905096	0.9546035	0.0423396849	3.962042e-01
PRG4	-0.5745701777	8.122379e-03	2.618209e-01	-0.3955202849	0.084766281	0.9995992	-1.546909e-01	0.755929664	0.9546035	-0.5191020348	9.861795e-01
TNPO1	-0.1341665284	3.154327e-01	7.197712e-01	0.0095961176	0.941544969	0.9995992	1.236810e-01	0.179901479	0.9546035	0.0937514855	2.297861e-01
USP9X	0.0596300325	4.716171e-01	8.125882e-01	-0.0975950117	0.242524346	0.9995992	1.030112e-03	0.495101314	0.9546035	0.0285159973	3.724596e-01
USP7	-0.1155819854	4.378062e-01	8.021362e-01	0.0239032544	0.870986449	0.9995992	-2.718393e-02	0.571968008	0.9546035	0.0520059539	3.639976e-01
ATP2A3	-0.8989340650	4.171744e-02	4.250863e-01	-0.1230963016	0.780768608	0.9995992	-1.061338e-01	0.596081521	0.9546035	0.0994582198	4.134336e-01
OSBP2	0.0425045680	8.426824e-01	9.500891e-01	0.4239880224	0.039524533	0.9995992	-1.847524e-01	0.802903172	0.9572213	0.1536840579	2.300402e-01
WBP2	0.0137960515	8.659839e-01	9.584204e-01	0.0358524238	0.670539523	0.9995992	8.691372e-02	0.149753663	0.9546035	-0.0584736228	7.585781e-01
PSMG2	-0.0103004699	9.327947e-01	9.813741e-01	-0.0632960647	0.599996383	0.9995992	-1.690904e-01	0.917771856	0.9644203	-0.2372195906	9.772998e-01
RPE	0.2628009291	6.781641e-02	4.639407e-01	0.1919068577	0.188258511	0.9995992	-5.806663e-02	0.653461391	0.9546035	-0.0346147911	5.899556e-01
AIDA	-0.1729513740	4.888877e-01	8.151018e-01	0.3320737033	0.177171001	0.9995992	4.055709e-02	0.436180858	0.9546035	0.2827754348	1.183313e-01
EFHD2;EFHD1	1.3928753461	1.223039e-02	3.084914e-01	-0.0674508123	0.895684748	0.9995992	1.282076e-01	0.402198656	0.9546035	0.3159264246	2.668177e-01
AP2M1	0.0140222455	8.946227e-01	9.687085e-01	-0.1319220119	0.207330640	0.9995992	4.908427e-02	0.322853231	0.9546035	-0.1132539413	8.646602e-01
CMBL	0.0147345837	9.113687e-01	9.722363e-01	0.1441566006	0.279633840	0.9995992	1.324716e-01	0.163857286	0.9546035	0.2558470230	2.094751e-02
DAZAP1	-0.0150234549	8.885957e-01	9.687085e-01	0.1161237687	0.277315776	0.9995992	1.289994e-01	0.117518401	0.9546035	0.1552286194	9.082466e-02
CNRIP1	0.0568311563	7.637919e-01	9.146931e-01	0.2375159064	0.200055037	0.9995992	-1.736186e-01	0.814874300	0.9572213	0.2466870148	8.257085e-02
DYNLL2	-0.0661786664	3.964741e-01	7.802736e-01	-0.0123994115	0.878282259	0.9995992	-9.957382e-02	0.894148693	0.9579808	-0.0893668045	8.649326e-01
OTUB1	-0.0077130813	9.206436e-01	9.747769e-01	-0.0313356963	0.685418068	0.9995992	6.479922e-02	0.205675068	0.9546035	0.0244710846	3.780320e-01
PGM2	-0.1074824559	6.429026e-01	8.486349e-01	0.0027285833	0.990643090	0.9995992	3.909593e-02	0.434503490	0.9546035	0.1431029575	2.771345e-01
PDXP	0.0884491705	9.581515e-02	5.292220e-01	0.0103535614	0.846043731	0.9995992	-4.626376e-02	0.799414570	0.9572213	0.0318910485	2.746102e-01
DCUN1D1	-0.0088522209	9.260334e-01	9.771058e-01	-0.1076658186	0.259644587	0.9995992	5.393949e-02	0.288283552	0.9546035	-0.0774261875	7.937086e-01
GMPPA	0.2411442505	4.276944e-01	7.973735e-01	-0.0246250662	0.939064520	0.9995992	-2.766288e-01	0.817948202	0.9572213	-0.3401260727	8.379682e-01
ABHD14B	-0.0149536903	8.409357e-01	9.500891e-01	-0.0227885588	0.755542877	0.9995992	-1.983053e-02	0.601516770	0.9546035	0.0215700219	3.788860e-01
CPB2	-0.3787875109	6.464010e-04	1.319384e-01	-0.1054584572	0.365797810	0.9995992	-1.608861e-01	0.922389440	0.9644203	-0.2643983113	9.899623e-01
USP47	0.1713290835	2.217388e-01	6.697768e-01	0.0015983343	0.991211374	0.9995992	-1.972351e-02	0.554614241	0.9546035	0.0091721686	4.742333e-01
CNDP1	-0.5200232662	2.938801e-02	3.864738e-01	-0.1009414462	0.674278366	0.9995992	-1.259069e-01	0.698649483	0.9546035	-0.4157205128	9.508931e-01
CNDP2	0.0548113029	8.012665e-01	9.331005e-01	0.2332928476	0.287442245	0.9995992	1.154358e-01	0.297951387	0.9546035	0.2507053273	1.241292e-01
RILP	-0.0268064095	8.600153e-01	9.551688e-01	-0.0560465913	0.726694073	0.9995992	5.679885e-02	0.356554941	0.9546035	0.1101261867	2.607186e-01
IPO9	0.0416232804	6.418937e-01	8.486349e-01	-0.1178498423	0.199765567	0.9995992	5.424997e-02	0.275952358	0.9546035	0.0423834440	3.297903e-01
PGLYRP2	-0.0596669100	5.011356e-01	8.151018e-01	-0.0339235434	0.704664626	0.9995992	-1.983623e-02	0.586050059	0.9546035	-0.1238673480	9.225012e-01
ERMAP	-0.4422359101	1.842769e-01	6.368870e-01	-0.0406573383	0.902581910	0.9995992	8.127062e-02	0.404619632	0.9546035	0.3258474270	1.635639e-01
NUDCD1	-0.0914349597	5.841716e-01	8.403054e-01	-0.2482500568	0.140676759	0.9995992	-1.710213e-01	0.846484959	0.9572213	-0.2205412405	9.034432e-01
PEBP4	-0.1374145609	6.933848e-01	8.660792e-01	0.3207543875	0.338236926	0.9995992	-4.535732e-01	0.899674327	0.9579808	-0.2382672363	7.353435e-01
TBCB	0.4226814082	1.815039e-01	6.347267e-01	0.2371745589	0.421278419	0.9995992	2.572331e-01	0.217980253	0.9546035	0.1708664037	2.677675e-01
PSMB7	0.1791375973	2.021003e-02	3.455296e-01	-0.0274167654	0.729947740	0.9995992	-6.553047e-02	0.793448626	0.9572213	0.0156254142	4.204430e-01
PSMD1	0.0666191718	4.094949e-01	7.883110e-01	-0.1747839109	0.034129836	0.9995992	5.612050e-02	0.241514670	0.9546035	-0.0149622051	5.662721e-01
PFDN5	-0.0299024052	9.019102e-01	9.710488e-01	0.2146133762	0.362807661	0.9995992	-7.359086e-02	0.615768144	0.9546035	0.0343170433	4.447115e-01
PARK7	0.1483946860	4.863285e-02	4.562264e-01	0.0072515322	0.926578304	0.9995992	6.511460e-02	0.201585526	0.9546035	0.0846892060	1.422657e-01
TSNAX	0.0029344227	9.762425e-01	9.928688e-01	0.1138348470	0.249365397	0.9995992	-7.827144e-02	0.785221759	0.9572213	0.0097930158	4.618438e-01
COPS8	-0.0056476233	9.527226e-01	9.905132e-01	0.1460236350	0.117625735	0.9995992	-1.039626e-02	0.543003288	0.9546035	0.1488039409	5.693740e-02
NAP1L4	-0.0337105923	7.777985e-01	9.239777e-01	0.1064905672	0.368965590	0.9995992	7.955369e-02	0.253308296	0.9546035	0.1197801570	1.482626e-01
MGST2	-1.0493389218	1.274595e-01	5.775504e-01	0.3305673760	0.601562103	0.9995992	2.770119e-01	0.343983964	0.9546035	0.5007407389	2.120391e-01
SLC29A1	-0.3717684673	5.699150e-01	8.344560e-01	0.1828225244	0.781479571	0.9995992	7.861719e-01	0.123002613	0.9546035	1.3827770068	1.793531e-02
CPNE1	0.2693319173	2.335145e-01	6.697768e-01	-0.0214144444	0.924519318	0.9995992	-1.089884e-01	0.680260881	0.9546035	0.2002913804	1.856611e-01
CCT7	-0.0252646133	6.705576e-01	8.576613e-01	0.0153337964	0.802288585	0.9995992	-2.513310e-02	0.662340499	0.9546035	0.0081275447	4.480520e-01
RARRES2	-0.0853051278	3.609398e-01	7.553917e-01	0.0266235119	0.769832325	0.9995992	-1.656104e-01	0.958717613	0.9769002	-0.0532731955	7.256107e-01
WDR77	-0.1212777811	2.848892e-01	6.987920e-01	-0.1239847872	0.284279641	0.9995992	4.495001e-02	0.350153574	0.9546035	-0.1080718911	8.247080e-01
TXNDC17	0.1091510493	2.063106e-01	6.658594e-01	-0.0347781141	0.692029314	0.9995992	-1.615865e-01	0.966737128	0.9774190	0.0041020422	4.809075e-01
CPPED1	0.6246109750	5.095043e-02	4.562264e-01	-0.0007167651	0.998231120	0.9995992	5.458073e-01	0.045215055	0.9546035	0.2084215904	2.664396e-01
MIEN1	-0.0678440290	6.150857e-01	8.419469e-01	0.2057573653	0.128091073	0.9995992	4.375634e-02	0.374852505	0.9546035	0.1943599848	8.855914e-02
ERP44	0.0692561831	6.478111e-01	8.489971e-01	-0.0854032118	0.570973288	0.9995992	5.569898e-02	0.359776851	0.9546035	0.2337044345	5.317859e-02
LXN	-0.2095718957	2.051725e-01	6.658594e-01	0.0539856069	0.749791951	0.9995992	5.083610e-02	0.381327141	0.9546035	0.1502587374	1.822336e-01
UBAC1	0.1688288061	8.421930e-02	5.027637e-01	0.0111306609	0.909891899	0.9995992	7.921909e-02	0.213960361	0.9546035	0.1312011787	9.849643e-02
PSMG3	-0.1580996148	1.075992e-01	5.408542e-01	0.0306413740	0.757894303	0.9995992	2.568028e-01	0.004364245	0.7369340	0.1260751982	1.140669e-01
COPS4	0.2946390538	3.479924e-05	2.056635e-02	0.1013494221	0.187803206	0.9995992	3.709144e-02	0.320051697	0.9546035	0.1389065147	4.164447e-02
MRI1	-0.2639092791	6.344089e-02	4.597775e-01	0.0376626459	0.796837936	0.9995992	-1.428875e-02	0.540821648	0.9546035	-0.0871747090	7.220832e-01
SELENOO	-0.1221021969	4.451154e-01	8.021362e-01	-0.0907645827	0.566075076	0.9995992	-8.303356e-02	0.694541274	0.9546035	-0.0678926272	6.629700e-01
ACAT2	0.0339237108	6.482623e-01	8.489971e-01	0.0394020522	0.602485412	0.9995992	-7.393332e-02	0.844449526	0.9572213	0.0473651755	2.674452e-01
COLEC11	0.2254370147	3.822277e-01	7.683557e-01	-0.3185190905	0.225272125	0.9995992	9.926857e-02	0.354238482	0.9546035	-0.2622604404	8.330334e-01
CFHR5	0.0087618443	9.503969e-01	9.905132e-01	0.0224817757	0.870214133	0.9995992	-3.043649e-03	0.508427082	0.9546035	-0.0829568334	7.133116e-01
CHMP4A	-0.3289601492	3.449646e-02	4.027342e-01	-0.3394903526	0.033362025	0.9995992	-4.684962e-02	0.613717793	0.9546035	-0.3163004870	9.771130e-01
SRXN1	0.0713901583	4.634336e-01	8.069339e-01	-0.0575620077	0.549011102	0.9995992	-7.482055e-02	0.774565862	0.9572213	-0.0597289888	7.266589e-01
UBE2O	0.0516941985	5.990733e-01	8.419469e-01	0.1063352312	0.299532899	0.9995992	4.781645e-02	0.316652076	0.9546035	0.0917756984	1.954271e-01
C20orf27	-1.2157877348	2.575678e-01	6.887899e-01	1.0546029440	0.293142191	0.9995992	1.085673e+00	0.161476156	0.9546035	0.9531253062	1.561219e-01
PITHD1	-0.0925519876	2.486265e-01	6.760373e-01	-0.0525169226	0.527312007	0.9995992	9.410071e-02	0.124369007	0.9546035	0.0525110533	2.716541e-01
NIF3L1	-0.0053967891	9.688180e-01	9.923248e-01	0.0721948367	0.606806640	0.9995992	-7.508282e-02	0.703505659	0.9546035	0.0784912763	2.945981e-01
UBA5	-0.4900447328	3.981749e-02	4.202168e-01	0.2600794185	0.268491445	0.9995992	1.954966e-01	0.208145017	0.9546035	0.2196790238	1.763670e-01
LHPP	-0.1206587437	4.983493e-01	8.151018e-01	-0.0568040329	0.752918277	0.9995992	-2.151217e-01	0.881931804	0.9579808	-0.1426368677	7.827238e-01
PCBD2	-0.0516911196	7.097526e-01	8.784582e-01	-0.1791924865	0.208266225	0.9995992	5.562288e-02	0.346471817	0.9546035	-0.0621269137	6.550306e-01
HDHD2	-0.2474002376	4.836360e-01	8.129736e-01	-0.3960673381	0.296220303	0.9995992	-4.240157e-01	0.883301659	0.9579808	-0.4486902388	8.636365e-01
RAB1B	-0.1386065312	2.839014e-01	6.987920e-01	0.0391837787	0.757450990	0.9995992	2.084257e-01	0.054608173	0.9546035	0.1388857438	1.428401e-01
SH3BGRL3	-0.0006539666	9.964030e-01	9.990571e-01	0.0301396731	0.837287640	0.9995992	-9.952117e-02	0.752523664	0.9546035	-0.0498768069	6.283147e-01
PPIL3	-1.0617764212	2.484641e-01	6.760373e-01	2.6885502154	0.015212235	0.9995992	1.934658e+00	0.028889420	0.9546035	2.6411569744	7.280907e-03
BOLA2	-0.0927117106	2.352318e-01	6.697768e-01	0.0554646741	0.484295632	0.9995992	7.492349e-02	0.172189571	0.9546035	0.0659702389	2.012404e-01
TMX1	-0.1428710853	5.476908e-01	8.281031e-01	0.0760586773	0.750676365	0.9995992	1.570191e-01	0.252593940	0.9546035	0.3178036742	1.019032e-01
CHMP4B	-0.1658594207	3.198931e-01	7.225946e-01	0.0181836498	0.913033483	0.9995992	-2.351951e-01	0.915276879	0.9644203	-0.1090618369	7.503362e-01
FN3K	-0.0176969983	8.924530e-01	9.687085e-01	-0.1646769169	0.204781241	0.9995992	1.085969e-01	0.207627928	0.9546035	0.0519726578	3.548766e-01
WNK1	0.1959731183	4.497178e-02	4.531788e-01	0.1542839061	0.115574351	0.9995992	1.184681e-01	0.115275601	0.9546035	0.2045068223	1.795549e-02
RNPEP	0.3279953190	2.147844e-02	3.455296e-01	0.0534040963	0.705509007	0.9995992	-1.914471e-01	0.901247571	0.9579808	0.0092896872	4.735027e-01
TUBB1	-0.5385122049	3.805289e-02	4.126470e-01	0.0455375511	0.858334515	0.9995992	-1.132514e-01	0.666757345	0.9546035	-0.1625524147	7.418048e-01
GLIPR2	-0.0075165723	9.560748e-01	9.907192e-01	0.1436325627	0.285368280	0.9995992	5.819978e-02	0.338050653	0.9546035	0.2164962937	4.872939e-02
EHD1	0.0173832218	8.819230e-01	9.661103e-01	0.0403894865	0.723740857	0.9995992	2.416309e-02	0.418261702	0.9546035	0.0111146774	4.611274e-01
AAMDC	-0.5500934070	4.772416e-03	2.131357e-01	-0.3232331602	0.095562473	0.9995992	-2.647938e-01	0.913690994	0.9644203	-0.3066204712	9.419362e-01
METTL7A	-0.6613963942	6.700096e-02	4.639407e-01	-0.1630648938	0.659097745	0.9995992	3.168411e-02	0.465189265	0.9546035	0.3744084852	1.617786e-01
COPS7B	0.1009770332	2.991177e-01	7.059294e-01	0.0070408799	0.945238590	0.9995992	3.333842e-02	0.369441893	0.9546035	0.0937286932	1.940984e-01
FN3KRP	0.0760003066	4.444968e-01	8.021362e-01	-0.0955008977	0.336595130	0.9995992	-9.198525e-03	0.537044042	0.9546035	0.0013452374	4.947725e-01
PPCS	-0.0483900281	5.938080e-01	8.419469e-01	-0.1267808944	0.156716195	0.9995992	6.934510e-03	0.470275210	0.9546035	-0.0328115865	6.425933e-01
PARVB	-0.5679520991	1.898195e-03	1.319384e-01	-0.1586315369	0.412423513	0.9995992	-1.835697e-01	0.835989102	0.9572213	-0.1367470161	7.447529e-01
GLOD4	0.0043255762	9.626177e-01	9.920806e-01	0.0047806712	0.959778308	0.9995992	8.486661e-02	0.182913132	0.9546035	0.0786925029	2.102029e-01
APMAP	-0.1207898038	6.094907e-01	8.419469e-01	-0.0057221272	0.980680033	0.9995992	4.311113e-02	0.429389794	0.9546035	0.0509499735	4.166419e-01
ABCB6	-0.6051237407	3.084680e-01	7.191501e-01	0.1652661940	0.775224756	0.9995992	-1.006035e-01	0.567843603	0.9546035	0.6739756817	1.263145e-01
SLC40A1	-0.6868281983	2.712789e-01	6.940168e-01	0.3473729285	0.563585935	0.9995992	-4.644441e-02	0.529443363	0.9546035	0.5923189292	1.596835e-01
RAB18	0.7133399155	5.983539e-02	4.562264e-01	-0.1069440507	0.782341750	0.9995992	-4.813907e-01	0.898104232	0.9579808	0.4095402782	1.504247e-01
VTA1	0.9271422605	7.750951e-02	4.905904e-01	-0.2507868015	0.629134885	0.9995992	4.465577e-01	0.200304603	0.9546035	-0.4813521955	8.149626e-01
RIC8A	-0.3001846615	2.976410e-01	7.050335e-01	-0.1450744307	0.616882959	0.9995992	-5.313342e-01	0.964814499	0.9774190	-0.3172872208	8.582316e-01
SPINK5	0.6799055060	2.032318e-01	6.646753e-01	0.0314545466	0.949728177	0.9995992	-5.107294e-01	0.827194085	0.9572213	-0.9130111008	9.313817e-01
CRTAC1	-0.4225001778	2.598365e-01	6.900429e-01	-0.3322774313	0.384130204	0.9995992	2.524679e-02	0.473649196	0.9546035	-0.3778498488	8.357533e-01
RTN4	0.0238928018	9.397999e-01	9.832514e-01	0.1874517122	0.554869965	0.9995992	1.353200e-01	0.335851920	0.9546035	0.5223320668	5.095218e-02
XPNPEP1	0.4016266754	2.368585e-01	6.697768e-01	0.0744692875	0.830280126	0.9995992	3.509792e-01	0.160373879	0.9546035	0.5077236028	5.121237e-02

**NANS**	0.0660175437	5.713731e-01	8.344560e-01	-0.0290697306	0.801040211	0.9995992	3.812054e-02	0.375046012	0.9546035	0.0705967022	2.674514e-01
**SH3GLB2**	-0.0210851067	8.637461e-01	9.568396e-01	-0.0140794174	0.909943825	0.9995992	2.663007e-02	0.416215368	0.9546035	-0.0276188018	5.868053e-01
**HEBP1**	0.0905231824	4.305423e-01	7.991327e-01	-0.0089031112	0.938677308	0.9995992	-1.695663e-01	0.924700484	0.9644203	-0.1186532930	8.637958e-01
**RAB6B**	-0.2002830954	2.291806e-01	6.697768e-01	-0.0669702723	0.691130810	0.9995992	6.630474e-02	0.347222868	0.9546035	-0.0844788773	6.858611e-01
**PHPT1**	-0.1256109515	4.269072e-01	7.973735e-01	-0.0751610530	0.635848218	0.9995992	-9.319132e-02	0.718637739	0.9546035	-0.0788579306	6.901038e-01
**FAM114A2**	-0.0930216082	2.626441e-01	6.900429e-01	0.0277902396	0.738569937	0.9995992	-4.319944e-02	0.694894774	0.9546035	-0.0361276200	6.711840e-01
**LANCL2**	0.1331098937	2.057064e-01	6.658594e-01	-0.0662406953	0.539167891	0.9995992	-5.982488e-02	0.707942796	0.9546035	0.0168898866	4.394476e-01
**KRT82**	0.0577554828	9.149498e-01	9.738869e-01	0.5244695328	0.334117091	0.9995992	-1.419748e+00	0.994120415	0.9949622	-0.6283928712	8.742196e-01
**ATG3**	0.2189331750	1.528176e-01	6.224838e-01	-0.0038027965	0.980501436	0.9995992	6.992984e-02	0.328638948	0.9546035	-0.0043350383	5.112401e-01
**SACM1L**	-0.5743426696	1.805238e-01	6.347267e-01	-0.1169243225	0.785465380	0.9995992	7.082932e-02	0.434564813	0.9546035	0.3380933286	2.092580e-01
**OLA1**	0.0908003979	1.795709e-01	6.347267e-01	-0.0555236647	0.416519013	0.9995992	3.016179e-03	0.482899192	0.9546035	-0.0286705143	6.588450e-01
**CYRIB**	0.0509491767	8.142475e-01	9.398834e-01	0.0364683247	0.866203830	0.9995992	-1.671300e-01	0.776024636	0.9572213	0.1221574787	2.906691e-01
**PNPO**	0.1204316759	6.423522e-01	8.486349e-01	-0.1408385635	0.588142543	0.9995992	-5.545648e-01	0.981837539	0.9860085	-0.3431436558	9.056355e-01
**CZIB**	-0.0561251214	5.822804e-01	8.397024e-01	0.0421761897	0.682391659	0.9995992	1.251552e-01	0.113343682	0.9546035	0.0890699124	1.889777e-01
**THG1L**	0.0847930501	4.339751e-01	8.014978e-01	-0.1240158808	0.251994832	0.9995992	-3.635795e-02	0.629154107	0.9546035	0.0455365929	3.384749e-01
**ADPRS**	0.0742782825	7.857626e-01	9.267668e-01	-0.0210144871	0.937405673	0.9995992	-3.790840e-01	0.915430633	0.9644203	-0.6100737996	9.714248e-01
**DPP3**	0.0372336875	6.409320e-01	8.486349e-01	0.0843691730	0.281201051	0.9995992	-2.017751e-02	0.601478194	0.9546035	0.1559443399	2.073595e-02
**AHSP**	-0.0093737713	9.586378e-01	9.912589e-01	0.2073588084	0.258281224	0.9995992	-8.288357e-02	0.673928207	0.9546035	0.0449517584	4.066001e-01
**C1RL**	-0.1826943738	4.808694e-02	4.562264e-01	-0.0369549417	0.703641968	0.9995992	-7.173092e-02	0.773224811	0.9572213	-0.1859748522	9.728990e-01
**KCMF1**	-0.0056591153	9.659808e-01	9.923248e-01	-0.1867938382	0.165490382	0.9995992	-2.163354e-01	0.947263778	0.9707604	-0.1059554746	7.705606e-01
**VAPA**	-0.4763842713	2.488525e-01	6.760373e-01	-0.1914904049	0.646097883	0.9995992	1.285461e-01	0.378294291	0.9546035	0.3738428572	1.817842e-01
**ABRACL**	-0.1446034701	5.632853e-01	8.323189e-01	-0.0405711395	0.872935444	0.9995992	-1.641316e-01	0.740838438	0.9546035	0.1942169151	2.194817e-01
**ANKFY1**	0.1169283789	1.110687e-01	5.470133e-01	0.0049473885	0.948546450	0.9995992	3.478056e-02	0.323781775	0.9546035	0.0657989517	2.022609e-01
**GMPR2**	0.0290591051	7.373488e-01	8.976831e-01	-0.0195830633	0.821004871	0.9995992	-6.757054e-03	0.530397108	0.9546035	-0.0302679103	6.357902e-01
**GRHPR**	-0.1861406854	1.174934e-01	5.618203e-01	0.1104740029	0.349121699	0.9995992	1.546361e-01	0.096179282	0.9546035	0.0593986638	3.085410e-01
**PEF1**	0.0876990414	5.713236e-01	8.344560e-01	-0.0995858969	0.513649539	0.9995992	-1.935580e-02	0.549637452	0.9546035	-0.0151753897	5.387990e-01
**BIN2**	-0.5169687335	6.379315e-02	4.597775e-01	0.2583754449	0.339722025	0.9995992	-3.519487e-01	0.902808419	0.9587777	0.0635571741	4.046346e-01
**COPS7A**	0.2691802642	1.675145e-01	6.287166e-01	0.1775400228	0.371451138	0.9995992	1.356978e-01	0.246957378	0.9546035	0.3325527224	5.253061e-02
**SEC14L4**	0.0981794593	4.622048e-01	8.069339e-01	0.1231770748	0.362050690	0.9995992	-1.644980e-01	0.887208987	0.9579808	-0.0979779270	7.741527e-01
**FETUB**	-0.1959956113	6.114133e-02	4.562264e-01	-0.0122805662	0.908030169	0.9995992	4.581716e-02	0.338752200	0.9546035	-0.0809034366	7.897234e-01
**PCYOX1**	-0.5344763874	1.359092e-03	1.319384e-01	-0.1813814340	0.283004097	0.9995992	4.320068e-02	0.403130090	0.9546035	-0.1154809469	7.460390e-01
**PFDN2**	-0.2469131020	5.409317e-01	8.281031e-01	0.8980742830	0.031978780	0.9995992	3.073795e-01	0.229430873	0.9546035	0.5908185589	8.387749e-02
**NRBP1**	0.0981572548	7.139066e-01	8.808326e-01	-0.1546954449	0.566406871	0.9995992	-1.490097e-01	0.706527028	0.9546035	-0.0320322383	5.446311e-01
**ENOPH1**	0.0679814833	7.716402e-01	9.203620e-01	0.1935070746	0.420361885	0.9995992	-2.059282e-01	0.812779770	0.9572213	0.0101683242	4.835871e-01
**XPO7**	0.0887648466	3.587479e-01	7.553917e-01	-0.0577171313	0.560986440	0.9995992	5.847491e-02	0.277428720	0.9546035	-0.0467779510	6.782003e-01
**DBNL**	-0.0960530585	2.294836e-01	6.697768e-01	0.0941796638	0.237876531	0.9995992	3.541093e-04	0.498256830	0.9546035	0.0743921432	1.842654e-01
**SERPINA10**	-0.0992856085	3.398091e-01	7.410597e-01	-0.0020995508	0.983971062	0.9995992	6.632054e-02	0.268851153	0.9546035	-0.0128122183	5.510304e-01
**NUDT5**	0.1799398189	1.746692e-02	3.225921e-01	0.0011911722	0.987754672	0.9995992	1.070833e-01	0.087465395	0.9546035	0.0437161991	2.896547e-01
**AGO2**	0.0282837571	6.177161e-01	8.419469e-01	-0.0313541375	0.572579990	0.9995992	-7.446478e-02	0.900758122	0.9579808	0.0204846708	3.611583e-01
**RAB21**	0.2079797130	3.784777e-01	7.647336e-01	-0.3744117062	0.124517384	0.9995992	9.709272e-02	0.344669441	0.9546035	0.0913710509	3.489061e-01
**PSME2**	0.0126854729	8.605926e-01	9.551688e-01	-0.0079393650	0.913623611	0.9995992	-7.009190e-02	0.830691659	0.9572213	-0.0009480472	5.050638e-01
**DNPEP**	0.1145745774	1.360073e-01	5.932126e-01	0.0005980474	0.994061880	0.9995992	3.024714e-02	0.350207251	0.9546035	0.0381881744	3.213465e-01
**MCTS1**	-0.0132810419	9.037912e-01	9.710488e-01	-0.0629546995	0.565270716	0.9995992	-7.964799e-02	0.759495119	0.9559168	-0.0002639211	5.009672e-01
**ASAP1**	-0.1897690867	8.844938e-02	5.150107e-01	-0.0283753919	0.805608166	0.9995992	8.654373e-02	0.194911576	0.9546035	0.0728828083	2.637903e-01
**TBC1D24**	0.6712757218	1.843098e-04	7.261804e-02	0.2730102011	0.141512791	0.9995992	1.741508e-01	0.182424614	0.9546035	0.2706276744	7.671271e-02
**CORO1C**	-0.3706766324	2.727242e-02	3.705288e-01	-0.2931393362	0.097225412	0.9995992	-3.112706e-01	0.967742829	0.9774190	-0.4229235227	9.899974e-01
**PSMD13**	0.0287033934	7.592586e-01	9.116018e-01	-0.1235585824	0.185267125	0.9995992	-6.744335e-02	0.761290814	0.9561271	0.0568386181	2.823296e-01
**COPS3**	0.1126021581	3.781777e-02	4.126470e-01	0.0260547710	0.637971807	0.9995992	-5.107668e-02	0.820249251	0.9572213	0.0115373731	4.171228e-01
**MINPP1**	0.0980387104	4.396397e-01	8.021362e-01	-0.1404230846	0.255047580	0.9995992	-4.711932e-02	0.641464136	0.9546035	-0.1539395155	9.024083e-01
**NSFL1C**	0.0201855208	7.607048e-01	9.119200e-01	-0.0402429904	0.542846547	0.9995992	6.882347e-03	0.459725312	0.9546035	-0.0311667796	6.820731e-01
**PPP6R1**	-0.1240677514	6.592447e-02	4.639407e-01	0.0538054224	0.424244821	0.9995992	-1.100401e-02	0.562072368	0.9546035	-0.0115929980	5.684488e-01
**PA2G4**	-0.3660083935	8.409669e-02	5.027637e-01	-0.0938254746	0.664469158	0.9995992	-1.049379e-01	0.684719423	0.9546035	-0.2016468326	8.228347e-01
**RUVBL2**	0.0504046484	6.960723e-01	8.669731e-01	-0.1157667341	0.356292251	0.9995992	2.102375e-02	0.437154349	0.9546035	0.0168531107	4.473033e-01
**RUVBL1**	0.1250467767	3.861135e-01	7.727636e-01	-0.1381717064	0.326703849	0.9995992	6.742599e-02	0.325501987	0.9546035	-0.0055913662	5.157890e-01
**ST3GAL6**	-0.0889503492	6.898427e-01	8.660792e-01	-0.0272159725	0.901928034	0.9995992	1.915171e-01	0.197165637	0.9546035	0.1203626610	2.965343e-01
**CARHSP1**	0.0592995847	5.040772e-01	8.151018e-01	-0.0120641861	0.893779156	0.9995992	4.524764e-02	0.303461693	0.9546035	0.0869282817	1.760188e-01
**PRG3**	-0.5057513078	9.340806e-02	5.196358e-01	0.0451277090	0.883225920	0.9995992	-1.683614e-01	0.710435241	0.9546035	-0.2311714505	7.605532e-01
**SUGT1**	0.1986329876	1.649197e-01	6.287166e-01	0.0855840562	0.557274843	0.9995992	4.553502e-02	0.373027307	0.9546035	0.1183035198	2.131262e-01
**DERA**	0.3367136050	1.226658e-02	3.084914e-01	0.2607781553	0.057838014	0.9995992	-1.487732e-01	0.855498175	0.9572213	0.2748152161	2.410688e-02
**REXO2**	-0.0173381631	8.685865e-01	9.586081e-01	-0.0910676262	0.375748218	0.9995992	-4.763413e-02	0.672063073	0.9546035	-0.1229781366	8.883569e-01
**UFC1**	-0.0899763877	2.035638e-01	6.646753e-01	-0.0139119793	0.848076520	0.9995992	-2.592821e-02	0.637991686	0.9546035	0.0332139750	3.248539e-01
**FBXO7**	0.0828513203	3.076460e-01	7.186513e-01	0.0128780874	0.876914492	0.9995992	1.321000e-01	0.052327699	0.9546035	0.1254062276	6.474413e-02
**TLN1**	-0.2619028944	6.175654e-02	4.562264e-01	-0.0145904980	0.918090825	0.9995992	-9.876067e-02	0.754122798	0.9546035	-0.0188528995	5.523440e-01
**USP15**	0.0813538290	2.491410e-01	6.760373e-01	-0.0516094816	0.460688212	0.9995992	-2.650101e-02	0.642768703	0.9546035	-0.0291637064	6.569214e-01
**PPME1**	0.1470975937	8.895879e-02	5.154377e-01	-0.0201863938	0.820765727	0.9995992	-5.327679e-02	0.723390514	0.9546035	0.0149488560	4.362653e-01
**UCHL5**	-0.0546063570	7.123080e-01	8.803438e-01	-0.1509179802	0.316611010	0.9995992	2.773863e-02	0.427433868	0.9546035	-0.0469514397	6.206706e-01
**CD2AP**	0.1571663599	5.484893e-01	8.281031e-01	0.1525580165	0.562114698	0.9995992	3.635794e-01	0.085687847	0.9546035	0.4998894320	2.883699e-02
**GMPPB**	0.4856988872	5.475999e-02	4.562264e-01	0.1226121884	0.612749667	0.9995992	-3.566270e-01	0.922114615	0.9644203	0.1546005751	2.587247e-01
**LYVE1**	-0.2238678087	2.493674e-01	6.760373e-01	-0.0531718772	0.783325181	0.9995992	-1.867991e-02	0.537150650	0.9546035	-0.0984185710	6.850864e-01
**HEBP2**	0.0820619269	5.365707e-01	8.281031e-01	0.2338483268	0.082822361	0.9995992	1.260208e-01	0.171357034	0.9546035	0.1974931565	7.916985e-02
**PSAT1**	-0.0639556696	9.340490e-01	9.813741e-01	-0.5246513876	0.519891706	0.9995992	-5.089829e-02	0.525736237	0.9546035	-1.0298918093	8.830616e-01
**F11R**	-0.5610191526	2.411471e-01	6.725635e-01	0.1455692620	0.752818151	0.9995992	2.623892e-02	0.478176725	0.9546035	0.5376963145	1.192782e-01
**CLIC4**	0.1910087062	3.565331e-01	7.538857e-01	-0.3650371771	0.088679830	0.9995992	-3.178874e-02	0.560071096	0.9546035	-0.3973480379	9.622508e-01
**FCGBP**	-0.2085229265	1.856326e-01	6.397019e-01	-0.0781281670	0.622443548	0.9995992	-1.061910e-02	0.526760417	0.9546035	-0.1327886734	7.871635e-01

SHRM	SRF	SRF	SRF	DiabMellitus	DiabMellitus	DiabMellitus	AHtn	AHtn	AHtn	Activesmoking	Activesmoking
Adj. p-val. (1-sided test, SHRM > no SHRM, Benjamini-H. cor.)	AVG.Log.Ratio	p-val. (1-sided test, SRF > no SRF)	Adj. p-val. (1-sided test, SRF > no SRF, Benjamini-H. cor.)	AVG.Log.Ratio	p-val. (2-sided test)	Adj. p-val. (2-sided test, Benjamini-H. cor.)	AVG.Log.Ratio	p-val. (2-sided test)	Adj. p-val. (2-sided test, Benjamini-H. cor.)	AVG.Log.Ratio	p-val. (2-sided test)
0.93650941	-2.746462e-01	8.107420e-01	0.9997779456	-0.0782252642	0.8295796153	0.96048889	-3.812821e-02	0.890930057	0.9982883	-2.730476e-01	0.4758576943
0.79213004	5.281618e-02	3.826782e-01	0.9997779456	-0.2222812001	0.4049598794	0.89501894	-1.939806e-01	0.379600663	0.9982883	-2.176575e-01	0.1964054368
0.80653217	9.936077e-02	4.304453e-01	0.9997779456	-0.2225912773	0.6341442342	0.93852299	-1.305824e-01	0.790470177	0.9982883	1.248891e-01	0.8368196200
0.88581308	5.755813e-01	1.167496e-01	0.9997779456	-0.4283731095	0.3354082165	0.85880449	7.819189e-02	0.844199898	0.9982883	3.956651e-02	0.9475270859
0.97050390	-9.388206e-01	8.416727e-01	0.9997779456	-0.1014691118	0.8747824429	0.96947881	-1.128130e+00	0.157968375	0.9982883	-7.254915e-01	0.1609723076
0.92876201	3.231207e-01	2.406992e-01	0.9997779456	0.2012224514	0.7317523720	0.94712279	-2.165309e-01	0.641049805	0.9982883	-2.892426e-01	0.4280925202
0.98996967	-1.210484e+00	8.633920e-01	0.9997779456	-1.7398054957	0.0148339186	0.49365173	-2.275224e-01	0.716554013	0.9982883	1.312001e-01	0.8753238818
0.87292023	-4.865342e-01	8.045893e-01	0.9997779456	0.1339702816	0.7223490934	0.94712279	-4.283486e-01	0.372127396	0.9982883	-5.213268e-01	0.2066957972
0.84824569	-2.188262e-01	8.498449e-01	0.9997779456	-0.1371764141	0.7149671601	0.94529215	-1.478803e-01	0.490449908	0.9982883	-3.699385e-02	0.8769040257
0.97273515	-2.929371e-01	9.146321e-01	0.9997779456	0.2208342086	0.5691002581	0.92122660	4.306976e-01	0.025271932	0.9982883	-5.671191e-01	0.0101237958
0.80653217	-3.965359e-01	9.910959e-01	0.9997779456	-0.2461069605	0.2907267476	0.84915316	9.559354e-02	0.568084123	0.9982883	-1.389324e-01	0.6606857836
0.80820047	-3.076418e-01	8.945090e-01	0.9997779456	0.3579306946	0.0537991465	0.83664873	1.633878e-01	0.414664209	0.9982883	-3.951610e-02	0.8208265518
0.97273515	-2.756053e-01	8.924706e-01	0.9997779456	0.1905769437	0.4566631616	0.90612020	1.237003e-01	0.548479451	0.9982883	-7.561464e-02	0.7369179765
0.90408005	-4.618923e-01	9.661048e-01	0.9997779456	-0.1546498991	0.5099517552	0.90612020	-3.134715e-01	0.171483844	0.9982883	-6.139976e-01	0.0039025729
0.97273515	-1.338820e+00	9.338369e-01	0.9997779456	-0.6790443525	0.2010239771	0.83807199	-4.685507e-01	0.534646902	0.9982883	-2.461247e-01	0.6128628701
0.96804147	-6.988670e-01	8.215756e-01	0.9997779456	0.7011833304	0.2352675808	0.84413548	6.552107e-01	0.222914700	0.9982883	-1.737321e-01	0.7243922107
0.80820047	1.801438e-01	3.295673e-01	0.9997779456	-0.3821013084	0.4875441508	0.90612020	7.057681e-01	0.066855891	0.9982883	-4.352875e-01	0.3351215956
0.95558280	-2.853912e-01	7.948580e-01	0.9997779456	-0.5882744090	0.0193687291	0.58702148	-1.713136e-01	0.533264189	0.9982883	-2.212476e-01	0.3614819677
0.77869908	2.207594e-01	2.682067e-01	0.9997779456	0.0606347006	0.8640919913	0.96707132	-3.845598e-01	0.516900853	0.9982883	-6.572352e-01	0.0606777812
0.87292023	8.650954e-02	3.944281e-01	0.9997779456	-0.1517672713	0.7067743177	0.94482995	-4.165456e-01	0.177175741	0.9982883	-4.618428e-01	0.1928300068
0.98226159	-5.015327e-01	8.758036e-01	0.9997779456	0.0732490109	0.8129955363	0.95972154	4.861149e-02	0.891252833	0.9982883	-7.233766e-01	0.0502558618
0.79578964	-1.250177e-01	7.118264e-01	0.9997779456	-0.4626279721	0.0264756276	0.64586404	-3.912779e-01	0.115157283	0.9982883	-3.292068e-01	0.2450727451
0.85414112	-8.596031e-01	9.558162e-01	0.9997779456	-0.8166766860	0.0239685678	0.64388289	-5.491604e-01	0.195596041	0.9982883	2.353675e-01	0.7477261319
0.94675587	1.409140e-01	2.880602e-01	0.9997779456	0.5965007208	0.0785894789	0.83807199	4.038419e-01	0.104660627	0.9982883	5.380044e-02	0.8938823534
0.97273515	-1.400283e+00	8.295448e-01	0.9997779456	-0.9393400703	0.2238353656	0.83807199	-1.182468e+00	0.345659976	0.9982883	-1.408063e+00	0.0943657437
0.97273515	-2.692168e-01	8.050248e-01	0.9997779456	0.2512410299	0.5010069315	0.90612020	-2.290915e-01	0.423344286	0.9982883	1.955620e-01	0.5877330571
0.96804147	3.999099e-02	4.577158e-01	0.9997779456	-1.3094736911	0.0003999359	0.09450935	-1.886389e-01	0.597422839	0.9982883	4.157088e-01	0.4877086770
0.77869908	-8.204628e-01	9.457244e-01	0.9997779456	-0.5317950844	0.1544156364	0.83807199	4.689505e-01	0.187767892	0.9982883	-6.473538e-01	0.0916759388
0.98998602	-5.376863e-02	5.535707e-01	0.9997779456	0.5836649788	0.3315548497	0.85754449	-5.055931e-02	0.888902126	0.9982883	2.407295e-01	0.5309887896
0.97273515	-5.725066e-01	9.397822e-01	0.9997779456	-0.0593596832	0.8461873202	0.96338860	8.402089e-02	0.757178532	0.9982883	1.068801e-01	0.8090236464
0.89369078	-3.442023e-01	9.551234e-01	0.9997779456	-0.1441126757	0.5945916860	0.92532140	-2.152248e-01	0.284001767	0.9982883	-1.611770e-01	0.4930219413
0.81552863	-1.249752e-01	6.521761e-01	0.9997779456	0.1001166044	0.7644734272	0.95505354	4.412912e-01	0.127146577	0.9982883	2.839982e-01	0.7333996573
0.86909435	-6.821095e-01	9.955366e-01	0.9997779456	-0.8988045287	0.0005578348	0.09450935	-8.652308e-02	0.701078627	0.9982883	-4.120112e-01	0.1402420528
0.94189574	-7.303703e-01	7.345307e-01	0.9997779456	0.1764030510	0.8396208887	0.96321135	-7.578844e-01	0.441755328	0.9982883	-7.086108e-02	0.8918712751
0.77869908	-2.695614e-01	8.713349e-01	0.9997779456	-0.0777261763	0.8432170216	0.96338860	-2.536431e-01	0.281582489	0.9982883	-4.795183e-01	0.0834856799
0.92583146	3.493532e-02	4.361982e-01	0.9997779456	-0.2427835423	0.3313096843	0.85754449	-2.298704e-01	0.201564200	0.9982883	2.725876e-01	0.3041624885
0.99066584	-4.843376e-01	9.522578e-01	0.9997779456	0.1823488436	0.7487820789	0.95349818	-4.379914e-01	0.144701968	0.9982883	-3.085488e-01	0.3401577881
0.96804147	-3.770353e-01	8.405151e-01	0.9997779456	-0.0266965444	0.9168861468	0.98077776	-2.653317e-01	0.422837821	0.9982883	-4.197403e-01	0.1057323117
0.97273515	-3.235823e-01	8.923192e-01	0.9997779456	-0.0232749678	0.9391174232	0.98648257	2.050389e-01	0.320741936	0.9982883	-2.407005e-01	0.4321506279
0.97273515	-6.722285e-01	9.298832e-01	0.9997779456	0.7098125084	0.2614718903	0.84915316	6.720004e-01	0.095785861	0.9982883	-4.302587e-02	0.9308657465
0.92890486	-1.328330e-01	6.967216e-01	0.9997779456	-0.2216239345	0.4528706694	0.90612020	9.369637e-02	0.730672929	0.9982883	-6.270833e-01	0.0028731177
0.84824569	-4.641125e-01	9.651583e-01	0.9997779456	-0.1351409397	0.4777699027	0.90612020	1.065084e-01	0.610650506	0.9982883	-2.897390e-01	0.2371007564
0.87292023	-3.778965e-01	9.617038e-01	0.9997779456	-0.2656468853	0.2593796287	0.84915316	3.079010e-02	0.881982001	0.9982883	4.129573e-02	0.9132498239
0.77869908	1.926020e-01	2.371175e-01	0.9997779456	0.3076856230	0.3902913797	0.87916124	-1.577082e-01	0.506106634	0.9982883	-3.135177e-02	0.9286495950
0.80653217	-4.815513e-01	9.709756e-01	0.9997779456	-0.0214289522	0.9316551387	0.98590149	-3.511173e-01	0.095110064	0.9982883	-2.326623e-01	0.3782462448
0.97273515	-5.776632e-01	9.043469e-01	0.9997779456	0.5276513189	0.2388268356	0.84413548	4.676224e-01	0.187637960	0.9982883	8.731692e-02	0.8542183071
0.83725376	-1.607584e-01	9.062827e-01	0.9997779456	0.0723246934	0.6515158132	0.93854411	-1.176251e-01	0.328496200	0.9982883	-4.284548e-02	0.8504322008
0.84824569	-1.341891e-01	7.936711e-01	0.9997779456	-0.0798997255	0.7408264411	0.95076748	-1.274979e-01	0.422050955	0.9982883	1.195081e-01	0.6741877305
0.77869908	1.114627e-01	3.766806e-01	0.9997779456	-0.2587596781	0.4629913244	0.90612020	4.918328e-01	0.222720877	0.9982883	-5.255333e-01	0.1489311123
0.77869908	-6.970665e-01	9.566802e-01	0.9997779456	-0.4753501653	0.2121591986	0.83807199	-7.404374e-01	0.045912084	0.9982883	-6.964143e-01	0.1027763227
0.77869908	-1.136526e-01	7.682761e-01	0.9997779456	0.1103282995	0.5217164016	0.90612020	1.177161e-01	0.391905503	0.9982883	-1.981784e-01	0.3617446598
0.95472383	-4.180148e-02	6.113098e-01	0.9997779456	-0.4153850585	0.0028199684	0.30301842	5.515848e-02	0.618103536	0.9982883	-3.152345e-02	0.8266857450
0.90482806	9.243797e-02	3.890185e-01	0.9997779456	-0.3563509152	0.3865015044	0.87916124	-1.924510e-02	0.941225830	0.9986411	5.021107e-01	0.1816397201
0.77869908	5.136274e-01	1.747689e-01	0.9997779456	0.9597897357	0.2259875952	0.83807199	1.163640e-01	0.854534762	0.9982883	-2.873512e-01	0.6007473920
0.95472383	-8.238235e-02	7.080771e-01	0.9997779456	-0.1154639629	0.4209401165	0.89947025	-2.333760e-01	0.098301516	0.9982883	3.084708e-02	0.8606261181
0.96804147	-1.094718e-01	7.687776e-01	0.9997779456	0.0102510288	0.9507611867	0.99100505	-1.191662e-01	0.433755633	0.9982883	-3.953147e-02	0.8545330017
0.89273364	-1.895927e-01	7.150973e-01	0.9997779456	0.2881741829	0.4704591514	0.90612020	1.324608e-01	0.627440985	0.9982883	-1.186979e-01	0.7026152240
0.98111638	-3.185130e-01	7.926179e-01	0.9997779456	0.1507122073	0.5924740183	0.92532140	1.440924e-02	0.953866139	0.9986411	-3.157259e-01	0.1289637921
0.87292023	-1.824957e-01	9.515987e-01	0.9997779456	-0.1039492471	0.2772704203	0.84915316	-3.803585e-02	0.643371699	0.9982883	-2.709048e-02	0.7525571601
0.97522657	-3.043763e-01	9.806487e-01	0.9997779456	0.0720735727	0.6882593208	0.94482995	7.607618e-03	0.951277993	0.9986411	-4.491605e-01	0.0013692453
0.77869908	4.881717e-02	3.544450e-01	0.9997779456	-0.3046656602	0.0607236739	0.83807199	-1.000049e-01	0.452607141	0.9982883	-1.383692e-01	0.2232708063
0.80820047	4.700599e-02	1.747519e-01	0.9997779456	-0.0022277477	0.9740040306	0.99380888	2.789615e-02	0.636542730	0.9982883	8.345452e-03	0.9185998605
0.97273515	-1.813977e-01	8.303429e-01	0.9997779456	0.1095055145	0.5192340211	0.90612020	2.697070e-01	0.032641399	0.9982883	-8.020860e-02	0.4911822922
0.77869908	3.071983e-01	1.383186e-02	0.9997779456	-0.2241926871	0.2786668021	0.84915316	-5.145771e-02	0.708411869	0.9982883	3.960978e-01	0.0203716054
0.77869908	-6.418187e-02	6.906250e-01	0.9997779456	-0.0802173678	0.4786777832	0.90612020	1.131677e-01	0.310937491	0.9982883	-2.943811e-01	0.0022843958
0.88203342	2.718050e-03	4.890240e-01	0.9997779456	-0.0740426257	0.4942544089	0.90612020	1.008165e-01	0.196055607	0.9982883	1.340804e-01	0.2521293820
0.78828782	2.822746e-01	2.424036e-01	0.9997779456	-0.4484887560	0.3815991101	0.87916124	-4.115487e-01	0.246134152	0.9982883	-1.370561e-01	0.7939574160
0.83140044	2.404678e+00	2.759925e-02	0.9997779456	-2.0464313317	0.0696902380	0.83807199	-3.796766e-01	0.763525664	0.9982883	3.713660e-01	0.8080047637
0.77869908	-1.846221e-01	6.178929e-01	0.9997779456	-0.6049083654	0.0650494669	0.83807199	-5.276738e-01	0.330894219	0.9982883	-4.395881e-01	0.1880374755
0.90482806	-6.634170e-02	6.632369e-01	0.9997779456	0.0306348576	0.8377544462	0.96321135	1.368729e-01	0.261311571	0.9982883	-3.578430e-03	0.9828643308
0.87466006	2.630893e-01	8.554536e-02	0.9997779456	0.4455603837	0.0316316111	0.66765293	8.607022e-02	0.631927816	0.9982883	1.699176e-01	0.4455093685
0.77869908	-1.818441e-01	5.770474e-01	0.9997779456	0.4682161353	0.6323217483	0.93852299	1.080054e-01	0.905669209	0.9982883	-2.874626e-01	0.7246444514
0.77869908	5.195260e-03	4.936662e-01	0.9997779456	0.2160895598	0.6167085445	0.93239494	-2.237829e-01	0.487248211	0.9982883	-6.516258e-02	0.8745805621
0.77869908	-2.233211e-02	5.906376e-01	0.9997779456	-0.0925123241	0.3913106396	0.87916124	2.208596e-02	0.800521168	0.9982883	5.190403e-02	0.5339430774
0.97273515	-5.234986e-01	8.741171e-01	0.9997779456	-0.8981443326	0.0836928355	0.83807199	-9.230144e-02	0.812655038	0.9982883	-2.011457e-01	0.6682331985
0.89369078	-1.005608e-01	9.462987e-01	0.9997779456	-0.0291943228	0.7589944732	0.95505354	1.911775e-02	0.772786200	0.9982883	1.493480e-02	0.8794607883
0.98996967	1.989862e-02	4.452277e-01	0.9997779456	0.1296468812	0.4413450998	0.90612020	1.659673e-01	0.169569803	0.9982883	4.149535e-02	0.8520682085
0.84600172	-1.632281e-01	9.199924e-01	0.9997779456	-0.2064522815	0.1222292721	0.83807199	7.239981e-02	0.420455521	0.9982883	-5.618078e-03	0.9579114444
0.97273515	-3.018251e-01	9.369283e-01	0.9997779456	-0.1536884557	0.2520997582	0.84467092	-1.629217e-01	0.320415676	0.9982883	-1.620274e-01	0.3542597063
0.87292023	1.411695e-01	1.586784e-01	0.9997779456	0.3857662706	0.2105361286	0.83807199	1.666744e-01	0.219601792	0.9982883	2.756181e-01	0.1259464052
0.79213004	1.620272e-02	4.319173e-01	0.9997779456	0.1008084224	0.3833642418	0.87916124	1.189036e-01	0.208271740	0.9982883	1.631190e-01	0.1362948893
0.77869908	7.139094e-02	1.827586e-01	0.9997779456	0.0476516125	0.6723475570	0.94130886	-6.618917e-03	0.925998741	0.9982883	9.338593e-02	0.2213958345
0.97050390	2.993828e-02	3.677730e-01	0.9997779456	-0.1047354158	0.3920964971	0.87916124	-1.023498e-01	0.187037431	0.9982883	-9.574266e-02	0.3786319284
0.89512661	1.111888e-01	1.249593e-01	0.9997779456	-0.1969924075	0.2197694976	0.83807199	-5.692685e-02	0.545383047	0.9982883	-2.540550e-02	0.8306212520
0.90482806	1.271099e-02	4.577257e-01	0.9997779456	-0.1576419664	0.2167350168	0.83807199	-1.476046e-01	0.089370117	0.9982883	1.557763e-05	0.9998858888
0.77869908	-4.336589e-02	5.244906e-01	0.9997779456	0.4459500819	0.5260035002	0.90612020	-9.455126e-02	0.898440112	0.9982883	-3.021345e-01	0.6467236837
0.99066584	-2.831195e-01	9.508216e-01	0.9997779456	-0.4282667876	0.0863878469	0.83807199	5.156874e-02	0.751267894	0.9982883	8.049497e-02	0.6616705597
0.80820047	4.939914e-02	4.519512e-01	0.9997779456	0.5426841755	0.3905084212	0.87916124	2.552916e-01	0.523728634	0.9982883	2.721462e-01	0.6573643539
0.77869908	3.842338e-01	2.195687e-01	0.9997779456	0.2603839005	0.6390322310	0.93854411	1.568280e-01	0.807221518	0.9982883	-2.544388e-01	0.6480888312
0.86909435	7.369798e-02	4.232830e-01	0.9997779456	0.0752145593	0.8215693174	0.95972154	9.531777e-02	0.808695392	0.9982883	6.934564e-03	0.9839627965
0.92876201	1.886730e-01	8.226621e-02	0.9997779456	-0.0899822006	0.4404651875	0.90612020	-1.054605e-01	0.364251500	0.9982883	-4.567654e-03	0.9634297788
0.92117830	2.041075e-03	4.919534e-01	0.9997779456	-0.2068667623	0.1266322676	0.83807199	-6.915834e-02	0.532863525	0.9982883	6.969908e-02	0.6892108012
0.77869908	-2.297945e-01	9.665362e-01	0.9997779456	-0.2455374209	0.2296163191	0.84082280	-1.076297e-01	0.306076923	0.9982883	-1.519063e-01	0.1625132956
0.82862325	-4.431814e-02	6.234702e-01	0.9997779456	-0.2151514585	0.1891621033	0.83807199	1.583135e-02	0.868371659	0.9982883	9.406586e-02	0.3121493243
0.77869908	4.757894e-02	2.899092e-01	0.9997779456	-0.0051362916	0.9650773269	0.99380888	1.112156e-01	0.206081200	0.9982883	-7.478819e-02	0.4721546032
0.77869908	4.264227e-01	9.863481e-02	0.9997779456	-0.2749511393	0.5824282836	0.92532140	-4.908182e-01	0.184337193	0.9982883	5.986896e-01	0.2951125885
0.77869908	1.793487e-01	1.447829e-02	0.9997779456	-0.0638656072	0.5230906299	0.90612020	-2.846749e-03	0.971957933	0.9986411	8.722231e-02	0.2393944145
0.82862325	-4.749961e-02	6.711825e-01	0.9997779456	-0.0099460795	0.9478647189	0.98973154	-3.822377e-02	0.702636944	0.9982883	2.547610e-01	0.0436492284
0.77869908	1.065487e-01	3.490600e-02	0.9997779456	-0.0732005645	0.3976394555	0.88596624	4.694568e-02	0.385061293	0.9982883	1.116665e-01	0.2232590402
0.77869908	-7.170981e-01	8.882148e-01	0.9997779456	-0.3243506082	0.3176168345	0.84915316	-2.524405e-01	0.608892884	0.9982883	-1.779725e-01	0.5737656549
0.92876201	-3.168861e-01	9.758743e-01	0.9997779456	-0.1919577805	0.0147318273	0.49365173	-1.010410e-01	0.351686114	0.9982883	-2.591152e-01	0.0038637434
0.77869908	-8.549957e-02	7.753807e-01	0.9997779456	0.0100203000	0.9331934131	0.98590149	6.975027e-02	0.538663812	0.9982883	1.673933e-01	0.1392498180
0.86909435	-1.785960e-02	5.266130e-01	0.9997779456	-0.5416019619	0.0908560459	0.83807199	-2.182823e-01	0.378477631	0.9982883	4.609332e-01	0.3438786215
0.99436660	-4.643254e-01	9.742923e-01	0.9997779456	-0.0940969006	0.8122352507	0.95972154	-5.899876e-02	0.830271469	0.9982883	-5.967903e-01	0.1630057978
0.81804251	1.140423e-01	1.170437e-01	0.9997779456	0.1863167461	0.0746638611	0.83807199	1.052831e-01	0.300010051	0.9982883	1.794426e-01	0.1785147974
0.88636459	5.890747e-01	6.626268e-02	0.9997779456	0.4901167326	0.5136960959	0.90612020	-9.553524e-02	0.826069465	0.9982883	-7.314079e-02	0.8837148918
0.97050390	3.473129e-02	4.534839e-01	0.9997779456	0.0613336681	0.8738551599	0.96947881	-1.925518e-01	0.478750221	0.9982883	1.987047e-01	0.5379201277
0.77869908	2.201715e-01	9.930026e-02	0.9997779456	0.2213219333	0.1343177400	0.83807199	7.635050e-02	0.541280343	0.9982883	2.484638e-01	0.0624414739
0.88540806	-6.130803e-03	5.259472e-01	0.9997779456	-0.2300648488	0.0388084700	0.76504055	-1.034187e-01	0.273572326	0.9982883	2.816046e-02	0.8463448181
0.77869908	1.447091e-01	9.948310e-02	0.9997779456	0.0044458798	0.9622313662	0.99380888	-3.989238e-02	0.681439672	0.9982883	1.157511e-01	0.1972113365
0.81804251	6.166015e-03	4.829548e-01	0.9997779456	-0.3249535109	0.0216954207	0.60526393	1.938697e-02	0.868535498	0.9982883	-3.904166e-02	0.8391802861
0.90482806	1.105746e-01	1.985794e-01	0.9997779456	0.0970326235	0.4757237506	0.90612020	-9.402357e-02	0.479698294	0.9982883	1.815695e-01	0.3269139561
0.77869908	-3.069863e-01	9.316526e-01	0.9997779456	-0.1274729145	0.5064685070	0.90612020	3.961013e-02	0.817147869	0.9982883	1.031831e-01	0.7076877126
0.97717546	-3.376104e-03	5.256186e-01	0.9997779456	0.0296891802	0.7633440884	0.95505354	8.935441e-02	0.126722422	0.9982883	7.637657e-02	0.3398791198
0.77869908	-1.445024e-02	5.919559e-01	0.9997779456	-0.0522333426	0.6303344525	0.93835683	-3.893125e-03	0.946308025	0.9986411	-5.173325e-02	0.6049585434
0.77869908	6.649458e-01	2.629942e-02	0.9997779456	0.3771039086	0.4141929599	0.89754931	-1.977306e-01	0.678382114	0.9982883	-1.559266e-01	0.7704571178
0.97273515	9.978639e-01	1.097334e-02	0.9997779456	-1.0759268364	0.0113369231	0.46180258	3.656200e-01	0.410901622	0.9982883	3.127066e-01	0.5998428594
0.88636459	-9.945713e-02	8.947120e-01	0.9997779456	0.0420483002	0.6269814139	0.93690522	-1.058207e-02	0.890036538	0.9982883	4.154674e-02	0.6772408410
0.83725376	-8.139273e-02	7.559659e-01	0.9997779456	0.0445581724	0.7699138122	0.95505354	6.240363e-02	0.549201043	0.9982883	-1.193698e-02	0.9123391381
0.79578964	7.288934e-02	3.072898e-01	0.9997779456	0.0564731614	0.7126160363	0.94482995	1.911292e-01	0.140454014	0.9982883	2.961973e-01	0.0421669140
0.77869908	3.978951e-01	3.774037e-01	0.9997779456	2.1540512043	0.2038869012	0.83807199	3.600475e-02	0.974925395	0.9986411	6.533837e-01	0.7019001660
0.97273515	6.755653e-02	4.146609e-01	0.9997779456	0.0129419478	0.9740442518	0.99380888	-4.531600e-01	0.142640784	0.9982883	-2.940633e-01	0.4162370067
0.77869908	9.228959e-02	4.229383e-01	0.9997779456	0.6133486306	0.2323807241	0.84413548	3.413280e-01	0.434580690	0.9982883	1.719309e-01	0.7618879560
0.77869908	1.683173e-01	2.626541e-01	0.9997779456	0.0476625821	0.8948394760	0.97246977	1.049753e-01	0.629058053	0.9982883	4.824599e-02	0.8252815864
0.84824569	-1.193485e-01	7.559690e-01	0.9997779456	-0.2255908443	0.1832810524	0.83807199	1.405768e-01	0.382861026	0.9982883	-3.151958e-02	0.8454621412
0.92583146	3.597576e-02	4.302666e-01	0.9997779456	0.0287386672	0.8876266296	0.97235837	-3.835791e-02	0.795754718	0.9982883	-9.297854e-02	0.5829132664
0.86909435	4.485461e-01	1.096073e-01	0.9997779456	0.5358387387	0.1481162163	0.83807199	-7.173265e-02	0.875920686	0.9982883	4.672896e-01	0.3813605359
0.99018039	-3.806403e-01	9.959091e-01	0.9997779456	-0.4016167607	0.0309585763	0.66532795	-1.295372e-01	0.318601823	0.9982883	5.109448e-02	0.7260793566
0.87292023	-2.180440e-03	5.139759e-01	0.9997779456	-0.0896865418	0.3084013659	0.84915316	-1.527172e-02	0.802749567	0.9982883	-8.442303e-02	0.2289069124
0.97273515	1.692433e-01	1.754118e-01	0.9997779456	0.1734231416	0.3392751164	0.85925321	-1.078388e-01	0.553996778	0.9982883	1.701428e-02	0.9516477360
0.93924745	5.739739e-02	3.320109e-01	0.9997779456	0.2725101211	0.1563202719	0.83807199	3.241676e-02	0.797208437	0.9982883	-3.059946e-01	0.0531145725
0.81804251	-2.453500e-01	8.593142e-01	0.9997779456	0.1959909385	0.5964959216	0.92532140	-2.184320e-01	0.366015481	0.9982883	-2.186504e-01	0.4718263664
0.84600172	-1.350872e-01	8.113607e-01	0.9997779456	-0.1413431874	0.3683562166	0.87664366	2.685253e-02	0.847250691	0.9982883	-1.581910e-01	0.2252410245
0.77869908	-1.326264e-01	7.205801e-01	0.9997779456	0.2560073393	0.4333990766	0.90612020	9.360282e-02	0.676540159	0.9982883	1.076712e-01	0.6660963941
0.81552863	7.381356e-02	2.170074e-01	0.9997779456	-0.0344439193	0.7884115715	0.95972154	7.452308e-02	0.390090551	0.9982883	7.416429e-02	0.3829279874

0.97273515	5.142027e-01	2.158380e-01	0.9997779456	-0.5032658209	0.4674743020	0.90612020	4.253533e-01	0.527920113	0.9982883	-1.073025e+00	0.1222495800
0.97273515	-2.968401e-01	9.767287e-01	0.9997779456	-0.1609501556	0.4443073239	0.90612020	1.719428e-01	0.243653450	0.9982883	1.566193e-01	0.2599764677
0.93081046	-4.151916e-02	5.320120e-01	0.9997779456	-0.8177756866	0.1510819123	0.83807199	-1.254076e-01	0.782119595	0.9982883	-2.567446e-01	0.7919706217
0.79578964	7.824358e-02	4.261807e-01	0.9997779456	0.0552777368	0.8942902567	0.97246977	8.618315e-02	0.836283025	0.9982883	-1.236258e-01	0.7889518494
0.77869908	1.688801e+00	7.037455e-02	0.9997779456	-0.0277651778	0.9816270811	0.99380888	2.371172e+00	0.063757953	0.9982883	1.091279e+00	0.5968015323
0.77869908	-1.480117e-02	5.703376e-01	0.9997779456	-0.2225869470	0.1639492083	0.83807199	2.597588e-02	0.719906450	0.9982883	-5.345094e-04	0.9962460267
0.96416435	2.990484e-02	3.647931e-01	0.9997779456	-0.0304433281	0.8188572864	0.95972154	-4.824024e-03	0.954532652	0.9986411	-1.581688e-01	0.1638333124
0.77869908	-1.459985e-01	7.963674e-01	0.9997779456	-0.2686566282	0.2942444747	0.84915316	-1.105615e-01	0.423476737	0.9982883	-1.776595e-01	0.5151121481
0.81552863	-8.072344e-02	6.815949e-01	0.9997779456	0.0645474623	0.8470200074	0.96338860	-1.640989e-02	0.915579437	0.9982883	-1.712445e-02	0.9078106015
0.98544646	2.943906e-02	4.546454e-01	0.9997779456	-0.2827440601	0.1644851887	0.83807199	-1.097865e-01	0.613116920	0.9982883	-2.443085e-01	0.5432744237
0.77869908	4.752300e-01	1.049349e-01	0.9997779456	0.2701103869	0.5950109428	0.92532140	5.520629e-01	0.154159335	0.9982883	-2.691251e-01	0.6990311333
0.90482806	-2.955081e-01	6.630615e-01	0.9997779456	0.5576388797	0.3662636906	0.87664366	5.876397e-01	0.281177979	0.9982883	2.259317e-01	0.6345712804
0.77869908	-7.165914e-02	7.224889e-01	0.9997779456	-0.0490022968	0.6418853345	0.93854411	3.188828e-02	0.745488825	0.9982883	-3.342328e-02	0.7959537922
0.97185522	-1.589487e-01	9.108737e-01	0.9997779456	-0.0333557991	0.7798443632	0.95657490	-9.468640e-03	0.926381617	0.9982883	3.430679e-02	0.7750399158
0.95639607	2.379207e-01	3.747486e-02	0.9997779456	0.1257323005	0.5247088836	0.90612020	-1.420025e-01	0.260998193	0.9982883	-3.604326e-02	0.8198578706
0.82743594	2.095984e-01	1.623342e-01	0.9997779456	-0.0397378244	0.8269568958	0.95988696	2.935511e-01	0.181486696	0.9982883	-2.279429e-01	0.5631013936
0.95284371	-1.809241e-01	8.802572e-01	0.9997779456	-0.1450088399	0.4546448708	0.90612020	-8.128925e-02	0.467106031	0.9982883	-3.694680e-02	0.7688744478
0.77869908	1.199660e-01	4.939360e-02	0.9997779456	-0.0390966125	0.6500181642	0.93854411	-5.202996e-03	0.942824448	0.9986411	-6.456493e-02	0.5894358524
0.89273364	-1.488298e-01	7.934419e-01	0.9997779456	0.1398214327	0.4215791182	0.89947025	2.289176e-01	0.084346356	0.9982883	5.348034e-02	0.8403885570
0.77869908	1.702484e-01	2.316324e-01	0.9997779456	0.1314027061	0.6366525895	0.93852299	6.230350e-02	0.745595956	0.9982883	1.808360e-01	0.5424780406
0.77869908	-8.874530e-02	7.694847e-01	0.9997779456	-0.1783809025	0.1424354259	0.83807199	-1.769023e-02	0.843062522	0.9982883	1.870522e-01	0.1287740029
0.81804251	2.064395e-01	4.123413e-01	0.9997779456	1.1074108188	0.3106372931	0.84915316	8.180158e-01	0.348305449	0.9982883	-3.933792e-02	0.9681677790
0.77869908	7.118911e-02	1.692153e-01	0.9997779456	0.0403695803	0.7160712353	0.94569408	4.491397e-02	0.498786637	0.9982883	5.020752e-02	0.5571294512
0.99258481	-2.752909e-01	9.622325e-01	0.9997779456	-0.1050275239	0.5332037137	0.90627295	2.682965e-02	0.822154257	0.9982883	-1.082655e-01	0.5287900912
0.97522657	-1.035871e-01	8.337120e-01	0.9997779456	-0.0228349888	0.8064280786	0.95972154	-2.758299e-02	0.767188007	0.9982883	-4.381890e-02	0.6778505861
0.77869908	1.011204e-02	4.577119e-01	0.9997779456	0.0593187108	0.5843816715	0.92532140	1.437605e-01	0.047810760	0.9982883	-4.697671e-03	0.9556009593
0.77869908	-5.553372e-02	7.529560e-01	0.9997779456	-0.0930316631	0.3127960671	0.84915316	6.616241e-02	0.328481605	0.9982883	1.257297e-01	0.1153548026
0.77869908	4.031147e-02	2.579569e-01	0.9997779456	0.0098430079	0.9210487081	0.98256279	5.474823e-02	0.301677360	0.9982883	9.719856e-02	0.1679957517
0.77869908	-6.589811e-02	6.828358e-01	0.9997779456	-0.2303718650	0.1380745782	0.83807199	-1.113060e-01	0.411792001	0.9982883	-7.772360e-02	0.4376409824
0.80820047	1.201071e-01	1.903806e-01	0.9997779456	0.0629941296	0.7322001738	0.94712279	-1.696751e-02	0.857814115	0.9982883	1.931924e-01	0.0882704917
0.99258481	-1.223635e-01	8.808410e-01	0.9997779456	0.1518295087	0.3872465756	0.87916124	8.068232e-03	0.936341567	0.9986411	-1.434347e-01	0.1184218060
0.95181672	2.445024e-02	4.656568e-01	0.9997779456	0.4072098076	0.2189046298	0.83807199	1.237849e-01	0.579006206	0.9982883	1.406393e-01	0.4892660339
0.92890486	2.157305e-02	4.567457e-01	0.9997779456	0.2311465830	0.3481409774	0.86676800	-1.246057e-01	0.609627133	0.9982883	-1.040880e-01	0.5945758430
0.93171119	9.301106e-02	3.195386e-01	0.9997779456	0.1334771705	0.5312532166	0.90612020	-4.880003e-02	0.780266198	0.9982883	1.373604e-01	0.4827201128
0.97273515	1.532317e-01	1.463274e-01	0.9997779456	0.1757130834	0.4945753509	0.90612020	4.329156e-02	0.744663714	0.9982883	-7.091221e-02	0.6043386652
0.97273515	-8.198090e-01	8.312189e-01	0.9997779456	-0.2207439790	0.5527752869	0.91608209	1.974857e-01	0.658616770	0.9982883	-6.419719e-01	0.1032919923
0.97050390	1.341117e-01	1.212053e-01	0.9997779456	0.1369716380	0.3413524498	0.86029551	-1.444198e-02	0.877756493	0.9982883	-5.159316e-02	0.7440135809
0.98111638	-4.030940e-02	6.213088e-01	0.9997779456	0.0892275472	0.4988670399	0.90612020	-4.405266e-02	0.720505845	0.9982883	1.991307e-01	0.1003366819
0.99066584	3.136701e-02	3.649542e-01	0.9997779456	0.2532949253	0.1504647556	0.83807199	2.060564e-01	0.025813143	0.9982883	1.356996e-01	0.2217526486
0.80653217	2.763405e-02	3.724870e-01	0.9997779456	-0.0245781990	0.7418016792	0.95098653	-1.556643e-02	0.844397631	0.9982883	8.023817e-02	0.4696038315
0.77869908	-9.611748e-03	5.335216e-01	0.9997779456	-0.1317079490	0.2415306843	0.84413548	-3.045744e-02	0.713677316	0.9982883	5.343654e-02	0.5605878248
0.96804147	-3.212658e-02	6.347577e-01	0.9997779456	0.0647932679	0.6562222615	0.93854411	2.779146e-03	0.973447317	0.9986411	-4.532245e-02	0.6081993012
0.97273515	-1.928328e-01	9.646793e-01	0.9997779456	-0.0003410065	0.9976913472	0.99853613	4.724451e-02	0.596434331	0.9982883	1.079049e-01	0.3345004294
0.92890486	-2.068205e-01	9.059993e-01	0.9997779456	-0.2195986632	0.0734184951	0.83807199	-1.517830e-01	0.201252650	0.9982883	5.676299e-02	0.7575022686
0.80820047	-1.488717e-01	7.984908e-01	0.9997779456	-0.3764070010	0.0062821467	0.40915301	-2.659633e-01	0.058456716	0.9982883	-9.483499e-02	0.6362190415
0.84824569	7.800897e-02	3.041481e-01	0.9997779456	0.3304277521	0.1899140936	0.83807199	8.840899e-02	0.564013608	0.9982883	3.795685e-02	0.8657995676
0.99018039	-4.696742e-02	6.806128e-01	0.9997779456	0.2296393949	0.2406210833	0.84413548	7.030644e-02	0.520367213	0.9982883	-1.511712e-01	0.2573669966
0.98996967	-9.211026e-02	7.776548e-01	0.9997779456	0.1341998983	0.4668328417	0.90612020	-7.084661e-02	0.470204701	0.9982883	-1.089504e-02	0.9200091107
0.99066584	3.803074e-02	4.105161e-01	0.9997779456	0.2161127989	0.3124312717	0.84915316	-1.546353e-02	0.916489794	0.9982883	-3.636168e-01	0.0164199493
0.97522657	-3.020280e-01	7.342587e-01	0.9997779456	-0.1635212166	0.5086246318	0.90612020	-1.740480e-01	0.490656906	0.9982883	-4.414413e-01	0.0558507822
0.97273515	6.040986e-02	2.957655e-01	0.9997779456	0.2650279391	0.1655165036	0.83807199	2.462343e-02	0.777846765	0.9982883	6.738986e-02	0.5071176308
0.81163165	-1.469051e-01	7.183217e-01	0.9997779456	0.0417422716	0.8399593388	0.96321135	1.584706e-01	0.437473510	0.9982883	2.059270e-02	0.8971868690
0.99066584	-2.164653e-01	9.275008e-01	0.9997779456	0.0589815264	0.7236275411	0.94712279	-2.209380e-02	0.875382063	0.9982883	-3.850836e-01	0.0012010214
0.96804147	-1.461494e-01	6.997683e-01	0.9997779456	-0.2529739406	0.4762349146	0.90612020	-3.888967e-01	0.158126237	0.9982883	-4.373730e-03	0.9871104010
0.97144018	-4.732465e-01	9.638978e-01	0.9997779456	-0.2407420571	0.2918934111	0.84915316	1.013414e-01	0.625302343	0.9982883	-7.096298e-01	0.0392184909
0.81552863	-1.651462e-01	8.736104e-01	0.9997779456	0.2210604753	0.4375013378	0.90612020	1.384044e-01	0.310877689	0.9982883	8.534843e-02	0.6954283407
0.87292023	-2.102400e-01	8.989912e-01	0.9997779456	-0.0773072277	0.7862499083	0.95907884	-2.021807e-01	0.289529063	0.9982883	-1.578629e-01	0.3945866994
0.77869908	-6.072872e-02	5.935898e-01	0.9997779456	-0.2839665524	0.3921684894	0.87916124	-1.071604e-01	0.684917344	0.9982883	-5.071018e-01	0.1623302217
0.77869908	1.832061e-01	3.438210e-01	0.9997779456	-0.5764208148	0.2204230095	0.83807199	4.682171e-01	0.327613473	0.9982883	-4.426181e-01	0.3365078162
0.97185522	-4.049116e-01	9.871581e-01	0.9997779456	-0.2343679151	0.3335756214	0.85880449	-2.274958e-01	0.177075365	0.9982883	-4.472576e-01	0.0374598677
0.98111638	-5.242068e-01	9.594476e-01	0.9997779456	0.1098668768	0.6798456755	0.94316618	-2.931997e-01	0.274917284	0.9982883	-2.379446e-01	0.3082085311
0.79578964	-2.477579e-01	8.822588e-01	0.9997779456	0.0415862638	0.9006165389	0.97484318	1.067819e-01	0.583199753	0.9982883	-2.533391e-03	0.9877348817
0.97522657	-1.017693e-01	6.600615e-01	0.9997779456	0.0280556090	0.9188987837	0.98201354	4.231758e-02	0.839987125	0.9982883	-3.601961e-01	0.1549245406
0.96804147	-7.269329e-02	6.771591e-01	0.9997779456	-0.4170932023	0.1537872456	0.83807199	-2.071651e-01	0.192616382	0.9982883	1.709676e-01	0.3633801460
0.99066584	-2.921302e-01	8.809102e-01	0.9997779456	0.1876170789	0.5639694247	0.91946463	1.255906e-01	0.465428264	0.9982883	5.243968e-02	0.7562412414
0.96804147	5.016558e-01	1.785323e-01	0.9997779456	0.7615053665	0.2271298804	0.83807199	5.459834e-01	0.245592353	0.9982883	3.949690e-01	0.5361275844
0.98996967	-9.975578e-02	6.124957e-01	0.9997779456	-0.3988746786	0.2627703100	0.84915316	-5.214091e-02	0.881453638	0.9982883	-2.747706e-01	0.4366250435
0.77869908	-3.046949e-01	8.737739e-01	0.9997779456	-0.3751638646	0.1198456139	0.83807199	2.855180e-01	0.178909621	0.9982883	-3.911171e-01	0.0522555341
0.97273515	-2.837009e-01	8.362525e-01	0.9997779456	0.0906446528	0.8162617948	0.95972154	-1.089259e-01	0.648857385	0.9982883	-4.506433e-01	0.0539875717
0.97273515	-4.193963e-01	9.691742e-01	0.9997779456	0.1101855817	0.7239959385	0.94712279	7.437965e-02	0.710726533	0.9982883	-3.725180e-01	0.0210792062
0.86909435	9.178698e-02	3.109433e-01	0.9997779456	0.1579821309	0.6216577677	0.93367151	2.193521e-01	0.214681526	0.9982883	-5.439937e-02	0.8155571025
0.87292023	9.785494e-02	3.069493e-01	0.9997779456	-0.4889405014	0.2259130521	0.83807199	-2.426297e-01	0.350101253	0.9982883	-2.688011e-02	0.9385509685
0.97273515	-5.771507e-01	9.794269e-01	0.9997779456	-0.2811104857	0.1830613701	0.83807199	7.144519e-02	0.700572598	0.9982883	-3.757592e-01	0.1077690858
0.77869908	-3.299697e-01	8.256588e-01	0.9997779456	-1.0773822283	0.0002182013	0.09450935	9.535747e-02	0.731407497	0.9982883	-1.700009e-01	0.7292277633
0.97522657	1.871951e-01	2.058488e-01	0.9997779456	-0.0470694933	0.8827996680	0.97066903	-2.213374e-01	0.459178200	0.9982883	4.698288e-01	0.3096981721
0.98226159	-8.091911e-02	6.312111e-01	0.9997779456	-0.0047593403	0.9856872555	0.99380888	-2.668381e-01	0.195434034	0.9982883	-4.768934e-01	0.0191112373
0.77869908	2.700922e-02	4.026977e-01	0.9997779456	0.2715969325	0.2473600534	0.84413548	7.202675e-02	0.566954691	0.9982883	-2.941771e-01	0.0962709084
0.89369078	-1.442013e-01	8.112604e-01	0.9997779456	-0.2404533782	0.3597219751	0.87404187	-1.812308e-01	0.336285452	0.9982883	-3.377919e-01	0.1093837011
0.88001839	2.085672e-01	1.558844e-01	0.9997779456	0.0348407983	0.9040181576	0.97582904	6.129685e-02	0.746412854	0.9982883	-3.531910e-02	0.9085394201
0.97185522	-1.435227e-02	5.198436e-01	0.9997779456	-0.0233403520	0.9120358654	0.97964668	9.834229e-02	0.671377394	0.9982883	5.108186e-01	0.1638151133
0.80820047	-6.784049e-02	6.896228e-01	0.9997779456	-0.0062619834	0.9692757397	0.99380888	-1.115149e-01	0.405658811	0.9982883	-4.229445e-01	0.0678820562
0.96804147	-4.464380e-02	5.868871e-01	0.9997779456	0.3404445270	0.3257853264	0.85614181	3.912726e-02	0.841097832	0.9982883	-5.171592e-01	0.0720305236
0.98996967	-2.110004e-01	7.074836e-01	0.9997779456	0.0154460142	0.9598032237	0.99380888	2.328990e-02	0.925091841	0.9982883	-4.216494e-01	0.0696747552
0.87099999	4.081623e-01	1.511387e-01	0.9997779456	0.7257976870	0.3596727173	0.87404187	4.133040e-01	0.327835963	0.9982883	-3.142334e-01	0.4945677785
0.92876201	1.862113e-01	2.707387e-01	0.9997779456	-0.5790149477	0.0499305412	0.83664873	-4.170466e-01	0.172342614	0.9982883	6.611657e-01	0.1791284082
0.77869908	-1.487739e-01	7.402527e-01	0.9997779456	0.2375443722	0.2433420273	0.84413548	1.335993e-01	0.474938664	0.9982883	-2.389593e-01	0.4416907076
0.97273515	9.335099e-01	2.998299e-01	0.9997779456	-2.4318999211	0.2807486118	0.84915316	-2.551256e+00	0.386472288	0.9982883	-1.205329e+00	0.4928942989
0.77869908	-1.423002e-01	5.810041e-01	0.9997779456	-0.3307212889	0.5742333443	0.92122660	-1.507594e-01	0.797348966	0.9982883	-5.810006e-01	0.3513992324
0.83725376	-2.913036e-02	5.967374e-01	0.9997779456	-0.0358480671	0.7944064898	0.95972154	2.826130e-02	0.766729465	0.9982883	-4.803804e-02	0.6712458115
0.97273515	-2.523455e-01	8.865187e-01	0.9997779456	-0.2648481632	0.5446385069	0.91515867	-1.879362e-01	0.375812985	0.9982883	1.655457e-02	0.9263386659
0.99066584	-1.005044e-02	5.138989e-01	0.9997779456	-0.4390773894	0.3206127743	0.85352320	-6.858448e-01	0.016973145	0.9982883	-4.013532e-01	0.4736884675
0.96804147	1.371320e-01	2.784134e-01	0.9997779456	0.0816693484	0.7446066201	0.95148651	-9.146268e-03	0.966259156	0.9986411	1.188650e-01	0.7002881127
0.80521409	-7.077022e-02	8.366006e-01	0.9997779456	-0.0105946572	0.9150628984	0.98058109	-8.535067e-03	0.900244758	0.9982883	6.429027e-02	0.5131092457
0.95472383	-4.622363e-02	6.431793e-01	0.9997779456	0.1413554197	0.5135772977	0.90612020	-2.664109e-02	0.795777599	0.9982883	-1.249842e-01	0.4823187055
0.97273515	-2.239341e-01	8.762715e-01	0.9997779456	0.2975833369	0.2360819418	0.84413548	1.800174e-01	0.216243389	0.9982883	3.222881e-02	0.8912268238
0.98071467	-2.501463e-02	5.853088e-01	0.9997779456	0.1172312267	0.5151395025	0.90612020	-3.105259e-02	0.758553111	0.9982883	1.543619e-02	0.8976683212
0.91967042	1.076414e-02	4.751407e-01	0.9997779456	0.2622860681	0.3076123748	0.84915316	2.800157e-02	0.832941820	0.9982883	-8.757901e-02	0.6278989579
0.95134638	1.293884e-01	2.983118e-01	0.9997779456	0.5744016380	0.1540158879	0.83807199	2.938812e-01	0.160690264	0.9982883	-1.292727e-01	0.5871194524
0.95181672	9.014480e-02	3.471321e-01	0.9997779456	0.4316310924	0.3057914636	0.84915316	2.076026e-01	0.329998141	0.9982883	-3.709865e-01	0.1163349873
0.99436660	-3.432148e-02	6.118757e-01	0.9997779456	0.3030522821	0.1671543425	0.83807199	1.560082e-01	0.119511160	0.9982883	-7.043479e-02	0.3470308086
0.98714249	-3.063650e-02	6.053961e-01	0.9997779456	0.0028664073	0.9785415872	0.99380888	1.026544e-01	0.266799204	0.9982883	1.651539e-01	0.1147958069
0.99066584	-4.351323e-02	6.367901e-01	0.9997779456	0.3192278002	0.1673274851	0.83807199	2.201113e-01	0.047766809	0.9982883	-5.061428e-02	0.6251496838
0.77869908	1.440605e-01	4.079309e-01	0.9997779456	0.9631165074	0.2245837142	0.83807199	5.181467e-01	0.317698967	0.9982883	-2.073766e-01	0.7437187984
0.77869908	3.808470e-02	4.704322e-01	0.9997779456	0.2222044548	0.6478568028	0.93854411	9.224306e-02	0.854177672	0.9982883	-7.130308e-02	0.8851939730
0.96804147	-2.752833e-01	7.268300e-01	0.9997779456	0.5791381261	0.5710539034	0.92122660	6.738336e-01	0.095428696	0.9982883	-2.942677e-01	0.4954991026
0.95472383	2.049253e-01	1.782998e-01	0.9997779456	0.3880801780	0.2783289377	0.84915316	1.704451e-01	0.372888675	0.9982883	-2.590891e-02	0.8686265979
0.97522657	-3.557545e-01	9.667271e-01	0.9997779456	-0.1477159742	0.2836461938	0.84915316	-1.151578e-02	0.926378904	0.9982883	-3.088648e-01	0.0750965286
0.77869908	6.940759e-02	4.273732e-01	0.9997779456	-0.0904598119	0.8165416235	0.95972154	-3.234136e-01	0.395852159	0.9982883	-8.481262e-01	0.0023995838
0.99066584	-2.397804e-01	9.606150e-01	0.9997779456	0.1203542665	0.2375393936	0.84413548	7.155376e-02	0.459619662	0.9982883	-1.108308e-01	0.3481581121
0.95472383	7.737687e-02	4.124277e-01	0.9997779456	0.5484474025	0.5368197233	0.90738404	2.615976e-01	0.448036681	0.9982883	1.520910e-01	0.6701650300
0.77869908	5.626454e-02	3.746712e-01	0.9997779456	0.0232840762	0.9197030353	0.98201354	-7.317384e-02	0.719258260	0.9982883	-1.199164e-02	0.9519431393
0.98996967	-6.001114e-01	9.589572e-01	0.9997779456	-0.1833019526	0.4788657465	0.90612020	-1.294227e-01	0.549765164	0.9982883	-2.652973e-01	0.3427656401
0.99066584	-1.213772e-01	8.412315e-01	0.9997779456	0.1116543552	0.5877510868	0.92532140	1.609388e-01	0.213162504	0.9982883	-4.032637e-01	0.0093188790
0.97273515	1.201840e-01	3.899037e-01	0.9997779456	0.4790010813	0.0842842663	0.83807199	2.671602e-01	0.273168652	0.9982883	-1.516296e-01	0.5357926410
0.97273515	-4.967863e-01	9.073096e-01	0.9997779456	-0.1018269378	0.6603033383	0.93982803	1.650258e-01	0.453692184	0.9982883	-1.375865e-01	0.5383028957
0.97273515	-3.008918e-01	8.148437e-01	0.9997779456	-0.3028930605	0.1576190417	0.83807199	1.240649e-01	0.544480054	0.9982883	3.652995e-01	0.1715529052
0.99018039	-4.041383e-02	6.571621e-01	0.9997779456	0.0788994090	0.5351764126	0.90627295	2.208632e-02	0.782113385	0.9982883	-8.041800e-02	0.3004685098
0.97185522	2.860888e-01	3.581987e-02	0.9997779456	0.3481645448	0.2742894538	0.84915316	2.169979e-01	0.098550274	0.9982883	-1.151246e-01	0.4406839311
0.80820047	2.358241e-02	3.576676e-01	0.9997779456	0.0170073401	0.9027129347	0.97582904	-4.031399e-02	0.549490617	0.9982883	-4.014106e-02	0.5809265927
0.99066584	-1.330071e-01	8.874363e-01	0.9997779456	0.0786185341	0.6140860332	0.93177111	5.941625e-04	0.994703434	0.9988193	3.117671e-02	0.7291927311
0.88505393	8.423974e-02	2.385619e-01	0.9997779456	0.0111033449	0.9383167319	0.98648257	-2.151566e-01	0.066733563	0.9982883	7.165819e-02	0.6175583847
0.90482806	-3.962998e-02	6.040092e-01	0.9997779456	0.0042603597	0.9777107328	0.99380888	-1.362722e-01	0.161288635	0.9982883	-1.024365e-01	0.3638826023
0.77869908	3.984981e-01	2.854836e-01	0.9997779456	1.5024669575	0.2814929326	0.84915316	8.125223e-01	0.268627487	0.9982883	1.163678e-01	0.8984573116
0.98670901	-1.623334e-02	5.646172e-01	0.9997779456	0.1509198318	0.2737033646	0.84915316	8.607017e-03	0.912276671	0.9982883	2.451360e-03	0.9757512606
0.77869908	1.434082e-01	2.355349e-01	0.9997779456	0.0046590276	0.9824312114	0.99380888	1.303930e-01	0.427416059	0.9982883	1.921688e-01	0.4438937237
0.90298719	5.532528e-02	3.800013e-01	0.9997779456	-0.1722209691	0.5577801518	0.91608209	-2.027724e-01	0.203698400	0.9982883	-9.502236e-02	0.4548086479
0.79799173	-1.809831e-01	6.014649e-01	0.9997779456	0.3198575202	0.6875827440	0.94482995	4.612402e-01	0.514651373	0.9982883	7.289599e-01	0.3326490653
0.99018039	1.094238e-01	1.710371e-01	0.9997779456	0.4012506932	0.1038264365	0.83807199	1.674706e-01	0.173994356	0.9982883	5.679259e-02	0.5976178350
0.97522657	1.032314e-01	2.638231e-01	0.9997779456	0.2845299234	0.2493988860	0.84466901	1.637026e-01	0.200549565	0.9982883	1.749347e-01	0.2375174798
0.97522657	-1.023243e-01	7.397037e-01	0.9997779456	0.0330761280	0.8665178573	0.96707132	1.439793e-03	0.992457365	0.9988193	-1.280207e-01	0.5515988972
0.96804147	1.619095e-01	1.159430e-01	0.9997779456	0.2667922978	0.0693996583	0.83807199	7.762872e-02	0.401224063	0.9982883	9.865842e-02	0.5424110139
0.93738259	4.055539e-02	3.535712e-01	0.9997779456	0.1382799976	0.2820611441	0.84915316	3.722006e-02	0.687805936	0.9982883	5.096308e-02	0.6283997270
0.92890486	1.515107e-01	2.415923e-01	0.9997779456	-0.3076881359	0.2182827174	0.83807199	-3.341484e-01	0.104097722	0.9982883	-9.671404e-02	0.6052009456
0.77869908	2.973185e-02	3.319133e-01	0.9997779456	-0.2594643290	0.0125913432	0.47899830	-2.029487e-02	0.757701159	0.9982883	1.427770e-01	0.1029782639
0.97273515	-1.084915e-01	7.044113e-01	0.9997779456	0.1792769248	0.5325550872	0.90627295	-1.513232e-02	0.938036824	0.9986411	7.631616e-02	0.7119920085
0.88581308	2.657061e-02	4.368678e-01	0.9997779456	-0.0518436189	0.7950483470	0.95972154	8.270196e-02	0.563475228	0.9982883	7.375505e-02	0.7258001880
0.97522657	-1.751352e-01	8.121701e-01	0.9997779456	-0.0068240775	0.9845605154	0.99380888	1.580664e-01	0.365785607	0.9982883	-3.489309e-01	0.2741030255
0.83237211	-1.709427e-01	8.141790e-01	0.9997779456	-0.1080921649	0.6206090212	0.93342431	8.869607e-02	0.625425067	0.9982883	-1.558682e-01	0.5179931567
0.92840513	1.303851e-01	1.539240e-01	0.9997779456	0.1388588237	0.4174932827	0.89856301	-6.612459e-02	0.552652888	0.9982883	-2.568270e-03	0.9812970025

0.81552863	-2.075556e-01	8.328385e-01	0.9997779456	-0.1661182147	0.5615328030	0.91675659	-2.697330e-01	0.188648794	0.9982883	3.763072e-02	0.8886276817
0.97273515	-4.650185e-02	6.420365e-01	0.9997779456	-0.1287589955	0.3630188968	0.87404187	-1.182354e-01	0.219939375	0.9982883	7.846931e-03	0.9599491378
0.98996967	-8.612865e-03	5.191176e-01	0.9997779456	0.3983585544	0.2979531432	0.84915316	2.548764e-01	0.228440366	0.9982883	-4.215171e-01	0.0386482624
0.98996967	-3.254818e-01	8.406147e-01	0.9997779456	0.2416129679	0.7494076193	0.95349818	-3.355431e-01	0.259782556	0.9982883	-5.606755e-02	0.8921494650
0.90581107	-7.936893e-02	9.337175e-01	0.9997779456	-0.0707773033	0.4277801264	0.90454000	7.157011e-02	0.233240150	0.9982883	5.188896e-02	0.5213495486
0.77869908	-9.283293e-02	8.328748e-01	0.9997779456	-0.1580621963	0.0796978836	0.83807199	-1.666232e-01	0.066638673	0.9982883	1.246972e-01	0.3755261092
0.97273515	-9.370499e-02	6.697901e-01	0.9997779456	0.1866156739	0.7014620981	0.94482995	-3.377672e-01	0.068263382	0.9982883	-2.772641e-01	0.0663585148
0.90581107	3.701070e-01	1.871836e-01	0.9997779456	0.5519730378	0.1461129958	0.83807199	2.574378e-01	0.562500823	0.9982883	-2.609233e-01	0.6234693521
0.80653217	-1.370794e-01	9.673274e-01	0.9997779456	-0.0729798467	0.4511630648	0.90612020	5.432777e-02	0.484613212	0.9982883	8.405888e-03	0.9423400159
0.96804147	-2.637059e-01	6.725480e-01	0.9997779456	-0.2976472230	0.5248415237	0.90612020	2.218903e-01	0.502220418	0.9982883	1.814693e-02	0.9686775502
0.80653217	3.928583e-01	9.584045e-02	0.9997779456	0.0655066706	0.8870034161	0.97235837	4.707482e-02	0.878623524	0.9982883	-1.431897e-01	0.6376765225
0.87292023	2.536570e-01	2.541824e-01	0.9997779456	0.8437132513	0.1783649513	0.83807199	2.482302e-01	0.569209973	0.9982883	-1.523044e-01	0.7347813119
0.77869908	3.591287e-01	2.608513e-01	0.9997779456	0.2914282174	0.5654248384	0.91973112	1.066938e-01	0.854842881	0.9982883	-3.735908e-01	0.4616611339
0.80868047	-5.036350e-01	6.967317e-01	0.9997779456	2.0232670256	0.2506346082	0.84467092	9.939615e-01	0.320766637	0.9982883	-2.356560e+00	0.0271062222
0.87292023	-2.953838e-01	8.980354e-01	0.9997779456	-0.6361459546	0.0003384800	0.09450935	-1.230540e-01	0.522739404	0.9982883	1.347545e-01	0.5079209318
0.84824569	-2.357979e-01	9.260647e-01	0.9997779456	-0.0419302806	0.8065141532	0.95972154	7.263670e-02	0.527961096	0.9982883	-5.017760e-01	0.0032459175
0.81552863	1.898725e-01	3.188626e-01	0.9997779456	0.4983120474	0.2454278156	0.84413548	3.679517e-02	0.924401295	0.9982883	-1.608754e-01	0.6984797278
0.82743594	3.980941e-01	1.175098e-01	0.9997779456	0.4532928302	0.3503864720	0.86676800	3.858021e-01	0.261163461	0.9982883	4.282479e-01	0.3966938762
0.86909435	-1.141916e-01	6.954959e-01	0.9997779456	-0.0824662151	0.7095656401	0.94482995	9.786665e-02	0.580042488	0.9982883	1.010152e-01	0.6343686539
0.77869908	3.730006e-01	1.659735e-01	0.9997779456	-0.3843775924	0.3358541462	0.85880449	-6.636845e-01	0.300407724	0.9982883	-4.630596e-01	0.2298768055
0.93430439	3.508183e-01	2.017838e-03	0.5962712336	0.4561072883	0.0308621449	0.66532795	1.059504e-01	0.423364345	0.9982883	-5.627426e-03	0.9736970110
0.99018039	1.853072e-02	4.371534e-01	0.9997779456	0.1794926803	0.1297949432	0.83807199	2.527019e-02	0.814662319	0.9982883	1.899106e-01	0.2443146982
0.99066584	-1.293762e-01	8.256471e-01	0.9997779456	-0.0647118263	0.7392071555	0.94973704	6.976161e-02	0.541828403	0.9982883	-1.467035e-01	0.4270815902
0.96416435	4.413186e-02	3.483560e-01	0.9997779456	0.1011976401	0.5712035961	0.92122660	8.281974e-02	0.427270794	0.9982883	-1.350429e-02	0.9056983225
0.97273515	-2.251067e-01	6.575312e-01	0.9997779456	0.1894720893	0.7924483948	0.95972154	9.035617e-02	0.823681476	0.9982883	-3.952426e-01	0.2326582283
0.81804251	2.049241e-01	1.328815e-01	0.9997779456	0.1266468531	0.6569776791	0.93854411	1.179298e-01	0.454662598	0.9982883	1.036836e-01	0.5582708026
0.99066584	-1.451365e-01	8.860289e-01	0.9997779456	-0.0348853937	0.7788629574	0.95657490	-1.138739e-01	0.209151126	0.9982883	-7.277745e-02	0.5295425321
0.92876201	-1.818767e-01	6.453686e-01	0.9997779456	0.0891842558	0.9048296387	0.97582904	-7.602783e-02	0.863718578	0.9982883	-2.806808e-02	0.9450840895
0.97522657	8.999265e-02	3.671228e-01	0.9997779456	0.5131558791	0.2421808441	0.84413548	2.923104e-01	0.244533292	0.9982883	-1.158754e-01	0.7021426460
0.81552863	1.915745e-01	2.716561e-01	0.9997779456	0.6231930835	0.2074678271	0.83807199	-4.287203e-02	0.869664815	0.9982883	2.189169e-01	0.5745294266
0.98146689	-3.299390e-01	9.954481e-01	0.9997779456	-0.0550305694	0.6878376666	0.94482995	-5.634445e-02	0.633210814	0.9982883	-2.743399e-01	0.0178950603
0.89512661	-1.928181e-01	9.150015e-01	0.9997779456	-0.0817717009	0.6111937456	0.93042774	1.094426e-01	0.399464960	0.9982883	-1.778956e-01	0.3451154780
0.97273515	1.156251e-01	2.321507e-01	0.9997779456	0.1863061351	0.2976871590	0.84915316	-4.003435e-02	0.730092979	0.9982883	1.312500e-02	0.9077867926
0.97522657	8.047310e-03	4.681680e-01	0.9997779456	0.2305801281	0.2031445718	0.83807199	-1.785162e-03	0.985541846	0.9986411	-4.196435e-02	0.6633525252
0.88581308	-4.233000e-01	6.358874e-01	0.9997779456	0.3277074389	0.7133768547	0.94482995	4.990493e-01	0.567770118	0.9982883	3.601969e-01	0.6895718132
0.87292023	4.201801e-02	3.947729e-01	0.9997779456	-0.0732804471	0.4852000874	0.90612020	1.999572e-01	0.064848225	0.9982883	1.557819e-01	0.3982119499
0.97273515	-3.092547e-01	9.453328e-01	0.9997779456	0.2022475978	0.1859686921	0.83807199	5.430782e-02	0.702355091	0.9982883	-2.745923e-01	0.0479981137
0.93688506	-2.916232e-02	5.521004e-01	0.9997779456	-0.1218795185	0.7306124402	0.94712279	-3.321393e-01	0.124499422	0.9982883	-1.529325e-01	0.5682358118
0.80820047	-1.730063e-01	8.263208e-01	0.9997779456	0.0597899754	0.7051798375	0.94482995	3.448127e-02	0.836598535	0.9982883	-3.112294e-01	0.1648208365
0.90482806	-5.487452e-01	9.995351e-01	0.9997779456	-0.3479548898	0.0303651638	0.66532795	-1.413158e-03	0.993275076	0.9988193	-2.460915e-01	0.2296482932
0.94189574	-1.883616e-01	9.305688e-01	0.9997779456	-0.0237377075	0.8626077420	0.96707132	-3.181741e-02	0.755998351	0.9982883	-4.046866e-01	0.0003856057
0.80868047	-1.662028e-01	7.315176e-01	0.9997779456	-1.0939860306	0.0085352423	0.43863723	-1.732334e-01	0.381747162	0.9982883	2.284446e-01	0.4135446741
0.80653217	2.265272e-01	1.908664e-01	0.9997779456	0.0877977098	0.7460949176	0.95235874	6.444372e-02	0.778587975	0.9982883	2.287771e-02	0.9359496314
0.96804147	-1.388237e-01	9.066330e-01	0.9997779456	0.1288402722	0.2800913993	0.84915316	8.376170e-02	0.417629827	0.9982883	2.328455e-02	0.8586267376
0.86278674	-3.973853e-02	5.765159e-01	0.9997779456	-0.0779178828	0.7441362304	0.95148651	1.063780e-01	0.531623498	0.9982883	1.887136e-01	0.4313071601
0.89512661	6.016032e-02	2.928084e-01	0.9997779456	0.1809343454	0.0741160486	0.83807199	1.028756e-01	0.276255397	0.9982883	-4.989138e-02	0.6624548485
0.92226585	-3.275999e-02	5.484991e-01	0.9997779456	0.4822830101	0.2421368378	0.84413548	-7.779077e-03	0.970960417	0.9986411	-2.833944e-01	0.3049232655
0.95284371	-1.151478e-01	8.757144e-01	0.9997779456	-0.1779546869	0.2215385097	0.83807199	-7.169486e-02	0.421254165	0.9982883	2.362675e-02	0.8332194482
0.97273515	-1.160847e-01	8.797944e-01	0.9997779456	-0.0261375897	0.8216905219	0.95972154	4.692823e-03	0.955355686	0.9986411	-1.679961e-02	0.8871494547
0.77869908	-6.598862e-02	6.289198e-01	0.9997779456	0.1158963455	0.4948396269	0.90612020	3.505754e-01	0.032362234	0.9982883	-3.088690e-01	0.1654702159
0.77869908	-1.725054e-02	5.496627e-01	0.9997779456	-0.4416843332	0.0106797003	0.46180258	-8.749423e-02	0.404798920	0.9982883	-1.634845e-02	0.8678509576
0.81163165	-4.287802e-02	7.222787e-01	0.9997779456	-0.1458997409	0.2000759378	0.83807199	-2.695844e-02	0.655856810	0.9982883	-7.337310e-02	0.4186421156
0.80985926	5.871410e-02	2.452503e-01	0.9997779456	0.0294950609	0.7781634982	0.95657490	9.842332e-02	0.237966913	0.9982883	1.473331e-01	0.0480859882
0.97273515	5.065678e-02	3.724376e-01	0.9997779456	-0.1408579494	0.2634195832	0.84915316	1.184724e-01	0.370620366	0.9982883	4.023420e-02	0.8393079303
0.95284371	1.882265e-01	1.011795e-01	0.9997779456	0.2312758696	0.1873346374	0.83807199	1.107778e-01	0.290729540	0.9982883	-5.859170e-02	0.5456208150
0.79972808	-9.532262e-03	5.107824e-01	0.9997779456	0.5656798865	0.2595809200	0.84915316	3.037879e-01	0.229262731	0.9982883	1.323045e-01	0.6629659837
0.77869908	-1.009051e-02	5.431581e-01	0.9997779456	-0.0425138874	0.7066825794	0.94482995	-9.815405e-02	0.255999301	0.9982883	1.371924e-02	0.8629719943
0.99062247	2.286455e-01	1.397207e-01	0.9997779456	0.4784908340	0.0809112156	0.83807199	1.570275e-01	0.358144623	0.9982883	-7.699342e-02	0.6389125268
0.99066584	-2.210037e-01	9.768471e-01	0.9997779456	0.1286317747	0.4675624335	0.90612020	-2.216013e-02	0.820267714	0.9982883	-1.806260e-01	0.1687396941
0.97273515	-5.311088e-02	7.031542e-01	0.9997779456	0.2225497384	0.1526270258	0.83807199	2.076942e-02	0.804466837	0.9982883	1.608396e-02	0.9179454564
0.95353311	-3.867045e-01	7.651418e-01	0.9997779456	-0.1462879642	0.6886332997	0.94482995	1.581896e-02	0.968250805	0.9986411	-3.756375e-01	0.3266715178
0.97273515	-4.345023e-02	6.179183e-01	0.9997779456	-0.0088784154	0.9532793723	0.99275438	5.760502e-02	0.621096424	0.9982883	-8.580354e-02	0.6888548723
0.77869908	-5.673926e-02	7.745985e-01	0.9997779456	-0.1111481966	0.0797987101	0.83807199	1.403100e-02	0.829746283	0.9982883	1.202664e-01	0.1499990503
0.82743594	5.214972e-02	3.316454e-01	0.9997779456	-0.0120018699	0.9565299988	0.99380888	-4.859649e-02	0.661931540	0.9982883	-1.856375e-01	0.0565353574
0.79578964	1.564134e-01	1.563232e-01	0.9997779456	-0.1133297762	0.5341540650	0.90627295	-1.788986e-01	0.222186467	0.9982883	3.584974e-01	0.1214190758
0.77869908	1.501524e-01	6.679963e-02	0.9997779456	0.0440849779	0.7200677973	0.94712279	3.925694e-02	0.699795256	0.9982883	1.112579e-01	0.1722450915
0.80820047	2.310545e-02	4.255728e-01	0.9997779456	-0.1355757755	0.2629642797	0.84915316	-2.872890e-02	0.793882603	0.9982883	5.297606e-02	0.7632819853
0.80820047	5.120444e-02	2.270143e-01	0.9997779456	0.0293498908	0.8158053844	0.95972154	1.121782e-01	0.095553227	0.9982883	3.898852e-02	0.6449668950
0.90482806	5.614470e-02	2.633016e-01	0.9997779456	0.0521233288	0.4856444069	0.90612020	-9.951464e-02	0.203500484	0.9982883	1.922351e-01	0.0747091790
0.93081046	-1.068320e-01	9.232059e-01	0.9997779456	-0.1127336347	0.3457147143	0.86676800	4.766268e-02	0.489160846	0.9982883	-5.905225e-02	0.6478712569
0.87292023	-3.695818e-01	7.368361e-01	0.9997779456	-0.2707502917	0.7058321711	0.94482995	-1.029488e+00	0.070081484	0.9982883	1.703349e-01	0.7738778634
0.82743594	-3.576574e-02	6.402959e-01	0.9997779456	0.0018575289	0.9884423329	0.99380888	1.227070e-02	0.891540472	0.9982883	1.396704e-01	0.2125964128
0.84315681	1.910189e-01	1.219970e-01	0.9997779456	-0.2534379108	0.1518819939	0.83807199	-1.470906e-01	0.410513331	0.9982883	-4.041633e-02	0.7919024948
0.97273515	-1.023739e+00	9.052337e-01	0.9997779456	0.0305468795	0.9384788793	0.98648257	3.578610e-01	0.389252810	0.9982883	-5.353966e-01	0.1783834544
0.83140044	8.746870e-02	4.211094e-01	0.9997779456	0.6272352969	0.2437189828	0.84413548	1.852766e-01	0.676239691	0.9982883	-2.012827e-01	0.6808280105
0.99066584	2.889370e-02	3.859685e-01	0.9997779456	0.3727028872	0.0286783711	0.66466342	3.231318e-02	0.725743660	0.9982883	-1.039452e-01	0.4554660429
0.77869908	1.321029e-02	4.430923e-01	0.9997779456	0.0428092611	0.6704326564	0.94130886	1.283510e-01	0.086058602	0.9982883	4.716879e-02	0.6173377627
0.82862325	2.247541e-02	3.892285e-01	0.9997779456	-0.1433984118	0.0536182926	0.83664873	3.022138e-02	0.713723313	0.9982883	-3.528847e-02	0.6865012548
0.77869908	8.489421e-02	3.637317e-01	0.9997779456	0.0503355647	0.7831318097	0.95657490	-5.132103e-02	0.774706573	0.9982883	2.461860e-01	0.3999758332
0.79972808	1.382301e-02	4.780063e-01	0.9997779456	0.3315412797	0.2718061534	0.84915316	1.592609e-01	0.435956636	0.9982883	2.332493e-01	0.3563554657
0.97273515	5.643319e-02	3.126141e-01	0.9997779456	-0.0422551535	0.7721686932	0.95505354	-4.858230e-03	0.955593005	0.9986411	1.548889e-02	0.9202638134
0.94675587	6.391584e-02	3.055673e-01	0.9997779456	-0.0515512623	0.7791175983	0.95657490	-1.392155e-01	0.335258061	0.9982883	-9.176948e-02	0.6634995156
0.97050390	4.883050e-01	2.153345e-02	0.9997779456	0.8685043455	0.0056503770	0.40915301	3.557993e-01	0.124115912	0.9982883	2.338848e-01	0.4193772697
0.92501596	-1.277158e-01	8.496439e-01	0.9997779456	-0.0408304854	0.7664094290	0.95505354	-1.478828e-02	0.882126605	0.9982883	-1.611890e-01	0.2483246000
0.99066584	-1.317856e-04	5.002651e-01	0.9997779456	0.0629005402	0.5530977940	0.91608209	-2.354348e-02	0.853671798	0.9982883	-2.396558e-01	0.0314826698
0.97273515	1.445516e-01	5.014807e-02	0.9997779456	0.1058818271	0.4352690709	0.90612020	3.549173e-02	0.612528862	0.9982883	-2.063830e-02	0.7492829800
0.77869908	4.329173e-01	7.047489e-02	0.9997779456	0.3469753356	0.3226000048	0.85589812	3.313825e-01	0.240091178	0.9982883	1.256074e-01	0.7401557098
0.89512661	-6.146312e-02	6.323647e-01	0.9997779456	-0.2119321905	0.2485271985	0.84413548	-1.845326e-02	0.907203425	0.9982883	2.795611e-01	0.2716196718
0.99066584	4.366686e-02	2.777296e-01	0.9997779456	0.3044952616	0.0534828712	0.83664873	8.834175e-02	0.184283571	0.9982883	7.523130e-02	0.4410904658
0.87099999	-2.675827e-01	9.550413e-01	0.9997779456	-0.0417615455	0.7078155865	0.94482995	-2.847501e-04	0.998726450	0.9989813	-4.337629e-01	0.0017527001
0.77869908	1.562149e-01	3.784889e-01	0.9997779456	0.3508554700	0.5228739435	0.90612020	2.126029e-01	0.707201748	0.9982883	2.917044e-02	0.9568278335
0.90581107	1.441313e-01	2.648752e-01	0.9997779456	-0.3545568465	0.3249939083	0.85614181	2.109931e-01	0.409742170	0.9982883	1.511866e-01	0.6074688120
0.90802583	-5.534487e-01	7.497382e-01	0.9997779456	-0.7435010673	0.0924334303	0.83807199	6.884901e-01	0.170003021	0.9982883	-4.656660e-01	0.2520709974
0.95472383	1.228164e-01	2.088345e-01	0.9997779456	-0.1536123525	0.5206008121	0.90612020	1.044023e-01	0.485648834	0.9982883	-1.401220e-01	0.5716659539
0.77869908	1.684151e-01	1.077545e-01	0.9997779456	0.0057629999	0.9766178617	0.99380888	1.047144e-01	0.415934463	0.9982883	1.910632e-01	0.3295620182
0.97273515	-7.733256e-02	5.973451e-01	0.9997779456	0.3078355397	0.5046986686	0.90612020	3.422866e-01	0.221000005	0.9982883	3.221089e-02	0.9078095716
0.87292023	-2.843720e-01	9.257919e-01	0.9997779456	0.0174284364	0.9339784522	0.98590149	-2.008944e-01	0.301619125	0.9982883	8.161539e-02	0.6730397934
0.77869908	8.971868e-02	2.178642e-01	0.9997779456	0.0910596867	0.5251775238	0.90612020	3.353211e-02	0.731476330	0.9982883	-3.677694e-02	0.7966749437
0.99066584	6.363735e-02	3.877851e-01	0.9997779456	0.1200253089	0.6684656699	0.94130886	-7.667760e-02	0.730521544	0.9982883	-2.294708e-01	0.4752834619
0.87292023	-5.591389e-02	7.255836e-01	0.9997779456	-0.0159780569	0.9151105073	0.98058109	8.217355e-02	0.434857903	0.9982883	1.479494e-01	0.2620253467
0.93650941	1.100777e-01	2.765307e-01	0.9997779456	0.2437567308	0.3049271177	0.84915316	2.643129e-01	0.096586456	0.9982883	-7.582364e-02	0.7234798634
0.94675587	-6.535047e-01	9.672141e-01	0.9997779456	-0.1672598810	0.5799974277	0.92532140	-1.167444e-01	0.648163921	0.9982883	1.366972e-01	0.7572716019
0.96471659	-3.123138e-02	6.279053e-01	0.9997779456	-0.0334227102	0.7699645218	0.95505354	3.518975e-02	0.706775173	0.9982883	2.075731e-02	0.7905096680
0.97273515	-2.614423e-02	5.406145e-01	0.9997779456	-0.0713596423	0.7287046515	0.94712279	-9.648439e-02	0.659575657	0.9982883	2.821559e-01	0.4933717636
0.99436660	-1.494966e-01	9.164231e-01	0.9997779456	0.1504191353	0.4333895608	0.90612020	3.472089e-02	0.707363161	0.9982883	-9.782209e-02	0.3055944988
0.86909435	-6.467156e-02	7.712471e-01	0.9997779456	-0.1746928608	0.0903109960	0.83807199	2.364580e-02	0.745972275	0.9982883	-3.175881e-02	0.7810730753
0.77869908	8.992942e-02	2.276202e-01	0.9997779456	-0.2410917757	0.1293040999	0.83807199	-8.290265e-02	0.461804865	0.9982883	3.224934e-02	0.7711895125
0.98996967	-3.103313e-01	8.760364e-01	0.9997779456	-0.0342408605	0.8770920406	0.96947881	-1.005599e-02	0.959732733	0.9986411	-3.045914e-01	0.0994508285
0.97522657	-2.394359e-01	7.936625e-01	0.9997779456	0.1479857159	0.4972878112	0.90612020	6.601818e-04	0.997129232	0.9988193	-2.187724e-01	0.1435225971
0.99066584	-2.863144e-01	8.159962e-01	0.9997779456	-0.0989481489	0.7056384883	0.94482995	1.470048e-01	0.500060771	0.9982883	-8.910208e-02	0.7922978436
0.80820047	1.276516e-01	2.575503e-01	0.9997779456	0.2166344500	0.5047959652	0.90612020	3.231246e-01	0.060103441	0.9982883	1.846115e-01	0.6150078063
0.77869908	-6.270659e-02	7.502281e-01	0.9997779456	0.0192024075	0.8431886769	0.96338860	-6.238917e-02	0.496193058	0.9982883	-7.050098e-02	0.4132049475
0.97273515	4.128560e-01	3.268510e-01	0.9997779456	2.1645709272	0.3689208146	0.87664366	1.095651e+00	0.298783473	0.9982883	-1.072679e+00	0.2425391285
0.97273515	-5.694152e-01	8.447706e-01	0.9997779456	0.9541396570	0.4660801208	0.90612020	6.400280e-01	0.293092046	0.9982883	-6.752293e-01	0.2203785120
0.77869908	-1.029988e-01	8.324207e-01	0.9997779456	-0.0537689159	0.4815765387	0.90612020	1.493702e-01	0.089316854	0.9982883	3.112345e-02	0.7833116058
0.77869908	-7.320130e-02	6.363962e-01	0.9997779456	0.2222502604	0.2807038550	0.84915316	1.027073e-01	0.602365896	0.9982883	-4.022739e-01	0.1446262709
0.97975724	-3.630135e-01	9.452787e-01	0.9997779456	0.0149418322	0.9582575351	0.99380888	-1.244639e-01	0.549125676	0.9982883	-2.179544e-01	0.4413323881
0.83725376	4.944722e-01	2.268241e-01	0.9997779456	0.6161415527	0.3389273361	0.85925321	-1.953583e-01	0.733897367	0.9982883	-4.238379e-01	0.5243310269
0.84600172	-2.662691e-01	9.781331e-01	0.9997779456	0.0041592657	0.9731256929	0.99380888	-3.048106e-02	0.794333219	0.9982883	-8.203586e-02	0.6747409621
0.82862325	1.694439e-01	2.824000e-01	0.9997779456	-0.7976722601	0.0273576049	0.64673378	-2.702395e-01	0.356115141	0.9982883	8.384525e-01	0.1862633885
0.81552863	-1.450319e-01	7.831228e-01	0.9997779456	0.0517684095	0.8272022391	0.95988696	-2.113814e-01	0.206129651	0.9982883	-3.424810e-01	0.1140894839
0.88636459	-1.353787e-01	7.977212e-01	0.9997779456	0.2795472909	0.1550847861	0.83807199	1.344301e-01	0.329915621	0.9982883	-3.149844e-01	0.0772802873
0.93651489	-2.940983e-01	9.290406e-01	0.9997779456	-0.1382694848	0.6786613187	0.94316618	-7.755121e-02	0.658285572	0.9982883	-3.317261e-01	0.0360570325
0.97273515	-4.790737e-03	5.269902e-01	0.9997779456	-0.0432661510	0.6779811958	0.94316618	-7.040164e-02	0.235118044	0.9982883	-8.323680e-02	0.4090943125
0.98996967	3.962021e-02	4.532305e-01	0.9997779456	0.2304493601	0.4470023367	0.90612020	-1.330087e-01	0.627468533	0.9982883	3.901058e-01	0.2690679085
0.90482806	6.059816e-02	3.549809e-01	0.9997779456	-0.1773356416	0.2463538428	0.84413548	1.874179e-01	0.187454378	0.9982883	1.729995e-01	0.5080227206
0.98996967	-6.921274e-02	8.097125e-01	0.9997779456	-0.0303450187	0.7511205542	0.95464999	4.157887e-02	0.501023095	0.9982883	-1.054977e-01	0.3006661824
0.86909435	-1.570573e-01	8.445837e-01	0.9997779456	0.0214737212	0.8782998501	0.96947881	8.490169e-02	0.516719132	0.9982883	-7.261353e-02	0.6222047103
0.81163165	-1.703477e-01	9.468998e-01	0.9997779456	-0.0850404722	0.6426976634	0.93854411	1.882078e-02	0.844893133	0.9982883	-1.776875e-01	0.1781100169
0.86909435	2.661032e-01	1.058414e-01	0.9997779456	0.5957976920	0.1074389137	0.83807199	-8.326625e-03	0.970939551	0.9986411	-1.349521e-01	0.5715682905
0.77869908	-1.160600e-01	9.066169e-01	0.9997779456	-0.0984363538	0.3591228265	0.87404187	-7.682947e-05	0.998981340	0.9989813	-5.940538e-02	0.5095890083
0.92890486	-1.129428e-02	5.108274e-01	0.9997779456	-0.1316589741	0.7109852215	0.94482995	5.425141e-01	0.114750332	0.9982883	4.433363e-01	0.4803404135
0.99066584	1.712392e-02	4.261242e-01	0.9997779456	0.2169458577	0.1487406566	0.83807199	1.096870e-02	0.887815870	0.9982883	5.394661e-03	0.9605036339
0.86909435	9.593347e-02	3.381449e-01	0.9997779456	0.5863215696	0.0887484304	0.83807199	1.050039e-01	0.588736838	0.9982883	2.275333e-02	0.9411842606
0.77869908	1.146617e-02	4.616804e-01	0.9997779456	-0.1836313996	0.0474447880	0.83664873	3.755414e-02	0.652311248	0.9982883	2.818409e-02	0.7649446747
0.77869908	2.323334e-01	3.210792e-01	0.9997779456	0.4550002969	0.4196334867	0.89856301	1.786485e-02	0.975449931	0.9986411	-2.111080e-01	0.6997841360
0.81552863	5.416430e-02	4.664454e-01	0.9997779456	0.3587345448	0.4837085932	0.90612020	-7.613881e-02	0.911039635	0.9982883	-1.015990e-01	0.8392226866
0.81552863	-5.152678e-02	8.155859e-01	0.9997779456	0.0404562451	0.6168636592	0.93239494	2.327233e-02	0.689204170	0.9982883	-1.551820e-01	0.0418297935
0.98111638	-7.675745e-01	9.210751e-01	0.9997779456	-0.1599707198	0.6053868255	0.93042774	1.033751e-01	0.762292229	0.9982883	-5.993824e-01	0.0439411223

0.80820047	-2.292180e-01	6.452973e-01	0.9997779456	-0.6417649745	0.1848170353	0.83807199	1.886857e-03	0.996512195	0.9988193	6.071102e-01	0.2830971356
0.77869908	-9.037554e-04	5.037077e-01	0.9997779456	-0.2666948598	0.0588612635	0.83807199	8.296835e-03	0.935510324	0.9986411	-6.679855e-02	0.6155851564
0.81552863	2.822131e-01	2.717339e-01	0.9997779456	0.8525465045	0.2929674824	0.84915316	4.184420e-01	0.312450201	0.9982883	-5.239261e-02	0.9311431185
0.77869908	4.982487e-02	2.196426e-01	0.9997779456	-0.1916812141	0.0857695903	0.83807199	-1.085182e-01	0.104151776	0.9982883	1.280896e-02	0.9119113045
0.97522657	1.858962e-03	4.942400e-01	0.9997779456	-0.1403942072	0.2893052915	0.84915316	-4.678196e-02	0.669581659	0.9982883	-5.189509e-02	0.6794121660
0.95558280	-1.572363e-01	8.014591e-01	0.9997779456	-0.4897429485	0.1161579435	0.83807199	-1.597029e-01	0.429886853	0.9982883	3.312679e-01	0.1069105057
0.88636459	2.484453e-01	1.119043e-01	0.9997779456	0.0444766231	0.8857324966	0.97215753	2.305993e-01	0.221462213	0.9982883	8.490389e-03	0.9753100040
0.97273515	-1.732231e+00	7.887713e-01	0.9997779456	-0.3286759747	0.7803117391	0.95657490	1.842103e+00	0.227892325	0.9982883	-2.145486e+00	0.1621424114
0.87292023	-3.656410e-02	6.211200e-01	0.9997779456	-0.2340046568	0.1216415113	0.83807199	-2.549641e-03	0.981643275	0.9986411	5.498154e-02	0.7337418914
0.81804251	2.021911e-02	4.808995e-01	0.9997779456	0.2100111231	0.7048212400	0.94482995	-2.126555e-01	0.561495227	0.9982883	-5.524368e-01	0.2267702741
0.77869908	-2.541038e-01	7.840701e-01	0.9997779456	0.6676770761	0.0540758177	0.83664873	5.216080e-01	0.092485625	0.9982883	-6.389801e-01	0.1767978568
0.97185522	1.615905e-02	4.538254e-01	0.9997779456	0.4813295138	0.0812240425	0.83807199	1.400742e-01	0.309427387	0.9982883	-7.638411e-02	0.7491309757
0.97273515	6.820855e-01	1.150135e-01	0.9997779456	-0.3502556424	0.4931655710	0.90612020	-7.328379e-01	0.455569039	0.9982883	-4.167202e-01	0.3983428003
0.96804147	6.593691e-01	5.062850e-03	0.9997779456	-0.1054762127	0.7074165866	0.94482995	2.508969e-01	0.285728834	0.9982883	4.765603e-01	0.3272700520
0.77869908	-1.038048e-02	5.311448e-01	0.9997779456	-0.1458602081	0.3294289544	0.85754449	-1.641668e-01	0.249855015	0.9982883	4.360054e-02	0.7807651387
0.81163165	-6.315645e-01	9.839477e-01	0.9997779456	0.0082793539	0.9621477603	0.99380888	-8.423769e-02	0.752913964	0.9982883	-2.537687e-01	0.1947106911
0.77869908	-5.659942e-02	6.558751e-01	0.9997779456	-0.3940008967	0.0267743976	0.64586404	3.663558e-02	0.733924296	0.9982883	-1.941997e-01	0.1252413921
0.80820047	3.626846e-01	3.224328e-01	0.9997779456	0.9528222714	0.3021508387	0.84915316	5.410502e-01	0.497755223	0.9982883	4.517393e-01	0.6422514607
0.79578964	1.390937e-01	3.764516e-01	0.9997779456	0.2839878076	0.5373678768	0.90738404	1.928889e-01	0.597914567	0.9982883	-1.312915e-02	0.9758521076
0.98226159	-5.811723e-02	5.531156e-01	0.9997779456	0.7032053520	0.3771429915	0.87916124	2.003157e-01	0.673242626	0.9982883	-1.131905e-01	0.7945602965
0.77869908	-1.239261e-01	9.160274e-01	0.9997779456	-0.0521646577	0.5192301801	0.90612020	1.516045e-02	0.855520650	0.9982883	1.081510e-01	0.3237161371
0.98731161	6.857890e-02	2.793389e-01	0.9997779456	-0.0961801972	0.5518703959	0.91608209	-1.465911e-01	0.196968594	0.9982883	-1.564319e-01	0.3968662461
0.91950332	4.913636e-02	4.159262e-01	0.9997779456	0.2322601986	0.4887622762	0.90612020	3.686818e-02	0.889100779	0.9982883	-3.519796e-02	0.9189772181
0.77869908	1.226917e-01	2.947428e-01	0.9997779456	0.0857939608	0.6897151130	0.94482995	-2.372168e-01	0.229423725	0.9982883	2.463221e-02	0.9262820804
0.89512661	1.954169e+00	1.439461e-01	0.9997779456	4.9918894721	0.1788025726	0.83807199	4.413458e+00	0.131238123	0.9982883	-3.814867e+00	0.1368992754
0.85691049	-5.935495e-02	6.361093e-01	0.9997779456	0.0393109122	0.8196939593	0.95972154	-1.709105e-01	0.187197843	0.9982883	2.920592e-01	0.1614214414
0.97185522	-2.354022e-01	9.522244e-01	0.9997779456	-0.0545129583	0.7354959179	0.94712279	-1.333142e-01	0.237557258	0.9982883	-8.899465e-02	0.5236882194
0.77869908	2.235689e-02	4.098325e-01	0.9997779456	-0.1065511137	0.3621335491	0.87404187	-2.193012e-02	0.780771121	0.9982883	7.785024e-02	0.5244060038
0.89273364	9.620939e-01	1.042804e-01	0.9997779456	2.2482906004	0.2070706200	0.83807199	1.241969e+00	0.129518018	0.9982883	-9.106340e-01	0.2084964356
0.84600172	-9.095411e-02	8.156174e-01	0.9997779456	-0.0914652456	0.3864398376	0.87916124	1.303261e-01	0.169612978	0.9982883	2.310647e-02	0.8377821855
0.77869908	-1.289245e-01	7.721726e-01	0.9997779456	-0.4261471783	0.0004360209	0.09450935	-1.142257e-02	0.926121769	0.9982883	-8.843827e-03	0.9447020255
0.87099999	-1.446842e-01	7.689833e-01	0.9997779456	-0.2555566388	0.4585670348	0.90612020	-5.597600e-02	0.793613338	0.9982883	6.265696e-02	0.8144565605
0.77869908	-3.546956e-02	5.274104e-01	0.9997779456	0.3957861403	0.4619923514	0.90612020	1.730630e-01	0.720106925	0.9982883	-1.513726e-01	0.7737083193
0.97273515	-4.744627e-01	9.690055e-01	0.9997779456	0.0047336463	0.9856924117	0.99380888	1.161873e-01	0.545830592	0.9982883	-4.062860e-01	0.1107001503
0.77869908	-1.417336e-01	8.408729e-01	0.9997779456	-0.3115682286	0.0003257453	0.09450935	2.098810e-02	0.835734504	0.9982883	-4.808754e-02	0.7507102336
0.97522657	4.873172e-01	2.619002e-01	0.9997779456	1.7935242858	0.3797364343	0.87916124	6.404655e-01	0.434546528	0.9982883	-5.668549e-01	0.4043217308
0.88001839	-5.096838e-02	5.583684e-01	0.9997779456	0.7665293284	0.1105684043	0.83807199	4.213190e-01	0.162606074	0.9982883	4.678065e-02	0.9341969531
0.97051145	7.327051e-02	3.741291e-01	0.9997779456	-0.0965492692	0.5026520086	0.90612020	-1.580474e-01	0.442944198	0.9982883	2.727788e-01	0.2810133734
0.77869908	6.356788e-02	3.123020e-01	0.9997779456	-0.1275036781	0.3147009293	0.84915316	-6.485402e-02	0.509607295	0.9982883	6.945722e-02	0.6582862004
0.83725376	-1.570105e-01	8.473370e-01	0.9997779456	-0.0346586763	0.8951329210	0.97246977	-1.474228e-02	0.922880554	0.9982883	1.470422e-01	0.5299311068
0.91950332	6.467580e-06	4.999814e-01	0.9997779456	-0.0286000608	0.8599907619	0.96707132	-4.013276e-02	0.752617533	0.9982883	-1.118674e-02	0.9543431154
0.79213004	1.229421e-01	3.122896e-01	0.9997779456	0.4435426292	0.3259423142	0.85614181	1.822040e-01	0.375257671	0.9982883	1.615391e-01	0.7044407692
0.77869908	4.713828e-02	3.618968e-01	0.9997779456	0.2423736899	0.1605419339	0.83807199	1.277420e-01	0.190051796	0.9982883	1.860120e-01	0.3245039041
0.82862325	2.270246e-01	3.388321e-01	0.9997779456	0.1779831885	0.6649129240	0.94041014	-7.089193e-03	0.988727042	0.9986411	-1.193003e-01	0.8126192520
0.77869908	-1.715559e-02	5.255436e-01	0.9997779456	0.0631400673	0.8448162078	0.96338860	1.075424e-01	0.652774692	0.9982883	1.987025e-01	0.5504851159
0.77869908	-2.630175e-02	6.319522e-01	0.9997779456	0.0728434178	0.5277071512	0.90612020	5.703075e-02	0.360358484	0.9982883	-3.502346e-02	0.6701428069
0.97273515	-6.452473e-02	6.759300e-01	0.9997779456	0.0133977545	0.9460232027	0.98868207	-7.091476e-02	0.524917602	0.9982883	-9.874252e-03	0.9449524121
0.97050390	-5.807340e-02	6.187785e-01	0.9997779456	0.0834445390	0.6634623620	0.94041014	1.564751e-01	0.289262898	0.9982883	6.117049e-02	0.7446356089
0.98226159	4.357314e-01	2.169950e-01	0.9997779456	-1.3174571137	0.0071466681	0.42236808	-4.703182e-01	0.472302087	0.9982883	1.621791e-02	0.9823207281
0.80820047	1.790794e-01	2.367690e-01	0.9997779456	-0.2936689057	0.5292037912	0.90612020	8.174883e-02	0.750851315	0.9982883	3.111217e-01	0.3448031260
0.99066584	-2.011411e-01	9.344380e-01	0.9997779456	0.0397615381	0.7331346420	0.94712279	-2.887092e-02	0.816714271	0.9982883	-2.446615e-01	0.1081735912
0.83683629	-3.789122e-02	6.012113e-01	0.9997779456	0.0705230612	0.6673396811	0.94128342	5.392976e-03	0.973799001	0.9986411	9.269459e-02	0.5651892960
0.97273515	-6.784786e-02	6.650004e-01	0.9997779456	-0.2101616719	0.3803941615	0.87916124	-1.564025e-01	0.253077262	0.9982883	2.350699e-01	0.2687641442
0.81163165	-6.043996e-02	7.427228e-01	0.9997779456	-0.1401561883	0.1220003733	0.83807199	5.232491e-02	0.513646029	0.9982883	-1.772283e-02	0.8328587173
0.88001839	-4.735782e-02	7.172891e-01	0.9997779456	-0.0931470447	0.4102265245	0.89501894	8.303872e-03	0.924776463	0.9982883	4.545773e-02	0.5985063655
0.82862325	-2.087417e-01	8.887687e-01	0.9997779456	-0.0628729150	0.8243838742	0.95988696	1.598758e-01	0.350024200	0.9982883	-1.048582e-01	0.6373290247
0.92890486	1.022530e+00	2.410452e-02	0.9997779456	1.6187523706	0.1804789683	0.83807199	8.962126e-01	0.092932894	0.9982883	-8.433810e-01	0.0706620545
0.77869908	2.732511e-01	2.199537e-01	0.9997779456	0.5118028689	0.2979457202	0.84915316	2.417778e-01	0.508270783	0.9982883	1.701879e-01	0.7216482865
0.77869908	4.705783e-01	8.507960e-02	0.9997779456	-0.3426619372	0.3041451456	0.84915316	3.579762e-01	0.222695060	0.9982883	-1.953033e-01	0.5492546181
0.83725376	-7.216749e-02	7.574937e-01	0.9997779456	-0.2114675670	0.0733292103	0.83807199	-7.821152e-02	0.393919251	0.9982883	5.759799e-02	0.6150869967
0.77869908	2.471137e-02	3.744302e-01	0.9997779456	-0.0697647364	0.4261612549	0.90454000	2.820091e-02	0.644732606	0.9982883	7.258072e-02	0.3304811936
0.89273364	-1.409260e-02	5.347586e-01	0.9997779456	-0.0370266921	0.8360175355	0.96321135	1.328994e-01	0.378211903	0.9982883	1.522237e-01	0.4690931893
0.80820047	6.208807e-02	4.074221e-01	0.9997779456	0.2313903277	0.5744027138	0.92122660	1.692026e-01	0.497808592	0.9982883	1.882552e-01	0.5813863176
0.77869908	-7.327908e-03	5.228375e-01	0.9997779456	-0.0831810592	0.4920611759	0.90612020	-1.722317e-02	0.880974519	0.9982883	4.154533e-02	0.7395467027
0.82862325	-8.906707e-01	9.086409e-01	0.9997779456	0.0060254921	0.9861553253	0.99380888	4.465648e-01	0.241767045	0.9982883	-4.275621e-01	0.2722532982
0.97185522	1.639484e-01	2.112586e-01	0.9997779456	-0.3962790886	0.1480834973	0.83807199	8.396985e-02	0.668082436	0.9982883	1.963693e-01	0.4915406885
0.97273515	3.407610e-01	1.731238e-01	0.9997779456	1.0808656133	0.3528940108	0.87067296	3.715111e-01	0.341270496	0.9982883	-1.543148e-01	0.6196805759
0.82862325	-1.736959e-01	6.577281e-01	0.9997779456	0.0966650804	0.8204558502	0.95972154	9.797241e-02	0.770192603	0.9982883	2.754644e-01	0.5590209127
0.77869908	-4.935286e-02	7.199107e-01	0.9997779456	-0.2449478857	0.0493295328	0.83664873	-2.861223e-02	0.639167578	0.9982883	6.799485e-02	0.4789515613
0.77869908	2.871485e-02	4.664182e-01	0.9997779456	0.1098517024	0.7303986801	0.94712279	8.221307e-02	0.790268801	0.9982883	7.727750e-01	0.1373068890
0.77869908	1.203793e-01	1.470246e-01	0.9997779456	-0.1004697384	0.3485431851	0.86676800	-6.036443e-02	0.584524499	0.9982883	-8.055714e-02	0.4658779793
0.89369078	1.504528e-02	4.476266e-01	0.9997779456	-0.1000748190	0.5580195470	0.91608209	-1.296593e-01	0.213124639	0.9982883	1.232005e-01	0.4476898229
0.79578964	1.494567e-01	1.718650e-02	0.9997779456	0.0480253847	0.4994274858	0.90612020	-3.171999e-02	0.660994425	0.9982883	8.138422e-02	0.3728498077
0.77869908	1.735671e-01	3.948194e-01	0.9997779456	0.1550609767	0.8487316684	0.96338860	-3.168712e-01	0.623249383	0.9982883	-7.042777e-01	0.1369330971
0.81163165	-2.628336e-01	9.437000e-01	0.9997779456	-0.1520170197	0.2478631705	0.84413548	1.019863e-01	0.507441752	0.9982883	2.618242e-01	0.1730657627
0.91967042	-3.086652e-01	6.485958e-01	0.9997779456	-1.5415155663	0.0156300466	0.49931662	7.836389e-01	0.229035578	0.9982883	6.439224e-01	0.5987348500
0.82862325	-1.403343e-01	9.239238e-01	0.9997779456	-0.0629308121	0.5538200280	0.91608209	-5.581740e-02	0.446454151	0.9982883	-5.288811e-02	0.5664806274
0.77869908	-1.121268e-01	8.638943e-01	0.9997779456	-0.0223086360	0.8551859230	0.96707132	9.453387e-03	0.916939203	0.9982883	-5.244905e-03	0.9575895741
0.98299735	-7.948516e-01	9.739237e-01	0.9997779456	-0.3096042251	0.2659707592	0.84915316	-2.342265e-01	0.346076991	0.9982883	-3.717527e-01	0.1598922750
0.99018039	-1.137873e-01	5.987815e-01	0.9997779456	0.1719900978	0.7528560417	0.95505354	1.133179e-01	0.781110512	0.9982883	4.457877e-01	0.3817410822
0.97273515	-3.257190e-02	6.066686e-01	0.9997779456	0.0104332049	0.9556786691	0.99380888	-1.159820e-01	0.314393271	0.9982883	-2.733374e-01	0.0866186570
0.97522657	-6.489045e-02	6.107837e-01	0.9997779456	0.1720484572	0.6885040116	0.94482995	-2.435170e-02	0.896770161	0.9982883	-1.268892e-02	0.9584427102
0.77869908	1.533094e-01	4.030796e-01	0.9997779456	0.7514951733	0.3182528347	0.84915316	1.604664e-01	0.796276622	0.9982883	-1.038757e-01	0.8661543918
0.80820047	1.024209e-02	4.454791e-01	0.9997779456	0.0484103435	0.5613968463	0.91675659	-5.161933e-02	0.494648197	0.9982883	-1.253357e-02	0.8944268447
0.88636459	1.465597e-01	3.103133e-01	0.9997779456	0.0311735070	0.9158726912	0.98058109	2.795380e-02	0.913310486	0.9982883	2.605773e-01	0.3780118228
0.77869908	-5.949122e-02	5.451422e-01	0.9997779456	0.6392187082	0.3593325577	0.87404187	1.471853e-01	0.762524167	0.9982883	-2.365133e-01	0.7085247346
0.95617257	-8.293380e-02	7.359649e-01	0.9997779456	-0.0757817824	0.4876279935	0.90612020	5.812824e-02	0.565578498	0.9982883	5.532038e-02	0.6186954856
0.95472383	-1.344933e-01	7.658697e-01	0.9997779456	0.0021956092	0.9899792846	0.99380888	9.131362e-02	0.530965290	0.9982883	-4.099198e-02	0.8075013668
0.99066584	-1.038438e-01	8.108704e-01	0.9997779456	0.2607069290	0.1239023824	0.83807199	-1.167063e-02	0.915808458	0.9982883	-7.069545e-02	0.5079454651
0.99018039	5.972581e-02	3.097777e-01	0.9997779456	0.3647521414	0.0533396214	0.83664873	6.265120e-02	0.540810034	0.9982883	-9.247799e-02	0.5163116888
0.77869908	7.827909e-01	3.320873e-02	0.9997779456	-0.1993278626	0.6359186817	0.93852299	-6.801764e-01	0.193044191	0.9982883	-7.612235e-01	0.1106907535
0.96804147	-2.353204e-02	5.572287e-01	0.9997779456	-0.3005344902	0.1769473925	0.83807199	-4.745507e-02	0.679735659	0.9982883	3.395779e-02	0.8453310224
0.97050390	1.703188e-03	4.921005e-01	0.9997779456	-0.0876471422	0.4326467531	0.90612020	-7.770439e-02	0.281278436	0.9982883	7.196627e-02	0.3523577637
0.94189574	3.223849e-01	6.042452e-02	0.9997779456	0.2826091662	0.3162135834	0.84915316	1.664475e-01	0.377164042	0.9982883	-7.469941e-02	0.7162360053
0.77869908	6.535540e-01	2.072019e-01	0.9997779456	0.6569636948	0.4868973870	0.90612020	6.693037e-01	0.411829820	0.9982883	3.173552e-01	0.7877808801
0.81552863	1.586684e-01	1.490248e-01	0.9997779456	0.1519175072	0.3394849830	0.85925321	1.484153e-01	0.252997466	0.9982883	1.759779e-01	0.5173452376
0.80820047	-1.787397e-01	9.851072e-01	0.9997779456	-0.1345398243	0.2478364166	0.84413548	3.281814e-02	0.658319397	0.9982883	-7.450664e-02	0.5704475039
0.77869908	-3.934408e-01	7.232906e-01	0.9997779456	-0.1136614844	0.8587430788	0.96707132	6.863956e-01	0.266669494	0.9982883	9.448916e-01	0.3749962827
0.79972808	-3.246651e-02	6.732593e-01	0.9997779456	-0.1013341195	0.2184092970	0.83807199	-3.800756e-02	0.570360174	0.9982883	-5.291150e-02	0.6134196872
0.99018039	2.230802e-01	4.329010e-02	0.9997779456	0.3938362564	0.1290760947	0.83807199	9.401011e-02	0.389581199	0.9982883	-1.294814e-01	0.2047563726
0.77869908	3.515667e-01	1.600358e-01	0.9997779456	-0.4305000398	0.2107030981	0.83807199	-1.979376e-01	0.557714801	0.9982883	9.559115e-01	0.3069723137
0.91967042	-2.439655e-01	9.977920e-01	0.9997779456	-0.1079139487	0.4271263054	0.90454000	1.844055e-02	0.820968312	0.9982883	-6.038169e-02	0.6152090696
0.77869908	5.848149e-01	1.673586e-01	0.9997779456	0.2580681718	0.7962360406	0.95972154	-2.023345e-02	0.972715978	0.9986411	-4.797668e-01	0.4652364322
0.96804147	-4.807070e-02	6.595136e-01	0.9997779456	-0.0343033973	0.7939870274	0.95972154	-1.332062e-01	0.228637539	0.9982883	-9.716505e-03	0.9428688959
0.83725376	3.252444e-02	4.070852e-01	0.9997779456	-0.0986941073	0.3652271936	0.87664366	-1.264055e-03	0.990151170	0.9986411	1.689069e-01	0.2708902059
0.82862325	2.539293e-01	1.274632e-01	0.9997779456	0.2305411996	0.3375021202	0.85925321	-3.926961e-02	0.873851542	0.9982883	-2.325485e-01	0.3664127480
0.97522657	-3.957690e-02	6.456603e-01	0.9997779456	-0.2510947226	0.0757521105	0.83807199	1.522624e-02	0.852441322	0.9982883	-1.666952e-02	0.9000092296
0.84824569	-3.660635e-02	6.925405e-01	0.9997779456	0.0479186939	0.6499249671	0.93854411	-1.794754e-02	0.765972509	0.9982883	-1.748751e-02	0.8345459433
0.94965779	3.125407e-01	1.161439e-01	0.9997779456	-0.2533141665	0.2938481791	0.84915316	-3.520699e-01	0.239064574	0.9982883	-5.335363e-02	0.8328489643
0.79213004	2.196747e-01	2.554572e-02	0.9997779456	0.0333023413	0.7920251778	0.95972154	9.942436e-02	0.301459724	0.9982883	2.529467e-01	0.0759561007
0.83725376	3.235725e-01	1.613003e-01	0.9997779456	0.1692119719	0.6479253250	0.93854411	-9.777470e-02	0.794323371	0.9982883	8.894719e-02	0.7530107132
0.77869908	7.406605e-02	2.643532e-01	0.9997779456	0.0695124387	0.6489939464	0.93854411	9.292057e-02	0.442912729	0.9982883	-3.636270e-02	0.7377015959
0.87684526	-2.895311e-02	6.395892e-01	0.9997779456	-0.0562560046	0.5124472797	0.90612020	3.599578e-02	0.549403443	0.9982883	1.053816e-01	0.0806854938
0.82743594	-1.044175e-01	7.546736e-01	0.9997779456	-0.2288806197	0.1750300251	0.83807199	-1.596296e-02	0.891681333	0.9982883	8.443435e-02	0.7263008340
0.84824569	-7.331228e-02	8.433571e-01	0.9997779456	-0.1413684179	0.1909236646	0.83807199	-7.202352e-03	0.898890860	0.9982883	-2.305169e-02	0.8408306272
0.97050390	-2.449643e-01	9.550113e-01	0.9997779456	-0.0967197103	0.7752736450	0.95657490	-2.868695e-02	0.852620285	0.9982883	8.411683e-02	0.6719990006
0.99066584	-4.905112e-02	6.347231e-01	0.9997779456	0.0619621260	0.6725994066	0.94130886	-6.146035e-03	0.960540839	0.9986411	-1.597581e-01	0.3170719171
0.97273515	7.356692e-01	1.187207e-01	0.9997779456	1.6844230621	0.2884290726	0.84915316	3.950968e-01	0.529368461	0.9982883	-1.977731e-01	0.7045102703
0.89369078	-3.612984e-01	8.478530e-01	0.9997779456	-0.3603262922	0.1522434759	0.83807199	-4.205991e-01	0.158054266	0.9982883	4.514645e-01	0.4123101474
0.97522657	-3.848873e-01	9.951088e-01	0.9997779456	0.0140403340	0.9361701133	0.98648257	1.247286e-01	0.375024640	0.9982883	-4.430512e-01	0.0039424095
0.82862325	7.867381e-02	4.385654e-01	0.9997779456	-0.1133059100	0.7606946155	0.95505354	6.850654e-02	0.879990669	0.9982883	1.780484e-01	0.7076794026
0.77869908	1.839245e-01	3.598263e-01	0.9997779456	0.4677369693	0.3858906485	0.87916124	1.389278e-01	0.791509440	0.9982883	-5.775608e-02	0.9238074396
0.77869908	4.328886e-02	3.465400e-01	0.9997779456	0.2687954233	0.0608052078	0.83807199	-5.300134e-03	0.953780053	0.9986411	1.635412e-02	0.9026699759
0.94189574	-1.580135e-01	9.694858e-01	0.9997779456	-0.0612752405	0.6229462804	0.93441942	-7.320363e-02	0.388212442	0.9982883	-1.156652e-01	0.3507050696
0.87292023	-9.849554e-02	8.487904e-01	0.9997779456	-0.1647309417	0.1661053264	0.83807199	-5.833739e-02	0.456495961	0.9982883	-2.220456e-01	0.1256714381
0.77869908	5.421028e-02	3.543345e-01	0.9997779456	-0.3687273236	0.0065041907	0.40915301	-1.903419e-01	0.083450049	0.9982883	-1.398736e-03	0.9894925610
0.93359368	-9.406266e-02	9.105027e-01	0.9997779456	-0.1764893487	0.0558531810	0.83807199	-9.347897e-02	0.108938484	0.9982883	-3.631562e-02	0.6644053790
0.79578964	6.814471e-02	4.382644e-01	0.9997779456	0.4088576083	0.3855277051	0.87916124	5.806133e-02	0.891665627	0.9982883	-6.934895e-02	0.8501316269
0.97273515	-4.423111e-02	6.325824e-01	0.9997779456	0.0727633921	0.5713283542	0.92122660	1.076997e-01	0.210514278	0.9982883	-5.701096e-02	0.5520210105
0.80820047	3.731950e-01	2.561264e-01	0.9997779456	-0.3297623004	0.5611829125	0.91675659	1.703288e-01	0.759006827	0.9982883	-2.560452e-01	0.6391504600
0.87292023	-8.707085e-02	6.338586e-01	0.9997779456	-0.0996769396	0.6253501334	0.93565045	9.623868e-02	0.636815287	0.9982883	3.209918e-01	0.2584554831
0.77869908	6.725559e-01	1.043814e-01	0.9997779456	0.6991398653	0.3176875632	0.84915316	-8.711055e-02	0.887094061	0.9982883	-2.092359e-01	0.7623994323
0.77869908	-3.529488e-01	8.284280e-01	0.9997779456	0.4191841462	0.4980022521	0.90612020	2.443209e-02	0.938538533	0.9986411	6.588326e-01	0.2980680891
0.98996967	5.195256e-02	4.533051e-01	0.9997779456	-0.3156811108	0.5965269939	0.92532140	-1.543718e-01	0.721679022	0.9982883	-1.129609e+00	0.1541019540
0.82743594	1.621965e-01	2.593203e-02	0.9997779456	-0.0628511154	0.6948408814	0.94482995	6.905231e-02	0.456902494	0.9982883	1.935122e-01	0.0535835430
0.97273515	2.635117e-01	4.528625e-02	0.9997779456	0.2892845911	0.4653588016	0.90612020	1.120711e-01	0.550153102	0.9982883	1.448198e-01	0.3634146816
0.77869908	-2.058652e-02	5.572070e-01	0.9997779456	-0.0787751676	0.6639102422	0.94041014	-1.250284e-02	0.921535507	0.9982883	2.323679e-01	0.0977963090
0.95558280	-9.713763e-02	6.588119e-01	0.9997779456	0.0097989570	0.9652162949	0.99380888	-4.362099e-02	0.816222097	0.9982883	-1.268277e-01	0.3465649615
0.77869908	-5.463217e-02	7.495606e-01	0.9997779456	-0.1689405562	0.2209904634	0.83807199	-2.359432e-02	0.722308910	0.9982883	-1.135298e-01	0.4271443463

0.96804147	1.606053e-01	1.782528e-01	0.9997779456	0.0876049159	0.6549398591	0.93854411	7.726059e-02	0.667772796	0.9982883	5.641133e-02	0.7219843181
0.77869908	-1.119766e-01	8.134124e-01	0.9997779456	-0.1323685283	0.3364014205	0.85880449	-7.752483e-02	0.538762208	0.9982883	-1.290287e-01	0.2948373302
0.90482806	-8.195204e-03	5.370831e-01	0.9997779456	0.0731479752	0.4430632933	0.90612020	-5.014513e-02	0.496099170	0.9982883	8.751704e-03	0.9051036254
0.77869908	2.403639e-01	1.705216e-01	0.9997779456	0.3004871579	0.2843083444	0.84915316	2.097747e-01	0.378488941	0.9982883	5.098658e-02	0.8930496754
0.95284371	-1.430141e-02	5.731284e-01	0.9997779456	-0.1147603446	0.3721180127	0.87664366	-1.755458e-02	0.804600982	0.9982883	-6.411079e-02	0.5325168373
0.77869908	-8.431727e-02	8.195382e-01	0.9997779456	-0.1772907612	0.3714580691	0.87664366	1.616467e-02	0.868705972	0.9982883	-6.995734e-02	0.5736417689
0.93081046	-1.098100e-01	9.217058e-01	0.9997779456	-0.0571153753	0.5957082267	0.92532140	1.466443e-02	0.835896452	0.9982883	-4.695732e-02	0.6461753057
0.88203342	-8.118327e-02	8.388630e-01	0.9997779456	-0.0436045391	0.6984181328	0.94482995	5.861108e-02	0.461204138	0.9982883	-1.713972e-02	0.8924156958
0.89512661	1.049923e-01	3.304482e-01	0.9997779456	-0.1570401762	0.5270791583	0.90612020	1.288429e-01	0.531748065	0.9982883	1.975815e-01	0.4430043696
0.77869908	-1.280638e-02	5.613007e-01	0.9997779456	-0.1385649648	0.0646451958	0.83807199	-8.645858e-02	0.165797422	0.9982883	-3.467002e-02	0.6047663950
0.79972808	-6.030463e-02	6.216520e-01	0.9997779456	-0.0322194851	0.8091057211	0.95972154	-9.513368e-02	0.603579240	0.9982883	-2.052588e-01	0.2634029979
0.77869908	-5.777231e-02	6.022646e-01	0.9997779456	-0.1273173275	0.6433383585	0.93854411	1.038443e-02	0.948584807	0.9986411	-2.567148e-01	0.3502932576
0.87292023	7.912128e-02	2.680092e-01	0.9997779456	-0.3417925441	0.0738951758	0.83807199	-1.698719e-01	0.205611568	0.9982883	1.382551e-01	0.5974760746
0.92004850	-4.593721e-02	5.837568e-01	0.9997779456	0.3069950832	0.4488987820	0.90612020	4.444815e-01	0.067370548	0.9982883	-1.299259e-01	0.6022388463
0.96804147	5.759900e-02	3.897670e-01	0.9997779456	0.4132174282	0.2067318398	0.83807199	1.380983e-01	0.490850870	0.9982883	3.045887e-01	0.2026676197
0.77869908	4.539963e-02	4.741058e-01	0.9997779456	0.3084833273	0.6372924959	0.93852299	1.070422e-01	0.874449479	0.9982883	-2.156946e-01	0.7393094736
0.98226159	-1.931303e-01	8.337561e-01	0.9997779456	0.1849591248	0.1809584581	0.83807199	6.067766e-02	0.723530146	0.9982883	-1.132225e-01	0.6144577098
0.90482806	-8.687217e-02	7.953475e-01	0.9997779456	-0.2050307770	0.0220189079	0.60526393	-7.914133e-02	0.446930945	0.9982883	-1.307998e-01	0.3990775367
0.86909435	6.341683e-02	4.202077e-01	0.9997779456	0.7315355641	0.1735582486	0.83807199	2.209587e-01	0.385295879	0.9982883	-1.391916e-02	0.9737313496
0.77869908	2.604882e-01	3.177822e-01	0.9997779456	0.9369537361	0.1858883892	0.83807199	3.786012e-01	0.483160813	0.9982883	1.181277e-01	0.8732444279
0.89369078	1.032368e-01	3.057609e-01	0.9997779456	-0.1413211877	0.4558683773	0.90612020	-2.235499e-02	0.907740930	0.9982883	-3.648894e-01	0.1168359250
0.89369078	-8.080502e-02	9.010103e-01	0.9997779456	-0.0742438927	0.5153571405	0.90612020	5.586755e-02	0.343232296	0.9982883	-1.843672e-02	0.8261890779
0.83140044	-4.568926e-02	6.727292e-01	0.9997779456	0.0674900236	0.6090890267	0.93042774	6.889553e-02	0.430608900	0.9982883	-1.024198e-01	0.2737641921
0.98996967	2.282367e-02	4.175344e-01	0.9997779456	0.0429133750	0.6439724458	0.93854411	7.503347e-02	0.387511092	0.9982883	-1.372873e-01	0.2452260740
0.87292023	-8.526091e-02	8.185558e-01	0.9997779456	-0.1186506288	0.3121675924	0.84915316	4.181074e-02	0.604001705	0.9982883	-3.630133e-02	0.7332323585
0.77869908	-2.065429e-02	5.981625e-01	0.9997779456	-0.0473857530	0.6975202253	0.94482995	7.211090e-03	0.921555456	0.9982883	9.790352e-02	0.3672749750
0.81163165	1.966502e-01	3.178904e-02	0.9997779456	0.2158939332	0.1660645033	0.83807199	1.190926e-01	0.253031663	0.9982883	4.193089e-01	0.0032283859
0.92876201	5.906815e-02	3.419949e-01	0.9997779456	-0.2083431401	0.1085002179	0.83807199	6.103656e-02	0.623652711	0.9982883	1.834966e-01	0.3737765026
0.77869908	3.381118e-01	9.548002e-02	0.9997779456	-0.3798548226	0.1245185836	0.83807199	-5.478439e-01	0.133352109	0.9982883	-3.066789e-01	0.4595201855
0.77869908	7.690920e-02	1.408517e-01	0.9997779456	0.0636361521	0.6087963135	0.93042774	1.033957e-01	0.127264823	0.9982883	7.404022e-02	0.3762875811
0.81163165	1.595817e-01	1.123114e-01	0.9997779456	-0.0589756948	0.6292087979	0.93786229	-2.512920e-02	0.801180280	0.9982883	-9.508749e-02	0.4390140025
0.83731157	2.749861e-01	1.590528e-02	0.9997779456	0.2902981322	0.1328246309	0.83807199	2.273824e-02	0.842405890	0.9982883	-1.080602e-01	0.5672131798
0.77869908	-7.847602e-02	5.460559e-01	0.9997779456	-0.8637506822	0.0514136569	0.83664873	-1.337141e+00	0.030360649	0.9982883	-1.796054e+00	0.0012180367
0.81552863	-2.664524e-01	6.080878e-01	0.9997779456	0.5222893679	0.4760269604	0.90612020	6.940336e-01	0.382406354	0.9982883	-3.123837e-01	0.7026064005
0.04929222	5.163365e-02	2.934999e-01	0.9997779456	-0.0471706176	0.6549761268	0.93854411	1.444520e-01	0.063817383	0.9982883	2.383845e-02	0.8016557030
0.77869908	-4.563450e-03	5.203249e-01	0.9997779456	-0.0214483603	0.7484007236	0.95349818	-8.802192e-02	0.158577998	0.9982883	-2.552828e-02	0.6615648013
0.81163165	-1.981528e-03	5.072609e-01	0.9997779456	0.0231752460	0.8792811839	0.96947881	1.523788e-01	0.110512589	0.9982883	-1.395431e-03	0.9919270755
0.77869908	1.347272e-01	1.055975e-01	0.9997779456	-0.1311828154	0.3693744291	0.87664366	4.270397e-02	0.608037799	0.9982883	-2.520252e-03	0.9741185725
0.96804147	6.328180e-02	3.409309e-01	0.9997779456	0.0764862907	0.4543299934	0.90612020	1.733891e-01	0.188579774	0.9982883	2.177914e-01	0.1793089018
0.77869908	-2.275149e-03	5.165307e-01	0.9997779456	0.0366988410	0.5549515550	0.91608209	4.877748e-02	0.341726821	0.9982883	5.745964e-02	0.2838497811
0.82862325	-1.098502e-01	7.041812e-01	0.9997779456	0.1104303242	0.6546750920	0.93854411	4.986135e-02	0.787038380	0.9982883	-4.179833e-02	0.8870753731
0.77869908	-3.165475e-02	6.553941e-01	0.9997779456	-0.0756274404	0.2367006914	0.84413548	-5.214560e-02	0.429458184	0.9982883	2.064489e-02	0.8250945264
0.87099999	-6.001142e-02	8.255832e-01	0.9997779456	-0.0780303501	0.3980424509	0.88596624	-3.593175e-02	0.550940763	0.9982883	9.574853e-03	0.9113468344
0.83725376	1.352519e-01	3.928381e-01	0.9997779456	0.5608773141	0.3028477445	0.84915316	1.997787e-01	0.639895282	0.9982883	-5.650644e-02	0.9106581718
0.93531428	-1.865549e-01	9.420411e-01	0.9997779456	0.0324343414	0.8156981276	0.95972154	-8.561973e-02	0.459774383	0.9982883	-1.915538e-01	0.0616131631
0.83563937	-3.448022e-02	6.270162e-01	0.9997779456	-0.1340908578	0.2446387867	0.84413548	-1.899295e-02	0.841450025	0.9982883	1.897840e-01	0.0802116176
0.97050390	-2.153278e-01	9.796839e-01	0.9997779456	-0.1290379296	0.1728432360	0.83807199	-2.448339e-02	0.806245889	0.9982883	-1.446422e-02	0.9048731397
0.77869908	-4.449626e-02	5.532328e-01	0.9997779456	-0.2333271912	0.4763225850	0.90612020	8.236246e-02	0.728531886	0.9982883	-9.782065e-02	0.7641470759
0.98996967	5.315483e-02	2.433080e-01	0.9997779456	0.1215366625	0.1700020788	0.83807199	5.722655e-02	0.439834266	0.9982883	-4.411330e-03	0.9685229844
0.97273515	-1.506743e-01	8.453827e-01	0.9997779456	-0.1359383525	0.4090382877	0.89501894	3.318090e-02	0.784210131	0.9982883	-1.814021e-01	0.3602032325
0.77869908	-5.605242e-02	6.753129e-01	0.9997779456	-0.0125293796	0.9123738825	0.97964668	1.320575e-01	0.219060062	0.9982883	-7.903234e-02	0.6505454388
0.77869908	1.022155e-01	1.598844e-01	0.9997779456	-0.0187581909	0.8672717121	0.96707132	6.263742e-02	0.456677498	0.9982883	5.150645e-02	0.7288795283
0.81804251	-9.241004e-02	8.057996e-01	0.9997779456	-0.2767918496	0.1271126562	0.83807199	-1.410184e-01	0.125291856	0.9982883	8.698947e-02	0.4070242239
0.80653217	-1.622698e-01	8.778686e-01	0.9997779456	-0.3125664538	0.1448812171	0.83807199	7.560565e-02	0.580037425	0.9982883	-3.707533e-02	0.7906884234
0.95284371	-1.308243e-01	9.085194e-01	0.9997779456	-0.1710468671	0.0987225124	0.83807199	1.022495e-01	0.195018289	0.9982883	-7.210753e-02	0.4997229205
0.84600172	-3.664711e-02	5.721187e-01	0.9997779456	0.0563044295	0.8333582613	0.96288315	-2.467344e-03	0.988110834	0.9986411	-1.781550e-02	0.9321531138
0.77869908	1.356155e-02	4.462721e-01	0.9997779456	-0.1613171788	0.2970012415	0.84915316	-1.147992e-01	0.103306714	0.9982883	-7.224290e-02	0.5825439096
0.97273515	-3.316406e-01	9.320695e-01	0.9997779456	0.0083138672	0.9570727829	0.99380888	4.532274e-02	0.791032413	0.9982883	-1.603298e-01	0.3509336531
0.77869908	4.526205e-02	2.603760e-01	0.9997779456	-0.0493681636	0.5412608040	0.91146188	6.383169e-02	0.229618949	0.9982883	1.062618e-01	0.0271242952
0.89273364	1.537517e-01	1.868589e-01	0.9997779456	-0.1651606004	0.5054583504	0.90612020	1.047621e-01	0.513900157	0.9982883	1.318498e-01	0.2703974472
0.95472383	1.978539e-01	3.810370e-01	0.9997779456	0.8390501105	0.4791466067	0.90612020	1.197076e+00	0.103109596	0.9982883	3.071246e-02	0.9738872467
0.98996967	-3.267399e-02	5.847381e-01	0.9997779456	0.4868250838	0.1053533321	0.83807199	1.564972e-01	0.278995467	0.9982883	-1.783865e-02	0.8822630326
0.80820047	-8.331229e-02	7.952606e-01	0.9997779456	0.0311712782	0.7833879037	0.95657490	-8.289862e-02	0.397014682	0.9982883	3.517336e-02	0.7684494619
0.97273515	-2.363262e-02	5.093299e-01	0.9997779456	2.0730465514	0.3861884828	0.87916124	3.564287e-01	0.736614257	0.9982883	-1.348712e+00	0.1791497148
0.77869908	-2.651797e-02	6.426387e-01	0.9997779456	-0.1072108924	0.2260883572	0.83807199	4.445602e-02	0.437310231	0.9982883	-5.424664e-02	0.5071523012
0.77869908	3.915205e-02	2.774676e-01	0.9997779456	-0.0015375055	0.9818264664	0.99380888	2.175819e-02	0.648090255	0.9982883	-5.246538e-02	0.2718725014
0.77869908	6.703938e-02	4.557091e-01	0.9997779456	0.7317973735	0.3092908458	0.84915316	1.832969e-01	0.745702866	0.9982883	-7.334088e-02	0.8950280861
0.79972808	-9.032401e-02	6.838417e-01	0.9997779456	0.0545821455	0.8479867599	0.96338860	2.314348e-01	0.164495697	0.9982883	2.513704e-01	0.2205156060
0.93081046	-1.195445e-01	7.993609e-01	0.9997779456	-0.2399988732	0.2805458223	0.84915316	1.043457e-01	0.477626050	0.9982883	-1.324315e-01	0.6231037218
0.97273515	-1.172003e-01	7.153128e-01	0.9997779456	-0.0658224604	0.7815581042	0.95657490	1.278686e-01	0.528043622	0.9982883	-3.934149e-01	0.2157397560
0.77869908	2.181476e+00	5.307418e-02	0.9997779456	3.1433480337	0.1789760664	0.83807199	1.326693e+00	0.263732325	0.9982883	4.390211e-02	0.9613549294
0.83140044	-8.605687e-02	8.444142e-01	0.9997779456	-0.0578789933	0.6207034753	0.93342431	-2.240335e-02	0.739783783	0.9982883	2.002858e-02	0.8446842205
0.98670901	2.698678e-01	1.110940e-01	0.9997779456	0.7062317856	0.2998942899	0.84915316	1.167209e-01	0.599999518	0.9982883	8.970093e-02	0.6681341166
0.77869908	-2.329864e-01	8.539005e-01	0.9997779456	-0.4601205988	0.0492115370	0.83664873	6.474621e-02	0.746387029	0.9982883	2.821589e-01	0.3180960408
0.95284371	-2.131516e-01	9.079324e-01	0.9997779456	-0.1213090943	0.5958832605	0.92532140	-1.722869e-01	0.288684575	0.9982883	-1.998957e-01	0.3975418008
0.89512661	1.075292e-02	4.324311e-01	0.9997779456	-0.0031383909	0.9718045324	0.99380888	3.916547e-02	0.589232438	0.9982883	7.192419e-02	0.3257296530
0.98996967	-1.034798e-02	5.193683e-01	0.9997779456	0.3637860558	0.1387112282	0.83807199	1.086617e-01	0.432716715	0.9982883	-1.739923e-01	0.2018859367
0.84315681	9.122241e-02	2.237202e-01	0.9997779456	-0.1900602852	0.2388637827	0.84413548	-4.739061e-02	0.655108044	0.9982883	-1.346271e-01	0.2360760511
0.77869908	1.317426e-01	2.319421e-01	0.9997779456	-0.2372033273	0.1189354575	0.83807199	-1.747118e-01	0.308182099	0.9982883	-4.684814e-02	0.8254381425
0.97273515	2.631061e-02	4.389015e-01	0.9997779456	0.2991456230	0.1552996102	0.83807199	-2.343730e-01	0.098886813	0.9982883	-3.715724e-01	0.1112179462
0.97273515	-8.208800e-04	5.046443e-01	0.9997779456	-0.0901811034	0.2374047068	0.84413548	-9.031791e-02	0.132426482	0.9982883	-2.143828e-02	0.8206800753
0.94008598	-1.697815e-01	9.725334e-01	0.9997779456	-0.2197589941	0.0117208775	0.46180258	4.852581e-02	0.520291101	0.9982883	-9.587447e-02	0.3531946879
0.96804147	-1.877927e-02	5.737274e-01	0.9997779456	0.0366195770	0.7903311060	0.95972154	-5.533657e-02	0.514777549	0.9982883	6.113876e-02	0.4804738009
0.92598653	-1.424286e-01	9.610611e-01	0.9997779456	-0.1866450106	0.1218298848	0.83807199	-3.998850e-02	0.526971277	0.9982883	3.001080e-02	0.7317168314
0.98996967	-5.906155e-01	8.916004e-01	0.9997779456	0.3946929057	0.2651772509	0.84915316	2.532423e-01	0.409639604	0.9982883	-3.872084e-01	0.1366366957
0.99066584	-8.616683e-01	9.981060e-01	0.9997779456	-0.6222897140	0.0683172063	0.83807199	3.788902e-01	0.186797185	0.9982883	-5.389566e-01	0.1748955037
0.86909435	4.101836e-02	4.592905e-01	0.9997779456	0.2416183886	0.3773104677	0.87916124	1.171156e-01	0.719491393	0.9982883	-1.452143e-01	0.6571166793
0.86909435	-1.103320e-01	9.330961e-01	0.9997779456	-0.2137581102	0.0460748196	0.83664873	-4.454126e-02	0.533161889	0.9982883	-4.537498e-02	0.7036617877
0.77869908	-2.802620e-02	6.447534e-01	0.9997779456	-0.1354143149	0.2736927633	0.84915316	-1.011383e-02	0.874160246	0.9982883	-1.115217e-02	0.8912995583
0.90581107	1.987290e-01	2.062557e-01	0.9997779456	0.1470272116	0.6729323085	0.94130886	-1.598224e-01	0.463729306	0.9982883	-2.943739e-01	0.2100631739
0.97996235	-5.245929e-02	7.479775e-01	0.9997779456	0.0029098217	0.9819981675	0.99380888	-6.220341e-02	0.307082501	0.9982883	-6.971345e-02	0.4820343851
0.95457754	1.479304e-01	4.367230e-01	0.9997779456	1.6345049681	0.4111256667	0.89501894	1.558223e+00	0.104428139	0.9982883	-1.097767e+00	0.1600498013
0.87099999	3.365441e-01	1.968074e-01	0.9997779456	0.1110884471	0.8646723662	0.96707132	3.704115e-01	0.406462778	0.9982883	-3.918640e-01	0.4747927452
0.97273515	1.452286e-01	2.126898e-01	0.9997779456	0.2087730058	0.2764955910	0.84915316	4.577891e-01	0.014829200	0.9982883	4.583800e-01	0.3329342376
0.81552863	-2.214710e-01	9.913916e-01	0.9997779456	-0.1777182063	0.0787354048	0.83807199	1.529673e-02	0.852503554	0.9982883	5.862218e-03	0.9528596791
0.90581107	-2.306798e-02	6.135779e-01	0.9997779456	-0.0063913330	0.9658738220	0.99380888	-1.001588e-01	0.203760754	0.9982883	-1.715939e-01	0.0490502683
0.90298719	-9.169734e-02	8.898401e-01	0.9997779456	-0.0178297198	0.8680733219	0.96707132	-4.269407e-03	0.952159101	0.9986411	1.648526e-02	0.8398960727
0.87292023	2.713461e-01	7.343833e-03	0.9997779456	0.1900439720	0.2206258023	0.83807199	7.845473e-02	0.457149278	0.9982883	-2.811450e-02	0.8486146560
0.97273515	-3.433818e-02	6.043232e-01	0.9997779456	0.1067088760	0.3697755583	0.87664366	-3.124322e-02	0.767600932	0.9982883	-9.139220e-02	0.4612089639
0.77869908	-1.727333e-02	5.638129e-01	0.9997779456	-0.1371153031	0.3229530959	0.85589812	-5.302586e-02	0.486726782	0.9982883	-3.888266e-02	0.7242413533
0.93382935	1.553692e-01	2.350020e-01	0.9997779456	-0.0918799934	0.7348716997	0.94712279	8.987066e-02	0.597276711	0.9982883	3.674907e-01	0.2964267694
0.95558280	-4.142438e-02	6.328703e-01	0.9997779456	-0.1133167951	0.4147631545	0.89754931	-6.944854e-03	0.949795947	0.9986411	1.998660e-01	0.1911946384
0.77869908	1.213776e-01	1.748867e-01	0.9997779456	-0.1973811860	0.0430370037	0.80745616	-8.582177e-02	0.523682226	0.9982883	1.925076e-01	0.2486720592
0.77869908	1.217457e-01	1.254667e-01	0.9997779456	0.0162449647	0.9040120817	0.97582904	3.283068e-03	0.961272214	0.9986411	3.376993e-02	0.7203138578
0.98323587	-2.783618e-02	5.492998e-01	0.9997779456	0.3257237639	0.5311943251	0.90612020	-7.484031e-02	0.717712234	0.9982883	-1.033624e-01	0.6124237114
0.81163165	-3.920025e-01	7.580997e-01	0.9997779456	-0.8774788726	0.0591659621	0.83807199	-6.440058e-01	0.181800922	0.9982883	1.040605e-01	0.8749086782
0.88636459	8.879918e-02	2.314203e-01	0.9997779456	-0.0688227957	0.5492620637	0.91608209	7.898326e-03	0.931313082	0.9986411	6.792634e-02	0.5245240314
0.96804147	-4.303735e-01	6.969144e-01	0.9997779456	-0.4401517539	0.2626986842	0.84915316	1.253549e-01	0.805941223	0.9982883	2.957865e-01	0.6738089753
0.84600172	-4.843109e-02	6.952279e-01	0.9997779456	-0.0470064935	0.5899695012	0.92532140	1.502373e-01	0.074406381	0.9982883	6.806002e-02	0.5441824293
0.98996967	-1.375066e-01	7.748745e-01	0.9997779456	-0.1304476979	0.3622394529	0.87404187	-1.636236e-01	0.266845043	0.9982883	7.664888e-02	0.6815434482
0.77869908	-3.680527e-01	6.022490e-01	0.9997779456	-0.7580164676	0.4885430271	0.90612020	-2.895297e-02	0.981743069	0.9986411	-2.061170e+00	0.0563778217
0.77869908	-4.935013e-03	5.290131e-01	0.9997779456	-0.0198435303	0.8065430253	0.95972154	5.683312e-02	0.309457487	0.9982883	-2.167572e-02	0.6918400678
0.79972808	-7.192344e-02	8.447218e-01	0.9997779456	-0.0187758095	0.8159585947	0.95972154	-1.834135e-02	0.782289212	0.9982883	-3.093196e-02	0.7611785401
0.77869908	1.210630e-02	4.654555e-01	0.9997779456	-0.2568348823	0.0296067203	0.66532795	4.216930e-02	0.704898621	0.9982883	-6.479151e-02	0.7017556638
0.77869908	-4.248020e-01	9.456432e-01	0.9997779456	-0.2223950525	0.2581633035	0.84915316	-1.442365e-01	0.518091309	0.9982883	1.790710e-01	0.6502388051
0.81552863	-3.629255e-02	7.562940e-01	0.9997779456	-0.0744945296	0.3630749226	0.87404187	1.025549e-01	0.049471456	0.9982883	-2.442087e-02	0.7802522347
0.86909435	-1.350842e-02	5.769561e-01	0.9997779456	-0.0314036382	0.7571380100	0.95505354	8.856170e-02	0.142029749	0.9982883	1.021299e-01	0.1369906931
0.77869908	2.463504e-01	2.253016e-02	0.9997779456	-0.2606847327	0.1958008634	0.83807199	-9.568456e-02	0.375112844	0.9982883	2.760570e-01	0.0373475751
0.93081046	3.152589e-01	8.553364e-02	0.9997779456	0.4732911501	0.1040168812	0.83807199	2.084100e-01	0.303487531	0.9982883	-8.164881e-02	0.7870461696
0.77869908	1.010061e-01	9.601384e-02	0.9997779456	-0.0162900755	0.8258725248	0.95988696	5.820041e-02	0.375418572	0.9982883	-1.063651e-01	0.2741373466
0.99018039	-3.372135e-01	9.786733e-01	0.9997779456	-0.2993855931	0.1334123782	0.83807199	-5.395016e-02	0.726396555	0.9982883	-3.297524e-01	0.1293531053
0.97050390	-3.824500e-02	6.135951e-01	0.9997779456	0.0033584255	0.9794952021	0.99380888	4.333961e-02	0.778900107	0.9982883	3.344234e-01	0.0405557237
0.83725376	1.341148e-01	4.687028e-02	0.9997779456	0.0946889183	0.4615647642	0.90612020	-7.932825e-03	0.920283896	0.9982883	1.141944e-01	0.2780933207
0.80820047	-1.157637e+00	8.243476e-01	0.9997779456	-0.8938435174	0.1425285521	0.83807199	5.115072e-01	0.463767238	0.9982883	2.031780e-01	0.7617034871
0.77869908	-2.811467e-02	6.150930e-01	0.9997779456	-0.1848244863	0.0381122440	0.76504055	-7.726610e-02	0.364488145	0.9982883	2.972784e-02	0.7599079701
0.97975724	-2.674590e-01	8.881133e-01	0.9997779456	0.0493842400	0.7227840623	0.94712279	-7.080206e-02	0.721112227	0.9982883	2.779190e-02	0.8387775409
0.86909435	-6.513071e-02	7.362526e-01	0.9997779456	-0.0696870791	0.5875025557	0.92532140	-1.001172e-02	0.915286704	0.9982883	-1.620246e-01	0.1967768881
0.77869908	-1.835231e-01	9.539550e-01	0.9997779456	-0.2097266397	0.2267326272	0.83807199	2.732568e-02	0.756496645	0.9982883	-8.672932e-03	0.9179929250
0.91765245	-2.496279e-01	7.927381e-01	0.9997779456	-0.0065441230	0.9866800552	0.99380888	-1.784678e-01	0.490068324	0.9982883	-8.154223e-01	0.0030114841
0.84824569	-8.298592e-02	7.881119e-01	0.9997779456	-0.0758160206	0.6417201441	0.93854411	7.243930e-02	0.389521451	0.9982883	7.793849e-02	0.5492860600
0.97273515	-5.350453e-02	6.994858e-01	0.9997779456	0.1911157314	0.1090275746	0.83807199	1.370198e-02	0.878697085	0.9982883	-1.170603e-01	0.3142899335
0.87292023	1.962479e-01	1.747648e-01	0.9997779456	0.2220561607	0.4610903539	0.90612020	9.717484e-02	0.557497545	0.9982883	-1.134087e-01	0.6305273157
0.97522657	-5.923329e-01	6.861455e-01	0.9997779456	0.1236770829	0.9125135344	0.97964668	-2.470077e-02	0.980647615	0.9986411	-1.837700e+00	0.1027516442
0.87891050	4.772315e-02	2.715283e-01	0.9997779456	0.0116008056	0.8857983588	0.97215753	6.249855e-02	0.328895952	0.9982883	1.486857e-01	0.0587288584
0.77869908	5.978821e-02	2.947522e-01	0.9997779456	-0.1327848860	0.1015165351	0.83807199	-3.889164e-02	0.650875019	0.9982883	2.313144e-02	0.6843372353
0.95134638	6.812914e-01	6.974179e-02	0.9997779456	1.7088295669	0.1638203462	0.83807199	5.882924e-01	0.224085771	0.9982883	-6.125088e-01	0.1356189737
0.77869908	1.272371e-01	6.621373e-02	0.9997779456	0.0349408621	0.7128589585	0.94482995	4.303627e-03	0.953618330	0.9986411	8.482501e-02	0.2366471752
0.77869908	2.481957e-01	9.622053e-02	0.9997779456	-0.3245799049	0.1922146458	0.83807199	-1.650021e-02	0.914392230	0.9982883	4.382558e-01	0.0784083565
0.77869908	6.453182e-01	8.207922e-02	0.9997779456	0.3955998795	0.5257707630	0.90612020	8.331827e-02	0.898746543	0.9982883	1.209529e-01	0.8515925558

0.77869908	-3.217480e-02	6.852103e-01	0.9997779456	0.0280055780	0.7978887283	0.95972154	2.791955e-02	0.667594705	0.9982883	5.722247e-02	0.5549846796
0.92890486	-1.051146e-02	5.556224e-01	0.9997779456	0.0245821920	0.7637114546	0.95505354	6.188058e-02	0.377537289	0.9982883	8.939885e-02	0.1619955855
0.77869908	3.849267e-02	4.231217e-01	0.9997779456	0.0853414893	0.7568156229	0.95505354	3.648648e-02	0.869702998	0.9982883	-1.413302e-01	0.5124617082
0.77869908	1.540772e-02	4.647284e-01	0.9997779456	-0.0018510910	0.9920241557	0.99454839	-1.718993e-02	0.913236872	0.9982883	-8.288671e-02	0.6971097741
0.80820047	-1.287329e-01	8.371665e-01	0.9997779456	-0.1205267125	0.3713019101	0.87664366	2.881547e-02	0.768079940	0.9982883	9.820401e-02	0.4266347552
0.90802583	-7.801097e-02	7.401674e-01	0.9997779456	-0.0163464623	0.9431458435	0.98742107	9.146300e-02	0.466533883	0.9982883	3.822874e-01	0.0176627262
0.97273515	-2.766280e-01	9.481589e-01	0.9997779456	0.0052788896	0.9797417439	0.99380888	3.218347e-02	0.802359838	0.9982883	-1.471291e-01	0.1984444753
0.77869908	-6.544979e-02	7.994849e-01	0.9997779456	-0.0553346213	0.5080018500	0.90612020	5.081166e-02	0.493861840	0.9982883	7.272918e-02	0.3934224911
0.77869908	-7.726983e-02	8.414786e-01	0.9997779456	-0.0236453114	0.7392200305	0.94973704	8.481795e-02	0.208066472	0.9982883	-9.157379e-02	0.3352564789
0.96804147	2.859371e-01	1.748314e-02	0.9997779456	0.2084695087	0.1646091920	0.83807199	9.782228e-02	0.396188988	0.9982883	2.708816e-01	0.0384132554
0.86909435	-9.071675e-03	5.296883e-01	0.9997779456	-0.0161197183	0.8613653562	0.96707132	4.424427e-02	0.648980300	0.9982883	5.059571e-02	0.8124193825
0.77869908	1.440324e-01	1.667633e-01	0.9997779456	0.1354831330	0.3093556390	0.84915316	-3.643760e-02	0.763007294	0.9982883	-8.256467e-04	0.9960254728
0.95284371	3.567739e-01	1.203265e-01	0.9997779456	-0.0508460224	0.8973481181	0.97363552	2.288504e-02	0.928207933	0.9986411	-4.658752e-01	0.1822906666
0.77869908	9.292229e-02	1.542315e-01	0.9997779456	-0.0974088728	0.6116263557	0.93042774	1.055098e-02	0.900824184	0.9982883	1.333469e-01	0.1185485645
0.81804251	3.174834e-02	4.039832e-01	0.9997779456	-0.1380023006	0.3039099400	0.84915316	1.878822e-03	0.985408833	0.9986411	1.076861e-01	0.3971335956
0.97273515	4.666039e-02	3.983139e-01	0.9997779456	0.2291114354	0.4790436452	0.90612020	1.934989e-01	0.263253525	0.9982883	1.381481e-01	0.3894393428
0.77869908	3.054296e-02	3.684748e-01	0.9997779456	-0.0194589560	0.8381344584	0.96321135	5.212266e-02	0.465598163	0.9982883	1.108550e-01	0.0884373135
0.95284371	4.465479e-02	3.141150e-01	0.9997779456	0.0383828246	0.7441068060	0.95148651	1.029122e-02	0.920254241	0.9982883	7.652061e-02	0.5025895261
0.86909435	1.072276e-01	2.976944e-01	0.9997779456	-0.1797997441	0.5023303519	0.90612020	-3.759490e-01	0.030070803	0.9982883	2.794971e-02	0.8993604668
0.88581308	-8.383802e-02	7.556616e-01	0.9997779456	-0.0199031146	0.8761657603	0.96947881	-3.711778e-02	0.732798771	0.9982883	1.704090e-01	0.2737262458
0.77869908	4.707595e-02	4.413418e-01	0.9997779456	0.3478765753	0.3277131054	0.85754449	9.218281e-02	0.740343348	0.9982883	-1.051372e-01	0.6697633114
0.77869908	-4.026519e-02	6.317768e-01	0.9997779456	-0.0015743528	0.9899490903	0.99380888	-8.829277e-03	0.932063488	0.9986411	1.309436e-01	0.3197400805
0.97273515	-2.548180e-01	8.491933e-01	0.9997779456	0.0994008132	0.5480483270	0.91608209	-1.024065e-01	0.660683974	0.9982883	1.896634e-02	0.9211801621
0.97273515	-4.088561e-01	9.921687e-01	0.9997779456	-0.1557274297	0.3297773501	0.85754449	-8.167338e-02	0.582979149	0.9982883	5.328199e-02	0.8293880206
0.77869908	-3.168769e-02	6.596388e-01	0.9997779456	-0.0921421136	0.3505203915	0.86676800	8.315716e-03	0.878987894	0.9982883	1.087120e-02	0.8953562521
0.90482806	-1.668384e-01	9.480996e-01	0.9997779456	-0.1824510245	0.1527490488	0.83807199	-6.687013e-02	0.462184815	0.9982883	-1.480708e-01	0.0480278737
0.93368199	-7.709291e-02	8.673673e-01	0.9997779456	-0.1335858883	0.0113074125	0.46180258	-6.377596e-02	0.270856327	0.9982883	7.551818e-02	0.3158761942
0.77869908	6.485578e-02	1.209774e-01	0.9997779456	-0.0313839880	0.6988567212	0.94482995	1.948634e-02	0.696915013	0.9982883	3.405906e-02	0.7087784773
0.89512661	-8.504297e-02	7.792560e-01	0.9997779456	-0.2005919809	0.1477893426	0.83807199	-3.074925e-02	0.714215263	0.9982883	-1.559337e-01	0.1960432182
0.94189574	-1.378861e-01	9.153256e-01	0.9997779456	-0.0963318907	0.4704242057	0.90612020	-1.340232e-02	0.849706149	0.9982883	-8.715609e-03	0.9018617800
0.81163165	5.050598e-01	9.564222e-02	0.9997779456	1.2494473146	0.2214211993	0.83807199	5.838921e-01	0.137442758	0.9982883	-1.325531e-01	0.7070090464
0.89369078	-2.221099e-01	9.285692e-01	0.9997779456	0.0284153356	0.9268364793	0.98469702	-7.213613e-02	0.601621996	0.9982883	1.025195e-02	0.9637866780
0.77869908	6.275008e-02	3.441291e-01	0.9997779456	-0.1550500611	0.2965364578	0.84915316	-4.589335e-02	0.765430036	0.9982883	2.199008e-01	0.3107750260
0.81552863	-1.000829e-01	9.118837e-01	0.9997779456	-0.0163350591	0.8910244371	0.97246977	6.564292e-02	0.376113220	0.9982883	-9.531555e-03	0.9170862700
0.86909435	7.179197e-02	2.818886e-01	0.9997779456	-0.2234183772	0.1868917318	0.83807199	-8.394823e-03	0.945650790	0.9986411	1.940334e-01	0.3143042713
0.94675587	-3.192854e-01	7.627327e-01	0.9997779456	-0.7135665922	0.0444170556	0.82032750	8.506776e-02	0.827006507	0.9982883	2.201970e-01	0.8006970392
0.78646912	-1.350897e-01	7.684167e-01	0.9997779456	-0.2255846288	0.1039959676	0.83807199	-1.985075e-01	0.268538427	0.9982883	-3.879561e-01	0.0050901015
0.79972808	-1.122700e-01	8.927545e-01	0.9997779456	-0.1630173396	0.2297679892	0.84082280	6.529189e-03	0.934054508	0.9986411	1.920469e-02	0.8771972267
0.98998602	-1.325245e-02	5.852418e-01	0.9997779456	0.2360360806	0.0065769096	0.40915301	7.206016e-02	0.290213023	0.9982883	1.779412e-01	0.0015474128
0.95472383	-2.348218e-01	9.670290e-01	0.9997779456	-0.0414556965	0.6788306335	0.94316618	5.118283e-02	0.559366721	0.9982883	-2.569430e-01	0.0100069249
0.77869908	9.877863e-02	1.698636e-01	0.9997779456	-0.2347444819	0.0874028879	0.83807199	-1.608022e-01	0.101430505	0.9982883	1.616863e-01	0.2095895897
0.84824569	1.823291e-01	2.215033e-01	0.9997779456	0.0495265895	0.8446933335	0.96338860	1.779906e-01	0.323896893	0.9982883	3.239963e-02	0.8980588371
0.87099999	-7.736860e-02	7.959609e-01	0.9997779456	-0.0287367266	0.7351584785	0.94712279	-9.662955e-02	0.324926981	0.9982883	1.183961e-01	0.4252511655
0.89512661	4.339832e-02	2.417394e-01	0.9997779456	0.0661418994	0.4624156639	0.90612020	1.128175e-01	0.053621108	0.9982883	2.245918e-02	0.8124479625
0.97185522	-1.391516e-01	8.456786e-01	0.9997779456	-0.1577129029	0.2909846873	0.84915316	-9.661390e-02	0.434766421	0.9982883	-2.474746e-01	0.2100754960
0.92890486	1.056281e-01	2.243406e-02	0.9997779456	0.0162441006	0.8808969909	0.96947881	-4.491002e-03	0.937269591	0.9986411	-1.175707e-01	0.2090859759
0.88203342	-5.101227e-02	6.392436e-01	0.9997779456	-0.1959939129	0.0912200291	0.83807199	-6.503518e-02	0.557677487	0.9982883	9.228876e-02	0.4762767433
0.77869908	1.387270e-01	9.654026e-02	0.9997779456	0.0520110957	0.6509274252	0.93854411	9.812833e-03	0.922399815	0.9982883	1.353644e-01	0.2157905254
0.80820047	-7.375856e-02	8.641425e-01	0.9997779456	-0.0751166074	0.4286261424	0.90470732	5.163200e-02	0.371120342	0.9982883	1.679084e-02	0.8186267834
0.77869908	-4.498724e-02	6.281575e-01	0.9997779456	0.0497049090	0.8275167649	0.95988696	-1.216362e-01	0.447415798	0.9982883	-3.097909e-01	0.0064457438
0.77869908	4.377623e-02	3.996985e-01	0.9997779456	-0.0519161928	0.8170771583	0.95972154	2.324720e-02	0.873369794	0.9982883	-3.796095e-01	0.0134245391
0.79578964	4.239596e-02	4.343107e-01	0.9997779456	0.2303178492	0.3477426202	0.86676800	2.001101e-01	0.408520846	0.9982883	1.535848e-01	0.5749601272
0.77869908	4.121796e-02	3.245531e-01	0.9997779456	0.0105831460	0.8933828502	0.97246977	6.379873e-02	0.480529587	0.9982883	-7.203017e-02	0.5100911895
0.77869908	-1.804035e-02	5.255359e-01	0.9997779456	0.0210711653	0.9397456667	0.98648257	-6.880226e-03	0.974814751	0.9986411	2.169467e-02	0.9199430412
0.77869908	-1.128333e-01	8.298483e-01	0.9997779456	-0.1504635096	0.2522578965	0.84467092	2.519598e-03	0.977771061	0.9986411	7.061323e-02	0.5334915795
0.89911801	-2.093777e-01	8.262086e-01	0.9997779456	0.0459898914	0.8800288682	0.96947881	-2.139906e-01	0.214844873	0.9982883	-9.175375e-02	0.6147025720
0.83725376	-1.846442e-02	5.585441e-01	0.9997779456	-0.0622688286	0.6858551156	0.94482995	4.683022e-02	0.675203389	0.9982883	1.899585e-01	0.3001006139
0.83731157	-6.479384e-03	5.473414e-01	0.9997779456	0.0166341119	0.8474400014	0.96338860	8.319020e-02	0.118041163	0.9982883	-4.483148e-02	0.4340794362
0.77869908	2.807695e-01	1.381288e-01	0.9997779456	0.3218456488	0.3552670152	0.87302622	-9.087452e-02	0.705298508	0.9982883	5.241035e-01	0.0433042186
0.87891050	-2.674560e-02	6.138159e-01	0.9997779456	-0.1645939674	0.0770010396	0.83807199	-8.276769e-02	0.353997641	0.9982883	-3.970001e-02	0.7551936713
0.93738259	-8.287216e-02	7.964668e-01	0.9997779456	0.1466935128	0.2400645441	0.84413548	-6.663856e-02	0.458771640	0.9982883	1.212205e-01	0.3794047894
0.88636459	-9.225882e-02	8.164996e-01	0.9997779456	-0.0784248699	0.4951882021	0.90612020	1.957755e-02	0.834248978	0.9982883	1.204604e-01	0.3119934188
0.82743594	-5.030200e-02	7.591896e-01	0.9997779456	0.0443532623	0.6989394166	0.94482995	7.697800e-02	0.261752997	0.9982883	-1.578772e-02	0.8498320260
0.90482806	4.507159e-03	4.691376e-01	0.9997779456	0.0845924233	0.3098048587	0.84915316	3.767400e-02	0.501050048	0.9982883	-6.548728e-02	0.4157273779
0.79213004	5.306501e-02	3.792342e-01	0.9997779456	0.2302368378	0.1197760450	0.83807199	4.557381e-02	0.779049790	0.9982883	-1.231834e-03	0.9953060609
0.77869908	-9.071084e-02	6.021630e-01	0.9997779456	0.1018421703	0.7703724185	0.95505354	6.424433e-02	0.862361358	0.9982883	3.115335e-02	0.9178635794
0.77869908	-1.015581e-01	6.915517e-01	0.9997779456	0.1470143887	0.4773605767	0.90612020	6.561405e-02	0.699540958	0.9982883	-1.197923e-01	0.5280104745
0.77869908	6.442287e-01	1.343660e-01	0.9997779456	-0.3737487319	0.4544441776	0.90612020	6.814177e-01	0.274943482	0.9982883	1.704680e+00	0.2939239858
0.84824569	-9.567592e-02	6.613924e-01	0.9997779456	-0.0090403175	0.9666010178	0.99380888	9.225159e-02	0.594005065	0.9982883	3.818603e-02	0.8291634747
0.97273515	-8.512881e-01	8.256506e-01	0.9997779456	-0.1679634389	0.6663784195	0.94105053	2.895450e-01	0.528584426	0.9982883	-3.413358e-01	0.4319305358
0.97273515	4.176043e-02	4.053753e-01	0.9997779456	0.1423628579	0.6238515715	0.93459133	-9.012068e-02	0.529823839	0.9982883	-1.849168e-02	0.8832516712
0.98146689	-2.398040e-01	9.997779e-01	0.9997779456	0.0135924881	0.8663151557	0.96707132	8.020206e-03	0.906989813	0.9982883	3.164658e-02	0.7382473521
0.80820047	-1.028395e-01	7.570120e-01	0.9997779456	-0.5172211342	0.0005597000	0.09450935	-9.900607e-02	0.354332293	0.9982883	-5.565769e-03	0.9738337575
0.77869908	-1.280114e-01	8.601207e-01	0.9997779456	0.0330350395	0.7685878964	0.95505354	7.590974e-02	0.462187635	0.9982883	1.192081e-02	0.8742949713
0.97273515	-1.109810e-01	8.887281e-01	0.9997779456	0.0069682558	0.9341875389	0.98590149	2.694355e-03	0.970697999	0.9986411	-6.829363e-02	0.3988025769
0.97273515	-1.311218e-01	8.161497e-01	0.9997779456	-0.1998720288	0.0590793614	0.83807199	-5.763255e-02	0.559083781	0.9982883	-2.830365e-01	0.0205657081
0.82862325	1.369379e-01	1.226023e-01	0.9997779456	-0.0990708506	0.4399498934	0.90612020	-5.962783e-02	0.538273259	0.9982883	2.590984e-01	0.0774015726
0.98787269	-6.454331e-02	7.070041e-01	0.9997779456	-0.1311540707	0.2868959906	0.84915316	-7.034682e-02	0.488847494	0.9982883	6.166010e-03	0.9687311266
0.77869908	-6.661297e-02	7.887431e-01	0.9997779456	-0.0809763016	0.5345662576	0.90627295	7.680960e-03	0.897947802	0.9982883	-5.555233e-02	0.5255042835
0.83140044	1.085879e-01	1.526410e-01	0.9997779456	-0.0985184276	0.4514063915	0.90612020	-7.502310e-02	0.403543031	0.9982883	-3.416707e-03	0.9832927200
0.82743594	1.025038e-02	4.651982e-01	0.9997779456	-0.0556820567	0.6359096382	0.93852299	-6.687821e-02	0.580796618	0.9982883	1.587453e-01	0.3898316880
0.89369078	1.498907e-01	1.652000e-02	0.9997779456	-0.0553104500	0.5984060896	0.92580628	-3.304116e-02	0.596481751	0.9982883	5.369804e-03	0.9488601288
0.79578964	3.208314e-01	3.422845e-01	0.9997779456	0.3951758025	0.6047929288	0.93042774	4.853642e-01	0.504081653	0.9982883	2.738007e-01	0.7599236533
0.98996967	-8.161223e-02	6.902569e-01	0.9997779456	0.0326712700	0.9336430665	0.98590149	-2.271995e-01	0.205787569	0.9982883	-2.149417e-02	0.9194742869
0.77869908	-9.583871e-02	8.296526e-01	0.9997779456	-0.2148693927	0.0633735707	0.83807199	-1.728540e-02	0.824631444	0.9982883	2.363936e-02	0.8068759656
0.86909435	-1.373133e-01	7.938299e-01	0.9997779456	0.1059440991	0.6651293364	0.94041014	-2.687776e-02	0.837731887	0.9982883	-1.192319e-01	0.3614378202
0.77869908	3.107261e-02	4.227682e-01	0.9997779456	0.0796275145	0.5671899318	0.92090453	-5.414209e-02	0.659811632	0.9982883	5.822912e-02	0.5857833678
0.97144018	1.144365e-01	4.812606e-02	0.9997779456	0.0040768273	0.9663136295	0.99380888	-1.932788e-02	0.718884024	0.9982883	-1.538876e-01	0.0507740219
0.83725376	-2.446321e-02	6.270702e-01	0.9997779456	0.0103749817	0.9237660531	0.98290228	-4.274931e-02	0.486927009	0.9982883	-3.271450e-02	0.6948540142
0.94990176	-3.640902e-01	9.377831e-01	0.9997779456	-0.0541034796	0.8890241245	0.97246977	-3.494639e-01	0.105971568	0.9982883	-2.154986e-01	0.4907553909
0.77869908	-3.470420e-03	5.213455e-01	0.9997779456	-0.0955651160	0.1831999347	0.83807199	-5.684032e-03	0.922109452	0.9982883	9.510589e-02	0.2172379628
0.79972808	-2.579225e-02	6.401282e-01	0.9997779456	-0.0402333917	0.5943156874	0.92532140	-2.056525e-02	0.729091719	0.9982883	2.565046e-02	0.7558828170
0.77869908	-1.194243e-01	6.421561e-01	0.9997779456	0.3280107638	0.3927217716	0.87916124	1.662633e-01	0.545284812	0.9982883	-1.732156e-01	0.5865668443
0.92890486	-2.988246e-01	9.822646e-01	0.9997779456	-0.1288391470	0.3071326979	0.84915316	-3.089364e-02	0.821574961	0.9982883	-1.369672e-01	0.5759964710
0.77869908	4.111272e-01	2.006762e-01	0.9997779456	0.1315372180	0.7288939782	0.94712279	-1.093095e-01	0.854987532	0.9982883	6.960240e-02	0.8943969978
0.77869908	8.988083e-02	4.416899e-01	0.9997779456	0.1961142415	0.6794771879	0.94316618	-1.469776e-01	0.822109585	0.9982883	1.380981e-01	0.8382961784
0.84824569	-1.260412e-01	9.284162e-01	0.9997779456	-0.2370230241	0.0263547013	0.64586404	-1.895444e-02	0.803150345	0.9982883	1.518264e-02	0.8596108166
0.87099999	-4.743149e-02	6.786186e-01	0.9997779456	0.2104403970	0.2995684418	0.84915316	2.301016e-01	0.012507269	0.9982883	-1.582498e-01	0.2270896065
0.80141971	-7.627341e-02	7.573125e-01	0.9997779456	-0.0681163201	0.4412069667	0.90612020	-7.059950e-02	0.368199654	0.9982883	5.181870e-02	0.6740231905
0.87292023	-1.835677e-01	9.919770e-01	0.9997779456	-0.1675316234	0.1889516842	0.83807199	-1.429748e-01	0.107254354	0.9982883	-6.981987e-02	0.4378979281
0.96804147	1.389894e-01	1.177163e-01	0.9997779456	0.1019305655	0.4505485651	0.90612020	-8.832058e-02	0.517612379	0.9982883	2.826748e-01	0.0632454550
0.77869908	4.820868e-02	2.742593e-01	0.9997779456	-0.0791831053	0.4882621725	0.90612020	-4.327210e-02	0.626933323	0.9982883	1.759379e-01	0.1176316675
0.80653217	3.017984e-01	2.654869e-01	0.9997779456	0.7584027968	0.1723680993	0.83807199	1.979218e-01	0.683635742	0.9982883	7.585789e-01	0.1640558408
0.92890486	2.019624e-02	3.687654e-01	0.9997779456	-0.0156305447	0.8803902844	0.96947881	-1.574430e-02	0.820191796	0.9982883	1.366759e-01	0.1792183878
0.77869908	9.552463e-02	9.606497e-02	0.9997779456	-0.0144100923	0.7899206798	0.95972154	-6.035293e-03	0.919996507	0.9982883	1.202252e-01	0.1373317226
0.98787269	-6.295115e-03	5.153406e-01	0.9997779456	-0.2027608932	0.2130247373	0.83807199	-1.504952e-01	0.251412804	0.9982883	2.054128e-01	0.1756543218
0.80653217	-4.851686e-02	6.711186e-01	0.9997779456	-0.2149744509	0.2141317795	0.83807199	-1.706007e-01	0.103213978	0.9982883	6.533040e-03	0.9520394557
0.97273515	1.498762e-01	8.085700e-02	0.9997779456	-0.1170864321	0.4226937233	0.90022339	-1.395739e-01	0.189879654	0.9982883	5.719612e-03	0.9675725089
0.84824569	1.207852e-01	8.083795e-02	0.9997779456	0.0565058023	0.7138182284	0.94482995	-1.837988e-01	0.064157147	0.9982883	-5.831251e-02	0.6412458472
0.92598653	-1.031997e-01	8.401356e-01	0.9997779456	-0.1557731396	0.3721553108	0.87664366	-8.033393e-02	0.349930744	0.9982883	-6.201659e-02	0.6235018485
0.77869908	3.715034e-01	2.005339e-01	0.9997779456	0.4074848365	0.4721588210	0.90612020	2.419452e-01	0.602861722	0.9982883	9.058814e-02	0.8846951833
0.77869908	-3.915654e-03	5.172643e-01	0.9997779456	-0.1287136614	0.3141597633	0.84915316	-3.319264e-02	0.656204817	0.9982883	5.264240e-03	0.9613950539
0.77869908	1.339978e-01	1.345813e-01	0.9997779456	-0.0360414458	0.7610092467	0.95505354	5.813593e-02	0.588405580	0.9982883	1.633401e-01	0.0869076461
0.97050390	5.265689e-02	4.687871e-01	0.9997779456	-0.5930254337	0.3995093133	0.88596624	7.418956e-01	0.194463376	0.9982883	1.050602e+00	0.3723487330
0.77869908	1.794930e-02	4.398041e-01	0.9997779456	-0.1084028584	0.6040070491	0.93042774	-2.011454e-02	0.852824517	0.9982883	-2.836525e-01	0.0142178293
0.77869908	-5.660928e-02	6.362709e-01	0.9997779456	-0.2023552737	0.5507570327	0.91608209	-1.196216e-01	0.413161119	0.9982883	1.732867e-01	0.5207248626
0.87099999	-7.065839e-01	8.906068e-01	0.9997779456	-0.2704018364	0.3054454391	0.84915316	-4.607199e-01	0.330062010	0.9982883	-7.295662e-01	0.0254653437
0.86909435	-7.728710e-02	8.974167e-01	0.9997779456	-0.0663356120	0.6162561113	0.93239494	2.699307e-02	0.666508407	0.9982883	2.047238e-02	0.8529731228
0.89369078	-6.173435e-02	7.096022e-01	0.9997779456	0.1302995988	0.5896027909	0.92532140	-1.051414e-02	0.926499404	0.9982883	-3.362376e-03	0.9817826902
0.87099999	7.913041e-02	2.622362e-01	0.9997779456	-0.0165643436	0.9101405206	0.97964668	8.083752e-02	0.490818300	0.9982883	1.982400e-01	0.1610259444
0.88203342	-2.112552e-03	5.073106e-01	0.9997779456	0.1016924971	0.5310768119	0.90612020	6.581465e-02	0.603656212	0.9982883	-2.558598e-01	0.0189120794
0.86909435	9.070897e-02	3.336300e-01	0.9997779456	-0.0167220251	0.9409306891	0.98685011	1.347186e-01	0.431061545	0.9982883	6.923783e-02	0.7881671179
0.79578964	8.629491e-02	3.139333e-01	0.9997779456	0.1753269182	0.4934482202	0.90612020	8.545930e-02	0.589546119	0.9982883	1.462340e-01	0.4815292884
0.97273515	4.000208e-01	1.357169e-01	0.9997779456	0.9656503504	0.3564974509	0.87404187	3.912601e-01	0.290263663	0.9982883	-4.241609e-01	0.1896978514
0.97185522	-3.730570e-01	7.986763e-01	0.9997779456	0.6720452180	0.4277816087	0.90454000	7.195289e-01	0.080380398	0.9982883	-7.707606e-01	0.0654128740
0.86909435	-3.946944e-01	9.743045e-01	0.9997779456	-0.3747740618	0.2570798603	0.84915316	4.570817e-02	0.851485164	0.9982883	2.504438e-01	0.2793985870
0.77869908	1.226842e-03	4.981679e-01	0.9997779456	0.1093266927	0.6551873901	0.93854411	1.114232e-01	0.606105169	0.9982883	1.072481e-01	0.5981996431
0.97144018	-3.012600e-01	9.798060e-01	0.9997779456	-0.0618251398	0.7598679476	0.95505354	1.099591e-02	0.928924398	0.9986411	-1.560813e-01	0.3795951885
0.88636459	4.540863e-01	1.691484e-01	0.9997779456	0.7812639174	0.2009992394	0.83807199	4.419651e-01	0.301098506	0.9982883	3.029599e-01	0.5076908402
0.97144018	1.270302e-01	3.865846e-01	0.9997779456	0.4104558048	0.6064897064	0.93042774	-5.168251e-01	0.318686800	0.9982883	1.231499e+00	0.1699320601
0.82862325	1.371881e-02	4.682035e-01	0.9997779456	0.0078811876	0.9699474495	0.99380888	1.002808e-01	0.524184706	0.9982883	1.021201e-01	0.6606640195
0.97273515	-1.978701e-01	8.877424e-01	0.9997779456	0.0071979230	0.9724011059	0.99380888	-1.379031e-01	0.330504676	0.9982883	-3.533642e-01	0.0140805218
0.77869908	1.278499e-01	4.165356e-01	0.9997779456	0.5257834530	0.4814481978	0.90612020	1.182437e-01	0.847655413	0.9982883	3.059643e-01	0.6782866510
0.90581107	1.274530e-02	4.611558e-01	0.9997779456	-0.2233952226	0.2139651037	0.83807199	-3.634781e-02	0.698730718	0.9982883	3.790499e-02	0.7739240941
0.96804147	-2.617582e-01	9.501754e-01	0.9997779456	-0.1065055523	0.5978688226	0.92580628	1.842369e-01	0.290849489	0.9982883	2.427995e-01	0.0521905318
0.77869908	2.955225e-02	3.733309e-01	0.9997779456	-0.2536392085	0.0735593489	0.83807199	-1.052848e-01	0.372245422	0.9982883	5.665628e-03	0.9632579129
0.89273364	-6.502852e-02	6.999745e-01	0.9997779456	-0.2339270589	0.0187417935	0.58296842	-1.210865e-01	0.248061328	0.9982883	4.494977e-02	0.7732702742
0.77869908	2.545151e-01	3.103978e-01	0.9997779456	0.2763018324	0.5413250763	0.91146188	-4.353355e-01	0.555544871	0.9982883	-6.391009e-01	0.2160676111
0.95134638	1.782993e-02	4.047039e-01	0.9997779456	0.0410893618	0.6568312137	0.93854411	2.711889e-02	0.683194891	0.9982883	1.024312e-01	0.2821470831

0.79654908	-7.892344e-02	6.747032e-01	0.9997779456	0.3562621406	0.3008634964	0.84915316	-7.066482e-02	0.665926585	0.9982883	5.099822e-02	0.8040153138
0.95558280	-4.414224e-02	6.184597e-01	0.9997779456	-0.1648882888	0.3032098521	0.84915316	-4.346049e-02	0.683860919	0.9982883	-2.058089e-02	0.8428225918
0.99525047	-1.032476e-01	7.491318e-01	0.9997779456	0.2264457432	0.2275982299	0.83807199	-8.214870e-03	0.947494820	0.9986411	-2.375903e-01	0.1026152586
0.87292023	1.010931e-01	1.207016e-01	0.9997779456	-0.1503447287	0.3535727774	0.87067296	-4.422824e-02	0.593408412	0.9982883	2.126344e-01	0.1087475193
0.80820047	-2.716338e-02	6.178835e-01	0.9997779456	0.0023585648	0.9892064070	0.99380888	7.527332e-03	0.944204293	0.9986411	-2.265393e-01	0.0863852825
0.94189574	-2.011515e-01	9.332608e-01	0.9997779456	0.3287807858	0.1301756073	0.83807199	-7.969098e-02	0.539429454	0.9982883	-1.973141e-01	0.3141996172
0.77869908	-7.409303e-02	8.645453e-01	0.9997779456	-0.0268012989	0.8152122314	0.95972154	-5.676154e-02	0.510067502	0.9982883	1.089908e-01	0.3688782427
0.97273515	-2.455147e-01	9.410772e-01	0.9997779456	-0.2897324566	0.0255951586	0.64586404	-7.183318e-02	0.501919835	0.9982883	-2.264866e-01	0.0781863211
0.80820047	-8.153891e-02	5.578187e-01	0.9997779456	0.2532677298	0.6290158640	0.93786229	8.920132e-02	0.855253375	0.9982883	-1.898005e-01	0.7273472996
0.95639607	9.671410e-02	3.902252e-01	0.9997779456	-0.2807813966	0.4083165630	0.89501894	3.251555e-01	0.240637228	0.9982883	-1.529044e-01	0.7380908593
0.89609137	5.121638e-02	3.876655e-01	0.9997779456	-0.1068995429	0.5857224663	0.92532140	-1.471973e-01	0.431783425	0.9982883	-8.271882e-02	0.7258426262
0.99066584	-5.316529e-01	9.612178e-01	0.9997779456	-0.1184643367	0.6038414288	0.93042774	-3.452441e-02	0.879544397	0.9982883	-2.454075e-02	0.9238628395
0.77869908	6.965661e-02	2.802630e-01	0.9997779456	-0.3214070607	0.1709864976	0.83807199	-1.116838e-01	0.289010386	0.9982883	-2.077128e-02	0.8889113068
0.77869908	-5.320640e-03	5.271922e-01	0.9997779456	-0.1470082538	0.1672115236	0.83807199	6.612428e-02	0.345715799	0.9982883	-5.460801e-03	0.9505189071
0.82743594	-1.818222e-01	9.960507e-01	0.9997779456	-0.1962041495	0.1177106166	0.83807199	-2.070222e-02	0.727038652	0.9982883	-1.448412e-01	0.2065400836
0.79972808	7.822693e-02	2.332620e-01	0.9997779456	-0.1410559641	0.4088611853	0.89501894	-1.196938e-01	0.222250898	0.9982883	-3.348956e-03	0.9671181128
0.77869908	-7.254087e-03	5.168026e-01	0.9997779456	-0.4015136543	0.0597877151	0.83807199	-5.480473e-02	0.671389938	0.9982883	9.132655e-02	0.6113165935
0.99436660	-3.532976e-02	5.459424e-01	0.9997779456	0.3493729221	0.3486236269	0.86676800	3.029289e-01	0.179379233	0.9982883	-1.215299e-01	0.6645788437
0.82862325	-7.704147e-02	8.452423e-01	0.9997779456	-0.1417600191	0.1845620644	0.83807199	-3.845873e-02	0.505954200	0.9982883	-8.144885e-02	0.2658730373
0.77869908	1.053424e-01	4.243978e-01	0.9997779456	0.5075456509	0.4111635223	0.89501894	1.551347e-01	0.776344989	0.9982883	2.129184e-02	0.9720452595
0.93924745	-5.261846e-02	6.649831e-01	0.9997779456	-0.5441938395	0.0081318271	0.43863723	-3.776383e-02	0.767641077	0.9982883	1.632486e-01	0.2600348452
0.93506890	-1.379875e-01	7.508071e-01	0.9997779456	-0.1151412730	0.5566943898	0.91608209	1.022292e-01	0.563686833	0.9982883	8.645799e-02	0.7527451496
0.93081046	-1.823586e-01	8.935983e-01	0.9997779456	-0.2166539727	0.1706522063	0.83807199	-4.634984e-02	0.695051967	0.9982883	5.893697e-04	0.9964785151
0.93359368	-1.707705e-01	8.876544e-01	0.9997779456	0.0242879300	0.8708990555	0.96930573	1.616211e-02	0.909086502	0.9982883	-2.927326e-01	0.0632407107
0.79578964	-1.502818e-02	5.127600e-01	0.9997779456	0.5725446215	0.3404173519	0.85977203	2.829295e-01	0.490469531	0.9982883	1.305525e-02	0.9828343674
0.95472383	-1.592700e-01	6.302271e-01	0.9997779456	0.5957236856	0.2152985809	0.83807199	-1.139661e-01	0.780927846	0.9982883	7.999231e-03	0.9767196575
0.88001839	1.155917e-01	9.453376e-02	0.9997779456	0.0384036751	0.7040000698	0.94482995	4.888846e-02	0.585326324	0.9982883	1.490250e-02	0.8872621029
0.79213004	-1.011640e-01	9.017980e-01	0.9997779456	-0.0515702047	0.5702205322	0.92122660	-4.523107e-02	0.500734265	0.9982883	-7.551982e-02	0.3126268128
0.97185522	-7.497082e-02	5.845992e-01	0.9997779456	0.6992276076	0.4369492135	0.90612020	5.090727e-02	0.884646770	0.9982883	-1.410617e-01	0.6477796859
0.93650941	-3.953807e-03	5.184517e-01	0.9997779456	-0.0536437942	0.4080748797	0.89501894	1.104060e-02	0.846706759	0.9982883	5.602857e-02	0.4346863309
0.97273515	6.609477e-02	3.355325e-01	0.9997779456	0.4585000237	0.2508286966	0.84467092	1.113093e-01	0.473601820	0.9982883	-1.945444e-01	0.2136356723
0.99066584	2.160062e-01	1.239422e-01	0.9997779456	0.3235877847	0.2625698506	0.84915316	-2.022102e-01	0.237559791	0.9982883	-4.488592e-01	0.0027274956
0.80521409	-1.231750e-02	5.353053e-01	0.9997779456	-0.2186109193	0.0736460468	0.83807199	3.728700e-02	0.814796124	0.9982883	1.647813e-01	0.3558147331
0.79578964	1.878395e-01	3.822043e-01	0.9997779456	0.2862476211	0.5860909090	0.92532140	-2.305596e-01	0.754681904	0.9982883	-3.741404e-01	0.5650863562
0.83731157	-1.809814e-01	9.739694e-01	0.9997779456	-0.1541597863	0.1850134225	0.83807199	1.947606e-02	0.798701192	0.9982883	-5.077452e-02	0.6779266961
0.97273515	-3.890166e-02	5.853444e-01	0.9997779456	-0.0203638698	0.9105235078	0.97964668	-2.930525e-01	0.081352787	0.9982883	-3.211714e-01	0.0324852210
0.92583146	3.876776e-02	3.497113e-01	0.9997779456	0.0664034707	0.5909611584	0.92532140	-5.363156e-03	0.956471980	0.9986411	2.930434e-01	0.0330201935
0.77869908	-3.000152e-02	5.357242e-01	0.9997779456	-0.1150446451	0.7972246113	0.95972154	2.881505e-03	0.992263862	0.9988193	5.745489e-01	0.2138378635
0.92876201	-2.421661e-01	9.625391e-01	0.9997779456	-0.2340338344	0.1209215204	0.83807199	7.394288e-02	0.534290108	0.9982883	-9.216092e-02	0.4795813270
0.77869908	-3.508810e-02	6.276285e-01	0.9997779456	-0.0831044046	0.6077451593	0.93042774	-8.413025e-02	0.358254151	0.9982883	-1.283012e-01	0.3204547376
0.77869908	5.996328e-02	2.739496e-01	0.9997779456	0.2097109080	0.1016959276	0.83807199	1.757365e-01	0.032849972	0.9982883	-3.869699e-02	0.6458764594
0.86909435	-1.753371e-01	9.072091e-01	0.9997779456	0.1450208936	0.3737972946	0.87664366	-3.652918e-03	0.971628603	0.9986411	-2.703446e-01	0.0183863714
0.98670901	-9.743631e-02	8.347783e-01	0.9997779456	0.0748618254	0.4747702473	0.90612020	1.233743e-01	0.163613896	0.9982883	-1.196591e-01	0.0956522969
0.99066584	-1.208327e-01	7.878818e-01	0.9997779456	-0.0484722983	0.7018298510	0.94482995	7.547304e-03	0.950413679	0.9986411	-9.274263e-02	0.4665035336
0.95284371	1.135124e-01	3.475628e-01	0.9997779456	0.4662760377	0.4984785725	0.90612020	2.529925e-01	0.376570193	0.9982883	-2.741937e-01	0.4768873649
0.94990176	-1.653480e-01	8.755277e-01	0.9997779456	0.0636211367	0.7710942736	0.95505354	-6.134300e-02	0.669689824	0.9982883	8.589498e-02	0.6403630764
0.97273515	-4.523902e-02	6.902634e-01	0.9997779456	0.1076219871	0.1817472303	0.83807199	4.536783e-02	0.611228522	0.9982883	-1.175055e-01	0.1987358664
0.77869908	-2.353939e-02	5.518220e-01	0.9997779456	-0.1236719221	0.7605852945	0.95505354	-1.207077e-01	0.555094074	0.9982883	-1.357682e-01	0.7089569095
0.92226585	1.831410e-01	1.547910e-01	0.9997779456	0.4058036415	0.4051601796	0.89501894	1.644430e-01	0.336041456	0.9982883	2.953104e-02	0.8785413912
0.97273515	2.436487e+00	1.096194e-01	0.9997779456	2.8851388473	0.4138292545	0.89754931	7.134760e-01	0.638557081	0.9982883	-8.687728e-01	0.5484788739
0.77869908	1.249588e-02	4.661047e-01	0.9997779456	-0.0610505111	0.6479086768	0.93854411	-5.733818e-02	0.653723838	0.9982883	1.010959e-01	0.6214183762
0.90482806	-2.341614e-01	9.223343e-01	0.9997779456	-0.4157746165	0.0150350782	0.49365173	-1.332072e-02	0.912112976	0.9982883	-1.937052e-01	0.1618213242
0.80820047	-1.724399e-01	8.801499e-01	0.9997779456	-0.0691367835	0.7271964658	0.94712279	-1.034921e-01	0.409707516	0.9982883	2.111844e-02	0.9001928467
0.97273515	-7.131325e-02	7.257486e-01	0.9997779456	0.0509435717	0.5728621209	0.92122660	-8.991777e-02	0.391863027	0.9982883	-2.997155e-01	0.0532224861
0.77869908	-4.715240e-02	5.329478e-01	0.9997779456	0.5979588421	0.3152354447	0.84915316	3.764183e-01	0.436888913	0.9982883	5.317212e-02	0.9249881899
0.77869908	3.162411e-01	3.439251e-01	0.9997779456	0.0757953397	0.9283455988	0.98501301	4.553276e-01	0.544967135	0.9982883	-4.031548e+00	0.0002432435
0.99066584	-9.128610e-03	5.379096e-01	0.9997779456	0.1323067464	0.3736903265	0.87664366	6.324804e-02	0.535074560	0.9982883	-1.459328e-01	0.2472165460
0.77869908	9.877127e-03	4.620488e-01	0.9997779456	0.1426618528	0.1875745618	0.83807199	1.391927e-01	0.062151659	0.9982883	1.160751e-01	0.2670588213
0.99066584	-5.260426e-02	6.551386e-01	0.9997779456	0.1139727670	0.5173291378	0.90612020	-8.962665e-02	0.372112853	0.9982883	1.652980e-01	0.1880463111
0.77869908	2.300167e-01	1.563280e-01	0.9997779456	0.1383882765	0.6335189962	0.93852299	-2.121872e-01	0.374797695	0.9982883	3.952344e-01	0.2666604548
0.95284371	-2.065118e-01	9.359976e-01	0.9997779456	-0.1686147049	0.4153633441	0.89754931	1.392253e-01	0.294132438	0.9982883	2.181672e-02	0.9237259364
0.97273515	3.891103e-01	4.974975e-02	0.9997779456	-0.0567126118	0.7943273250	0.95972154	-4.234823e-02	0.836022130	0.9982883	3.411501e-02	0.9060163128
0.77869908	2.057230e-01	3.332402e-01	0.9997779456	-0.1723578133	0.6753280381	0.94316618	2.677816e-01	0.542085263	0.9982883	-2.537202e-01	0.6124653953
0.93081046	7.878375e-02	2.303000e-01	0.9997779456	0.2310234645	0.0060204465	0.40915301	2.531409e-02	0.776722622	0.9982883	1.938929e-01	0.0154847912
0.97522657	-4.531305e-01	9.778368e-01	0.9997779456	-0.1312613344	0.5497830356	0.91608209	-2.195307e-02	0.912125687	0.9982883	-8.364388e-02	0.8144968526
0.95284371	1.641864e-02	4.150380e-01	0.9997779456	-0.1717894268	0.0990387256	0.83807199	-1.179094e-01	0.062989530	0.9982883	3.999930e-02	0.6128623019
0.83140044	-4.472525e-02	6.768431e-01	0.9997779456	-0.0667442896	0.5450691222	0.91515867	-3.948850e-02	0.592485975	0.9982883	-4.600228e-02	0.5358883589
0.80653217	-9.918606e-01	8.019069e-01	0.9997779456	1.5504865750	0.3039494464	0.84915316	3.331491e-01	0.698198127	0.9982883	-6.514243e-01	0.5920782216
0.87292023	3.262689e-01	9.949701e-02	0.9997779456	0.4121959249	0.1591274437	0.83807199	1.650647e-02	0.937230571	0.9986411	-7.889732e-02	0.7942012584
0.77869908	-3.044881e-02	6.443675e-01	0.9997779456	-0.0736452342	0.4760902543	0.90612020	3.324332e-02	0.572983254	0.9982883	-2.991556e-02	0.6869213685
0.79972808	-6.421425e-01	8.725215e-01	0.9997779456	-0.2532433447	0.6907351301	0.94482995	-1.356490e+00	0.024849836	0.9982883	1.183800e-01	0.8229575327
0.78828782	6.712595e-02	2.759942e-01	0.9997779456	-0.1194867975	0.1896979732	0.83807199	2.616162e-01	0.012805363	0.9982883	1.297454e-01	0.3387033956
0.89273364	1.923114e-02	4.307394e-01	0.9997779456	-0.2063765299	0.1409283996	0.83807199	-1.534808e-01	0.059599466	0.9982883	5.799244e-02	0.6463002798
0.77869908	1.153482e-01	1.336511e-01	0.9997779456	-0.1840682314	0.1333494749	0.83807199	-6.261894e-02	0.504169651	0.9982883	1.510520e-01	0.2604629647
0.87099999	-9.215610e-01	9.523750e-01	0.9997779456	-0.4368807056	0.5814162335	0.92532140	-4.597019e-01	0.417381244	0.9982883	-1.910388e+00	0.0166878568
0.97522657	6.760331e-01	1.357870e-01	0.9997779456	-0.7623886100	0.1757962970	0.83807199	5.777776e-01	0.350216336	0.9982883	-4.609762e-01	0.4072618460
0.81837375	6.278082e-02	4.317440e-01	0.9997779456	0.6520321114	0.1987898336	0.83807199	1.090873e-01	0.786834969	0.9982883	-2.560746e-01	0.6008447574
0.77869908	1.364684e-01	8.281682e-02	0.9997779456	0.0191601302	0.8206045052	0.95972154	-2.400503e-02	0.761879598	0.9982883	9.574426e-02	0.2655868284
0.97050390	-3.363817e-02	6.293449e-01	0.9997779456	-0.0716965518	0.3360409344	0.85880449	-5.022862e-02	0.496254279	0.9982883	2.555604e-03	0.9781805228
0.88505393	1.425517e-01	1.355929e-01	0.9997779456	0.0519902751	0.7132777121	0.94482995	-1.798332e-01	0.217928586	0.9982883	-8.913273e-03	0.9525839507
0.97522657	-2.055683e-01	9.719437e-01	0.9997779456	0.0351262778	0.8331517274	0.96288315	-4.533785e-03	0.966114069	0.9986411	3.999731e-02	0.7894546085
0.90897285	9.733017e-02	1.382671e-01	0.9997779456	-0.0233712594	0.8355020279	0.96321135	-8.263320e-02	0.291361733	0.9982883	-3.834790e-02	0.6917531349
0.77869908	-5.288606e-02	7.050968e-01	0.9997779456	-0.1321167812	0.2256528546	0.83807199	-1.311908e-01	0.106776300	0.9982883	-2.136310e-02	0.8167932621
0.97050390	-2.158223e-01	6.821326e-01	0.9997779456	0.4671195434	0.5088745017	0.90612020	-3.652281e-01	0.432086418	0.9982883	-2.613609e-01	0.6132513182
0.95472383	-4.235799e-01	9.631945e-01	0.9997779456	-0.1928489660	0.1413742258	0.83807199	1.359760e-01	0.331824357	0.9982883	-4.967929e-01	0.0013306676
0.94675587	4.255223e-02	2.358393e-01	0.9997779456	-0.0001083892	0.9989540475	0.99895405	-4.572120e-02	0.469296413	0.9982883	-8.816727e-02	0.1985174518
0.96804147	-9.737097e-02	8.211434e-01	0.9997779456	0.0363095244	0.5732715563	0.92122660	-1.048539e-01	0.312973682	0.9982883	-1.441529e-01	0.1210774992
0.89273364	-7.697307e-02	7.943288e-01	0.9997779456	-0.1020499250	0.5288915292	0.90612020	-3.619223e-03	0.971230494	0.9986411	-7.972025e-02	0.5179231952
0.80925874	4.925508e-02	3.865049e-01	0.9997779456	-0.1287291190	0.5556668339	0.91608209	-7.541681e-03	0.963440389	0.9986411	1.796269e-01	0.5501497695
0.88636459	-7.492279e-01	9.991978e-01	0.9997779456	-0.6136206193	0.0083411331	0.43863723	-1.009253e-01	0.683823024	0.9982883	3.350319e-01	0.2433201401
0.96804147	2.955167e-03	4.905411e-01	0.9997779456	-0.0355311544	0.8401615109	0.96321135	-1.376564e-01	0.296909578	0.9982883	9.135216e-03	0.9497604559
0.81163165	-2.292919e-01	7.801710e-01	0.9997779456	-0.7845955763	0.0095075699	0.45854692	-1.286553e-01	0.508830179	0.9982883	1.629930e-01	0.3777178517
0.77869908	1.161243e-01	1.889528e-01	0.9997779456	0.0039161705	0.9724519648	0.99380888	-1.745223e-02	0.889436811	0.9982883	1.543018e-01	0.2282601449
0.77869908	1.072880e-02	4.578231e-01	0.9997779456	-0.0341409982	0.7234145982	0.94712279	8.298398e-02	0.368572290	0.9982883	4.172671e-03	0.9697905077
0.89911801	2.163339e-01	1.050029e-01	0.9997779456	0.4107927760	0.3987726388	0.88596624	1.214287e-01	0.468181033	0.9982883	-1.432177e-01	0.3289984523
0.97522657	-1.026994e-01	6.426195e-01	0.9997779456	0.0506368053	0.8802356181	0.96947881	-1.556870e-01	0.493529712	0.9982883	-6.432699e-02	0.7616198959
0.99018039	-7.868742e-02	7.504129e-01	0.9997779456	0.2385857432	0.3495194905	0.86676800	1.217272e-01	0.342335500	0.9982883	-1.237473e-01	0.3016257521
0.77869908	-9.380995e-02	8.157910e-01	0.9997779456	-0.0385177092	0.7355826711	0.94712279	-3.292686e-02	0.693377326	0.9982883	4.721132e-02	0.7053396537
0.95134638	2.389323e-02	4.097147e-01	0.9997779456	-0.1810929023	0.2044516959	0.83807199	1.027651e-01	0.315889062	0.9982883	1.076062e-01	0.4601228789
0.84600172	1.062479e-01	1.031159e-01	0.9997779456	-0.0600806695	0.4910350386	0.90612020	5.405952e-02	0.525413367	0.9982883	-1.115871e-01	0.2065591482
0.77869908	4.821168e-02	2.554515e-01	0.9997779456	-0.1562917525	0.0667481209	0.83807199	-5.364660e-02	0.397580663	0.9982883	9.433163e-02	0.2709703927
0.96780790	1.317348e-01	2.399166e-01	0.9997779456	-0.2825270589	0.1525801579	0.83807199	-1.629520e-01	0.245537807	0.9982883	-1.235760e-01	0.4425830276
0.87292023	1.458312e-01	1.355492e-01	0.9997779456	-0.0531331444	0.7724460105	0.95505354	7.752693e-02	0.506581183	0.9982883	-6.874836e-02	0.6738062113
0.90581107	3.260621e-02	3.674576e-01	0.9997779456	-0.0323142341	0.7899949980	0.95972154	-5.108831e-02	0.522672976	0.9982883	5.145745e-02	0.6023544875
0.77869908	4.403618e-01	6.131951e-02	0.9997779456	0.0275915628	0.8978534027	0.97363552	-5.286163e-02	0.875671198	0.9982883	4.545396e-02	0.8272471884
0.88636459	-2.011675e-01	8.377626e-01	0.9997779456	-0.1496628586	0.5567783798	0.91608209	-2.481523e-02	0.874914625	0.9982883	-2.070525e-01	0.4146025867
0.77869908	-3.022249e-01	8.769793e-01	0.9997779456	-0.5129753394	0.1146785928	0.83807199	-9.479174e-02	0.688247983	0.9982883	-1.528768e-01	0.3576166080
0.77869908	-8.019694e-02	5.602663e-01	0.9997779456	0.4361234654	0.4438767705	0.90612020	2.163314e-01	0.653021204	0.9982883	-3.470026e-01	0.5044052480
0.81804251	-3.854195e-02	5.683550e-01	0.9997779456	-0.8191405148	0.0096985390	0.45854692	-2.113933e-01	0.370357058	0.9982883	3.224049e-01	0.2794362233
0.84600172	-1.703890e-01	7.075189e-01	0.9997779456	-0.5278601778	0.1123673722	0.83807199	-5.114868e-01	0.043425308	0.9982883	-1.744083e-02	0.9660750008
0.90581107	2.853401e-02	3.898353e-01	0.9997779456	-0.0327471535	0.6930479666	0.94482995	-1.774720e-03	0.982898152	0.9986411	-4.228809e-02	0.7350809136
0.98996967	7.882191e-02	4.279641e-01	0.9997779456	-0.6126357623	0.1965457632	0.83807199	-2.416067e-01	0.547054485	0.9982883	-1.239204e+00	0.0072319008
0.77869908	-3.053858e-02	5.967945e-01	0.9997779456	-0.2615804834	0.0213952533	0.60526393	5.161213e-02	0.533126386	0.9982883	1.029202e-01	0.2369028824
0.91967042	-1.677485e+00	9.740665e-01	0.9997779456	-0.6929869449	0.1502547550	0.83807199	-4.135534e-01	0.526950231	0.9982883	-1.076641e+00	0.0216070309
0.92891556	-6.382193e-02	6.830142e-01	0.9997779456	-0.1879556545	0.1997670714	0.83807199	-1.720183e-02	0.881200497	0.9982883	-2.687390e-02	0.7446047001
0.77869908	1.212854e-01	1.445400e-01	0.9997779456	-0.2212037364	0.1077365488	0.83807199	-9.455959e-02	0.305321040	0.9982883	2.198894e-01	0.0283526897
0.77869908	1.167494e-02	4.456171e-01	0.9997779456	0.0242814124	0.8666492918	0.96707132	1.032665e-01	0.212502982	0.9982883	-5.089365e-02	0.6720474357
0.77869908	-6.912848e-02	7.523637e-01	0.9997779456	-0.0242986580	0.8071315770	0.95972154	1.094502e-02	0.889984256	0.9982883	1.062102e-01	0.2637054434
0.83725376	4.001937e-02	4.541065e-01	0.9997779456	-0.3193378422	0.2719018714	0.84915316	2.448296e-01	0.312864739	0.9982883	5.183112e-01	0.1585648006
0.94464659	-3.913456e-01	9.453663e-01	0.9997779456	-0.0780048044	0.6607420430	0.93982803	-3.355277e-01	0.068261824	0.9982883	-5.786330e-01	0.0029843441
0.96804147	-2.325189e-01	7.069771e-01	0.9997779456	-0.0432925187	0.9272147031	0.98469702	5.667705e-01	0.110272600	0.9982883	4.308591e-01	0.2123261581
0.90482806	-1.643819e-01	8.752275e-01	0.9997779456	-0.0193039960	0.9430492485	0.98742107	4.681202e-02	0.732357299	0.9982883	-4.123645e-01	0.0682739203
0.97050390	-1.692761e-01	9.072543e-01	0.9997779456	-0.1178540604	0.2120373167	0.83807199	-1.628446e-02	0.870554861	0.9982883	-4.805217e-02	0.6730683909
0.77869908	2.702146e-01	9.482151e-02	0.9997779456	-0.3526248179	0.0389252103	0.76504055	1.947788e-01	0.228198752	0.9982883	1.370121e-01	0.4676452019
0.92117830	5.625333e-01	8.751456e-02	0.9997779456	0.1621753794	0.7582926925	0.95505354	3.662670e-01	0.310033244	0.9982883	2.455047e-01	0.5733752411
0.83237211	6.722522e-02	2.713229e-01	0.9997779456	-0.1051815603	0.5294916519	0.90612020	5.418036e-02	0.637517588	0.9982883	1.687093e-01	0.1374127234
0.77869908	-1.635772e-01	6.892744e-01	0.9997779456	0.2099974561	0.6721852509	0.94130886	2.082913e-01	0.416742080	0.9982883	-3.738688e-01	0.1452700109
0.95134638	3.722968e-02	4.028911e-01	0.9997779456	0.2174114988	0.1291405148	0.83807199	1.535801e-01	0.276899838	0.9982883	1.365129e-01	0.3982926151
0.79578964	-4.642792e-01	6.078765e-01	0.9997779456	1.3219707197	0.5253013915	0.90612020	-5.523883e-01	0.770186826	0.9982883	-3.449946e+00	0.1084262392
0.98996967	-1.328851e-01	6.740156e-01	0.9997779456	0.1174718730	0.7268078426	0.94712279	-2.925151e-02	0.894765266	0.9982883	-5.508244e-01	0.0480394723
0.77869908	-2.938374e-02	5.969213e-01	0.9997779456	-0.0018934913	0.9904457388	0.99380888	5.054170e-02	0.670382453	0.9982883	-3.822855e-02	0.7745202499
0.96804147	7.084514e-02	3.045440e-01	0.9997779456	0.2205045113	0.1756680330	0.83807199	2.977520e-02	0.802432186	0.9982883	2.388793e-01	0.0618456042
0.80820047	4.273531e-02	3.119813e-01	0.9997779456	0.0977472712	0.0620862964	0.83807199	-4.121228e-02	0.575108552	0.9982883	3.520529e-02	0.7255616880
0.96804147	-1.441544e-01	6.188114e-01	0.9997779456	0.4997189685	0.3935918676	0.87944345	-3.305018e-01	0.458498942	0.9982883	-1.200479e+00	0.0007368967
0.99066584	-1.177731e-01	7.581819e-01	0.9997779456	0.1413104344	0.3628646990	0.87404187	-8.093997e-02	0.538913049	0.9982883	-3.301987e-01	0.0571531661
0.79578964	-4.638080e-02	6.947252e-01	0.9997779456	-0.1434270656	0.1974221304	0.83807199	-1.036005e-01	0.100364702	0.9982883	-1.435838e-01	0.2670039507
0.98226159	5.132920e-02	4.095343e-01	0.9997779456	0.6789343462	0.3343376720	0.85880449	1.339429e-01	0.580295271	0.9982883	2.719076e-02	0.8929066044
0.84824569	4.624178e-02	3.254353e-01	0.9997779456	-0.0617715315	0.5890184172	0.92532140	-2.859003e-02	0.753173227	0.9982883	1.221585e-01	0.1671304421
0.77869908	-7.835721e-02	7.132594e-01	0.9997779456	-0.0187497987	0.8934678981	0.97246977	-1.804423e-02	0.896576374	0.9982883	3.195408e-01	0.0588326478

0.87292023	1.103506e+00	4.441280e-02	0.9997779456	0.5860612904	0.5530985488	0.91608209	-2.899083e-01	0.599905130	0.9982883	-2.758079e-01	0.6851149486
0.95472383	-2.684603e-01	9.954653e-01	0.9997779456	-0.2158721626	0.0926917431	0.83807199	-1.459507e-01	0.136330164	0.9982883	-1.148560e-01	0.2079350678
0.94189574	3.246966e-02	3.327093e-01	0.9997779456	0.0016957963	0.9845634423	0.99380888	9.959950e-02	0.125952916	0.9982883	-2.109453e-03	0.9817771477
0.77869908	-6.607550e-01	8.142061e-01	0.9997779456	-0.7103077370	0.0871876743	0.83807199	2.924159e-01	0.576396939	0.9982883	-9.493024e-01	0.0321460303
0.81163165	1.033649e+00	7.608595e-02	0.9997779456	-0.0334147499	0.9703298766	0.99380888	-1.794442e-01	0.780105085	0.9982883	8.623673e-01	0.3404724318
0.79578964	6.722598e-03	4.679957e-01	0.9997779456	-0.1217604056	0.1999443960	0.83807199	-9.865114e-02	0.175308163	0.9982883	1.033578e-01	0.2574331839
0.80820047	-6.491697e-02	7.432198e-01	0.9997779456	-0.1971848864	0.1568003365	0.83807199	-1.601294e-01	0.086331273	0.9982883	8.644454e-02	0.1991406498
0.99018039	7.484939e-02	4.408123e-01	0.9997779456	0.2406439042	0.6712201908	0.94130886	2.855644e-01	0.491845005	0.9982883	2.329859e-01	0.7211141334
0.86909435	-9.834408e-02	5.775464e-01	0.9997779456	-0.0462416339	0.9388429769	0.98648257	-5.408206e-02	0.908945833	0.9982883	6.522795e-01	0.2338800301
0.77869908	5.691283e-02	2.589802e-01	0.9997779456	-0.0561776489	0.6313278017	0.93852299	-4.734841e-02	0.589977383	0.9982883	-3.886155e-02	0.7868751553
0.97273515	1.544000e-01	3.688571e-01	0.9997779456	1.0574956424	0.3873040119	0.87916124	2.848100e-02	0.948326516	0.9986411	-7.358562e-01	0.0959929542
0.87292023	7.121100e-02	3.785595e-01	0.9997779456	0.5956065299	0.1615955764	0.83807199	1.998202e-01	0.341102243	0.9982883	3.785328e-01	0.1609685268
0.97273515	2.327913e+00	1.685881e-01	0.9997779456	4.8342526512	0.3039158208	0.84915316	3.447839e+00	0.261446490	0.9982883	-2.999333e+00	0.2719219638
0.97273515	3.326934e-01	2.573650e-01	0.9997779456	0.7745498411	0.2866287453	0.84915316	-1.715209e-01	0.769776684	0.9982883	-6.452257e-04	0.9990098920
0.92876201	-4.386273e-02	5.499140e-01	0.9997779456	0.6603432066	0.2087690732	0.83807199	2.541251e-01	0.340975929	0.9982883	2.795252e-01	0.5752222021
0.77869908	-2.919675e-01	9.424523e-01	0.9997779456	-0.3136336261	0.3493222378	0.86676800	3.038757e-03	0.986241818	0.9986411	-1.218355e-01	0.6013277059
0.90482806	5.875552e-02	3.680811e-01	0.9997779456	-0.0321512646	0.8214732472	0.95972154	1.142180e-02	0.938328458	0.9986411	1.733222e-01	0.2877293900
0.97100315	-6.566192e-02	7.029274e-01	0.9997779456	-0.1496728795	0.2018569712	0.83807199	5.826863e-02	0.522503477	0.9982883	-2.040154e-02	0.8227128617
0.97273515	-1.285566e-01	6.705362e-01	0.9997779456	0.0626746029	0.8563215860	0.96707132	-1.682673e-01	0.465753440	0.9982883	-5.731396e-01	0.0224096743
0.90802583	-2.236311e-01	5.684110e-01	0.9997779456	0.6464936302	0.5755664281	0.92184217	7.464306e-01	0.454184866	0.9982883	-2.599152e-01	0.8274669882
0.77869908	-8.462321e-02	7.457405e-01	0.9997779456	-0.1117349763	0.4712189379	0.90612020	-8.227893e-02	0.526379382	0.9982883	1.514101e-01	0.2955851520
0.82862325	1.949417e-01	1.248809e-01	0.9997779456	0.0737759868	0.7571226736	0.95505354	1.456281e-01	0.255740099	0.9982883	1.789438e-01	0.4034565635
0.80820047	1.470664e-01	2.997005e-01	0.9997779456	0.3984045469	0.1763296393	0.83807199	2.006091e-01	0.439376427	0.9982883	4.308280e-02	0.9116735084
0.87292023	-9.067975e-02	8.841726e-01	0.9997779456	-0.1192570152	0.1947390440	0.83807199	-4.496532e-02	0.482387722	0.9982883	-5.335338e-02	0.5509547707
0.84824569	1.539381e-01	4.172555e-02	0.9997779456	0.0496679926	0.5567047504	0.91608209	-5.584007e-02	0.496476017	0.9982883	6.188644e-02	0.6337832356
0.96804147	8.436086e-01	4.605888e-05	0.0272208001	0.2846260807	0.4731926780	0.90612020	2.460614e-01	0.238931034	0.9982883	6.278445e-01	0.0205171855
0.88636459	4.071480e-02	4.541797e-01	0.9997779456	0.3624943899	0.4419811840	0.90612020	-2.764797e-01	0.416786339	0.9982883	-5.312446e-01	0.4096279551
0.90482806	-2.073090e-01	8.827284e-01	0.9997779456	-0.2777218913	0.0943887787	0.83807199	1.068482e-01	0.503606012	0.9982883	-2.474524e-01	0.1937535193
0.79578964	-4.498631e-02	6.702177e-01	0.9997779456	-0.0207110614	0.8949252125	0.97246977	-1.074547e-01	0.257114356	0.9982883	-3.827736e-03	0.9730335381
0.82862325	5.880176e-01	4.105163e-02	0.9997779456	-0.4191352549	0.1722316020	0.83807199	-6.888757e-01	0.177088700	0.9982883	-4.979046e-01	0.1563859546
0.93924745	1.919991e-01	2.486964e-01	0.9997779456	0.4413398153	0.4374753361	0.90612020	6.430340e-01	0.030751818	0.9982883	-3.654664e-01	0.1516528090
0.83725376	1.136255e-01	2.190580e-01	0.9997779456	0.1174737196	0.5029799409	0.90612020	-3.233608e-02	0.766358046	0.9982883	-1.056722e-01	0.5218780409
0.97273515	4.380077e-01	4.114434e-02	0.9997779456	-0.1415422381	0.6100141285	0.93042774	-2.175339e-01	0.373605932	0.9982883	3.456062e-01	0.2223116318
0.77869908	1.510665e-01	3.816682e-01	0.9997779456	0.4377559149	0.4651170084	0.90612020	-1.338075e-02	0.982087631	0.9986411	-2.219769e-01	0.6131018610
0.77869908	-1.575236e-01	9.493149e-01	0.9997779456	-0.1641493664	0.0394817882	0.76504055	-1.195511e-01	0.100712486	0.9982883	-2.494640e-02	0.7820867831
0.90581107	-2.479832e-01	6.526768e-01	0.9997779456	0.2821299516	0.7240580796	0.94712279	-8.773522e-03	0.983013338	0.9986411	3.239568e-01	0.6805952200
0.97273515	1.002274e-01	4.472320e-01	0.9997779456	-0.3107968780	0.6964251993	0.94482995	1.015423e+00	0.234142585	0.9982883	-1.154289e+00	0.1509778334
0.90581107	-1.813629e-01	8.026449e-01	0.9997779456	0.6438160722	0.0507571295	0.83664873	2.412470e-01	0.248817857	0.9982883	1.924855e-01	0.6239626300
0.99018039	-9.757485e-01	8.767891e-01	0.9997779456	-0.9535975750	0.1031721496	0.83807199	-9.133756e-01	0.258706329	0.9982883	1.162323e+00	0.3537425174
0.83725376	-1.187499e-02	5.549554e-01	0.9997779456	0.0450231346	0.8253254813	0.95988696	1.537998e-01	0.149714081	0.9982883	7.125461e-02	0.6200441156
0.86909435	1.133999e-01	2.695025e-01	0.9997779456	0.3244272403	0.1382275744	0.83807199	2.402537e-01	0.178048988	0.9982883	1.766256e-01	0.3628002660
0.99258481	4.060032e-01	3.376122e-02	0.9997779456	0.5588347795	0.1742028620	0.83807199	3.337317e-01	0.099185736	0.9982883	-2.494863e-02	0.9278286851
0.79972808	-1.347646e-01	8.804441e-01	0.9997779456	-0.1193120985	0.5040605857	0.90612020	-1.194530e-01	0.457095753	0.9982883	-4.589985e-02	0.7598130856
0.84824569	-1.671511e-01	9.632111e-01	0.9997779456	0.0103622766	0.9221348994	0.98283449	-3.355085e-03	0.971431718	0.9986411	1.162539e-01	0.1956840773
0.84600172	-2.612064e-01	9.580274e-01	0.9997779456	-0.4469233999	0.0647982235	0.83807199	1.740655e-01	0.298445142	0.9982883	-9.252141e-02	0.6143022343
0.86909435	4.945494e-02	4.659681e-01	0.9997779456	0.4117252048	0.3873911006	0.87916124	5.004175e-01	0.276534542	0.9982883	-4.371531e-02	0.9353534582
0.79972808	-2.904571e-01	8.759165e-01	0.9997779456	-0.1685376469	0.3916356430	0.87916124	-1.133010e-01	0.640824746	0.9982883	-2.475431e-01	0.4469631347
0.97144018	4.405183e-02	3.439227e-01	0.9997779456	0.1401909736	0.2242526137	0.83807199	-5.446493e-02	0.555532963	0.9982883	-5.861466e-02	0.4479446692
0.99066584	-1.541043e-01	8.883571e-01	0.9997779456	0.1540390921	0.2208811361	0.83807199	-8.778804e-02	0.515714039	0.9982883	-2.677665e-01	0.1116118049
0.92876201	1.539268e-01	1.675510e-01	0.9997779456	0.2659930016	0.3047246750	0.84915316	2.641069e-01	0.065472340	0.9982883	-9.823156e-03	0.9498464148
0.77869908	2.649304e-01	1.747706e-01	0.9997779456	0.0886987344	0.8102447356	0.95972154	-1.399570e-03	0.996061388	0.9988193	-1.301749e-01	0.6901056982
0.80820047	2.365759e-01	3.504947e-01	0.9997779456	-0.2033928084	0.6647177006	0.94041014	7.492447e-01	0.226185159	0.9982883	8.223387e-01	0.1635578190
0.97522657	-2.152731e-01	9.686660e-01	0.9997779456	-0.0240984421	0.8417775836	0.96338860	-2.937708e-02	0.806477743	0.9982883	-1.366394e-01	0.2094956496
0.77869908	-1.170055e-01	7.945280e-01	0.9997779456	0.1856480213	0.3105953588	0.84915316	4.801995e-02	0.732943201	0.9982883	1.307522e-01	0.5185238573
0.77869908	2.098020e-01	7.085270e-02	0.9997779456	0.0903968793	0.6071664600	0.93042774	-9.179598e-02	0.371999227	0.9982883	2.835877e-02	0.7739335113
0.77869908	-5.216248e-04	5.009957e-01	0.9997779456	-0.4060523319	0.0839509966	0.83807199	-4.159480e-01	0.038287848	0.9982883	-2.192883e-01	0.3561750444
0.97522657	-8.191183e-02	8.407397e-01	0.9997779456	0.0136358856	0.8771963376	0.96947881	5.858143e-02	0.486729809	0.9982883	-7.470761e-04	0.9952721427
0.84824569	-1.352236e-01	9.029476e-01	0.9997779456	-0.0533506942	0.6199299659	0.93342431	5.605868e-02	0.448371334	0.9982883	1.477238e-02	0.8802791326
0.81163165	1.069931e-01	3.573427e-01	0.9997779456	0.2243391842	0.5783593573	0.92506192	-1.380568e-01	0.607239091	0.9982883	-2.232142e-01	0.3471037714
0.81163165	2.063760e-02	3.714021e-01	0.9997779456	0.0457355871	0.5226094750	0.90612020	7.018739e-02	0.215549259	0.9982883	1.013492e-01	0.1626448575
0.97273515	-2.262279e-01	9.891063e-01	0.9997779456	-0.1190999420	0.2682494804	0.84915316	5.217344e-02	0.554691375	0.9982883	-2.618290e-01	0.0914789815
0.97273515	-1.558710e-01	6.375122e-01	0.9997779456	0.3286215734	0.3279412039	0.85754449	-5.032834e-01	0.174453824	0.9982883	-1.145445e-01	0.7762164407
0.84824569	-4.873108e-02	7.230481e-01	0.9997779456	0.0184456506	0.8529613950	0.96663506	-5.763122e-02	0.458210218	0.9982883	-2.641049e-01	0.0236058457
0.99420295	-8.637847e-02	7.456160e-01	0.9997779456	0.0628656660	0.7136767769	0.94482995	-2.212361e-02	0.849272034	0.9982883	-2.043223e-01	0.3168978108
0.88581308	-1.942882e-01	9.287195e-01	0.9997779456	-0.1019120877	0.7196165468	0.94712279	4.856825e-03	0.969187876	0.9986411	-3.040001e-02	0.8984981478
0.99018039	2.093161e-01	1.976157e-01	0.9997779456	0.4842358982	0.2131629191	0.83807199	4.166628e-01	0.107724879	0.9982883	-4.693137e-01	0.0854471592
0.77869908	-2.593145e-01	8.572507e-01	0.9997779456	-0.7900003641	0.0006952627	0.10272506	-1.369310e-01	0.515413801	0.9982883	6.579917e-02	0.8195067622
0.80820047	1.251586e-01	2.112259e-01	0.9997779456	-0.2884805621	0.1538196026	0.83807199	-1.127811e-01	0.435136307	0.9982883	5.075594e-01	0.0937060445
0.82862325	-1.250007e-01	8.944673e-01	0.9997779456	0.0693105117	0.6375921866	0.93852299	-2.603811e-03	0.977424290	0.9986411	1.103482e-01	0.2468564031
0.98996967	-5.150401e-02	6.921630e-01	0.9997779456	0.1255802906	0.4681998796	0.90612020	-1.125828e-02	0.903348452	0.9982883	-1.175599e-01	0.1553019012
0.77869908	9.726503e-03	4.904029e-01	0.9997779456	0.2190623017	0.5922039764	0.92532140	5.129794e-02	0.890923540	0.9982883	-1.012641e-01	0.8129688505
0.98299735	-1.408854e-01	7.810448e-01	0.9997779456	0.0563730152	0.8492816557	0.96338860	-2.261874e-01	0.190930240	0.9982883	-1.461611e-01	0.5179640341
0.96804147	-1.756729e-01	6.391970e-01	0.9997779456	-0.4983722559	0.1964542368	0.83807199	9.752125e-02	0.748916889	0.9982883	-5.120351e-01	0.0672646409
0.80820047	7.141676e-02	3.868351e-01	0.9997779456	0.7455999920	0.3313040262	0.85754449	9.764523e-02	0.708629159	0.9982883	-4.306057e-01	0.0975122125
0.87099999	3.396281e-03	4.853007e-01	0.9997779456	0.0260041983	0.7829684390	0.95657490	1.140315e-01	0.188575010	0.9982883	-3.961843e-02	0.7264641083
0.91967042	-2.368798e-01	9.844970e-01	0.9997779456	-0.0754844111	0.4694787147	0.90612020	5.465631e-02	0.519954575	0.9982883	-1.308055e-01	0.1831775697
0.87292023	-4.181614e-02	5.758104e-01	0.9997779456	-0.1979608057	0.6397362351	0.93854411	-4.324161e-01	0.121611903	0.9982883	-3.085451e-01	0.2036284942
0.77869908	-8.057526e-02	7.982870e-01	0.9997779456	0.0053331949	0.9624175121	0.99380888	4.916324e-02	0.493127354	0.9982883	-4.945656e-02	0.6624382552
0.88001839	5.642563e-02	2.893191e-01	0.9997779456	-0.0398347288	0.8296608763	0.96048889	-2.972346e-02	0.751302823	0.9982883	-5.091583e-02	0.7156121374
0.77869908	4.936141e-02	2.922538e-01	0.9997779456	-0.0005799904	0.9968978513	0.99853613	-7.519961e-02	0.479762959	0.9982883	-4.038418e-02	0.7500558186
0.77869908	-2.178793e-01	9.479563e-01	0.9997779456	-0.2076049729	0.1283467324	0.83807199	-3.101732e-02	0.813055272	0.9982883	-2.354269e-01	0.0409204750
0.79578964	6.371068e-01	1.576528e-01	0.9997779456	-0.1893941223	0.7673042664	0.95505354	6.291472e-01	0.296357894	0.9982883	1.237184e+00	0.3596745404
0.77869908	3.249157e-01	3.480652e-01	0.9997779456	0.8342450380	0.4346144162	0.90612020	8.517568e-01	0.179073363	0.9982883	1.047809e-01	0.8898257921
0.77869908	-8.679650e-02	6.198099e-01	0.9997779456	-0.4203897760	0.1588390768	0.83807199	-4.540710e-02	0.850117210	0.9982883	-6.710067e-02	0.7908847514
0.87292023	-7.272048e-02	8.944303e-01	0.9997779456	-0.0658135038	0.4191296100	0.89856301	2.400581e-03	0.968086352	0.9986411	3.935965e-02	0.6875509431
0.96804147	1.044213e-01	1.001803e-01	0.9997779456	0.0481198331	0.6785268979	0.94316618	-7.777364e-02	0.346846098	0.9982883	6.155833e-02	0.5162307917
0.97273515	-1.777510e-01	8.877663e-01	0.9997779456	-0.2053526945	0.2736070689	0.84915316	-1.264886e-01	0.307115497	0.9982883	-1.434911e-01	0.3021843764
0.88636459	-3.567327e-02	6.437618e-01	0.9997779456	-0.0498344586	0.5610526404	0.91675659	1.067652e-01	0.245299557	0.9982883	2.131106e-01	0.0778084779
0.80820047	1.656053e-01	3.290767e-01	0.9997779456	0.1511164408	0.6601419201	0.93982803	1.115627e-01	0.748184538	0.9982883	5.207124e-01	0.2224977124
0.77869908	1.241650e-01	1.697742e-01	0.9997779456	-0.3166243890	0.0732473543	0.83807199	-1.055767e-01	0.437566999	0.9982883	2.146598e-01	0.0962289628
0.77869908	-5.879559e-02	6.287998e-01	0.9997779456	0.1977433586	0.3891800522	0.87916124	2.114859e-03	0.989738906	0.9986411	1.244099e-01	0.5413978240
0.77869908	-2.320485e-01	8.631076e-01	0.9997779456	-0.4330776380	0.0404426609	0.77101976	-1.065304e-01	0.531044419	0.9982883	-2.538675e-01	0.2208474662
0.77869908	6.081043e-02	3.129686e-01	0.9997779456	-0.0477422473	0.6553450456	0.93854411	-5.416573e-02	0.571621574	0.9982883	1.919745e-02	0.8963792577
0.77869908	-1.491423e-02	5.465218e-01	0.9997779456	-0.3145505982	0.0063869307	0.40915301	-1.492159e-01	0.148394316	0.9982883	1.614790e-01	0.1152145849
0.77869908	1.173337e-01	8.727180e-02	0.9997779456	0.0996550008	0.3732031863	0.87664366	3.635458e-02	0.660231143	0.9982883	8.140462e-02	0.4345082242
0.96804147	6.678018e-02	3.095539e-01	0.9997779456	-0.0420759523	0.8041947480	0.95972154	6.482765e-02	0.695371273	0.9982883	-2.306712e-01	0.4521347860
0.95284371	5.788468e-02	3.673781e-01	0.9997779456	-0.0716774570	0.7997702935	0.95972154	-3.310397e-02	0.840701326	0.9982883	2.389644e-01	0.1939858022
0.80820047	1.430209e-02	4.316550e-01	0.9997779456	-0.0531298246	0.7006065871	0.94482995	9.868378e-02	0.168124987	0.9982883	-1.653233e-01	0.0695566270
0.97273515	-2.353376e-01	7.554390e-01	0.9997779456	0.3703200965	0.1202122239	0.83807199	1.311702e-01	0.619788544	0.9982883	-2.897124e-01	0.3845632576
0.96804147	-5.064525e-02	6.240823e-01	0.9997779456	0.2620391656	0.2016502820	0.83807199	2.917676e-02	0.836276416	0.9982883	2.488473e-02	0.8812335495
0.99066584	-4.557915e-02	6.004846e-01	0.9997779456	0.3725891601	0.0903145122	0.83807199	7.904065e-02	0.674320235	0.9982883	-1.782476e-02	0.9338798149
0.96804147	-2.220751e-02	5.794655e-01	0.9997779456	0.2841668976	0.0255898313	0.64586404	-4.241820e-02	0.684213760	0.9982883	-7.177870e-02	0.5794670195
0.78646912	3.459329e-02	3.980647e-01	0.9997779456	-0.2488139754	0.1504244537	0.83807199	1.534254e-02	0.879702292	0.9982883	1.609251e-01	0.1108717338
0.77869908	9.513296e-01	1.271354e-01	0.9997779456	-0.5684780055	0.4165186668	0.89840340	-1.238488e+00	0.365926000	0.9982883	-8.318198e-01	0.2696273938
0.80985926	-4.332644e-02	6.731477e-01	0.9997779456	-0.1530393367	0.1690915436	0.83807199	5.375931e-02	0.468652211	0.9982883	9.977358e-02	0.4614375935
0.81552863	1.203103e-01	2.249279e-01	0.9997779456	-0.1985162193	0.3179461852	0.84915316	-1.668830e-01	0.223177765	0.9982883	-2.544413e-01	0.1283727459
0.77869908	2.517393e-01	1.410696e-01	0.9997779456	-0.3891413101	0.0545024979	0.83664873	-4.992014e-01	0.086134646	0.9982883	-2.703562e-01	0.2358648876
0.97273515	-2.026357e-01	8.727678e-01	0.9997779456	-0.6697072002	0.0055797145	0.40915301	-3.243756e-01	0.073134545	0.9982883	-1.971546e-01	0.3670274289
0.95134638	-1.111951e-01	7.918070e-01	0.9997779456	0.1039117808	0.7253936436	0.94712279	-5.234667e-02	0.660956407	0.9982883	-1.845089e-01	0.1241343968
0.97522657	2.730054e-01	1.594447e-01	0.9997779456	0.6593483568	0.4505386951	0.90612020	1.538029e-01	0.590879080	0.9982883	-3.190177e-01	0.2889929885
0.77869908	1.506357e-02	4.575728e-01	0.9997779456	-0.2528034017	0.1255525937	0.83807199	-1.394060e-01	0.320076456	0.9982883	-9.616351e-04	0.9957614483
0.94008598	1.324894e-02	4.702290e-01	0.9997779456	0.0540338273	0.7785028254	0.95657490	-1.520929e-01	0.281451757	0.9982883	-3.920469e-01	0.0039596045
0.77869908	-9.030129e-02	5.381359e-01	0.9997779456	-2.3374244528	0.0137179778	0.49135302	-1.620559e+00	0.127642333	0.9982883	-2.272942e+00	0.0149552201
0.79213004	-9.670855e-02	8.754115e-01	0.9997779456	-0.1991889557	0.1047356952	0.83807199	-2.648885e-02	0.742504310	0.9982883	-1.248040e-01	0.2752647013
0.77869908	-7.892337e-02	6.083427e-01	0.9997779456	-0.1109420442	0.6884310938	0.94482995	5.797636e-02	0.827020064	0.9982883	-2.058948e-01	0.5339883592
0.97050390	2.876960e-01	9.358436e-02	0.9997779456	0.2074775630	0.1809231666	0.83807199	-3.451767e-01	0.025513665	0.9982883	2.064821e-01	0.3344815164
0.83725376	-1.578076e-01	8.387027e-01	0.9997779456	-0.1420918375	0.4441991478	0.90612020	-1.949430e-02	0.872282771	0.9982883	-9.849720e-02	0.6578042713
0.77869908	1.100024e-01	1.396862e-01	0.9997779456	-0.0108463955	0.9454180204	0.98868207	3.209154e-02	0.747338972	0.9982883	2.478689e-01	0.1217510030
0.88581308	-2.792633e-02	5.809647e-01	0.9997779456	-0.1561390187	0.1911314466	0.83807199	4.130588e-02	0.800053567	0.9982883	2.519562e-01	0.1921733717
0.97050390	4.022812e-01	2.707973e-02	0.9997779456	-0.0419008023	0.8618751792	0.96707132	-5.557757e-01	0.095455582	0.9982883	-2.738760e-01	0.3093187412
0.77869908	5.800640e-02	3.344816e-01	0.9997779456	-0.0210724564	0.9238616210	0.98290228	1.901165e-02	0.903226550	0.9982883	8.277566e-02	0.5859881697
0.88001839	1.527981e-01	9.592490e-02	0.9997779456	-0.1939549930	0.0781850057	0.83807199	4.743201e-02	0.682535779	0.9982883	-1.234797e-01	0.3571209238
0.99018039	-7.755176e-03	5.141678e-01	0.9997779456	0.2125705770	0.2015879057	0.83807199	-9.949520e-02	0.676071832	0.9982883	-3.124642e-01	0.3178647178
0.77869908	-6.896719e-02	5.589160e-01	0.9997779456	0.5296431950	0.3243263584	0.85614181	1.948377e-01	0.571561890	0.9982883	1.638691e-01	0.7417446578
0.78569981	-1.000252e-01	8.231238e-01	0.9997779456	-0.2272690270	0.0823725957	0.83807199	-1.014637e-01	0.283569278	0.9982883	1.444635e-01	0.1175402976
0.89273364	7.669792e-02	2.734474e-01	0.9997779456	-0.0148512461	0.8992688363	0.97427659	-2.418269e-02	0.813607182	0.9982883	-7.509682e-02	0.6648895336
0.94675587	-6.896049e-02	7.161659e-01	0.9997779456	-0.1599668793	0.0951400494	0.83807199	-1.927943e-01	0.018139093	0.9982883	-1.593357e-01	0.1645263738
0.97050390	1.581963e-01	2.335134e-01	0.9997779456	-0.0407360841	0.8613533829	0.96707132	-5.744402e-02	0.750677525	0.9982883	-1.760237e-01	0.3970619386
0.79578964	-1.288070e-01	8.625402e-01	0.9997779456	-0.2424983538	0.0105084080	0.46180258	-2.207317e-02	0.805645710	0.9982883	-1.516380e-01	0.1499396887
0.86955753	-3.506903e-01	9.080009e-01	0.9997779456	0.1351648523	0.6191899568	0.93342431	5.934354e-02	0.816765351	0.9982883	-1.606189e-01	0.6346214162
0.77869908	3.366249e-01	2.928693e-01	0.9997779456	0.4303169662	0.5153424139	0.90612020	2.581561e-02	0.970908179	0.9986411	6.920412e-02	0.9273733623
0.77869908	2.999225e-01	3.206609e-01	0.9997779456	0.2733868584	0.6125371925	0.93061563	-1.354615e-01	0.857336422	0.9982883	-1.763817e-01	0.7905148491
0.77869908	-5.152312e-02	5.502490e-01	0.9997779456	0.8220303093	0.1934586374	0.83807199	-7.208321e-02	0.858747202	0.9982883	1.784209e-01	0.7567298128
0.97273515	6.257834e-01	1.207514e-01	0.9997779456	0.9577756133	0.2065585444	0.83807199	1.981593e-02	0.970094303	0.9986411	-3.115525e-01	0.6439065583
0.97522657	2.581424e-01	2.419959e-01	0.9997779456	-0.7585446135	0.0207170348	0.60526393	-8.783667e-01	0.005892289	0.9982883	-1.500148e-01	0.6727898065
0.98996967	-8.431254e-01	8.535594e-01	0.9997779456	-0.1054953450	0.8116932984	0.95972154	3.552768e-01	0.446717623	0.9982883	-6.385100e-01	0.1877055221
0.97273515	-6.500511e-02	5.637664e-01	0.9997779456	-0.5658339933	0.2550300824	0.84915316	-3.662617e-01	0.367789826	0.9982883	2.101033e-01	0.7026403238
0.77869908	4.273553e-01	1.332225e-01	0.9997779456	0.1406441949	0.7571394118	0.95505354	1.206226e-01	0.739712268	0.9982883	3.813536e-01	0.2967046839
0.77869908	-5.547505e-02	5.467666e-01	0.9997779456	0.4625400585	0.4952763136	0.90612020	-4.210336e-02	0.908940574	0.9982883	3.646936e-02	0.9128054558

0.80820047	-1.241165e-01	8.184487e-01	0.9997779456	-0.2221433590	0.2960161919	0.84915316	1.151007e-01	0.281027340	0.9982883	3.556493e-02	0.7648420901
0.92876201	9.217259e-02	2.833959e-01	0.9997779456	-0.0278508337	0.8037948652	0.95972154	-1.592834e-01	0.273241903	0.9982883	-5.696705e-02	0.7710534729
0.97522657	6.335286e-02	3.328976e-01	0.9997779456	0.0864376673	0.7243977803	0.94712279	1.218710e-01	0.287537292	0.9982883	3.152236e-02	0.7682207894
0.95487380	7.170637e-02	3.468708e-01	0.9997779456	0.3503184029	0.1625126845	0.83807199	2.139098e-01	0.242059231	0.9982883	-2.870243e-02	0.8719297458
0.95558280	-2.643281e-01	9.151647e-01	0.9997779456	-0.2017203835	0.2361379676	0.84413548	-1.663322e-01	0.310547706	0.9982883	-5.035532e-01	0.0023578299
0.95353311	2.217487e-01	1.746871e-02	0.9997779456	0.0070961395	0.9494871139	0.99055055	-1.300925e-01	0.169660643	0.9982883	5.452342e-03	0.9625478856
0.87292023	1.047122e-01	1.989295e-01	0.9997779456	0.2587291392	0.0020706521	0.24475108	1.442060e-01	0.266993873	0.9982883	-3.232696e-02	0.8061227039
0.97522657	-2.743794e-01	6.761298e-01	0.9997779456	0.5964790895	0.4397120559	0.90612020	4.724012e-01	0.421725717	0.9982883	-1.803021e+00	0.0336780742
0.89512661	-9.037820e-02	6.791118e-01	0.9997779456	0.4216689498	0.2121959966	0.83807199	1.969316e-01	0.130842509	0.9982883	-1.141140e-01	0.4934178442
0.79578964	8.129109e-02	4.353404e-01	0.9997779456	0.6891291492	0.2646466024	0.84915316	2.279115e-01	0.625262315	0.9982883	-1.021916e-02	0.9866398021
0.95202293	-7.174956e-02	7.965116e-01	0.9997779456	-0.0173303381	0.8656661848	0.96707132	9.051295e-03	0.897912676	0.9982883	-1.597356e-01	0.0189950451
0.81552863	-2.385369e-01	8.363427e-01	0.9997779456	-0.2178672513	0.3783249154	0.87916124	-3.797424e-02	0.880176218	0.9982883	-6.942784e-01	0.0162938816
0.98323587	-1.875070e-01	7.095142e-01	0.9997779456	-0.0450655407	0.8589414812	0.96707132	-9.080622e-02	0.749659522	0.9982883	-4.461040e-02	0.8519048076
0.77869908	-7.407521e-02	7.198048e-01	0.9997779456	-0.2626588477	0.0129678050	0.47899830	-2.906718e-02	0.810953631	0.9982883	-1.202770e-02	0.8956444005
0.83237211	-3.840714e-01	9.995623e-01	0.9997779456	-0.1496731879	0.2669319830	0.84915316	-1.186081e-01	0.300172827	0.9982883	-2.688817e-01	0.0738207666
0.99066584	-4.040856e-01	8.327921e-01	0.9997779456	0.0111255544	0.9700143499	0.99380888	1.527184e-01	0.537953716	0.9982883	-3.151691e-01	0.1284785969
0.77869908	-5.408884e-02	7.668636e-01	0.9997779456	-0.0201043587	0.8656964929	0.96707132	6.371215e-02	0.489662722	0.9982883	-2.712676e-02	0.7920518958
0.86909435	6.734135e-01	1.706691e-04	0.0672436197	0.4087814643	0.1470151767	0.83807199	-1.581913e-02	0.929698519	0.9986411	3.138459e-01	0.1846152003
0.99066584	5.169252e-02	3.341360e-01	0.9997779456	0.1713120921	0.2473213155	0.84413548	2.308987e-02	0.817933456	0.9982883	-2.082009e-01	0.1020478786
0.97273515	9.000028e-04	4.974569e-01	0.9997779456	0.1885865337	0.4429363080	0.90612020	1.599241e-03	0.989973844	0.9986411	-2.101062e-01	0.1065050284
0.77869908	7.626627e-02	4.326077e-01	0.9997779456	0.6478902211	0.2942004451	0.84915316	-2.945004e-03	0.994796061	0.9988193	2.901785e-01	0.6574521631
0.79578964	2.843697e-02	4.629517e-01	0.9997779456	-0.3982941629	0.2650227528	0.84915316	-1.630146e-01	0.562503060	0.9982883	8.282059e-03	0.9827055546
0.79213004	-8.441899e-02	8.259928e-01	0.9997779456	0.0367370411	0.6907116768	0.94482995	-3.558192e-02	0.623707875	0.9982883	1.278391e-01	0.2829251819
0.94189574	1.688295e-01	5.304014e-02	0.9997779456	-0.0107457915	0.9329081420	0.98590149	7.581621e-03	0.922576594	0.9982883	-8.720096e-02	0.5044260445
0.82138617	4.325041e-02	3.752449e-01	0.9997779456	-0.1298401566	0.2712173698	0.84915316	-8.136588e-02	0.577825307	0.9982883	-7.757626e-02	0.6113105017
0.90581107	-3.808283e-02	6.096672e-01	0.9997779456	-0.2519862359	0.4190887058	0.89856301	-3.059775e-01	0.027551418	0.9982883	2.348271e-01	0.1963459153
0.86909435	5.087168e-02	4.179811e-01	0.9997779456	-0.1623824205	0.6574572928	0.93854411	-7.177891e-02	0.775929271	0.9982883	-4.432607e-01	0.1460981084
0.77869908	3.262849e-01	1.235795e-01	0.9997779456	0.0504071832	0.8148549135	0.95972154	-2.606655e-01	0.167869926	0.9982883	2.740498e-01	0.2375492496
0.97273515	6.073565e-02	3.377841e-01	0.9997779456	-0.2866335364	0.1241587056	0.83807199	4.218703e-02	0.749710568	0.9982883	-1.066113e-01	0.6079927174
0.97273515	9.782790e-02	2.266213e-01	0.9997779456	0.0886203968	0.4872565529	0.90612020	1.828728e-01	0.113921031	0.9982883	7.390979e-02	0.5436926097
0.97050390	-5.861658e-02	6.150014e-01	0.9997779456	-0.0876946264	0.7687952871	0.95505354	1.533273e-01	0.331593882	0.9982883	9.879482e-03	0.9673837306
0.77869908	3.148192e+00	6.186185e-07	0.0007312071	0.1537928310	0.7699063333	0.95505354	3.321325e-01	0.440924385	0.9982883	6.257843e-01	0.1874504831
0.90581107	-3.140021e-01	8.538312e-01	0.9997779456	-0.5077756305	0.2413447454	0.84413548	-3.182113e-01	0.175642793	0.9982883	-1.915024e-02	0.9601994793
0.88636459	1.444759e-02	4.777923e-01	0.9997779456	-0.0480574963	0.8734960444	0.96947881	-4.125647e-02	0.858331093	0.9982883	1.594241e-01	0.6728562032
0.95472383	-1.572701e-01	8.959066e-01	0.9997779456	-0.1567072417	0.2838516473	0.84915316	9.695628e-02	0.301373713	0.9982883	1.324416e-01	0.3624086169
0.77869908	1.593094e-02	4.305038e-01	0.9997779456	-0.0209453232	0.8161007374	0.95972154	3.927091e-02	0.661685649	0.9982883	-3.332786e-02	0.8227513634
0.90802583	2.128323e-01	4.547286e-02	0.9997779456	0.1830280992	0.3901357068	0.87916124	3.701205e-02	0.717560682	0.9982883	3.047939e-01	0.0020756404
0.81552863	4.983025e-02	2.982540e-01	0.9997779456	0.0787204307	0.4077208400	0.89501894	1.107538e-01	0.158792003	0.9982883	-2.371631e-03	0.9818596663
0.84315681	6.064246e-02	1.819700e-01	0.9997779456	0.1570748849	0.0060528836	0.40915301	3.522932e-02	0.546816660	0.9982883	-9.337731e-02	0.1354401574
0.83725376	1.493878e-01	2.880106e-01	0.9997779456	0.6600703713	0.1285518537	0.83807199	3.169812e-01	0.173972734	0.9982883	5.528312e-01	0.1142842054
0.89369078	-6.195493e-02	7.996615e-01	0.9997779456	-0.1422410576	0.0611271150	0.83807199	1.194558e-02	0.867015596	0.9982883	-1.263739e-01	0.2346880059
0.82862325	-1.474071e-01	9.398455e-01	0.9997779456	0.0241142765	0.7991448157	0.95972154	1.222047e-01	0.147251560	0.9982883	3.080312e-02	0.7553418446
0.89369078	-2.614813e-01	9.932837e-01	0.9997779456	-0.0531214784	0.5886303822	0.92532140	1.340011e-01	0.304934187	0.9982883	1.864385e-01	0.0786884709
0.80820047	-2.559370e-01	9.860757e-01	0.9997779456	-0.1244431035	0.2902750065	0.84915316	-1.280162e-01	0.282048528	0.9982883	-2.362126e-01	0.2137607165
0.77869908	2.446674e-01	1.155479e-01	0.9997779456	0.4051287241	0.1458324123	0.83807199	2.496313e-01	0.209389931	0.9982883	2.981579e-01	0.1910483644
0.99420295	-1.847258e-01	8.395686e-01	0.9997779456	-0.1375001867	0.4807471484	0.90612020	-1.037869e-01	0.525893153	0.9982883	-9.991718e-02	0.6687598948
0.81163165	-1.117751e-01	8.317517e-01	0.9997779456	-0.0726724435	0.5018882973	0.90612020	1.602051e-02	0.867633733	0.9982883	-2.026325e-02	0.8489371793
0.86955753	4.412138e-02	2.428422e-01	0.9997779456	0.1030530650	0.2016291988	0.83807199	3.790994e-02	0.530399980	0.9982883	-5.134733e-02	0.6380843091
0.98299735	8.393150e-02	2.475155e-01	0.9997779456	0.4259505867	0.0014825729	0.19471124	9.607577e-02	0.500720326	0.9982883	-6.221214e-02	0.6619732060
0.95472383	-5.960743e-02	7.643514e-01	0.9997779456	0.0631593385	0.4601230160	0.90612020	5.354124e-02	0.438328195	0.9982883	-8.997753e-03	0.8879865186
0.92041992	2.744103e-02	3.512379e-01	0.9997779456	-0.0308346243	0.7039112562	0.94482995	-2.763939e-02	0.719251597	0.9982883	-5.268490e-02	0.6241606431
0.97273515	-2.326015e-01	8.123433e-01	0.9997779456	-0.3323210985	0.0943818663	0.83807199	-3.364894e-01	0.121465373	0.9982883	-5.421189e-01	0.0123857877
0.87292023	-1.003760e-01	7.754704e-01	0.9997779456	-0.1809845008	0.2001378584	0.83807199	-1.474164e-01	0.311099972	0.9982883	-1.716153e-01	0.2695970425
0.89656266	-1.982093e-01	9.150910e-01	0.9997779456	-0.1668400678	0.2141202783	0.83807199	-1.552793e-01	0.322930876	0.9982883	-1.240384e-01	0.4950347417
0.81552863	-5.274942e-01	9.817887e-01	0.9997779456	-0.1708873031	0.5945331992	0.92532140	1.695949e-01	0.435229335	0.9982883	-3.241137e-01	0.3525736223
0.77869908	1.481740e-01	7.880491e-02	0.9997779456	0.2303746561	0.1152526469	0.83807199	-1.078765e-03	0.990192395	0.9986411	2.641997e-01	0.0302419252
0.97185522	5.798294e-01	3.401758e-02	0.9997779456	0.2350115317	0.6439911386	0.93854411	2.441713e-01	0.416515569	0.9982883	-4.291533e-01	0.3798209003
0.79578964	2.290435e-01	6.819882e-02	0.9997779456	-0.0324808364	0.8946121931	0.97246977	8.283355e-03	0.954610023	0.9986411	2.782447e-01	0.2492424147
0.77869908	2.241258e-01	9.167129e-02	0.9997779456	-0.0818704870	0.7004010685	0.94482995	-7.265511e-02	0.561419958	0.9982883	1.288323e-01	0.3991273453
0.98042753	1.542913e-02	4.456196e-01	0.9997779456	0.1821203898	0.3021480910	0.84915316	1.994512e-01	0.068352555	0.9982883	2.312791e-01	0.0805454859
0.82862325	-4.514748e-02	7.295478e-01	0.9997779456	-0.0769293666	0.5228285708	0.90612020	9.587300e-03	0.903617299	0.9982883	2.996221e-02	0.7677875546
0.77869908	-8.252228e-02	7.761686e-01	0.9997779456	-0.0228189531	0.8257216364	0.95988696	-8.826702e-02	0.326578945	0.9982883	1.181169e-01	0.3508527295
0.90802583	2.830022e-03	4.921972e-01	0.9997779456	0.1102153813	0.6111027899	0.93042774	-1.437610e-01	0.317314251	0.9982883	-9.649112e-02	0.5252130148
0.95181672	-7.930521e-02	8.456266e-01	0.9997779456	0.0288398392	0.8159982383	0.95972154	1.051805e-01	0.115257434	0.9982883	1.077715e-01	0.3189323117
0.87292023	-4.761021e-02	6.760674e-01	0.9997779456	0.0403507905	0.6959501922	0.94482995	7.193582e-02	0.403959598	0.9982883	1.886896e-01	0.1208607111
0.93695105	-2.634725e-02	5.536440e-01	0.9997779456	-0.1340412254	0.5656891051	0.91973112	1.997990e-03	0.989669041	0.9986411	-1.414643e-01	0.4118021273
0.77869908	-1.018443e-01	6.267130e-01	0.9997779456	-0.8315226674	0.0349854615	0.72548799	5.403139e-02	0.849026724	0.9982883	4.955944e-01	0.0415534449
0.80820047	9.021357e-02	3.172461e-01	0.9997779456	0.5923782105	0.3000475061	0.84915316	4.335077e-01	0.040903409	0.9982883	1.788557e-01	0.5905489570
0.95487380	1.338630e-02	4.753710e-01	0.9997779456	-0.0604655015	0.8376950871	0.96321135	-1.358905e-01	0.500913770	0.9982883	-1.151968e-01	0.6583997179
0.77869908	-8.579334e-02	6.937168e-01	0.9997779456	-0.0473255754	0.7721738969	0.95505354	-2.383309e-01	0.072426618	0.9982883	-1.649182e-01	0.3151612664
0.97823692	7.096516e-01	1.409487e-01	0.9997779456	-1.0882049176	0.0804829320	0.83807199	-7.113257e-01	0.463195310	0.9982883	-2.111911e-01	0.7409248241
0.77869908	1.714821e-01	3.657676e-01	0.9997779456	0.1393403143	0.7830604244	0.95657490	-1.427485e-01	0.804082665	0.9982883	-1.698835e-02	0.9791263242
0.99066584	-3.796737e-01	9.273574e-01	0.9997779456	-0.1227610911	0.7057219771	0.94482995	-1.471400e-01	0.544874524	0.9982883	-4.488315e-01	0.1495315888
0.97273515	-9.357064e-02	7.021253e-01	0.9997779456	0.2636182256	0.1809897059	0.83807199	1.156825e-01	0.478732171	0.9982883	-4.219433e-01	0.1408860230

Activesmoking	Allergy	Allergy	Allergy	PrCI	PrCI	PrCI
Adj. p-val. (2-sided test, Benjamini-H. cor.)	AVG.Log.Ratio	p-val. (2-sided test)	Adj. p-val. (2-sided test, Benjamini-H. cor.)	AVG.Log.Ratio	p-val. (2-sided test)	Adj. p-val. (2-sided test, Benjamini-H. cor.)
0.9923046	-0.0384131929	9.005915e-01	0.9969722	0.0928284216	8.020690e-01	9.207928e-01
0.8497626	0.1394959691	5.699765e-01	0.9641086	-0.4063845427	1.273771e-01	4.047304e-01
0.9949051	-0.8090177606	5.120539e-02	0.7921508	-0.0428904548	9.274500e-01	9.835175e-01
0.9949051	-0.2348907008	6.354114e-01	0.9689755	-0.3102466520	4.342856e-01	7.199517e-01
0.8394240	0.2815026205	6.091330e-01	0.9641086	-1.1300490874	3.096886e-02	1.848747e-01
0.9656591	0.2433755404	6.733245e-01	0.9717521	-0.8117161646	3.548698e-02	2.026358e-01
0.9949051	-0.7716564809	1.902003e-01	0.8526631	-0.7164419946	2.221028e-01	5.328026e-01
0.8532963	-0.1813086873	7.480137e-01	0.9823061	-0.7485862790	5.718821e-02	2.714717e-01
0.9949051	-0.5988013736	8.234041e-03	0.3705021	-0.1201309834	6.594688e-01	8.563248e-01
0.4126320	-0.4488913667	7.697931e-02	0.7921508	-0.0927269384	7.423648e-01	8.836608e-01
0.9949051	0.1146802248	5.784051e-01	0.9641086	-0.1641645659	5.332831e-01	7.882615e-01
0.9949051	-0.0714178330	7.211404e-01	0.9733364	0.2135597897	4.083493e-01	6.954883e-01
0.9949051	-0.4623196398	1.034397e-02	0.3705021	-0.1424364744	3.823842e-01	6.715871e-01
0.2034892	0.0114862517	9.619074e-01	0.9969722	-0.0388144645	8.484851e-01	9.481630e-01
0.9949051	-0.9103469230	7.262280e-02	0.7921508	-0.5555174080	3.019218e-01	6.206462e-01
0.9949051	0.6917953994	4.375791e-01	0.9482489	-0.6808525475	2.367177e-01	5.464911e-01
0.9210048	0.2415005092	5.509507e-01	0.9641086	0.0308250688	9.444174e-01	9.835175e-01
0.9210048	-0.5093535574	7.471011e-02	0.7921508	-0.2434086924	2.791358e-01	5.902299e-01
0.7475613	0.5243558356	5.250620e-01	0.9641086	-0.6615656814	6.245319e-02	2.813791e-01
0.8497626	-0.7022248870	1.655841e-02	0.4760109	-0.0644959210	8.894322e-01	9.646093e-01
0.7251512	-0.0655407058	8.731800e-01	0.9969722	0.1314013978	6.787465e-01	8.656656e-01
0.8730639	-0.0355759383	9.087042e-01	0.9969722	-0.0803221310	7.162609e-01	8.762099e-01
0.9949051	-0.4258250814	2.975133e-01	0.9286466	-0.6362527427	6.686264e-02	2.894932e-01
0.9949051	-0.1635736477	4.692179e-01	0.9641086	0.2826894155	2.336882e-01	5.448022e-01
0.8194410	-0.5824920325	4.396554e-01	0.9482489	-0.8509041136	2.784020e-01	5.897332e-01
0.9949051	-0.3529202412	1.440213e-01	0.8166268	-0.1901990237	4.886156e-01	7.621198e-01
0.9949051	-1.1999017887	3.455469e-04	0.0782857	-0.2732188544	5.525670e-01	7.993060e-01
0.8194410	-0.3468905991	3.183402e-01	0.9300968	-0.3209458982	4.728981e-01	7.506714e-01
0.9949051	-0.4420311327	1.577704e-01	0.8386867	0.0222177089	9.643284e-01	9.911619e-01
0.9949051	-0.0982835064	7.237403e-01	0.9733364	-0.1054070574	7.039557e-01	8.762099e-01
0.9949051	-0.2048677567	3.992543e-01	0.9430561	-0.1898711418	3.441145e-01	6.539822e-01
0.9949051	-0.2751653741	3.054104e-01	0.9286466	0.5781147627	3.902754e-01	6.774824e-01
0.8394240	0.1260704721	7.039967e-01	0.9733364	-0.4508814628	8.582686e-02	3.339128e-01
0.9949051	-1.0141029166	1.366314e-01	0.8166268	-0.6083665545	3.174639e-01	6.316783e-01
0.8022770	-0.3996320828	1.498916e-01	0.8317927	-0.2697565188	3.289520e-01	6.412262e-01
0.9148164	0.3585356661	2.809931e-02	0.6133169	-0.1701048738	4.987558e-01	7.643323e-01
0.9210048	-0.0123130680	9.611977e-01	0.9969722	-0.5935363096	6.078064e-02	2.794946e-01
0.8194410	-0.3718597560	9.904905e-02	0.8015257	-0.3243317723	1.137968e-01	3.920865e-01
0.9692638	-0.5455600928	2.151874e-02	0.5298990	-0.5282315593	7.458743e-02	3.159940e-01
0.9949051	0.0170779629	9.693684e-01	0.9969722	-0.3079180617	4.992085e-01	7.643323e-01
0.2019302	0.0008755298	9.974102e-01	0.9997294	-0.2281887775	4.748882e-01	7.506714e-01
0.8612982	-0.2239215483	3.979878e-01	0.9430561	0.0918172310	7.791874e-01	9.053594e-01
0.9949051	-0.2954987763	2.063155e-01	0.8617136	-0.1475828491	4.975011e-01	7.643323e-01
0.9949051	-0.3335481998	1.635457e-01	0.8441531	-0.3123238929	2.298426e-01	5.401073e-01
0.9275791	-0.0295943500	9.064985e-01	0.9969722	-0.1025812035	6.638404e-01	8.572639e-01
0.9949051	-1.3597453829	3.139268e-04	0.0782857	0.1739369619	6.550307e-01	8.556317e-01
0.9949051	0.0042199062	9.732796e-01	0.9969722	-0.1417867064	3.890019e-01	6.774824e-01
0.9949051	-0.2228666204	2.202721e-01	0.8800012	0.1585571399	5.811031e-01	8.225914e-01
0.8394240	-0.5534007967	1.460414e-01	0.8181086	0.8116520412	4.070229e-01	6.952547e-01
0.8194410	0.5404552594	2.092852e-01	0.8699563	-0.4897969725	1.962700e-01	4.999809e-01
0.9210048	-0.3368928723	2.568330e-02	0.5838011	-0.0740022618	6.883582e-01	8.702025e-01
0.9949051	-0.1041991282	3.371202e-01	0.9366787	0.0094188359	9.410395e-01	9.835175e-01
0.8497626	-0.5871145393	3.878526e-02	0.6813116	-0.3341901616	3.569106e-01	6.629639e-01
0.9949051	0.6354113305	4.106374e-01	0.9430561	2.7104065622	3.926840e-04	6.537359e-03
0.9949051	0.0666916110	5.262206e-01	0.9641086	-0.2174575706	1.252452e-01	4.011918e-01
0.9949051	0.0040446452	9.812637e-01	0.9969722	-0.2748699044	1.033011e-01	3.707823e-01
0.9949051	0.0229370348	9.284662e-01	0.9969722	1.1367035357	1.182049e-03	1.440394e-02
0.8264615	0.4578842547	3.096130e-01	0.9288274	-0.3684211142	1.195076e-01	3.995752e-01
0.9949051	-0.1367291015	1.870961e-01	0.8526631	-0.1552983427	8.781231e-02	3.369940e-01
0.2019302	-0.0989501873	4.650261e-01	0.9641086	-0.1916625969	1.828061e-01	4.855658e-01
0.8540650	-0.0210224788	8.975701e-01	0.9969722	0.0861330610	6.129270e-01	8.394899e-01
0.9949051	0.0694716246	3.584951e-01	0.9430561	-0.0792406256	2.367203e-01	5.464911e-01
0.9949051	-0.0153634857	9.373863e-01	0.9969722	-0.0181652816	9.042711e-01	9.710339e-01
0.5172057	-0.0185604990	9.194666e-01	0.9969722	0.0545224666	7.093463e-01	8.762099e-01
0.2019302	-0.0765815337	5.294235e-01	0.9641086	-0.2023238754	3.078472e-02	1.847083e-01
0.8791060	-0.0470667007	6.959512e-01	0.9733364	-0.1836422397	3.073148e-02	1.847083e-01
0.9949051	-0.2211528378	6.272780e-01	0.9689755	0.1989328722	6.555443e-01	8.556317e-01
0.9949051	-1.1266972237	2.699760e-01	0.9106469	0.8570605889	5.321494e-01	7.882615e-01
0.8497626	0.9180332772	1.964474e-01	0.8526631	0.4808493156	2.666159e-01	5.814391e-01
0.9949051	-0.4079797313	3.872185e-03	0.2875687	-0.1253413245	4.124492e-01	6.984455e-01
0.9715057	0.3264972326	1.777733e-01	0.8526631	-0.4150310213	8.152986e-02	3.294921e-01
0.9949051	-0.0396432286	9.648196e-01	0.9969722	4.2258131673	4.645747e-04	7.131524e-03
0.9949051	-0.2111920438	5.475821e-01	0.9641086	-0.9546605263	1.668654e-02	1.188040e-01
0.9949051	-0.1801084018	7.777591e-02	0.7921508	-0.0726769562	4.755940e-01	7.506714e-01
0.9949051	-0.2485333226	5.830121e-01	0.9641086	-0.1984665470	6.482469e-01	8.523113e-01
0.9949051	-0.1283642738	1.124598e-01	0.8166268	-0.0860517402	3.165690e-01	6.316783e-01
0.9949051	0.0698827740	6.597313e-01	0.9689755	0.1832976633	3.025248e-01	6.208061e-01
0.9949051	-0.3006975349	6.530147e-03	0.3216097	-0.0678469099	6.334365e-01	8.479297e-01
0.9210048	-0.0103249030	9.590722e-01	0.9969722	-0.1952357445	2.879400e-01	6.045220e-01
0.8264615	0.1328474027	5.722439e-01	0.9641086	0.0259281201	8.286460e-01	9.372819e-01
0.8329325	-0.0996459332	4.393129e-01	0.9482489	-0.1161674086	1.507444e-01	4.410394e-01
0.8540650	-0.0162874636	8.684248e-01	0.9969722	-0.1020077932	2.131396e-01	5.215962e-01
0.9275791	-0.0107091728	9.032781e-01	0.9969722	-0.2061810699	5.561772e-02	2.661544e-01
0.9949051	0.1628318670	1.662940e-01	0.8467562	-0.2023073634	5.908739e-02	2.771480e-01
0.9998859	0.0861176218	4.152592e-01	0.9430561	-0.2175341330	6.168619e-03	5.441274e-02
0.9949051	0.0229789152	9.779757e-01	0.9969722	2.8851022132	1.636141e-03	1.834638e-02
0.9949051	-0.5422254266	1.060212e-04	0.0626585	0.0336889971	8.736820e-01	9.553119e-01
0.9949051	-0.0858104188	8.194457e-01	0.9957784	1.9409901437	3.057407e-04	5.646648e-03
0.9949051	0.2832449394	6.766091e-01	0.9717521	2.4977297885	4.037273e-04	6.625290e-03
0.9949051	0.0323041968	9.382643e-01	0.9969722	1.5444303387	3.540918e-04	6.154948e-03
0.9949051	-0.2192403355	1.596719e-01	0.8388097	0.1634864074	2.424168e-01	5.531595e-01
0.9949051	0.0960528469	4.538516e-01	0.9579510	-0.3478965251	7.592442e-04	1.008345e-02
0.8394240	-0.2910741926	6.932220e-02	0.7921508	-0.1731938070	2.380653e-01	5.474575e-01
0.9148164	-0.1471202801	1.834579e-01	0.8526631	-0.0281115565	8.104941e-01	9.247143e-01
0.9923046	-0.0727146480	3.790625e-01	0.9430561	-0.0459885197	6.264615e-01	8.433108e-01
0.9109219	-0.2199884325	6.572164e-01	0.9689755	0.4299499276	2.726150e-01	5.858743e-01
0.8653339	-0.0340661562	7.190266e-01	0.9733364	-0.0675208993	3.747039e-01	6.700454e-01
0.6925121	-0.1771859926	6.881394e-02	0.7921508	-0.1144740182	2.954840e-01	6.138173e-01
0.8540650	-0.1035747726	8.995534e-02	0.7921508	-0.0938354349	1.647790e-01	4.666833e-01
0.9949051	0.4281510072	5.267260e-01	0.9641086	-0.4320790131	1.768132e-01	4.802300e-01
0.2034892	-0.0728322880	4.848915e-01	0.9641086	-0.1665855930	1.038316e-01	3.707823e-01
0.8394240	-0.2488023865	7.156431e-02	0.7921508	0.0314899063	7.923673e-01	9.155212e-01
0.9210048	-0.0206471607	9.407901e-01	0.9969722	0.1979997903	5.836953e-01	8.247616e-01
0.8394240	-0.2132625988	5.488312e-01	0.9641086	-0.4046176860	9.869967e-02	3.627027e-01
0.8477725	0.0627138900	6.061663e-01	0.9641086	0.0760718924	3.283937e-01	6.412262e-01
0.9949051	0.9993357920	1.162104e-01	0.8166268	-0.0980792079	8.640418e-01	9.515996e-01
0.9949051	-0.0653224226	7.989710e-01	0.9957784	-0.3803278336	3.292930e-01	6.412262e-01
0.7475613	0.0194154446	8.595979e-01	0.9969722	-0.1343527022	3.598114e-01	6.629639e-01
0.9949051	-0.0794943969	5.159296e-01	0.9641086	0.0204196066	8.878287e-01	9.645345e-01
0.8497626	-0.0529715960	6.168096e-01	0.9641086	0.0701239585	4.553453e-01	7.382965e-01
0.9949051	0.0649552462	7.086701e-01	0.9733364	-0.0802244142	5.656783e-01	8.104628e-01
0.9210048	-0.0844967748	6.158404e-01	0.9641086	-0.2370933348	1.372974e-01	4.237219e-01
0.9949051	-0.0949141124	4.967976e-01	0.9641086	-0.0664041167	6.805929e-01	8.659428e-01
0.9210048	-0.0310676718	7.170803e-01	0.9733364	-0.1866186735	1.070037e-03	1.331351e-02
0.9949051	-0.0779912919	3.022580e-01	0.9286466	-0.0886164965	3.641905e-01	6.629639e-01
0.9949051	0.7073858247	1.589389e-01	0.8386867	1.7963135486	1.559487e-03	1.800451e-02
0.9949051	-0.5868543157	2.619661e-01	0.9053915	0.2631050662	6.771805e-01	8.656656e-01
0.9949051	-0.0334526752	7.241920e-01	0.9733364	-0.1579207705	7.010974e-02	2.991687e-01
0.9949051	-0.0407758664	7.295280e-01	0.9733364	-0.0444803546	7.143908e-01	8.762099e-01
0.6922402	-0.0009767085	9.947235e-01	0.9997294	0.1863321730	1.587182e-01	4.553517e-01
0.9949051	-0.7960505771	4.238795e-01	0.9430561	5.2884235584	1.280058e-02	9.889073e-02
0.9516289	-0.1444825136	6.611851e-01	0.9689755	-0.4849283992	1.222463e-01	3.995752e-01
0.9949051	0.0426059273	9.357013e-01	0.9969722	2.0898045188	1.451665e-04	3.299747e-03
0.9949051	0.1666841695	5.903330e-01	0.9641086	-0.1010852537	6.796496e-01	8.656744e-01
0.9949051	-0.3348391423	6.944487e-02	0.7921508	-0.0714795791	6.414254e-01	8.512682e-01
0.9949051	0.0798255114	6.927024e-01	0.9733364	-0.1656505368	3.860333e-01	6.760473e-01
0.9284320	0.1425604487	7.371769e-01	0.9767472	1.3368082893	1.779382e-03	1.947435e-02
0.9949051	-0.4198325541	4.400657e-03	0.2875687	-0.0278121809	8.595470e-01	9.515996e-01
0.8598556	-0.1214929135	1.427021e-01	0.8166268	-0.0736001491	2.471536e-01	5.585765e-01
0.9949051	0.3186403883	9.680207e-02	0.7921508	0.1021300401	5.895547e-01	8.264179e-01
0.7251512	0.1821296546	1.776353e-01	0.8526631	0.0233627383	8.376786e-01	9.420896e-01
0.9923046	-0.0919598892	7.109322e-01	0.9733364	-0.1089151087	7.213637e-01	8.762099e-01
0.8588222	-0.2893873307	9.356815e-02	0.7921508	-0.2412730326	6.436014e-02	2.838570e-01
0.9949051	-0.0713472196	7.898047e-01	0.9957784	1.0342094226	5.547768e-04	8.300585e-03
0.9294063	-0.1579320936	1.431009e-01	0.8166268	-0.0662561668	4.524696e-01	7.363525e-01

0.8210171	-0.3516465080	5.767453e-01	0.9641086	1.6087761796	2.767826e-01	5.897332e-01
0.8805172	-0.4459560167	1.574044e-02	0.4760109	-0.0962642704	5.464724e-01	7.942706e-01
0.9949051	0.0510162782	9.193793e-01	0.9969722	-0.3790021824	4.595197e-01	7.420113e-01
0.9949051	-0.0430714128	9.258165e-01	0.9969722	1.9694488423	4.565904e-05	1.686531e-03
0.9949051	0.3106335053	7.943788e-01	0.9957784	-2.6192625747	4.099895e-02	2.233215e-01
0.9981675	-0.1488634710	1.367642e-01	0.8166268	-0.0104498783	9.319049e-01	9.835175e-01
0.8394240	-0.1193548064	3.256547e-01	0.9364851	0.0022086485	9.863212e-01	9.950155e-01
0.9949051	0.0047943347	9.784033e-01	0.9969722	-0.1464366139	4.212185e-01	7.092312e-01
0.9949051	-0.1204193040	5.098270e-01	0.9641086	-0.1799138617	3.227101e-01	6.359740e-01
0.9949051	-0.3614233983	1.718722e-01	0.8467562	-0.0318475303	9.052332e-01	9.710339e-01
0.9949051	0.3156093377	4.153149e-01	0.9430561	1.3782416160	2.202358e-03	2.303706e-02
0.9949051	0.1879128682	7.005420e-01	0.9733364	2.4301113365	3.746681e-04	6.418228e-03
0.9949051	-0.0729164062	4.385100e-01	0.9482489	-0.0220762974	8.661113e-01	9.515996e-01
0.9949051	0.0024489177	9.814890e-01	0.9969722	-0.1275462088	2.750966e-01	5.881219e-01
0.9949051	-0.0421807757	7.795931e-01	0.9932650	-0.3951628125	7.905818e-03	6.722790e-02
0.9949051	0.0476507989	8.256536e-01	0.9964090	-0.1110991451	6.970188e-01	8.762099e-01
0.9949051	-0.1745052469	1.930189e-01	0.8526631	-0.2269762969	1.296856e-01	4.087689e-01
0.9949051	0.0320230164	7.504419e-01	0.9823061	0.0300889083	6.988954e-01	8.762099e-01
0.9949051	-0.0113461445	9.486583e-01	0.9969722	-0.0038887179	9.822698e-01	9.950155e-01
0.9949051	0.0365141921	8.886158e-01	0.9969722	0.9926491750	4.620883e-04	7.131524e-03
0.8264615	-0.1290226883	2.110439e-01	0.8699563	-0.0839356037	4.754919e-01	7.506714e-01
0.9949051	0.6380675532	5.040001e-01	0.9641086	5.3653555399	1.056252e-06	2.496980e-04
0.9949051	-0.0108294120	9.058574e-01	0.9969722	-0.1331853868	1.765036e-01	4.802300e-01
0.9949051	-0.0060855478	9.719637e-01	0.9969722	0.1346585456	3.614711e-01	6.629639e-01
0.9949051	-0.0586468072	4.929364e-01	0.9641086	-0.0355808611	7.887334e-01	9.122142e-01
0.9949051	-0.1645475294	9.461341e-02	0.7921508	-0.0379340105	7.055155e-01	8.762099e-01
0.8194410	-0.1966575230	1.118337e-02	0.3776783	-0.0732829051	3.228295e-01	6.359740e-01
0.8449829	-0.0156804392	8.320268e-01	0.9964090	0.0090910818	8.597024e-01	9.515996e-01
0.9696873	-0.1325443697	3.675193e-01	0.9430561	-0.0488164722	7.514101e-01	8.876755e-01
0.8103326	0.1786817645	5.828668e-02	0.7921508	-0.2038435199	1.669834e-01	4.676259e-01
0.8194410	0.1728216093	1.717585e-01	0.8467562	-0.0736490001	5.684697e-01	8.127545e-01
0.9949051	0.1118468871	7.044427e-01	0.9733364	-0.2354027487	2.978875e-01	6.166427e-01
0.9949051	0.3739023458	2.410562e-01	0.8975264	0.0247476738	9.332694e-01	9.835175e-01
0.9923046	-0.1474568439	3.292335e-01	0.9366787	-0.3108686053	1.213293e-01	3.995752e-01
0.9949051	0.1438361412	2.762992e-01	0.9110414	-0.4467483873	2.078530e-02	1.409284e-01
0.8194410	1.0421160292	2.847210e-01	0.9245610	-0.3399724154	3.782017e-01	6.707992e-01
0.9949051	0.0284998615	7.886937e-01	0.9957784	-0.0580947180	6.290773e-01	8.433108e-01
0.8194410	-0.0031249886	9.780749e-01	0.9969722	-0.0535121832	6.976653e-01	8.762099e-01
0.8540650	0.0354133363	7.800733e-01	0.9932650	0.0686615971	5.704384e-01	8.131842e-01
0.9923046	-0.1683011521	1.001920e-01	0.8015257	-0.1715609305	6.579759e-02	2.881189e-01
0.9949051	-0.0955092362	3.879496e-01	0.9430561	-0.0573476120	5.907979e-01	8.264179e-01
0.9949051	-0.0991354380	3.585338e-01	0.9430561	-0.2236426157	4.561489e-02	2.354314e-01
0.9210048	-0.0113837816	9.176491e-01	0.9969722	-0.0590531407	4.561558e-01	7.385974e-01
0.9949051	0.1855440842	1.742355e-01	0.8526631	-0.1937807402	1.046275e-01	3.724992e-01
0.9949051	0.1054459168	6.262073e-01	0.9689755	-0.2220321931	2.100501e-01	5.205014e-01
0.9949051	0.0156134398	9.325449e-01	0.9969722	0.3348584011	4.655485e-02	2.371889e-01
0.8805172	0.1337559248	2.563244e-01	0.9016579	-0.0183914231	8.633485e-01	9.515996e-01
0.9949051	0.0659515980	5.872580e-01	0.9641086	-0.0387963887	7.097710e-01	8.762099e-01
0.5133973	0.3730592099	1.875218e-02	0.5037516	-0.1072424436	5.717056e-01	8.131842e-01
0.7342939	0.4336279223	4.236519e-01	0.9430561	-0.3038156954	2.516217e-01	5.601071e-01
0.9949051	0.0502869977	7.121789e-01	0.9733364	-0.0962147415	4.101671e-01	6.975791e-01
0.9949051	-0.0907766256	6.353970e-01	0.9689755	1.0088288223	9.727631e-04	1.236350e-02
0.2019302	0.0372301561	8.312929e-01	0.9964090	-0.4391941684	2.836400e-03	2.865491e-02
0.9961627	-0.4167726055	7.047349e-02	0.7921508	-0.0710030989	7.575410e-01	8.905775e-01
0.6918844	-0.0448159623	8.599625e-01	0.9969722	0.0841688723	8.066371e-01	9.220939e-01
0.9949051	-0.4784299389	9.877313e-04	0.1459373	0.1253417769	5.361860e-01	7.882615e-01
0.9379096	-0.0615308648	8.091276e-01	0.9957784	-0.2652541866	1.459799e-01	4.367095e-01
0.8394240	-0.2567925007	4.042904e-01	0.9430561	0.6238697864	6.109396e-02	2.794946e-01
0.9210048	-0.4724133125	2.914427e-01	0.9286466	0.8245595758	4.743601e-01	7.506714e-01
0.6918369	-0.1646157941	4.478956e-01	0.9515413	-0.0908774738	7.088593e-01	8.762099e-01
0.9148164	-0.1708771402	4.119431e-01	0.9430561	-0.1704881454	3.802330e-01	6.707992e-01
0.9961627	0.2478058761	2.492339e-01	0.8975264	0.0564826868	7.599672e-01	8.905775e-01
0.8394240	-0.3056907202	2.211421e-01	0.8801009	-0.0816929016	8.020214e-01	9.207928e-01
0.9210048	-0.2164503197	3.422283e-01	0.9398602	-0.1090806022	5.123697e-01	7.784331e-01
0.9949051	0.1557309211	6.368198e-01	0.9689755	-0.1074964127	6.191226e-01	8.433108e-01
0.9949051	0.0800022023	8.852530e-01	0.9969722	-1.2503980474	2.893664e-02	1.785439e-01
0.9696873	-0.6709313928	3.811771e-02	0.6813116	-0.9419861023	7.588287e-03	6.546975e-02
0.7251512	-0.1708406355	5.241186e-01	0.9641086	0.0704294519	8.465154e-01	9.481630e-01
0.7251512	0.3965308336	2.015009e-01	0.8526631	0.3622138181	1.668769e-01	4.676259e-01
0.5190755	0.0939657689	7.122834e-01	0.9733364	0.1169247913	6.713910e-01	8.607204e-01
0.9949051	-0.2703396510	1.972323e-01	0.8526631	0.0953291516	6.268656e-01	8.433108e-01
0.9949051	-0.5028163687	9.527446e-02	0.7921508	-0.0494008677	8.691773e-01	9.529580e-01
0.8194410	-0.3328617962	8.217172e-02	0.7921508	-0.1848420946	3.455661e-01	6.539822e-01
0.9949051	-0.6409817722	3.565965e-02	0.6690430	-0.5608838841	6.326061e-02	2.821662e-01
0.9148164	-0.1485299034	5.710135e-01	0.9641086	0.0459166703	8.285536e-01	9.372819e-01
0.5133973	-0.0494167844	7.993029e-01	0.9957784	-0.4721043072	2.649387e-03	2.703053e-02
0.8194410	-0.1087962582	4.189925e-01	0.9430561	-0.0759578082	5.279077e-01	7.878622e-01
0.8194410	-0.1301341038	5.527712e-01	0.9641086	-0.1093998825	5.808879e-01	8.225914e-01
0.9949051	-0.2463115749	2.770426e-01	0.9110414	0.1473967714	4.900262e-01	7.621198e-01
0.8394240	-0.3051882628	2.261823e-01	0.8836164	-0.1769775402	5.236822e-01	7.867567e-01
0.7685693	-0.3349698256	9.714107e-03	0.3705021	-0.1658900022	4.722032e-01	7.506714e-01
0.7811016	-0.1286931577	5.133156e-01	0.9641086	-0.0849883384	7.220207e-01	8.762099e-01
0.7696781	0.1997951041	6.468605e-01	0.9689755	-0.1341766122	6.200152e-01	8.433108e-01
0.9949051	-0.6726475261	1.109809e-01	0.8166268	-0.4817135012	2.260415e-01	5.365081e-01
0.8477725	0.0904511462	7.619705e-01	0.9864723	-0.0272829907	9.218517e-01	9.834194e-01
0.9696873	-0.6979925682	9.596489e-05	0.0626585	-0.0526826819	8.138570e-01	9.267620e-01
0.9949051	-3.3833259298	2.247950e-01	0.8836164	-3.4969965871	2.181499e-01	5.283877e-01
0.9210048	0.8455320768	1.973725e-01	0.8526631	0.5441676927	4.916797e-01	7.636864e-01
0.9949051	-0.1954254275	6.115971e-02	0.7921508	-0.2735112051	1.345016e-02	1.030432e-01
0.9949051	-0.7056160858	6.347644e-03	0.3216097	-0.4165572137	1.760153e-01	4.802300e-01
0.9923046	0.4658680243	1.406403e-01	0.8166268	-0.5649771373	2.396793e-01	5.481636e-01
0.9949051	0.1081622055	6.680688e-01	0.9717521	-0.1257877021	6.465856e-01	8.520225e-01
0.9949051	-0.0770433130	3.529962e-01	0.9430561	-0.0926231801	2.140411e-01	5.227202e-01
0.9923046	-0.0799328916	4.730390e-01	0.9641086	-0.1966698507	2.047892e-01	5.128408e-01
0.9949051	-0.1318602814	4.349356e-01	0.9482489	0.0966061902	6.387004e-01	8.511205e-01
0.9949051	-0.0352137990	8.027971e-01	0.9957784	-0.2623285776	3.798606e-02	2.110347e-01
0.9949051	0.0240315237	8.715882e-01	0.9969722	0.0109409355	9.428291e-01	9.835175e-01
0.9949051	-0.1049778071	6.578481e-01	0.9689755	-0.1494341151	4.999566e-01	7.644874e-01
0.8194410	-0.0575180224	8.123714e-01	0.9957784	-0.2049502237	3.075814e-01	6.214128e-01
0.9210048	0.1308204153	4.018579e-01	0.9430561	0.0175188724	8.981652e-01	9.695263e-01
0.8194410	-0.0023491116	9.839758e-01	0.9969722	-0.0167289489	8.027605e-01	9.207928e-01
0.9949051	-0.0034993749	9.810814e-01	0.9969722	-0.0492503131	7.172306e-01	8.762099e-01
0.9949051	-0.0133079381	9.793542e-01	0.9969722	2.6596973847	6.698964e-05	2.058028e-03
0.9949051	-0.0137743505	9.781453e-01	0.9969722	2.2639709916	2.213157e-04	4.433815e-03
0.9949051	0.4623142433	5.499141e-01	0.9641086	0.6903982974	3.860676e-01	6.760473e-01
0.9949051	0.4587297472	3.919744e-02	0.6813116	-0.0199909289	9.368667e-01	9.835175e-01
0.7815947	-0.0631906322	6.979741e-01	0.9733364	0.2576317772	2.481543e-01	5.587016e-01
0.2019302	0.4573622354	3.088199e-01	0.9288274	-0.5403261164	1.262554e-01	4.033348e-01
0.9210048	0.0421501867	7.284509e-01	0.9733364	0.1467682015	2.011534e-01	5.091291e-01
0.9949051	1.0065779145	1.340404e-01	0.8166268	-0.6269876284	3.336888e-02	1.952575e-01
0.9949051	0.1104873605	6.894031e-01	0.9733364	-0.0684526554	7.059256e-01	8.762099e-01
0.9210048	0.3919321992	2.973327e-01	0.9286466	-0.3327469763	1.103002e-01	3.834553e-01
0.4079598	-0.1591203274	2.464693e-01	0.8975264	-0.2223708984	1.597298e-01	4.560401e-01
0.9949051	0.5507822842	2.184505e-01	0.8800012	-0.2083458045	4.294232e-01	7.159073e-01
0.9949051	0.2663954025	5.681925e-01	0.9641086	-0.2132445594	3.963410e-01	6.839053e-01
0.8477725	0.2205502391	5.428313e-01	0.9641086	-0.0956446697	5.906405e-01	8.264179e-01
0.9135088	-0.0856087224	3.183453e-01	0.9300968	-0.1150383621	1.457037e-01	4.367095e-01
0.9696873	0.1618133049	4.474106e-01	0.9515413	-0.3479622229	5.414541e-02	2.612240e-01
0.9949051	0.0761402510	4.006189e-01	0.9430561	-0.1536513060	2.874392e-02	1.785439e-01
0.9949051	0.1888740276	1.057652e-01	0.8117824	-0.1748751033	1.357636e-01	4.216554e-01
0.9949051	0.0167423188	8.829314e-01	0.9969722	-0.2444207413	5.788072e-02	2.731360e-01
0.9210048	-0.0054773921	9.634656e-01	0.9969722	-0.1028464127	3.498657e-01	6.564148e-01
0.9949051	-0.4588287464	4.803122e-01	0.9641086	0.6299316645	3.540243e-01	6.621152e-01
0.9949051	-0.1664216257	1.011580e-01	0.8015257	-0.1125829760	3.903262e-01	6.774824e-01
0.9698380	0.0798197720	7.712092e-01	0.9919874	-0.1179497070	4.944697e-01	7.643323e-01
0.9824103	-0.0012218265	9.940929e-01	0.9997294	-0.2558658220	8.028501e-02	3.293325e-01
0.9210048	-0.9451264080	2.009678e-01	0.8526631	-0.1197057456	8.675346e-01	9.521132e-01
0.9949051	0.0857654803	5.978911e-01	0.9641086	0.0055818401	9.705643e-01	9.915358e-01
0.8612982	0.0233560111	8.906463e-01	0.9969722	-0.0557829148	7.544752e-01	8.900096e-01
0.9949051	0.0892163135	6.623792e-01	0.9689755	-0.0735453622	6.815341e-01	8.662079e-01
0.9949051	0.0556168331	6.116721e-01	0.9641086	0.4596371078	1.260156e-03	1.484355e-02
0.9949051	-0.0583325702	6.470408e-01	0.9689755	-0.2305427034	1.252028e-02	9.800240e-02
0.9949051	-0.1013159290	6.150510e-01	0.9641086	-0.3766365592	1.221855e-01	3.995752e-01
0.8194410	-0.0648845661	4.751580e-01	0.9641086	-0.0186958770	8.031619e-01	9.207928e-01
0.9949051	-0.6316699631	1.691409e-02	0.4760109	-0.5265231663	4.150680e-02	2.240230e-01
0.9949051	0.1191995614	5.282760e-01	0.9641086	-0.1198780522	5.365010e-01	7.882615e-01
0.8805172	0.1213395070	5.904150e-01	0.9641086	0.0800297622	7.263301e-01	8.773616e-01
0.9949051	-0.2924495603	2.177836e-01	0.8800012	0.0586259832	7.580106e-01	8.905775e-01
0.9949051	0.0251850580	8.091999e-01	0.9957784	0.0326382838	7.797427e-01	9.053594e-01

0.9949051	-0.0114201501	9.523048e-01	0.9969722	-0.0837007966	6.417793e-01	8.512682e-01
0.9949051	-0.1580565560	9.558527e-02	0.7921508	-0.2212158820	4.123288e-02	2.235654e-01
0.6918844	-0.3911833634	3.113927e-02	0.6526535	-0.0339582422	8.504188e-01	9.481630e-01
0.9949051	0.2924679757	5.927756e-01	0.9641086	-0.1522495946	6.450009e-01	8.512682e-01
0.9949051	-0.0669769873	3.757158e-01	0.9430561	-0.1265056030	6.906611e-02	2.968587e-01
0.9275791	-0.0076222495	9.469195e-01	0.9969722	-0.1408528477	3.612140e-01	6.629639e-01
0.7685693	-0.0384150041	8.309727e-01	0.9964090	-0.2310558802	2.052902e-01	5.130084e-01
0.9949051	0.3300329318	5.542799e-01	0.9641086	1.7386498022	5.851034e-04	8.538176e-03
0.9949051	-0.1081568540	2.503880e-01	0.8975264	-0.2075327307	2.141813e-02	1.438422e-01
0.9949051	0.2473287565	7.143279e-01	0.9733364	0.0637186043	8.570337e-01	9.513693e-01
0.9949051	-0.2073380684	6.102525e-01	0.9641086	1.1129211407	5.445028e-06	6.436023e-04
0.9949051	0.6460223422	2.367285e-01	0.8975264	1.8171481398	1.393720e-05	8.670406e-04
0.9867694	-0.0623976419	9.112599e-01	0.9969722	2.4121790777	6.256440e-04	8.909773e-03
0.5937207	1.2333131868	2.940834e-01	0.9286466	-0.7989087867	3.551737e-01	6.629639e-01
0.9949051	0.0093872794	9.621745e-01	0.9969722	-0.3821481334	6.260997e-02	2.813791e-01
0.2019302	-0.0758999862	5.550568e-01	0.9641086	-0.2805005266	1.260268e-02	9.800240e-02
0.9949051	0.4230931817	4.007341e-01	0.9430561	1.8400100172	1.404287e-04	3.254641e-03
0.9379096	0.2292415507	5.941712e-01	0.9641086	1.7940745340	5.210093e-05	1.811274e-03
0.9949051	0.1751490751	5.148072e-01	0.9641086	-0.1863962616	3.617139e-01	6.629639e-01
0.8598556	-0.3767710703	3.299151e-01	0.9366787	0.0860508872	7.955011e-01	9.182445e-01
0.9949051	0.1323852064	3.895482e-01	0.9430561	-0.2013213164	1.861433e-01	4.889364e-01
0.8730639	0.1092349927	4.042711e-01	0.9430561	-0.1083578169	3.038843e-01	6.214128e-01
0.9653626	0.1821730082	2.324850e-01	0.8921990	0.0872138794	5.368098e-01	7.882615e-01
0.9949051	0.0222685393	8.452639e-01	0.9969722	-0.0053503766	9.669478e-01	9.912683e-01
0.8612982	0.5693359534	4.052670e-01	0.9430561	-0.1287690076	7.971794e-01	9.183880e-01
0.9949051	0.2038548079	4.483997e-01	0.9515413	0.3298580865	9.506257e-02	3.558867e-01
0.9949051	-0.0273983124	7.723893e-01	0.9920566	-0.1449729497	1.288687e-01	4.082215e-01
0.9949051	0.5312803534	3.441100e-01	0.9398602	0.9064074591	8.006445e-02	3.293325e-01
0.9949051	0.3767893821	3.372699e-01	0.9366787	-0.1528362965	5.205836e-01	7.867567e-01
0.9949051	-0.1460639730	6.303056e-01	0.9689755	-0.6251562976	1.351243e-02	1.030432e-01
0.5133973	0.1381828053	3.693345e-01	0.9430561	-0.1540203936	1.922145e-01	4.928717e-01
0.9210048	0.1478493862	3.090105e-01	0.9288274	0.1229449993	1.922283e-01	4.928717e-01
0.9949051	0.1211202013	3.957342e-01	0.9430561	-0.2609817606	1.011780e-01	3.676741e-01
0.9949051	0.0342578988	7.896756e-01	0.9957784	-0.0975930700	3.765294e-01	6.707992e-01
0.9949051	0.6509797091	4.736360e-01	0.9641086	3.9514859567	5.446421e-03	4.914252e-02
0.9379096	-0.1249860136	2.532702e-01	0.9016579	-0.1806816890	6.021651e-02	2.791213e-01
0.7194638	-0.0193232862	8.878891e-01	0.9969722	0.0025156190	9.862754e-01	9.950155e-01
0.9949051	-0.2191234688	3.983609e-01	0.9430561	-0.2735563585	3.625748e-01	6.629639e-01
0.8394240	-0.0358130352	8.682732e-01	0.9969722	-0.2233176456	1.614376e-01	4.598055e-01
0.8598556	-0.4341910787	1.172741e-02	0.3850499	-0.1938012136	3.086403e-01	6.214870e-01
0.2019302	0.1666719547	2.741915e-01	0.9110414	-0.1864665618	3.611635e-02	2.050213e-01
0.9509918	0.0321940384	9.032744e-01	0.9969722	-0.0545382434	8.503270e-01	9.481630e-01
0.9949051	-0.1799054526	5.133389e-01	0.9641086	1.0280256829	2.458112e-04	4.762236e-03
0.9949051	0.0517711807	5.792207e-01	0.9641086	0.1486943073	2.006958e-01	5.090609e-01
0.9692638	0.1273632113	5.953813e-01	0.9641086	-0.1843200406	3.404845e-01	6.522734e-01
0.9949051	-0.0542207855	5.509168e-01	0.9641086	0.0864467721	2.397636e-01	5.481636e-01
0.9148164	0.1936117693	4.871685e-01	0.9641086	-0.1214228191	6.359460e-01	8.493805e-01
0.9949051	-0.1917263249	1.047367e-01	0.8091427	0.0479536871	7.211710e-01	8.762099e-01
0.9949051	-0.0482921183	6.169211e-01	0.9641086	-0.2397396858	1.545543e-02	1.120756e-01
0.8394240	-0.0290547749	8.594006e-01	0.9969722	0.0238078284	8.955353e-01	9.686740e-01
0.9949051	-0.2915928425	5.897041e-02	0.7921508	-0.1908167290	1.406911e-01	4.313058e-01
0.9551136	-0.1143342869	1.551043e-01	0.8386867	-0.1624837070	4.683461e-02	2.375902e-01
0.7194638	-0.2319468099	3.957647e-02	0.6813116	-0.0014616961	9.876889e-01	9.950155e-01
0.9949051	-0.3261335275	9.210699e-03	0.3705021	-0.1230906483	4.035875e-01	6.943819e-01
0.9949051	0.1795472181	1.699390e-01	0.8467562	-0.2070402844	1.989182e-01	5.056372e-01
0.9949051	0.0739583978	8.195038e-01	0.9957784	1.4996359235	1.281213e-05	8.413298e-04
0.9949051	-0.0536192829	5.635648e-01	0.9641086	-0.0302882036	7.465429e-01	8.850046e-01
0.9949051	0.4054888382	1.118903e-01	0.8166268	0.2457963542	2.585589e-01	5.701802e-01
0.8451285	0.1641320536	2.275026e-01	0.8836164	0.0314350989	7.601172e-01	8.905775e-01
0.9949051	0.1132288491	3.369239e-01	0.9366787	0.1386314671	2.447793e-01	5.554637e-01
0.9210048	0.5187791488	3.069429e-01	0.9288274	1.5253306990	7.533647e-03	6.546975e-02
0.9949051	0.0660789260	6.517749e-01	0.9689755	0.1950565895	1.962457e-01	4.999809e-01
0.8394240	-0.1096727484	1.381728e-01	0.8166268	-0.0469403498	5.568462e-01	8.036535e-01
0.7342939	0.1573711730	2.774745e-01	0.9110414	0.0298962152	8.541642e-01	9.506799e-01
0.8210171	0.0146063248	9.405175e-01	0.9969722	-0.3028524282	2.669969e-02	1.699335e-01
0.8477725	0.0280438132	7.879235e-01	0.9957784	-0.0799223863	3.603204e-01	6.629639e-01
0.9949051	0.0828922569	5.389396e-01	0.9641086	-0.2812819300	5.817472e-03	5.179572e-02
0.9949051	-0.1328659922	6.235455e-02	0.7921508	-0.1660982995	5.171483e-02	2.536387e-01
0.7815947	0.1248193633	1.588364e-01	0.8386867	-0.0747500147	2.865522e-01	6.045220e-01
0.9949051	0.0008284783	9.898433e-01	0.9997294	-0.0933595111	3.683457e-01	6.675072e-01
0.9949051	0.0397603075	9.504990e-01	0.9969722	-0.9833463937	8.587943e-02	3.339128e-01
0.8539066	-0.2018314511	4.721934e-02	0.7645652	0.0204554661	8.662616e-01	9.515996e-01
0.9949051	0.0359512868	8.345855e-01	0.9969722	0.0368521922	8.662238e-01	9.515996e-01
0.8477725	0.6592121363	3.863685e-01	0.9430561	-0.6958526914	1.312120e-01	4.113862e-01
0.9949051	0.3361699636	5.050084e-01	0.9641086	2.0567723378	5.051266e-05	1.809271e-03
0.9824103	0.2078738307	1.220082e-01	0.8166268	0.0609203110	6.704145e-01	8.607204e-01
0.9949051	-0.0945356873	2.695872e-01	0.9106469	0.0293818957	6.833713e-01	8.676100e-01
0.9949051	0.0387060005	7.058140e-01	0.9733364	0.0103209634	9.341952e-01	9.835175e-01
0.9380386	-0.0122629346	9.491299e-01	0.9969722	-0.0928400797	5.483290e-01	7.942706e-01
0.9210048	0.0431877675	8.912183e-01	0.9969722	0.4817423073	7.929895e-02	3.288820e-01
0.9949051	-0.0152445330	8.645048e-01	0.9969722	-0.1881763944	1.542282e-01	4.468082e-01
0.9949051	0.0208464878	8.794803e-01	0.9969722	-0.2220628603	1.691319e-01	4.714951e-01
0.9551136	0.0786918962	7.875836e-01	0.9957784	-0.0242245602	9.410234e-01	9.835175e-01
0.8741974	0.0338025263	6.925196e-01	0.9733364	0.0049457490	9.751245e-01	9.927624e-01
0.6504978	0.2943718771	1.655989e-01	0.8467562	-0.0613708702	5.223210e-01	7.867567e-01
0.9949051	0.1229279473	1.272207e-01	0.8166268	0.1048273104	1.908377e-01	4.925112e-01
0.9949051	0.5187674220	2.023446e-01	0.8526631	0.7217565661	1.195949e-01	3.995752e-01
0.8805172	-0.3178338357	7.907473e-02	0.7921508	-0.1976717154	2.275299e-01	5.378807e-01
0.9696873	0.0915597369	3.034874e-01	0.9286466	-0.0338479778	7.438376e-01	8.842500e-01
0.2019302	-0.1029683072	5.247132e-01	0.9641086	0.3181406705	8.080069e-02	3.293325e-01
0.9949051	0.1478724400	7.596429e-01	0.9855489	2.5185010666	6.909292e-05	2.058028e-03
0.9949051	-0.2479498339	4.012810e-01	0.9430561	-0.2534335033	4.463852e-01	7.297749e-01
0.8791060	-0.3544415635	4.167783e-01	0.9430561	-0.4678151374	2.973950e-01	6.166427e-01
0.9949051	-0.1381717002	3.785216e-01	0.9430561	-0.4343895454	3.656435e-02	2.058050e-01
0.9210048	-0.2209737865	6.262979e-02	0.7921508	-0.2182883186	1.359143e-01	4.216554e-01
0.9949051	-0.4489106274	1.699689e-01	0.8467562	0.1409568200	6.452930e-01	8.512682e-01
0.9949051	-0.0797721904	7.123217e-01	0.9733364	-0.3771030338	7.886909e-02	3.288820e-01
0.9949051	-0.0075761909	9.457212e-01	0.9969722	0.0678237047	6.286058e-01	8.433108e-01
0.9923046	0.2142492779	3.306911e-01	0.9366787	-0.2411491240	4.064870e-01	6.952547e-01
0.8805172	-0.2004545973	1.117559e-01	0.8166268	-0.2281545467	1.678533e-02	1.188040e-01
0.9949051	-0.0955432760	6.519019e-01	0.9689755	-0.4009904186	7.915589e-02	3.288820e-01
0.9949051	-0.0167780092	9.356283e-01	0.9969722	-0.2130338686	4.284445e-01	7.159073e-01
0.9949051	-0.2313105445	1.005697e-02	0.3705021	0.1096009249	3.728729e-01	6.687949e-01
0.9949051	0.0742941882	7.076242e-01	0.9733364	-0.6177818268	4.069697e-03	3.848305e-02
0.9148164	0.1493966886	2.972268e-01	0.9286466	-0.1048538081	4.422842e-01	7.260833e-01
0.9949051	-0.1181548212	1.440466e-01	0.8166268	-0.1148021501	2.284548e-01	5.383552e-01
0.9949051	-0.0541717845	7.440590e-01	0.9796283	0.0733738814	6.148932e-01	8.412081e-01
0.8194410	0.1833818613	5.246643e-01	0.9641086	-0.0934247134	6.160143e-01	8.417676e-01
0.8394240	0.2942490597	3.513169e-01	0.9430561	-0.1113601212	5.343458e-01	7.882615e-01
0.9949051	0.2065755370	5.767916e-01	0.9641086	-0.0029207405	9.913112e-01	9.950155e-01
0.9949051	0.2260056126	3.456021e-01	0.9398602	0.9640374148	1.268357e-03	1.484355e-02
0.9509918	0.1153930241	2.646796e-01	0.9092411	-0.0972035947	3.893371e-01	6.774824e-01
0.8730639	1.8081288647	3.632926e-01	0.9430561	1.8605757699	3.793746e-01	6.707992e-01
0.8540650	1.1702705618	2.619311e-01	0.9053915	1.3200239452	2.338754e-01	5.448022e-01
0.9949051	0.0303242678	7.573217e-01	0.9851603	0.0033798861	9.693483e-01	9.915358e-01
0.8394240	-0.6761895847	3.973893e-04	0.0782857	-0.0776626225	7.119162e-01	8.762099e-01
0.9696873	-0.4301659909	1.673223e-02	0.4760109	-0.4613217009	9.370340e-03	7.638443e-02
0.9949051	0.0289217847	9.664042e-01	0.9969722	-0.5030560462	4.365984e-01	7.217613e-01
0.9949051	-0.2346207231	7.800689e-02	0.7921508	-0.0335804335	8.494400e-01	9.481630e-01
0.8497626	-0.0306066651	9.284602e-01	0.9969722	0.2412131895	6.427646e-01	8.512682e-01
0.8194410	-0.3317459966	1.028759e-01	0.8036101	-0.1323017414	6.068565e-01	8.360191e-01
0.7815947	-0.2164368804	1.370864e-01	0.8166268	0.0036936830	9.789129e-01	9.950155e-01
0.6874099	-0.1071875972	6.163048e-01	0.9641086	-0.2314185738	3.056152e-01	6.214128e-01
0.9493730	-0.0704501702	2.906719e-01	0.9286466	-0.0993091927	2.130293e-01	5.215962e-01
0.8805172	-0.4790573605	6.361732e-02	0.7921508	-0.0233033024	9.422396e-01	9.835175e-01
0.9949051	0.0708292492	6.074481e-01	0.9641086	0.3739780326	6.630147e-02	2.881189e-01
0.9135088	-0.0726460912	2.895144e-01	0.9286466	-0.1523103404	7.913376e-02	3.288820e-01
0.9949051	-0.0834607372	6.164595e-01	0.9641086	0.1326390295	3.942544e-01	6.822968e-01
0.8477725	-0.1211565721	4.034487e-01	0.9430561	-0.0053007494	9.632933e-01	9.911619e-01
0.9949051	-0.0060644975	9.809554e-01	0.9969722	-0.2579944311	2.877175e-01	6.045220e-01
0.9949051	-0.1676740214	3.169611e-02	0.6526535	-0.0998219437	2.317920e-01	5.436074e-01
0.9923046	-0.4308670269	2.236344e-01	0.8836164	1.0027485596	5.678266e-04	8.389638e-03
0.9949051	0.1041444901	2.725680e-01	0.9110414	-0.0808709141	5.224828e-01	7.867567e-01
0.9949051	0.0777971346	7.604235e-01	0.9855489	1.1436852040	8.751541e-07	2.496980e-04
0.9949051	0.0178730052	8.390977e-01	0.9969722	-0.0471759376	6.248445e-01	8.433108e-01
0.9949051	0.1491288319	7.963975e-01	0.9957784	2.5123637927	1.360035e-04	3.215124e-03
0.9949051	0.6898246993	4.276036e-01	0.9478644	2.2981723828	8.656103e-03	7.222035e-02
0.6922402	-0.1038133410	1.447822e-01	0.8166268	0.0721687698	3.251104e-01	6.382430e-01
0.6925121	-0.1297632842	7.057858e-01	0.9733364	0.7085581144	1.788231e-01	4.809469e-01

0.8899481	0.4754288980	4.292473e-01	0.9478644	-0.0226841126	9.575417e-01	9.906899e-01
0.9949051	-0.2798716907	3.743670e-02	0.6813116	-0.0417314001	7.617376e-01	8.905775e-01
0.9949051	0.5204771416	3.518434e-01	0.9430561	2.1144802943	1.199883e-04	2.894413e-03
0.9949051	0.0498608432	6.067635e-01	0.9641086	-0.1068482210	2.776535e-01	5.897332e-01
0.9949051	-0.3456664168	3.253519e-03	0.2875687	-0.2594330050	5.800097e-02	2.731360e-01
0.8194410	-0.3066392728	2.286622e-01	0.8836164	-0.2669811317	3.451105e-01	6.539822e-01
0.9949051	0.1879761935	5.344340e-01	0.9641086	0.5990813949	5.357677e-03	4.871365e-02
0.8394240	2.2338882522	3.446684e-01	0.9398602	-1.4153876606	3.169821e-01	6.316783e-01
0.9949051	0.0128093612	9.181263e-01	0.9969722	-0.1621888348	2.769389e-01	5.897332e-01
0.8598556	-0.1802826462	7.078991e-01	0.9733364	-0.2362753952	6.047062e-01	8.359439e-01
0.8477725	-0.4693726041	7.064771e-02	0.7921508	0.0602431311	8.699748e-01	9.529580e-01
0.9949051	0.1769007075	3.781679e-01	0.9430561	0.6073990252	1.645276e-03	1.834638e-02
0.9379096	-0.4473254356	3.904793e-01	0.9430561	-0.7323396587	1.752605e-01	4.802300e-01
0.9210048	-0.1622917381	5.191269e-01	0.9641086	0.5573333448	8.452824e-02	3.330413e-01
0.9949051	0.0857807830	5.031747e-01	0.9641086	-0.2134533710	1.568290e-01	4.523748e-01
0.8497626	0.1999992518	5.787972e-01	0.9641086	-0.3475647995	3.957352e-02	2.175623e-01
0.8264615	-0.0604444468	6.499849e-01	0.9689755	-0.0226153575	8.895297e-01	9.646093e-01
0.9949051	0.5834521256	5.007482e-01	0.9641086	4.0021394629	1.670543e-05	9.872909e-04
0.9949051	0.6541515791	1.289600e-01	0.8166268	1.8529996240	7.187570e-05	2.072124e-03
0.9949051	0.8684203726	1.874190e-01	0.8526631	1.0152376396	1.574430e-01	4.527923e-01
0.9210048	-0.1260501035	2.112330e-01	0.8699563	-0.0876583293	3.065781e-01	6.214128e-01
0.9379096	-0.1902542246	6.782323e-02	0.7921508	-0.1546255553	2.702101e-01	5.849604e-01
0.9949051	-0.4491426292	9.218059e-02	0.7921508	-0.0977454649	6.614096e-01	8.563248e-01
0.9949051	0.1005251559	6.762806e-01	0.9717521	-0.0994049769	6.093706e-01	8.375303e-01
0.8329325	-4.4808299350	1.260611e-01	0.8166268	-3.3788657293	1.522183e-01	4.442521e-01
0.8394240	0.1562646849	2.791127e-01	0.9128476	0.0206242171	8.965561e-01	9.686740e-01
0.9949051	0.0078294357	9.552644e-01	0.9969722	-0.2683371638	3.485390e-02	2.004620e-01
0.9949051	-0.0799824409	3.131319e-01	0.9300968	-0.1077994622	3.384074e-01	6.512563e-01
0.8532963	0.2862641426	7.629527e-01	0.9866632	-0.2545801777	7.014386e-01	8.762099e-01
0.9949051	-0.1064699049	4.244550e-01	0.9430561	-0.0579675122	6.236646e-01	8.433108e-01
0.9949051	-0.1198646955	4.306287e-01	0.9478644	0.1545017813	4.268514e-01	7.146435e-01
0.9949051	0.3066009753	2.579799e-01	0.9021664	0.1237189095	6.859143e-01	8.689718e-01
0.9949051	0.2711756945	5.960226e-01	0.9641086	2.3511468127	1.802046e-05	1.014295e-03
0.8194410	0.0152274175	9.535961e-01	0.9969722	-0.2299247738	3.420842e-01	6.539822e-01
0.9949051	-0.1021224172	3.929676e-01	0.9430561	0.0177038521	8.926647e-01	9.671216e-01
0.9444828	1.5541474941	3.449347e-01	0.9398602	0.5646352767	3.790870e-01	6.707992e-01
0.9949051	0.1759731091	5.821209e-01	0.9641086	1.5624784042	3.757161e-05	1.586059e-03
0.8899481	0.4142831834	1.033407e-01	0.8036101	-0.1601894394	3.351592e-01	6.473173e-01
0.9949051	-0.0106791579	9.027952e-01	0.9969722	-0.2143708456	1.291665e-01	4.082215e-01
0.9949051	0.0531632820	7.803440e-01	0.9932650	-0.4363720334	2.829515e-02	1.785439e-01
0.9949051	0.2416548799	1.227830e-01	0.8166268	-0.3133405992	1.062622e-02	8.429655e-02
0.9949051	-0.1648683925	5.028007e-01	0.9641086	1.1920517739	7.951265e-05	2.237713e-03
0.9210048	0.0025793423	9.842888e-01	0.9969722	0.0012548502	9.933318e-01	9.950155e-01
0.9949051	0.5492614026	3.889304e-01	0.9430561	2.1103176715	1.599277e-03	1.817640e-02
0.9949051	0.3590252293	3.559308e-01	0.9430561	-0.0976816851	7.190759e-01	8.762099e-01
0.9949051	0.1628630378	1.710459e-01	0.8467562	-0.1383039305	1.746136e-02	1.221262e-01
0.9949051	-0.3241946506	8.544282e-03	0.3705021	-0.1994868223	1.634034e-01	4.642856e-01
0.9949051	0.1356647779	3.849728e-01	0.9430561	-0.1856125246	2.086873e-01	5.182110e-01
0.9949051	-0.3924401087	6.348943e-01	0.9689755	-1.6259305134	1.840673e-03	1.996032e-02
0.9210048	0.6306386040	6.198927e-02	0.7921508	0.6839209061	3.446131e-02	1.996729e-01
0.8194410	0.0672642890	5.378581e-01	0.9641086	-0.0327199906	7.830147e-01	9.081934e-01
0.9949051	0.0096322511	9.521762e-01	0.9969722	-0.0967774718	5.438340e-01	7.934534e-01
0.8805172	0.0409200743	8.075559e-01	0.9957784	-0.0787554906	7.472374e-01	8.850046e-01
0.9949051	-0.2499222006	6.946817e-04	0.1173020	0.0124005114	9.042656e-01	9.710339e-01
0.9949051	-0.2741394856	2.183758e-03	0.2868002	-0.0408428737	7.457435e-01	8.850046e-01
0.9949051	-0.3846000036	2.340057e-02	0.5531895	0.0172396733	9.306832e-01	9.835175e-01
0.7733569	0.4288153046	6.386070e-01	0.9689755	-0.0469287977	9.469943e-01	9.835175e-01
0.9949051	0.1802553465	5.937544e-01	0.9641086	1.8397460648	8.776333e-06	7.408616e-04
0.9949051	-0.0662094408	8.661196e-01	0.9969722	-0.1440980194	7.205106e-01	8.762099e-01
0.9949051	-0.0057522134	9.541226e-01	0.9969722	0.2051806391	1.246744e-01	4.004487e-01
0.9210048	-0.1245576332	1.170071e-01	0.8166268	-0.0324082623	6.735614e-01	8.625673e-01
0.9923046	0.2353159448	2.369364e-01	0.8975264	0.2606352681	1.708301e-01	4.751087e-01
0.9949051	0.4192410746	1.604325e-01	0.8390760	1.2597196899	9.514383e-05	2.499111e-03
0.9949051	-0.2236600015	9.469564e-02	0.7921508	0.4257587397	1.773099e-02	1.232826e-01
0.8805172	-0.0666715013	8.570028e-01	0.9969722	0.4139074171	2.719613e-01	5.856155e-01
0.9949051	0.0910197361	6.299617e-01	0.9689755	-0.5569445247	1.371412e-02	1.039108e-01
0.9949051	0.7994688049	3.813920e-01	0.9430561	-0.3119962003	3.496789e-01	6.564148e-01
0.9949051	-0.2979541771	3.592795e-01	0.9430561	1.6090116576	1.236307e-03	1.476075e-02
0.9923046	-0.1066579584	2.499421e-01	0.8975264	-0.1859289558	8.630441e-02	3.344650e-01
0.8329325	0.1495793578	6.506061e-01	0.9689755	0.4760923929	1.898931e-01	4.922230e-01
0.9917395	-0.0243897802	8.288528e-01	0.9964090	0.2892249221	1.815944e-02	1.255232e-01
0.9715057	-0.0254955067	8.551711e-01	0.9969722	-0.2529270653	4.601071e-02	2.354314e-01
0.9275791	-0.0404544968	6.889095e-01	0.9733364	0.1034493467	1.139665e-01	3.920865e-01
0.8329325	-0.2650901887	6.319306e-01	0.9689755	2.4666522972	5.828114e-03	5.179572e-02
0.8477725	-0.1747455865	2.312194e-01	0.8902324	-0.1397832205	3.324427e-01	6.431217e-01
0.9949051	0.1659275820	8.289908e-01	0.9964090	-0.6865642057	2.236629e-01	5.330032e-01
0.9949051	-0.0877657684	3.887632e-01	0.9430561	-0.1053063022	2.909097e-01	6.064468e-01
0.9949051	-0.0127170100	8.736695e-01	0.9969722	0.3748645397	7.657322e-03	6.558662e-02
0.8394240	0.4114268975	4.164180e-01	0.9430561	-0.5248965217	4.272187e-02	2.277429e-01
0.9284320	0.1282773059	7.789948e-01	0.9932650	-1.3855453737	2.900206e-02	1.785439e-01
0.8088570	0.0330940502	7.442523e-01	0.9796283	-0.2662004833	8.443199e-02	3.330413e-01
0.9949051	0.2783552264	3.862843e-01	0.9430561	0.1003461134	7.604379e-01	8.905775e-01
0.9949051	0.2275764852	7.217873e-01	0.9733364	2.9299143163	4.095438e-05	1.613603e-03
0.9949051	0.0457332636	6.578436e-01	0.9689755	-0.0655081370	3.590291e-01	6.629639e-01
0.9275791	0.0121025593	9.622646e-01	0.9969722	1.0296143858	3.185867e-04	5.718726e-03
0.9949051	-0.0904714274	8.475094e-01	0.9969722	-1.4971554150	9.769918e-03	7.855811e-02
0.9949051	0.0453719688	7.017433e-01	0.9733364	0.1127895821	3.709717e-01	6.676641e-01
0.9949051	0.4838292523	2.212680e-02	0.5337527	-0.0536247852	7.609499e-01	8.905775e-01
0.9949051	0.0723147787	4.884461e-01	0.9641086	-0.1070555373	3.455949e-01	6.539822e-01
0.9949051	0.1506816619	2.011735e-01	0.8526631	0.0086445420	9.420657e-01	9.835175e-01
0.8194410	0.0742414693	9.095069e-01	0.9969722	0.7645972534	8.553050e-02	3.339128e-01
0.9949051	-0.0267283080	8.730997e-01	0.9969722	0.0749215406	6.789103e-01	8.656656e-01
0.9210048	0.0759562102	3.937233e-01	0.9430561	-0.0127924331	8.485392e-01	9.481630e-01
0.9949051	0.2872726473	3.181008e-01	0.9300968	0.1186093871	6.226762e-01	8.433108e-01
0.9949051	0.3917773927	6.500972e-01	0.9689755	4.7028180359	4.059347e-06	5.997686e-04
0.9949051	0.0148084249	9.170917e-01	0.9969722	0.4496829456	1.025211e-03	1.289148e-02
0.9949051	-0.0944635516	2.178242e-01	0.8800012	-0.0919603552	3.645740e-01	6.629639e-01
0.9275791	-0.0807889898	9.033177e-01	0.9969722	-0.6804748291	2.106736e-01	5.209544e-01
0.9949051	-0.0647063933	4.352678e-01	0.9482489	0.0696641950	4.681528e-01	7.506714e-01
0.8532963	0.2423239386	1.540454e-01	0.8386867	-0.1228722746	5.397383e-01	7.905460e-01
0.9148164	0.0079177183	9.812467e-01	0.9969722	-0.6471160059	4.591208e-02	2.354314e-01
0.9949051	-0.0778364125	3.953800e-01	0.9430561	-0.2392969852	2.046769e-02	1.398428e-01
0.9917395	0.6632836404	3.322018e-01	0.9366787	-0.0818194540	9.102837e-01	9.730907e-01
0.9949051	-0.0501056048	5.602578e-01	0.9641086	-0.1261103805	3.435296e-01	6.539822e-01
0.8805172	-0.1750615016	2.223890e-01	0.8820931	-0.2145235315	4.920778e-02	2.440266e-01
0.9236575	0.3741443457	2.372515e-01	0.8975264	1.1901658145	8.715823e-05	2.395838e-03
0.9949051	-0.1684331825	9.546935e-02	0.7921508	-0.1835518543	8.429551e-02	3.330413e-01
0.9949051	-0.0031697977	9.731853e-01	0.9969722	-0.1008659549	1.800628e-01	4.826173e-01
0.9949051	-0.4782560131	3.556812e-02	0.6690430	-0.3840053840	9.651445e-02	3.587424e-01
0.7815947	-0.0128639955	9.087604e-01	0.9969722	-0.0705190932	5.886595e-01	8.264179e-01
0.9949051	0.1667351086	7.250270e-01	0.9733364	0.8731615351	4.551546e-02	2.354314e-01
0.9949051	0.0742201927	5.915756e-01	0.9641086	0.1131093041	4.230837e-01	7.103479e-01
0.7817234	-0.0170816934	8.435973e-01	0.9969722	0.0696793180	3.815203e-01	6.715871e-01
0.9949051	-0.1884472391	1.976978e-01	0.8526631	0.0584089193	7.422474e-01	8.836608e-01
0.9949051	-0.1149952952	1.256216e-01	0.8166268	-0.0778407475	4.402802e-01	7.248067e-01
0.9949051	0.2435543973	2.287535e-01	0.8836164	-0.0044581742	9.867366e-01	9.950155e-01
0.9148164	-0.0848026556	4.945805e-01	0.9641086	-0.0099411493	9.381854e-01	9.835175e-01
0.9949051	1.0736273851	4.228699e-01	0.9430561	-1.0491686870	1.035384e-01	3.707823e-01
0.9509918	-0.2793487901	2.600029e-01	0.9038923	-0.4377422416	1.207234e-01	3.995752e-01
0.2034892	0.0144460219	9.343452e-01	0.9969722	-0.0760335571	5.869021e-01	8.264179e-01
0.9949051	0.7544841553	3.051428e-01	0.9286466	-0.7000380888	3.775790e-02	2.110347e-01
0.9949051	0.2065020358	7.146374e-01	0.9733364	2.5373892185	8.822860e-06	7.408616e-04
0.9949051	0.0627835282	5.022238e-01	0.9641086	0.2398748893	4.277405e-02	2.277429e-01
0.9210048	-0.0188060232	8.522551e-01	0.9969722	-0.0972004999	4.222778e-01	7.100034e-01
0.8264615	-0.0911846796	3.263597e-01	0.9364851	-0.0507317713	6.490270e-01	8.523888e-01
0.9970846	-0.0529783917	6.508751e-01	0.9689755	-0.1136720391	3.960782e-01	6.839053e-01
0.9949051	-0.0960152818	1.932770e-01	0.8526631	-0.0646608652	5.030279e-01	7.681899e-01
0.9949051	0.0463003130	9.120421e-01	0.9969722	1.9141980207	2.137750e-04	4.433815e-03
0.9949051	-0.0642483125	5.654767e-01	0.9641086	-0.1763568375	8.131330e-02	3.294921e-01
0.9949051	-0.2567652544	6.315678e-01	0.9689755	-0.7045189716	2.025066e-01	5.098358e-01
0.8805172	-0.1986739823	4.017127e-01	0.9430561	-0.2104073602	3.322887e-01	6.431217e-01
0.9949051	0.5860733832	3.511270e-01	0.9430561	2.3686724189	6.420197e-04	9.034135e-03
0.9127370	-0.1531794437	7.286704e-01	0.9733364	0.4988441881	3.215195e-01	6.355119e-01
0.8394240	0.1159452922	8.193617e-01	0.9957784	0.3996156029	4.714473e-01	7.506714e-01
0.7251512	-0.1663874172	1.873657e-01	0.8526631	-0.0411894862	7.417187e-01	8.836608e-01
0.9210048	0.1010307606	7.408157e-01	0.9772814	-0.2350841720	1.856727e-01	4.889364e-01
0.8194410	0.0225796169	8.958809e-01	0.9969722	0.2503395853	1.088786e-01	3.796298e-01
0.9210048	0.2794484583	3.532106e-01	0.9430561	-0.2844202761	1.227122e-01	3.995752e-01
0.9653626	-0.0816327930	2.978762e-01	0.9286466	-0.2031772448	8.291252e-02	3.328401e-01

0.9949051	0.0144135616	9.524368e-01	0.9969722	-0.3610732490	6.284609e-02	2.813791e-01
0.9109219	-0.0887693908	5.146995e-01	0.9641086	-0.0777129680	4.988731e-01	7.643323e-01
0.9949051	-0.1364580403	1.089319e-01	0.8149209	0.0300423046	6.953956e-01	8.762099e-01
0.9949051	0.1724748702	5.316625e-01	0.9641086	1.2935442547	4.701120e-06	6.174137e-04
0.9949051	-0.1751565860	4.622562e-02	0.7588705	-0.1645868981	9.547591e-02	3.560017e-01
0.9949051	-0.2086908563	7.455331e-02	0.7921508	0.0057591580	9.641062e-01	9.911619e-01
0.9949051	-0.1016909166	2.017850e-01	0.8526631	-0.1086603143	2.231280e-01	5.328026e-01
0.9949051	-0.2163575267	1.934453e-02	0.5081163	-0.1297272740	1.652860e-01	4.666833e-01
0.9696873	0.0325351869	9.343879e-01	0.9969722	-0.0884384191	6.614421e-01	8.563248e-01
0.9949051	-0.0466910636	5.044661e-01	0.9641086	-0.1383071179	9.723395e-02	3.602838e-01
0.8805172	-0.2978086497	4.489233e-02	0.7473625	0.0596010912	7.355384e-01	8.795259e-01
0.9210048	0.2153757908	3.264229e-01	0.9364851	0.1405865446	4.948598e-01	7.643323e-01
0.9949051	-0.0280514647	8.525486e-01	0.9969722	-0.0018982120	9.915415e-01	9.950155e-01
0.9949051	0.3866959128	4.168585e-01	0.9430561	-0.1340641636	7.024294e-01	8.762099e-01
0.8532963	-0.1120526165	6.928943e-01	0.9733364	-0.0898947279	7.270473e-01	8.773616e-01
0.9949051	0.2164069107	7.712660e-01	0.9919874	3.0233410443	2.179950e-04	4.433815e-03
0.9949051	-0.0844116522	6.607346e-01	0.9689755	-0.2759514164	5.240593e-02	2.559662e-01
0.9379096	-0.0393871367	6.722095e-01	0.9717521	-0.1769933325	2.176688e-01	5.283051e-01
0.9949051	0.4089024205	2.570717e-01	0.9016579	1.4544447363	1.026492e-04	2.637637e-03
0.9949051	0.2845042046	6.130141e-01	0.9641086	2.7955520605	6.381597e-07	2.496980e-04
0.8194410	0.1811644792	2.436883e-01	0.8975264	-0.0869670416	5.889497e-01	8.264179e-01
0.9949051	-0.0895340532	3.087213e-01	0.9288274	-0.0410742919	6.574407e-01	8.558314e-01
0.8805172	-0.2260298859	3.522674e-03	0.2875687	0.0410233980	5.654652e-01	8.104628e-01
0.8730639	-0.0564064952	4.982727e-01	0.9641086	-0.0626079471	4.122317e-01	6.984455e-01
0.9949051	-0.2347871214	3.147329e-03	0.2875687	-0.0516550604	5.840317e-01	8.247616e-01
0.9236575	-0.0760920978	4.366197e-01	0.9482489	-0.0984801278	2.222034e-01	5.328026e-01
0.2019302	-0.0255518111	8.222332e-01	0.9957784	-0.0715012165	5.801368e-01	8.225914e-01
0.9275791	-0.0705671860	6.061327e-01	0.9641086	-0.1783873345	1.477051e-01	4.368450e-01
0.9867694	0.2207519695	5.123420e-01	0.9641086	-0.5201204403	8.167613e-02	3.294921e-01
0.9275791	-0.0617285799	5.388246e-01	0.9641086	-0.1183170826	1.059039e-01	3.751228e-01
0.9696873	0.1166453156	4.439305e-01	0.9499322	-0.2450446647	6.093420e-02	2.794946e-01
0.9949051	0.0157478242	9.000427e-01	0.9969722	-0.1009232046	5.267373e-01	7.878622e-01
0.2019302	0.6743808587	3.056206e-01	0.9286466	-0.6325752388	3.640366e-01	6.629639e-01
0.9949051	0.1999378573	7.964958e-01	0.9957784	3.8765904393	4.091761e-04	6.625290e-03
0.9949051	0.0204010151	8.434096e-01	0.9969722	0.1918503280	5.007073e-02	2.465984e-01
0.9949051	-0.0017653123	9.811420e-01	0.9969722	-0.0298330933	6.089178e-01	8.375303e-01
0.9981675	-0.2011578550	5.762701e-02	0.7921508	-0.0493977340	6.112028e-01	8.384287e-01
0.9949051	-0.0986047022	3.155073e-01	0.9300968	-0.0838854350	4.671395e-01	7.502159e-01
0.8477725	-0.2733692093	6.221910e-02	0.7921508	0.0100626365	9.410675e-01	9.835175e-01
0.8899481	-0.1006782979	1.017165e-01	0.8015257	-0.0609484051	1.905291e-01	4.925112e-01
0.9949051	-0.0148360547	9.446832e-01	0.9969722	0.9972488498	8.153854e-06	7.408616e-04
0.9949051	-0.0944667105	2.387611e-01	0.8975264	-0.1136329338	1.478325e-01	4.368450e-01
0.9949051	-0.1091961627	1.331353e-01	0.8166268	-0.0719243867	4.552412e-01	7.382965e-01
0.9949051	0.3682792362	4.222279e-01	0.9430561	2.0310733675	1.203174e-05	8.413298e-04
0.7475613	0.0113657181	9.254052e-01	0.9969722	0.0587354656	5.984876e-01	8.312719e-01
0.7817234	-0.1208527288	2.704205e-01	0.9106469	-0.0184163626	8.504966e-01	9.481630e-01
0.9949051	-0.0705121579	4.947074e-01	0.9641086	-0.1216548061	3.195261e-01	6.336910e-01
0.9949051	-0.1778048096	5.006845e-01	0.9641086	-0.1359852238	6.652934e-01	8.575538e-01
0.9949051	0.0189309066	8.508635e-01	0.9969722	-0.0227759038	7.965977e-01	9.183880e-01
0.9210048	0.1063529867	5.445699e-01	0.9641086	-0.1979724747	1.238154e-01	4.002115e-01
0.9949051	-0.0709246808	4.492904e-01	0.9517226	-0.1471513415	1.815875e-01	4.845067e-01
0.9949051	-0.2237642111	6.748337e-02	0.7921508	-0.1295661368	2.230777e-01	5.328026e-01
0.9476053	-0.0558436784	5.216052e-01	0.9641086	-0.1234981080	2.500434e-01	5.599450e-01
0.9949051	-0.3357429151	9.964986e-03	0.3705021	0.0409206179	8.117054e-01	9.252033e-01
0.9949051	-0.2169957171	4.589469e-03	0.2875687	-0.0797812481	3.586447e-01	6.629639e-01
0.9949051	0.1586509591	4.883374e-01	0.9641086	-0.2193919947	2.227910e-01	5.328026e-01
0.9949051	-0.0943402434	3.706266e-01	0.9430561	-0.2408688117	1.059992e-01	3.751228e-01
0.9210048	0.0601675231	8.054935e-01	0.9957784	-0.0490276706	8.249654e-01	9.358053e-01
0.5937207	-0.0267780232	7.312375e-01	0.9733364	0.0455606570	5.435675e-01	7.934534e-01
0.8805172	0.0718399624	7.383261e-01	0.9767472	0.2917881893	5.451354e-02	2.619309e-01
0.9949051	0.3434590937	6.845777e-01	0.9733364	-0.1184556210	8.867404e-01	9.645345e-01
0.9949051	0.2981108484	1.757511e-01	0.8526631	0.0439654579	8.571982e-01	9.513693e-01
0.9949051	0.0571130833	5.937127e-01	0.9641086	-0.2305578399	1.516597e-02	1.106554e-01
0.8477725	0.0003624208	9.997294e-01	0.9997294	-1.5685226570	1.485215e-01	4.377867e-01
0.9949051	-0.1244714862	1.145231e-01	0.8166268	-0.0772929650	3.716776e-01	6.676641e-01
0.8805172	-0.0451309603	5.997952e-01	0.9641086	0.0950453122	1.072775e-01	3.780833e-01
0.9949051	0.1968325632	7.392237e-01	0.9767472	2.6764209288	3.327715e-05	1.456800e-03
0.8540650	-0.1929411512	4.002648e-01	0.9430561	-0.0286115864	8.642564e-01	9.515996e-01
0.9949051	-0.1533141775	3.620111e-01	0.9430561	-0.1625281731	4.076240e-01	6.952547e-01
0.8540650	-0.0236084998	9.202090e-01	0.9969722	-0.2738428406	3.276655e-01	6.412262e-01
0.9949051	-0.0073602844	9.940101e-01	0.9997294	-0.7747341159	4.411200e-01	7.251792e-01
0.9949051	-0.1668883409	5.837867e-02	0.7921508	-0.1782098032	8.696376e-02	3.348246e-01
0.9949051	0.4054373634	4.452305e-01	0.9499322	-0.3191198355	1.499351e-01	4.408538e-01
0.9148164	-0.2036013867	3.330814e-01	0.9366787	0.0284593001	9.064726e-01	9.713967e-01
0.9379096	-0.1083139086	4.638374e-01	0.9641086	-0.4438654850	2.457239e-03	2.547769e-02
0.9210048	-0.0448124045	6.275802e-01	0.9689755	0.1386488816	1.014059e-01	3.676741e-01
0.8532963	0.3604779211	1.309954e-01	0.8166268	-0.0751948147	6.986158e-01	8.762099e-01
0.8612982	-0.1656169283	1.391589e-01	0.8166268	-0.1460403193	2.128357e-01	5.215962e-01
0.9949051	0.0789107277	6.716015e-01	0.9717521	-0.1261600611	4.935534e-01	7.643323e-01
0.8194410	0.3007561292	6.866954e-02	0.7921508	-0.0565504162	7.118932e-01	8.762099e-01
0.9949051	0.0744447085	1.719302e-01	0.8467562	-0.1699097656	5.401131e-02	2.612240e-01
0.9210048	-0.1563707747	7.407727e-02	0.7921508	-0.0316491780	7.648254e-01	8.933039e-01
0.9923046	0.0452635251	6.800351e-01	0.9733364	-0.0640168612	4.981990e-01	7.643323e-01
0.9949051	-0.1238358378	1.066386e-01	0.8123872	-0.1701574769	6.225148e-02	2.813791e-01
0.8329325	0.8571783218	1.286548e-01	0.8166268	0.0304214740	9.248589e-01	9.835175e-01
0.8477725	-0.6176779904	8.357461e-02	0.7921508	-0.3874698223	2.364801e-01	5.464911e-01
0.9949051	0.2237967017	5.713789e-01	0.9641086	1.4565502039	1.721581e-03	1.901784e-02
0.9949051	-0.1020816101	1.468795e-01	0.8189227	-0.0585886889	5.931743e-01	8.274987e-01
0.9949051	-0.1622164090	1.173612e-01	0.8166268	-0.1483130077	1.506094e-01	4.410394e-01
0.8532963	-0.2742910424	2.027059e-01	0.8526631	0.0619726787	8.006495e-01	9.207928e-01
0.9923046	0.0310885210	6.476351e-01	0.9689755	-0.2050392293	2.652742e-03	2.703053e-02
0.8394240	1.3464559766	4.012210e-01	0.9430561	-1.3023985047	1.218945e-01	3.995752e-01
0.9923046	-0.2577015455	5.272464e-01	0.9641086	0.2098195898	6.643433e-01	8.572639e-01
0.9210048	-0.3686881490	6.709734e-02	0.7921508	-0.2972935100	1.542167e-01	4.468082e-01
0.9949051	-0.1826327670	3.257746e-02	0.6526535	-0.0900467211	4.307670e-01	7.171360e-01
0.7247177	-0.0613527091	5.881922e-01	0.9641086	-0.1917956767	1.197154e-01	3.995752e-01
0.9949051	-0.2334211239	4.190045e-03	0.2875687	-0.0259829780	7.592528e-01	8.905775e-01
0.9949051	0.0338604030	7.841218e-01	0.9955231	0.0613099574	5.068879e-01	7.730858e-01
0.9867694	-0.0007351032	9.950232e-01	0.9997294	-0.2717755828	9.704261e-03	7.855811e-02
0.9949051	-0.1781174781	8.404338e-02	0.7921508	-0.2028780462	1.154007e-01	3.942300e-01
0.9109219	0.2565067392	3.052217e-01	0.9286466	0.3088321741	3.691120e-01	6.675072e-01
0.8497626	-0.1749044560	1.356522e-01	0.8166268	-0.0090423384	9.302637e-01	9.835175e-01
0.8741974	-0.0302281845	8.437044e-01	0.9969722	-0.0460964803	7.175108e-01	8.762099e-01
0.9949051	-0.0279395126	7.515578e-01	0.9826784	0.0452364384	7.196428e-01	8.762099e-01
0.9949051	0.3628003308	3.581543e-01	0.9430561	-0.1194257230	5.907584e-01	8.264179e-01
0.9949051	-0.3952912031	4.029832e-01	0.9430561	-0.1218905665	8.375686e-01	9.420896e-01
0.9949051	-0.0706767037	4.673629e-01	0.9641086	-0.0978827906	2.689227e-01	5.832415e-01
0.9949051	0.0005780718	9.992325e-01	0.9997294	-0.6575809015	1.197645e-01	3.995752e-01
0.9949051	0.0143544871	8.861906e-01	0.9969722	-0.1556523241	9.443362e-02	3.558867e-01
0.9949051	0.1015102415	4.708503e-01	0.9641086	0.0626763162	6.480450e-01	8.523113e-01
0.7342939	0.6060075916	7.002305e-01	0.9733364	-0.1439567331	9.087851e-01	9.729928e-01
0.9949051	-0.0255301564	7.252489e-01	0.9733364	0.0032343704	9.608524e-01	9.906899e-01
0.9949051	-0.0246981088	7.386623e-01	0.9767472	-0.0382801102	6.114429e-01	8.384287e-01
0.9949051	-0.0001984782	9.991121e-01	0.9997294	0.1601796813	4.071233e-01	6.952547e-01
0.9949051	-0.3155460304	8.327952e-02	0.7921508	-0.0545970154	7.302717e-01	8.778587e-01
0.9949051	-0.0720723291	2.986653e-01	0.9286466	-0.1025682116	1.654317e-01	4.666833e-01
0.8329325	-0.0169954400	8.594879e-01	0.9969722	0.1257842277	1.859660e-01	4.889364e-01
0.6918369	-0.1383090980	4.110386e-01	0.9430561	0.1229974739	3.899350e-01	6.774824e-01
0.9949051	0.1077515134	7.105627e-01	0.9733364	1.0658961730	3.529626e-06	5.960026e-04
0.8805172	0.0172157893	8.364844e-01	0.9969722	-0.0001814861	9.977720e-01	9.977720e-01
0.8264615	-0.1031314148	5.999459e-01	0.9641086	0.0139528005	9.329959e-01	9.835175e-01
0.6922402	-0.3021183283	9.394943e-02	0.7921508	-0.0797920152	5.804190e-01	8.225914e-01
0.8878861	0.0458613711	6.961040e-01	0.9733364	-0.1469942531	4.489742e-02	2.348175e-01
0.9949051	-0.8418128096	1.917984e-01	0.8526631	-0.6923446209	2.478209e-01	5.587016e-01
0.9949051	-0.0339779427	7.226045e-01	0.9733364	-0.0160802679	8.778846e-01	9.590199e-01
0.9949051	-0.0753910674	6.130835e-01	0.9641086	-0.1642937483	2.553026e-01	5.661682e-01
0.8497626	-0.0147807247	9.056744e-01	0.9969722	-0.0204124080	8.325936e-01	9.402361e-01
0.9949051	-0.1812735068	1.676055e-01	0.8467562	0.0953403772	5.407285e-01	7.910162e-01
0.2019302	0.6452404812	7.202540e-02	0.7921508	-0.0232595594	9.311631e-01	9.835175e-01
0.9949051	-0.1763672103	8.905595e-02	0.7921508	-0.0526631259	6.709703e-01	8.607204e-01
0.9148164	-0.0426490312	6.965165e-01	0.9733364	-0.0922267028	4.252080e-01	7.129020e-01
0.9949051	0.0326758876	8.998022e-01	0.9969722	0.1480786272	5.289384e-01	7.882615e-01
0.8194410	0.5725958925	5.885161e-01	0.9641086	-6.7354305177	3.258402e-03	3.183001e-02
0.7397893	-0.0375659628	5.982473e-01	0.9641086	-0.0151775390	8.328488e-01	9.402361e-01
0.9949051	-0.0728958451	4.119188e-01	0.9430561	-0.1085024033	3.388517e-01	6.512563e-01
0.8329325	0.6032863290	4.759595e-01	0.9641086	-0.7250724982	9.944992e-02	3.628080e-01
0.8612982	-0.1255156919	1.698394e-01	0.8467562	-0.1417717127	4.038192e-02	2.209789e-01
0.7815947	-0.1001710750	6.140396e-01	0.9641086	-0.1774890885	4.456493e-01	7.295810e-01
0.9949051	-0.2593095532	6.190850e-01	0.9641086	2.1222156378	8.676218e-03	7.222035e-02

0.9949051	-0.0353916714	6.942172e-01	0.9733364	-0.0688632858	4.742480e-01	7.506714e-01
0.8394240	-0.0049236219	9.506901e-01	0.9969722	0.0613471199	2.880165e-01	6.045220e-01
0.9949051	-0.2014870786	5.080365e-01	0.9641086	0.7764991646	3.416068e-03	3.302506e-02
0.9949051	0.1682745563	4.097692e-01	0.9430561	0.7800312558	4.391094e-05	1.674282e-03
0.9653626	-0.1530073089	1.580349e-01	0.8386867	-0.0953910029	3.638522e-01	6.629639e-01
0.5133973	-0.0417017240	8.237046e-01	0.9964090	-0.0269606235	8.791565e-01	9.595226e-01
0.8497626	0.0354809694	8.576851e-01	0.9969722	-0.2112662801	1.721314e-01	4.776041e-01
0.9379096	-0.0274546881	7.809501e-01	0.9932650	-0.0792120620	3.458028e-01	6.539822e-01
0.9210048	0.0273264335	6.738950e-01	0.9717521	-0.0197533435	7.308062e-01	8.778587e-01
0.6918844	0.0301702648	8.266019e-01	0.9964090	0.0460504383	7.517455e-01	8.876755e-01
0.9949051	-0.1669418303	1.540478e-01	0.8386867	0.1549166171	3.113848e-01	6.238252e-01
0.9981675	0.0087388307	9.414816e-01	0.9969722	-0.1483367743	2.077621e-01	5.169997e-01
0.8497626	-0.0150909373	9.583586e-01	0.9969722	-0.0885041597	7.797263e-01	9.053594e-01
0.8194410	-0.0914664764	4.802315e-01	0.9641086	-0.2030710888	4.750334e-02	2.391031e-01
0.9379096	-0.2095332181	8.067931e-02	0.7921508	-0.0557846523	6.246323e-01	8.433108e-01
0.9379096	0.2851131564	2.391883e-01	0.8975264	-0.0086059998	9.679419e-01	9.914275e-01
0.8103326	-0.0641040458	4.576020e-01	0.9607204	-0.0533420222	4.961010e-01	7.643323e-01
0.9949051	-0.1079865556	4.427724e-01	0.9499322	-0.1461901047	1.758186e-01	4.802300e-01
0.9949051	0.0087076212	9.665244e-01	0.9969722	-0.1384441513	5.154455e-01	7.810982e-01
0.8805172	-0.0494990804	6.611232e-01	0.9689755	-0.0081861340	9.483806e-01	9.835175e-01
0.9949051	-0.1002806224	7.541191e-01	0.9839586	1.2335778535	4.613069e-04	7.131524e-03
0.9149215	-0.2302005974	1.172877e-01	0.8166268	-0.0214876898	8.620565e-01	9.515996e-01
0.9949051	0.2163629589	2.461904e-01	0.8975264	-0.3390405790	1.460729e-01	4.367095e-01
0.9949051	-0.1116667395	5.149556e-01	0.9641086	-0.2608103867	9.388297e-02	3.558867e-01
0.9949051	-0.0823359998	3.031149e-01	0.9286466	-0.1724639173	3.493670e-02	2.004620e-01
0.7194638	-0.1783746338	6.051857e-02	0.7921508	-0.2600407726	2.585610e-02	1.660974e-01
0.9148164	-0.0734787018	3.150255e-01	0.9300968	-0.0372478119	4.897597e-01	7.621198e-01
0.9949051	0.0185259343	7.940539e-01	0.9957784	-0.0334853481	6.441280e-01	8.512682e-01
0.8497626	-0.2135487677	5.233313e-02	0.7921508	-0.1524453229	1.241271e-01	4.002115e-01
0.9949051	-0.1682452635	7.455131e-02	0.7921508	-0.1442052241	1.187748e-01	3.995752e-01
0.9949051	-0.3024257983	4.093273e-01	0.9430561	-0.3954591274	2.751535e-01	5.881219e-01
0.9949051	-0.0828755315	6.306213e-01	0.9689755	-0.2206174420	2.394322e-01	5.481636e-01
0.9148164	-0.1876695968	2.503239e-01	0.8975264	0.2440387608	1.596492e-01	4.560401e-01
0.9949051	-0.0946407365	3.510529e-01	0.9430561	-0.1121876032	2.614982e-01	5.723906e-01
0.9148164	-0.0536439030	6.753515e-01	0.9717521	-0.2766164181	3.809146e-02	2.110347e-01
0.9949051	0.6842950356	1.312026e-01	0.8166268	-0.4882174860	1.882522e-01	4.902249e-01
0.2506875	-0.0942090440	5.758955e-01	0.9641086	-0.1545411955	3.312345e-01	6.431217e-01
0.9949051	-0.1290573592	2.281179e-01	0.8836164	-0.1104532261	3.712977e-01	6.676641e-01
0.2019302	-0.0628041645	5.264716e-01	0.9641086	-0.1439491697	9.885870e-02	3.627027e-01
0.4126320	-0.1220471993	1.833969e-01	0.8526631	0.0053626105	9.729170e-01	9.922242e-01
0.8532963	-0.0646167307	6.081490e-01	0.9641086	-0.0749349120	5.653213e-01	8.104628e-01
0.9949051	0.0858464218	8.002326e-01	0.9957784	-0.1185369950	5.714756e-01	8.131842e-01
0.9653626	0.0652496906	5.511484e-01	0.9641086	-0.1468948084	9.911418e-02	3.627027e-01
0.9949051	-0.0686714480	4.316740e-01	0.9482489	-0.0924553927	2.351052e-01	5.459614e-01
0.8532963	0.0161573126	8.884295e-01	0.9969722	-0.1865600159	2.781744e-01	5.897332e-01
0.8532963	0.0524258783	3.783228e-01	0.9430561	-0.0924792137	1.756489e-01	4.802300e-01
0.9923046	0.0038689442	9.762982e-01	0.9969722	-0.1555986151	1.851808e-01	4.889364e-01
0.8540650	-0.0082747659	9.412388e-01	0.9969722	-0.1771812616	6.576071e-03	5.757716e-02
0.9949051	-0.1402120290	8.721382e-02	0.7921508	-0.0461486536	5.584098e-01	8.039469e-01
0.3047548	-0.0236867108	9.074279e-01	0.9969722	0.4517519963	3.063753e-02	1.847083e-01
0.5092568	0.1309685696	5.161019e-01	0.9641086	0.0730172295	6.927215e-01	8.738493e-01
0.9949051	-0.1186574528	6.076315e-01	0.9641086	0.7318220384	2.980944e-03	2.960904e-02
0.9949051	0.0557120825	5.912088e-01	0.9641086	0.3224180996	6.120541e-04	8.822535e-03
0.9949051	0.0668839837	7.986824e-01	0.9957784	1.1359353520	2.334637e-04	4.599235e-03
0.9949051	-0.0521450214	5.430900e-01	0.9641086	-0.1738388159	1.790327e-01	4.809469e-01
0.9949051	-0.3708216727	5.019002e-02	0.7909947	-0.0908515016	6.565634e-01	8.556317e-01
0.9135088	-0.2651360226	1.214241e-02	0.3879007	-0.1365844887	2.547283e-01	5.659565e-01
0.9694325	-0.1429240452	9.512019e-03	0.3705021	-0.0485525585	3.384343e-01	6.512563e-01
0.6925121	0.1826963370	4.773889e-01	0.9641086	0.7941963756	4.962985e-03	4.547479e-02
0.9949051	0.1007161718	3.627482e-01	0.9430561	-0.1321046527	1.475911e-01	4.368450e-01
0.9275791	-0.0220089872	8.192254e-01	0.9957784	-0.0988137231	2.988628e-01	6.175802e-01
0.9148164	-0.0508678830	5.611678e-01	0.9641086	-0.1178699197	1.897722e-01	4.922230e-01
0.9949051	-0.1586612565	1.434487e-01	0.8166268	-0.1205331345	1.755466e-01	4.802300e-01
0.9516289	-0.1183184578	1.438143e-01	0.8166268	0.0387687306	5.255993e-01	7.873997e-01
0.9981675	0.0400363724	8.396140e-01	0.9969722	0.8140956687	3.089432e-05	1.451602e-03
0.9949051	-0.1512152591	6.547069e-01	0.9689755	1.5772343886	6.798151e-04	9.243899e-03
0.9949051	0.0050339737	9.777208e-01	0.9969722	0.9098074911	2.046755e-04	4.398662e-03
0.9109219	-0.4884787666	3.651312e-01	0.9430561	-0.3729313304	4.730411e-01	7.506714e-01
0.9949051	0.0658113570	7.992037e-01	0.9957784	-0.3598743258	2.086503e-02	1.409284e-01
0.9692638	1.0660831356	2.982487e-01	0.9286466	-0.3653081503	3.738518e-01	6.695347e-01
0.9949051	0.0240749939	9.146403e-01	0.9969722	0.1249591096	5.865598e-01	8.264179e-01
0.9949051	-0.0529145944	4.353356e-01	0.9482489	-0.0496432076	5.444084e-01	7.934534e-01
0.9949051	-0.1164188653	3.560829e-01	0.9430561	0.1232088431	4.382222e-01	7.234338e-01
0.9949051	-0.1363794126	1.508736e-01	0.8333300	0.3883765498	9.963521e-03	7.957353e-02
0.9379096	-0.0818015471	2.598188e-01	0.9038923	-0.1576553210	1.569605e-02	1.131264e-01
0.5172057	-0.2232219826	2.038184e-02	0.5125815	-0.2404537372	6.124288e-02	2.794946e-01
0.7815947	0.0685126778	5.850807e-01	0.9641086	-0.2244758659	1.270081e-01	4.046458e-01
0.9949051	0.0763171156	3.775705e-01	0.9430561	-0.2421121883	3.625164e-02	2.050213e-01
0.9949051	-0.2030106843	1.070917e-02	0.3723011	-0.1613678198	8.306924e-02	3.328401e-01
0.9949051	-0.1154633334	2.714804e-01	0.9110414	0.2453846123	6.612407e-02	2.881189e-01
0.9379096	0.1063258508	3.720837e-01	0.9430561	-0.0936999826	3.314455e-01	6.431217e-01
0.9949051	0.0950039547	1.586241e-01	0.8386867	-0.1890362083	3.108778e-03	3.062147e-02
0.9949051	0.3358673658	6.583880e-01	0.9689755	3.8047008914	6.209903e-06	6.672823e-04
0.9949051	0.1523069416	5.801461e-01	0.9641086	0.6155465581	4.478689e-02	2.348175e-01
0.9949051	-0.1714226144	5.458008e-02	0.7921508	-0.0853214559	4.056432e-01	6.952547e-01
0.9210048	0.1066003489	5.012477e-01	0.9641086	0.6679694842	9.226262e-04	1.198400e-02
0.9949051	0.1015228845	4.015292e-01	0.9430561	-0.0393162057	7.015193e-01	8.762099e-01
0.7251512	0.0105029094	8.747852e-01	0.9969722	-0.0586791685	4.955003e-01	7.643323e-01
0.9949051	-0.1323080867	9.430683e-02	0.7921508	0.0288553991	6.053875e-01	8.359439e-01
0.9949051	0.2097993208	4.531878e-01	0.9579510	-0.0196152047	9.479147e-01	9.835175e-01
0.8540650	-0.0743333443	3.329795e-01	0.9366787	-0.1279295917	8.345924e-02	3.328497e-01
0.9949051	-0.0904305134	2.424821e-01	0.8975264	-0.0737643392	2.222890e-01	5.328026e-01
0.9949051	0.0471525459	8.703365e-01	0.9969722	1.4573242299	6.665362e-05	2.058028e-03
0.9949051	-0.2979265865	3.227821e-03	0.2875687	-0.2237307116	6.394452e-02	2.838570e-01
0.9949051	-0.2247050710	5.747047e-01	0.9641086	2.0755942845	4.840756e-03	4.470136e-02
0.9949051	0.4523059171	5.162952e-01	0.9641086	2.7025323304	6.898401e-04	9.265807e-03
0.9949051	-0.1352644510	1.576401e-01	0.8386867	-0.0989485109	2.999647e-01	6.187753e-01
0.8598556	-0.0444431657	6.547765e-01	0.9689755	0.0199555894	8.192996e-01	9.302711e-01
0.9949051	-0.0026337893	9.783302e-01	0.9969722	-0.2245521383	2.306685e-02	1.506355e-01
0.9696873	-0.0694784957	4.298352e-01	0.9478644	0.0412736500	7.266548e-01	8.773616e-01
0.7475613	-0.0272992229	8.693764e-01	0.9969722	-0.2433352080	1.470820e-01	4.368450e-01
0.8194410	-0.0395849059	7.303726e-01	0.9733364	-0.0083889356	9.457852e-01	9.835175e-01
0.8394240	0.2834851066	5.879702e-01	0.9641086	2.2409703776	2.172294e-05	1.126414e-03
0.8477725	-0.0660643191	4.449915e-01	0.9499322	-0.2463527813	3.193197e-04	5.718726e-03
0.8329325	-0.0228711335	7.086020e-01	0.9733364	-0.0544236382	4.329273e-01	7.187080e-01
0.8477725	-0.2572937232	9.653674e-02	0.7921508	-0.1540620477	3.080777e-01	6.214128e-01
0.9949051	-0.0047165084	9.682718e-01	0.9969722	-0.1010806448	5.102947e-01	7.770557e-01
0.9949051	0.0414441189	7.143190e-01	0.9733364	-0.2057484397	5.269955e-02	2.563410e-01
0.9949051	0.1165266588	2.653876e-01	0.9092411	-0.1977599780	2.454795e-02	1.585556e-01
0.9949051	-0.1103624077	3.631612e-01	0.9430561	-0.2032110516	1.077953e-01	3.780833e-01
0.9949051	0.4945548300	3.670779e-01	0.9430561	2.3041899786	9.401796e-06	7.408616e-04
0.9949051	-0.1498404811	7.349220e-02	0.7921508	-0.1226820281	1.950238e-01	4.989570e-01
0.8088570	-0.0156878378	8.775920e-01	0.9969722	-0.0614440729	6.500714e-01	8.528129e-01
0.9275791	0.4934586103	4.300467e-01	0.9478644	0.6252328923	5.470511e-01	7.942706e-01
0.5092568	-0.1346209719	2.497983e-01	0.8975264	-0.1659103978	2.646665e-01	5.782547e-01
0.9949051	-0.0003300541	9.986978e-01	0.9997294	0.0265458353	9.053125e-01	9.710339e-01
0.5788469	0.3131003121	6.409044e-01	0.9689755	-0.6855542237	4.773971e-02	2.391031e-01
0.9949051	0.0101089515	9.017943e-01	0.9969722	-0.0437407264	6.560770e-01	8.556317e-01
0.9949051	0.1067023045	4.957750e-01	0.9641086	-0.2886313932	1.428870e-02	1.068940e-01
0.8394240	-0.0725671630	6.007687e-01	0.9641086	-0.2496824125	3.396883e-02	1.977890e-01
0.5133973	0.1395415749	3.515176e-01	0.9430561	-0.2755569772	6.779081e-02	2.924407e-01
0.9949051	0.2620363667	3.039477e-01	0.9286466	-0.3268434600	1.166325e-01	3.972899e-01
0.9923046	0.1970081087	4.356547e-01	0.9482489	-0.1854449880	3.781925e-01	6.707992e-01
0.8497626	1.1710612693	1.315257e-01	0.8166268	-0.1212034575	7.096529e-01	8.762099e-01
0.7655249	-0.2323625715	6.581365e-01	0.9689755	-1.1272440343	1.832006e-02	1.258972e-01
0.8878861	-0.3572077682	2.125441e-01	0.8723164	-0.0875652235	7.091948e-01	8.762099e-01
0.9949051	-0.1148032285	5.807870e-01	0.9641086	1.1100356923	2.497958e-04	4.762236e-03
0.9275791	-0.0772474942	5.863809e-01	0.9641086	-0.1446504604	2.884521e-01	6.045220e-01
0.9949051	0.3802410148	4.611318e-01	0.9641086	2.1720660134	1.935972e-06	3.813864e-04
0.8475093	-0.3805744438	4.197482e-01	0.9430561	0.4473895529	5.278623e-01	7.878622e-01
0.9949051	-0.0356284207	8.579878e-01	0.9969722	-0.2498339575	1.772535e-01	4.802300e-01
0.5092568	-0.0637822359	6.334634e-01	0.9689755	-0.0600256211	6.681392e-01	8.600198e-01
0.9949051	0.2174840517	7.543888e-01	0.9839586	2.8032263546	1.102836e-04	2.773515e-03
0.9949051	-0.1305305278	2.694772e-01	0.9106469	-0.2157428086	1.141282e-01	3.920865e-01
0.7251512	0.0775116662	6.718791e-01	0.9717521	-0.1275240128	5.176526e-01	7.834384e-01
0.9949051	-0.1448393608	1.856890e-01	0.8526631	-0.3058621699	4.787393e-03	4.455668e-02
0.9949051	-0.1029897485	3.103949e-01	0.9288274	-0.0965136637	4.131322e-01	6.986013e-01
0.8540650	-0.1737195462	8.340836e-01	0.9969722	1.7777357685	5.608363e-02	2.673018e-01
0.8899481	-0.0841508809	2.733505e-01	0.9110414	-0.1132355517	1.727300e-01	4.781426e-01

0.9949051	-0.1225289385	4.085784e-01	0.9430561	-0.0547518048	7.174900e-01	8.762099e-01
0.9949051	-0.0142041025	9.302218e-01	0.9969722	-0.0876176026	5.238388e-01	7.867567e-01
0.8194410	0.0790354016	6.423345e-01	0.9689755	-0.0541360552	6.906595e-01	8.721790e-01
0.8194410	0.0177762668	8.664713e-01	0.9969722	-0.0313234953	7.215281e-01	8.762099e-01
0.8088570	-0.0713657036	4.963552e-01	0.9641086	-0.0692512631	5.686531e-01	8.127545e-01
0.9148164	-0.0634330526	6.708669e-01	0.9717521	-0.1063875589	4.816479e-01	7.570582e-01
0.9257199	0.0315331685	7.779451e-01	0.9932650	-0.2428074887	1.129757e-02	8.902485e-02
0.7815947	0.0513873455	6.345614e-01	0.9689755	-0.1567457971	2.027266e-01	5.098358e-01
0.9949051	-0.7375804405	7.523072e-02	0.7921508	2.0197890021	2.149205e-03	2.268179e-02
0.9949051	0.2170843036	5.491599e-01	0.9641086	0.1494612143	6.873801e-01	8.698964e-01
0.9949051	-0.0040345148	9.815230e-01	0.9969722	-0.1773579558	2.286415e-01	5.383552e-01
0.9949051	0.0785085252	8.028674e-01	0.9957784	-0.3338507544	6.015881e-02	2.791213e-01
0.9949051	-0.1756470408	2.811397e-01	0.9154467	-0.1734429023	2.373245e-01	5.468179e-01
0.9949051	-0.1340073464	1.286838e-01	0.8166268	0.0414532229	6.847359e-01	8.684097e-01
0.8532963	-0.1247439884	1.291658e-01	0.8166268	-0.2010774127	6.269007e-02	2.813791e-01
0.9949051	-0.1719728043	2.203726e-01	0.8800012	0.0604060401	6.212172e-01	8.433108e-01
0.9949051	-0.0780479729	6.158645e-01	0.9641086	-0.0985695952	5.531576e-01	7.993060e-01
0.9949051	-0.0506650070	8.692402e-01	0.9969722	0.3873407986	1.241728e-01	4.002115e-01
0.8805172	-0.2027987289	5.840679e-03	0.3216097	-0.0342407459	7.151523e-01	8.762099e-01
0.9949051	0.1323411476	8.194963e-01	0.9957784	2.6413533684	1.240218e-05	8.413298e-04
0.8805172	-0.0065842945	9.676899e-01	0.9969722	-0.0712696609	5.233321e-01	7.867567e-01
0.9949051	-0.0941007015	6.905361e-01	0.9733364	0.2467706187	3.067687e-01	6.214128e-01
0.9981675	-0.2914006624	4.061180e-02	0.6857593	-0.2612565670	6.419772e-02	2.838570e-01
0.7475613	0.0956162278	6.498295e-01	0.9689755	-0.0427277828	7.341275e-01	8.794980e-01
0.9949051	0.3203650771	4.764825e-01	0.9641086	2.3023789396	5.861794e-09	6.928640e-06
0.9949051	-0.0988387423	7.653690e-01	0.9876268	0.1991411266	5.599180e-01	8.051376e-01
0.9949051	-0.0643230987	5.730882e-01	0.9641086	0.0800926731	3.921747e-01	6.796928e-01
0.9148164	-0.1039436865	2.773454e-01	0.9110414	-0.0800773514	2.891260e-01	6.048618e-01
0.9949051	0.6871388996	3.184065e-01	0.9300968	-0.3805004477	2.613797e-01	5.723906e-01
0.9694325	-0.1213890267	3.255280e-02	0.6526535	-0.0065118801	9.375558e-01	9.835175e-01
0.8539066	0.3068646557	3.374639e-01	0.9366787	-0.1040348468	3.869655e-01	6.766173e-01
0.2019302	0.2940746979	1.786443e-01	0.8526631	-0.1489443924	4.880565e-01	7.621198e-01
0.9210048	-0.1118625958	5.639571e-01	0.9641086	-0.0720674290	6.292725e-01	8.433108e-01
0.9949051	0.0657647696	9.216326e-01	0.9969722	2.7483659395	2.102760e-03	2.239156e-02
0.9949051	-0.1775887151	2.642611e-02	0.5893521	-0.0927948567	3.710999e-01	6.676641e-01
0.6504978	-0.0230860076	8.941464e-01	0.9969722	-0.3204337737	2.222303e-02	1.467465e-01
0.6504978	0.0429182600	7.097572e-01	0.9733364	-0.1414453250	3.820763e-02	2.110347e-01
0.8539066	-0.1278429958	7.122564e-01	0.9733364	-0.5811145971	4.241613e-02	2.277429e-01
0.9923046	-0.1632314533	2.422365e-01	0.8975264	-0.2835327949	4.204137e-03	3.943881e-02
0.9149215	0.0839085528	5.214914e-01	0.9641086	-0.2836526471	1.692853e-02	1.191043e-01
0.9949051	-0.0864214403	4.977159e-01	0.9641086	-0.0516304583	6.007390e-01	8.314678e-01
0.5133973	-0.0180678485	8.843341e-01	0.9969722	0.0118827035	9.310031e-01	9.835175e-01
0.8194410	-0.2541184474	3.538291e-02	0.6690430	-0.0789326862	3.151753e-01	6.303507e-01
0.9917395	-0.0481555587	6.082107e-01	0.9641086	-0.0820356996	4.796463e-01	7.551927e-01
0.9923046	0.5558766523	2.772826e-01	0.9110414	-0.5593020668	4.487223e-02	2.348175e-01
0.9949051	-0.0659364398	7.261546e-01	0.9733364	-0.3143001252	1.077659e-01	3.780833e-01
0.8497626	0.0103366110	8.826035e-01	0.9969722	0.0111287358	8.555526e-01	9.513294e-01
0.9949051	-0.0858379119	7.395844e-01	0.9767472	-0.4922213289	3.165025e-02	1.879929e-01
0.9949051	-0.2310653489	1.401243e-01	0.8166268	-0.2916163479	9.842027e-02	3.627027e-01
0.9949051	3.2462895331	2.868865e-01	0.9286466	-2.4155230034	2.126030e-01	5.215962e-01
0.9949051	-0.1430057321	1.833138e-01	0.8526631	-0.1468707212	2.570040e-01	5.688741e-01
0.8394240	-0.0169049874	9.080433e-01	0.9969722	-0.1844933790	6.949266e-02	2.976099e-01
0.9949051	0.0680768110	6.742662e-01	0.9717521	-0.3967273666	2.674080e-02	1.699335e-01
0.7251512	0.0002033438	9.984458e-01	0.9997294	-0.0425612757	6.410753e-01	8.512682e-01
0.9949051	0.0081902289	9.872120e-01	0.9981904	2.4791612486	2.295697e-05	1.130631e-03
0.2019302	0.0247791792	9.771579e-01	0.9969722	0.4628534203	5.315116e-01	7.882615e-01
0.8741974	0.0388419335	7.503638e-01	0.9823061	-0.0124594966	9.048471e-01	9.710339e-01
0.8805172	-0.1159762191	1.629743e-01	0.8441531	0.1868342990	4.532936e-02	2.354314e-01
0.8497626	0.0097792136	9.277637e-01	0.9969722	-0.1970156347	1.822219e-01	4.851043e-01
0.8805172	0.4157918446	1.961499e-01	0.8526631	0.7328063274	6.640598e-05	2.058028e-03
0.9949051	0.0370296659	8.193978e-01	0.9957784	0.1778195959	3.771280e-01	6.707992e-01
0.9949051	-0.1047320027	5.902492e-01	0.9641086	-0.1393488593	5.108056e-01	7.770557e-01
0.9949051	0.0775269050	8.926331e-01	0.9969722	1.8420932388	3.465006e-04	6.112892e-03
0.5133973	0.0070597198	9.484321e-01	0.9969722	-0.0413190510	6.633760e-01	8.572639e-01
0.9949051	-0.4998431371	1.817754e-02	0.4996710	-0.1671248117	4.585689e-01	7.414889e-01
0.9949051	-0.1334160991	9.296381e-02	0.7921508	-0.1078690829	3.055929e-01	6.214128e-01
0.9949051	-0.1614665983	9.041872e-02	0.7921508	-0.0594816667	5.368447e-01	7.882615e-01
0.9949051	-0.1387972893	8.637255e-01	0.9969722	3.9711205482	2.075753e-03	2.230491e-02
0.9949051	0.1399743828	5.890982e-01	0.9641086	0.6552774311	2.382820e-02	1.547524e-01
0.9949051	-0.0203344963	8.292978e-01	0.9964090	-0.0369577735	6.365105e-01	8.493805e-01
0.9949051	-0.2088340003	7.650811e-01	0.9876268	-0.6961598659	3.065022e-01	6.214128e-01
0.9210048	-0.2498437447	2.853844e-02	0.6133169	-0.0215968517	8.364163e-01	9.420896e-01
0.9949051	-0.0544582295	5.643468e-01	0.9641086	-0.2257539208	7.136921e-02	3.034475e-01
0.8805172	-0.0521052409	6.047563e-01	0.9641086	-0.1436704279	2.149517e-01	5.227838e-01
0.5133973	0.6235366294	3.122988e-01	0.9300968	-0.1449958162	8.154100e-01	9.276368e-01
0.9476053	-0.8786633749	1.437155e-01	0.8166268	1.2293368566	3.793550e-01	6.707992e-01
0.9949051	0.1135571211	7.989812e-01	0.9957784	1.8277455327	6.272493e-05	2.058028e-03
0.8805172	-0.0048704423	9.651981e-01	0.9969722	-0.0111119753	8.959442e-01	9.686740e-01
0.9949051	0.0229338744	8.179377e-01	0.9957784	-0.0203563836	7.837202e-01	9.081934e-01
0.9949051	0.1681946616	3.699986e-01	0.9430561	-0.0271217483	9.012719e-01	9.710339e-01
0.9949051	-0.2057026251	7.346768e-02	0.7921508	-0.2540779130	2.843736e-02	1.785439e-01
0.9949051	-0.0636593428	5.181189e-01	0.9641086	-0.2007433944	2.221356e-02	1.467465e-01
0.9949051	-0.1189746677	2.642729e-01	0.9092411	-0.0465752117	7.053479e-01	8.762099e-01
0.9949051	0.5304671313	3.939172e-01	0.9430561	0.4497562236	4.741299e-01	7.506714e-01
0.2019302	0.1100266873	6.841788e-01	0.9733364	-0.0017972413	9.925696e-01	9.950155e-01
0.8497626	0.0803529763	2.995541e-01	0.9286466	0.0727365398	4.657258e-01	7.489631e-01
0.8210171	-0.1041428445	1.869861e-01	0.8526631	-0.0574623149	6.285416e-01	8.433108e-01
0.9949051	-0.1200733990	2.946337e-01	0.9286466	-0.1850248902	1.242620e-01	4.002115e-01
0.9949051	-0.0464163402	8.214496e-01	0.9957784	-0.3284301532	1.463087e-01	4.367095e-01
0.8730639	0.1350759265	6.158245e-01	0.9641086	-0.3268455907	2.451177e-01	5.554637e-01
0.9949051	0.0629082413	5.770311e-01	0.9641086	-0.2405820834	8.057305e-02	3.293325e-01
0.9275791	-0.4212893560	8.796665e-02	0.7921508	-0.3036155712	2.121613e-01	5.215962e-01
0.8598556	-0.2016582208	9.717586e-02	0.7921508	-0.1690813258	1.328028e-01	4.152722e-01
0.9949051	-0.0788216453	4.050993e-01	0.9430561	-0.0423764998	6.257250e-01	8.433108e-01
0.9210048	-0.0920662987	5.528385e-01	0.9641086	0.1192021789	5.934655e-01	8.274987e-01
0.9949051	0.0794744065	6.658844e-01	0.9717521	-0.4593480648	3.320794e-02	1.952575e-01
0.9135088	0.0578227160	7.815029e-01	0.9932650	-0.1970371652	9.468793e-02	3.558867e-01
0.9949051	-0.0883898058	4.102577e-01	0.9430561	-0.1009008991	2.683658e-01	5.832415e-01
0.9867694	-0.1752713659	1.856815e-01	0.8526631	-0.1070720489	5.459699e-01	7.942706e-01
0.8532963	-0.0021950240	9.828815e-01	0.9969722	-0.0961851222	2.024366e-01	5.098358e-01
0.8805172	-0.0290548410	7.236994e-01	0.9733364	-0.1190475404	1.302311e-01	4.093966e-01
0.9696873	-0.2733043823	1.257487e-01	0.8166268	-0.1933673535	3.623120e-01	6.629639e-01
0.9949051	-0.1022643318	3.194749e-01	0.9300968	-0.2011675637	1.362914e-01	4.217184e-01
0.9949051	0.0137359573	9.021467e-01	0.9969722	-0.1023162115	2.499895e-01	5.599450e-01
0.9949051	0.0610523105	8.979459e-01	0.9969722	-0.4330615596	1.342438e-01	4.186707e-01
0.9515733	0.0889506483	6.690105e-01	0.9717521	-0.2340024501	2.064048e-01	5.147057e-01
0.9210048	-0.1145206519	6.673982e-01	0.9717521	-0.1656282876	5.337124e-01	7.882615e-01
0.9949051	0.0354700979	9.433266e-01	0.9969722	2.2101068827	1.709290e-04	3.762862e-03
0.8878861	-0.3791784590	2.049521e-01	0.8590544	-0.0252185670	9.216962e-01	9.834194e-01
0.9949051	-0.3113551325	1.945338e-01	0.8526631	-0.2142914319	5.341706e-01	7.882615e-01
0.9949051	-0.0888277738	3.375847e-01	0.9366787	-0.0892432168	3.017265e-01	6.206462e-01
0.3287733	-1.3596679394	3.841497e-04	0.0782857	-0.5228828076	2.214048e-01	5.328026e-01
0.8612982	-0.2285616264	6.687471e-02	0.7921508	0.0851943322	4.509317e-01	7.361896e-01
0.5212145	-0.2658149509	7.064062e-01	0.9733364	-0.6544828629	1.878629e-01	4.902249e-01
0.9949051	-0.2329217309	8.910299e-02	0.7921508	-0.0019549204	9.875474e-01	9.950155e-01
0.6093251	-0.0209455779	8.633308e-01	0.9969722	0.0392813420	7.612914e-01	8.905775e-01
0.9949051	-0.0499428608	6.544454e-01	0.9689755	-0.0324363326	7.279294e-01	8.773616e-01
0.8805172	-0.2346564626	2.023805e-02	0.5125815	0.0252385569	7.772855e-01	9.051738e-01
0.8394240	-0.0341388538	8.852658e-01	0.9969722	0.1430029890	6.779780e-01	8.656656e-01
0.2019302	0.0008506750	9.963767e-01	0.9997294	-0.3618113047	9.158610e-02	3.503391e-01
0.8539066	-0.5936016263	1.634224e-01	0.8441531	-0.4273940448	3.077797e-01	6.214128e-01
0.7685693	-0.2532505776	1.892451e-01	0.8526631	-0.5223254878	1.444841e-02	1.074089e-01
0.9949051	-0.1920239040	1.009202e-01	0.8015257	-0.1232389002	2.515485e-01	5.601071e-01
0.9923046	-0.2963795236	6.592283e-02	0.7921508	0.3845822986	2.180883e-02	1.456386e-01
0.9949051	0.4228413325	4.571142e-01	0.9607204	0.5650978441	2.340967e-01	5.448022e-01
0.8329325	-0.0026423326	9.843203e-01	0.9969722	0.1364947613	2.035119e-01	5.107242e-01
0.8394240	-0.7554706158	4.622508e-03	0.2875687	-0.3099354313	1.775470e-01	4.802300e-01
0.9379096	-0.0843430729	6.144732e-01	0.9641086	0.0098889210	9.425850e-01	9.835175e-01
0.8194410	1.5307288375	5.009721e-01	0.9641086	1.7712115873	3.237436e-01	6.367136e-01
0.7194638	0.3860024145	2.340618e-01	0.8953432	-0.3687953434	1.225253e-01	3.995752e-01
0.9949051	-0.0863431899	5.594889e-01	0.9641086	-0.0758967007	6.612315e-01	8.563248e-01
0.7475613	-0.0334825353	8.220763e-01	0.9957784	0.1408325284	4.009712e-01	6.908862e-01
0.9949051	-0.0427458274	6.699221e-01	0.9717521	-0.1704108111	6.612105e-02	2.881189e-01
0.2019302	0.3149585436	5.248062e-01	0.9641086	-0.1681245102	7.423230e-01	8.836608e-01
0.7342939	0.0599964693	7.028999e-01	0.9733364	-0.1321494938	2.432974e-01	5.540992e-01
0.8805172	-0.0454463177	5.560985e-01	0.9641086	-0.1492455333	1.204191e-01	3.995752e-01
0.9949051	0.6112316485	2.395615e-01	0.8975264	0.0823177585	7.325610e-01	8.790733e-01
0.8442230	-0.1328427007	2.504479e-01	0.8975264	0.0390193098	7.203007e-01	8.762099e-01
0.7397893	-0.1808213458	1.414138e-01	0.8166268	-0.0362367215	7.875016e-01	9.116816e-01

0.9949051	-0.2984706035	6.091587e-01	0.9641086	-2.4277404321	2.866003e-04	5.377167e-03
0.8532963	-0.0954207464	3.660826e-01	0.9430561	-0.1434493614	2.509722e-01	5.601071e-01
0.9949051	-0.1929596206	2.898413e-03	0.2875687	-0.0314134658	7.118098e-01	8.762099e-01
0.6504978	-0.7856715639	7.847352e-02	0.7921508	0.3308966202	3.818718e-01	6.715871e-01
0.9210048	0.3684292419	6.254343e-01	0.9689755	0.0368725922	9.609803e-01	9.906899e-01
0.8805172	-0.0038868529	9.704448e-01	0.9969722	-0.0898010227	3.497288e-01	6.564148e-01
0.8497626	-0.0349883571	7.746770e-01	0.9920566	-0.0049377194	9.704461e-01	9.915358e-01
0.9949051	-0.6821997565	1.075818e-01	0.8123872	0.0156471611	9.653329e-01	9.911825e-01
0.8612982	0.5941188128	1.960021e-01	0.8526631	0.0029457188	9.953516e-01	9.961944e-01
0.9949051	0.0031669708	9.769391e-01	0.9969722	-0.1178742528	1.882928e-01	4.902249e-01
0.8194410	1.1011967286	2.258198e-01	0.8836164	-0.1287745187	7.696566e-01	8.980594e-01
0.8394240	0.2207127991	5.032746e-01	0.9641086	0.6864388601	4.934210e-02	2.440266e-01
0.8805172	4.3308168736	2.990816e-01	0.9286466	-0.5645640865	7.276220e-01	8.773616e-01
0.9998558	1.0788256873	1.345406e-01	0.8166268	2.0235074005	3.566208e-03	3.399401e-02
0.9949051	0.0281343420	9.235712e-01	0.9969722	1.4046503817	4.836725e-04	7.329498e-03
0.9949051	-0.1380471783	5.479635e-01	0.9641086	-0.4580355814	8.363482e-02	3.328497e-01
0.8997252	-0.1633012356	3.856441e-01	0.9430561	0.2334346453	1.083946e-01	3.790604e-01
0.9949051	-0.1367165477	8.766476e-02	0.7921508	-0.0777509944	4.882578e-01	7.621198e-01
0.5297647	0.2702556596	2.662687e-01	0.9096231	-0.2157082642	3.656589e-01	6.639152e-01
0.9949051	0.8620744303	4.430560e-01	0.9499322	6.2076958197	1.568920e-03	1.800451e-02
0.9109219	0.1170133185	4.881478e-01	0.9641086	-0.2788171713	8.683308e-02	3.348246e-01
0.9443280	-0.1147083751	5.401938e-01	0.9641086	0.0899621191	6.279334e-01	8.433108e-01
0.9949051	0.3162347081	2.161429e-01	0.8800012	0.9978934217	9.391021e-05	2.499111e-03
0.9949051	-0.0699297456	4.330079e-01	0.9482489	0.0055463499	9.512790e-01	9.854617e-01
0.9949051	-0.0106661932	9.143757e-01	0.9969722	-0.0785763656	3.109195e-01	6.238252e-01
0.5172057	-0.0768576578	8.089373e-01	0.9957784	0.0034239190	9.899525e-01	9.950155e-01
0.9493730	-0.1740685410	6.964578e-01	0.9733364	-0.9795524820	1.476737e-02	1.090940e-01
0.8497626	0.0824998082	6.436543e-01	0.9689755	-0.1244996851	5.583700e-01	8.039469e-01
0.9949051	-0.0362861629	7.550343e-01	0.9839586	-0.1080282124	4.154329e-01	7.014880e-01
0.8394240	0.5263507316	4.404303e-01	0.9482489	0.1316957881	8.079502e-01	9.227025e-01
0.8394240	-0.6285949426	4.938750e-02	0.7888653	-0.3499325752	1.920520e-01	4.928717e-01
0.9949051	-0.1268840144	3.337402e-01	0.9366787	0.1495232688	2.274876e-01	5.378807e-01
0.8540650	0.2493588452	4.185942e-01	0.9430561	-0.4817600803	1.409337e-01	4.313058e-01
0.9949051	0.0481601032	9.312230e-01	0.9969722	2.4088479820	4.541317e-04	7.131524e-03
0.9949051	-0.1509563551	1.829805e-01	0.8526631	-0.1361455577	9.425651e-02	3.558867e-01
0.9949051	0.0239805477	9.561618e-01	0.9969722	0.8651949890	3.204416e-01	6.344422e-01
0.8394240	-1.3053963953	1.198793e-01	0.8166268	1.6490058513	3.403477e-01	6.522734e-01
0.9949051	-0.0138411155	9.517057e-01	0.9969722	0.1280723939	7.281656e-01	8.773616e-01
0.9210048	-0.1087177058	8.868625e-01	0.9969722	-1.8965701588	9.070314e-03	7.497281e-02
0.9949051	0.0380564015	8.025276e-01	0.9957784	-0.0835101733	5.326472e-01	7.882615e-01
0.9210048	0.1245717112	5.551309e-01	0.9641086	-0.1854970566	3.567310e-01	6.629639e-01
0.9949051	0.2307492611	4.972735e-01	0.9641086	0.3628685027	2.580357e-01	5.700900e-01
0.9949051	-0.2610015891	3.977200e-02	0.6813116	-0.0215872322	8.840923e-01	9.640195e-01
0.8497626	-0.0951495447	3.926469e-01	0.9430561	0.0986205689	2.943132e-01	6.124616e-01
0.9949051	-0.0979804905	6.133687e-01	0.9641086	0.1165243704	4.526058e-01	7.363525e-01
0.9949051	0.1991057062	7.233593e-01	0.9733364	2.2987968583	6.964560e-05	2.058028e-03
0.9715057	0.4119065676	1.865247e-01	0.8526631	-0.0494438596	8.707230e-01	9.529580e-01
0.9715057	0.0840724086	3.663417e-01	0.9430561	0.0953619524	2.341451e-01	5.448022e-01
0.8194410	0.1031901388	5.368797e-01	0.9641086	0.0307236008	8.511006e-01	9.481630e-01
0.9949051	0.0592595153	7.576233e-01	0.9851603	-0.1838086787	2.904086e-01	6.064468e-01
0.9949051	0.0281745598	9.211981e-01	0.9969722	0.1017303420	7.344032e-01	8.794980e-01
0.8394240	-0.2955516208	7.225343e-01	0.9733364	-0.4737292702	3.256011e-01	6.382430e-01
0.8532963	-0.0249002729	8.601061e-01	0.9969722	0.0603632152	6.585743e-01	8.563248e-01
0.9949051	0.1516655072	3.458876e-01	0.9398602	-0.0030917285	9.858100e-01	9.950155e-01
0.9949051	0.0768639697	6.185092e-01	0.9641086	-0.1203493542	3.525701e-01	6.604404e-01
0.9210048	0.0244676080	9.260954e-01	0.9969722	0.0065913779	9.829589e-01	9.950155e-01
0.9981675	-0.0052162261	9.516926e-01	0.9969722	-0.0833041511	3.495482e-01	6.564148e-01
0.9949051	-0.0716238745	3.929502e-01	0.9430561	-0.0677700006	4.866651e-01	7.621198e-01
0.9210048	0.3435337665	2.689340e-01	0.9106469	0.2798469213	2.606325e-01	5.723906e-01
0.8394240	-0.0895941342	1.370353e-01	0.8166268	-0.0547787557	2.719991e-01	5.856155e-01
0.8194410	-0.2766659006	5.220341e-03	0.3085222	-0.1950198697	1.119020e-01	3.878831e-01
0.9949051	-0.1827886214	4.736902e-01	0.9641086	-0.4897319349	8.561527e-02	3.339128e-01
0.5471002	0.0624466522	5.192921e-01	0.9641086	-0.0823716998	5.140939e-01	7.800501e-01
0.9148164	-0.0803625240	5.896566e-01	0.9641086	-0.0863613178	5.707695e-01	8.131842e-01
0.9949051	0.1406104377	4.574989e-01	0.9607204	-0.4336175580	7.514541e-02	3.172210e-01
0.8088570	-0.3642588997	2.546955e-01	0.9016579	-0.1419071470	6.366761e-01	8.493805e-01
0.9949051	0.0850993997	7.306178e-01	0.9733364	-0.2045933053	4.052099e-01	6.952547e-01
0.8194410	0.0438924478	7.711145e-01	0.9919874	-0.1801291961	2.684831e-01	5.832415e-01
0.8741974	0.0660691225	6.187484e-01	0.9641086	0.0501508636	6.435498e-01	8.512682e-01
0.8394240	-0.0299244212	6.957404e-01	0.9733364	-0.2315738590	1.512719e-02	1.106554e-01
0.9949051	-0.0165875950	9.670545e-01	0.9969722	1.8393864285	4.002388e-05	1.613603e-03
0.9949051	0.0502294357	8.115428e-01	0.9957784	-0.2578781367	2.501277e-01	5.599450e-01
0.7685693	0.2288429661	6.870524e-01	0.9733364	-0.5337894267	1.184209e-01	3.995752e-01
0.8194410	-0.1252504433	6.063247e-01	0.9641086	-0.2829717988	2.245104e-01	5.339463e-01
0.9949051	-0.1102512255	3.155869e-01	0.9300968	-0.0444323938	6.218472e-01	8.433108e-01
0.8497626	-0.1193408665	2.401003e-01	0.8975264	-0.0825251707	3.693314e-01	6.675072e-01
0.8532963	0.1834593649	6.275509e-01	0.9689755	-0.7242336248	2.918355e-03	2.923301e-02
0.9949051	0.0313337507	7.275314e-01	0.9733364	-0.1246454247	1.417247e-01	4.317490e-01
0.9949051	-0.0584135877	6.061678e-01	0.9641086	-0.0085466489	9.444330e-01	9.835175e-01
0.9949051	0.0797817400	4.677551e-01	0.9641086	-0.0037767978	9.745725e-01	9.927624e-01
0.6922402	-0.2368794598	8.030195e-02	0.7921508	-0.0970749962	5.996776e-01	8.313707e-01
0.9210048	-0.0066527842	9.916883e-01	0.9997294	1.8072232015	9.352322e-02	3.558867e-01
0.9949051	0.6689899565	3.925972e-01	0.9430561	3.1045039015	1.225898e-03	1.476075e-02
0.9949051	0.0563071819	8.493669e-01	0.9969722	0.0702416248	8.259028e-01	9.359704e-01
0.9949051	-0.0291974091	6.780405e-01	0.9726260	-0.0106292873	9.018196e-01	9.710339e-01
0.9949051	0.1498393959	7.372916e-02	0.7921508	-0.1207872312	9.514399e-02	3.558867e-01
0.9135088	-0.1196937450	3.391163e-01	0.9387248	-0.0586023952	7.133506e-01	8.762099e-01
0.7815947	-0.2326766423	1.662719e-02	0.4760109	-0.0344140028	7.252583e-01	8.773616e-01
0.8540650	-0.0999921458	7.995333e-01	0.9957784	-0.1842347648	6.191225e-01	8.433108e-01
0.8194410	0.0745060714	5.646834e-01	0.9641086	-0.1151227411	4.606507e-01	7.428229e-01
0.9949051	-0.0305338534	8.491357e-01	0.9969722	0.7517554553	9.391109e-04	1.206553e-02
0.8540650	-0.2613971630	2.020970e-01	0.8526631	0.0108714766	9.613547e-01	9.906899e-01
0.9949051	-0.0762784847	5.555143e-01	0.9641086	-0.0677389710	6.686618e-01	8.600198e-01
0.8194410	-0.0059470003	9.670745e-01	0.9969722	-0.0517745390	7.359147e-01	8.795259e-01
0.9694325	-0.0179109986	8.749187e-01	0.9969722	-0.1469477685	5.973135e-02	2.790611e-01
0.9787973	-0.2792386559	2.202461e-01	0.8800012	0.1184041354	4.971565e-01	7.643323e-01
0.8497626	-0.0712223217	7.741977e-01	0.9920566	-0.0867056993	5.972476e-01	8.305255e-01
0.7696781	-0.1399788731	8.621385e-02	0.7921508	-0.0566948352	5.381235e-01	7.891587e-01
0.9314626	0.1853123332	5.192143e-01	0.9641086	-0.0298423448	9.105231e-01	9.730907e-01
0.9949051	0.2921286283	2.001158e-01	0.8526631	0.1050693859	5.950940e-01	8.285054e-01
0.9949051	-0.0819371750	6.505486e-01	0.9689755	0.2353729491	1.412143e-01	4.313058e-01
0.9949051	0.0981323737	4.612887e-01	0.9641086	-0.0040198124	9.724271e-01	9.922242e-01
0.8194410	-0.1018018969	3.814095e-01	0.9430561	-0.2318135943	4.394703e-02	2.329390e-01
0.8805172	1.6625759901	3.435242e-01	0.9398602	1.7052944574	3.627573e-01	6.629639e-01
0.9867694	-0.0946404943	3.247879e-01	0.9364851	-0.0827161539	4.186793e-01	7.059613e-01
0.8264615	-0.2011677849	2.513378e-01	0.8975264	-0.0768687673	7.072982e-01	8.762099e-01
0.8612982	-0.0829836515	7.343993e-01	0.9764454	-0.5226590074	2.886166e-02	1.785439e-01
0.9236575	-0.0785758992	7.241095e-01	0.9733364	-0.2679213566	3.068233e-01	6.214128e-01
0.8264615	0.1141813733	6.255424e-01	0.9689755	-0.2677250809	2.939652e-02	1.800346e-01
0.9012921	0.6322123350	3.234658e-01	0.9364851	-0.4169484481	1.569151e-01	4.523748e-01
0.9981675	0.0634075810	7.489295e-01	0.9823061	0.5097407783	8.217890e-03	6.938247e-02
0.2034892	0.1193761124	4.788923e-01	0.9641086	-0.3774985718	2.291127e-02	1.504507e-01
0.5133973	0.8663438826	4.803375e-01	0.9641086	-2.3644485762	1.418332e-02	1.067814e-01
0.8817422	-0.1407609439	1.750145e-01	0.8526631	-0.0062599286	9.582368e-01	9.906899e-01
0.9949051	-0.2226310855	4.885161e-01	0.9641086	1.0380331530	2.210162e-04	4.433815e-03
0.9210048	-0.0337692960	8.827700e-01	0.9969722	0.0535531258	8.040848e-01	9.209576e-01
0.9949051	-0.1563281233	2.562630e-01	0.9016579	-0.0742305172	5.936708e-01	8.274987e-01
0.8210171	-0.0016851966	9.866788e-01	0.9981904	0.0742891211	4.899366e-01	7.621198e-01
0.8497626	0.2517325258	2.512024e-01	0.8975264	-0.2240210373	1.019662e-01	3.685750e-01
0.9148164	-0.0238472628	8.951797e-01	0.9969722	-0.4455863248	3.040716e-02	1.847083e-01
0.9949051	0.1489471754	3.683260e-01	0.9430561	0.6959123367	1.719074e-04	3.762862e-03
0.9210048	0.0801063348	5.620670e-01	0.9641086	0.2461787344	1.207529e-01	3.995752e-01
0.9148164	0.0862163085	7.200174e-01	0.9733364	-0.0695242003	7.443560e-01	8.842500e-01
0.9949051	0.1026865167	8.033647e-01	0.9957784	1.9777938502	2.191837e-05	1.126414e-03
0.8194410	-0.1838218284	1.284139e-01	0.8166268	-0.1194040952	4.289743e-01	7.159073e-01
0.9949051	-0.1604914566	1.884286e-01	0.8526631	-0.0413620147	7.485960e-01	8.857262e-01
0.8394240	-0.1473380812	1.330325e-01	0.8166268	-0.0791654581	4.621918e-01	7.442924e-01
0.9379096	-0.2545566783	2.560629e-01	0.9016579	0.2150069221	3.452416e-01	6.539822e-01
0.8394240	-0.1333162365	2.197161e-01	0.8800012	-0.1259839303	2.599759e-01	5.722374e-01
0.9949051	-0.1544923211	5.675948e-01	0.9641086	0.4759393472	7.563416e-02	3.181480e-01
0.9949051	0.1590965008	8.220153e-01	0.9957784	3.1161283580	1.159143e-04	2.854391e-03
0.9949051	0.3787638458	6.495492e-01	0.9689755	2.9793852743	6.595928e-04	9.172220e-03
0.9949051	0.3638369776	5.167544e-01	0.9641086	-0.3676564896	1.787627e-01	4.809469e-01
0.9949051	-0.3256248203	5.885510e-01	0.9641086	0.1629641085	8.162416e-01	9.276900e-01
0.9949051	0.1109199535	7.384652e-01	0.9767472	0.0823043251	8.352425e-01	9.420388e-01
0.8497626	0.8824502996	3.288510e-01	0.9366787	-0.0169421210	9.660255e-01	9.911825e-01
0.9949051	-1.0231313868	6.038504e-03	0.3216097	-1.2536408302	3.819075e-04	6.448781e-03
0.9109219	0.4170829658	2.920861e-01	0.9286466	1.5576764171	3.193034e-05	1.451602e-03
0.9949051	-0.4692865381	1.716302e-01	0.8467562	0.4234376152	4.529004e-01	7.363525e-01

0.9949051	-0.1797009651	1.961725e-01	0.8526631	-0.1237388526	3.801932e-01	6.707992e-01
0.9949051	-0.0782626436	5.815475e-01	0.9641086	-0.1544952400	2.707378e-01	5.850313e-01
0.9949051	-0.1330575168	4.072653e-01	0.9430561	-0.3751329597	4.757611e-02	2.391031e-01
0.9949051	0.1753893935	4.917820e-01	0.9641086	0.7105332191	3.436618e-03	3.302506e-02
0.2019302	-0.1130050618	5.565919e-01	0.9641086	-0.1873185534	3.577382e-01	6.629639e-01
0.9949051	-0.0329872349	7.743501e-01	0.9920566	-0.0105283845	9.323986e-01	9.835175e-01
0.9949051	0.0445787087	7.589807e-01	0.9855489	0.2645469557	1.173676e-03	1.440394e-02
0.6525817	0.1469291555	8.117491e-01	0.9957784	-1.0741115885	1.144415e-01	3.920865e-01
0.9949051	-0.0615214545	6.619906e-01	0.9689755	0.0158795835	9.485701e-01	9.835175e-01
0.9961627	0.3574895251	4.891525e-01	0.9641086	2.4184838244	5.772679e-07	2.496980e-04
0.5133973	-0.0749125228	4.215569e-01	0.9430561	-0.0537871827	5.249301e-01	7.873952e-01
0.5133973	-0.1077093007	6.609224e-01	0.9689755	0.2355270894	4.711473e-01	7.506714e-01
0.9949051	-0.2882333197	3.435788e-01	0.9398602	0.0417430694	8.856397e-01	9.645345e-01
0.9949051	-0.0158621907	8.850935e-01	0.9969722	-0.1733785826	2.453063e-01	5.554637e-01
0.7815947	-0.1849807464	1.209735e-01	0.8166268	-0.1872761370	1.810370e-01	4.841307e-01
0.8264615	0.3930870504	4.097994e-01	0.9430561	-0.1222639999	6.512429e-01	8.534026e-01
0.9949051	-0.0646817822	4.920222e-01	0.9641086	-0.0528866891	5.299484e-01	7.882615e-01
0.8497626	0.2095419113	3.659031e-01	0.9430561	-0.0320822123	8.508605e-01	9.481630e-01
0.8194410	0.1788857292	9.457845e-02	0.7921508	0.0573236728	5.999655e-01	8.313707e-01
0.8194410	-0.1989485750	2.110569e-01	0.8699563	0.0106558012	9.302558e-01	9.835175e-01
0.9949051	0.0878454147	8.498921e-01	0.9969722	2.0347795210	6.803885e-04	9.243899e-03
0.9949051	-0.5969068266	2.392882e-02	0.5545856	0.0493336219	8.636541e-01	9.515996e-01
0.8899481	-0.0914761142	2.954452e-01	0.9286466	-0.1283829525	1.453403e-01	4.367095e-01
0.9949051	0.0608280365	5.154835e-01	0.9641086	-0.1510676994	1.538092e-01	4.468082e-01
0.9949051	0.0412700889	8.188960e-01	0.9957784	-0.0032157786	9.813823e-01	9.950155e-01
0.8497626	0.1209421641	5.696015e-01	0.9641086	-0.1688549939	4.327399e-01	7.187080e-01
0.8394240	-0.0471119625	8.175660e-01	0.9957784	-0.3799210066	1.224904e-01	3.995752e-01
0.8612982	0.2335536067	3.260264e-01	0.9364851	-0.0134592208	9.589559e-01	9.906899e-01
0.9949051	0.0053604535	9.684146e-01	0.9969722	-0.0161277558	9.315717e-01	9.835175e-01
0.9949051	0.0555137147	7.036087e-01	0.9733364	-0.0018690179	9.904276e-01	9.950155e-01
0.9949051	-0.1487631241	5.049923e-01	0.9641086	-0.0486876038	8.052220e-01	9.213673e-01
0.8497626	0.4027415000	3.322759e-01	0.9366787	-0.2923802478	5.477884e-01	7.942706e-01
0.9949051	-0.2322367519	4.965154e-01	0.9641086	-0.2580624920	4.756792e-01	7.506714e-01
0.9949051	-0.0543792304	8.311516e-01	0.9964090	-0.5008165520	1.026625e-01	3.699605e-01
0.9210048	-0.2338254102	6.839536e-03	0.3233732	-0.1454039905	1.842667e-01	4.883481e-01
0.9949051	-0.0053226403	9.583951e-01	0.9969722	-0.1998510513	4.901149e-02	2.440266e-01
0.2019302	-0.1258119039	2.489890e-01	0.8975264	-0.0524143243	7.044277e-01	8.762099e-01
0.9949051	0.0181847127	8.313342e-01	0.9964090	-0.0866051114	3.179768e-01	6.316783e-01
0.8329325	-0.0284247784	6.464281e-01	0.9689755	0.1313834657	1.625726e-02	1.164611e-01
0.8194410	0.0222095477	9.285639e-01	0.9969722	0.3560881674	2.740451e-01	5.878791e-01
0.8612982	-0.0654570113	4.217826e-01	0.9430561	-0.1205355095	1.878537e-01	4.902249e-01
0.9949051	0.0191987041	7.918298e-01	0.9957784	-0.0591301084	4.798221e-01	7.551927e-01
0.7815947	-0.1576906055	2.795692e-01	0.9128476	0.0961631382	4.394800e-01	7.244984e-01
0.8539066	-0.1247654978	3.657274e-01	0.9430561	-0.0657523375	6.067154e-01	8.360191e-01
0.8497626	-0.0265137362	9.030955e-01	0.9969722	0.0414804676	8.441055e-01	9.481630e-01
0.9949051	-0.0108314463	9.434920e-01	0.9969722	-0.2712514409	3.091853e-01	6.215256e-01
0.9949051	-0.1900940469	1.079059e-01	0.8123872	-0.1475016522	1.393298e-01	4.288747e-01
0.9949051	-0.0976022999	1.844083e-01	0.8526631	-0.0091988421	8.877113e-01	9.645345e-01
0.9949051	-0.0573070062	8.127038e-01	0.9957784	0.1825386342	8.043080e-02	3.293325e-01
0.9949051	-0.1238537102	1.278491e-01	0.8166268	0.0134606232	8.620432e-01	9.515996e-01
0.9949051	-0.0939124630	3.193831e-01	0.9300968	0.0758158543	4.123435e-01	6.984455e-01
0.4880000	0.0535474771	8.369989e-01	0.9969722	-0.1390791343	6.542333e-01	8.556317e-01
0.8805172	-0.2650113520	8.031871e-02	0.7921508	-0.2171829650	1.451518e-01	4.367095e-01
0.9949051	-0.3283403120	3.393336e-02	0.6684872	-0.2759874166	1.765751e-01	4.802300e-01
0.9210048	-0.3890605504	1.192068e-01	0.8166268	-0.1872531885	4.437237e-01	7.274360e-01
0.6383206	0.0709486046	5.842494e-01	0.9641086	0.1270765466	1.669443e-01	4.676259e-01
0.9275791	0.0573348238	8.781793e-01	0.9969722	0.5519186425	1.445746e-01	4.367095e-01
0.8741974	0.1071255968	5.483134e-01	0.9641086	0.0013581593	9.929049e-01	9.950155e-01
0.9379096	-0.0338174744	8.050012e-01	0.9957784	-0.0449588946	7.301648e-01	8.778587e-01
0.7817234	-0.1192755776	4.178576e-01	0.9430561	0.0657433691	6.444720e-01	8.512682e-01
0.9949051	-0.0459318426	6.899839e-01	0.9733364	-0.0784821034	4.880648e-01	7.621198e-01
0.9210048	-0.0232901695	8.191638e-01	0.9957784	-0.1323811851	1.673484e-01	4.676259e-01
0.9949051	-0.0654097021	5.530803e-01	0.9641086	-0.1226016551	4.362379e-01	7.217613e-01
0.9149215	-0.1413236841	1.450860e-01	0.8166268	-0.1104522136	2.148346e-01	5.227838e-01
0.8210171	-0.0857618031	4.760962e-01	0.9641086	-0.0083410629	9.323935e-01	9.835175e-01
0.9509918	-0.2561924104	1.985913e-01	0.8526631	-0.2334207404	3.467819e-01	6.547862e-01
0.6922402	-0.5038100964	1.252427e-01	0.8166268	0.0050323142	9.854143e-01	9.950155e-01
0.9949051	-0.3045161787	1.237596e-01	0.8166268	-0.5179842018	9.247293e-03	7.590486e-02
0.9949051	0.1893579803	4.735586e-01	0.9641086	-0.4813548846	3.247664e-02	1.919370e-01
0.9148164	0.0464116734	7.469457e-01	0.9820799	0.0493986034	7.743734e-01	9.026719e-01
0.9949051	-0.8882642604	1.564126e-01	0.8386867	-0.8957941660	1.459797e-01	4.367095e-01
0.9949051	0.0671031410	9.062983e-01	0.9969722	2.3238708336	8.814453e-04	1.157632e-02
0.8394240	-0.2488406505	2.966816e-01	0.9286466	-0.0160334553	9.360851e-01	9.835175e-01
0.8394240	0.0295101708	8.929535e-01	0.9969722	-0.0029102060	9.871003e-01	9.950155e-01
