# Supplementary material for: Systemic Blood Proteome Patterns Reflect Disease Phenotypes in Neovascular Age-Related Macular Degeneration
Source: Int J Mol Sci. 2023 Jun 19;24(12):10327. doi: 10.3390/ijms241210327 (PMC10299094; doi:10.3390/ijms241210327)
Supplement: Supplementary file 1 [file ijms-24-10327-s001.zip › Table S7.pdf]

	Age	Age	BCVAlogmar	BCVAlogmar	CRT	CRT
.	Pearson R	Adj. p-val (Benjamini-H. cor.)	Pearson R	Adj. p-val (Benjamini-H. cor.)	Pearson R	Adj. p-val (Benjamini-H. cor.)
**HBB-HUMAN**	-0.0094747507	0.9989736	-1.132545e-01	0.999995089	1.025305e-01	0.9996259
**HBA-HUMAN**	-0.2524118991	0.9989736	-8.129292e-02	0.999995089	-9.518872e-02	0.9996259
**HBG1-HUMAN**	0.2226029774	0.9989736	1.280711e-01	0.999995089	2.625570e-02	0.9996259
**ALBU-HUMAN**	-0.1507857994	0.9989736	2.406242e-02	0.999995089	2.325082e-01	0.9996259
**HBD-HUMAN**	-0.1365847156	0.9989736	-1.296714e-02	0.999995089	-8.144655e-02	0.9996259
**IGG1-HUMAN**	0.1879431784	0.9989736	1.426181e-01	0.999995089	-1.372544e-01	0.9996259
**CAH1-HUMAN**	-0.1001769750	0.9989736	8.192268e-02	0.999995089	-5.382155e-02	0.9996259
**PRDX2-HUMAN**	0.1385683080	0.9989736	-6.896846e-02	0.999995089	-3.337711e-02	0.9996259
**IGLL1-HUMAN**	0.0273209346	0.9989736	3.457592e-02	0.999995089	-8.364049e-02	0.9996259
**IGKC-HUMAN**	0.2844264556	0.9989736	1.470249e-02	0.999995089	-1.574230e-01	0.9996259
**APOA1-HUMAN**	0.0981835589	0.9989736	-1.322333e-01	0.999995089	1.295967e-01	0.9996259
**TRFE-HUMAN**	-0.0900151846	0.9989736	5.276310e-02	0.999995089	2.216107e-01	0.9996259
**FIBB-HUMAN**	0.0255274006	0.9989736	-2.973093e-01	0.999995089	-1.360823e-01	0.9996259
**FIBG-HUMAN**	0.0867500087	0.9989736	-1.651768e-01	0.999995089	-1.295869e-01	0.9996259
**A1AT-HUMAN**	0.1107520220	0.9989736	-1.526973e-01	0.999995089	6.642712e-02	0.9996259
**IGL1-HUMAN**	0.0311613581	0.9989736	1.526464e-01	0.999995089	-7.114064e-02	0.9996259
**IGHG2-HUMAN**	-0.0147820690	0.9989736	-4.510608e-02	0.999995089	2.257594e-01	0.9996259
**BLVRB-HUMAN**	0.0263927877	0.9989736	-6.901870e-02	0.999995089	-5.443670e-02	0.9996259
**IGK-HUMAN**	0.2626556894	0.9989736	1.431021e-01	0.999995089	-1.524098e-02	0.9996259
**CD2AP-HUMAN**	-0.0027995248	0.9989736	-1.103446e-01	0.999995089	-7.228017e-02	0.9996259
**CAH2-HUMAN**	0.1063319954	0.9989736	3.552441e-02	0.999995089	-3.948630e-02	0.9996259
**FIBA-HUMAN**	0.0862491007	0.9989736	-1.296168e-01	0.999995089	-1.662204e-02	0.9996259
**B3AT-HUMAN**	0.2291645101	0.9989736	8.486740e-02	0.999995089	-3.269296e-02	0.9996259
**IGHA1-HUMAN**	0.2980048678	0.9989736	2.660745e-01	0.999995089	-7.741874e-02	0.9996259
**RL11-HUMAN**	-0.1315238210	0.9989736	-5.133354e-02	0.999995089	-7.745822e-02	0.9996259
**IGLC7-HUMAN**	0.1258883754	0.9989736	1.538208e-01	0.999995089	-1.609290e-01	0.9996259
**A2MG-HUMAN**	0.3430264338	0.9989736	9.182834e-02	0.999995089	-1.226215e-01	0.9996259
**CATA-HUMAN**	-0.0579507898	0.9989736	1.226700e-01	0.999995089	-4.503422e-02	0.9996259
**PLF4-HUMAN**	-0.1472029138	0.9989736	-5.119846e-02	0.999995089	8.155337e-02	0.9996259
**DEF3-HUMAN**	0.2102108627	0.9989736	-3.321043e-02	0.999995089	2.369613e-01	0.9996259
**HPT-HUMAN**	0.0082059850	0.9989736	-4.134052e-02	0.999995089	-9.548382e-02	0.9996259
**IGLC2-HUMAN;IGLC3-HUMAN**	-0.1366688555	0.9989736	2.887275e-02	0.999995089	-1.736008e-01	0.9996259
**APOB-HUMAN**	0.2455709681	0.9989736	-1.111385e-01	0.999995089	-2.942724e-02	0.9996259
**CO3-HUMAN**	-0.0848837616	0.9989736	2.833569e-03	0.999995089	9.997077e-02	0.9996259
**APOA2-HUMAN**	-0.2143323858	0.9989736	-1.959415e-01	0.999995089	9.977885e-02	0.9996259
**A1AG1-HUMAN**	-0.0287283891	0.9989736	-4.083543e-02	0.999995089	-2.375006e-01	0.9996259
**ACTB-HUMAN**	-0.0222338919	0.9989736	-4.716090e-02	0.999995089	1.078308e-01	0.9996259
**PRDX6-HUMAN**	0.1890053908	0.9989736	8.439363e-03	0.999995089	9.018326e-02	0.9996259
**MINP1-HUMAN**	-0.1843097948	0.9989736	1.001554e-02	0.999995089	-2.331534e-01	0.9996259
**IGM-HUMAN**	-0.1991984642	0.9989736	-3.967645e-02	0.999995089	3.231271e-01	0.9996259
**TRY1-HUMAN**	-0.0785847729	0.9989736	6.724557e-04	0.999995089	4.770085e-03	0.9996259
**G3P-HUMAN**	-0.1639693230	0.9989736	-1.315195e-01	0.999995089	-1.017513e-02	0.9996259
**S10A6-HUMAN**	-0.1878397162	0.9989736	-2.312244e-02	0.999995089	-1.382038e-01	0.9996259
**ACTA-HUMAN;ACTH-HUMAN**	0.0034559405	0.9989736	-5.823069e-02	0.999995089	1.365077e-01	0.9996259
**FETUA-HUMAN**	0.0107638414	0.9989736	-1.221247e-01	0.999995089	2.250591e-02	0.9996259
**UBB-HUMAN;UBC-HUMAN;RS27A-HUMAN;RL40-HUMAN**	-0.0599673389	0.9989736	4.142764e-02	0.999995089	3.487862e-02	0.9996259
**IGA2-HUMAN**	0.1730712761	0.9989736	2.382867e-01	0.999995089	-2.006378e-02	0.9996259
**APOA4-HUMAN**	0.0752050119	0.9989736	-1.377486e-01	0.999995089	-3.695902e-02	0.9996259
**ANK1-HUMAN**	-0.1059293762	0.9989736	-1.797187e-01	0.999995089	6.575143e-02	0.9996259
**SPTB1-HUMAN**	0.1478063487	0.9989736	-1.122896e-01	0.999995089	-1.850929e-01	0.9996259
**IGHG3-HUMAN**	0.0675431104	0.9989736	-1.173897e-01	0.999995089	-2.613125e-01	0.9996259
**HEMO-HUMAN**	-0.0121964911	0.9989736	-1.754047e-01	0.999995089	2.551521e-01	0.9996259
**PMGE-HUMAN**	-0.0424810452	0.9989736	2.670387e-02	0.999995089	-5.122575e-02	0.9996259
**APOC3-HUMAN**	-0.3282899935	0.9989736	-1.256748e-01	0.999995089	4.571085e-02	0.9996259
**EPB42-HUMAN**	0.0937247954	0.9989736	7.054944e-02	0.999995089	-1.860398e-02	0.9996259
**SBP1-HUMAN**	-0.0386962579	0.9989736	-3.772170e-02	0.999995089	-2.232810e-01	0.9996259
**PFD2-HUMAN**	-0.0786903132	0.9989736	-1.692650e-01	0.999995089	3.883127e-02	0.9996259
**A1AG2-HUMAN**	-0.0480204818	0.9989736	-3.044017e-02	0.999995089	4.937619e-03	0.9996259
**SPTA1-HUMAN**	0.0112087025	0.9989736	9.303369e-02	0.999995089	-1.648866e-02	0.9996259
**HEM2-HUMAN**	-0.0695119600	0.9989736	5.241783e-02	0.999995089	-1.499500e-01	0.9996259
**VTDB-HUMAN**	-0.0399855222	0.9989736	-1.093890e-01	0.999995089	8.494070e-02	0.9996259
**ITIH4-HUMAN**	-0.0542390726	0.9989736	-9.856462e-02	0.999995089	1.072151e-02	0.9996259
**KV228-HUMAN;KVD28-HUMAN**	-0.0841110758	0.9989736	2.718649e-01	0.999995089	2.923523e-01	0.9996259
**PRDX1-HUMAN**	-0.0514880183	0.9989736	2.191758e-01	0.999995089	4.217880e-02	0.9996259
**RARR2-HUMAN**	-0.0198801228	0.9989736	-4.579771e-02	0.999995089	9.885206e-02	0.9996259
**KV315-HUMAN**	0.0529940532	0.9989736	1.361835e-01	0.999995089	3.241009e-02	0.9996259
**CO4A-HUMAN**	0.1484128791	0.9989736	-2.414746e-01	0.999995089	-2.188607e-01	0.9996259
**KVD20-HUMAN**	0.1602110015	0.9989736	1.118842e-01	0.999995089	2.963319e-01	0.9996259
**AACT-HUMAN**	0.1020944660	0.9989736	-1.088984e-01	0.999995089	7.812739e-03	0.9996259

**A1BG-HUMAN**	-0.0013684860	0.9989736	-1.621034e-01	0.999995089	-7.051880e-02	0.9996259
**FINC-HUMAN**	-0.1714269974	0.9989736	-2.489976e-01	0.999995089	1.237711e-01	0.9996259
**RAN-HUMAN**	-0.0637983465	0.9989736	-2.207141e-01	0.999995089	-1.087625e-01	0.9996259
**KNG1-HUMAN**	0.0825807136	0.9989736	2.902992e-02	0.999995089	-1.365044e-01	0.9996259
**C4BPA-HUMAN**	-0.0173020103	0.9989736	-9.085677e-02	0.999995089	-1.218628e-01	0.9996259
**PNPH-HUMAN**	-0.0824303263	0.9989736	1.562048e-01	0.999995089	5.307557e-04	0.9996259
**IC1-HUMAN**	0.1165955736	0.9989736	-6.902439e-03	0.999995089	-1.232932e-01	0.9996259
**VTNC-HUMAN**	-0.0189833433	0.9989736	8.199065e-02	0.999995089	1.416948e-01	0.9996259
**GSTO1-HUMAN**	-0.1840385038	0.9989736	-4.570042e-02	0.999995089	-1.411473e-01	0.9996259
**ANT3-HUMAN**	0.0397431130	0.9989736	-9.728562e-02	0.999995089	3.511257e-03	0.9996259
**UB2V1-HUMAN**	-0.0893921209	0.9989736	6.678570e-02	0.999995089	-5.405692e-02	0.9996259
**KV320-HUMAN**	-0.0869490119	0.9989736	7.147099e-02	0.999995089	-5.207086e-02	0.9996259
**MDHC-HUMAN**	0.1360577475	0.9989736	-3.784280e-02	0.999995089	-2.363459e-01	0.9996259
**NDKB-HUMAN**	-0.1769253156	0.9989736	-1.801290e-02	0.999995089	1.571046e-01	0.9996259
**PARK7-HUMAN**	-0.1360051227	0.9989736	3.775277e-02	0.999995089	5.498032e-02	0.9996259
**HBG2-HUMAN**	-0.2314903534	0.9989736	1.128268e-01	0.999995089	-3.312514e-02	0.9996259
**ALDOA-HUMAN**	0.0988163008	0.9989736	2.144225e-02	0.999995089	-3.520728e-02	0.9996259
**ITIH2-HUMAN**	0.0176872615	0.9989736	5.044747e-03	0.999995089	1.394576e-01	0.9996259
**FCG3A-HUMAN**	-0.0985333692	0.9989736	2.142370e-01	0.999995089	8.602853e-02	0.9996259
**AL1A1-HUMAN**	0.0193320509	0.9989736	1.080418e-01	0.999995089	-2.202604e-01	0.9996259
**SYUA-HUMAN**	0.0710385338	0.9989736	-1.203446e-01	0.999995089	1.166551e-01	0.9996259
**PLMN-HUMAN**	-0.1604107811	0.9989736	9.625698e-02	0.999995089	1.157532e-01	0.9996259
**CSN5-HUMAN**	-0.1623792073	0.9989736	1.182512e-01	0.999995089	8.328411e-02	0.9996259
**TERA-HUMAN**	-0.2011442121	0.9989736	1.097759e-01	0.999995089	-4.957896e-02	0.9996259
**GDIB-HUMAN**	-0.1182617991	0.9989736	-1.036763e-01	0.999995089	-4.874906e-02	0.9996259
**LDHA-HUMAN**	-0.0929712061	0.9989736	-4.258636e-03	0.999995089	8.682824e-02	0.9996259
**LDHB-HUMAN**	0.1978603913	0.9989736	4.890326e-02	0.999995089	-2.584479e-01	0.9996259
**S10A4-HUMAN**	0.0010575295	0.9989736	-4.230648e-02	0.999995089	-3.109163e-01	0.9996259
**CFAH-HUMAN**	-0.0231096255	0.9989736	-2.667530e-02	0.999995089	2.085269e-02	0.9996259
**1433B-HUMAN**	0.2703082754	0.9989736	1.154455e-01	0.999995089	-1.508731e-01	0.9996259
**KVD11-HUMAN;KV311-HUMAN**	0.0728747399	0.9989736	-3.429461e-02	0.999995089	1.080595e-01	0.9996259
**KV401-HUMAN**	0.0426350471	0.9989736	1.137564e-01	0.999995089	5.616868e-02	0.9996259
**CFAB-HUMAN**	-0.1362195744	0.9989736	-5.869694e-02	0.999995089	-2.019863e-02	0.9996259
**PPIA-HUMAN**	-0.0188964081	0.9989736	8.436790e-02	0.999995089	1.282114e-01	0.9996259
**C1QC-HUMAN**	-0.0422216906	0.9989736	-8.720201e-02	0.999995089	-1.200393e-01	0.9996259
**KAD1-HUMAN**	-0.0646199671	0.9989736	-5.540832e-02	0.999995089	-1.969628e-01	0.9996259
**C1QB-HUMAN**	0.1455666199	0.9989736	1.394468e-01	0.999995089	1.708475e-01	0.9996259
**LV861-HUMAN**	-0.1014964427	0.9989736	-2.042139e-02	0.999995089	-1.042306e-01	0.9996259
**HV118-HUMAN**	0.0633408843	0.9989736	-1.742586e-01	0.999995089	-4.899490e-02	0.9996259
**TPIS-HUMAN**	-0.0207938003	0.9989736	7.563657e-02	0.999995089	-8.842008e-02	0.9996259
**HRG-HUMAN**	0.1675703210	0.9989736	-8.126611e-02	0.999995089	1.479105e-01	0.9996259
**DEMA-HUMAN**	-0.1528923929	0.9989736	1.452500e-02	0.999995089	-7.044767e-03	0.9996259
**DOPD-HUMAN**	-0.2152417044	0.9989736	3.894527e-02	0.999995089	-2.144156e-01	0.9996259
**PGK1-HUMAN**	0.0296371199	0.9989736	1.055654e-01	0.999995089	-4.147435e-02	0.9996259
**HV335-HUMAN**	0.1097486486	0.9989736	8.208320e-02	0.999995089	5.253100e-02	0.9996259
**CERU-HUMAN**	0.1458524654	0.9989736	-2.927111e-02	0.999995089	-1.498133e-02	0.9996259
**UB2Q2-HUMAN**	-0.0962888026	0.9989736	-1.572991e-01	0.999995089	7.439145e-02	0.9996259
**H2A1-HUMAN;H2A2C-HUMAN;H2A2A-HUMAN;H2A1C-HUM**	0.2114594473	0.9989736	-4.325975e-03	0.999995089	5.483808e-02	0.9996259
**APOH-HUMAN**	0.1167699368	0.9989736	1.195874e-02	0.999995089	-1.147236e-01	0.9996259
**S10A8-HUMAN**	0.1946190642	0.9989736	1.949784e-02	0.999995089	1.414712e-01	0.9996259
**PRPS1-HUMAN**	-0.0978444889	0.9989736	1.513393e-01	0.999995089	-4.343656e-02	0.9996259
**41-HUMAN**	0.1150376464	0.9989736	-7.352677e-02	0.999995089	4.073608e-02	0.9996259
**IMB1-HUMAN**	0.1297826930	0.9989736	-2.268611e-02	0.999995089	-2.285635e-02	0.9996259
**RINI-HUMAN**	-0.0691716488	0.9989736	-3.846273e-02	0.999995089	-6.777749e-02	0.9996259
**6PGD-HUMAN**	-0.0205874112	0.9989736	1.308134e-01	0.999995089	-1.658346e-02	0.9996259
**APOE-HUMAN**	-0.0351051525	0.9989736	-1.148011e-01	0.999995089	-2.835372e-01	0.9996259
**AXA2L-HUMAN;ANXA2-HUMAN**	-0.0422952228	0.9989736	-1.114311e-01	0.999995089	3.293656e-02	0.9996259
**BIEA-HUMAN**	-0.1110590517	0.9989736	-3.481020e-02	0.999995089	-1.110132e-01	0.9996259
**MIF-HUMAN**	-0.0296550363	0.9989736	-2.339011e-01	0.999995089	4.916649e-03	0.9996259
**HPRT-HUMAN**	-0.1408074146	0.9989736	-3.374936e-02	0.999995089	-2.750742e-01	0.9996259
**THRB-HUMAN**	-0.1155098154	0.9989736	1.183372e-02	0.999995089	1.758664e-01	0.9996259
**TGM2-HUMAN**	0.0364855810	0.9989736	-1.812876e-01	0.999995089	-1.734031e-01	0.9996259
**RD23A-HUMAN**	-0.0640995543	0.9989736	7.427906e-03	0.999995089	-4.223559e-02	0.9996259
**HV373-HUMAN**	-0.1372220552	0.9989736	4.422843e-02	0.999995089	3.446642e-02	0.9996259
**PPAC-HUMAN**	0.2479213475	0.9989736	4.832112e-02	0.999995089	4.617590e-02	0.9996259
**AFAM-HUMAN**	-0.1322883623	0.9989736	7.465464e-02	0.999995089	3.473894e-01	0.9996259
**TPM4-HUMAN**	-0.0837605498	0.9989736	1.429711e-02	0.999995089	2.817498e-02	0.9996259
**KVD24-HUMAN;KV224-HUMAN**	-0.0318711792	0.9989736	1.999404e-02	0.999995089	-5.370693e-03	0.9996259
**UBE2N-HUMAN**	-0.1344323369	0.9989736	-7.879542e-02	0.999995089	-8.616327e-02	0.9996259
**F10A1-HUMAN**	0.0279709901	0.9989736	-5.670236e-02	0.999995089	-1.741697e-01	0.9996259
**LGUL-HUMAN**	-0.4117511181	0.9989736	7.985251e-02	0.999995089	-4.433465e-02	0.9996259
**STOM-HUMAN**	0.1989968644	0.9989736	9.131282e-02	0.999995089	-4.479086e-02	0.9996259

**S10A9-HUMAN**	0.1786085157	0.9989736	4.647567e-02	0.999995089	4.243184e-02	0.9996259
**CXCL7-HUMAN**	-0.0885836833	0.9989736	-1.168799e-01	0.999995089	1.492524e-01	0.9996259
**KV230-HUMAN**	-0.1595067489	0.9989736	4.767374e-02	0.999995089	4.184703e-02	0.9996259
**AIDA-HUMAN**	0.1989011593	0.9989736	9.623641e-04	0.999995089	-4.084669e-02	0.9996259
**LV147-HUMAN**	-0.1243813191	0.9989736	5.359273e-03	0.999995089	9.021883e-04	0.9996259
**CD5L-HUMAN**	-0.1411384299	0.9989736	1.979592e-02	0.999995089	3.130898e-01	0.9996259
**GLO2-HUMAN**	-0.3021274358	0.9989736	-2.684474e-02	0.999995089	4.208596e-02	0.9996259
**HBAT-HUMAN**	-0.2439356470	0.9989736	-3.744704e-02	0.999995089	2.052404e-01	0.9996259
**APOC2-HUMAN**	-0.2482191664	0.9989736	-2.663285e-03	0.999995089	2.191907e-02	0.9996259
**COF1-HUMAN**	0.0100770287	0.9989736	-1.094364e-01	0.999995089	-5.677435e-02	0.9996259
**SAA4-HUMAN**	0.0909875286	0.9989736	-8.302921e-02	0.999995089	2.981002e-02	0.9996259
**GLRX1-HUMAN**	-0.0786792975	0.9989736	7.513418e-02	0.999995089	1.021366e-02	0.9996259
**TALDO-HUMAN**	-0.0917404063	0.9989736	6.247906e-02	0.999995089	-5.993712e-02	0.9996259
**CAN1-HUMAN**	-0.1212934576	0.9989736	6.494025e-02	0.999995089	-1.185815e-01	0.9996259
**APOM-HUMAN**	-0.0276815367	0.9989736	-1.345181e-01	0.999995089	2.500282e-01	0.9996259
**CAH3-HUMAN**	0.0903028735	0.9989736	8.544432e-02	0.999995089	-6.325933e-02	0.9996259
**APOD-HUMAN**	0.0515065694	0.9989736	-1.522972e-01	0.999995089	2.635354e-02	0.9996259
**C1S-HUMAN**	-0.0001950440	0.9989736	-9.052763e-02	0.999995089	-3.556692e-02	0.9996259
**RS26-HUMAN;RS26L-HUMAN**	-0.0907947100	0.9989736	1.156034e-02	0.999995089	1.052825e-01	0.9996259
**HV307-HUMAN**	-0.0883322612	0.9989736	1.085599e-01	0.999995089	2.235871e-01	0.9996259
**GPX1-HUMAN**	-0.0027756487	0.9989736	-4.741209e-03	0.999995089	-1.540716e-01	0.9996259
**HV169-HUMAN**	0.1252284084	0.9989736	6.125420e-02	0.999995089	-1.245227e-01	0.9996259
**KLKB1-HUMAN**	-0.1712937031	0.9989736	-6.639841e-02	0.999995089	-3.084178e-02	0.9996259
**ARF3-HUMAN;ARF1-HUMAN**	0.0335521047	0.9989736	1.190029e-02	0.999995089	-1.122135e-01	0.9996259
**HSP7C-HUMAN**	-0.1500684221	0.9989736	8.288920e-02	0.999995089	-4.778364e-02	0.9996259
**HV374-HUMAN**	-0.0887513535	0.9989736	1.077873e-01	0.999995089	3.196998e-01	0.9996259
**1433Z-HUMAN**	-0.0577154639	0.9989736	-4.576466e-02	0.999995089	-1.184867e-01	0.9996259
**PIMT-HUMAN**	-0.1853565427	0.9989736	9.420628e-02	0.999995089	1.073096e-01	0.9996259
**ACPH-HUMAN**	-0.2171947669	0.9989736	-4.620987e-02	0.999995089	-1.931803e-01	0.9996259
**IGHG4-HUMAN**	-0.0814774698	0.9989736	1.834405e-01	0.999995089	2.390303e-01	0.9996259
**HV309-HUMAN**	0.0482711570	0.9989736	-6.297360e-02	0.999995089	-8.343139e-02	0.9996259
**NTF2-HUMAN**	0.0740491223	0.9989736	-1.351959e-01	0.999995089	-1.219211e-02	0.9996259
**DDI2-HUMAN**	-0.1641079112	0.9989736	-1.568782e-01	0.999995089	-9.156537e-02	0.9996259
**PGM2-HUMAN**	-0.1277767836	0.9989736	3.983030e-02	0.999995089	-3.511230e-02	0.9996259
**UBP5-HUMAN**	0.0817027406	0.9989736	7.882780e-04	0.999995089	-7.577101e-02	0.9996259
**PSA4-HUMAN**	-0.2239041605	0.9989736	2.268987e-01	0.999995089	6.823594e-03	0.9996259
**KVD13-HUMAN;KV113-HUMAN**	-0.1121082672	0.9989736	-1.117891e-01	0.999995089	-9.531692e-02	0.9996259
**EM55-HUMAN**	0.1805609095	0.9989736	1.130493e-01	0.999995089	-8.838476e-02	0.9996259
**ADDB-HUMAN**	0.0441344482	0.9989736	3.741630e-02	0.999995089	1.251840e-01	0.9996259
**GELS-HUMAN**	0.1393834665	0.9989736	-2.077051e-01	0.999995089	-2.967697e-02	0.9996259
**APOC1-HUMAN**	-0.0023019601	0.9989736	-1.281137e-01	0.999995089	1.231201e-01	0.9996259
**LV319-HUMAN**	0.1280506687	0.9989736	9.107876e-02	0.999995089	-2.880230e-01	0.9996259
**PROS-HUMAN**	-0.1005552042	0.9989736	-4.431419e-02	0.999995089	1.172384e-01	0.9996259
**CFAI-HUMAN**	0.0426333218	0.9989736	-6.499877e-02	0.999995089	-1.822232e-02	0.9996259
**THIO-HUMAN**	-0.1958148316	0.9989736	-8.930773e-02	0.999995089	-1.039418e-01	0.9996259
**CCS-HUMAN**	-0.3471491773	0.9989736	7.761532e-02	0.999995089	-1.224500e-01	0.9996259
**ITIH1-HUMAN**	-0.0298573785	0.9989736	6.264941e-02	0.999995089	5.784975e-02	0.9996259
**NCEH1-HUMAN**	0.0647089873	0.9989736	1.003342e-01	0.999995089	-4.436504e-02	0.9996259
**KV112-HUMAN;KVD12-HUMAN**	0.2347703201	0.9989736	-1.352077e-01	0.999995089	-1.632072e-01	0.9996259
**HV434-HUMAN**	0.2730789083	0.9989736	7.364894e-02	0.999995089	-1.695200e-01	0.9996259
**PEBP1-HUMAN**	-0.1893636242	0.9989736	1.444231e-01	0.999995089	-9.530434e-02	0.9996259
**TCPQ-HUMAN**	-0.0010908858	0.9989736	2.694410e-01	0.999995089	2.074623e-01	0.9996259
**CAND1-HUMAN**	0.1003567721	0.9989736	1.555273e-01	0.999995089	-3.039345e-02	0.9996259
**1433E-HUMAN**	0.0753517868	0.9989736	-5.553479e-02	0.999995089	-1.075901e-01	0.9996259
**A2AP-HUMAN**	-0.0660847356	0.9989736	4.693738e-02	0.999995089	8.048487e-02	0.9996259
**SORCN-HUMAN**	-0.0610441289	0.9989736	-1.774657e-01	0.999995089	-1.350390e-01	0.9996259
**SAHH-HUMAN**	-0.0464445084	0.9989736	-3.273703e-02	0.999995089	-4.233963e-02	0.9996259
**SODC-HUMAN**	0.1421611121	0.9989736	7.143195e-02	0.999995089	-5.736284e-02	0.9996259
**HARS1-HUMAN**	-0.0249840462	0.9989736	2.136649e-01	0.999995089	-9.766080e-02	0.9996259
**CPNS1-HUMAN**	-0.1287985467	0.9989736	-3.847889e-02	0.999995089	-8.524133e-02	0.9996259
**HEP2-HUMAN**	-0.1223128733	0.9989736	-5.507865e-03	0.999995089	-9.868935e-02	0.9996259
**TYB4-HUMAN**	0.0358609939	0.9989736	-8.063614e-02	0.999995089	6.597747e-02	0.9996259
**LUM-HUMAN**	0.1073225235	0.9989736	-8.991847e-02	0.999995089	-7.169896e-02	0.9996259
**CALM1-HUMAN;CALM2-HUMAN;CALM3-HUMAN**	-0.0902306307	0.9989736	1.239313e-01	0.999995089	5.722621e-02	0.9996259
**PTPRJ-HUMAN**	-0.0360921315	0.9989736	1.540004e-01	0.999995089	8.691904e-02	0.9996259
**HEM3-HUMAN**	-0.2046652391	0.9989736	-9.559893e-02	0.999995089	1.703461e-01	0.9996259
**IGHM-HUMAN**	-0.1646239528	0.9989736	4.595898e-02	0.999995089	2.706583e-01	0.9996259
**ANGT-HUMAN**	-0.0148588525	0.9989736	-1.402698e-01	0.999995089	-3.904551e-03	0.9996259
**ICAL-HUMAN**	-0.1675706038	0.9989736	-2.344154e-02	0.999995089	3.298423e-02	0.9996259
**TRFL-HUMAN**	0.2100974502	0.9989736	-5.206327e-02	0.999995089	1.718471e-01	0.9996259
**FKB1A-HUMAN**	0.0421511094	0.9989736	-2.894310e-02	0.999995089	-9.811025e-02	0.9996259
**PSMF1-HUMAN**	0.0139216613	0.9989736	3.304488e-02	0.999995089	-9.454760e-02	0.9996259

**HYAS1-HUMAN**	-0.0595473038	0.9989736	1.522878e-01	0.999995089	1.665446e-01	0.9996259
**CO5-HUMAN**	0.1680173093	0.9989736	1.624493e-02	0.999995089	5.639741e-02	0.9996259
**PTPA-HUMAN**	-0.1946796792	0.9989736	-3.954482e-02	0.999995089	-1.350885e-01	0.9996259
**HS71A-HUMAN;HS71B-HUMAN**	-0.1728178041	0.9989736	1.573072e-01	0.999995089	-1.937063e-01	0.9996259
**FHR3-HUMAN**	-0.0245678727	0.9989736	-9.747157e-02	0.999995089	-4.721561e-02	0.9996259
**APOA-HUMAN**	-0.0420222828	0.9989736	5.439553e-02	0.999995089	-2.245863e-02	0.9996259
**CO4B-HUMAN**	0.0868745324	0.9989736	5.968546e-02	0.999995089	-1.588600e-01	0.9996259
**KPYR-HUMAN**	0.2529007310	0.9989736	-1.118951e-01	0.999995089	-8.537062e-02	0.9996259
**PSME1-HUMAN**	-0.1665956740	0.9989736	1.380054e-01	0.999995089	1.819052e-03	0.9996259
**C1QA-HUMAN**	-0.0066185562	0.9989736	-7.427744e-02	0.999995089	-1.096605e-01	0.9996259
**TSP1-HUMAN**	0.0014985494	0.9989736	-1.534033e-01	0.999995089	-6.408056e-02	0.9996259
**FBX7-HUMAN**	0.0457250351	0.9989736	1.438374e-01	0.999995089	-3.728350e-03	0.9996259
**HPTR-HUMAN**	0.1651565191	0.9989736	5.139339e-02	0.999995089	-9.696921e-02	0.9996259
**ADDA-HUMAN**	-0.0565799782	0.9989736	8.616422e-02	0.999995089	-6.380468e-02	0.9996259
**PITH1-HUMAN**	0.0686120068	0.9989736	-2.708902e-02	0.999995089	-1.141602e-01	0.9996259
**LV144-HUMAN**	0.1647306860	0.9989736	-2.590991e-02	0.999995089	-3.056552e-01	0.9996259
**TCPG-HUMAN**	0.1210457114	0.9989736	2.944272e-01	0.999995089	-1.018878e-01	0.9996259
**STIP1-HUMAN**	0.0194113932	0.9989736	9.084739e-02	0.999995089	-3.027083e-01	0.9996259
**DCUP-HUMAN**	-0.4406025854	0.9989736	1.425857e-01	0.999995089	2.337726e-02	0.9996259
**AHSP-HUMAN**	-0.1591592672	0.9989736	5.870120e-02	0.999995089	1.827081e-01	0.9996259
**PSA1-HUMAN**	-0.2579680222	0.9989736	5.404502e-02	0.999995089	5.147756e-03	0.9996259
**FHR1-HUMAN**	0.0239135922	0.9989736	-5.903164e-02	0.999995089	-1.491798e-02	0.9996259
**ENOA-HUMAN**	-0.0751147982	0.9989736	3.405949e-02	0.999995089	4.878411e-02	0.9996259
**CLIC1-HUMAN**	-0.0966825326	0.9989736	3.949712e-02	0.999995089	-1.437940e-01	0.9996259
**IGJ-HUMAN**	0.1498649693	0.9989736	1.605269e-01	0.999995089	1.541806e-01	0.9996259
**BMP1-HUMAN;TLL2-HUMAN**	0.1852597667	0.9989736	-3.243174e-02	0.999995089	5.765826e-02	0.9996259
**APOL1-HUMAN**	-0.1698062215	0.9989736	-1.433744e-01	0.999995089	3.656175e-02	0.9996259
**QCR7-HUMAN**	0.1341500874	0.9989736	-9.208595e-02	0.999995089	-6.531799e-02	0.9996259
**TCPZ-HUMAN**	0.0500829918	0.9989736	1.630485e-01	0.999995089	3.120621e-02	0.9996259
**UBP14-HUMAN**	-0.2178223483	0.9989736	2.220972e-02	0.999995089	-1.414079e-01	0.9996259
**FCL-HUMAN**	-0.0838674072	0.9989736	1.408645e-01	0.999995089	-4.667219e-02	0.9996259
**2AAA-HUMAN**	0.0109417813	0.9989736	1.442863e-01	0.999995089	-5.008865e-02	0.9996259
**CLUS-HUMAN**	-0.0660116407	0.9989736	-1.460572e-01	0.999995089	9.967900e-02	0.9996259
**GSHR-HUMAN**	-0.1159156119	0.9989736	5.006272e-02	0.999995089	-2.056381e-01	0.9996259
**IF5A1-HUMAN**	0.2184808482	0.9989736	2.530275e-02	0.999995089	-1.355570e-01	0.9996259
**TCPE-HUMAN**	0.0304612425	0.9989736	1.073435e-01	0.999995089	8.033348e-02	0.9996259
**FLNA-HUMAN**	0.0153020399	0.9989736	-4.933535e-02	0.999995089	6.306951e-02	0.9996259
**HEBP1-HUMAN**	-0.3491159815	0.9989736	-2.392880e-02	0.999995089	7.051499e-02	0.9996259
**PSA7-HUMAN**	-0.1492267699	0.9989736	2.158705e-03	0.999995089	-4.740648e-03	0.9996259
**PA1B3-HUMAN**	0.0956663960	0.9989736	-4.983955e-02	0.999995089	-2.487889e-01	0.9996259
**TCPB-HUMAN**	0.0141574509	0.9989736	1.909822e-01	0.999995089	1.773130e-01	0.9996259
**SKP1-HUMAN**	-0.0285711363	0.9989736	9.977982e-02	0.999995089	-2.664629e-01	0.9996259
**LV39-HUMAN**	-0.1394855664	0.9989736	1.187238e-01	0.999995089	-2.845807e-01	0.9996259
**NDKA-HUMAN**	0.0154136509	0.9989736	1.208887e-01	0.999995089	2.936127e-03	0.9996259
**TCPH-HUMAN**	0.0377761931	0.9989736	1.913275e-01	0.999995089	1.108814e-01	0.9996259
**TXNL1-HUMAN**	-0.1800972819	0.9989736	4.812635e-02	0.999995089	2.316968e-02	0.9996259
**GSTP1-HUMAN**	-0.0836622263	0.9989736	7.760860e-02	0.999995089	-2.027205e-01	0.9996259
**HS90B-HUMAN**	-0.0226778732	0.9989736	-1.924778e-03	0.999995089	-3.623881e-02	0.9996259
**HV5X1-HUMAN**	0.0042935609	0.9989736	7.644162e-02	0.999995089	5.618437e-02	0.9996259
**RPIA-HUMAN**	-0.1031132726	0.9989736	2.499407e-02	0.999995089	2.749102e-02	0.9996259
**RET4-HUMAN**	0.0154412821	0.9989736	3.683176e-02	0.999995089	3.802006e-03	0.9996259
**BOLA2-HUMAN**	-0.2120560765	0.9989736	-1.231432e-01	0.999995089	-1.104998e-01	0.9996259
**H2B1K-HUMAN;H2B1J-HUMAN;H2B1O-HUMAN;H2B1B-HU**	0.1717973753	0.9989736	-7.674870e-02	0.999995089	5.424166e-02	0.9996259
**FA12-HUMAN**	-0.0730918027	0.9989736	-1.411414e-01	0.999995089	7.935112e-02	0.9996259
**IF4A1-HUMAN**	0.0253682941	0.9989736	2.657734e-02	0.999995089	1.801032e-01	0.9996259
**SHPS1-HUMAN;SIRBL-HUMAN;SIRPG-HUMAN**	0.1768558785	0.9989736	-8.760667e-02	0.999995089	1.759633e-01	0.9996259
**UBA1-HUMAN**	-0.0768419117	0.9989736	1.834359e-02	0.999995089	-1.149577e-01	0.9996259
**SH3L3-HUMAN**	-0.0301509019	0.9989736	-4.979790e-02	0.999995089	1.173137e-01	0.9996259
**PNCB-HUMAN**	-0.0379387035	0.9989736	-6.824006e-02	0.999995089	5.741040e-02	0.9996259
**GTR1-HUMAN**	0.2611084169	0.9989736	1.071427e-01	0.999995089	-3.257682e-02	0.9996259
**PSB2-HUMAN**	0.1890062208	0.9989736	1.277673e-01	0.999995089	-2.293854e-02	0.9996259
**RAC1-HUMAN**	0.2129810905	0.9989736	1.024761e-01	0.999995089	-2.387499e-02	0.9996259
**CO7-HUMAN**	0.2916433587	0.9989736	4.411081e-02	0.999995089	-1.433282e-01	0.9996259
**PUR6-HUMAN**	-0.1514432726	0.9989736	-4.532981e-02	0.999995089	1.474521e-01	0.9996259
**ACLY-HUMAN**	-0.1471229664	0.9989736	1.361118e-01	0.999995089	-1.131905e-01	0.9996259
**WDR1-HUMAN**	-0.0970277279	0.9989736	-2.590333e-02	0.999995089	2.780154e-02	0.9996259
**HS90A-HUMAN**	-0.1059899525	0.9989736	3.654323e-02	0.999995089	6.090765e-02	0.9996259
**CO9-HUMAN**	-0.0259378106	0.9989736	3.555754e-03	0.999995089	4.864641e-02	0.9996259
**LV310-HUMAN**	-0.0204625592	0.9989736	6.146126e-02	0.999995089	-5.860490e-02	0.9996259
**TPM3-HUMAN**	-0.0349588344	0.9989736	1.312223e-02	0.999995089	3.450717e-02	0.9996259
**PSA5-HUMAN**	-0.1548823462	0.9989736	1.289584e-01	0.999995089	3.399919e-02	0.9996259
**RPE-HUMAN**	0.1284787600	0.9989736	4.788415e-02	0.999995089	2.290310e-01	0.9996259

**PLSL-HUMAN**	0.2374966254	0.9989736	1.018736e-01	0.999995089	-1.536361e-01	0.9996259
**DAZP1-HUMAN**	0.0876916997	0.9989736	-4.364465e-02	0.999995089	2.676626e-01	0.9996259
**THTM-HUMAN**	-0.0526503604	0.9989736	1.004927e-01	0.999995089	-2.923985e-02	0.9996259
**OLA1-HUMAN**	-0.0076941876	0.9989736	5.060033e-03	0.999995089	1.156481e-02	0.9996259
**TAGL2-HUMAN**	-0.0030562160	0.9989736	4.929939e-03	0.999995089	4.899240e-03	0.9996259
**TBCA-HUMAN**	-0.0330529862	0.9989736	6.285821e-02	0.999995089	6.193704e-02	0.9996259
**PSB1-HUMAN**	-0.0682534625	0.9989736	2.553937e-01	0.999995089	-1.115853e-01	0.9996259
**CO2-HUMAN**	-0.1558513964	0.9989736	4.066860e-02	0.999995089	-5.455127e-02	0.9996259
**SUMO3-HUMAN;SUMO2-HUMAN;SUMO4-HUMAN**	0.1121862939	0.9989736	-1.348740e-01	0.999995089	1.387942e-01	0.9996259
**HV551-HUMAN**	0.0822270949	0.9989736	7.289986e-02	0.999995089	2.191466e-02	0.9996259
**PSMD9-HUMAN**	-0.0031991410	0.9989736	-1.725175e-02	0.999995089	-8.508293e-02	0.9996259
**PGRP2-HUMAN**	-0.0270446239	0.9989736	-1.329931e-01	0.999995089	7.565741e-02	0.9996259
**AMBP-HUMAN**	0.0558803568	0.9989736	3.029310e-02	0.999995089	-1.827525e-01	0.9996259
**ESTD-HUMAN**	0.1493938465	0.9989736	3.524836e-02	0.999995089	-1.276690e-01	0.9996259
**TCPD-HUMAN**	0.1975523378	0.9989736	2.495124e-01	0.999995089	-1.971501e-02	0.9996259
**ELNE-HUMAN**	0.2453612385	0.9989736	-2.800889e-03	0.999995089	9.315044e-02	0.9996259
**NSF1C-HUMAN**	-0.0775602284	0.9989736	1.597683e-01	0.999995089	-2.881049e-02	0.9996259
**TTHY-HUMAN**	-0.0240581133	0.9989736	7.609069e-02	0.999995089	3.287758e-01	0.9996259
**PUR9-HUMAN**	0.0655478325	0.9989736	2.100704e-02	0.999995089	-2.732924e-01	0.9996259
**GLOD4-HUMAN**	-0.1830138760	0.9989736	-1.829152e-01	0.999995089	4.086946e-03	0.9996259
**AL1L1-HUMAN**	-0.1122633552	0.9989736	-1.482203e-01	0.999995089	-1.544005e-01	0.9996259
**IGD-HUMAN**	0.1337661602	0.9989736	1.159477e-01	0.999995089	1.848567e-01	0.9996259
**TKT-HUMAN**	0.0056018992	0.9989736	-1.434915e-02	0.999995089	2.557621e-01	0.9996259
**TLN1-HUMAN**	0.0276651856	0.9989736	-5.383856e-02	0.999995089	-3.711469e-02	0.9996259
**CYTB-HUMAN**	-0.1417040460	0.9989736	-2.314802e-01	0.999995089	-1.147205e-01	0.9996259
**LIMS1-HUMAN**	-0.0865465755	0.9989736	7.571790e-03	0.999995089	8.524864e-02	0.9996259
**ENOG-HUMAN**	-0.1181730116	0.9989736	1.810906e-01	0.999995089	1.640604e-01	0.9996259
**PRTN3-HUMAN**	0.2838633052	0.9989736	6.614653e-02	0.999995089	1.283401e-02	0.9996259
**A2GL-HUMAN**	0.1883028974	0.9989736	-2.180438e-01	0.999995089	-1.340850e-01	0.9996259
**AATC-HUMAN**	-0.1242866969	0.9989736	2.985420e-02	0.999995089	9.113583e-02	0.9996259
**GSH1-HUMAN**	0.1621228793	0.9989736	2.965137e-02	0.999995089	5.342014e-02	0.9996259
**HBE-HUMAN**	-0.0286127661	0.9989736	3.211235e-02	0.999995089	-3.021109e-01	0.9996259
**LV325-HUMAN**	0.0286156940	0.9989736	-8.307928e-02	0.999995089	5.879386e-02	0.9996259
**HINT1-HUMAN**	-0.1726047512	0.9989736	1.530233e-02	0.999995089	9.470141e-02	0.9996259
**KVD15-HUMAN**	0.2409730643	0.9989736	-3.012501e-01	0.999995089	-9.169863e-03	0.9996259
**CO6-HUMAN**	0.0835224329	0.9989736	-9.477601e-02	0.999995089	-1.142768e-01	0.9996259
**SH3L1-HUMAN**	0.0288785078	0.9989736	7.096783e-02	0.999995089	2.025547e-01	0.9996259
**HV330-HUMAN;HVC05-HUMAN**	-0.0160410976	0.9989736	3.173421e-02	0.999995089	-6.543230e-03	0.9996259
**CO8A-HUMAN**	0.0480315539	0.9989736	2.703132e-03	0.999995089	-2.787335e-02	0.9996259
**URP2-HUMAN**	-0.0042720386	0.9989736	5.534958e-02	0.999995089	-2.997527e-01	0.9996259
**HV601-HUMAN**	-0.0153490271	0.9989736	8.195907e-02	0.999995089	-5.275768e-02	0.9996259
**UBAC1-HUMAN**	0.0953119836	0.9989736	5.045459e-02	0.999995089	8.181486e-02	0.9996259
**LV151-HUMAN**	-0.0944819260	0.9989736	2.653462e-02	0.999995089	-1.443390e-01	0.9996259
**HEM4-HUMAN**	0.2257062837	0.9989736	-6.339408e-02	0.999995089	1.206252e-01	0.9996259
**PSB4-HUMAN**	-0.1408310069	0.9989736	2.066947e-01	0.999995089	-8.437583e-02	0.9996259
**TEBP-HUMAN**	0.0719384564	0.9989736	-7.408473e-02	0.999995089	1.297008e-01	0.9996259
**PRS4-HUMAN**	0.0822010479	0.9989736	1.519789e-01	0.999995089	-6.510415e-02	0.9996259
**ZA2G-HUMAN**	-0.0277943086	0.9989736	-8.497793e-03	0.999995089	-1.048541e-01	0.9996259
**HV70D-HUMAN**	-0.0626627437	0.9989736	5.705999e-02	0.999995089	6.726064e-03	0.9996259
**MOES-HUMAN**	0.1470644577	0.9989736	-1.286042e-02	0.999995089	-6.877914e-02	0.9996259
**APMAP-HUMAN**	0.1853609697	0.9989736	-6.844881e-02	0.999995089	8.796264e-02	0.9996259
**SNAA-HUMAN**	-0.1040676726	0.9989736	1.883160e-01	0.999995089	2.837233e-01	0.9996259
**FLOT1-HUMAN**	0.2607427078	0.9989736	1.159596e-01	0.999995089	-1.920814e-01	0.9996259
**PSA6-HUMAN**	-0.1396074413	0.9989736	1.082544e-01	0.999995089	3.407194e-02	0.9996259
**CUTA-HUMAN**	0.1175270677	0.9989736	-1.283247e-02	0.999995089	-3.977099e-01	0.9996259
**PSA3-HUMAN**	-0.0085802816	0.9989736	1.521598e-01	0.999995089	5.968732e-02	0.9996259
**UB2D3-HUMAN;UB2D2-HUMAN**	-0.0190268938	0.9989736	1.117787e-01	0.999995089	-2.305305e-02	0.9996259
**ANM5-HUMAN**	0.1606777339	0.9989736	1.532580e-01	0.999995089	9.774918e-02	0.9996259
**LV743-HUMAN**	-0.1640623898	0.9989736	3.899878e-02	0.999995089	1.020829e-01	0.9996259
**ITA2B-HUMAN**	0.0055504763	0.9989736	-2.612769e-02	0.999995089	-4.600325e-02	0.9996259
**SEPP1-HUMAN**	-0.0031004055	0.9989736	-2.685898e-02	0.999995089	7.463677e-02	0.9996259
**CATG-HUMAN**	0.3272526903	0.9989736	7.878421e-03	0.999995089	1.087451e-01	0.9996259
**UB2L3-HUMAN**	0.0652878527	0.9989736	1.392779e-01	0.999995089	-1.141793e-01	0.9996259
**LKHA4-HUMAN**	-0.0828741760	0.9989736	-1.175587e-02	0.999995089	-2.329460e-01	0.9996259
**CO8G-HUMAN**	-0.0712050196	0.9989736	-3.990610e-02	0.999995089	1.204814e-02	0.9996259
**LXN-HUMAN**	0.0237883953	0.9989736	-1.303919e-01	0.999995089	-6.709243e-02	0.9996259
**H4-HUMAN**	0.2330914978	0.9989736	7.173199e-02	0.999995089	4.356304e-02	0.9996259
**TCPA-HUMAN**	0.1881634990	0.9989736	2.149525e-01	0.999995089	1.061002e-01	0.9996259
**PERM-HUMAN**	0.3014309670	0.9989736	-2.531049e-02	0.999995089	7.272681e-02	0.9996259
**DPP3-HUMAN**	-0.1024455255	0.9989736	1.002003e-01	0.999995089	-4.832814e-02	0.9996259
**IDHC-HUMAN**	-0.0336207906	0.9989736	-1.167458e-01	0.999995089	4.785929e-02	0.9996259
**MCTS1-HUMAN**	0.1786710958	0.9989736	3.777740e-02	0.999995089	-6.416742e-02	0.9996259

**RAP1A-HUMAN**	0.1564265322	0.9989736	4.331219e-02	0.999995089	-8.603267e-02	0.9996259
**HV432-HUMAN**	-0.1200000429	0.9989736	5.373199e-02	0.999995089	-1.482644e-01	0.9996259
**CHSP1-HUMAN**	0.0268766231	0.9989736	1.451840e-01	0.999995089	-6.877311e-03	0.9996259
**HBM-HUMAN**	-0.1842163978	0.9989736	1.442428e-01	0.999995089	-1.037654e-01	0.9996259
**K2C6A-HUMAN**	0.2067602024	0.9989736	-7.376095e-02	0.999995089	2.022118e-02	0.9996259
**CAZA1-HUMAN**	0.0279468101	0.9989736	3.728372e-02	0.999995089	1.205488e-01	0.9996259
**ANXA7-HUMAN**	0.0373693207	0.9989736	1.110783e-01	0.999995089	2.048690e-01	0.9996259
**HV338-HUMAN**	-0.0289594373	0.9989736	6.661655e-02	0.999995089	2.494338e-01	0.9996259
**G6PD-HUMAN**	-0.0547485879	0.9989736	3.247562e-01	0.999995089	7.015683e-02	0.9996259
**G6PI-HUMAN**	0.0830780817	0.9989736	-1.427241e-01	0.999995089	-1.861913e-01	0.9996259
**OTU1-HUMAN**	-0.3280873285	0.9989736	3.587377e-02	0.999995089	1.055274e-01	0.9996259
**DNPH1-HUMAN**	-0.1111879959	0.9989736	-9.739214e-02	0.999995089	6.378859e-02	0.9996259
**OXSR1-HUMAN**	0.0577304257	0.9989736	1.490898e-01	0.999995089	-1.140866e-03	0.9996259
**PSME2-HUMAN**	-0.2180056213	0.9989736	-9.716729e-02	0.999995089	-1.013453e-01	0.9996259
**KV139-HUMAN;KVD39-HUMAN**	0.1489879978	0.9989736	1.321980e-02	0.999995089	-5.353267e-02	0.9996259
**RHOA-HUMAN**	0.2018944876	0.9989736	6.486694e-02	0.999995089	-3.143817e-02	0.9996259
**MYL6-HUMAN**	0.0589996607	0.9989736	-6.000381e-02	0.999995089	8.277452e-02	0.9996259
**CPN2-HUMAN**	-0.1287282705	0.9989736	-2.114099e-01	0.999995089	-1.260707e-02	0.9996259
**PSB6-HUMAN**	-0.1582330682	0.9989736	5.445963e-02	0.999995089	-2.467312e-03	0.9996259
**GSHB-HUMAN**	-0.2196614829	0.9989736	-1.721591e-01	0.999995089	-4.565092e-02	0.9996259
**FAAA-HUMAN**	0.0056756482	0.9989736	-2.416070e-01	0.999995089	-3.960250e-01	0.9996259
**UBE2K-HUMAN**	-0.0762476602	0.9989736	-8.340797e-02	0.999995089	1.117722e-01	0.9996259
**MYH9-HUMAN**	-0.1052756562	0.9989736	4.377510e-02	0.999995089	1.334360e-01	0.9996259
**PI42A-HUMAN**	0.0588134813	0.9989736	5.032248e-02	0.999995089	3.840171e-03	0.9996259
**C1RL-HUMAN**	-0.0283618607	0.9989736	-1.529498e-01	0.999995089	-3.167327e-03	0.9996259
**RANG-HUMAN**	-0.1641254278	0.9989736	1.629525e-01	0.999995089	-5.386674e-02	0.9996259
**THIC-HUMAN**	-0.0837303268	0.9989736	-7.430724e-02	0.999995089	-4.140389e-02	0.9996259
**DCXR-HUMAN**	-0.2117723814	0.9989736	8.761485e-02	0.999995089	1.737370e-01	0.9996259
**PA1B2-HUMAN**	0.0250425636	0.9989736	4.144032e-02	0.999995089	-1.893544e-01	0.9996259
**KPYM-HUMAN**	0.0951904457	0.9989736	-4.556500e-02	0.999995089	1.080784e-01	0.9996259
**HABP2-HUMAN**	-0.0575862039	0.9989736	-2.674184e-02	0.999995089	1.095082e-01	0.9996259
**UBE2O-HUMAN**	-0.3101326851	0.9989736	3.674385e-02	0.999995089	2.022050e-03	0.9996259
**VINC-HUMAN**	-0.0055603241	0.9989736	-1.238145e-01	0.999995089	1.842903e-02	0.9996259
**PP2AA-HUMAN**	0.1054903022	0.9989736	2.024372e-01	0.999995089	-2.517171e-01	0.9996259
**ALDOC-HUMAN**	-0.0542391533	0.9989736	-1.318844e-02	0.999995089	-1.750619e-01	0.9996259
**PRPS2-HUMAN**	0.2288625265	0.9989736	-9.620632e-02	0.999995089	1.882228e-01	0.9996259
**PON1-HUMAN**	-0.1370465736	0.9989736	6.892441e-03	0.999995089	-1.394635e-01	0.9996259
**HGFL-HUMAN**	0.1686344217	0.9989736	6.420036e-02	0.999995089	1.556248e-01	0.9996259
**GPX3-HUMAN**	-0.0346036459	0.9989736	5.616494e-02	0.999995089	1.651511e-01	0.9996259
**RLA2-HUMAN**	0.2164174085	0.9989736	4.736243e-02	0.999995089	6.479790e-02	0.9996259
**PSB5-HUMAN**	0.0382345636	0.9989736	1.997962e-01	0.999995089	-3.222410e-02	0.9996259
**H14-HUMAN**	0.1066304478	0.9989736	-1.508445e-01	0.999995089	1.016932e-01	0.9996259
**CO8B-HUMAN**	0.0536380630	0.9989736	8.071922e-02	0.999995089	7.403919e-02	0.9996259
**6PGL-HUMAN**	0.0022212232	0.9989736	-6.664369e-02	0.999995089	-6.304315e-02	0.9996259
**CBPB2-HUMAN**	0.0496393620	0.9989736	-4.660887e-02	0.999995089	-4.978617e-02	0.9996259
**HV64D-HUMAN**	0.1561213750	0.9989736	1.327730e-02	0.999995089	2.700555e-02	0.9996259
**TETN-HUMAN**	0.0066098497	0.9989736	-1.620075e-01	0.999995089	-8.278336e-02	0.9996259
**GLPC-HUMAN**	0.2293134264	0.9989736	2.510598e-02	0.999995089	-6.106291e-02	0.9996259
**PEDF-HUMAN**	-0.0880256793	0.9989736	3.348310e-02	0.999995089	-1.243160e-01	0.9996259
**H31-HUMAN;H31T-HUMAN;H32-HUMAN**	0.2258643160	0.9989736	5.502747e-02	0.999995089	6.539575e-02	0.9996259
**UBP15-HUMAN**	-0.2800189032	0.9989736	2.003430e-02	0.999995089	2.521719e-02	0.9996259
**GSH0-HUMAN**	-0.0739046023	0.9989736	-5.583440e-02	0.999995089	3.132129e-02	0.9996259
**PRS7-HUMAN**	0.0026690152	0.9989736	1.634561e-01	0.999995089	-2.799488e-01	0.9996259
**PGBM-HUMAN**	-0.0726366009	0.9989736	2.796281e-01	0.999995089	-6.096231e-02	0.9996259
**PGAM1-HUMAN**	-0.1056035890	0.9989736	6.775007e-02	0.999995089	-7.681297e-02	0.9996259
**CSN4-HUMAN**	-0.2032293208	0.9989736	2.068515e-01	0.999995089	1.637979e-01	0.9996259
**PSB7-HUMAN**	-0.1554084629	0.9989736	1.639869e-01	0.999995089	1.065752e-01	0.9996259
**NP1L4-HUMAN**	0.2458340556	0.9989736	8.247800e-02	0.999995089	2.580735e-02	0.9996259
**KV108-HUMAN**	0.1612732142	0.9989736	1.779759e-01	0.999995089	8.351769e-03	0.9996259
**FN3K-HUMAN**	0.1422217132	0.9989736	-1.370182e-01	0.999995089	-1.745525e-01	0.9996259
**HV313-HUMAN**	-0.0898787171	0.9989736	1.259705e-01	0.999995089	-8.405117e-02	0.9996259
**ACTN1-HUMAN**	0.0757765915	0.9989736	-7.514820e-04	0.999995089	3.314880e-02	0.9996259
**HV404-HUMAN**	0.1818048414	0.9989736	1.053904e-01	0.999995089	1.285459e-01	0.9996259
**RAB1B-HUMAN**	0.0596881304	0.9989736	1.661418e-01	0.999995089	1.071066e-02	0.9996259
**ANXA3-HUMAN**	-0.1890111542	0.9989736	-2.062885e-02	0.999995089	3.836690e-02	0.9996259
**AP2S1-HUMAN**	0.0246063926	0.9989736	-2.009103e-01	0.999995089	1.004785e-01	0.9996259
**LYSC-HUMAN**	0.1804679579	0.9989736	-6.989333e-02	0.999995089	-3.466828e-02	0.9996259
**CAP1-HUMAN**	0.0801506297	0.9989736	-5.457438e-02	0.999995089	-1.305854e-01	0.9996259
**XPO7-HUMAN**	-0.0113475140	0.9989736	-9.899885e-02	0.999995089	1.117133e-01	0.9996259
**GMPR1-HUMAN**	-0.2512790154	0.9989736	-1.142972e-01	0.999995089	-9.498699e-02	0.9996259
**THBG-HUMAN**	0.0320073299	0.9989736	-1.496759e-01	0.999995089	-6.294836e-02	0.9996259
**NLTP-HUMAN**	0.0648596227	0.9989736	1.227140e-01	0.999995089	1.877507e-01	0.9996259

PEPD-HUMAN	-0.0847113269	0.9989736	-2.804423e-01	0.999995089	9.631975e-02	0.9996259
RN123-HUMAN	0.0773891067	0.9989736	1.172505e-01	0.999995089	9.074804e-02	0.9996259
RAB5B-HUMAN	0.0209944713	0.9989736	9.830456e-02	0.999995089	1.572277e-01	0.9996259
KCY-HUMAN	0.2842311075	0.9989736	1.526153e-01	0.999995089	-4.858618e-02	0.9996259
CBPN-HUMAN	-0.1193677803	0.9989736	-2.396967e-01	0.999995089	-6.516739e-02	0.9996259
PRDX5-HUMAN	0.0662408789	0.9989736	7.199227e-03	0.999995089	-8.102464e-02	0.9996259
TNG2-HUMAN	-0.0507771942	0.9989736	1.412653e-01	0.999995089	1.693693e-02	0.9996259
ABHEB-HUMAN	-0.0513004283	0.9989736	-4.624140e-02	0.999995089	-1.858239e-01	0.9996259
PA2G4-HUMAN	-0.0166752316	0.9989736	-1.744223e-02	0.999995089	-2.589825e-02	0.9996259
CD47-HUMAN	0.2238369363	0.9989736	9.643545e-02	0.999995089	-3.949584e-03	0.9996259
TMOD1-HUMAN	-0.1166922386	0.9989736	1.124002e-01	0.999995089	-1.426656e-01	0.9996259
TBA1B-HUMAN	-0.0806050377	0.9989736	2.439899e-02	0.999995089	-1.151305e-01	0.9996259
C1R-HUMAN	0.0211998756	0.9989736	-1.367970e-01	0.999995089	1.185578e-02	0.9996259
KVD33-HUMAN;KV133-HUMAN	0.0057213430	0.9989736	1.389250e-01	0.999995089	-8.049787e-02	0.9996259
TBB4B-HUMAN	-0.0813309887	0.9989736	-5.024638e-02	0.999995089	-5.302430e-02	0.9996259
PRS6A-HUMAN	-0.0532306879	0.9989736	7.659611e-02	0.999995089	-9.932038e-03	0.9996259
ARPC5-HUMAN	0.0280197660	0.9989736	-1.093861e-01	0.999995089	8.332051e-02	0.9996259
ETFA-HUMAN	-0.1664709931	0.9989736	3.868004e-02	0.999995089	-5.017691e-02	0.9996259
UCHL3-HUMAN	-0.0988381900	0.9989736	3.238188e-02	0.999995089	-2.439171e-01	0.9996259
APT-HUMAN	-0.0091931676	0.9989736	1.012755e-01	0.999995089	1.997858e-01	0.9996259
FA10-HUMAN	-0.2447574134	0.9989736	-6.069616e-02	0.999995089	8.677989e-02	0.9996259
PCBP1-HUMAN	0.0324565957	0.9989736	9.782394e-03	0.999995089	1.646859e-01	0.9996259
F13A-HUMAN	0.0265638038	0.9989736	-1.739593e-01	0.999995089	3.815745e-02	0.9996259
DHPR-HUMAN	0.1585359722	0.9989736	1.182169e-01	0.999995089	4.491360e-02	0.9996259
ITIH3-HUMAN	0.2143813484	0.9989736	-4.789589e-02	0.999995089	-6.166178e-02	0.9996259
WBP2-HUMAN	0.0724303197	0.9989736	1.739718e-01	0.999995089	2.138827e-01	0.9996259
ARGI1-HUMAN	-0.2567327300	0.9989736	5.162354e-02	0.999995089	-3.316855e-01	0.9996259
IF6-HUMAN	-0.0942003568	0.9989736	-1.565080e-02	0.999995089	-1.271380e-01	0.9996259
TPP2-HUMAN	-0.1214092490	0.9989736	4.150768e-02	0.999995089	8.184750e-03	0.9996259
PSMD5-HUMAN	0.1615466284	0.9989736	1.298648e-01	0.999995089	-1.817771e-01	0.9996259
ARK72-HUMAN	-0.2709077151	0.9989736	1.420675e-01	0.999995089	-1.349163e-01	0.9996259
ML12B-HUMAN;ML12A-HUMAN	-0.0795559411	0.9989736	-2.220863e-02	0.999995089	-8.264628e-03	0.9996259
GGCT-HUMAN	-0.2407758345	0.9989736	-6.136757e-02	0.999995089	1.733306e-01	0.9996259
GET3-HUMAN	-0.1357312065	0.9989736	2.697675e-02	0.999995089	9.146261e-02	0.9996259
CBR1-HUMAN	0.1118384207	0.9989736	2.142489e-01	0.999995089	1.600306e-02	0.9996259
PUR4-HUMAN	-0.0529020723	0.9989736	2.696068e-01	0.999995089	6.395353e-02	0.9996259
SAMP-HUMAN	-0.1949896303	0.9989736	-1.036585e-01	0.999995089	-8.235078e-02	0.9996259
HV372-HUMAN	0.2251275378	0.9989736	1.049617e-01	0.999995089	-2.106028e-02	0.9996259
CSN1-HUMAN	0.0013549470	0.9989736	-9.971884e-02	0.999995089	1.036944e-02	0.9996259
FHR2-HUMAN	0.1278036776	0.9989736	-7.102872e-02	0.999995089	-5.172914e-02	0.9996259
TPM1-HUMAN	-0.0294863778	0.9989736	-9.309992e-02	0.999995089	3.918488e-02	0.9996259
PFKAL-HUMAN	-0.3468028315	0.9989736	-4.356021e-02	0.999995089	4.112162e-02	0.9996259
HSP74-HUMAN	-0.1496275667	0.9989736	2.066705e-01	0.999995089	-3.067622e-02	0.9996259
KPRB-HUMAN	0.0366597876	0.9989736	1.043119e-01	0.999995089	-1.727076e-02	0.9996259
FA9-HUMAN	-0.0671444859	0.9989736	-3.843507e-02	0.999995089	3.630026e-01	0.9996259
PGP-HUMAN	-0.2088367521	0.9989736	-9.083133e-03	0.999995089	3.868248e-02	0.9996259
KRT83-HUMAN	0.0648504989	0.9989736	5.047509e-02	0.999995089	8.937080e-02	0.9996259
TENA-HUMAN	0.0968902809	0.9989736	1.567359e-01	0.999995089	-1.161172e-02	0.9996259
PUR8-HUMAN	-0.0319832815	0.9989736	1.971825e-02	0.999995089	3.283927e-03	0.9996259
CT027-HUMAN	0.0230109614	0.9989736	1.655709e-02	0.999995089	-6.431194e-02	0.9996259
S14L4-HUMAN	0.0739428674	0.9989736	-1.031032e-01	0.999995089	2.981087e-02	0.9996259
CBG-HUMAN	0.0353124151	0.9989736	-7.862551e-02	0.999995089	2.084026e-02	0.9996259
CYBR1-HUMAN	0.2357992985	0.9989736	7.573342e-02	0.999995089	-2.426226e-03	0.9996259
LEG3-HUMAN	-0.0359048889	0.9989736	3.837066e-02	0.999995089	-1.506144e-01	0.9996259
DCNL1-HUMAN	0.2676048289	0.9989736	-6.496259e-02	0.999995089	-2.561467e-01	0.9996259
ICLN-HUMAN	0.1280431665	0.9989736	-1.290711e-01	0.999995089	-1.124725e-01	0.9996259
1433G-HUMAN	-0.0059753552	0.9989736	-6.990145e-02	0.999995089	-1.100858e-01	0.9996259
RD23B-HUMAN	-0.1895882486	0.9989736	7.720707e-02	0.999995089	-3.139259e-02	0.9996259
FHR4-HUMAN	-0.1090020492	0.9989736	-2.662241e-01	0.999995089	-7.738182e-02	0.9996259
FLOT2-HUMAN	0.2064843671	0.9989736	8.814227e-02	0.999995089	-4.585033e-02	0.9996259
KV137-HUMAN;KVD37-HUMAN	-0.1118783757	0.9989736	4.914676e-02	0.999995089	6.547899e-04	0.9996259
FCN3-HUMAN	-0.1572646604	0.9989736	-4.276339e-02	0.999995089	-1.464333e-01	0.9996259
UB2V2-HUMAN	0.0932353091	0.9989736	4.610397e-02	0.999995089	-1.107024e-01	0.9996259
1433T-HUMAN	0.0243011604	0.9989736	-7.060350e-02	0.999995089	1.560077e-02	0.9996259
RB11A-HUMAN;RB11B-HUMAN	0.0313481685	0.9989736	9.129746e-03	0.999995089	2.806395e-02	0.9996259
TBA1A-HUMAN	0.1409306677	0.9989736	1.915330e-01	0.999995089	-2.255083e-01	0.9996259
LIS1-HUMAN	-0.1603674148	0.9989736	-1.545750e-01	0.999995089	-1.115237e-01	0.9996259
AL9A1-HUMAN	0.0544699724	0.9989736	2.595798e-02	0.999995089	-8.980643e-02	0.9996259
RUVB2-HUMAN	-0.0507549552	0.9989736	-9.535338e-02	0.999995089	3.521735e-02	0.9996259
TTL12-HUMAN	0.0617276661	0.9989736	9.057661e-02	0.999995089	5.199820e-03	0.9996259
PPME1-HUMAN	-0.1863675141	0.9989736	1.497171e-01	0.999995089	-7.852247e-02	0.9996259
SIA10-HUMAN	0.1279748636	0.9989736	1.080449e-01	0.999995089	9.546079e-02	0.9996259

**CLH1-HUMAN**	0.0171863322	0.9989736	-7.307841e-02	0.999995089	8.392375e-02	0.9996259
**PCBP2-HUMAN**	0.0202040721	0.9989736	-1.011815e-01	0.999995089	1.323370e-01	0.9996259
**ACTZ-HUMAN**	0.0941404373	0.9989736	2.095172e-01	0.999995089	-3.507178e-01	0.9996259
**MTAP-HUMAN**	0.1666903903	0.9989736	-1.060381e-01	0.999995089	-6.667148e-02	0.9996259
**PP1R7-HUMAN**	0.2577163611	0.9989736	-1.329303e-02	0.999995089	-1.653817e-01	0.9996259
**HV102-HUMAN**	0.0295023858	0.9989736	1.083346e-01	0.999995089	-1.050081e-01	0.9996259
**NSF-HUMAN**	0.1722616608	0.9989736	1.229027e-01	0.999995089	3.428807e-01	0.9996259
**DESP-HUMAN**	0.0922574387	0.9989736	2.495247e-03	0.999995089	1.901145e-01	0.9996259
**KAIN-HUMAN**	0.0267779192	0.9989736	-1.141690e-01	0.999995089	2.389321e-01	0.9996259
**FETUB-HUMAN**	-0.0189708155	0.9989736	-1.121265e-01	0.999995089	-3.861922e-02	0.9996259
**TBA4A-HUMAN**	0.0348819876	0.9989736	-1.533044e-01	0.999995089	2.263036e-02	0.9996259
**PERE-HUMAN**	0.3608306208	0.9989736	4.404411e-02	0.999995089	1.429364e-01	0.9996259
**H15-HUMAN**	0.1040898279	0.9989736	1.406843e-02	0.999995089	1.105456e-01	0.9996259
**VATA-HUMAN**	-0.1670834333	0.9989736	9.730948e-02	0.999995089	-7.873373e-02	0.9996259
**GDIR2-HUMAN**	0.0793634543	0.9989736	1.174533e-01	0.999995089	1.153091e-01	0.9996259
**VCAM1-HUMAN**	-0.1700541314	0.9989736	1.153482e-01	0.999995089	-1.211765e-01	0.9996259
**A16A1-HUMAN**	-0.2333186644	0.9989736	4.489455e-02	0.999995089	1.170685e-01	0.9996259
**PTGDS-HUMAN**	-0.0529817177	0.9989736	1.710225e-01	0.999995089	-6.330986e-02	0.9996259
**PSB3-HUMAN**	-0.0699658867	0.9989736	2.578666e-01	0.999995089	3.159607e-02	0.9996259
**UGPA-HUMAN**	-0.1568890711	0.9989736	-8.624844e-02	0.999995089	-9.757000e-02	0.9996259
**PLEK-HUMAN**	0.0440501612	0.9989736	-2.713720e-02	0.999995089	-1.379052e-01	0.9996259
**PSA2-HUMAN**	-0.0141333752	0.9989736	2.753098e-01	0.999995089	-1.569965e-01	0.9996259
**HV428-HUMAN**	0.2682999225	0.9989736	1.725871e-01	0.999995089	-1.424900e-01	0.9996259
**IPO9-HUMAN**	0.0443844452	0.9989736	1.351284e-01	0.999995089	-1.879754e-01	0.9996259
**GLRX3-HUMAN**	0.0704580448	0.9989736	1.463751e-01	0.999995089	2.620164e-02	0.9996259
**TXD17-HUMAN**	-0.0516377742	0.9989736	5.656692e-02	0.999995089	-1.456962e-01	0.9996259
**CD59-HUMAN**	0.1960835351	0.9989736	1.206391e-01	0.999995089	-1.210315e-02	0.9996259
**FSCN1-HUMAN**	-0.0689371243	0.9989736	-1.970536e-01	0.999995089	-2.757626e-02	0.9996259
**RIDA-HUMAN**	-0.1519441821	0.9989736	-2.783765e-02	0.999995089	-1.425504e-01	0.9996259
**LANC1-HUMAN**	0.1247943905	0.9989736	1.794043e-01	0.999995089	-2.641347e-01	0.9996259
**RHCE-HUMAN**	0.2934625308	0.9989736	1.043303e-01	0.999995089	-7.744899e-02	0.9996259
**PDC6I-HUMAN**	-0.1356412061	0.9989736	-5.692939e-02	0.999995089	1.402054e-02	0.9996259
**KJ01-HUMAN**	0.2606283735	0.9989736	3.750527e-02	0.999995089	2.133006e-01	0.9996259
**FBLN1-HUMAN**	0.3356833943	0.9989736	-1.269967e-01	0.999995089	-1.141144e-01	0.9996259
**HV364-HUMAN**	0.0051155450	0.9989736	1.210815e-01	0.999995089	-1.864761e-01	0.9996259
**PSDE-HUMAN**	-0.3165590664	0.9989736	1.476201e-01	0.999995089	5.841329e-02	0.9996259
**VWF-HUMAN**	0.0339259566	0.9989736	-6.764031e-03	0.999995089	-2.323409e-02	0.9996259
**CAPZB-HUMAN**	0.0033497165	0.9989736	-2.779976e-02	0.999995089	-2.021693e-01	0.9996259
**PHLD-HUMAN**	-0.1613233404	0.9989736	9.869079e-02	0.999995089	1.996869e-01	0.9996259
**PPIL3-HUMAN**	0.1326847301	0.9989736	6.087972e-02	0.999995089	2.238742e-02	0.9996259
**KV117-HUMAN**	0.0118546614	0.9989736	2.538775e-02	0.999995089	6.930438e-02	0.9996259
**RASK-HUMAN**	0.1161589937	0.9989736	1.049424e-01	0.999995089	-1.037842e-01	0.9996259
**LV208-HUMAN**	-0.1718321683	0.9989736	1.680931e-01	0.999995089	-4.159976e-02	0.9996259
**IF4B-HUMAN**	-0.0893325353	0.9989736	-2.305626e-02	0.999995089	1.368622e-02	0.9996259
**PGM2L-HUMAN**	0.1837947801	0.9989736	8.306156e-02	0.999995089	-2.663226e-01	0.9996259
**2AAB-HUMAN**	-0.0335760274	0.9989736	-5.024370e-02	0.999995089	-7.831035e-02	0.9996259
**PRS10-HUMAN**	-0.0647960279	0.9989736	1.499820e-01	0.999995089	4.643968e-02	0.9996259
**DYN2-HUMAN**	-0.0933228504	0.9989736	-8.509629e-02	0.999995089	1.046477e-01	0.9996259
**CYB5-HUMAN**	0.0278370051	0.9989736	-1.392042e-02	0.999995089	-1.428699e-01	0.9996259
**GSTM3-HUMAN**	-0.2463347638	0.9989736	9.248734e-02	0.999995089	-7.133599e-02	0.9996259
**NB5R3-HUMAN**	-0.0418174761	0.9989736	-1.544588e-02	0.999995089	-1.883687e-02	0.9996259
**PROF1-HUMAN**	0.0530414433	0.9989736	-3.311146e-03	0.999995089	4.029488e-02	0.9996259
**HV124-HUMAN**	0.1550539157	0.9989736	-2.814262e-01	0.999995089	3.641722e-02	0.9996259
**ADT2-HUMAN;ADT1-HUMAN;ADT3-HUMAN;ADT4-HUMAN**	0.1026992191	0.9989736	1.189177e-01	0.999995089	-3.826283e-02	0.9996259
**LV949-HUMAN**	-0.1326154776	0.9989736	1.027504e-01	0.999995089	1.467710e-01	0.9996259
**ANXA1-HUMAN**	0.0954078112	0.9989736	-8.778482e-02	0.999995089	5.298148e-02	0.9996259
**IF2G-HUMAN**	0.0421428771	0.9989736	7.441181e-02	0.999995089	2.096334e-01	0.9996259
**BROX-HUMAN**	-0.0693045164	0.9989736	2.015901e-01	0.999995089	1.504840e-01	0.9996259
**CNRP1-HUMAN**	0.1329379973	0.9989736	-6.250702e-06	0.999995089	-3.131195e-02	0.9996259
**MGST2-HUMAN**	0.0316761093	0.9989736	5.020312e-02	0.999995089	5.150375e-02	0.9996259
**PRG2-HUMAN**	0.0563079994	0.9989736	4.942136e-02	0.999995089	2.620453e-01	0.9996259
**FABP5-HUMAN**	0.0156059144	0.9989736	9.935462e-02	0.999995089	5.499774e-02	0.9996259
**IPYR-HUMAN**	-0.0947249263	0.9989736	1.460469e-01	0.999995089	-2.220534e-02	0.9996259
**KVD16-HUMAN**	0.2114159942	0.9989736	1.012881e-01	0.999995089	2.241919e-01	0.9996259
**MISSL-HUMAN**	0.2106133255	0.9989736	-1.959743e-02	0.999995089	-1.152457e-01	0.9996259
**CUL3-HUMAN**	-0.1799169782	0.9989736	-4.171184e-02	0.999995089	-2.383274e-01	0.9996259
**DDB1-HUMAN**	-0.0176800947	0.9989736	6.468928e-02	0.999995089	-1.636943e-01	0.9996259
**NGAL-HUMAN**	0.2023150548	0.9989736	2.071420e-02	0.999995089	-4.726717e-02	0.9996259
**RAB10-HUMAN**	0.2356631657	0.9989736	9.417607e-02	0.999995089	-8.414614e-02	0.9996259
**IMA4-HUMAN**	-0.1528358599	0.9989736	-1.716778e-01	0.999995089	1.149023e-01	0.9996259
**LV211-HUMAN**	-0.0622499269	0.9989736	4.600051e-02	0.999995089	-1.244172e-01	0.9996259
**IGF2-HUMAN**	-0.0424444751	0.9989736	4.376322e-02	0.999995089	1.427544e-01	0.9996259

**RAP2B-HUMAN**	0.2250157793	0.9989736	5.942097e-02	0.999995089	-6.707804e-02	0.9996259
**PSMD6-HUMAN**	-0.0047118079	0.9989736	5.234965e-02	0.999995089	4.944526e-02	0.9996259
**TBB5-HUMAN**	0.0300349651	0.9989736	-2.526206e-02	0.999995089	-2.122021e-01	0.9996259
**RAB35-HUMAN**	0.2837245568	0.9989736	-2.350447e-01	0.999995089	-1.661692e-01	0.9996259
**KV240-HUMAN;KVD40-HUMAN**	0.1874090607	0.9989736	1.358651e-01	0.999995089	7.336187e-02	0.9996259
**LASP1-HUMAN**	0.0691665988	0.9989736	1.098074e-01	0.999995089	1.504171e-01	0.9996259
**PSMD1-HUMAN**	-0.0475621494	0.9989736	6.095476e-02	0.999995089	4.398108e-03	0.9996259
**CI040-HUMAN**	0.0997293423	0.9989736	3.924642e-02	0.999995089	-3.560898e-02	0.9996259
**MIEN1-HUMAN**	-0.2129609846	0.9989736	2.058189e-01	0.999995089	-1.680028e-01	0.9996259
**KVD08-HUMAN**	0.3040257218	0.9989736	7.783574e-02	0.999995089	1.263689e-01	0.9996259
**TBB1-HUMAN**	-0.0216344977	0.9989736	-4.100705e-02	0.999995089	-3.533256e-02	0.9996259
**EHD1-HUMAN**	0.0108773086	0.9989736	2.153272e-03	0.999995089	9.617951e-02	0.9996259
**MEP50-HUMAN**	0.0862988388	0.9989736	-3.269808e-02	0.999995089	-1.219958e-02	0.9996259
**PRS8-HUMAN**	-0.1251383601	0.9989736	1.444115e-01	0.999995089	-4.265768e-02	0.9996259
**HV343-HUMAN**	0.2999865182	0.9989736	4.759073e-02	0.999995089	-1.462128e-01	0.9996259
**COR1A-HUMAN**	0.1984460034	0.9989736	-8.135744e-02	0.999995089	3.116230e-02	0.9996259
**GBB1-HUMAN**	0.0287316233	0.9989736	1.603416e-02	0.999995089	7.198577e-03	0.9996259
**RSU1-HUMAN**	0.0588530717	0.9989736	-1.800331e-02	0.999995089	1.740642e-01	0.9996259
**UFC1-HUMAN**	-0.0808817511	0.9989736	-2.479931e-02	0.999995089	-2.072557e-01	0.9996259
**FA5-HUMAN**	0.1059795831	0.9989736	-4.915219e-02	0.999995089	1.397093e-03	0.9996259
**SRXN1-HUMAN**	-0.0851233839	0.9989736	2.606773e-01	0.999995089	8.056378e-02	0.9996259
**ABRAL-HUMAN**	0.0664354269	0.9989736	2.968680e-02	0.999995089	-2.380627e-01	0.9996259
**FPPS-HUMAN**	-0.2805097840	0.9989736	1.805744e-01	0.999995089	1.323699e-01	0.9996259
**PDCD6-HUMAN**	0.1075106581	0.9989736	-2.282605e-01	0.999995089	1.960610e-01	0.9996259
**STAM1-HUMAN**	0.1363813444	0.9989736	1.326543e-01	0.999995089	-2.478978e-01	0.9996259
**C1TC-HUMAN**	0.0296184070	0.9989736	4.684401e-02	0.999995089	-7.047622e-02	0.9996259
**PSD10-HUMAN**	-0.1267438083	0.9989736	-9.971844e-02	0.999995089	-1.199574e-01	0.9996259
**GRHPR-HUMAN**	-0.0875770770	0.9989736	-1.325566e-01	0.999995089	2.333423e-02	0.9996259
**PSMD4-HUMAN**	-0.1198272165	0.9989736	1.390168e-01	0.999995089	-1.652300e-01	0.9996259
**MK01-HUMAN**	-0.1625741520	0.9989736	7.514102e-02	0.999995089	3.466994e-01	0.9996259
**SYDC-HUMAN**	0.1034829887	0.9989736	9.728317e-02	0.999995089	1.897350e-01	0.9996259
**C4BPB-HUMAN**	-0.0537188313	0.9989736	-3.244015e-02	0.999995089	-1.508277e-02	0.9996259
**MASP1-HUMAN**	-0.1451024438	0.9989736	-1.027420e-01	0.999995089	-1.304335e-01	0.9996259
**CRKL-HUMAN**	0.1169569296	0.9989736	2.069202e-01	0.999995089	2.863517e-02	0.9996259
**GDIR1-HUMAN**	-0.0091306413	0.9989736	9.797537e-02	0.999995089	1.119630e-01	0.9996259
**LV140-HUMAN**	0.0163499294	0.9989736	2.303423e-01	0.999995089	-1.545082e-01	0.9996259
**CDC42-HUMAN**	0.2675034352	0.9989736	1.037522e-01	0.999995089	-1.199003e-01	0.9996259
**HNRPD-HUMAN**	-0.2396845849	0.9989736	4.046216e-02	0.999995089	-1.401428e-01	0.9996259
**ATRN-HUMAN**	0.0846092701	0.9989736	-1.800498e-01	0.999995089	1.094065e-01	0.9996259
**UBA5-HUMAN**	-0.0461612992	0.9989736	7.998597e-02	0.999995089	-9.543138e-02	0.9996259
**GAPD1-HUMAN**	-0.1009448400	0.9989736	9.029860e-02	0.999995089	-3.944544e-03	0.9996259
**PFKAM-HUMAN**	-0.3293980099	0.9989736	7.627322e-02	0.999995089	-1.874583e-01	0.9996259
**RLA0-HUMAN;RLA0L-HUMAN**	0.2089872785	0.9989736	1.666386e-01	0.999995089	1.701055e-01	0.9996259
**CMBL-HUMAN**	-0.0717564139	0.9989736	9.806553e-02	0.999995089	2.814639e-01	0.9996259
**OTUB1-HUMAN**	0.1014202397	0.9989736	1.036072e-01	0.999995089	-3.116428e-02	0.9996259
**NUDC2-HUMAN**	-0.0388550356	0.9989736	2.505282e-02	0.999995089	1.939922e-01	0.9996259
**CSN7B-HUMAN**	-0.0794362392	0.9989736	-2.359800e-01	0.999995089	-1.032570e-02	0.9996259
**PROP-HUMAN**	-0.0823968484	0.9989736	9.441760e-07	0.999995089	7.068765e-02	0.9996259
**KV105-HUMAN**	0.1301073978	0.9989736	9.647788e-02	0.999995089	-8.725502e-02	0.9996259
**MYL4-HUMAN**	-0.2167686084	0.9989736	1.360789e-01	0.999995089	3.671835e-02	0.9996259
**GDIA-HUMAN**	-0.0316787306	0.9989736	1.532815e-01	0.999995089	-3.531216e-02	0.9996259
**HV349-HUMAN**	-0.1060292517	0.9989736	5.151457e-02	0.999995089	8.193658e-02	0.9996259
**ADHX-HUMAN**	0.0578824340	0.9989736	1.509613e-01	0.999995089	-3.159269e-01	0.9996259
**CZIB-HUMAN**	-0.4533108165	0.9989736	1.049583e-01	0.999995089	-1.151695e-01	0.9996259
**CISD2-HUMAN**	0.0902650181	0.9989736	3.807242e-02	0.999995089	-1.567997e-01	0.9996259
**KAP0-HUMAN**	0.0242328091	0.9989736	2.778130e-02	0.999995089	4.966824e-02	0.9996259
**FUMH-HUMAN**	-0.0233001924	0.9989736	2.090266e-01	0.999995089	2.398634e-02	0.9996259
**F13B-HUMAN**	0.0099640602	0.9989736	-4.538898e-02	0.999995089	1.027055e-01	0.9996259
**AP1B1-HUMAN**	-0.2154599399	0.9989736	-2.661351e-01	0.999995089	-6.223178e-02	0.9996259
**LBP-HUMAN**	0.2144951211	0.9989736	-9.390878e-02	0.999995089	-1.145999e-01	0.9996259
**EF1A1-HUMAN;EF1A3-HUMAN**	-0.0181468276	0.9989736	-9.108696e-02	0.999995089	8.904777e-03	0.9996259
**RAB6A-HUMAN**	-0.1174448794	0.9989736	7.747257e-02	0.999995089	3.786486e-02	0.9996259
**FBLN3-HUMAN**	0.2690527853	0.9989736	-1.720783e-01	0.999995089	-7.532749e-02	0.9996259
**CD14-HUMAN**	0.0447244771	0.9989736	-1.986680e-01	0.999995089	-1.937058e-01	0.9996259
**VIME-HUMAN**	0.0434279468	0.9989736	9.414155e-03	0.999995089	-2.618814e-02	0.9996259
**AGO2-HUMAN**	-0.0237446796	0.9989736	1.295836e-03	0.999995089	1.270155e-01	0.9996259
**S10AC-HUMAN**	0.2579411675	0.9989736	1.097743e-01	0.999995089	4.739673e-02	0.9996259
**PLPP-HUMAN**	-0.1157324677	0.9989736	4.407291e-02	0.999995089	1.295822e-01	0.9996259
**PSMD7-HUMAN**	0.1062290690	0.9989736	1.657992e-01	0.999995089	-1.986507e-01	0.9996259
**IPO7-HUMAN**	-0.2330441302	0.9989736	5.972208e-02	0.999995089	1.260898e-01	0.9996259
**HNRPK-HUMAN**	0.0606898497	0.9989736	2.621470e-02	0.999995089	-7.359861e-02	0.9996259
**VATB2-HUMAN**	-0.0548199629	0.9989736	1.289451e-01	0.999995089	4.670681e-02	0.9996259

**RFESD-HUMAN**	0.0635702033	0.9989736	6.462410e-02	0.999995089	1.331741e-01	0.9996259
**NIF3L-HUMAN**	-0.0305456007	0.9989736	-1.521792e-01	0.999995089	-1.421448e-01	0.9996259
**KT3K-HUMAN**	-0.0655798905	0.9989736	-2.356760e-01	0.999995089	-8.431500e-02	0.9996259
**NEDD8-HUMAN**	-0.0704606122	0.9989736	6.513055e-02	0.999995089	1.148243e-01	0.9996259
**ERMAP-HUMAN**	0.2207425834	0.9989736	8.735132e-02	0.999995089	-1.793767e-02	0.9996259
**TKFC-HUMAN**	-0.2236394022	0.9989736	1.895391e-02	0.999995089	1.362012e-01	0.9996259
**DYL2-HUMAN**	-0.0248910967	0.9989736	2.794982e-02	0.999995089	-1.225110e-01	0.9996259
**ATG3-HUMAN**	0.0446617493	0.9989736	1.156759e-01	0.999995089	2.515930e-02	0.9996259
**ACYP1-HUMAN**	-0.2017277168	0.9989736	4.089433e-02	0.999995089	5.329699e-02	0.9996259
**AMPL-HUMAN**	-0.0155540088	0.9989736	1.314480e-01	0.999995089	1.400717e-01	0.9996259
**LV218-HUMAN**	0.0203096551	0.9989736	-2.629295e-04	0.999995089	-3.627194e-02	0.9996259
**PSD13-HUMAN**	-0.2129410609	0.9989736	6.192040e-02	0.999995089	-1.082248e-01	0.9996259
**GP1BB-HUMAN**	0.1154804812	0.9989736	8.722265e-02	0.999995089	1.208245e-02	0.9996259
**HGFA-HUMAN**	0.3423676651	0.9989736	1.614202e-02	0.999995089	9.583842e-02	0.9996259
**AT2B4-HUMAN**	0.2312104548	0.9989736	9.197634e-02	0.999995089	-3.251365e-02	0.9996259
**GP1BA-HUMAN**	0.1217104494	0.9989736	2.624962e-03	0.999995089	-2.094926e-01	0.9996259
**SMIM1-HUMAN**	0.2402044275	0.9989736	9.530614e-02	0.999995089	-3.910563e-02	0.9996259
**THOP1-HUMAN**	-0.1398479266	0.9989736	-1.697956e-01	0.999995089	4.791708e-02	0.9996259
**GSTT1-HUMAN**	-0.2327991080	0.9989736	9.888938e-02	0.999995089	-1.158241e-01	0.9996259
**ANXA5-HUMAN**	0.1538977876	0.9989736	-1.697022e-01	0.999995089	-2.623664e-02	0.9996259
**QSOX1-HUMAN**	0.1171290206	0.9989736	-3.230131e-03	0.999995089	7.109419e-05	0.9996259
**ITB3-HUMAN**	0.1572276444	0.9989736	7.609936e-02	0.999995089	-3.287620e-02	0.9996259
**CARM1-HUMAN**	-0.0866129220	0.9989736	7.618638e-02	0.999995089	-8.587646e-02	0.9996259
**LV214-HUMAN**	-0.2854613638	0.9989736	1.037210e-01	0.999995089	-7.180798e-02	0.9996259
**RAB18-HUMAN**	-0.1011616212	0.9989736	3.758196e-02	0.999995089	-1.515528e-01	0.9996259
**CSN3-HUMAN**	-0.1533834660	0.9989736	9.590960e-02	0.999995089	1.247236e-01	0.9996259
**CD9-HUMAN**	0.1298545263	0.9989736	8.678173e-02	0.999995089	-7.637233e-03	0.9996259
**LV136-HUMAN**	0.3091331606	0.9989736	-5.666069e-02	0.999995089	-8.269445e-02	0.9996259
**DNJB2-HUMAN**	0.0592518159	0.9989736	9.889714e-02	0.999995089	1.702884e-01	0.9996259
**ECM1-HUMAN**	-0.0281464850	0.9989736	-9.299772e-02	0.999995089	-7.238174e-02	0.9996259
**OSCAR-HUMAN**	-0.2671947480	0.9989736	1.500874e-01	0.999995089	-3.327210e-02	0.9996259
**PDIA6-HUMAN**	0.2078353011	0.9989736	-4.703259e-02	0.999995089	5.944431e-02	0.9996259
**HV146-HUMAN**	0.2344118135	0.9989736	9.288182e-02	0.999995089	-1.724898e-01	0.9996259
**PPCS-HUMAN**	0.0189663444	0.9989736	-9.290736e-02	0.999995089	2.862797e-02	0.9996259
**ALS-HUMAN**	-0.1921853883	0.9989736	-6.164703e-02	0.999995089	3.187296e-01	0.9996259
**SHBG-HUMAN**	0.2584577455	0.9989736	8.520353e-03	0.999995089	-1.075588e-01	0.9996259
**PLTP-HUMAN**	0.1375751373	0.9989736	6.455898e-03	0.999995089	9.350556e-02	0.9996259
**SYWC-HUMAN**	-0.1407980883	0.9989736	1.719251e-02	0.999995089	1.152316e-01	0.9996259
**GAPR1-HUMAN**	0.2527893874	0.9989736	1.187723e-01	0.999995089	-4.121074e-02	0.9996259
**ARPC2-HUMAN**	0.0481922253	0.9989736	-1.541696e-01	0.999995089	1.461844e-01	0.9996259
**KTHY-HUMAN**	-0.1013989724	0.9989736	-2.672700e-02	0.999995089	1.606219e-01	0.9996259
**DNJB1-HUMAN**	-0.1827199881	0.9989736	-3.228163e-02	0.999995089	-2.019068e-01	0.9996259
**CD44-HUMAN**	0.2618643895	0.9989736	5.052834e-02	0.999995089	-7.194153e-02	0.9996259
**LV657-HUMAN**	-0.1259358766	0.9989736	3.055656e-03	0.999995089	-6.695351e-02	0.9996259
**CAMP-HUMAN**	0.1987667554	0.9989736	-6.960201e-02	0.999995089	1.037854e-01	0.9996259
**CDK2-HUMAN**	0.0331616717	0.9989736	4.227079e-02	0.999995089	-2.146929e-02	0.9996259
**AMPN-HUMAN**	0.2309187260	0.9989736	6.486616e-02	0.999995089	-1.428826e-01	0.9996259
**HV205-HUMAN**	-0.0501440442	0.9989736	6.088110e-02	0.999995089	8.846403e-03	0.9996259
**CSN6-HUMAN**	-0.0840996355	0.9989736	6.854475e-02	0.999995089	6.497944e-02	0.9996259
**GNPI1-HUMAN**	-0.1272356681	0.9989736	-2.157575e-01	0.999995089	2.628970e-02	0.9996259
**IF2A-HUMAN**	-0.0421246988	0.9989736	1.117792e-01	0.999995089	2.336436e-01	0.9996259
**UBR4-HUMAN**	-0.1338913567	0.9989736	1.681207e-01	0.999995089	-3.673893e-02	0.9996259
**IPO5-HUMAN**	0.1031346840	0.9989736	8.358223e-02	0.999995089	7.973135e-02	0.9996259
**H13-HUMAN**	0.1516546616	0.9989736	1.785199e-02	0.999995089	-9.830155e-03	0.9996259
**BPI-HUMAN**	0.1863145897	0.9989736	9.620961e-02	0.999995089	1.619934e-01	0.9996259
**ILEU-HUMAN**	0.0333345652	0.9989736	2.735213e-02	0.999995089	-2.772379e-02	0.9996259
**PSMD2-HUMAN**	0.0079840957	0.9989736	1.059920e-01	0.999995089	1.018443e-01	0.9996259
**HV145-HUMAN**	-0.1316480623	0.9989736	7.950684e-02	0.999995089	2.791980e-02	0.9996259
**MET7A-HUMAN**	0.1969383794	0.9989736	1.105447e-01	0.999995089	-6.930987e-02	0.9996259
**AP2M1-HUMAN**	0.0088928438	0.9989736	-1.158532e-01	0.999995089	2.539296e-02	0.9996259
**AP2A1-HUMAN**	-0.0970710883	0.9989736	-1.167836e-01	0.999995089	-2.034725e-02	0.9996259
**TYB10-HUMAN**	0.0891897041	0.9989736	-2.303136e-02	0.999995089	-4.786465e-02	0.9996259
**LYPA1-HUMAN**	0.0013365464	0.9989736	-1.116861e-01	0.999995089	-1.186442e-01	0.9996259
**PSD11-HUMAN**	-0.1887278959	0.9989736	4.747523e-02	0.999995089	2.966515e-02	0.9996259
**RUVB1-HUMAN**	0.0591104269	0.9989736	-1.027995e-01	0.999995089	-9.537392e-02	0.9996259
**GMFG-HUMAN**	0.0313664302	0.9989736	-9.692526e-02	0.999995089	-2.161763e-01	0.9996259
**IDE-HUMAN**	-0.0372918207	0.9989736	1.271248e-01	0.999995089	9.458103e-02	0.9996259
**RS10-HUMAN**	0.1529229215	0.9989736	5.599871e-02	0.999995089	-1.248136e-01	0.9996259
**AK1A1-HUMAN**	-0.0114699977	0.9989736	3.275045e-02	0.999995089	-7.052621e-02	0.9996259
**RAP1B-HUMAN**	0.1784075473	0.9989736	4.444940e-02	0.999995089	1.606480e-02	0.9996259
**CYTC-HUMAN**	0.0651256757	0.9989736	5.211282e-02	0.999995089	-2.367072e-01	0.9996259
**CRLF3-HUMAN**	-0.2125245950	0.9989736	3.180325e-02	0.999995089	1.119229e-01	0.9996259

**CRP-HUMAN**	0.1728177770	0.9989736	-5.475664e-02	0.999995089	-2.222121e-01	0.9996259
**PPIB-HUMAN**	0.2035157533	0.9989736	3.814593e-02	0.999995089	8.491847e-02	0.9996259
**IF4E-HUMAN**	-0.0403671129	0.9989736	1.760360e-01	0.999995089	-3.932223e-02	0.9996259
**ARP2-HUMAN**	0.0375112085	0.9989736	8.603849e-02	0.999995089	6.638122e-02	0.9996259
**MARE1-HUMAN**	0.1801657189	0.9989736	-5.502693e-02	0.999995089	2.341544e-02	0.9996259
**APOC4-HUMAN**	-0.0981630399	0.9989736	5.304757e-02	0.999995089	2.933367e-01	0.9996259
**APOF-HUMAN**	0.1843810648	0.9989736	4.229957e-02	0.999995089	1.179159e-02	0.9996259
**KV116-HUMAN**	-0.2374979060	0.9989736	1.009881e-01	0.999995089	7.470267e-03	0.9996259
**PYRG1-HUMAN**	-0.3620322999	0.9989736	9.087242e-02	0.999995089	6.936057e-03	0.9996259
**EIF3J-HUMAN**	0.1338719105	0.9989736	3.018989e-02	0.999995089	-3.214804e-02	0.9996259
**EF2-HUMAN**	-0.0176287741	0.9989736	1.488743e-01	0.999995089	1.819962e-01	0.9996259
**ZYX-HUMAN**	-0.0462165471	0.9989736	-1.438091e-01	0.999995089	2.673982e-02	0.9996259
**CSN8-HUMAN**	0.3935724812	0.9989736	2.473140e-02	0.999995089	9.032153e-02	0.9996259
**HBAZ-HUMAN**	0.0796755476	0.9989736	-1.520527e-02	0.999995089	-1.716421e-01	0.9996259
**AP2B1-HUMAN**	0.1167488583	0.9989736	-1.689415e-01	0.999995089	-1.534326e-01	0.9996259
**RTN3-HUMAN**	-0.3136444043	0.9989736	5.830782e-02	0.999995089	9.727732e-02	0.9996259
**PDE12-HUMAN**	-0.1394923768	0.9989736	-3.374532e-02	0.999995089	-1.402894e-02	0.9996259
**LV469-HUMAN**	0.0952773462	0.9989736	-2.275347e-01	0.999995089	-2.835905e-01	0.9996259
**NUDT5-HUMAN**	-0.0568552851	0.9989736	3.575518e-03	0.999995089	-1.185333e-01	0.9996259
**1433F-HUMAN**	-0.0942710342	0.9989736	-9.072606e-02	0.999995089	1.024880e-01	0.9996259
**PSD12-HUMAN**	-0.0804076554	0.9989736	1.258505e-01	0.999995089	-6.969112e-02	0.9996259
**GMPR2-HUMAN**	-0.1814715198	0.9989736	-6.782859e-02	0.999995089	8.007904e-02	0.9996259
**XPO1-HUMAN**	-0.1437889731	0.9989736	1.072634e-01	0.999995089	5.753888e-02	0.9996259
**PSMG3-HUMAN**	-0.1035608502	0.9989736	-8.601928e-02	0.999995089	4.769265e-02	0.9996259
**PZP-HUMAN**	-0.1708888644	0.9989736	9.668376e-02	0.999995089	1.796705e-01	0.9996259
**LV321-HUMAN**	-0.3149982444	0.9989736	1.172382e-01	0.999995089	-2.829570e-01	0.9996259
**DCTN2-HUMAN**	-0.0918437354	0.9989736	1.773144e-01	0.999995089	-1.140547e-01	0.9996259
**ANFY1-HUMAN**	-0.1283148334	0.9989736	8.694564e-02	0.999995089	-1.019682e-01	0.9996259
**BID-HUMAN**	-0.0435687087	0.9989736	3.104019e-02	0.999995089	1.100182e-01	0.9996259
**PDIA3-HUMAN**	0.2145902355	0.9989736	1.657935e-02	0.999995089	-1.695840e-02	0.9996259
**TRML1-HUMAN**	0.1474290675	0.9989736	7.565544e-02	0.999995089	-2.445802e-01	0.9996259
**ENPL-HUMAN**	0.2840832647	0.9989736	2.884895e-02	0.999995089	-1.133092e-01	0.9996259
**BGH3-HUMAN**	0.0808209440	0.9989736	-2.474615e-03	0.999995089	-1.681506e-01	0.9996259
**CNDP2-HUMAN**	0.1123113593	0.9989736	2.024707e-01	0.999995089	-1.026392e-01	0.9996259
**PSA-HUMAN**	-0.0516237048	0.9989736	5.170280e-02	0.999995089	-1.873672e-01	0.9996259
**CLCA-HUMAN**	-0.0936126560	0.9989736	-1.677751e-01	0.999995089	7.880444e-02	0.9996259
**ADIPO-HUMAN**	-0.0319675578	0.9989736	-1.256402e-01	0.999995089	-3.465982e-02	0.9996259
**LV223-HUMAN**	0.0658748577	0.9989736	-2.564776e-01	0.999995089	-1.323265e-01	0.9996259
**P5CR3-HUMAN**	0.1595851702	0.9989736	-2.559730e-02	0.999995089	9.475843e-02	0.9996259
**PIPNA-HUMAN**	-0.0608273999	0.9989736	8.490030e-02	0.999995089	-1.362843e-01	0.9996259
**DBNL-HUMAN**	-0.1220886674	0.9989736	5.374223e-02	0.999995089	1.253141e-01	0.9996259
**RTCA-HUMAN**	-0.0791282466	0.9989736	-4.480519e-02	0.999995089	-4.022662e-02	0.9996259
**CFAD-HUMAN**	0.0935582078	0.9989736	4.392187e-03	0.999995089	-1.893930e-01	0.9996259
**UBXN1-HUMAN**	-0.2320389939	0.9989736	-2.356064e-02	0.999995089	5.987284e-02	0.9996259
**DNJC9-HUMAN**	0.1450381583	0.9989736	1.571500e-01	0.999995089	1.144034e-01	0.9996259
**PP1G-HUMAN;PP1A-HUMAN;PP1B-HUMAN**	-0.0187151603	0.9989736	-1.194014e-01	0.999995089	-8.747298e-03	0.9996259
**MNDA-HUMAN**	-0.0518057445	0.9989736	3.741830e-02	0.999995089	-9.398307e-02	0.9996259
**HSPB1-HUMAN**	0.0969012504	0.9989736	1.795379e-02	0.999995089	-2.267453e-01	0.9996259
**CUL1-HUMAN**	0.0156343079	0.9989736	1.572909e-01	0.999995089	5.421180e-02	0.9996259
**AK1C3-HUMAN**	0.0270470595	0.9989736	1.000980e-01	0.999995089	-1.232505e-01	0.9996259
**CHLE-HUMAN**	-0.1108947000	0.9989736	-8.363186e-02	0.999995089	-1.890552e-01	0.9996259
**GRAN-HUMAN**	0.1853655964	0.9989736	-5.390470e-02	0.999995089	-5.468078e-02	0.9996259
**GMPPB-HUMAN**	-0.3801111626	0.9989736	3.664572e-02	0.999995089	-1.000438e-01	0.9996259
**VPS4B-HUMAN;FIGL1-HUMAN;VPS4A-HUMAN**	0.2282396029	0.9989736	2.073307e-01	0.999995089	-1.867152e-01	0.9996259
**RHG01-HUMAN**	-0.0550093025	0.9989736	-3.373335e-02	0.999995089	-1.706824e-01	0.9996259
**KELL-HUMAN**	0.2308131944	0.9989736	9.305568e-02	0.999995089	2.861113e-04	0.9996259
**ACES-HUMAN**	0.0855901394	0.9989736	-9.920565e-02	0.999995089	-1.485887e-02	0.9996259
**PHP14-HUMAN**	-0.1339806281	0.9989736	-1.664932e-01	0.999995089	-3.424865e-03	0.9996259
**CAZA2-HUMAN**	-0.1437904217	0.9989736	1.926348e-02	0.999995089	-4.685059e-02	0.9996259
**CAVN2-HUMAN**	-0.2795126845	0.9989736	-4.858828e-02	0.999995089	1.186021e-01	0.9996259
**ORN-HUMAN**	-0.0326994403	0.9989736	3.772545e-03	0.999995089	1.558854e-01	0.9996259
**CAP7-HUMAN**	0.0862166133	0.9989736	2.173259e-02	0.999995089	1.424773e-01	0.9996259
**IAH1-HUMAN**	-0.3274035544	0.9989736	-1.391131e-01	0.999995089	-3.735130e-02	0.9996259
**UT1-HUMAN**	0.2445608642	0.9989736	9.481697e-02	0.999995089	-9.338786e-03	0.9996259
**KGUA-HUMAN**	-0.1335570447	0.9989736	5.944760e-02	0.999995089	-7.679113e-02	0.9996259
**ICAM2-HUMAN**	-0.0680637068	0.9989736	9.339887e-02	0.999995089	-4.612912e-02	0.9996259
**PHS2-HUMAN**	0.0844472963	0.9989736	-3.121575e-02	0.999995089	1.081043e-02	0.9996259
**PRG3-HUMAN**	0.0370876615	0.9989736	1.003316e-01	0.999995089	1.375993e-01	0.9996259
**CPIN1-HUMAN**	0.0436197225	0.9989736	-7.579393e-02	0.999995089	1.060446e-02	0.9996259
**SGTA-HUMAN**	0.1508231784	0.9989736	-3.806342e-02	0.999995089	1.487048e-02	0.9996259
**SGT1-HUMAN**	0.0038926513	0.9989736	6.928032e-02	0.999995089	1.924582e-01	0.9996259
**ARPC3-HUMAN**	0.1279477288	0.9989736	9.534410e-02	0.999995089	1.913331e-01	0.9996259

ATPB-HUMAN	0.1492781990	0.9989736	-3.965186e-02	0.999995089	-1.442524e-01	0.9996259
LRC20-HUMAN	-0.2454534659	0.9989736	-1.912096e-01	0.999995089	8.725721e-02	0.9996259
IF4A2-HUMAN	-0.0650928766	0.9989736	1.423067e-01	0.999995089	1.175187e-01	0.9996259
STX7-HUMAN	0.1794789598	0.9989736	8.623552e-02	0.999995089	-7.535998e-02	0.9996259
DSG2-HUMAN	-0.1306561803	0.9989736	7.783876e-02	0.999995089	-4.110844e-02	0.9996259
RAGP1-HUMAN	-0.0578011234	0.9989736	8.975402e-02	0.999995089	-3.163114e-03	0.9996259
IBP6-HUMAN	0.2316853373	0.9989736	-2.608840e-02	0.999995089	-3.124127e-01	0.9996259
IPSP-HUMAN	-0.1416237830	0.9989736	1.397016e-03	0.999995089	-2.398013e-02	0.9996259
KV621-HUMAN	-0.2445937224	0.9989736	2.589172e-02	0.999995089	-9.895427e-02	0.9996259
CDC37-HUMAN	0.0199742055	0.9989736	6.484998e-02	0.999995089	5.354457e-02	0.9996259
PRS6B-HUMAN	-0.1343200700	0.9989736	8.332560e-02	0.999995089	6.943094e-02	0.9996259
BLMH-HUMAN	0.0429653013	0.9989736	7.723529e-02	0.999995089	1.709556e-02	0.9996259
PLIN3-HUMAN	0.0599790753	0.9989736	2.572374e-01	0.999995089	1.715255e-01	0.9996259
ARPC4-HUMAN	0.0429450489	0.9989736	-2.396735e-02	0.999995089	-1.330787e-01	0.9996259
NIT1-HUMAN	0.0146798953	0.9989736	-1.337210e-01	0.999995089	-9.671272e-02	0.9996259
FA11-HUMAN	-0.0137420471	0.9989736	-8.072322e-02	0.999995089	1.219787e-01	0.9996259
NQO2-HUMAN	-0.0269720860	0.9989736	6.108577e-02	0.999995089	1.756855e-02	0.9996259
PIN1-HUMAN	-0.1446171563	0.9989736	5.192266e-02	0.999995089	9.751443e-02	0.9996259
ENDD1-HUMAN	0.1908472659	0.9989736	5.715719e-02	0.999995089	-5.007556e-02	0.9996259
XPO2-HUMAN	0.1178088252	0.9989736	3.996470e-02	0.999995089	-4.660443e-02	0.9996259
VATG1-HUMAN	-0.0582074933	0.9989736	3.804071e-01	0.999995089	6.862126e-02	0.9996259
GMDS-HUMAN	-0.1137805545	0.9989736	5.347797e-02	0.999995089	1.057651e-01	0.9996259
RBX1-HUMAN	-0.1705845265	0.9989736	2.235593e-02	0.999995089	-1.381259e-01	0.9996259
TSN-HUMAN	0.0765454883	0.9989736	4.594751e-02	0.999995089	6.185218e-02	0.9996259
KAPCA-HUMAN	0.0461226347	0.9989736	-2.041420e-01	0.999995089	1.258355e-02	0.9996259
PSMD3-HUMAN	0.0937284806	0.9989736	1.157406e-01	0.999995089	1.273474e-01	0.9996259
MTPN-HUMAN	-0.1354989039	0.9989736	2.843761e-01	0.999995089	-7.118701e-02	0.9996259
PNPO-HUMAN	0.1459813172	0.9989736	-1.885807e-01	0.999995089	1.574075e-01	0.9996259
CRAC1-HUMAN	0.2438818856	0.9989736	3.955108e-02	0.999995089	-1.144042e-01	0.9996259
TMX1-HUMAN	0.0647760349	0.9989736	9.350858e-02	0.999995089	5.492719e-02	0.9996259
IMA3-HUMAN	-0.0409019240	0.9989736	1.664753e-01	0.999995089	-2.532884e-01	0.9996259
CSN2-HUMAN	-0.1081283716	0.9989736	-4.862182e-02	0.999995089	1.551411e-01	0.9996259
RAC2-HUMAN	0.1087297156	0.9989736	2.806194e-04	0.999995089	-5.084197e-03	0.9996259
DHSO-HUMAN	-0.2817761878	0.9989736	3.615796e-02	0.999995089	6.121337e-02	0.9996259
STMN1-HUMAN	-0.0200272127	0.9989736	2.600654e-02	0.999995089	-6.477222e-02	0.9996259
MMP9-HUMAN	0.1898594716	0.9989736	4.099789e-02	0.999995089	1.287754e-01	0.9996259
DYL1-HUMAN	0.2606930378	0.9989736	-4.333792e-03	0.999995089	-4.799040e-02	0.9996259
FHR5-HUMAN	0.1289407751	0.9989736	4.630755e-02	0.999995089	1.134011e-02	0.9996259
PTN11-HUMAN	-0.0871519171	0.9989736	-1.093954e-01	0.999995089	2.941763e-01	0.9996259
LHPP-HUMAN	-0.3309851101	0.9989736	-1.601301e-01	0.999995089	-2.891965e-01	0.9996259
CETP-HUMAN	0.2009346643	0.9989736	-6.284568e-02	0.999995089	-3.163428e-02	0.9996259
ULA1-HUMAN	-0.3658452828	0.9989736	-1.056298e-01	0.999995089	1.375371e-02	0.9996259
SYYC-HUMAN	-0.0342748875	0.9989736	5.242123e-02	0.999995089	1.122531e-01	0.9996259
THG1-HUMAN	0.0750443461	0.9989736	1.621268e-01	0.999995089	-4.807978e-02	0.9996259
RGS10-HUMAN	-0.1802517224	0.9989736	-5.321880e-02	0.999995089	-3.680263e-02	0.9996259
S14L2-HUMAN	0.0005435141	0.9989736	9.944192e-02	0.999995089	-9.037650e-02	0.9996259
PROC-HUMAN	-0.2997819828	0.9989736	-1.507143e-01	0.999995089	-9.555280e-02	0.9996259
RBBP9-HUMAN	0.0235847409	0.9989736	1.730292e-01	0.999995089	-1.922110e-02	0.9996259
PDIA1-HUMAN	0.2451149096	0.9989736	8.775282e-02	0.999995089	-7.381972e-02	0.9996259
VASN-HUMAN	0.1522016061	0.9989736	4.592084e-02	0.999995089	-1.917963e-01	0.9996259
TBCEL-HUMAN	-0.0728360518	0.9989736	-9.391872e-02	0.999995089	-2.773620e-02	0.9996259
BASI-HUMAN	0.2683864133	0.9989736	9.542059e-02	0.999995089	-5.059863e-02	0.9996259
IF5-HUMAN	0.0940077459	0.9989736	-6.419754e-02	0.999995089	2.654386e-02	0.9996259
BPNT1-HUMAN	-0.0444555010	0.9989736	2.708287e-02	0.999995089	-1.490312e-01	0.9996259
GMFB-HUMAN	0.0579298000	0.9989736	7.909042e-02	0.999995089	-1.872049e-02	0.9996259
ATPA-HUMAN	0.1302521224	0.9989736	5.547193e-02	0.999995089	9.963370e-03	0.9996259
KC1A-HUMAN	-0.0279957049	0.9989736	-3.535275e-02	0.999995089	1.085491e-01	0.9996259
DHR11-HUMAN	0.0865036652	0.9989736	1.665821e-01	0.999995089	3.849308e-02	0.9996259
IF1AX-HUMAN	-0.0011624531	0.9989736	1.339018e-01	0.999995089	1.113181e-01	0.9996259
HV315-HUMAN	-0.2693063008	0.9989736	7.164462e-02	0.999995089	3.891564e-02	0.9996259
MOT1-HUMAN	0.1926019682	0.9989736	1.057932e-01	0.999995089	-1.293725e-01	0.9996259
ALDR-HUMAN	-0.1290627256	0.9989736	1.947987e-02	0.999995089	-5.699039e-02	0.9996259
RILP-HUMAN	0.1235759024	0.9989736	7.647033e-02	0.999995089	-1.887394e-01	0.9996259
B2MG-HUMAN	0.0529475455	0.9989736	3.638307e-02	0.999995089	-2.244116e-01	0.9996259
MVD1-HUMAN	-0.0602209570	0.9989736	2.346248e-01	0.999995089	1.053244e-01	0.9996259
OSBP2-HUMAN	0.1147499860	0.9989736	1.464056e-02	0.999995089	-1.231854e-01	0.9996259
DHX9-HUMAN	0.0222305070	0.9989736	-1.070604e-01	0.999995089	-5.896657e-02	0.9996259
LFA3-HUMAN	0.1511754299	0.9989736	1.134016e-01	0.999995089	-3.917966e-03	0.9996259
IBP2-HUMAN	0.1915494242	0.9989736	-1.787328e-02	0.999995089	-1.121868e-01	0.9996259
NCHL1-HUMAN	-0.1248967699	0.9989736	4.369257e-02	0.999995089	1.199708e-02	0.9996259
RAB1A-HUMAN	0.0527766581	0.9989736	7.452567e-02	0.999995089	-1.103949e-01	0.9996259
BACH-HUMAN	-0.0691374865	0.9989736	2.797314e-01	0.999995089	-2.480477e-02	0.9996259

**KPRA-HUMAN**	0.1189478240	0.9989736	3.704474e-01	0.999995089	-5.633912e-02	0.9996259
**VATE1-HUMAN**	-0.1568001402	0.9989736	1.421888e-01	0.999995089	-1.173694e-01	0.9996259
**IBP3-HUMAN**	0.0010238027	0.9989736	9.034998e-02	0.999995089	-1.702649e-01	0.9996259
**MTNA-HUMAN**	0.1740134923	0.9989736	-4.373799e-02	0.999995089	4.100029e-02	0.9996259
**FKBP4-HUMAN**	0.1593431374	0.9989736	1.353928e-01	0.999995089	-6.645491e-02	0.9996259
**MP2K1-HUMAN**	-0.2363354957	0.9989736	-8.256677e-02	0.999995089	5.471355e-02	0.9996259
**PPM1A-HUMAN**	-0.0701574075	0.9989736	6.754338e-02	0.999995089	-9.389535e-02	0.9996259
**LANC2-HUMAN**	-0.0470102644	0.9989736	2.006913e-01	0.999995089	3.216407e-02	0.9996259
**S100P-HUMAN**	0.2477068567	0.9989736	-4.101191e-02	0.999995089	9.411443e-02	0.9996259
**TIMP1-HUMAN**	0.1438762250	0.9989736	7.593359e-02	0.999995089	1.522023e-01	0.9996259
**RAB2A-HUMAN**	-0.0365201662	0.9989736	9.683823e-02	0.999995089	1.430763e-01	0.9996259
**2A5D-HUMAN**	0.0925481240	0.9989736	-6.384336e-03	0.999995089	-8.167141e-02	0.9996259
**ICAM4-HUMAN**	0.0944470764	0.9989736	1.272332e-01	0.999995089	-9.308797e-02	0.9996259
**PDLI1-HUMAN**	0.0657138068	0.9989736	-5.961200e-02	0.999995089	-7.615783e-03	0.9996259
**CRK-HUMAN**	0.0834698144	0.9989736	2.286910e-01	0.999995089	-2.796673e-01	0.9996259
**LSM3-HUMAN**	0.0515452667	0.9989736	-9.568222e-02	0.999995089	-1.628618e-01	0.9996259
**TCTP-HUMAN**	0.0547751769	0.9989736	8.329874e-02	0.999995089	1.188334e-01	0.9996259
**PP6R1-HUMAN**	-0.0557636418	0.9989736	1.184086e-01	0.999995089	1.787149e-01	0.9996259
**DNPEP-HUMAN**	-0.0951826995	0.9989736	1.618645e-02	0.999995089	3.255327e-02	0.9996259
**F1142-HUMAN**	-0.0513939186	0.9989736	-4.562717e-02	0.999995089	3.730068e-02	0.9996259
**ACTN4-HUMAN**	-0.0312224857	0.9989736	4.191771e-02	0.999995089	1.164745e-01	0.9996259
**RTN4-HUMAN**	0.0917152642	0.9989736	1.411591e-01	0.999995089	-9.396283e-04	0.9996259
**RSSA-HUMAN**	-0.0177335275	0.9989736	-6.295672e-01	0.004259862	-2.898288e-01	0.9996259
**BIN2-HUMAN**	-0.0911640988	0.9989736	-1.526291e-02	0.999995089	2.325153e-02	0.9996259
**4EBP1-HUMAN**	-0.0664653324	0.9989736	2.974390e-02	0.999995089	-7.289958e-02	0.9996259
**BTD-HUMAN**	0.0638531730	0.9989736	1.162821e-01	0.999995089	-3.193602e-02	0.9996259
**PFD5-HUMAN**	0.0073262586	0.9989736	3.379591e-02	0.999995089	1.076699e-01	0.9996259
**PIPNB-HUMAN**	-0.0319290643	0.9989736	1.695499e-02	0.999995089	9.084731e-02	0.9996259
**SPP24-HUMAN**	-0.0685200796	0.9989736	-1.401528e-01	0.999995089	-5.896144e-02	0.9996259
**SAA1-HUMAN**	0.1171566270	0.9989736	3.470589e-02	0.999995089	-1.545034e-01	0.9996259
**METK2-HUMAN**	-0.2200045592	0.9989736	-9.387050e-02	0.999995089	1.159320e-01	0.9996259
**ARP3-HUMAN**	0.1895288110	0.9989736	-3.207046e-02	0.999995089	3.883741e-02	0.9996259
**COTL1-HUMAN**	-0.0706656465	0.9989736	-8.296643e-02	0.999995089	-1.622388e-01	0.9996259
**WNK1-HUMAN**	-0.0977531597	0.9989736	3.045441e-02	0.999995089	1.342330e-01	0.9996259
**PCOC1-HUMAN**	-0.0874499627	0.9989736	1.059573e-01	0.999995089	1.385832e-01	0.9996259
**NR2CA-HUMAN**	-0.1087782182	0.9989736	-3.165204e-01	0.999995089	-6.391563e-02	0.9996259
**GPIX-HUMAN**	0.0477384083	0.9989736	6.072311e-02	0.999995089	-1.584415e-02	0.9996259
**FKBP3-HUMAN**	0.2412522616	0.9989736	8.939687e-02	0.999995089	1.850733e-01	0.9996259
**RAB6B-HUMAN**	0.0545504445	0.9989736	1.712620e-01	0.999995089	-1.513083e-01	0.9996259
**ENOPH-HUMAN**	0.0037820081	0.9989736	2.202048e-02	0.999995089	3.558130e-02	0.9996259
**ZPI-HUMAN**	-0.2534594703	0.9989736	7.164133e-02	0.999995089	1.551976e-01	0.9996259
**UBC12-HUMAN**	-0.0911625385	0.9989736	5.800538e-02	0.999995089	-9.105995e-02	0.9996259
**AQP1-HUMAN**	0.1612607708	0.9989736	8.831864e-02	0.999995089	-2.702774e-02	0.9996259
**QSPP-HUMAN**	-0.2035511623	0.9989736	8.996693e-02	0.999995089	-1.129885e-01	0.9996259
**HV226-HUMAN**	0.0660356550	0.9989736	1.217125e-01	0.999995089	-1.949042e-01	0.9996259
**AT2B1-HUMAN**	0.0120874200	0.9989736	1.511534e-02	0.999995089	2.038355e-01	0.9996259
**HXK1-HUMAN**	0.1435171747	0.9989736	-9.717581e-02	0.999995089	-3.222362e-03	0.9996259
**CUL2-HUMAN**	-0.0403518040	0.9989736	9.402211e-02	0.999995089	1.083396e-02	0.9996259
**DAF-HUMAN**	0.1937533524	0.9989736	9.202481e-02	0.999995089	-7.613465e-02	0.9996259
**DEOC-HUMAN**	-0.1237668229	0.9989736	2.612271e-02	0.999995089	5.185652e-02	0.9996259
**PTBP1-HUMAN**	0.1420126438	0.9989736	-7.008524e-02	0.999995089	-1.220756e-01	0.9996259
**LCAT-HUMAN**	-0.0442556367	0.9989736	2.330580e-02	0.999995089	-1.768059e-01	0.9996259
**PLP2-HUMAN**	0.3639840120	0.9989736	4.583353e-02	0.999995089	-4.956977e-02	0.9996259
**DIAP1-HUMAN**	0.0683658207	0.9989736	-8.530447e-03	0.999995089	-1.843005e-01	0.9996259
**VAC14-HUMAN**	0.1320169510	0.9989736	-6.127090e-01	0.009080189	-1.315031e-01	0.9996259
**SHLB2-HUMAN**	-0.0293558943	0.9989736	-6.964323e-02	0.999995089	1.307553e-01	0.9996259
**AT1A1-HUMAN**	0.1955126635	0.9989736	6.921941e-02	0.999995089	-8.360478e-02	0.9996259
**RAB7A-HUMAN**	0.0783888440	0.9989736	9.779462e-02	0.999995089	8.654005e-02	0.9996259
**MDHM-HUMAN**	0.1461815226	0.9989736	-3.253733e-01	0.999995089	-3.567551e-02	0.9996259
**DNJA4-HUMAN**	0.1681695368	0.9989736	2.257391e-02	0.999995089	-1.003912e-01	0.9996259
**RAB21-HUMAN**	0.1883566143	0.9989736	1.761484e-01	0.999995089	-1.557694e-01	0.9996259
**SC22B-HUMAN**	0.1337919162	0.9989736	8.198829e-02	0.999995089	-8.200687e-02	0.9996259
**ENOF1-HUMAN**	0.1765788525	0.9989736	9.598961e-02	0.999995089	-3.453370e-02	0.9996259
**XPP1-HUMAN**	0.1697073015	0.9989736	3.911442e-02	0.999995089	-8.358135e-02	0.9996259
**VASP-HUMAN**	0.0230491470	0.9989736	4.765565e-02	0.999995089	2.333469e-01	0.9996259
**CNDP1-HUMAN**	-0.2949487593	0.9989736	-1.475077e-01	0.999995089	1.550159e-01	0.9996259
**ERF1-HUMAN**	0.0629791233	0.9989736	8.503271e-02	0.999995089	1.881045e-01	0.9996259
**ARLY-HUMAN**	-0.1409108923	0.9989736	-2.610766e-02	0.999995089	1.394219e-01	0.9996259
**COR1C-HUMAN**	0.0167018389	0.9989736	-1.205614e-01	0.999995089	-1.448033e-01	0.9996259
**RAB5C-HUMAN**	-0.0386507171	0.9989736	5.565106e-02	0.999995089	-1.213148e-01	0.9996259
**TRXR1-HUMAN**	-0.0837402050	0.9989736	1.223453e-01	0.999995089	-4.946083e-02	0.9996259
**IF1AY-HUMAN**	-0.2899738692	0.9989736	7.537030e-02	0.999995089	7.924968e-03	0.9996259

**FCGBP-HUMAN**	0.2246849089	0.9989736	-3.203592e-02	0.999995089	-1.616010e-01	0.9996259
**NUDC1-HUMAN**	0.0537557641	0.9989736	1.129392e-01	0.999995089	8.811785e-02	0.9996259
**AMPD3-HUMAN**	-0.2621429744	0.9989736	3.235283e-01	0.999995089	8.962348e-03	0.9996259
**RIC8A-HUMAN**	-0.1115310668	0.9989736	-1.232315e-01	0.999995089	-1.417281e-01	0.9996259
**AN32A-HUMAN**	0.0303235131	0.9989736	-2.798502e-01	0.999995089	-2.954516e-01	0.9996259
**GNL1-HUMAN**	-0.1083378994	0.9989736	-4.900727e-02	0.999995089	-3.010197e-02	0.9996259
**ARC1B-HUMAN**	-0.0403397277	0.9989736	-1.379098e-01	0.999995089	-4.593597e-02	0.9996259
**CHM4B-HUMAN**	-0.0005280134	0.9989736	-8.988566e-02	0.999995089	1.579608e-01	0.9996259
**AP4A-HUMAN**	-0.1411967756	0.9989736	-3.872033e-01	0.999995089	2.246503e-01	0.9996259
**KRT82-HUMAN**	-0.0789093609	0.9989736	-1.650868e-01	0.999995089	-7.340474e-03	0.9996259
**GANAB-HUMAN**	0.0053416573	0.9989736	-5.347091e-02	0.999995089	7.388720e-02	0.9996259
**HEBP2-HUMAN**	0.2124818931	0.9989736	-9.596423e-02	0.999995089	1.321591e-01	0.9996259
**HNRPF-HUMAN**	0.1528925735	0.9989736	-4.883744e-02	0.999995089	1.996151e-01	0.9996259
**AAMDC-HUMAN**	-0.0855306151	0.9989736	-6.688245e-02	0.999995089	-1.898333e-02	0.9996259
**UBP7-HUMAN**	0.0193930039	0.9989736	7.111007e-03	0.999995089	-2.636217e-01	0.9996259
**LG3BP-HUMAN**	-0.2508554713	0.9989736	5.192815e-02	0.999995089	-1.533508e-01	0.9996259
**GPV-HUMAN**	0.0345773570	0.9989736	4.224218e-02	0.999995089	-4.418621e-02	0.9996259
**CYRIB-HUMAN**	0.2861604767	0.9989736	-8.993746e-02	0.999995089	-5.128116e-02	0.9996259
**GMPPA-HUMAN**	-0.2072741776	0.9989736	8.508260e-02	0.999995089	1.389211e-01	0.9996259
**FCN2-HUMAN**	-0.0034356060	0.9989736	1.103220e-01	0.999995089	-2.415864e-02	0.9996259
**USP9X-HUMAN**	0.0117811344	0.9989736	9.569830e-02	0.999995089	2.981854e-02	0.9996259
**CN37-HUMAN**	0.2017653615	0.9989736	-9.007002e-02	0.999995089	5.572207e-02	0.9996259
**PYGL-HUMAN**	0.0906724057	0.9989736	1.213423e-01	0.999995089	1.660047e-01	0.9996259
**ILK-HUMAN**	-0.0078875814	0.9989736	4.242682e-02	0.999995089	6.894491e-02	0.9996259
**PSMG1-HUMAN**	0.1640485312	0.9989736	-1.507274e-01	0.999995089	-6.575654e-02	0.9996259
**SRC8-HUMAN**	-0.0307755400	0.9989736	-2.333155e-03	0.999995089	7.103988e-02	0.9996259
**HDHD2-HUMAN**	-0.0529004592	0.9989736	1.921605e-02	0.999995089	-1.093515e-01	0.9996259
**CHM4A-HUMAN**	0.0188881224	0.9989736	-1.897762e-01	0.999995089	-4.213041e-02	0.9996259
**ABCB6-HUMAN**	0.2438469829	0.9989736	9.352622e-02	0.999995089	-1.493172e-02	0.9996259
**TBC24-HUMAN**	-0.0028587955	0.9989736	1.787795e-01	0.999995089	2.578059e-01	0.9996259
**PARVB-HUMAN**	0.0451951803	0.9989736	-1.475554e-01	0.999995089	-7.807337e-03	0.9996259
**CPPED-HUMAN**	-0.1564255521	0.9989736	1.619401e-01	0.999995089	2.673814e-01	0.9996259
**TMED2-HUMAN**	0.2104955489	0.9989736	6.134324e-02	0.999995089	-8.825121e-02	0.9996259
**FAS-HUMAN**	0.2291978043	0.9989736	4.949474e-02	0.999995089	-1.870390e-01	0.9996259
**BIP-HUMAN**	0.2309345327	0.9989736	1.226010e-01	0.999995089	-5.868886e-02	0.9996259
**ELOB-HUMAN**	-0.0673792589	0.9989736	1.318156e-01	0.999995089	1.561231e-02	0.9996259
**YKT6-HUMAN**	-0.1585289685	0.9989736	-6.747366e-02	0.999995089	-5.897383e-02	0.9996259
**LV460-HUMAN**	0.0176357188	0.9989736	1.302781e-01	0.999995089	-2.462352e-01	0.9996259
**ITB2-HUMAN**	0.1871812139	0.9989736	1.400702e-01	0.999995089	1.736700e-01	0.9996259
**RADI-HUMAN**	0.1876548070	0.9989736	3.919527e-02	0.999995089	-5.318526e-02	0.9996259
**HNRPQ-HUMAN**	-0.0317788411	0.9989736	6.010705e-02	0.999995089	8.391290e-02	0.9996259
**COASY-HUMAN**	-0.0032764950	0.9989736	3.446398e-02	0.999995089	-9.076076e-02	0.9996259
**PI16-HUMAN**	-0.0145961075	0.9989736	-1.854867e-01	0.999995089	-2.739200e-02	0.9996259
**S10AB-HUMAN**	0.1262216569	0.9989736	1.002143e-01	0.999995089	1.279107e-01	0.9996259
**UCHL5-HUMAN**	-0.2931990013	0.9989736	6.884423e-02	0.999995089	-1.008018e-01	0.9996259
**LZIC-HUMAN**	0.1746036072	0.9989736	-6.318267e-02	0.999995089	3.061350e-02	0.9996259
**HGS-HUMAN**	-0.0737092874	0.9989736	-4.340728e-03	0.999995089	4.244910e-02	0.9996259
**RS12-HUMAN**	0.0367265722	0.9989736	1.372751e-01	0.999995089	1.196276e-01	0.9996259
**TFR1-HUMAN**	-0.0038503082	0.9989736	7.765232e-02	0.999995089	2.560806e-01	0.9996259
**IF2B-HUMAN**	-0.0338381852	0.9989736	-1.278805e-01	0.999995089	7.083666e-02	0.9996259
**ECP-HUMAN**	0.1835578485	0.9989736	5.791834e-02	0.999995089	9.647962e-02	0.9996259
**PUR2-HUMAN**	-0.1698283590	0.9989736	7.113397e-02	0.999995089	-2.135256e-01	0.9996259
**MASP2-HUMAN**	0.0956707065	0.9989736	-1.488345e-01	0.999995089	-8.911628e-02	0.9996259
**LYAM1-HUMAN**	-0.1057187559	0.9989736	-8.206520e-02	0.999995089	-2.393744e-01	0.9996259
**MOB1B-HUMAN;MOB1A-HUMAN**	-0.0863316782	0.9989736	-5.970032e-03	0.999995089	6.580101e-02	0.9996259
**CLIC2-HUMAN**	0.1692956301	0.9989736	-1.134938e-01	0.999995089	3.714657e-03	0.9996259
**SIAS-HUMAN**	-0.1699803982	0.9989736	3.577281e-02	0.999995089	-5.549497e-02	0.9996259
**PRG4-HUMAN**	-0.0779327796	0.9989736	-9.706713e-02	0.999995089	2.408113e-02	0.9996259
**ADK-HUMAN**	-0.1668533349	0.9989736	6.335138e-02	0.999995089	-1.015789e-01	0.9996259
**TNPO1-HUMAN**	0.0324724143	0.9989736	4.493331e-02	0.999995089	-1.020260e-01	0.9996259
**ADPRS-HUMAN**	0.1161968613	0.9989736	-2.381806e-01	0.999995089	-2.065970e-01	0.9996259
**CADH5-HUMAN**	0.0290598169	0.9989736	9.687026e-03	0.999995089	-1.349932e-01	0.9996259
**COMP-HUMAN**	0.0532857147	0.9989736	-5.673322e-02	0.999995089	1.095480e-01	0.9996259
**KVD29-HUMAN;KV229-HUMAN**	-0.0402327519	0.9989736	5.424961e-02	0.999995089	-4.987567e-02	0.9996259
**ACOC-HUMAN**	-0.1244342292	0.9989736	1.881623e-01	0.999995089	-1.322435e-01	0.9996259
**CRIS3-HUMAN**	-0.0174370851	0.9989736	1.384125e-01	0.999995089	4.552491e-03	0.9996259
**LEG10-HUMAN**	0.0241245179	0.9989736	1.483239e-01	0.999995089	3.465732e-02	0.9996259
**GPX4-HUMAN**	0.0020455326	0.9989736	1.256240e-01	0.999995089	-1.679465e-02	0.9996259
**UBP47-HUMAN**	-0.2003825205	0.9989736	6.594747e-02	0.999995089	1.367546e-01	0.9996259
**DCTN1-HUMAN**	-0.1537019151	0.9989736	5.602703e-02	0.999995089	-1.973472e-01	0.9996259
**CPNE1-HUMAN**	0.0187658601	0.9989736	2.131372e-02	0.999995089	1.905760e-02	0.9996259
**PCYOX-HUMAN**	0.2199580413	0.9989736	-1.361308e-01	0.999995089	-5.876011e-02	0.9996259

IBP5-HUMAN	-0.0314981994	0.9989736	-2.161481e-02	0.999995089	1.243975e-01	0.9996259
RAB14-HUMAN	0.0295119873	0.9989736	1.723558e-01	0.999995089	4.128475e-02	0.9996259
JAM1-HUMAN	0.1934086624	0.9989736	7.002137e-02	0.999995089	4.538368e-02	0.9996259
TBCB-HUMAN	0.0487297771	0.9989736	8.175743e-03	0.999995089	3.254364e-01	0.9996259
ERP44-HUMAN	0.1854669210	0.9989736	6.627701e-02	0.999995089	9.328249e-02	0.9996259
SRGN-HUMAN	-0.0561057662	0.9989736	5.984456e-02	0.999995089	3.947752e-03	0.9996259
CALD1-HUMAN	0.1418136258	0.9989736	-1.283767e-03	0.999995089	2.302051e-02	0.9996259
PLS1-HUMAN	0.2164417679	0.9989736	6.431046e-02	0.999995089	-4.224341e-02	0.9996259
KS6A3-HUMAN	-0.0309620855	0.9989736	9.810425e-02	0.999995089	1.268090e-01	0.9996259
EZRI-HUMAN	-0.1177370311	0.9989736	1.187107e-01	0.999995089	-7.552687e-02	0.9996259
LV746-HUMAN	-0.0990504935	0.9989736	-4.708869e-02	0.999995089	2.045623e-01	0.9996259
KCMF1-HUMAN	-0.1793863669	0.9989736	6.198912e-02	0.999995089	-1.096834e-01	0.9996259
QORX-HUMAN	-0.1978872895	0.9989736	9.708079e-02	0.999995089	-1.131799e-01	0.9996259
PTRD1-HUMAN	-0.0299497956	0.9989736	1.083766e-01	0.999995089	-1.239948e-01	0.9996259
AT2A3-HUMAN	0.0966802362	0.9989736	3.850315e-02	0.999995089	-7.270978e-02	0.9996259
LAT4-HUMAN	-0.0170942666	0.9989736	1.164658e-01	0.999995089	8.793110e-05	0.9996259
SYCC-HUMAN	-0.0652639846	0.9989736	7.557365e-02	0.999995089	4.669420e-02	0.9996259
LV316-HUMAN	-0.0991005328	0.9989736	-1.626443e-02	0.999995089	8.053392e-02	0.9996259
GTR3-HUMAN;GTR14-HUMAN	0.0877238575	0.9989736	4.503724e-02	0.999995089	-1.001030e-01	0.9996259
KVD21-HUMAN	-0.0339690019	0.9989736	1.481383e-02	0.999995089	3.011249e-02	0.9996259
ELOC-HUMAN	-0.1830246452	0.9989736	5.776713e-02	0.999995089	1.354321e-01	0.9996259
PEF1-HUMAN	-0.1159697515	0.9989736	1.478815e-01	0.999995089	-5.598580e-02	0.9996259
RNAS1-HUMAN	0.2425835542	0.9989736	1.835302e-02	0.999995089	-2.547885e-01	0.9996259
ATG4A-HUMAN	-0.1629796610	0.9989736	1.156850e-01	0.999995089	1.812015e-01	0.9996259
LYVE1-HUMAN	0.2621285230	0.9989736	-1.025330e-01	0.999995089	-9.019282e-02	0.9996259
MPCP-HUMAN	0.2159226648	0.9989736	7.748281e-02	0.999995089	-6.252730e-02	0.9996259
IGE-HUMAN	-0.1265475871	0.9989736	1.172185e-01	0.999995089	1.765501e-01	0.9996259
ASAP1-HUMAN	0.0165944416	0.9989736	-1.102340e-01	0.999995089	-6.513740e-02	0.9996259
GNAI2-HUMAN	0.2349948007	0.9989736	8.591072e-02	0.999995089	-1.067407e-02	0.9996259
SCFD1-HUMAN	-0.0271432297	0.9989736	5.466023e-02	0.999995089	1.479758e-03	0.9996259
CLIC4-HUMAN	0.0045722354	0.9989736	5.714921e-02	0.999995089	-3.641648e-02	0.9996259
TSNAX-HUMAN	0.0323067779	0.9989736	2.669598e-02	0.999995089	1.788846e-01	0.9996259
GGEE3-HUMAN;PAGE2-HUMAN	0.0039235363	0.9989736	9.864570e-02	0.999995089	8.649681e-03	0.9996259
GLGB-HUMAN	-0.2401043846	0.9989736	-2.269575e-01	0.999995089	1.653650e-02	0.9996259
DENR-HUMAN	-0.0488427335	0.9989736	5.413944e-03	0.999995089	5.133815e-02	0.9996259
PGRP1-HUMAN	0.1527082876	0.9989736	-1.515367e-02	0.999995089	8.066284e-02	0.9996259
SELO-HUMAN	0.0890315975	0.9989736	1.772391e-01	0.999995089	-1.845640e-02	0.9996259
CD99-HUMAN	-0.0164636620	0.9989736	3.191129e-02	0.999995089	-6.231336e-02	0.9996259
VAMP2-HUMAN;VAMP3-HUMAN	0.1982129187	0.9989736	8.931167e-02	0.999995089	1.042729e-01	0.9996259
PSMD8-HUMAN	0.0646284594	0.9989736	1.286712e-01	0.999995089	-9.887279e-02	0.9996259
CH10-HUMAN	-0.2914779323	0.9989736	3.678177e-02	0.999995089	-9.476413e-02	0.9996259
WIPI1-HUMAN	0.0172861552	0.9989736	2.221396e-01	0.999995089	-6.804736e-02	0.9996259
ANXA4-HUMAN	0.2290321408	0.9989736	-7.733365e-02	0.999995089	-1.321021e-01	0.9996259
HMGN2-HUMAN	0.2167476849	0.9989736	5.150920e-03	0.999995089	3.789472e-02	0.9996259
AMPB-HUMAN	-0.1154836328	0.9989736	-4.595829e-02	0.999995089	-6.556765e-02	0.9996259
LTBP1-HUMAN	-0.1649671865	0.9989736	3.988965e-02	0.999995089	1.029086e-01	0.9996259
EF1G-HUMAN	0.0882214123	0.9989736	-2.611378e-01	0.999995089	1.833552e-01	0.9996259
GLU2B-HUMAN	0.1580516858	0.9989736	6.963346e-02	0.999995089	-6.396850e-03	0.9996259
HMGB2-HUMAN	0.1458826705	0.9989736	-1.084573e-01	0.999995089	1.605359e-01	0.9996259
EF1D-HUMAN	0.1348794551	0.9989736	4.589012e-02	0.999995089	-2.796048e-02	0.9996259
CALR-HUMAN	0.2107491466	0.9989736	6.391054e-02	0.999995089	-5.295799e-02	0.9996259
CATD-HUMAN	0.1642723892	0.9989736	1.348146e-01	0.999995089	-2.219261e-02	0.9996259
MMRN1-HUMAN	-0.1917905021	0.9989736	-1.333479e-01	0.999995089	1.885730e-02	0.9996259
CSRP1-HUMAN	0.1310710317	0.9989736	1.057193e-01	0.999995089	3.465253e-01	0.9996259
ERF3A-HUMAN	0.1226765305	0.9989736	-1.021194e-02	0.999995089	1.975713e-01	0.9996259
PEBP4-HUMAN	0.0462635278	0.9989736	-2.068997e-01	0.999995089	-1.508082e-01	0.9996259
GNAI3-HUMAN	0.2478810458	0.9989736	1.240438e-01	0.999995089	4.532656e-02	0.9996259
UBA3-HUMAN	0.0789263262	0.9989736	4.519161e-02	0.999995089	8.612760e-02	0.9996259
TBG1-HUMAN;TBG2-HUMAN	-0.0954760980	0.9989736	1.092022e-01	0.999995089	2.762061e-01	0.9996259
VTA1-HUMAN	-0.0405629316	0.9989736	6.435326e-02	0.999995089	2.786423e-01	0.9996259
K1H1-HUMAN	-0.0607581662	0.9989736	6.066211e-02	0.999995089	3.682216e-02	0.9996259
RS17-HUMAN	0.0854521008	0.9989736	-6.022799e-02	0.999995089	-1.938867e-01	0.9996259
BCAM-HUMAN	0.1968226979	0.9989736	8.282528e-02	0.999995089	-4.411820e-03	0.9996259
YBOX3-HUMAN	0.1598017238	0.9989736	5.748986e-02	0.999995089	4.384931e-02	0.9996259
ISG15-HUMAN	-0.2732433684	0.9989736	-3.272069e-02	0.999995089	-2.115367e-01	0.9996259
LV548-HUMAN	0.1822015708	0.9989736	3.090367e-02	0.999995089	1.025879e-01	0.9996259
NNRE-HUMAN	-0.1743887420	0.9989736	-1.610954e-02	0.999995089	-1.061631e-01	0.9996259
DPYL2-HUMAN	-0.1068731797	0.9989736	1.041002e-01	0.999995089	9.472106e-02	0.9996259
BASP1-HUMAN	0.0443269378	0.9989736	1.553986e-02	0.999995089	4.318928e-02	0.9996259
XRP2-HUMAN	0.1883966700	0.9989736	8.730048e-02	0.999995089	4.815476e-02	0.9996259
DOPO-HUMAN	0.1389203510	0.9989736	1.203336e-01	0.999995089	4.417609e-02	0.9996259
MBL2-HUMAN	0.2232757424	0.9989736	-3.265113e-02	0.999995089	-2.209481e-03	0.9996259

**PURA-HUMAN**	-0.0165319780	0.9989736	6.238054e-02	0.999995089	-1.074078e-02	0.9996259
**RHAG-HUMAN**	0.2716338597	0.9989736	7.537770e-02	0.999995089	-7.880204e-02	0.9996259
**PSMG2-HUMAN**	0.0621758005	0.9989736	-2.701606e-02	0.999995089	-1.271364e-01	0.9996259
**VP26B-HUMAN**	0.1495647937	0.9989736	2.662458e-02	0.999995089	2.904430e-01	0.9996259
**SAA2-HUMAN**	0.0310877604	0.9989736	3.467825e-02	0.999995089	-1.353062e-01	0.9996259
**DNJA2-HUMAN**	0.0858404164	0.9989736	1.798985e-01	0.999995089	1.044955e-01	0.9996259
**GLPA-HUMAN**	0.3114296968	0.9989736	1.043603e-01	0.999995089	-7.939369e-02	0.9996259
**PIGR-HUMAN**	-0.0058653259	0.9989736	8.215337e-02	0.999995089	8.298623e-02	0.9996259
**LMAN2-HUMAN**	0.2403941270	0.9989736	1.204324e-01	0.999995089	-5.352440e-02	0.9996259
**HCLS1-HUMAN**	-0.1528473382	0.9989736	3.256105e-02	0.999995089	-8.584154e-02	0.9996259
**ANGI-HUMAN**	-0.0062739459	0.9989736	1.013512e-01	0.999995089	1.972173e-01	0.9996259
**SPRC-HUMAN**	-0.2002254613	0.9989736	-1.560490e-01	0.999995089	1.042987e-01	0.9996259
**RAB2B-HUMAN**	0.0352598605	0.9989736	-1.817598e-01	0.999995089	1.350852e-01	0.9996259
**MP2K3-HUMAN**	0.0225338004	0.9989736	1.754803e-02	0.999995089	-2.015027e-02	0.9996259
**MP2K2-HUMAN**	-0.0935705818	0.9989736	2.627934e-02	0.999995089	-8.802831e-03	0.9996259
**LA-HUMAN**	-0.1473199122	0.9989736	-4.062340e-02	0.999995089	-1.658145e-01	0.9996259
**CSN7A-HUMAN**	-0.0748183444	0.9989736	-1.145704e-01	0.999995089	3.127560e-01	0.9996259
**NRBP-HUMAN**	0.0238795255	0.9989736	1.089986e-01	0.999995089	-1.948698e-02	0.9996259
**HLAA-HUMAN**	0.0142284702	0.9989736	2.242993e-03	0.999995089	-9.135095e-02	0.9996259
**PDCD5-HUMAN**	-0.0893820138	0.9989736	-2.447046e-03	0.999995089	-1.245579e-01	0.9996259
**CY24B-HUMAN**	0.1327820995	0.9989736	4.265482e-02	0.999995089	3.645900e-02	0.9996259
**CD36-HUMAN**	0.2169069550	0.9989736	9.727706e-02	0.999995089	-1.786121e-01	0.9996259
**SERC-HUMAN**	0.1016468524	0.9989736	6.504150e-02	0.999995089	-1.517501e-01	0.9996259
**AHNK-HUMAN**	0.1129490283	0.9989736	3.817329e-02	0.999995089	-1.193623e-01	0.9996259
**LAMP2-HUMAN**	-0.0223827753	0.9989736	1.033819e-02	0.999995089	-1.476102e-01	0.9996259
**IDHP-HUMAN**	0.1685506904	0.9989736	5.489637e-02	0.999995089	-1.500179e-02	0.9996259
**FCN1-HUMAN**	-0.1568211996	0.9989736	1.697477e-02	0.999995089	2.136869e-01	0.9996259
**PDIA4-HUMAN**	-0.2391621836	0.9989736	4.247440e-02	0.999995089	2.261087e-01	0.9996259
**FHL1-HUMAN**	-0.1027662914	0.9989736	1.127430e-01	0.999995089	-8.655303e-02	0.9996259
**CALX-HUMAN**	0.2266291327	0.9989736	8.746010e-02	0.999995089	-4.451167e-04	0.9996259
**NEUG-HUMAN**	-0.0086551694	0.9989736	4.477483e-02	0.999995089	-1.289699e-01	0.9996259
**ITAM-HUMAN**	0.2709998695	0.9989736	9.326719e-02	0.999995089	-1.816554e-01	0.9996259
**VNN1-HUMAN**	0.1769738165	0.9989736	6.215860e-02	0.999995089	2.423206e-01	0.9996259
**THAS-HUMAN**	0.2724763025	0.9989736	1.477089e-02	0.999995089	-3.492742e-02	0.9996259
**ROA1-HUMAN**	-0.2108495549	0.9989736	3.629587e-02	0.999995089	8.062061e-02	0.9996259
**HUWE1-HUMAN**	-0.0370504608	0.9989736	1.473840e-01	0.999995089	-2.172585e-02	0.9996259
**PGTB2-HUMAN**	0.1479010578	0.9989736	7.250238e-02	0.999995089	8.386496e-02	0.9996259
**YBOX1-HUMAN**	0.2144246215	0.9989736	1.318756e-01	0.999995089	4.542685e-02	0.9996259
**PIEZ1-HUMAN**	0.3356095176	0.9989736	6.768638e-02	0.999995089	-5.787886e-02	0.9996259
**SEM7A-HUMAN**	0.1805401197	0.9989736	6.773981e-02	0.999995089	9.955259e-02	0.9996259
**RIR1-HUMAN**	0.0421374653	0.9989736	9.224670e-02	0.999995089	-2.046260e-03	0.9996259
**SAC1-HUMAN**	0.2178854120	0.9989736	8.047380e-02	0.999995089	-1.982473e-02	0.9996259
**LVX54-HUMAN**	-0.0691355862	0.9989736	8.329463e-02	0.999995089	6.890862e-02	0.9996259
**PPP5-HUMAN**	-0.0017815689	0.9989736	4.507898e-02	0.999995089	-1.255675e-01	0.9996259
**ADRM1-HUMAN**	-0.1930007484	0.9989736	1.609017e-01	0.999995089	-1.119559e-01	0.9996259
**PLPL6-HUMAN**	0.1287830135	0.9989736	1.599842e-01	0.999995089	-9.590286e-02	0.9996259
**SNP23-HUMAN**	0.1310264281	0.9989736	9.588027e-02	0.999995089	-7.637653e-03	0.9996259
**CH60-HUMAN**	0.1542973157	0.9989736	1.589386e-01	0.999995089	-4.253337e-02	0.9996259
**IGBP1-HUMAN**	-0.2588241390	0.9989736	5.125426e-03	0.999995089	2.748331e-02	0.9996259
**DCA11-HUMAN**	-0.2854022817	0.9989736	2.640017e-02	0.999995089	1.284914e-01	0.9996259
**GGH-HUMAN**	0.0150260248	0.9989736	5.276965e-02	0.999995089	3.893966e-02	0.9996259
**HV320-HUMAN**	-0.2268891097	0.9989736	8.539793e-02	0.999995089	-1.316700e-01	0.9996259
**PCNP-HUMAN**	-0.1341619995	0.9989736	-1.831652e-01	0.999995089	-1.959454e-02	0.9996259
**H2AZ-HUMAN;H2AV-HUMAN**	0.2392798514	0.9989736	-5.405643e-02	0.999995089	-5.511276e-02	0.9996259
**TXND5-HUMAN**	0.1527557347	0.9989736	1.072098e-01	0.999995089	-1.706080e-01	0.9996259
**AL5AP-HUMAN**	0.2201979589	0.9989736	8.158809e-02	0.999995089	7.373255e-02	0.9996259
**GNAS2-HUMAN;GNAS1-HUMAN**	0.0955255084	0.9989736	6.925039e-02	0.999995089	4.750269e-02	0.9996259
**COIA1-HUMAN**	0.1270157324	0.9989736	-5.994184e-03	0.999995089	-1.748687e-01	0.9996259
**ROA2-HUMAN**	0.0634830310	0.9989736	-9.446694e-03	0.999995089	-1.125358e-01	0.9996259
**TTC38-HUMAN**	0.0993387266	0.9989736	-1.708411e-01	0.999995089	3.056128e-02	0.9996259
**COL11-HUMAN**	0.0952389401	0.9989736	9.843781e-02	0.999995089	7.026841e-02	0.9996259
**ACSL3-HUMAN**	0.1183046677	0.9989736	3.536430e-02	0.999995089	-5.824975e-02	0.9996259
**PROZ-HUMAN**	-0.0781592053	0.9989736	8.543720e-02	0.999995089	6.107802e-03	0.9996259
**CAN5-HUMAN**	0.2826064946	0.9989736	7.463883e-02	0.999995089	-4.091469e-02	0.9996259
**PPP6-HUMAN**	-0.0051221760	0.9989736	1.394499e-01	0.999995089	6.552468e-02	0.9996259
**LV545-HUMAN;LV539-HUMAN**	-0.1335808650	0.9989736	-6.299719e-02	0.999995089	-2.062188e-01	0.9996259
**S29A1-HUMAN**	0.1348422888	0.9989736	7.674533e-02	0.999995089	4.323509e-02	0.9996259
**KT33A-HUMAN**	0.0485449158	0.9989736	-2.790125e-02	0.999995089	-2.590749e-02	0.9996259
**LAMP1-HUMAN**	0.2312388450	0.9989736	6.607707e-02	0.999995089	-5.686266e-03	0.9996259
**VAPA-HUMAN**	0.2214393664	0.9989736	9.198882e-02	0.999995089	-5.117454e-02	0.9996259
**CAD13-HUMAN**	-0.0959692893	0.9989736	9.067702e-02	0.999995089	6.270166e-02	0.9996259
**FCERG-HUMAN**	0.2786475173	0.9989736	2.758506e-02	0.999995089	-1.713656e-03	0.9996259

PTMA-HUMAN	-0.1430391087	0.9989736	-6.128354e-02	0.999995089	-3.883949e-02	0.9996259
PLBL1-HUMAN	0.2230561121	0.9989736	1.382785e-02	0.999995089	1.459168e-01	0.9996259
S43A3-HUMAN	0.1104148678	0.9989736	1.025085e-01	0.999995089	-1.686480e-01	0.9996259
CR1-HUMAN	0.3807418089	0.9989736	7.813553e-02	0.999995089	3.310308e-02	0.9996259
S40A1-HUMAN	0.0679039952	0.9989736	9.787802e-02	0.999995089	3.210506e-02	0.9996259
MYG-HUMAN	-0.1304367611	0.9989736	2.766888e-02	0.999995089	-1.450738e-01	0.9996259
MRP4-HUMAN	0.2331969383	0.9989736	9.117534e-02	0.999995089	-7.221998e-02	0.9996259
ARI2-HUMAN	0.0144611870	0.9989736	1.159903e-01	0.999995089	6.385985e-02	0.9996259
SNX3-HUMAN	-0.0806137280	0.9989736	1.547805e-01	0.999995089	1.410932e-02	0.9996259
CIAO1-HUMAN	-0.2084979694	0.9989736	-1.078381e-02	0.999995089	-1.012312e-01	0.9996259
EFHD2-HUMAN;EFHD1-HUMAN	0.0830932263	0.9989736	-1.154971e-02	0.999995089	2.081526e-01	0.9996259
RL7-HUMAN	-0.1024206149	0.9989736	1.729018e-01	0.999995089	-4.952705e-02	0.9996259
MYH10-HUMAN	-0.4399007099	0.9989736	3.335942e-02	0.999995089	-1.487024e-02	0.9996259
REEP5-HUMAN	0.1296477171	0.9989736	8.467021e-02	0.999995089	-3.904027e-02	0.9996259
K1C9-HUMAN	0.0144722593	0.9989736	7.463472e-02	0.999995089	9.659844e-02	0.9996259
COX41-HUMAN	0.1916220486	0.9989736	3.999787e-02	0.999995089	-4.513613e-02	0.9996259
KRT35-HUMAN	0.0381756511	0.9989736	2.555867e-02	0.999995089	-2.477071e-02	0.9996259
ISK5-HUMAN	0.0097115834	0.9989736	3.530132e-03	0.999995089	-2.019665e-01	0.9996259
C2C2L-HUMAN	0.2728681986	0.9989736	8.584932e-02	0.999995089	-3.931872e-02	0.9996259
TPM2-HUMAN	0.0976672061	0.9989736	7.903851e-02	0.999995089	3.468784e-02	0.9996259
K22E-HUMAN	-0.0234629163	0.9989736	6.339199e-02	0.999995089	-5.031756e-02	0.9996259
ITB1-HUMAN	0.0381953722	0.9989736	7.458553e-02	0.999995089	-9.034012e-02	0.9996259
KR111-HUMAN	-0.0528853027	0.9989736	-1.557037e-01	0.999995089	1.013963e-02	0.9996259
MPU1-HUMAN	0.3684074025	0.9989736	6.075785e-02	0.999995089	-7.159056e-02	0.9996259
COX2-HUMAN	0.1955098625	0.9989736	8.444590e-02	0.999995089	-6.853380e-02	0.9996259
IGHD-HUMAN	-0.1380776579	0.9989736	1.314595e-02	0.999995089	-7.851834e-02	0.9996259
UTER-HUMAN	0.0733373418	0.9989736	4.234770e-02	0.999995089	-2.609737e-01	0.9996259
GUAA-HUMAN	-0.2536638122	0.9989736	6.708942e-02	0.999995089	5.130506e-02	0.9996259
WFDC2-HUMAN	-0.0429579459	0.9989736	8.989891e-02	0.999995089	7.296579e-02	0.9996259
AR6P1-HUMAN	0.3495816886	0.9989736	7.652060e-02	0.999995089	-7.559020e-02	0.9996259
SBSN-HUMAN	0.2809149398	0.9989736	4.368258e-02	0.999995089	1.003791e-02	0.9996259
CD177-HUMAN	-0.0022914280	0.9989736	3.069727e-02	0.999995089	-5.864914e-02	0.9996259
RDH11-HUMAN	0.0730182254	0.9989736	6.587838e-02	0.999995089	-5.291100e-02	0.9996259
