# Supplementary material for: Systemic Blood Proteome Patterns Reflect Disease Phenotypes in Neovascular Age-Related Macular Degeneration
Source: Int J Mol Sci. 2023 Jun 19;24(12):10327. doi: 10.3390/ijms241210327 (PMC10299094; doi:10.3390/ijms241210327)
Supplement: Supplementary file 1 [file ijms-24-10327-s001.zip › Table S8.pdf]

Suppl. Table. S8_CorrelationCoeffSignifSVM

	Pearson R	Adj. p-val. (2 sided test, Benjamini-H. cor.)
**HBB-HUMAN**	c(cor = 0.387156375830354)	0.99904062373978
**HBA-HUMAN**	c(cor = 0.0469912356350863)	0.99904062373978
**HBG1-HUMAN**	c(cor = 0.157955745493422)	0.99904062373978
**ALBU-HUMAN**	c(cor = -0.40235252416678)	0.99904062373978
**HBD-HUMAN**	c(cor = 0.267551569590014)	0.99904062373978
**IGG1-HUMAN**	c(cor = -0.394268802683467)	0.99904062373978
**CAH1-HUMAN**	c(cor = -0.237273238661242)	0.99904062373978
**PRDX2-HUMAN**	c(cor = 0.121546868784057)	0.99904062373978
**IGLL1-HUMAN**	c(cor = 0.0129123477995402)	0.99904062373978
**IGKC-HUMAN**	c(cor = -0.164093167364994)	0.99904062373978
**APOA1-HUMAN**	c(cor = -0.301053365436796)	0.99904062373978
**TRFE-HUMAN**	c(cor = -0.438650346372733)	0.99904062373978
**FIBB-HUMAN**	c(cor = -0.199307254234097)	0.99904062373978
**FIBG-HUMAN**	c(cor = -0.40998195445516)	0.99904062373978
**A1AT-HUMAN**	c(cor = -0.283846851544192)	0.99904062373978
**IGL1-HUMAN**	c(cor = -0.503901821226149)	0.99904062373978
**IGHG2-HUMAN**	c(cor = -0.423049996318855)	0.99904062373978
**BLVRB-HUMAN**	c(cor = -0.0194061778419403)	0.99904062373978
**IGK-HUMAN**	c(cor = -0.301099823234324)	0.99904062373978
**CD2AP-HUMAN**	c(cor = 0.155976316007266)	0.99904062373978
**CAH2-HUMAN**	c(cor = 0.164650384502133)	0.99904062373978
**FIBA-HUMAN**	c(cor = -0.452353532610202)	0.99904062373978
**B3AT-HUMAN**	c(cor = 0.334405126137004)	0.99904062373978
**IGHA1-HUMAN**	c(cor = -0.179781222098731)	0.99904062373978
**RL11-HUMAN**	c(cor = 0.00710440909770293)	0.99904062373978
**IGLC7-HUMAN**	c(cor = -0.334540622894896)	0.99904062373978
**A2MG-HUMAN**	c(cor = -0.0872485178789517)	0.99904062373978
**CATA-HUMAN**	c(cor = -0.408661469155341)	0.99904062373978
**PLF4-HUMAN**	c(cor = -0.283723963309929)	0.99904062373978
**DEF3-HUMAN**	c(cor = -0.122588043477338)	0.99904062373978
**HPT-HUMAN**	c(cor = -0.399301349611062)	0.99904062373978
**IGLC2-HUMAN;IGLC3-HUMAN**	c(cor = 0.00218459917939126)	0.99904062373978
**APOB-HUMAN**	c(cor = 0.0875957570598987)	0.99904062373978
**CO3-HUMAN**	c(cor = -0.321770256265665)	0.99904062373978
**APOA2-HUMAN**	c(cor = -0.27754457676826)	0.99904062373978
**A1AG1-HUMAN**	c(cor = -0.277189573950929)	0.99904062373978
**ACTB-HUMAN**	c(cor = -0.399607294811159)	0.99904062373978
**PRDX6-HUMAN**	c(cor = -0.211954646049482)	0.99904062373978
**MINP1-HUMAN**	c(cor = -0.112516988615088)	0.99904062373978
**IGM-HUMAN**	c(cor = -0.179078104498959)	0.99904062373978
**TRY1-HUMAN**	c(cor = -0.290739718124264)	0.99904062373978
**G3P-HUMAN**	c(cor = -0.312998719565031)	0.99904062373978
**S10A6-HUMAN**	c(cor = -0.45894750631433)	0.99904062373978
**ACTA-HUMAN;ACTH-HUMAN**	c(cor = -0.264579816578492)	0.99904062373978
**FETUA-HUMAN**	c(cor = -0.343045757192561)	0.99904062373978
**UBB-HUMAN;UBC-HUMAN;RS27A-HUMAN;RL40-HUMAN**	c(cor = -0.42809732230434)	0.99904062373978
**IGA2-HUMAN**	c(cor = -0.296058914779771)	0.99904062373978

**APOA4-HUMAN**	c(cor = -0.30708111360025)	0.99904062373978
**ANK1-HUMAN**	c(cor = 0.0971364252171818)	0.99904062373978
**SPTB1-HUMAN**	c(cor = -0.0499609546330647)	0.99904062373978
**IGHG3-HUMAN**	c(cor = -0.213515160101679)	0.99904062373978
**HEMO-HUMAN**	c(cor = -0.0484127668137968)	0.99904062373978
**PMGE-HUMAN**	c(cor = -0.256595483566693)	0.99904062373978
**APOC3-HUMAN**	c(cor = -0.510523109116954)	0.99904062373978
**EPB42-HUMAN**	c(cor = 0.0193094503318195)	0.99904062373978
**SBP1-HUMAN**	c(cor = 0.306785863164802)	0.99904062373978
**PFD2-HUMAN**	c(cor = 0.024246565165018)	0.99904062373978
**A1AG2-HUMAN**	c(cor = -0.337717412927966)	0.99904062373978
**SPTA1-HUMAN**	c(cor = -0.137283149072925)	0.99904062373978
**HEM2-HUMAN**	c(cor = -0.216732015588859)	0.99904062373978
**VTDB-HUMAN**	c(cor = -0.404952185590639)	0.99904062373978
**ITIH4-HUMAN**	c(cor = -0.28757847566588)	0.99904062373978
**KV228-HUMAN;KVD28-HUMAN**	c(cor = -0.254035646392488)	0.99904062373978
**PRDX1-HUMAN**	c(cor = -0.302413852325867)	0.99904062373978
**RARR2-HUMAN**	c(cor = -0.363654506766461)	0.99904062373978
**KV315-HUMAN**	c(cor = -0.402059102467419)	0.99904062373978
**CO4A-HUMAN**	c(cor = -0.261159037490995)	0.99904062373978
**KVD20-HUMAN**	c(cor = -0.394644405338852)	0.99904062373978
**AACT-HUMAN**	c(cor = -0.227576426640783)	0.99904062373978
**A1BG-HUMAN**	c(cor = -0.0187609204145218)	0.99904062373978
**FINC-HUMAN**	c(cor = -0.259675618736297)	0.99904062373978
**RAN-HUMAN**	c(cor = 0.411221455645501)	0.99904062373978
**KNG1-HUMAN**	c(cor = -0.443135857839535)	0.99904062373978
**C4BPA-HUMAN**	c(cor = -0.216815597753718)	0.99904062373978
**PNPH-HUMAN**	c(cor = -0.153355387827646)	0.99904062373978
**IC1-HUMAN**	c(cor = -0.266994281323197)	0.99904062373978
**VTNC-HUMAN**	c(cor = -0.46804305387849)	0.99904062373978
**GSTO1-HUMAN**	c(cor = -0.00813420036862601)	0.99904062373978
**ANT3-HUMAN**	c(cor = -0.322164359777951)	0.99904062373978
**UB2V1-HUMAN**	c(cor = -0.00310081261054906)	0.99904062373978
**KV320-HUMAN**	c(cor = -0.380163208726325)	0.99904062373978
**MDHC-HUMAN**	c(cor = 0.100185950794702)	0.99904062373978
**NDKB-HUMAN**	c(cor = -0.218468370277447)	0.99904062373978
**PARK7-HUMAN**	c(cor = -0.368098479449817)	0.99904062373978
**HBG2-HUMAN**	c(cor = -0.000537283163822594	0.99904062373978
**ALDOA-HUMAN**	c(cor = 0.156496091327956)	0.99904062373978
**ITIH2-HUMAN**	c(cor = -0.218136468556333)	0.99904062373978
**FCG3A-HUMAN**	c(cor = -0.294033006636289)	0.99904062373978
**AL1A1-HUMAN**	c(cor = -0.402562928783654)	0.99904062373978
**SYUA-HUMAN**	c(cor = -0.018742826505428)	0.99904062373978
**PLMN-HUMAN**	c(cor = -0.435462600349777)	0.99904062373978
**CSN5-HUMAN**	c(cor = -0.236943300023423)	0.99904062373978
**TERA-HUMAN**	c(cor = -0.243163945773213)	0.99904062373978
**GDIB-HUMAN**	c(cor = -0.320491300713591)	0.99904062373978
**LDHA-HUMAN**	c(cor = -0.235916099974491)	0.99904062373978
**LDHB-HUMAN**	c(cor = -0.173397380081993)	0.99904062373978

**S10A4-HUMAN**	c(cor = -0.0918894779462478)	0.99904062373978
**CFAH-HUMAN**	c(cor = -0.38238350374661)	0.99904062373978
**1433B-HUMAN**	c(cor = -0.0831114331448882)	0.99904062373978
**KVD11-HUMAN;KV311-HUMAN**	c(cor = 0.00209510683165828)	0.99904062373978
**KV401-HUMAN**	c(cor = -0.510288276449414)	0.99904062373978
**CFAB-HUMAN**	c(cor = -0.424634299374084)	0.99904062373978
**PPIA-HUMAN**	c(cor = -0.363760920751828)	0.99904062373978
**C1QC-HUMAN**	c(cor = -0.331370113730943)	0.99904062373978
**KAD1-HUMAN**	c(cor = 0.182749343873619)	0.99904062373978
**C1QB-HUMAN**	c(cor = -0.408764543056319)	0.99904062373978
**LV861-HUMAN**	c(cor = -0.14545106191726)	0.99904062373978
**HV118-HUMAN**	c(cor = 0.214659318666929)	0.99904062373978
**TPIS-HUMAN**	c(cor = -0.421663930677376)	0.99904062373978
**HRG-HUMAN**	c(cor = -0.289219787082709)	0.99904062373978
**DEMA-HUMAN**	c(cor = -0.163184654013789)	0.99904062373978
**DOPD-HUMAN**	c(cor = -0.162934995099528)	0.99904062373978
**PGK1-HUMAN**	c(cor = -0.162988204127667)	0.99904062373978
**HV335-HUMAN**	c(cor = -0.24985333363357)	0.99904062373978
**CERU-HUMAN**	c(cor = -0.316724951664106)	0.99904062373978
**UB2Q2-HUMAN**	c(cor = -0.139791640732757)	0.99904062373978
**H2A1-HUMAN;H2A2C-HUMAN;H2A2A-HUMAN;H2A1C-HUM**	c(cor = -0.142621295609009)	0.99904062373978
**APOH-HUMAN**	c(cor = -0.0656437945181799)	0.99904062373978
**S10A8-HUMAN**	c(cor = -0.186856456217331)	0.99904062373978
**PRPS1-HUMAN**	c(cor = -0.324260751889262)	0.99904062373978
**41-HUMAN**	c(cor = -0.179112059321501)	0.99904062373978
**IMB1-HUMAN**	c(cor = -0.0583895378648863)	0.99904062373978
**RINI-HUMAN**	c(cor = -0.0116084176366536)	0.99904062373978
**6PGD-HUMAN**	c(cor = -0.2073177416024)	0.99904062373978
**APOE-HUMAN**	c(cor = -0.279770442901811)	0.99904062373978
**AXA2L-HUMAN;ANXA2-HUMAN**	c(cor = 0.116826374455725)	0.99904062373978
**BIEA-HUMAN**	c(cor = 0.171980180582123)	0.99904062373978
**MIF-HUMAN**	c(cor = 0.185909762618638)	0.99904062373978
**HPRT-HUMAN**	c(cor = -0.0888373059383634)	0.99904062373978
**THRB-HUMAN**	c(cor = -0.461040559993192)	0.99904062373978
**TGM2-HUMAN**	c(cor = 0.0814173245101096)	0.99904062373978
**RD23A-HUMAN**	c(cor = -0.041839232366224)	0.99904062373978
**HV373-HUMAN**	c(cor = -0.374962509742549)	0.99904062373978
**PPAC-HUMAN**	c(cor = 0.00956263634632115)	0.99904062373978
**AFAM-HUMAN**	c(cor = -0.373716582061178)	0.99904062373978
**TPM4-HUMAN**	c(cor = -0.361875698017814)	0.99904062373978
**KVD24-HUMAN;KV224-HUMAN**	c(cor = -0.153273642247007)	0.99904062373978
**UBE2N-HUMAN**	c(cor = -0.214087350032735)	0.99904062373978
**F10A1-HUMAN**	c(cor = -0.0129146780400964)	0.99904062373978
**LGUL-HUMAN**	c(cor = -0.449378998283956)	0.99904062373978
**STOM-HUMAN**	c(cor = 0.2779940957407)	0.99904062373978
**S10A9-HUMAN**	c(cor = -0.102257143938635)	0.99904062373978
**CXCL7-HUMAN**	c(cor = -0.174845164996362)	0.99904062373978
**KV230-HUMAN**	c(cor = -0.0608801917537324)	0.99904062373978
**AIDA-HUMAN**	c(cor = 0.119268615056845)	0.99904062373978

**LV147-HUMAN**	c(cor = -0.348934664441663)	0.99904062373978
**CD5L-HUMAN**	c(cor = -0.247390319782454)	0.99904062373978
**GLO2-HUMAN**	c(cor = -0.224372911997561)	0.99904062373978
**HBAT-HUMAN**	c(cor = -0.399555821802796)	0.99904062373978
**APOC2-HUMAN**	c(cor = -0.541039774572481)	0.770751712688323
**COF1-HUMAN**	c(cor = 0.268149075051628)	0.99904062373978
**SAA4-HUMAN**	c(cor = -0.398409507441332)	0.99904062373978
**GLRX1-HUMAN**	c(cor = 0.266782936347368)	0.99904062373978
**TALDO-HUMAN**	c(cor = -0.113182152351298)	0.99904062373978
**CAN1-HUMAN**	c(cor = -0.0767340821533609)	0.99904062373978
**APOM-HUMAN**	c(cor = -0.254284218934433)	0.99904062373978
**CAH3-HUMAN**	c(cor = 0.164923000427582)	0.99904062373978
**APOD-HUMAN**	c(cor = -0.262651962871881)	0.99904062373978
**C1S-HUMAN**	c(cor = -0.381600328105887)	0.99904062373978
**RS26-HUMAN;RS26L-HUMAN**	c(cor = -0.293124301477235)	0.99904062373978
**HV307-HUMAN**	c(cor = -0.578967750097819)	0.254565471583673
**GPX1-HUMAN**	c(cor = 0.109102433419511)	0.99904062373978
**HV169-HUMAN**	c(cor = -0.190821321611385)	0.99904062373978
**KLKB1-HUMAN**	c(cor = -0.432330760503061)	0.99904062373978
**ARF3-HUMAN;ARF1-HUMAN**	c(cor = -0.359203544795479)	0.99904062373978
**HSP7C-HUMAN**	c(cor = 0.0800026284807712)	0.99904062373978
**HV374-HUMAN**	c(cor = -0.393423110566547)	0.99904062373978
**1433Z-HUMAN**	c(cor = -0.0639963604085912)	0.99904062373978
**PIMT-HUMAN**	c(cor = -0.402449290589518)	0.99904062373978
**ACPH-HUMAN**	c(cor = 0.249451263820902)	0.99904062373978
**IGHG4-HUMAN**	c(cor = -0.318342877819393)	0.99904062373978
**HV309-HUMAN**	c(cor = -0.0871924432554222)	0.99904062373978
**NTF2-HUMAN**	c(cor = 0.0613395138197615)	0.99904062373978
**DDI2-HUMAN**	c(cor = 0.282374404676814)	0.99904062373978
**PGM2-HUMAN**	c(cor = -0.216265719884488)	0.99904062373978
**UBP5-HUMAN**	c(cor = -0.047432542902448)	0.99904062373978
**PSA4-HUMAN**	c(cor = -0.191864678784296)	0.99904062373978
**KVD13-HUMAN;KV113-HUMAN**	c(cor = 0.0645391630295674)	0.99904062373978
**EM55-HUMAN**	c(cor = -0.018183044216059)	0.99904062373978
**ADDB-HUMAN**	c(cor = -0.33293921437672)	0.99904062373978
**GELS-HUMAN**	c(cor = -0.320552765690063)	0.99904062373978
**APOC1-HUMAN**	c(cor = -0.450118371285276)	0.99904062373978
**LV319-HUMAN**	c(cor = -0.0700963930018632)	0.99904062373978
**PROS-HUMAN**	c(cor = -0.470681968492168)	0.99904062373978
**CFAI-HUMAN**	c(cor = -0.349178670460636)	0.99904062373978
**THIO-HUMAN**	c(cor = -0.239888704125278)	0.99904062373978
**CCS-HUMAN**	c(cor = -0.0833428710651788)	0.99904062373978
**ITIH1-HUMAN**	c(cor = -0.359698540683298)	0.99904062373978
**NCEH1-HUMAN**	c(cor = 0.114713737019647)	0.99904062373978
**KV112-HUMAN;KVD12-HUMAN**	c(cor = -0.168687486611416)	0.99904062373978
**HV434-HUMAN**	c(cor = -0.141684966561163)	0.99904062373978
**PEBP1-HUMAN**	c(cor = -0.403753147071288)	0.99904062373978
**TCPQ-HUMAN**	c(cor = -0.343121490093448)	0.99904062373978
**CAND1-HUMAN**	c(cor = -0.363286702958541)	0.99904062373978

**1433E-HUMAN**	c(cor = -0.0104376132842813)	0.99904062373978
**A2AP-HUMAN**	c(cor = -0.483823714281916)	0.99904062373978
**SORCN-HUMAN**	c(cor = 0.0205983587491761)	0.99904062373978
**SAHH-HUMAN**	c(cor = 0.0894291575595227)	0.99904062373978
**SODC-HUMAN**	c(cor = -0.231996610534816)	0.99904062373978
**HARS1-HUMAN**	c(cor = -0.100836068393577)	0.99904062373978
**CPNS1-HUMAN**	c(cor = -0.226085242115978)	0.99904062373978
**HEP2-HUMAN**	c(cor = -0.428042981634609)	0.99904062373978
**TYB4-HUMAN**	c(cor = -0.120655469260637)	0.99904062373978
**LUM-HUMAN**	c(cor = -0.325202771697278)	0.99904062373978
**CALM1-HUMAN;CALM2-HUMAN;CALM3-HUMAN**	c(cor = -0.318074409817839)	0.99904062373978
**PTPRJ-HUMAN**	c(cor = -0.390426526192669)	0.99904062373978
**HEM3-HUMAN**	c(cor = -0.0999344358211752)	0.99904062373978
**IGHM-HUMAN**	c(cor = -0.237168334054245)	0.99904062373978
**ANGT-HUMAN**	c(cor = -0.0500242146503892)	0.99904062373978
**ICAL-HUMAN**	c(cor = 0.000207731534602111)	0.99904062373978
**TRFL-HUMAN**	c(cor = -0.0490314204991952)	0.99904062373978
**FKB1A-HUMAN**	c(cor = -0.175347082118163)	0.99904062373978
**PSMF1-HUMAN**	c(cor = 0.00267240378528711)	0.99904062373978
**HYAS1-HUMAN**	c(cor = -0.181328511929474)	0.99904062373978
**CO5-HUMAN**	c(cor = -0.349048642986455)	0.99904062373978
**PTPA-HUMAN**	c(cor = -0.168460627144703)	0.99904062373978
**HS71A-HUMAN;HS71B-HUMAN**	c(cor = -0.297924572966214)	0.99904062373978
**FHR3-HUMAN**	c(cor = -0.256996234312156)	0.99904062373978
**APOA-HUMAN**	c(cor = -0.302574303232259)	0.99904062373978
**CO4B-HUMAN**	c(cor = -0.45921253716142)	0.99904062373978
**KPYR-HUMAN**	c(cor = -0.0287701668534052)	0.99904062373978
**PSME1-HUMAN**	c(cor = -0.487631214824571)	0.99904062373978
**C1QA-HUMAN**	c(cor = -0.287993170990251)	0.99904062373978
**TSP1-HUMAN**	c(cor = -0.0930095931755415)	0.99904062373978
**FBX7-HUMAN**	c(cor = -0.165829654153964)	0.99904062373978
**HPTR-HUMAN**	c(cor = 0.0780307375597918)	0.99904062373978
**ADDA-HUMAN**	c(cor = -0.336497575203709)	0.99904062373978
**PITH1-HUMAN**	c(cor = -0.202160546732296)	0.99904062373978
**LV144-HUMAN**	c(cor = -0.0706996676818565)	0.99904062373978
**TCPG-HUMAN**	c(cor = 0.0688373780412744)	0.99904062373978
**STIP1-HUMAN**	c(cor = 0.00842445312211781)	0.99904062373978
**DCUP-HUMAN**	c(cor = -0.066825396937526)	0.99904062373978
**AHSP-HUMAN**	c(cor = -0.181459310178098)	0.99904062373978
**PSA1-HUMAN**	c(cor = -0.23252778521397)	0.99904062373978
**FHR1-HUMAN**	c(cor = -0.322559257841844)	0.99904062373978
**ENOA-HUMAN**	c(cor = -0.273496229724067)	0.99904062373978
**CLIC1-HUMAN**	c(cor = -0.316062167042301)	0.99904062373978
**IGJ-HUMAN**	c(cor = -0.238144166383918)	0.99904062373978
**BMP1-HUMAN;TLL2-HUMAN**	c(cor = 0.104198653904855)	0.99904062373978
**APOL1-HUMAN**	c(cor = -0.511693281706841)	0.99904062373978
**QCR7-HUMAN**	c(cor = -0.0905667044148587)	0.99904062373978
**TCPZ-HUMAN**	c(cor = 0.00412991093548457)	0.99904062373978
**UBP14-HUMAN**	c(cor = -0.174736362951607)	0.99904062373978

FCL-HUMAN	c(cor = -0.320445254945975)	0.99904062373978
2AAA-HUMAN	c(cor = -0.387459596355859)	0.99904062373978
CLUS-HUMAN	c(cor = -0.483462913081415)	0.99904062373978
GSHR-HUMAN	c(cor = 0.253042419172245)	0.99904062373978
IF5A1-HUMAN	c(cor = -0.145678152485257)	0.99904062373978
TCPE-HUMAN	c(cor = -0.334018539834914)	0.99904062373978
FLNA-HUMAN	c(cor = -0.264483669886454)	0.99904062373978
HEBP1-HUMAN	c(cor = -0.413148258561319)	0.99904062373978
PSA7-HUMAN	c(cor = -0.107738132418281)	0.99904062373978
PA1B3-HUMAN	c(cor = -0.183214889104441)	0.99904062373978
TCPB-HUMAN	c(cor = -0.146929156917145)	0.99904062373978
SKP1-HUMAN	c(cor = -0.0312359536431004)	0.99904062373978
LV39-HUMAN	c(cor = -0.438170696263912)	0.99904062373978
NDKA-HUMAN	c(cor = -0.191579232056095)	0.99904062373978
TCPH-HUMAN	c(cor = -0.169980763238045)	0.99904062373978
TXNL1-HUMAN	c(cor = 0.261841933412438)	0.99904062373978
GSTP1-HUMAN	c(cor = -0.150770746530722)	0.99904062373978
HS90B-HUMAN	c(cor = -0.289229126918585)	0.99904062373978
HV5X1-HUMAN	c(cor = -0.212531521458288)	0.99904062373978
RPIA-HUMAN	c(cor = -0.338875146224814)	0.99904062373978
RET4-HUMAN	c(cor = -0.495493153559297)	0.99904062373978
BOLA2-HUMAN	c(cor = -0.0255501115998549)	0.99904062373978
H2B1K-HUMAN;H2B1J-HUMAN;H2B1O-HUMAN;H2B1B-HU	c(cor = 0.104699181823662)	0.99904062373978
FA12-HUMAN	c(cor = -0.120588759738806)	0.99904062373978
IF4A1-HUMAN	c(cor = 0.18433068938471)	0.99904062373978
SHPS1-HUMAN;SIRBL-HUMAN;SIRPG-HUMAN	c(cor = -0.277368195917318)	0.99904062373978
UBA1-HUMAN	c(cor = -0.242959408557873)	0.99904062373978
SH3L3-HUMAN	c(cor = -0.126754828778694)	0.99904062373978
PNCB-HUMAN	c(cor = -0.246590458933576)	0.99904062373978
GTR1-HUMAN	c(cor = -0.0206490957534335)	0.99904062373978
PSB2-HUMAN	c(cor = -0.212071617737876)	0.99904062373978
RAC1-HUMAN	c(cor = -0.365838641930562)	0.99904062373978
CO7-HUMAN	c(cor = -0.140447503034074)	0.99904062373978
PUR6-HUMAN	c(cor = -0.258132953227639)	0.99904062373978
ACLY-HUMAN	c(cor = -0.186118220678333)	0.99904062373978
WDR1-HUMAN	c(cor = -0.310198028919002)	0.99904062373978
HS90A-HUMAN	c(cor = -0.207880287180586)	0.99904062373978
CO9-HUMAN	c(cor = -0.385697174756609)	0.99904062373978
LV310-HUMAN	c(cor = -0.0855101881702172)	0.99904062373978
TPM3-HUMAN	c(cor = -0.192628837578008)	0.99904062373978
PSA5-HUMAN	c(cor = -0.306208680957417)	0.99904062373978
RPE-HUMAN	c(cor = -0.466313818237304)	0.99904062373978
PLSL-HUMAN	c(cor = -0.12279871537045)	0.99904062373978
DAZP1-HUMAN	c(cor = -0.329653494176232)	0.99904062373978
THTM-HUMAN	c(cor = -0.297191117808386)	0.99904062373978
OLA1-HUMAN	c(cor = -0.161040849530169)	0.99904062373978
TAGL2-HUMAN	c(cor = -0.271912761381624)	0.99904062373978
TBCA-HUMAN	c(cor = -0.493849814206698)	0.99904062373978
PSB1-HUMAN	c(cor = -0.18390973485708)	0.99904062373978

**CO2-HUMAN**	c(cor = -0.460019119491325)	0.99904062373978
**SUMO3-HUMAN;SUMO2-HUMAN;SUMO4-HUMAN**	c(cor = -0.10261158139321)	0.99904062373978
**HV551-HUMAN**	c(cor = -0.247822464299069)	0.99904062373978
**PSMD9-HUMAN**	c(cor = 0.181827387862851)	0.99904062373978
**PGRP2-HUMAN**	c(cor = -0.354551926792814)	0.99904062373978
**AMBP-HUMAN**	c(cor = -0.152244569883228)	0.99904062373978
**ESTD-HUMAN**	c(cor = -0.0201694215230899)	0.99904062373978
**TCPD-HUMAN**	c(cor = -0.256767165950372)	0.99904062373978
**ELNE-HUMAN**	c(cor = 0.0749445791752602)	0.99904062373978
**NSF1C-HUMAN**	c(cor = -0.215510474617908)	0.99904062373978
**TTHY-HUMAN**	c(cor = -0.439131928402155)	0.99904062373978
**PUR9-HUMAN**	c(cor = 0.0608712666515361)	0.99904062373978
**GLOD4-HUMAN**	c(cor = 0.0718769027101117)	0.99904062373978
**AL1L1-HUMAN**	c(cor = -0.0823782906248872)	0.99904062373978
**IGD-HUMAN**	c(cor = -0.161374421264072)	0.99904062373978
**TKT-HUMAN**	c(cor = -0.00736791963357074)	0.99904062373978
**TLN1-HUMAN**	c(cor = -0.27473082481559)	0.99904062373978
**CYTB-HUMAN**	c(cor = 0.183219504320643)	0.99904062373978
**LIMS1-HUMAN**	c(cor = -0.248399438963908)	0.99904062373978
**ENOG-HUMAN**	c(cor = -0.0641923537918932)	0.99904062373978
**PRTN3-HUMAN**	c(cor = 0.101400585912253)	0.99904062373978
**A2GL-HUMAN**	c(cor = -0.176966651760694)	0.99904062373978
**AATC-HUMAN**	c(cor = -0.0642993253450529)	0.99904062373978
**GSH1-HUMAN**	c(cor = -0.367587582936034)	0.99904062373978
**HBE-HUMAN**	c(cor = 0.151274997875797)	0.99904062373978
**LV325-HUMAN**	c(cor = -0.306636154511568)	0.99904062373978
**HINT1-HUMAN**	c(cor = 0.00658548168756516)	0.99904062373978
**KVD15-HUMAN**	c(cor = -0.0896535134357702)	0.99904062373978
**CO6-HUMAN**	c(cor = -0.052463650279389)	0.99904062373978
**SH3L1-HUMAN**	c(cor = -0.1773379319543)	0.99904062373978
**HV330-HUMAN;HVC05-HUMAN**	c(cor = -0.403585310762128)	0.99904062373978
**CO8A-HUMAN**	c(cor = -0.412667109258756)	0.99904062373978
**URP2-HUMAN**	c(cor = -0.273839933720047)	0.99904062373978
**HV601-HUMAN**	c(cor = -0.235845328644741)	0.99904062373978
**UBAC1-HUMAN**	c(cor = -0.199140221474445)	0.99904062373978
**LV151-HUMAN**	c(cor = -0.133700179199573)	0.99904062373978
**HEM4-HUMAN**	c(cor = -0.318870289846193)	0.99904062373978
**PSB4-HUMAN**	c(cor = -0.0655710571246436)	0.99904062373978
**TEBP-HUMAN**	c(cor = -0.0320853159073206)	0.99904062373978
**PRS4-HUMAN**	c(cor = -0.0544133214602397)	0.99904062373978
**ZA2G-HUMAN**	c(cor = -0.39036926887284)	0.99904062373978
**HV70D-HUMAN**	c(cor = -0.141667406553275)	0.99904062373978
**MOES-HUMAN**	c(cor = -0.205605528528735)	0.99904062373978
**APMAP-HUMAN**	c(cor = -0.00640159151112996)	0.99904062373978
**SNAA-HUMAN**	c(cor = -0.52816280240322)	0.99904062373978
**FLOT1-HUMAN**	c(cor = 0.0256001546486644)	0.99904062373978
**PSA6-HUMAN**	c(cor = -0.407679072103545)	0.99904062373978
**CUTA-HUMAN**	c(cor = 0.237301657827889)	0.99904062373978
**PSA3-HUMAN**	c(cor = -0.324422283391102)	0.99904062373978

**UB2D3-HUMAN;UB2D2-HUMAN**	c(cor = -0.329659748090452)	0.99904062373978
**ANM5-HUMAN**	c(cor = -0.354065015002484)	0.99904062373978
**LV743-HUMAN**	c(cor = -0.268957524005868)	0.99904062373978
**ITA2B-HUMAN**	c(cor = -0.033326808494776)	0.99904062373978
**SEPP1-HUMAN**	c(cor = -0.466321008647134)	0.99904062373978
**CATG-HUMAN**	c(cor = 0.077901401011126)	0.99904062373978
**UB2L3-HUMAN**	c(cor = -0.178637232753913)	0.99904062373978
**LKHA4-HUMAN**	c(cor = -0.0220756852960116)	0.99904062373978
**CO8G-HUMAN**	c(cor = -0.180348484937147)	0.99904062373978
**LXN-HUMAN**	c(cor = 0.308247896744752)	0.99904062373978
**H4-HUMAN**	c(cor = -0.0420830263020282)	0.99904062373978
**TCPA-HUMAN**	c(cor = -0.280206301077964)	0.99904062373978
**PERM-HUMAN**	c(cor = -0.0859446186929751)	0.99904062373978
**DPP3-HUMAN**	c(cor = 0.0242353955041995)	0.99904062373978
**IDHC-HUMAN**	c(cor = -0.144141338346935)	0.99904062373978
**MCTS1-HUMAN**	c(cor = 0.0167438701910554)	0.99904062373978
**RAP1A-HUMAN**	c(cor = 0.105537512163604)	0.99904062373978
**HV432-HUMAN**	c(cor = 0.138653262461609)	0.99904062373978
**CHSP1-HUMAN**	c(cor = -0.239271723224266)	0.99904062373978
**HBM-HUMAN**	c(cor = 0.0679184360528276)	0.99904062373978
**K2C6A-HUMAN**	c(cor = 0.0624399692513013)	0.99904062373978
**CAZA1-HUMAN**	c(cor = 0.00777392003848261)	0.99904062373978
**ANXA7-HUMAN**	c(cor = -0.186635407488119)	0.99904062373978
**HV338-HUMAN**	c(cor = -0.27945121319602)	0.99904062373978
**G6PD-HUMAN**	c(cor = -0.14661615526887)	0.99904062373978
**G6PI-HUMAN**	c(cor = 0.26578802860576)	0.99904062373978
**OTU1-HUMAN**	c(cor = 0.0931491236752187)	0.99904062373978
**DNPH1-HUMAN**	c(cor = -0.0320732204243913)	0.99904062373978
**OXSR1-HUMAN**	c(cor = -0.10284748200097)	0.99904062373978
**PSME2-HUMAN**	c(cor = -0.278432895895658)	0.99904062373978
**KV139-HUMAN;KVD39-HUMAN**	c(cor = -0.268425202541189)	0.99904062373978
**RHOA-HUMAN**	c(cor = -0.308577452645864)	0.99904062373978
**MYL6-HUMAN**	c(cor = -0.190861498622489)	0.99904062373978
**CPN2-HUMAN**	c(cor = -0.180796327169804)	0.99904062373978
**PSB6-HUMAN**	c(cor = -0.232367457853223)	0.99904062373978
**GSHB-HUMAN**	c(cor = -0.354946201130595)	0.99904062373978
**FAAA-HUMAN**	c(cor = 0.103679620293189)	0.99904062373978
**UBE2K-HUMAN**	c(cor = 0.0791403633690516)	0.99904062373978
**MYH9-HUMAN**	c(cor = -0.424350756550681)	0.99904062373978
**PI42A-HUMAN**	c(cor = -0.161231395130417)	0.99904062373978
**C1RL-HUMAN**	c(cor = -0.378598235952472)	0.99904062373978
**RANG-HUMAN**	c(cor = -0.400317237549196)	0.99904062373978
**THIC-HUMAN**	c(cor = -0.0947179718836678)	0.99904062373978
**DCXR-HUMAN**	c(cor = -0.4213575270303)	0.99904062373978
**PA1B2-HUMAN**	c(cor = 0.0300994154764218)	0.99904062373978
**KPYM-HUMAN**	c(cor = -0.258806434873098)	0.99904062373978
**HABP2-HUMAN**	c(cor = -0.376729254186649)	0.99904062373978
**UBE2O-HUMAN**	c(cor = -0.0858973479107974)	0.99904062373978
**VINC-HUMAN**	c(cor = -0.208952575416141)	0.99904062373978

**PP2AA-HUMAN**	c(cor = 0.0163262866998799)	0.99904062373978
**ALDOC-HUMAN**	c(cor = 0.656739843829011)	0.0160853389685454
**PRPS2-HUMAN**	c(cor = -0.0265081202863347)	0.99904062373978
**PON1-HUMAN**	c(cor = -0.100928539107027)	0.99904062373978
**HGFL-HUMAN**	c(cor = -0.2519189444962)	0.99904062373978
**GPX3-HUMAN**	c(cor = -0.0262182607771088)	0.99904062373978
**RLA2-HUMAN**	c(cor = -0.196400548582742)	0.99904062373978
**PSB5-HUMAN**	c(cor = -0.192007563474267)	0.99904062373978
**H14-HUMAN**	c(cor = 0.122897596466173)	0.99904062373978
**CO8B-HUMAN**	c(cor = -0.467922993379384)	0.99904062373978
**6PGL-HUMAN**	c(cor = 0.0655930739180926)	0.99904062373978
**CBPB2-HUMAN**	c(cor = -0.236926409639231)	0.99904062373978
**HV64D-HUMAN**	c(cor = -0.381289216807031)	0.99904062373978
**TETN-HUMAN**	c(cor = -0.294791101662748)	0.99904062373978
**GLPC-HUMAN**	c(cor = 0.127786063436981)	0.99904062373978
**PEDF-HUMAN**	c(cor = -0.486000647173624)	0.99904062373978
**H31-HUMAN;H31T-HUMAN;H32-HUMAN**	c(cor = -0.252519010782087)	0.99904062373978
**UBP15-HUMAN**	c(cor = -0.217584346030441)	0.99904062373978
**GSH0-HUMAN**	c(cor = 0.139762508389916)	0.99904062373978
**PRS7-HUMAN**	c(cor = -0.0281305382187784)	0.99904062373978
**PGBM-HUMAN**	c(cor = 0.155254029417778)	0.99904062373978
**PGAM1-HUMAN**	c(cor = -0.365973152876155)	0.99904062373978
**CSN4-HUMAN**	c(cor = -0.208302549100994)	0.99904062373978
**PSB7-HUMAN**	c(cor = -0.345158990093087)	0.99904062373978
**NP1L4-HUMAN**	c(cor = -0.203900016977945)	0.99904062373978
**KV108-HUMAN**	c(cor = -0.428381068844003)	0.99904062373978
**FN3K-HUMAN**	c(cor = -0.0750088405748896)	0.99904062373978
**HV313-HUMAN**	c(cor = -0.21499828565829)	0.99904062373978
**ACTN1-HUMAN**	c(cor = -0.282294649379241)	0.99904062373978
**HV404-HUMAN**	c(cor = -0.234348123133739)	0.99904062373978
**RAB1B-HUMAN**	c(cor = 0.00791401960845732)	0.99904062373978
**ANXA3-HUMAN**	c(cor = 0.0109968544534973)	0.99904062373978
**AP2S1-HUMAN**	c(cor = -0.107664561823162)	0.99904062373978
**LYSC-HUMAN**	c(cor = -0.402465930426318)	0.99904062373978
**CAP1-HUMAN**	c(cor = -0.0196946224095917)	0.99904062373978
**XPO7-HUMAN**	c(cor = 0.250978715907788)	0.99904062373978
**GMPR1-HUMAN**	c(cor = 0.184521556675074)	0.99904062373978
**THBG-HUMAN**	c(cor = -0.364797300449616)	0.99904062373978
**NLTP-HUMAN**	c(cor = -0.0785759545369538)	0.99904062373978
**PEPD-HUMAN**	c(cor = 0.134728558060981)	0.99904062373978
**RN123-HUMAN**	c(cor = -0.16987773334787)	0.99904062373978
**RAB5B-HUMAN**	c(cor = -0.488927849268104)	0.99904062373978
**KCY-HUMAN**	c(cor = 0.0563416822097104)	0.99904062373978
**CBPN-HUMAN**	c(cor = -0.0699969180067188)	0.99904062373978
**PRDX5-HUMAN**	c(cor = -0.0927283766756957)	0.99904062373978
**TNG2-HUMAN**	c(cor = -0.216319518946266)	0.99904062373978
**ABHEB-HUMAN**	c(cor = -0.494722153906044)	0.99904062373978
**PA2G4-HUMAN**	c(cor = 0.169666645420311)	0.99904062373978
**CD47-HUMAN**	c(cor = -0.153543913623171)	0.99904062373978

**TMOD1-HUMAN**	c(cor = -0.225555046802238)	0.99904062373978
**TBA1B-HUMAN**	c(cor = -0.0545557731931362)	0.99904062373978
**C1R-HUMAN**	c(cor = -0.355032602904887)	0.99904062373978
**KVD33-HUMAN;KV133-HUMAN**	c(cor = -0.211091140419182)	0.99904062373978
**TBB4B-HUMAN**	c(cor = -0.206111396113542)	0.99904062373978
**PRS6A-HUMAN**	c(cor = -0.0927631776572972)	0.99904062373978
**ARPC5-HUMAN**	c(cor = -0.0790680671393907)	0.99904062373978
**ETFA-HUMAN**	c(cor = 0.015721055365686)	0.99904062373978
**UCHL3-HUMAN**	c(cor = -0.029388204200723)	0.99904062373978
**APT-HUMAN**	c(cor = -0.229103202274426)	0.99904062373978
**FA10-HUMAN**	c(cor = -0.513396197908733)	0.99904062373978
**PCBP1-HUMAN**	c(cor = -0.0459484976727032)	0.99904062373978
**F13A-HUMAN**	c(cor = -0.391206825491533)	0.99904062373978
**DHPR-HUMAN**	c(cor = -0.373641458620894)	0.99904062373978
**ITIH3-HUMAN**	c(cor = -0.0627288426784536)	0.99904062373978
**WBP2-HUMAN**	c(cor = -0.384092907896701)	0.99904062373978
**ARGI1-HUMAN**	c(cor = 0.0499757185699023)	0.99904062373978
**IF6-HUMAN**	c(cor = -0.030507345585213)	0.99904062373978
**TPP2-HUMAN**	c(cor = -0.268242782228425)	0.99904062373978
**PSMD5-HUMAN**	c(cor = -0.0805336705765636)	0.99904062373978
**ARK72-HUMAN**	c(cor = -0.490142798576198)	0.99904062373978
**ML12B-HUMAN;ML12A-HUMAN**	c(cor = -0.186038833833653)	0.99904062373978
**GGCT-HUMAN**	c(cor = -0.396359014409015)	0.99904062373978
**GET3-HUMAN**	c(cor = -0.334320918010683)	0.99904062373978
**CBR1-HUMAN**	c(cor = -0.0754856246713125)	0.99904062373978
**PUR4-HUMAN**	c(cor = -0.188640975252133)	0.99904062373978
**SAMP-HUMAN**	c(cor = -0.575117065894326)	0.286502559342318
**HV372-HUMAN**	c(cor = -0.206901895638674)	0.99904062373978
**CSN1-HUMAN**	c(cor = -0.0763436387937446)	0.99904062373978
**FHR2-HUMAN**	c(cor = -0.341547761295677)	0.99904062373978
**TPM1-HUMAN**	c(cor = -0.0683209031085074)	0.99904062373978
**PFKAL-HUMAN**	c(cor = -0.369611739712701)	0.99904062373978
**HSP74-HUMAN**	c(cor = -0.369800783854117)	0.99904062373978
**KPRB-HUMAN**	c(cor = -0.312065340449041)	0.99904062373978
**FA9-HUMAN**	c(cor = -0.325543557577788)	0.99904062373978
**PGP-HUMAN**	c(cor = 0.136992751178258)	0.99904062373978
**KRT83-HUMAN**	c(cor = -0.248152304999965)	0.99904062373978
**TENA-HUMAN**	c(cor = -0.092193317895074)	0.99904062373978
**PUR8-HUMAN**	c(cor = -0.17083993166062)	0.99904062373978
**CT027-HUMAN**	c(cor = -0.350939722363401)	0.99904062373978
**S14L4-HUMAN**	c(cor = 0.0668697718752585)	0.99904062373978
**CBG-HUMAN**	c(cor = -0.503395971679801)	0.99904062373978
**CYBR1-HUMAN**	c(cor = -0.276567694906565)	0.99904062373978
**LEG3-HUMAN**	c(cor = 0.151447345191993)	0.99904062373978
**DCNL1-HUMAN**	c(cor = 0.143467511474356)	0.99904062373978
**ICLN-HUMAN**	c(cor = 0.0684875627290644)	0.99904062373978
**1433G-HUMAN**	c(cor = 0.149062502321077)	0.99904062373978
**RD23B-HUMAN**	c(cor = -0.138632414577717)	0.99904062373978
**FHR4-HUMAN**	c(cor = -0.111002985025463)	0.99904062373978

**FLOT2-HUMAN**	c(cor = -0.117240762311611)	0.99904062373978
**KV137-HUMAN;KVD37-HUMAN**	c(cor = -0.405151118117525)	0.99904062373978
**FCN3-HUMAN**	c(cor = -0.407388211755151)	0.99904062373978
**UB2V2-HUMAN**	c(cor = -0.0148362914783214)	0.99904062373978
**1433T-HUMAN**	c(cor = -0.366520137317794)	0.99904062373978
**RB11A-HUMAN;RB11B-HUMAN**	c(cor = 0.0619140720708263)	0.99904062373978
**TBA1A-HUMAN**	c(cor = -0.199438559447092)	0.99904062373978
**LIS1-HUMAN**	c(cor = -0.0271057608251226)	0.99904062373978
**AL9A1-HUMAN**	c(cor = -0.104949739823729)	0.99904062373978
**RUVB2-HUMAN**	c(cor = -0.266493410644738)	0.99904062373978
**TTL12-HUMAN**	c(cor = -0.150507547323489)	0.99904062373978
**PPME1-HUMAN**	c(cor = -0.166164103579505)	0.99904062373978
**SIA10-HUMAN**	c(cor = -0.252161581395082)	0.99904062373978
**CLH1-HUMAN**	c(cor = -0.196173899421863)	0.99904062373978
**PCBP2-HUMAN**	c(cor = -0.029881763274798)	0.99904062373978
**ACTZ-HUMAN**	c(cor = -0.0621661172369772)	0.99904062373978
**MTAP-HUMAN**	c(cor = -0.187227417244101)	0.99904062373978
**PP1R7-HUMAN**	c(cor = 0.185705704314268)	0.99904062373978
**HV102-HUMAN**	c(cor = -0.101613457948729)	0.99904062373978
**NSF-HUMAN**	c(cor = -0.282710518978502)	0.99904062373978
**DESP-HUMAN**	c(cor = -0.302953644671378)	0.99904062373978
**KAIN-HUMAN**	c(cor = -0.343810955869735)	0.99904062373978
**FETUB-HUMAN**	c(cor = -0.427470048293603)	0.99904062373978
**TBA4A-HUMAN**	c(cor = -0.0529283273406734)	0.99904062373978
**PERE-HUMAN**	c(cor = 0.0483807559973571)	0.99904062373978
**H15-HUMAN**	c(cor = -0.15168622250146)	0.99904062373978
**VATA-HUMAN**	c(cor = -0.149420989107704)	0.99904062373978
**GDIR2-HUMAN**	c(cor = -0.372330196162407)	0.99904062373978
**VCAM1-HUMAN**	c(cor = -0.372840961445654)	0.99904062373978
**A16A1-HUMAN**	c(cor = -0.268616416960995)	0.99904062373978
**PTGDS-HUMAN**	c(cor = -0.0871851095959352)	0.99904062373978
**PSB3-HUMAN**	c(cor = -0.333351933607403)	0.99904062373978
**UGPA-HUMAN**	c(cor = 0.000891213461395714)	0.99904062373978
**PLEK-HUMAN**	c(cor = -0.235664467246484)	0.99904062373978
**PSA2-HUMAN**	c(cor = 0.0354924877916789)	0.99904062373978
**HV428-HUMAN**	c(cor = -0.280823655105225)	0.99904062373978
**IPO9-HUMAN**	c(cor = -0.169793960492186)	0.99904062373978
**GLRX3-HUMAN**	c(cor = -0.0162374673071272)	0.99904062373978
**TXD17-HUMAN**	c(cor = -0.20190722101253)	0.99904062373978
**CD59-HUMAN**	c(cor = -0.381331236768029)	0.99904062373978
**FSCN1-HUMAN**	c(cor = -0.16573504791785)	0.99904062373978
**RIDA-HUMAN**	c(cor = -0.15513833652679)	0.99904062373978
**LANC1-HUMAN**	c(cor = -0.190888421174082)	0.99904062373978
**RHCE-HUMAN**	c(cor = -0.0206642685800708)	0.99904062373978
**PDC6I-HUMAN**	c(cor = -0.110674415322267)	0.99904062373978
**KJ01-HUMAN**	c(cor = -0.255588448956059)	0.99904062373978
**FBLN1-HUMAN**	c(cor = -0.236778722033336)	0.99904062373978
**HV364-HUMAN**	c(cor = -0.331044691160793)	0.99904062373978
**PSDE-HUMAN**	c(cor = -0.186984477213872)	0.99904062373978

**VWF-HUMAN**	c(cor = -0.338581288052595)	0.99904062373978
**CAPZB-HUMAN**	c(cor = -0.110597099284913)	0.99904062373978
**PHLD-HUMAN**	c(cor = -0.35943048086855)	0.99904062373978
**PPIL3-HUMAN**	c(cor = -0.320956482789605)	0.99904062373978
**KV117-HUMAN**	c(cor = -0.301624348006138)	0.99904062373978
**RASK-HUMAN**	c(cor = -0.225466521901195)	0.99904062373978
**LV208-HUMAN**	c(cor = -0.388684614761983)	0.99904062373978
**IF4B-HUMAN**	c(cor = -0.0255050643043265)	0.99904062373978
**PGM2L-HUMAN**	c(cor = 0.0689653669922147)	0.99904062373978
**2AAB-HUMAN**	c(cor = -0.277067812690912)	0.99904062373978
**PRS10-HUMAN**	c(cor = -0.132859295602032)	0.99904062373978
**DYN2-HUMAN**	c(cor = -0.190217436727349)	0.99904062373978
**CYB5-HUMAN**	c(cor = -0.0605608031344107)	0.99904062373978
**GSTM3-HUMAN**	c(cor = -0.373273039981271)	0.99904062373978
**NB5R3-HUMAN**	c(cor = 0.0864187808624429)	0.99904062373978
**PROF1-HUMAN**	c(cor = -0.238320776695924)	0.99904062373978
**HV124-HUMAN**	c(cor = -0.250812838871242)	0.99904062373978
**ADT2-HUMAN;ADT1-HUMAN;ADT3-HUMAN;ADT4-HUMAN**	c(cor = -0.320814175464798)	0.99904062373978
**LV949-HUMAN**	c(cor = -0.00604602404301392)	0.99904062373978
**ANXA1-HUMAN**	c(cor = 0.0136573497892362)	0.99904062373978
**IF2G-HUMAN**	c(cor = -0.152077204511186)	0.99904062373978
**BROX-HUMAN**	c(cor = -0.560648718652129)	0.441596537440559
**CNRP1-HUMAN**	c(cor = -0.198515271886752)	0.99904062373978
**MGST2-HUMAN**	c(cor = 0.374269522506171)	0.99904062373978
**PRG2-HUMAN**	c(cor = -0.185939045103309)	0.99904062373978
**FABP5-HUMAN**	c(cor = -0.368443641349382)	0.99904062373978
**IPYR-HUMAN**	c(cor = -0.0217123228600787)	0.99904062373978
**KVD16-HUMAN**	c(cor = -0.122714157797461)	0.99904062373978
**MISSL-HUMAN**	c(cor = 0.365961260803399)	0.99904062373978
**CUL3-HUMAN**	c(cor = -0.0543729329678761)	0.99904062373978
**DDB1-HUMAN**	c(cor = -0.135819227482431)	0.99904062373978
**NGAL-HUMAN**	c(cor = -0.293381595670384)	0.99904062373978
**RAB10-HUMAN**	c(cor = 0.035289308996156)	0.99904062373978
**IMA4-HUMAN**	c(cor = 0.106109689048119)	0.99904062373978
**LV211-HUMAN**	c(cor = -0.39077991807372)	0.99904062373978
**IGF2-HUMAN**	c(cor = -0.422571442434818)	0.99904062373978
**RAP2B-HUMAN**	c(cor = 0.0309732940527564)	0.99904062373978
**PSMD6-HUMAN**	c(cor = -0.130902175688503)	0.99904062373978
**TBB5-HUMAN**	c(cor = -0.0706577189829394)	0.99904062373978
**RAB35-HUMAN**	c(cor = 0.20921420165119)	0.99904062373978
**KV240-HUMAN;KVD40-HUMAN**	c(cor = -0.310443748352252)	0.99904062373978
**LASP1-HUMAN**	c(cor = -0.414628867546656)	0.99904062373978
**PSMD1-HUMAN**	c(cor = -0.171984426726147)	0.99904062373978
**CI040-HUMAN**	c(cor = -0.00302244508440233)	0.99904062373978
**MIEN1-HUMAN**	c(cor = -0.199536017279869)	0.99904062373978
**KVD08-HUMAN**	c(cor = -0.111192954086717)	0.99904062373978
**TBB1-HUMAN**	c(cor = 0.0547927592794664)	0.99904062373978
**EHD1-HUMAN**	c(cor = -0.246598069294115)	0.99904062373978
**MEP50-HUMAN**	c(cor = 0.0820088091448433)	0.99904062373978

**PRS8-HUMAN**	c(cor = -0.213009871308445)	0.99904062373978
**HV343-HUMAN**	c(cor = -0.0706477778574204)	0.99904062373978
**COR1A-HUMAN**	c(cor = -0.0582112509226437)	0.99904062373978
**GBB1-HUMAN**	c(cor = -0.34170682252684)	0.99904062373978
**RSU1-HUMAN**	c(cor = -0.154340979573033)	0.99904062373978
**UFC1-HUMAN**	c(cor = -0.219707372813658)	0.99904062373978
**FA5-HUMAN**	c(cor = -0.418560696893934)	0.99904062373978
**SRXN1-HUMAN**	c(cor = -0.158827185003157)	0.99904062373978
**ABRAL-HUMAN**	c(cor = 0.279495174662866)	0.99904062373978
**FPPS-HUMAN**	c(cor = -0.129027840030783)	0.99904062373978
**PDCD6-HUMAN**	c(cor = 0.0522934628956228)	0.99904062373978
**STAM1-HUMAN**	c(cor = -0.00923233923781499)	0.99904062373978
**C1TC-HUMAN**	c(cor = -0.0417340456544081)	0.99904062373978
**PSD10-HUMAN**	c(cor = -0.166642437620705)	0.99904062373978
**GRHPR-HUMAN**	c(cor = 0.00104871279307838)	0.99904062373978
**PSMD4-HUMAN**	c(cor = -0.0186103037868055)	0.99904062373978
**MK01-HUMAN**	c(cor = -0.571033136184557)	0.324259162812141
**SYDC-HUMAN**	c(cor = -0.469141313385984)	0.99904062373978
**C4BPB-HUMAN**	c(cor = -0.495400218984325)	0.99904062373978
**MASP1-HUMAN**	c(cor = -0.0570500707529785)	0.99904062373978
**CRKL-HUMAN**	c(cor = -0.254442780625722)	0.99904062373978
**GDIR1-HUMAN**	c(cor = -0.347951271664719)	0.99904062373978
**LV140-HUMAN**	c(cor = -0.267638577041156)	0.99904062373978
**CDC42-HUMAN**	c(cor = 0.134850546323068)	0.99904062373978
**HNRPD-HUMAN**	c(cor = -0.202804742406754)	0.99904062373978
**ATRN-HUMAN**	c(cor = -0.421607723572018)	0.99904062373978
**UBA5-HUMAN**	c(cor = 0.126428253242597)	0.99904062373978
**GAPD1-HUMAN**	c(cor = -0.0462187162303017)	0.99904062373978
**PFKAM-HUMAN**	c(cor = -0.304295808373548)	0.99904062373978
**RLA0-HUMAN;RLA0L-HUMAN**	c(cor = -0.242148514892409)	0.99904062373978
**CMBL-HUMAN**	c(cor = -0.131315500641232)	0.99904062373978
**OTUB1-HUMAN**	c(cor = -0.154385816294714)	0.99904062373978
**NUDC2-HUMAN**	c(cor = -0.110310035519457)	0.99904062373978
**CSN7B-HUMAN**	c(cor = -0.072292436568921)	0.99904062373978
**PROP-HUMAN**	c(cor = -0.149793647606876)	0.99904062373978
**KV105-HUMAN**	c(cor = -0.0936647975952449)	0.99904062373978
**MYL4-HUMAN**	c(cor = -0.252140165876858)	0.99904062373978
**GDIA-HUMAN**	c(cor = -0.351151238642897)	0.99904062373978
**HV349-HUMAN**	c(cor = -0.410450679227864)	0.99904062373978
**ADHX-HUMAN**	c(cor = -0.0476239782405219)	0.99904062373978
**CZIB-HUMAN**	c(cor = -0.141729043136604)	0.99904062373978
**CISD2-HUMAN**	c(cor = -0.136053526722087)	0.99904062373978
**KAP0-HUMAN**	c(cor = -0.136949690513221)	0.99904062373978
**FUMH-HUMAN**	c(cor = -0.307112031809788)	0.99904062373978
**F13B-HUMAN**	c(cor = -0.479589257939032)	0.99904062373978
**AP1B1-HUMAN**	c(cor = 0.0680296098326184)	0.99904062373978
**LBP-HUMAN**	c(cor = -0.169783465380978)	0.99904062373978
**EF1A1-HUMAN;EF1A3-HUMAN**	c(cor = 0.11404195388818)	0.99904062373978
**RAB6A-HUMAN**	c(cor = -0.446796088336602)	0.99904062373978

**FBLN3-HUMAN**	c(cor = -0.292840224990033)	0.99904062373978
**CD14-HUMAN**	c(cor = -0.234577835738831)	0.99904062373978
**VIME-HUMAN**	c(cor = 0.240470126048467)	0.99904062373978
**AGO2-HUMAN**	c(cor = -0.529450273064582)	0.99904062373978
**S10AC-HUMAN**	c(cor = -0.163480892646771)	0.99904062373978
**PLPP-HUMAN**	c(cor = -0.350988188018344)	0.99904062373978
**PSMD7-HUMAN**	c(cor = -0.131852596529833)	0.99904062373978
**IPO7-HUMAN**	c(cor = -0.0539604699383976)	0.99904062373978
**HNRPK-HUMAN**	c(cor = -0.345109041111026)	0.99904062373978
**VATB2-HUMAN**	c(cor = -0.466397161281164)	0.99904062373978
**RFESD-HUMAN**	c(cor = -0.186883364327635)	0.99904062373978
**NIF3L-HUMAN**	c(cor = 0.0819988210431465)	0.99904062373978
**KT3K-HUMAN**	c(cor = -0.0546306596937184)	0.99904062373978
**NEDD8-HUMAN**	c(cor = -0.0489563202697285)	0.99904062373978
**ERMAP-HUMAN**	c(cor = -0.0618919043416171)	0.99904062373978
**TKFC-HUMAN**	c(cor = -0.178767327794766)	0.99904062373978
**DYL2-HUMAN**	c(cor = -0.265241675695938)	0.99904062373978
**ATG3-HUMAN**	c(cor = -0.0361400325505994)	0.99904062373978
**ACYP1-HUMAN**	c(cor = -0.271802260912697)	0.99904062373978
**AMPL-HUMAN**	c(cor = -0.283630342095819)	0.99904062373978
**LV218-HUMAN**	c(cor = 0.180138249020993)	0.99904062373978
**PSD13-HUMAN**	c(cor = 0.0268972369678774)	0.99904062373978
**GP1BB-HUMAN**	c(cor = -0.335064830961595)	0.99904062373978
**HGFA-HUMAN**	c(cor = -0.126462011212632)	0.99904062373978
**AT2B4-HUMAN**	c(cor = -0.12822790680745)	0.99904062373978
**GP1BA-HUMAN**	c(cor = -0.177660918506108)	0.99904062373978
**SMIM1-HUMAN**	c(cor = -0.473377586705732)	0.99904062373978
**THOP1-HUMAN**	c(cor = -0.0305408247944788)	0.99904062373978
**GSTT1-HUMAN**	c(cor = -0.0202866973459412)	0.99904062373978
**ANXA5-HUMAN**	c(cor = -0.0734684979955012)	0.99904062373978
**QSOX1-HUMAN**	c(cor = -0.229944687638912)	0.99904062373978
**ITB3-HUMAN**	c(cor = -0.278375277458276)	0.99904062373978
**CARM1-HUMAN**	c(cor = -0.164694336596187)	0.99904062373978
**LV214-HUMAN**	c(cor = -0.451639615894883)	0.99904062373978
**RAB18-HUMAN**	c(cor = -0.0378356388865249)	0.99904062373978
**CSN3-HUMAN**	c(cor = -0.502043816888245)	0.99904062373978
**CD9-HUMAN**	c(cor = -0.260146379201053)	0.99904062373978
**LV136-HUMAN**	c(cor = 0.159533761699745)	0.99904062373978
**DNJB2-HUMAN**	c(cor = -0.15956123448802)	0.99904062373978
**ECM1-HUMAN**	c(cor = -0.354649902817001)	0.99904062373978
**OSCAR-HUMAN**	c(cor = -0.21116241959352)	0.99904062373978
**PDIA6-HUMAN**	c(cor = -0.182779193150548)	0.99904062373978
**HV146-HUMAN**	c(cor = 0.169426949044417)	0.99904062373978
**PPCS-HUMAN**	c(cor = 0.194604736808879)	0.99904062373978
**ALS-HUMAN**	c(cor = -0.230805392791293)	0.99904062373978
**SHBG-HUMAN**	c(cor = 0.00372824547267688)	0.99904062373978
**PLTP-HUMAN**	c(cor = -0.284604973354285)	0.99904062373978
**SYWC-HUMAN**	c(cor = -0.199136951288353)	0.99904062373978
**GAPR1-HUMAN**	c(cor = -0.162952721653007)	0.99904062373978

**ARPC2-HUMAN**	c(cor = -0.0736976632675768)	0.99904062373978
**KTHY-HUMAN**	c(cor = -0.0634605081562639)	0.99904062373978
**DNJB1-HUMAN**	c(cor = -0.0304148820035215)	0.99904062373978
**CD44-HUMAN**	c(cor = -0.409122018052504)	0.99904062373978
**LV657-HUMAN**	c(cor = -0.103443674279225)	0.99904062373978
**CAMP-HUMAN**	c(cor = 0.0334326273087099)	0.99904062373978
**CDK2-HUMAN**	c(cor = 0.0941270190617255)	0.99904062373978
**AMPN-HUMAN**	c(cor = 0.396649028394583)	0.99904062373978
**HV205-HUMAN**	c(cor = -0.0161487228437355)	0.99904062373978
**CSN6-HUMAN**	c(cor = -0.338683550492488)	0.99904062373978
**GNPI1-HUMAN**	c(cor = 0.171795444909544)	0.99904062373978
**IF2A-HUMAN**	c(cor = -0.19494313104647)	0.99904062373978
**UBR4-HUMAN**	c(cor = 0.0674674019792199)	0.99904062373978
**IPO5-HUMAN**	c(cor = -0.0930904980514972)	0.99904062373978
**H13-HUMAN**	c(cor = -0.104310361268837)	0.99904062373978
**BPI-HUMAN**	c(cor = 0.0448737253259691)	0.99904062373978
**ILEU-HUMAN**	c(cor = -0.406970647184905)	0.99904062373978
**PSMD2-HUMAN**	c(cor = -0.31275299226397)	0.99904062373978
**HV145-HUMAN**	c(cor = -0.0669375120155266)	0.99904062373978
**MET7A-HUMAN**	c(cor = 0.0612854883905372)	0.99904062373978
**AP2M1-HUMAN**	c(cor = -0.246408990656067)	0.99904062373978
**AP2A1-HUMAN**	c(cor = -0.293595074181074)	0.99904062373978
**TYB10-HUMAN**	c(cor = -0.0739873629957261)	0.99904062373978
**LYPA1-HUMAN**	c(cor = 0.200014713887421)	0.99904062373978
**PSD11-HUMAN**	c(cor = 0.00143425705015495)	0.99904062373978
**RUVB1-HUMAN**	c(cor = -0.192047669848914)	0.99904062373978
**GMFG-HUMAN**	c(cor = -0.148470330658164)	0.99904062373978
**IDE-HUMAN**	c(cor = -0.502093398090903)	0.99904062373978
**RS10-HUMAN**	c(cor = 0.61415797437656)	0.0799653730866883
**AK1A1-HUMAN**	c(cor = -0.178874099835554)	0.99904062373978
**RAP1B-HUMAN**	c(cor = -0.341387325686054)	0.99904062373978
**CYTC-HUMAN**	c(cor = -0.223975281306754)	0.99904062373978
**CRLF3-HUMAN**	c(cor = -0.2170560159591)	0.99904062373978
**CRP-HUMAN**	c(cor = -0.113534865246666)	0.99904062373978
**PPIB-HUMAN**	c(cor = -0.351659206300787)	0.99904062373978
**IF4E-HUMAN**	c(cor = -0.014407315670737)	0.99904062373978
**ARP2-HUMAN**	c(cor = -0.232300568945068)	0.99904062373978
**MARE1-HUMAN**	c(cor = -0.262425714130637)	0.99904062373978
**APOC4-HUMAN**	c(cor = -0.364753110907303)	0.99904062373978
**APOF-HUMAN**	c(cor = -0.237311917452957)	0.99904062373978
**KV116-HUMAN**	c(cor = -0.421445549585872)	0.99904062373978
**PYRG1-HUMAN**	c(cor = -0.104197530503352)	0.99904062373978
**EIF3J-HUMAN**	c(cor = 0.166224707152903)	0.99904062373978
**EF2-HUMAN**	c(cor = -0.196250812812097)	0.99904062373978
**ZYX-HUMAN**	c(cor = -0.242679188683135)	0.99904062373978
**CSN8-HUMAN**	c(cor = -0.116161152611783)	0.99904062373978
**HBAZ-HUMAN**	c(cor = 0.126994183915562)	0.99904062373978
**AP2B1-HUMAN**	c(cor = -0.0759091197243033)	0.99904062373978
**RTN3-HUMAN**	c(cor = -0.586031091805659)	0.204179530916438

**PDE12-HUMAN**	c(cor = -0.286431448369015)	0.99904062373978
**LV469-HUMAN**	c(cor = 0.109282798676973)	0.99904062373978
**NUDT5-HUMAN**	c(cor = -0.196038657523286)	0.99904062373978
**1433F-HUMAN**	c(cor = -0.206747234801126)	0.99904062373978
**PSD12-HUMAN**	c(cor = 0.0627463607033483)	0.99904062373978
**GMPR2-HUMAN**	c(cor = -0.311276565245723)	0.99904062373978
**XPO1-HUMAN**	c(cor = -0.302007828690854)	0.99904062373978
**PSMG3-HUMAN**	c(cor = 0.0104823100972063)	0.99904062373978
**PZP-HUMAN**	c(cor = -0.138375148141019)	0.99904062373978
**LV321-HUMAN**	c(cor = -0.437121747254317)	0.99904062373978
**DCTN2-HUMAN**	c(cor = -0.164467922657368)	0.99904062373978
**ANFY1-HUMAN**	c(cor = -0.333246813491704)	0.99904062373978
**BID-HUMAN**	c(cor = -0.44926628638293)	0.99904062373978
**PDIA3-HUMAN**	c(cor = -0.0924270209788488)	0.99904062373978
**TRML1-HUMAN**	c(cor = 0.00619657267988326)	0.99904062373978
**ENPL-HUMAN**	c(cor = 0.0530390706471619)	0.99904062373978
**BGH3-HUMAN**	c(cor = -0.270300599210592)	0.99904062373978
**CNDP2-HUMAN**	c(cor = -0.010258131545234)	0.99904062373978
**PSA-HUMAN**	c(cor = -0.089576239814887)	0.99904062373978
**CLCA-HUMAN**	c(cor = -0.271849150822892)	0.99904062373978
**ADIPO-HUMAN**	c(cor = -0.162969958577435)	0.99904062373978
**LV223-HUMAN**	c(cor = 0.156694927476086)	0.99904062373978
**P5CR3-HUMAN**	c(cor = -0.0432187950432143)	0.99904062373978
**PIPNA-HUMAN**	c(cor = -0.284111899583591)	0.99904062373978
**DBNL-HUMAN**	c(cor = -0.352388828886507)	0.99904062373978
**RTCA-HUMAN**	c(cor = -0.131977695862604)	0.99904062373978
**CFAD-HUMAN**	c(cor = -0.264686664078454)	0.99904062373978
**UBXN1-HUMAN**	c(cor = -0.211195446399388)	0.99904062373978
**DNJC9-HUMAN**	c(cor = 0.0155927621603454)	0.99904062373978
**PP1G-HUMAN;PP1A-HUMAN;PP1B-HUMAN**	c(cor = 0.11705442362648)	0.99904062373978
**MNDA-HUMAN**	c(cor = -0.127267369396074)	0.99904062373978
**HSPB1-HUMAN**	c(cor = -0.175713635275618)	0.99904062373978
**CUL1-HUMAN**	c(cor = -0.471773600046115)	0.99904062373978
**AK1C3-HUMAN**	c(cor = -0.14156833817907)	0.99904062373978
**CHLE-HUMAN**	c(cor = -0.146739502834318)	0.99904062373978
**GRAN-HUMAN**	c(cor = 0.133786584923926)	0.99904062373978
**GMPPB-HUMAN**	c(cor = -0.408991732522538)	0.99904062373978
**VPS4B-HUMAN;FIGL1-HUMAN;VPS4A-HUMAN**	c(cor = -0.242895847296832)	0.99904062373978
**RHG01-HUMAN**	c(cor = 0.0209824275860511)	0.99904062373978
**KELL-HUMAN**	c(cor = -0.129338071854856)	0.99904062373978
**ACES-HUMAN**	c(cor = -0.323291514718855)	0.99904062373978
**PHP14-HUMAN**	c(cor = -0.0602229956914522)	0.99904062373978
**CAZA2-HUMAN**	c(cor = 0.234578841572922)	0.99904062373978
**CAVN2-HUMAN**	c(cor = -0.361420642009034)	0.99904062373978
**ORN-HUMAN**	c(cor = -0.359542768099462)	0.99904062373978
**CAP7-HUMAN**	c(cor = -0.236728095583892)	0.99904062373978
**IAH1-HUMAN**	c(cor = -0.136062913129932)	0.99904062373978
**UT1-HUMAN**	c(cor = -0.216930758151104)	0.99904062373978
**KGUA-HUMAN**	c(cor = -0.0958618351797572)	0.99904062373978

**ICAM2-HUMAN**	c(cor = -0.0858070154012816)	0.99904062373978
**PHS2-HUMAN**	c(cor = -0.182530601769144)	0.99904062373978
**PRG3-HUMAN**	c(cor = 0.0672073325262783)	0.99904062373978
**CPIN1-HUMAN**	c(cor = -0.199899727767361)	0.99904062373978
**SGTA-HUMAN**	c(cor = 0.0471444275197282)	0.99904062373978
**SGT1-HUMAN**	c(cor = -0.182379008557277)	0.99904062373978
**ARPC3-HUMAN**	c(cor = -0.235595659338825)	0.99904062373978
**ATPB-HUMAN**	c(cor = 0.0511436749213904)	0.99904062373978
**LRC20-HUMAN**	c(cor = 0.0793419477525326)	0.99904062373978
**IF4A2-HUMAN**	c(cor = -0.123186666463726)	0.99904062373978
**STX7-HUMAN**	c(cor = -0.147429467801969)	0.99904062373978
**DSG2-HUMAN**	c(cor = -0.0802320506036843)	0.99904062373978
**RAGP1-HUMAN**	c(cor = -0.200515562845445)	0.99904062373978
**IBP6-HUMAN**	c(cor = -0.073098418155487)	0.99904062373978
**IPSP-HUMAN**	c(cor = -0.407148246691331)	0.99904062373978
**KV621-HUMAN**	c(cor = -0.203157579676695)	0.99904062373978
**CDC37-HUMAN**	c(cor = -0.11419976022858)	0.99904062373978
**PRS6B-HUMAN**	c(cor = -0.243957083515254)	0.99904062373978
**BLMH-HUMAN**	c(cor = -0.398266772262945)	0.99904062373978
**PLIN3-HUMAN**	c(cor = -0.168158819886358)	0.99904062373978
**ARPC4-HUMAN**	c(cor = -0.1706710154186)	0.99904062373978
**NIT1-HUMAN**	c(cor = -0.20484887450726)	0.99904062373978
**FA11-HUMAN**	c(cor = -0.375327416600594)	0.99904062373978
**NQO2-HUMAN**	c(cor = -0.0255659897223495)	0.99904062373978
**PIN1-HUMAN**	c(cor = -0.207257539138737)	0.99904062373978
**ENDD1-HUMAN**	c(cor = 0.314811660941595)	0.99904062373978
**XPO2-HUMAN**	c(cor = 0.0382221357709919)	0.99904062373978
**VATG1-HUMAN**	c(cor = -0.583542367484909)	0.220753210401455
**GMDS-HUMAN**	c(cor = -0.461657366140213)	0.99904062373978
**RBX1-HUMAN**	c(cor = -0.222381804933483)	0.99904062373978
**TSN-HUMAN**	c(cor = -0.359844927962908)	0.99904062373978
**KAPCA-HUMAN**	c(cor = -0.215849717775158)	0.99904062373978
**PSMD3-HUMAN**	c(cor = -0.144112587685323)	0.99904062373978
**MTPN-HUMAN**	c(cor = -0.118747388678162)	0.99904062373978
**PNPO-HUMAN**	c(cor = -0.0317738342510607)	0.99904062373978
**CRAC1-HUMAN**	c(cor = 0.195792368675792)	0.99904062373978
**TMX1-HUMAN**	c(cor = 0.314126610191496)	0.99904062373978
**IMA3-HUMAN**	c(cor = -0.255897193594492)	0.99904062373978
**CSN2-HUMAN**	c(cor = -0.376516155917526)	0.99904062373978
**RAC2-HUMAN**	c(cor = -0.273727080440899)	0.99904062373978
**DHSO-HUMAN**	c(cor = -0.160091399926557)	0.99904062373978
**STMN1-HUMAN**	c(cor = -0.137128217222263)	0.99904062373978
**MMP9-HUMAN**	c(cor = -0.122957232719846)	0.99904062373978
**DYL1-HUMAN**	c(cor = -0.235453315209716)	0.99904062373978
**FHR5-HUMAN**	c(cor = -0.432083567707482)	0.99904062373978
**PTN11-HUMAN**	c(cor = -0.0263463963421301)	0.99904062373978
**LHPP-HUMAN**	c(cor = 0.112641566475999)	0.99904062373978
**CETP-HUMAN**	c(cor = 0.0978742963889491)	0.99904062373978
**ULA1-HUMAN**	c(cor = -0.27890397244804)	0.99904062373978

**SYYC-HUMAN**	c(cor = -0.00300317466453643)	0.99904062373978
**THG1-HUMAN**	c(cor = -0.0936351352465562)	0.99904062373978
**RGS10-HUMAN**	c(cor = -0.297213717980054)	0.99904062373978
**S14L2-HUMAN**	c(cor = 0.0328740235110598)	0.99904062373978
**PROC-HUMAN**	c(cor = -0.002100942856115)	0.99904062373978
**RBBP9-HUMAN**	c(cor = -0.385702164881956)	0.99904062373978
**PDIA1-HUMAN**	c(cor = -0.189247217727315)	0.99904062373978
**VASN-HUMAN**	c(cor = 0.0457044015181284)	0.99904062373978
**TBCEL-HUMAN**	c(cor = -0.0818753452601729)	0.99904062373978
**BASI-HUMAN**	c(cor = -0.0301369008191886)	0.99904062373978
**IF5-HUMAN**	c(cor = -0.0911990245148422)	0.99904062373978
**BPNT1-HUMAN**	c(cor = -0.320131787408205)	0.99904062373978
**GMFB-HUMAN**	c(cor = 0.0893636064023773)	0.99904062373978
**ATPA-HUMAN**	c(cor = -0.349802020165379)	0.99904062373978
**KC1A-HUMAN**	c(cor = 0.144142649443762)	0.99904062373978
**DHR11-HUMAN**	c(cor = -0.259193228873166)	0.99904062373978
**IF1AX-HUMAN**	c(cor = -0.321787545924475)	0.99904062373978
**HV315-HUMAN**	c(cor = -0.272783311390955)	0.99904062373978
**MOT1-HUMAN**	c(cor = -0.139098245743348)	0.99904062373978
**ALDR-HUMAN**	c(cor = -0.151907190784969)	0.99904062373978
**RILP-HUMAN**	c(cor = 0.125527412256603)	0.99904062373978
**B2MG-HUMAN**	c(cor = -0.226990421246944)	0.99904062373978
**MVD1-HUMAN**	c(cor = -0.47175572643403)	0.99904062373978
**OSBP2-HUMAN**	c(cor = -0.233105511239858)	0.99904062373978
**DHX9-HUMAN**	c(cor = 0.330759997709047)	0.99904062373978
**LFA3-HUMAN**	c(cor = 0.0818385795416702)	0.99904062373978
**IBP2-HUMAN**	c(cor = -0.206724600040145)	0.99904062373978
**NCHL1-HUMAN**	c(cor = -0.431855443501488)	0.99904062373978
**RAB1A-HUMAN**	c(cor = -0.064187150644549)	0.99904062373978
**BACH-HUMAN**	c(cor = -0.173052473352508)	0.99904062373978
**KPRA-HUMAN**	c(cor = -0.287217733083362)	0.99904062373978
**VATE1-HUMAN**	c(cor = -0.157484711905763)	0.99904062373978
**IBP3-HUMAN**	c(cor = -0.252661116508515)	0.99904062373978
**MTNA-HUMAN**	c(cor = -0.175210747585078)	0.99904062373978
**FKBP4-HUMAN**	c(cor = 0.0144116113560853)	0.99904062373978
**MP2K1-HUMAN**	c(cor = -0.410331641694332)	0.99904062373978
**PPM1A-HUMAN**	c(cor = 0.0698250183299178)	0.99904062373978
**LANC2-HUMAN**	c(cor = -0.472145787323169)	0.99904062373978
**S100P-HUMAN**	c(cor = -0.00397363263255792)	0.99904062373978
**TIMP1-HUMAN**	c(cor = -0.250462144474707)	0.99904062373978
**RAB2A-HUMAN**	c(cor = -0.288739310428228)	0.99904062373978
**2A5D-HUMAN**	c(cor = -0.0255546900943185)	0.99904062373978
**ICAM4-HUMAN**	c(cor = 0.0541623663730435)	0.99904062373978
**PDLI1-HUMAN**	c(cor = -0.299820146718517)	0.99904062373978
**CRK-HUMAN**	c(cor = -0.0780607186332038)	0.99904062373978
**LSM3-HUMAN**	c(cor = -0.129540931855074)	0.99904062373978
**TCTP-HUMAN**	c(cor = -0.160866241101958)	0.99904062373978
**PP6R1-HUMAN**	c(cor = -0.520092689156856)	0.99904062373978
**DNPEP-HUMAN**	c(cor = -0.514660012467533)	0.99904062373978

**F1142-HUMAN**	c(cor = 0.0127035819208408)	0.99904062373978
**ACTN4-HUMAN**	c(cor = -0.137472550601083)	0.99904062373978
**RTN4-HUMAN**	c(cor = 0.0447266026303565)	0.99904062373978
**RSSA-HUMAN**	c(cor = 0.136465617137824)	0.99904062373978
**BIN2-HUMAN**	c(cor = -0.146023338289796)	0.99904062373978
**4EBP1-HUMAN**	c(cor = 0.0364767878961805)	0.99904062373978
**BTD-HUMAN**	c(cor = -0.33839487885957)	0.99904062373978
**PFD5-HUMAN**	c(cor = 0.163733347242194)	0.99904062373978
**PIPNB-HUMAN**	c(cor = -0.220576585696654)	0.99904062373978
**SPP24-HUMAN**	c(cor = -0.0921717601656354)	0.99904062373978
**SAA1-HUMAN**	c(cor = -0.0090284551283231)	0.99904062373978
**METK2-HUMAN**	c(cor = -0.113084010421779)	0.99904062373978
**ARP3-HUMAN**	c(cor = -0.0795261257149486)	0.99904062373978
**COTL1-HUMAN**	c(cor = -0.040043242303039)	0.99904062373978
**WNK1-HUMAN**	c(cor = -0.352441874538682)	0.99904062373978
**PCOC1-HUMAN**	c(cor = 0.0272865597303456)	0.99904062373978
**NR2CA-HUMAN**	c(cor = 0.255260610328402)	0.99904062373978
**GPIX-HUMAN**	c(cor = -0.19536227815538)	0.99904062373978
**FKBP3-HUMAN**	c(cor = -0.0860621900536646)	0.99904062373978
**RAB6B-HUMAN**	c(cor = -0.209077860977686)	0.99904062373978
**ENOPH-HUMAN**	c(cor = -0.262845539481194)	0.99904062373978
**ZPI-HUMAN**	c(cor = -0.367929988845848)	0.99904062373978
**UBC12-HUMAN**	c(cor = -0.141018984122664)	0.99904062373978
**AQP1-HUMAN**	c(cor = -0.0610540374614495)	0.99904062373978
**QSPP-HUMAN**	c(cor = 0.0266595710100712)	0.99904062373978
**HV226-HUMAN**	c(cor = -0.322509451952662)	0.99904062373978
**AT2B1-HUMAN**	c(cor = -0.193036294763031)	0.99904062373978
**HXK1-HUMAN**	c(cor = -0.332878344944486)	0.99904062373978
**CUL2-HUMAN**	c(cor = -0.342020205982264)	0.99904062373978
**DAF-HUMAN**	c(cor = -0.29836163089173)	0.99904062373978
**DEOC-HUMAN**	c(cor = -0.24479572646814)	0.99904062373978
**PTBP1-HUMAN**	c(cor = -0.0185860907795497)	0.99904062373978
**LCAT-HUMAN**	c(cor = -0.197153924919937)	0.99904062373978
**PLP2-HUMAN**	c(cor = 0.355163924901178)	0.99904062373978
**DIAP1-HUMAN**	c(cor = -0.0115897543257508)	0.99904062373978
**VAC14-HUMAN**	c(cor = 0.0953407746177485)	0.99904062373978
**SHLB2-HUMAN**	c(cor = -0.100996957857493)	0.99904062373978
**AT1A1-HUMAN**	c(cor = -0.31905982961775)	0.99904062373978
**RAB7A-HUMAN**	c(cor = -0.483641318194721)	0.99904062373978
**MDHM-HUMAN**	c(cor = 0.222645084108652)	0.99904062373978
**DNJA4-HUMAN**	c(cor = -0.0937148257968996)	0.99904062373978
**RAB21-HUMAN**	c(cor = 0.271790758296157)	0.99904062373978
**SC22B-HUMAN**	c(cor = -0.439655465321095)	0.99904062373978
**ENOF1-HUMAN**	c(cor = -0.12247953517923)	0.99904062373978
**XPP1-HUMAN**	c(cor = 0.250637194262539)	0.99904062373978
**VASP-HUMAN**	c(cor = -0.305948325603503)	0.99904062373978
**CNDP1-HUMAN**	c(cor = -0.123569680299129)	0.99904062373978
**ERF1-HUMAN**	c(cor = -0.250113956398749)	0.99904062373978
**ARLY-HUMAN**	c(cor = -0.0992094935604971)	0.99904062373978

**COR1C-HUMAN**	c(cor = -0.0451634527041809)	0.99904062373978
**RAB5C-HUMAN**	c(cor = 0.0358742883616808)	0.99904062373978
**TRXR1-HUMAN**	c(cor = 0.294323870280321)	0.99904062373978
**IF1AY-HUMAN**	c(cor = -0.164507422843967)	0.99904062373978
**FCGBP-HUMAN**	c(cor = -0.116396093959031)	0.99904062373978
**NUDC1-HUMAN**	c(cor = 0.182648021190459)	0.99904062373978
**AMPD3-HUMAN**	c(cor = -0.141352313610592)	0.99904062373978
**RIC8A-HUMAN**	c(cor = 0.102953116023738)	0.99904062373978
**AN32A-HUMAN**	c(cor = 0.210052100169089)	0.99904062373978
**GNL1-HUMAN**	c(cor = -0.229225481175972)	0.99904062373978
**ARC1B-HUMAN**	c(cor = -0.0557677446200148)	0.99904062373978
**CHM4B-HUMAN**	c(cor = -0.0769991639896906)	0.99904062373978
**AP4A-HUMAN**	c(cor = 0.0124697677238576)	0.99904062373978
**KRT82-HUMAN**	c(cor = -0.425142506359337)	0.99904062373978
**GANAB-HUMAN**	c(cor = 0.199831472842336)	0.99904062373978
**HEBP2-HUMAN**	c(cor = -0.27093856330475)	0.99904062373978
**HNRPF-HUMAN**	c(cor = -0.172273405469828)	0.99904062373978
**AAMDC-HUMAN**	c(cor = 0.0220320020136003)	0.99904062373978
**UBP7-HUMAN**	c(cor = -0.0385600335529468)	0.99904062373978
**LG3BP-HUMAN**	c(cor = -0.350506380371619)	0.99904062373978
**GPV-HUMAN**	c(cor = -0.180218915179994)	0.99904062373978
**CYRIB-HUMAN**	c(cor = -0.120260747296452)	0.99904062373978
**GMPPA-HUMAN**	c(cor = -0.205528720305889)	0.99904062373978
**FCN2-HUMAN**	c(cor = -0.0152496080372353)	0.99904062373978
**USP9X-HUMAN**	c(cor = -0.418501393891335)	0.99904062373978
**CN37-HUMAN**	c(cor = 0.0722396368612661)	0.99904062373978
**PYGL-HUMAN**	c(cor = -0.212984675929031)	0.99904062373978
**ILK-HUMAN**	c(cor = -0.200345103233258)	0.99904062373978
**PSMG1-HUMAN**	c(cor = 0.0603617133651021)	0.99904062373978
**SRC8-HUMAN**	c(cor = -0.143067587005615)	0.99904062373978
**HDHD2-HUMAN**	c(cor = -0.040425735004661)	0.99904062373978
**CHM4A-HUMAN**	c(cor = -0.114712891579891)	0.99904062373978
**ABCB6-HUMAN**	c(cor = 0.0245105205963869)	0.99904062373978
**TBC24-HUMAN**	c(cor = -0.215452803741722)	0.99904062373978
**PARVB-HUMAN**	c(cor = 0.117123736929957)	0.99904062373978
**CPPED-HUMAN**	c(cor = -0.331782609611409)	0.99904062373978
**TMED2-HUMAN**	c(cor = -0.0186497908931921)	0.99904062373978
**FAS-HUMAN**	c(cor = -0.148158562368994)	0.99904062373978
**BIP-HUMAN**	c(cor = -0.261165193525458)	0.99904062373978
**ELOB-HUMAN**	c(cor = -0.420394223620752)	0.99904062373978
**YKT6-HUMAN**	c(cor = 0.0637401223136498)	0.99904062373978
**LV460-HUMAN**	c(cor = 0.163406423759157)	0.99904062373978
**ITB2-HUMAN**	c(cor = -0.15652620713893)	0.99904062373978
**RADI-HUMAN**	c(cor = 0.0983027332827556)	0.99904062373978
**HNRPQ-HUMAN**	c(cor = -0.0965264736128903)	0.99904062373978
**COASY-HUMAN**	c(cor = -0.339016955343516)	0.99904062373978
**PI16-HUMAN**	c(cor = -0.0424625286034255)	0.99904062373978
**S10AB-HUMAN**	c(cor = -0.284010008160065)	0.99904062373978
**UCHL5-HUMAN**	c(cor = -0.415813801049047)	0.99904062373978

**LZIC-HUMAN**	c(cor = 0.276475112965211)	0.99904062373978
**HGS-HUMAN**	c(cor = -0.212490214949246)	0.99904062373978
**RS12-HUMAN**	c(cor = -0.28511779171262)	0.99904062373978
**TFR1-HUMAN**	c(cor = -0.465979076347377)	0.99904062373978
**IF2B-HUMAN**	c(cor = 0.246730693760669)	0.99904062373978
**ECP-HUMAN**	c(cor = 0.084306907877349)	0.99904062373978
**PUR2-HUMAN**	c(cor = -0.0241573184126706)	0.99904062373978
**MASP2-HUMAN**	c(cor = -0.23145277685864)	0.99904062373978
**LYAM1-HUMAN**	c(cor = -0.182250744113665)	0.99904062373978
**MOB1B-HUMAN;MOB1A-HUMAN**	c(cor = -0.101686220792885)	0.99904062373978
**CLIC2-HUMAN**	c(cor = 0.23638557151329)	0.99904062373978
**SIAS-HUMAN**	c(cor = 0.12194931395781)	0.99904062373978
**PRG4-HUMAN**	c(cor = -0.251862296408487)	0.99904062373978
**ADK-HUMAN**	c(cor = -0.0419048440451103)	0.99904062373978
**TNPO1-HUMAN**	c(cor = 0.0258600069677993)	0.99904062373978
**ADPRS-HUMAN**	c(cor = -0.23775899449562)	0.99904062373978
**CADH5-HUMAN**	c(cor = -0.411568134994034)	0.99904062373978
**COMP-HUMAN**	c(cor = -0.305323332306927)	0.99904062373978
**KVD29-HUMAN;KV229-HUMAN**	c(cor = 0.179950758311563)	0.99904062373978
**ACOC-HUMAN**	c(cor = -0.0610511278608139)	0.99904062373978
**CRIS3-HUMAN**	c(cor = -0.323890653508693)	0.99904062373978
**LEG10-HUMAN**	c(cor = -0.112193541361)	0.99904062373978
**GPX4-HUMAN**	c(cor = 0.00861458817134458)	0.99904062373978
**UBP47-HUMAN**	c(cor = -0.359602323985925)	0.99904062373978
**DCTN1-HUMAN**	c(cor = 0.199917834291891)	0.99904062373978
**CPNE1-HUMAN**	c(cor = 0.0715664269798992)	0.99904062373978
**PCYOX-HUMAN**	c(cor = -0.146582901007112)	0.99904062373978
**IBP5-HUMAN**	c(cor = 0.185493864984665)	0.99904062373978
**RAB14-HUMAN**	c(cor = -0.157992407106697)	0.99904062373978
**JAM1-HUMAN**	c(cor = -0.115402401914259)	0.99904062373978
**TBCB-HUMAN**	c(cor = -0.275193987969293)	0.99904062373978
**ERP44-HUMAN**	c(cor = -0.290568279954593)	0.99904062373978
**SRGN-HUMAN**	c(cor = -0.197570856422072)	0.99904062373978
**CALD1-HUMAN**	c(cor = -0.162689729929205)	0.99904062373978
**PLS1-HUMAN**	c(cor = -0.482677648326528)	0.99904062373978
**KS6A3-HUMAN**	c(cor = -0.0881635795869825)	0.99904062373978
**EZRI-HUMAN**	c(cor = 0.0253519683070457)	0.99904062373978
**LV746-HUMAN**	c(cor = -0.247137550710093)	0.99904062373978
**KCMF1-HUMAN**	c(cor = -0.200573190555303)	0.99904062373978
**QORX-HUMAN**	c(cor = -0.150397983583344)	0.99904062373978
**PTRD1-HUMAN**	c(cor = -0.309437438336456)	0.99904062373978
**AT2A3-HUMAN**	c(cor = -0.300209849452113)	0.99904062373978
**LAT4-HUMAN**	c(cor = -0.116048208334051)	0.99904062373978
**SYCC-HUMAN**	c(cor = -0.282174033122168)	0.99904062373978
**LV316-HUMAN**	c(cor = -0.258323105318076)	0.99904062373978
**GTR3-HUMAN;GTR14-HUMAN**	c(cor = -0.0899153432357494)	0.99904062373978
**KVD21-HUMAN**	c(cor = 0.0981567060691078)	0.99904062373978
**ELOC-HUMAN**	c(cor = -0.395585163792658)	0.99904062373978
**PEF1-HUMAN**	c(cor = -0.0347849378120183)	0.99904062373978

**RNAS1-HUMAN**	c(cor = -0.34222530623255)	0.99904062373978
**ATG4A-HUMAN**	c(cor = 0.0160760298616153)	0.99904062373978
**LYVE1-HUMAN**	c(cor = -0.195594716246034)	0.99904062373978
**MPCP-HUMAN**	c(cor = -0.127942202218832)	0.99904062373978
**IGE-HUMAN**	c(cor = -0.0802532448905675)	0.99904062373978
**ASAP1-HUMAN**	c(cor = 0.000519616974193046)	0.99904062373978
**GNAI2-HUMAN**	c(cor = -0.18404259182113)	0.99904062373978
**SCFD1-HUMAN**	c(cor = 0.0813336116334611)	0.99904062373978
**CLIC4-HUMAN**	c(cor = -0.00443969776727321)	0.99904062373978
**TSNAX-HUMAN**	c(cor = -0.196880457587184)	0.99904062373978
**GGEE3-HUMAN;PAGE2-HUMAN**	c(cor = 0.232472769163206)	0.99904062373978
**GLGB-HUMAN**	c(cor = -0.291055234480255)	0.99904062373978
**DENR-HUMAN**	c(cor = 0.327216474764447)	0.99904062373978
**PGRP1-HUMAN**	c(cor = -0.145781280027657)	0.99904062373978
**SELO-HUMAN**	c(cor = -0.163947295415641)	0.99904062373978
**CD99-HUMAN**	c(cor = -0.130641578505008)	0.99904062373978
**VAMP2-HUMAN;VAMP3-HUMAN**	c(cor = -0.0938258808622186)	0.99904062373978
**PSMD8-HUMAN**	c(cor = 0.00960246789544205)	0.99904062373978
**CH10-HUMAN**	c(cor = -0.305011391699473)	0.99904062373978
**WIPI1-HUMAN**	c(cor = -0.36365525722282)	0.99904062373978
**ANXA4-HUMAN**	c(cor = 0.0377555821371823)	0.99904062373978
**HMGN2-HUMAN**	c(cor = -0.0668478490979389)	0.99904062373978
**AMPB-HUMAN**	c(cor = -0.277744329329266)	0.99904062373978
**LTBP1-HUMAN**	c(cor = -0.395861427058091)	0.99904062373978
**EF1G-HUMAN**	c(cor = -0.152660628158788)	0.99904062373978
**GLU2B-HUMAN**	c(cor = -0.358183932342269)	0.99904062373978
**HMGB2-HUMAN**	c(cor = -0.0740921210219794)	0.99904062373978
**EF1D-HUMAN**	c(cor = -0.262080332906342)	0.99904062373978
**CALR-HUMAN**	c(cor = -0.252393430653059)	0.99904062373978
**CATD-HUMAN**	c(cor = -0.28245859621647)	0.99904062373978
**MMRN1-HUMAN**	c(cor = -0.303957018693508)	0.99904062373978
**CSRP1-HUMAN**	c(cor = -0.421492807770962)	0.99904062373978
**ERF3A-HUMAN**	c(cor = -0.187247104946938)	0.99904062373978
**PEBP4-HUMAN**	c(cor = -0.220259330522154)	0.99904062373978
**GNAI3-HUMAN**	c(cor = -0.254811737242055)	0.99904062373978
**UBA3-HUMAN**	c(cor = -0.128487784399993)	0.99904062373978
**TBG1-HUMAN;TBG2-HUMAN**	c(cor = -0.36729373078086)	0.99904062373978
**VTA1-HUMAN**	c(cor = -0.183749305507748)	0.99904062373978
**K1H1-HUMAN**	c(cor = -0.0219446279348677)	0.99904062373978
**RS17-HUMAN**	c(cor = 0.184335166764568)	0.99904062373978
**BCAM-HUMAN**	c(cor = -0.0893182967862054)	0.99904062373978
**YBOX3-HUMAN**	c(cor = 0.165782100714467)	0.99904062373978
**ISG15-HUMAN**	c(cor = -0.400581853568038)	0.99904062373978
**LV548-HUMAN**	c(cor = -0.162208991501577)	0.99904062373978
**NNRE-HUMAN**	c(cor = 0.0602570549128655)	0.99904062373978
**DPYL2-HUMAN**	c(cor = -0.496403968050215)	0.99904062373978
**BASP1-HUMAN**	c(cor = -0.179540383323109)	0.99904062373978
**XRP2-HUMAN**	c(cor = -0.0187236994247065)	0.99904062373978
**DOPO-HUMAN**	c(cor = -0.129722134915934)	0.99904062373978

**MBL2-HUMAN**	c(cor = -0.206846811697857)	0.99904062373978
**PURA-HUMAN**	c(cor = 0.0755511719370262)	0.99904062373978
**RHAG-HUMAN**	c(cor = 0.126871815166079)	0.99904062373978
**PSMG2-HUMAN**	c(cor = -0.419927259041576)	0.99904062373978
**VP26B-HUMAN**	c(cor = -0.142983706811634)	0.99904062373978
**SAA2-HUMAN**	c(cor = 0.00241272140576969)	0.99904062373978
**DNJA2-HUMAN**	c(cor = -0.376344219726504)	0.99904062373978
**GLPA-HUMAN**	c(cor = -0.0257805535699884)	0.99904062373978
**PIGR-HUMAN**	c(cor = -0.0820186935031516)	0.99904062373978
**LMAN2-HUMAN**	c(cor = -0.160953144606888)	0.99904062373978
**HCLS1-HUMAN**	c(cor = -0.267367122997828)	0.99904062373978
**ANGI-HUMAN**	c(cor = -0.14024670634721)	0.99904062373978
**SPRC-HUMAN**	c(cor = -0.412013116204169)	0.99904062373978
**RAB2B-HUMAN**	c(cor = -0.332595435310612)	0.99904062373978
**MP2K3-HUMAN**	c(cor = -0.138498478641819)	0.99904062373978
**MP2K2-HUMAN**	c(cor = -0.23814480071795)	0.99904062373978
**LA-HUMAN**	c(cor = -0.305813758456647)	0.99904062373978
**CSN7A-HUMAN**	c(cor = -0.244581193624743)	0.99904062373978
**NRBP-HUMAN**	c(cor = 0.0620267247340258)	0.99904062373978
**HLAA-HUMAN**	c(cor = -0.15585582914222)	0.99904062373978
**PDCD5-HUMAN**	c(cor = 0.169202395259065)	0.99904062373978
**CY24B-HUMAN**	c(cor = -0.215495287092099)	0.99904062373978
**CD36-HUMAN**	c(cor = -0.144881841117867)	0.99904062373978
**SERC-HUMAN**	c(cor = 0.287354754156802)	0.99904062373978
**AHNK-HUMAN**	c(cor = -0.211639451056284)	0.99904062373978
**LAMP2-HUMAN**	c(cor = -0.30447151911646)	0.99904062373978
**IDHP-HUMAN**	c(cor = -0.358408669278353)	0.99904062373978
**FCN1-HUMAN**	c(cor = -0.0903140643664067)	0.99904062373978
**PDIA4-HUMAN**	c(cor = -0.306230612230064)	0.99904062373978
**FHL1-HUMAN**	c(cor = -0.149532607325298)	0.99904062373978
**CALX-HUMAN**	c(cor = -0.285372206044022)	0.99904062373978
**NEUG-HUMAN**	c(cor = -0.136019712065953)	0.99904062373978
**ITAM-HUMAN**	c(cor = 0.175034205303667)	0.99904062373978
**VNN1-HUMAN**	c(cor = -0.019625481022752)	0.99904062373978
**THAS-HUMAN**	c(cor = 0.087507194751175)	0.99904062373978
**ROA1-HUMAN**	c(cor = -0.321607256796336)	0.99904062373978
**HUWE1-HUMAN**	c(cor = -0.151786532763859)	0.99904062373978
**PGTB2-HUMAN**	c(cor = -0.291898119748009)	0.99904062373978
**YBOX1-HUMAN**	c(cor = 0.106114267923658)	0.99904062373978
**PIEZ1-HUMAN**	c(cor = -0.0578913863056333)	0.99904062373978
**SEM7A-HUMAN**	c(cor = -0.13883441405894)	0.99904062373978
**RIR1-HUMAN**	c(cor = -0.146675049811889)	0.99904062373978
**SAC1-HUMAN**	c(cor = -0.334246461338007)	0.99904062373978
**LVX54-HUMAN**	c(cor = -0.0293570521471055)	0.99904062373978
**PPP5-HUMAN**	c(cor = -0.0816953313387025)	0.99904062373978
**ADRM1-HUMAN**	c(cor = 0.259075910711302)	0.99904062373978
**PLPL6-HUMAN**	c(cor = 0.0260410943512949)	0.99904062373978
**SNP23-HUMAN**	c(cor = -0.117384091779461)	0.99904062373978
**CH60-HUMAN**	c(cor = -0.00820122430071313)	0.99904062373978

**IGBP1-HUMAN**	c(cor = 0.111035313270508)	0.99904062373978
**DCA11-HUMAN**	c(cor = -0.297986491860647)	0.99904062373978
**GGH-HUMAN**	c(cor = -0.109123249587567)	0.99904062373978
**HV320-HUMAN**	c(cor = -0.215166137980006)	0.99904062373978
**PCNP-HUMAN**	c(cor = 0.168228583254485)	0.99904062373978
**H2AZ-HUMAN;H2AV-HUMAN**	c(cor = -0.0587972603875595)	0.99904062373978
**TXND5-HUMAN**	c(cor = -0.167485956798239)	0.99904062373978
**AL5AP-HUMAN**	c(cor = -0.231694179744765)	0.99904062373978
**GNAS2-HUMAN;GNAS1-HUMAN**	c(cor = -0.0165359475014286)	0.99904062373978
**COIA1-HUMAN**	c(cor = -0.340887067320104)	0.99904062373978
**ROA2-HUMAN**	c(cor = -0.031405993494187)	0.99904062373978
**TTC38-HUMAN**	c(cor = -0.0217211348462091)	0.99904062373978
**COL11-HUMAN**	c(cor = -0.344822148175097)	0.99904062373978
**ACSL3-HUMAN**	c(cor = -0.399085124611188)	0.99904062373978
**PROZ-HUMAN**	c(cor = -0.123374307591958)	0.99904062373978
**CAN5-HUMAN**	c(cor = 0.0165449601650309)	0.99904062373978
**PPP6-HUMAN**	c(cor = -0.486194665814468)	0.99904062373978
**LV545-HUMAN;LV539-HUMAN**	c(cor = -0.207787779078091)	0.99904062373978
**S29A1-HUMAN**	c(cor = -0.165998244406645)	0.99904062373978
**KT33A-HUMAN**	c(cor = 0.0320493805797334)	0.99904062373978
**LAMP1-HUMAN**	c(cor = -0.268344710609193)	0.99904062373978
**VAPA-HUMAN**	c(cor = -0.155822069096)	0.99904062373978
**CAD13-HUMAN**	c(cor = -0.189308189637914)	0.99904062373978
**FCERG-HUMAN**	c(cor = -0.439897295282151)	0.99904062373978
**PTMA-HUMAN**	c(cor = 0.117028466698353)	0.99904062373978
**PLBL1-HUMAN**	c(cor = -0.111259085376643)	0.99904062373978
**S43A3-HUMAN**	c(cor = -0.161359610528418)	0.99904062373978
**CR1-HUMAN**	c(cor = 0.0650344342940954)	0.99904062373978
**S40A1-HUMAN**	c(cor = -0.202453279055798)	0.99904062373978
**MYG-HUMAN**	c(cor = 0.257995082024513)	0.99904062373978
**MRP4-HUMAN**	c(cor = -0.151397999522882)	0.99904062373978
**ARI2-HUMAN**	c(cor = -0.361373601664358)	0.99904062373978
**SNX3-HUMAN**	c(cor = -0.285827863994142)	0.99904062373978
**CIAO1-HUMAN**	c(cor = -0.22697316503749)	0.99904062373978
**EFHD2-HUMAN;EFHD1-HUMAN**	c(cor = -0.0115925240751714)	0.99904062373978
**RL7-HUMAN**	c(cor = 0.0337578099778495)	0.99904062373978
**MYH10-HUMAN**	c(cor = -0.29383357906321)	0.99904062373978
**REEP5-HUMAN**	c(cor = -0.0844526246642403)	0.99904062373978
**K1C9-HUMAN**	c(cor = -0.0834631744311981)	0.99904062373978
**COX41-HUMAN**	c(cor = -0.271043639600525)	0.99904062373978
**KRT35-HUMAN**	c(cor = 0.0428468411757835)	0.99904062373978
**ISK5-HUMAN**	c(cor = -0.219045389053453)	0.99904062373978
**C2C2L-HUMAN**	c(cor = 0.064333597443607)	0.99904062373978
**TPM2-HUMAN**	c(cor = -0.0598397599536449)	0.99904062373978
**K22E-HUMAN**	c(cor = -0.112151097475135)	0.99904062373978
**ITB1-HUMAN**	c(cor = -0.0811069108844359)	0.99904062373978
**KR111-HUMAN**	c(cor = -0.235990305981181)	0.99904062373978
**MPU1-HUMAN**	c(cor = -0.167109015728211)	0.99904062373978
**COX2-HUMAN**	c(cor = -0.173022386508497)	0.99904062373978

**IGHD-HUMAN**	c(cor = 0.0537139401382002)	0.99904062373978
**UTER-HUMAN**	c(cor = -0.177038505871611)	0.99904062373978
**GUAA-HUMAN**	c(cor = 0.0360526323087688)	0.99904062373978
**WFDC2-HUMAN**	c(cor = -0.359637288963737)	0.99904062373978
**AR6P1-HUMAN**	c(cor = 0.02156743851591)	0.99904062373978
**SBSN-HUMAN**	c(cor = 0.0666923029669058)	0.99904062373978
**CD177-HUMAN**	c(cor = -0.106539923288167)	0.99904062373978
**RDH11-HUMAN**	c(cor = -0.128962810211886)	0.99904062373978
